# Metal‐Free Arylation of Benzothiophenes at C4 by Activation as their Benzothiophene *S*‐Oxides

**DOI:** 10.1002/anie.202302418

**Published:** 2023-05-24

**Authors:** Ranjana Bisht, Mihai V. Popescu, Zhen He, Ameer M. Ibrahim, Giacomo E. M. Crisenza, Robert S. Paton, David J. Procter

**Affiliations:** ^1^ Department of Chemistry University of Manchester Oxford Road Manchester M13 9PL UK; ^2^ Department of Chemistry Colorado State University Center Ave Fort Collins CO 80523 USA

**Keywords:** Arylation, Benzothiophene, Phenol, Pummerer, Sulfoxide

## Abstract

Benzothiophenes, activated by oxidation to the corresponding *S*‐oxides, undergo C−H/C−H‐type coupling with phenols to give C4 arylation products. While an electron‐withdrawing group at C3 of the benzothiophene is important, the process operates without a directing group and a metal catalyst, thus rendering it compatible with sensitive functionalities—e.g. halides and formyl groups. Quantum chemical calculations suggest a formal stepwise mechanism involving heterolytic cleavage of an aryloxysulfur species to give a π‐complex of the corresponding benzothiophene and a phenoxonium cation. Subsequent addition of the phenoxonium cation to the C4 position of the benzothiophene is favored over the addition to C3; Fukui functions predict that the major regioisomer is formed at the more electron‐rich position between C3 and C4. Varied selective manipulation of the benzothiophene products showcase the synthetic utility of the metal‐free arylation process.

## Graphical abstract

Benzothiophenes are key structures in materials and medicines and methods for their selective C−H functionalization are highly prized. Activation of benzothiophenes bearing electron‐withdrawing groups at C3 as their *S*‐oxides enables formal C−H/C−H coupling with phenols to access C4 arylated benzothiophenes. Computational studies suggest the intermediacy of a π‐complex between the benzothiophene and a phenoxonium cation.

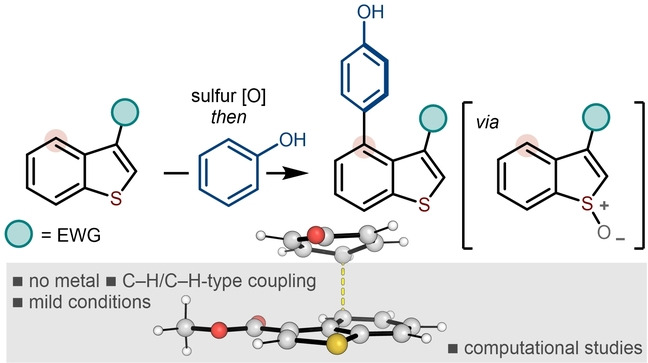

## Introduction

**Scheme 1:**
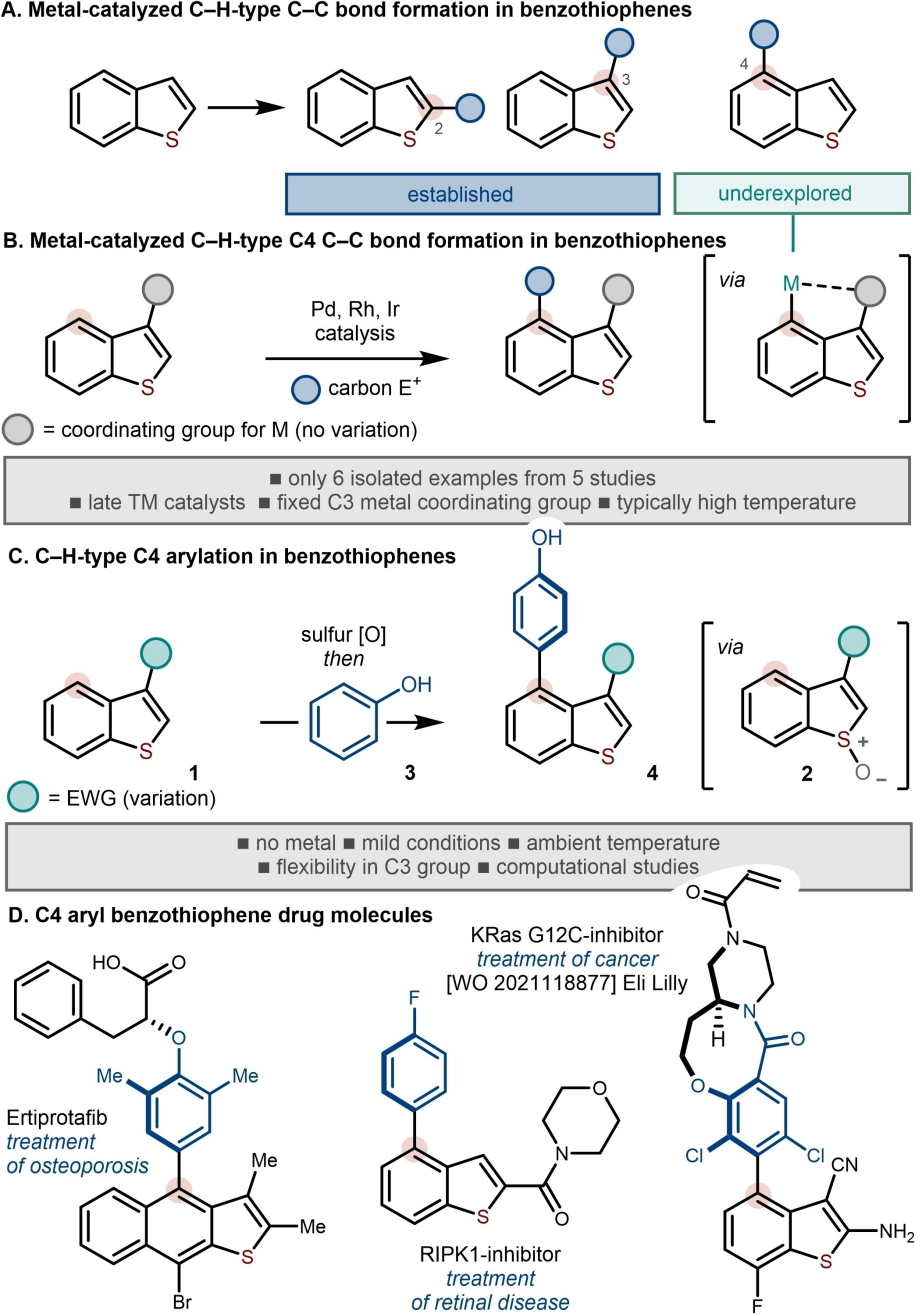
A) C−H‐type carbon–carbon bond formation in benzothiophenes; B) The few examples of direct C4 coupling of benziothiophenes require a specific metal coordinating group at C3; C) This work: Metal‐free C4 C−H‐type couplings of benzothiophenes—activated as their benzothiophene *S*‐oxides—with phenols. D) C4‐arylbenzothiophenes in drug discovery. TM, transition metal.

The benzothiophene heteroaromatic motif resides at the heart of natural products and drug molecules,^[1]^ and forms the template upon which many organic electronic materials are built.^[2]^ As such, methods for the decoration of the benzothiophene motif are highly sought after. In particular, the development of methods that allow benzothiophenes to be arylated or alkylated directly,^[3]^ without prefunctionalization, promises efficient routes to benzothiophenes for applications in medicine and materials science (Scheme 1A). While direct C3^[4]^ and C2^[5]^ couplings are now relatively established, C−H couplings at the C4 position of benzothiophenes are rare (Scheme 1B); only a handful of isolated examples have been reported and, in all cases, a defined coordinating substituent at C3 is required to direct metalation to C4.^[6]^ Furthermore, these isolated examples rely on the use of late transition metal catalysts (e.g. Rh and Ir). Of the reported examples, only one describes C4 C−H arylation and relies on the use of a palladium catalyst at 60 °C.^[6f]^ The above promising examples come from studies focused on indoles and no study dedicated to the development of a general process for the direct C4 arylation of benzothiophenes has been described.

In an attempt to develop metal‐free processes that complement transition metal‐catalyzed couplings, we recently reported the propargylation, allylation and arylation of benzothiophenes at C3 or C2 by exploiting straight‐forward activation of the parent benzothiophenes **1** as their *S*‐oxides **2**.[^7^, ^8^]

Herein, we report the development of a C4 selective C−H‐type coupling of benzothiophenes—activated as their *S*‐oxides—with phenols **3** that operates at low temperatures (Scheme 1C). Crucially, the strategy allows metal‐free access to functionalized C4‐arylbenzothiophenes **4** that are of value in drug discovery (Scheme 1D).^[1]^

## Results and Discussion

Optimization studies focused on the coupling of benzothiophene *S*‐oxide **2 a**—readily prepared from the parent benzothiophene in 85 % yield using H_2_O_2_/CF_3_CO_2_H/CH_2_Cl_2_—and 2‐bromophenol **3 a**, in the presence of trifluoroacetic anhydride (TFAA, Table 1). The order of addition proved to be important; addition of a solution of sulfoxide and phenol to TFAA was optimal (entry 3 versus entry 4). While increasing the equivalents of phenol and TFAA (entries 1–7) improved the yield—**3 a** was obtained in an NMR yield of 77 % using 3 equivalents of phenol—for complex phenol partners, lower equivalents would give acceptable yields. Finally, by keeping the reaction at −50 °C, it was clear that the reaction proceeds at that temperature, however, the rate of arylation is diminished (entry 8).^[9]^ In all entries, by‐product **5**—the product of C3 arylation—was formed in approx. 20 % NMR yield.

**Table 1:** Screening of Coupling Conditions.^[a]^

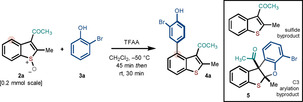				
entry	ArOH [equiv]	TFAA [equiv]	Yield of sulfide byproduct [%]	Yield of **4 a** [%]^[b]^
1	1.0	1.0	15	37
2	1.0	1.5	23	44
3	1.5	1.5	14	57
4^[c]^	1.5	1.5	8	46
5	2.0	1.5	11	52
6	2.5	1.5	7	69
7	3.0	1.5	3	77
8^[d]^	3.0	1.5	5	48

[a] Reaction conditions: A solution of sulfoxide **2 a** (1 equiv, 0.2 mmol) and phenol **3 a** in CH_2_Cl_2_ was added to a solution of TFAA in CH_2_Cl_2_ at −50 °C. After 45 min, the reaction was warmed to room temperature and stirred for 30 min. [b] Yield determined by ^1^H NMR using nitromethane as internal standard. [c] Alternative order of addition: TFAA added to a solution of sulfoxide **2 a** and phenol **3 a** in CH_2_Cl_2_ at −50 °C. [d] Reaction kept at −50 °C and quenched at −50 °C after 2 h. Could you please add additional twitter handles? Please see below: @bobbypaton @MihaiPopescu94 @csu chemistry

**Scheme 2:**
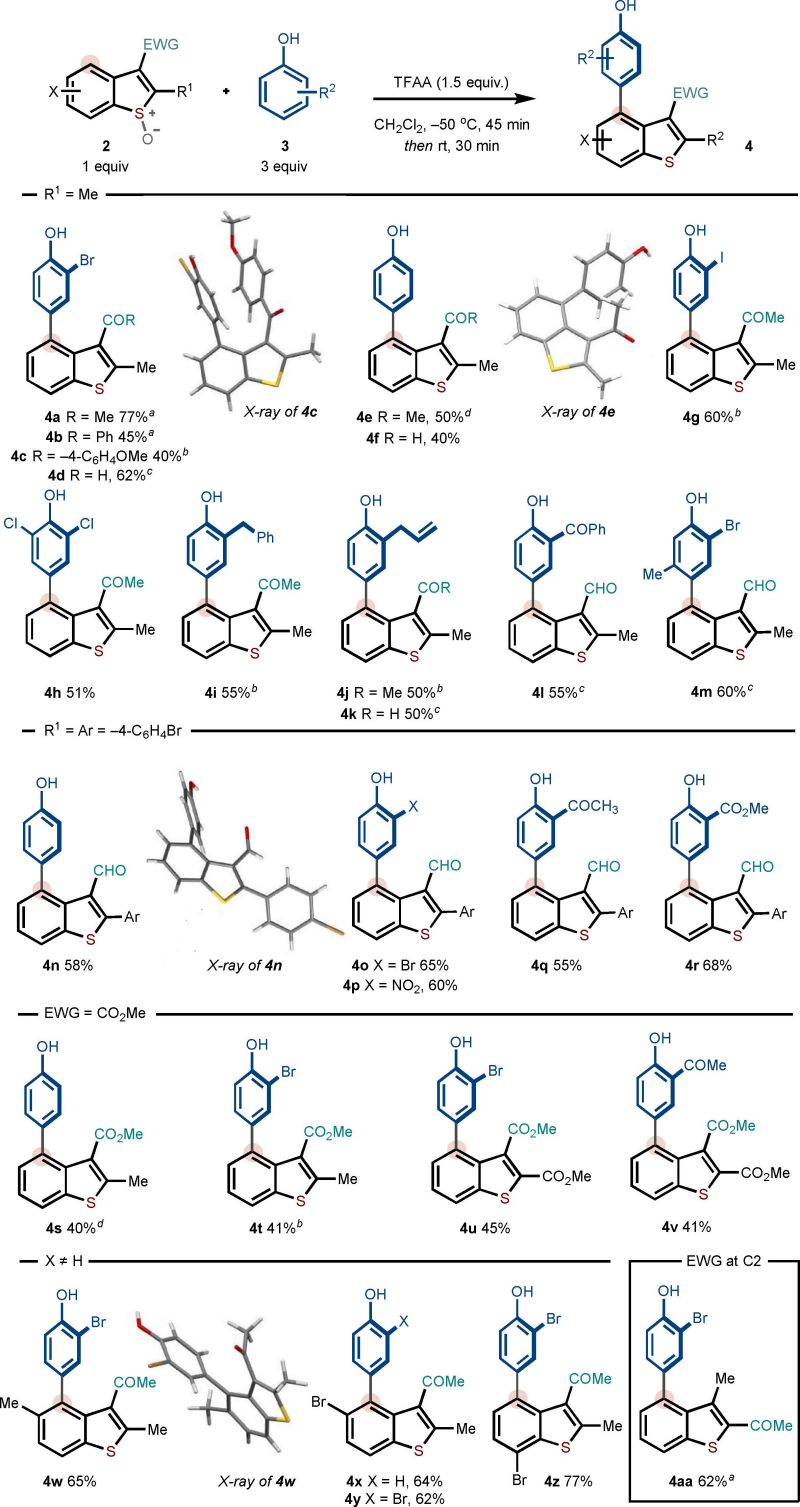
Scope with respect to C2, C3 disubstituted benzothiophene systems. Reaction conditions: **2** (1 equiv, 0.2 mmol), **3** (3 equiv), TFAA (1.5 equiv), in CH_2_Cl_2_.^[a]^ 10–11 % C3 coupling product isolated.^[b]^ 14–16 % C3 coupling product isolated.^[c]^ **3** (5 equiv), TFAA (3 equiv)^[d]^ 31–38 % C3 coupling product isolated. The amount of isolated C3 coupling product for each entry can be found in the Supporting Information. For entries without a footnote, the C3 arylated product was not observed.

The scope of the reaction for 2,3‐disubstituted benzothiophenes, bearing an electron‐withdrawing group at C3 was assessed (Scheme 2). Benzothiophenes bearing electron‐withdrawing keto, formyl or ester groups at C3, and methyl, aryl and ester groups at C2, underwent cross‐coupling with a range of *ortho* and *meta*‐substituted phenols to give products **4 a**–**4 z** in 40–77 % yield. The most significant byproduct was typically the corresponding product of C3 arylation, although for many substrates the coupling was completely selective. Under the metal‐free conditions of the coupling, the presence of carbon‐halogen bonds in either partner was tolerated thus allowing access to products containing chloro (**4 h**), bromo (**4 a**–**4 d**, **4 m**, **4 o**, **4 t**–**4 u**, **4 w**–**4 z**), and iodo (**4 g**) functionality. In addition, benzyl (**4 i**), allyl (**4 j**, **4 k**), keto (**4 l**, **4 q**, **4 v**), ester (**4 r**), and nitro (**4 p**) groups in the phenol partner were also compatible. Substitution on the benzenoid ring of the benzothiophene ring system was possible (**4 w**–**4 z**). In several cases, the structure of the product was confirmed by X‐ray crystallography (**4 c**, **4 e**, **4 n**, **4 w**) thus confirming the regioselectivity of the coupling.^[9]^

Finally, swapping the C3 and C2 substituents also gave a substrate that underwent effective coupling (**4 aa**); however, the coupling of substrates bearing an electron‐drawing group at C2 proved to be less general than that of the analogous C3 substrates (Scheme 2). In all cases, *para*‐coupling of the phenol partner was observed with no trace of *ortho*‐coupling. For the examples in Scheme 2, the benzothiophene *S*‐oxides were typically obtained in 80–96 % yield. For the S‐oxidation of 3‐formyl benzothiophenes, low conversion was used to ensure selective oxidation and avoid Baeyer–Villiger side‐reactivity of the aldehyde. Unreacted benzothiophenes were easily recovered and reused. Crucially, 2,3‐dimethylbenzothiophene *S*‐oxide gave a product of C3 coupling in 65 % (see Section 5.2 of the Supporting Information) thus underlining the important role of the electron‐withdrawing group at C3.

**Scheme 3:**
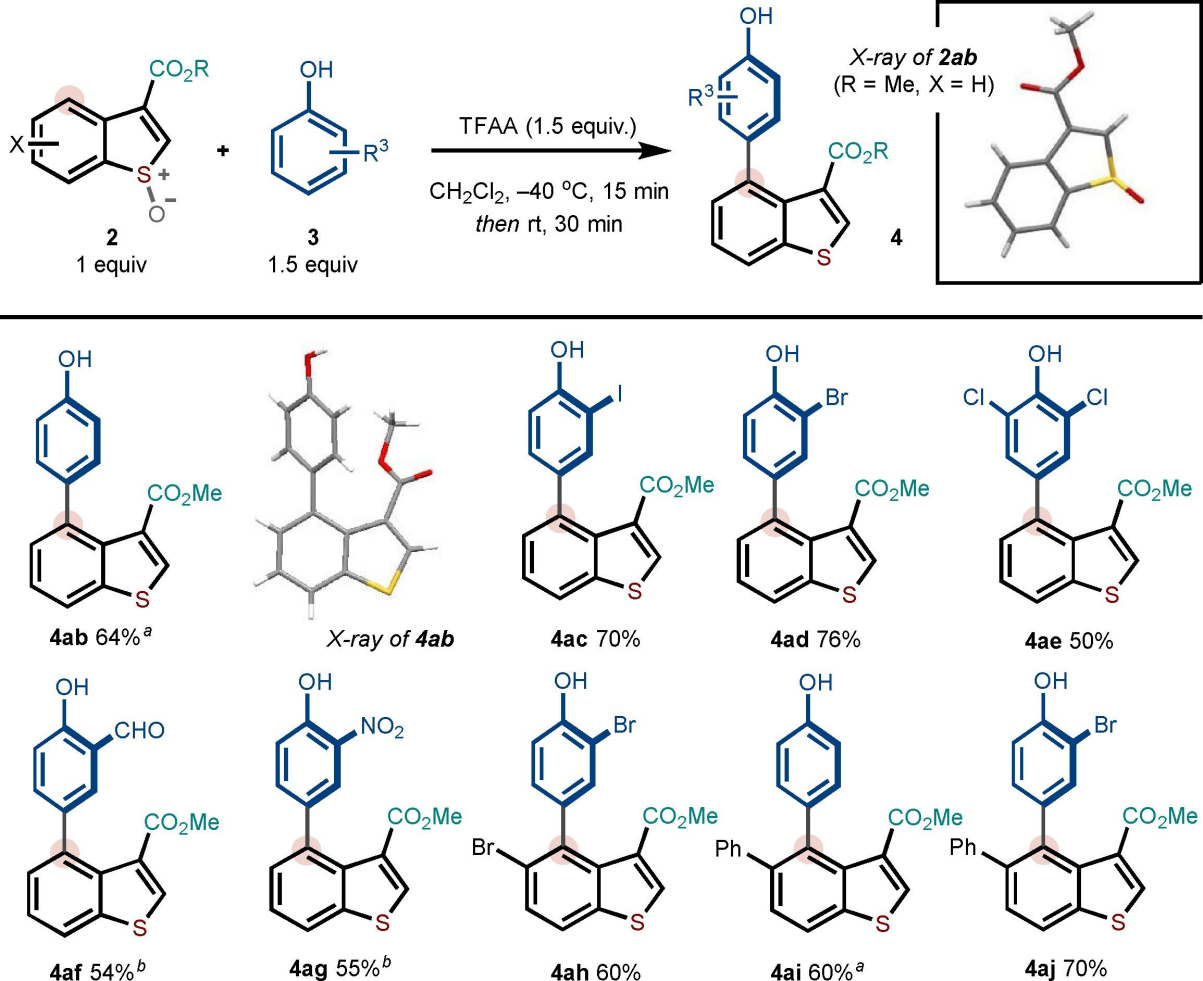
Scope with respect to C3 monosubstituted benzothiophene systems. Reaction conditions: **2** (1 equiv, 0.2 mmol), **3** (1.5 equiv), TFAA (1.5 equiv), in CH_2_Cl_2_.^[a]^ 5–7 % C3 coupling observed.^[b]^ 12–15 % C3 coupling observed. The amount of isolated C3 coupling product for each entry can be found in the Supporting Information. For entries without a footnote, the C3 arylated product was not observed.

C2 Substitution is not required for the selective metal‐free C4 arylation process. In particular, C3 ester benzothiophenes—with no substitution at C2—underwent effective coupling with a range of phenol partners under slightly modified conditions (phenol partner [1.5 equiv], TFAA [1.5 equiv]; see Supporting Information for additional optimization details) to give the desired products of direct arylation in 50–76 % yield. Again, the functional group compatibility of the process was highlighted through the use of phenols containing iodo, bromo, chloro, formyl and nitro groups, and a bromo‐substituted benzothiophene (to give **4 ah**) (Scheme 3). Again, *para*‐coupling of the phenol partner was observed with no trace of *ortho*‐coupling. For the examples in Scheme 3, the benzothiophene *S*‐oxides were obtained in 50–78 % yield.

**Scheme 4:**
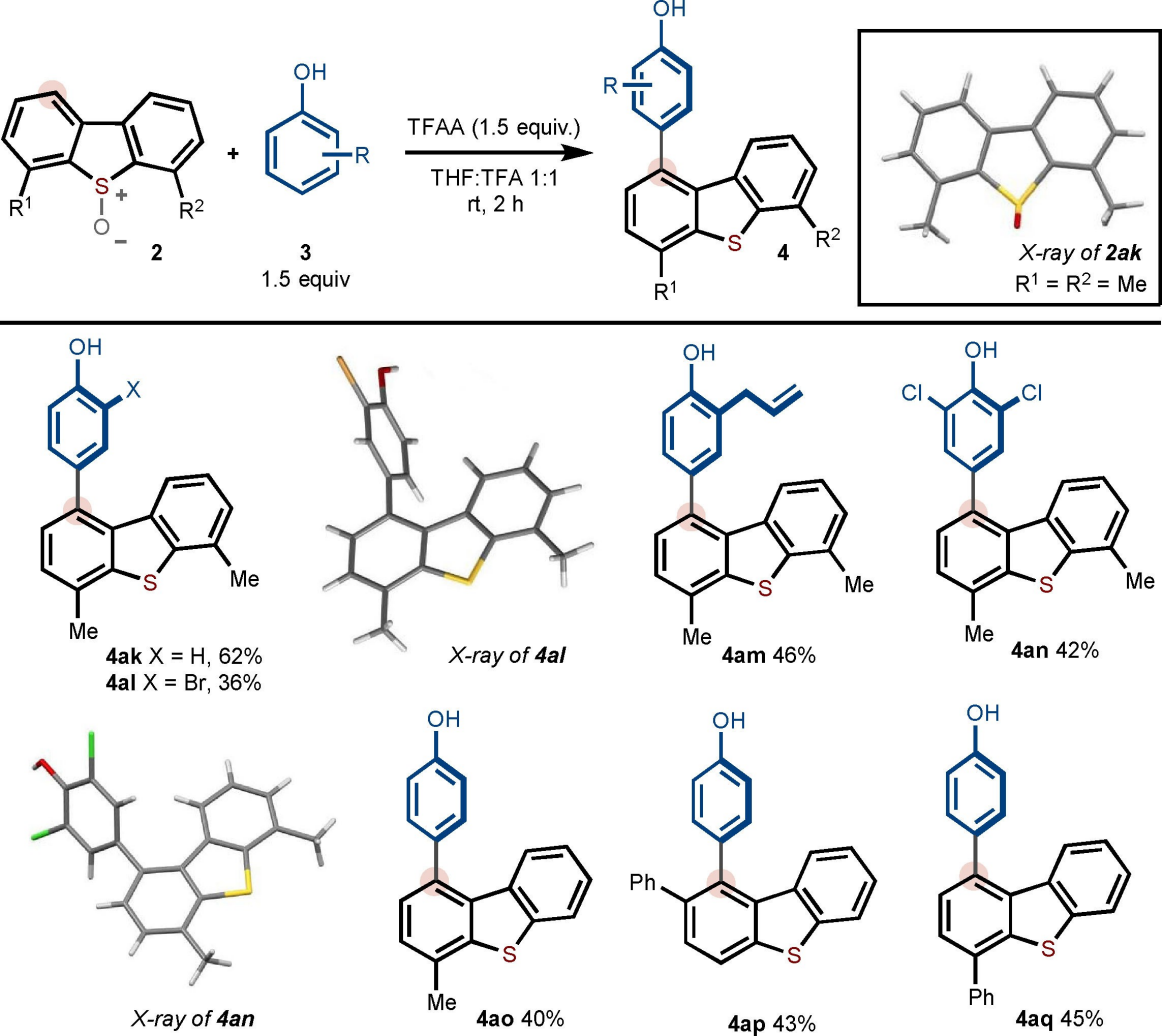
Scope with respect to dibenzothiophene systems. Reaction conditions: **2** (1 equiv, 0.2 mmol), **3** (1.5 equiv), TFAA (3.0 equiv), in THF/TFA.

No product **4 ak** was observed when applying our optimized conditions (Schemes 2 and 3) to the metal‐free arylation of 4,6‐dimethyldibenzthiophene, conveniently activated as its *S*‐oxide. However, switching from CH_2_Cl_2_ to a THF/TFA solvent mix resulted in effective coupling to give **4 ak** in 64 % yield (Scheme 4). In addition to selective arylation of symmetrical dibenzothiophenes (**4 ak**–**4 an**), unsymmetrical dibenzothiophenes underwent selective coupling with arylation taking place on the more electron‐rich substituted ring (**4 ao**–**4 aq**), which is in line with our mechanistic proposal (vide infra). *para*‐Coupling of the phenol partner was exclusively observed. For the examples in Scheme 4, the benzothiophene *S*‐oxides were obtained in 87–92 % yield.

**Scheme 5:**
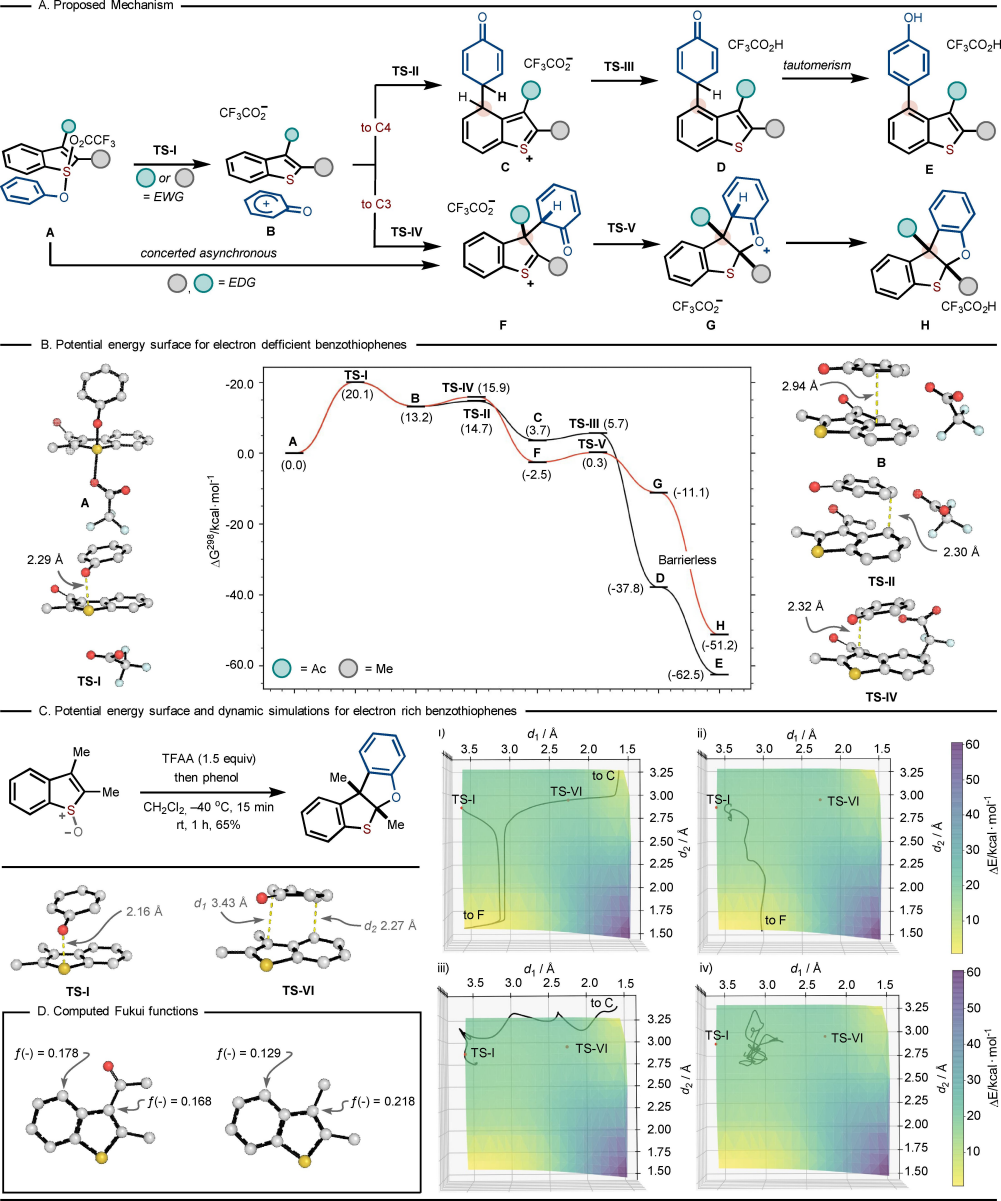
Computational studies. A) Proposed reaction mechanism; B) Potential energy surface of an electron‐deficient benzothiophene computed at the M06‐2X‐D3/Def2‐QZVPP(SMD=CH_2_Cl_2_)//M06‐2X‐D3/6‐31+G(d,p)(SMD=CH_2_Cl_2_) level of theory; C) Two‐dimensional potential energy surface of an electron‐rich benzothiophene computed at the M06‐2X‐D3/6‐31+G(d,p)(SMD=CH_2_Cl_2_), together with i) Intrinsic Reaction Coordinates (IRCs) for **TS‐I** and **TS‐IV**, ii) representative dynamic trajectory leading to product **F**, iii) representative trajectory leading to product **C** and iv) representative trajectory that does not lead to product formation; D) Atom‐condensed Fukui functions (ƒ(−)) for two substrates giving contrasting regioselectivities.

To further understand the mechanism and factors dictating reaction outcome and regioselectivity, the rearrangements and associated transition structures (TSs) of several benzothiophene *S*‐oxides were studied using density functional theory (DFT) with the M06‐2X‐D3/def2‐QZVPP (SMD=CH_2_Cl_2_)//M06‐2X‐D3/6‐31+G(d,p) (SMD=CH_2_Cl_2_) level of theory (Scheme 5).^[10]^ In line with previous studies from our group[^7a^, ^7b^, ^8e^] and from others[^8c^, ^8d^, ^8h^, ^8i^, ^11^] we propose a mechanism involving initial activation of the sulfoxide moiety in benzothiophene *S*‐oxides as the corresponding trifluoroacetate followed by addition of phenol to give aryloxysulfonium salt or sulfurane **A**; use of anisole in place of phenol gave no reaction (see Supporting Information) thus underlining the importance of the free hydroxyl group in the phenol coupling partners.

For the coupling of **2 a** and phenol, we computed Gibbs energy profiles for the competing regioisomeric pathways leading to 3‐ and 4‐functionalized products **H** and **E**, respectively (Scheme 5B). Although potential concerted [3,3]‐ and [5,5]‐sigmatropic rearrangements were explored, the computed potential energy surface (PES) instead reveals a stepwise process, in which the activated benzothiophene *S*‐oxide **A** first undergoes heterolytic cleavage of both S−O bonds via **TS‐I** (Δ*G* ^≠^ 20.1 kcal mol^−1^) to form intermediate **B** in an endergonic step (Δ*G* 13.2 kcal mol^−1^). In intermediate **B**, the benzothiophene and phenoxonium aromatic rings are closely π‐stacked with a parallel‐displaced geometry (with a perpendicular distance of 3.0 Å between the rings). Most of the positive charge in this cationic complex resides on the phenoxonium fragment (+0.64 e), indicating stabilizing charge transfer from benzothiophene. From this point on, the trifluoroacetate counteranion interacts weakly with the substrate: the PESs computed in the presence (Scheme 5), and absence (see Supporting Information) of this counteranion are qualitatively and quantitatively unchanged, and there is no influence on the computed regioselectivity. Intermediate **B** can undergo recombination at the 4‐position or 3‐position of the benzothiophene heterocycle via C−C bond‐forming **TS‐II** or **TS‐IV**, respectively. Reactivity via **TS‐II** was found to be preferred by 1.2 kcal mol^−1^, which agrees with the experimentally determined product distribution favoring the 4‐position (**E : H**–1.7 : 1 at rt). Based on our computed PES, we propose that C−C formation is the regiodetermining step, while the formation of intermediate **B** is rate‐determining.^[12]^ An interesting feature of this region of the PES is that, while **B** exists as a local potential energy minimum, the barriers for ensuing C−C bond‐forming are relatively small (1.5 and 2.7 kcal mol^−1^). Thus, we expect **B** to be short‐lived, potentially preventing equilibration of atomic motions and the surrounding solvent.^[13]^ While we have been able to reproduce the experimental sense and trends in regioselectivity by applying transition‐state theory (TST), we also investigated the relevance of dynamic effects in this transformation, performing quasi‐classical molecular dynamics trajectories in the region of this intermediate (see below).^[14]^

Upon C−C bond‐formation at C4 via **TS‐II**, the resulting intermediate **C** can undergo facile (Δ*G* ^≠^ 2.0 kcal mol^−1^) and irreversible (i.e., highly exergonic by 41.5 kcal mol^−1^) rearomatization of the benzothiophene moiety (via **TS‐III**), leading to the formation of intermediate **D**. Subsequent tautomerization to the experimentally isolated major product **E** is then highly favorable by 24.7 kcal mol^−1^. On the other hand, the competing functionalization at the C3 position via **TS‐IV** leads to the formation of thiocarbenium ion **F**, which can readily undergo an intramolecular cyclization via **TS‐V** (Δ*G* ^≠^=2.7 kcal mol^−1^) to form tetracyclic intermediate **G**. **G** was found to undergo barrierless deprotonation (see Supporting Information) by the trifluoroacetate counterion, forming the minor C3‐arylated product **H**.

To explain the importance of the electron‐withdrawing group at C3 upon the product distribution, we analyzed the electron distribution and local reactivity descriptors of various benzothiophenes. Conceptually, consistent with the computed charge distribution in intermediate **B**, the formation of C4‐ or C3‐adducts can be interpreted as competing electrophilic aromatic substitutions (EAS) of the relatively electron‐rich benzothiophene with a phenoxonium cation. Consequently, the more electron‐rich positions on the benzothiophene should favor reactivity at that site, as described by atom‐condensed Fukui ƒ^−^ functions (see Supporting Information).^[15]^ The largest ƒ^−^ values occur at the C4‐position for benzothiophenes with an electron‐withdrawing group (−CHO, −COMe, −CO_2_Me) positioned at C3. In contrast, with a weakly‐donating methyl group at C3, the largest ƒ^−^ value is found at the C3‐position (Scheme 5D). These observations are consistent with the observed regiochemistry in both cases: this is therefore influenced by the difference in benzothiophene C3 and C4 nucleophilicity induced by differing C3‐substituents.

The Gibbs energy profile of 2,3‐dimethylbenzothiophene *S*‐oxide was further investigated (Scheme 5C). Experimentally this substrate leads to exclusive C3‐arylation: computationally, a qualitatively different PES is obtained compared to the 3‐acyl analogue. For this more electron‐rich substrate, the evolution of **TS‐I** (S−O bond breaking) along the intrinsic reaction coordinate (IRC) led to the direct formation of thiocarbenium ion **F** in a concerted asynchronous manner, bypassing intermediate π‐adduct **B** as a stable reaction local minimum (i.e. a hidden intermediate). This is consistent with the experimentally observed outcome, where the C3‐arylated product was isolated as the sole reaction product in 65 % yield. Attempts at finding equivalent **TS‐II** and **TS‐IV** points, as previously identified for the electron‐deficient benzothiophene system, proved unfruitful. However, we were able to identify a new transition state **TS‐VI**, which formally corresponds to a [3,3]‐sigmatropic rearrangement between intermediates **C** and **F**. After **TS‐I** the PES enters a relatively flat region, which led us to consider the possibility of non‐intrinsic reaction coordinate (non‐IRC) pathways (Scheme 5C).^[16]^ We therefore initiated 100 quasi‐classical molecular dynamics trajectories in the region of **TS‐I** (see Supporting Information, Section 8.4 for computational detail), which revealed the reaction to occur in a dynamically stepwise fashion. While most of the reactive trajectories (39) led to formation of the IRC‐expected intermediate **F**, we found a small number (6) resulted in non‐IRC intermediate **C**. Further, half of all trajectories (50) remained in the PES region associated with a π‐stacked benzothiophene—phenoxonium species and did not exit after 500 fs of simulation time. These results suggest that although **B** is not an energy minimum for this substrate (unlike those bearing C3 electron‐withdrawing groups), it does play a role as an “entropic intermediate” with an average lifetime in excess of several hundred fs. As such, formation of the C3 arylated product may also be considered dynamically stepwise.^[17]^

**Scheme 6:**
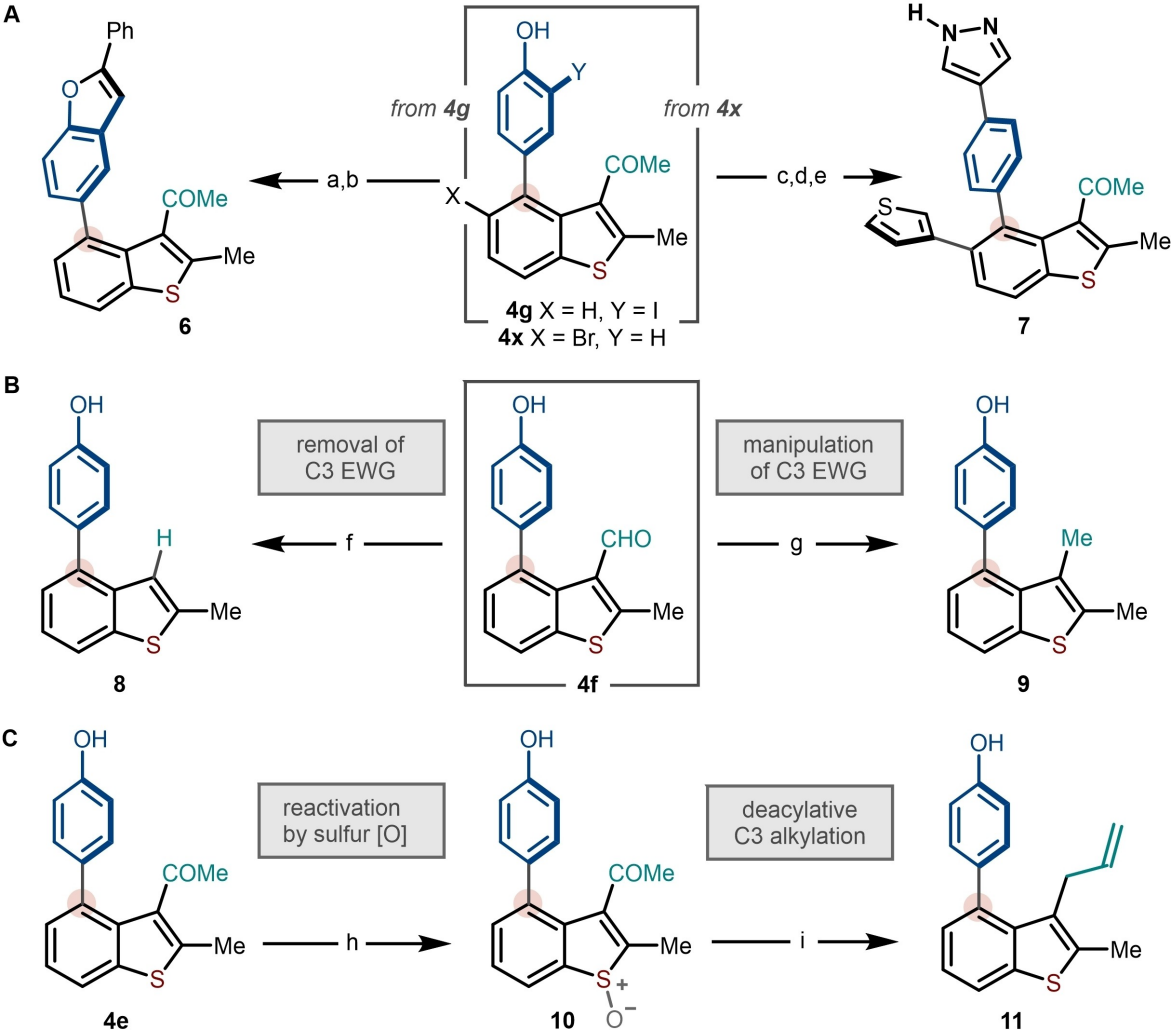
Selective manipulation of the products of C4‐arylation. a) styrene (1.3 equiv), Pd(OAc)_2_ (10 mol %), PPh_3_ (4 mol %), Et_3_N, 80 °C, 9 h, 60 %. b) K_2_CO_3_ (5.6 equiv), I_2_ (5.6 equiv), THF, rt, 5 h, 50 % yield. c) 3‐thienylboronic acid (1.1 equiv), Pd(PPh_3_)_4_ (5 mol %), Na_2_CO_3_ (4 equiv), PhMe:H_2_O:MeOH, 100 °C, 8 h, 81 % yield. d) Tf_2_O (1.5 equiv), Et_3_N (2 equiv), CH_2_Cl_2_, 0 °C to rt, 8 h. e) 1*H*‐pyrazole‐4‐boronic acid (1.2 equiv), Pd(PPh_3_)_4_ (10 mol %), 2 M *aq*. Na_2_CO_3_, PhMe‐EtOH, 100 °C, 12 h, 80 % (over two steps). f) Pd(OAc)_2_ (32 mol %), 3 Å MS, cyclohexane, 140 °C, 24 h, 55 %. g) N_2_H_4_⋅H_2_O (1.15 equiv), KOH (1.03 equiv), ethylene glycol, 130 °C, 4 h, 70 %. h) H_2_O_2_ (1 equiv), TFA:CH_2_Cl_2_, rt, 4 h. i) allyltrimethylsilane (1.5 equiv), TFAA (1.5 equiv), MeCN, 0 °C to 80 °C, 3 h, 60 % (over two steps).

Finally, synthetic manipulation of the C4 arylated benzothiophenes was explored. The metal‐free nature of the arylation allows transition metal‐sensitive halide and hydroxyl functionalities to be incorporated in products **4**; these serve as strategic handles for the formation of poly‐heteroaromatic systems (Scheme 6A). For example, the 2‐iodophenol motif in product **4 g** was readily converted to the benzofuran ring of **6**, through a sequential Pd‐catalyzed Heck reaction with styrene, followed by an iodine‐mediated heterocyclization.^[18]^ The phenol moiety in the C4 aryl unit can alternatively be employed as an electrophilic handle for cross‐coupling. First, bromo‐analogue **4 x** was coupled with 3‐thienylboronic acid to access the corresponding C5‐thienyl derivative. Conversion to the triflate allowed facile Pd‐catalyzed Suzuki–Miyaura coupling with 1*H*‐pyrazole‐4‐boronic acid to provide tetracyclic heteroarene **7**. While an electron‐withdrawing group on the benzothiophene ring is needed, selective modification of the C3‐substituent allows access to an even wider range of benzothiophene scaffolds. For example, the formyl moiety in **4 f** can be either removed—by Pd‐catalyzed deformylation^[19]^—leading to C3‐H derivative **8**, or reduced to methyl benzothiophene **9**, under Wolff–Kishner conditions (Scheme 6B). In the case of keto‐derivative **4 e**, the benzothiophene core can be “re‐activated” by oxidation to *S*‐oxide **10**, which undergoes deacylative C3‐allylation in the presence of allyltrimethylsilane to give **11** via the interrupted Pummerer/[3,3]‐sigmatropic rearrangement cascade developed by our group^[7a]^ (Scheme 6C). These studies highlight the synthetic versatility of the C4 arylated benzothiophene products and their viability as intermediates en route to complex polyaromatic materials and bioactive scaffolds (c.f. Scheme 1D).

## Conclusion

In summary, straight‐forward oxidation of benzothiophenes to the corresponding benzothiophene *S*‐oxides allows formal C−H/C−H‐type coupling with phenols to give C4 arylated benzothiophene products. In contrast to the scarce literature precedent for the direct C4 arylation of benzothiophenes—which requires the use of late transition metals and specific C3 directing groups—our method operates under metal‐free conditions and simply requires an electron‐withdrawing group at C3 of the benzothiophene. Crucially, the protocol is compatible with functionalities—e.g. carbon‐halogen bonds and formyl groups—that do not tolerate metal catalysts. Computational studies suggest a mechanism involving heterolytic cleavage of an aryloxysulfur species to give a benzothiophene and a phenoxonium cation that combine preferentially at the C4 position. The synthetic utility of the metal‐free, direct arylation process has been underlined by selective manipulation of the versatile benzothiophene products.

## Conflict of interest

The authors declare no conflict of interest.

1

## Supporting information

As a service to our authors and readers, this journal provides supporting information supplied by the authors. Such materials are peer reviewed and may be re‐organized for online delivery, but are not copy‐edited or typeset. Technical support issues arising from supporting information (other than missing files) should be addressed to the authors.

Supporting Information

## Data Availability

The data that support the findings of this study are available in the Supporting Information of this article.
