# Supplementary material for: Metal‐Free Arylation of Benzothiophenes at C4 by Activation as their Benzothiophene S‐Oxides
Source: Angew Chem Int Ed Engl. 2023 May 24;62(29):e202302418. doi: 10.1002/anie.202302418 (PMC10953450; doi:10.1002/anie.202302418)

Angewandte Chemie
Eine Zeitschrift der Gesellschaft Deutscher Chemiker

Supporting Information

Metal-Free Arylation of Benzothiophenes at C4 by Activation as their Benzothiophene *S*-Oxides

R. Bisht, M. V. Popescu, Z. He, A. M. Ibrahim, G. E. M. Crisenza, R. S. Paton, D. J. Procter**

Table of Contents

1. General Experimental S3

2. General Procedures

2.1 General Procedure A: Acetylation of substituted benzothiophenes........ S4

2.2 General procedure B: Synthesis of substituted 2-methylbenzothiophenes........ S4

2.3 General Procedure C: Synthesis of 2-methyl 3-aroylbenzothiophenes........ S4

2.4 General Procedure D: Synthesis of methyl benzothiophene-3-carboxylates........ S4

2.5 General Procedure E: Oxidation of benzothiophenes to benzothiophene *S*-oxides using H_2O_2/TFA........ S5

2.6 General Procedure F: Oxidation of benzothiophenes to benzothiophene *S*-oxides using *m*-CPBA/$BF_3 \cdot OEt_2$........ S5

2.7 General Procedure G: C4 C–H Arylation of 2-substituted benzothiophene *S*-oxides........ S6

2.8 General Procedure H: C4 C–H Arylation of 2-unsubstituted benzothiophene *S*-oxides........ S6

2.9 General Procedure I: C4 C–H Arylation of dibenzothiophene *S*-oxides........ S7

3. Optimization of the C–H type C4-arylation of benzothiophenes........ S7

4. Compound characterization data........ S9

5. Control experiments........ S48

6. Synthetic transformations of the products of C4 C–H arylation........ S49

7. X-Ray structures........ S55

8. DFT studies........ S75

9. References........ S153

10. NMR spectra........ S156

General Experimental

All experiments were performed under an atmosphere of nitrogen, using anhydrous solvents, unless stated otherwise. All other solvents and reagents were purchased from commercial sources and used as supplied. ^{1}H NMR spectra were recorded on NMR spectrometers at 400 MHz and 500 MHz and ^{13}C NMR at 101 MHz and 126 MHz. ^{1}H NMR chemical shifts (δ_H) and ^{13}C NMR chemical shifts (δ_C) are quoted in parts per million (ppm) downfield from trimethylsilane (TMS) and coupling constants (*J*) are quoted in Hertz (Hz). Splitting patterns are assigned s = singlet, d = doublet, t = triplet, q = quartet, dd = doublet of doublets, td = triplet of doublets, dt = doublet of triplets, ddt = doublet of doublet of triplets, m = multiplet and br. = broad. Infrared (IR) spectra were recorded on an FTIR spectrometer and mass spectra were obtained using positive or negative electrospray ionisation (ESI), atmospheric pressure chemical ionization (APCI) techniques. Column chromatography was carried out using silica gel 60 Angstrom (Å), 240-400 mesh. Routine TLC analysis was carried out on aluminium sheets coated with silica gel 60 F254, 0.2 mm thickness. Plates were viewed under a 254 nm UV lamp or visualised by staining with potassium permanganate, p-anisaldehyde, or vanillin followed by heating.

Substrate Synthesis

General Method A: Acetylation of substituted benzothiophenes

Substituted benzothiophene (8.5 mmol, 1 equiv) and acetyl chloride (913 μL, 12.8 mmol, 1.5 equiv) were dissolved in dry benzene (80 mL) and the mixture was cooled to 5 °C. Then, a solution of $SnCl_4$ (1.5 mL, 1.5 equiv, 12.8 mmol) in dry benzene (12 mL) was added dropwise over 30 min. The reaction mixture was stirred at room temperature for 3 h and acidified with cold 10% aqueous HCl (50 mL). The organic layer was separated, dried over $MgSO_4$, and concentrated *in vacuo*. The crude product was purified by column chromatography.

General Method B: Synthesis of substituted 2-methylbenzothiophenes

To a solution of benzothiophene (3.00 mmol) in THF (15 mL) was added dropwise *n*-BuLi in hexane (1.6 M, 2.3 mL, 3.59 mmol) at −78 ^{o}C. After stirring for 30 min, iodomethane (928 μL, 14.9 mmol) was added to the reaction mixture at −78 ^{o}C. After stirring at room temperature for 2 h, the reaction mixture was quenched with saturated aqueous NH_4Cl (7 mL) and extracted with Et_2O (2 x 20 mL). The combined organic layer was washed with brine, dried over $MgSO_4$, and concentrated *in vacuo*. The material was used without further purification.

General Method C: Synthesis of 2-methyl 3-aroylbenzothiophenes

In an oven-dried N_2-flushed vial, 2-methylbenzothiophene (3 equiv, 4.5 mmol) was dissolved in HFIP (2.0 mL), and acid chloride (1 equiv, 1.5 mmol) was then added. The resultant mixture was stirred at room temperature for 5 h. The reaction mixture was concentrated and purified by column chromatography.

General Method D: Synthesis of methyl benzothiophene-3-carboxylates

Benzo[*b*]thiophene-3-carboxylic acid (10 mmol, 1 equiv) was dissolved in *N*,*N*-dimethylformamide (20 mL) and K_2CO_3 (2 equiv) was then added. The mixture was stirred at room temperature for 15 min and MeI (2 equiv) was added. After stirring overnight at the same temperature, the reaction mixture was poured into cold water. The aqueous phase was extracted with EtOAc (2 x 20 mL). The organic layer was washed with H_2O, brine, dried over $MgSO_4$, and concentrated *in vacuo*. The crude mixture was purified by column chromatography.

General Procedure E: Oxidation of benzothiophenes to benzothiophene *S*-oxides using H_2O_2/TFA

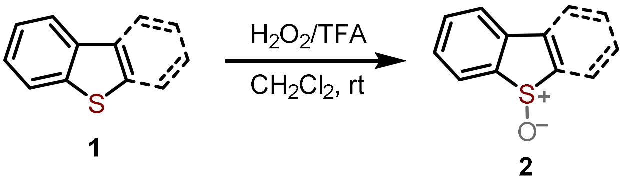

To an oven-dried vial under nitrogen was added the benzothiophene (1.0 mmol), CH_2Cl_2 (2.2 mL), and TFA (2.2 mL). H_2O_2 (50% aq., 57 μL, 1.0 mmol) was added to the solution at room temperature and the reaction was monitored by TLC (10% EtOAc in CH_2Cl_2). More H_2O_2 was added until complete consumption of the starting material was observed. The reaction was then quenched with saturated aqueous $NaHCO_3$ at 0 °C and the aqueous phase was extracted with CH_2Cl_2 (2 x 5 mL). The combined organic layers were dried with $MgSO_4$ and concentrated *in vacuo*. The crude mixture was purified by column chromatography (20% EtOAc in CH_2Cl_2) to give the benzothiophene *S*-oxide.

General Procedure F. Oxidation of benzothiophenes to benzothiophene *S*-oxides using *m*-CPBA/BF_3·OEt_2

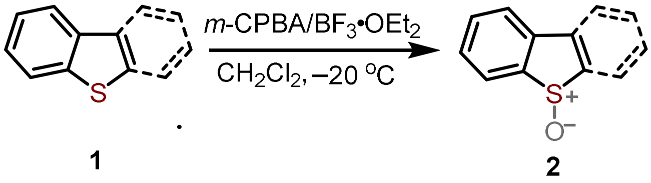

The benzothiophene (1 mmol) was dissolved in CH_2Cl_2 (0.5 M) in an oven-dried tube flushed with N_2 at −20 °C and BF_3·OEt_2 (1 mL, 8 equiv) was added. *m*-CPBA (269 mg, 1.2 equiv) in CH_2Cl_2 (0.25 M) was then added in two portions over 4 h at the same temperature. The reaction was monitored by TLC, and after the disappearance of the starting material, saturated Na_2CO_3 was added to the mixture, followed by K_2CO_3 at −20 °C. The mixture was then filtered through a plug of $MgSO_4$ and K_2CO_3, and washed with CH_2Cl_2. The combined organic layers were dried with $MgSO_4$ and concentrated *in vacuo*. The crude product was recrystallized from hexane and CH_2Cl_2.

General Procedure G: C4 C-H Arylation of 2-substituted benzothiophene *S*-oxides

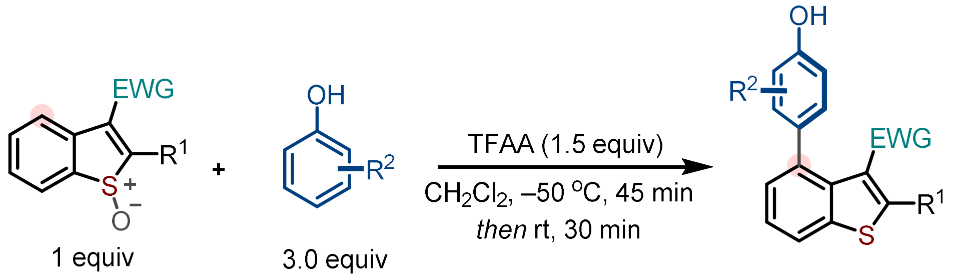

In an oven-dried tube, TFAA (1.5 equiv) in CH_2Cl_2 (0.5 mL) was cooled to −50 °C. Sulfoxide (1.0 equiv) and phenol (3.0 equiv) were dissolved in CH_2Cl_2 (1.5 mL) and the solution added to the TFAA solution dropwise. After 45 min at −50 °C, the mixture was warmed to room temperature and stirred for 30 min. Saturated aqueous $NaHCO_3$ was then added and the aqueous phase was extracted with CH_2Cl_2 (3 × 3 mL). The combined organic layers were dried over $MgSO_4$ and concentrated *in vacuo*. The crude product was purified by column chromatography on silica gel eluting with *n*-hexane in EtOAc.

General Procedure H. C4 C-H Arylation of 2-unsubstituted benzothiophene *S*-oxides

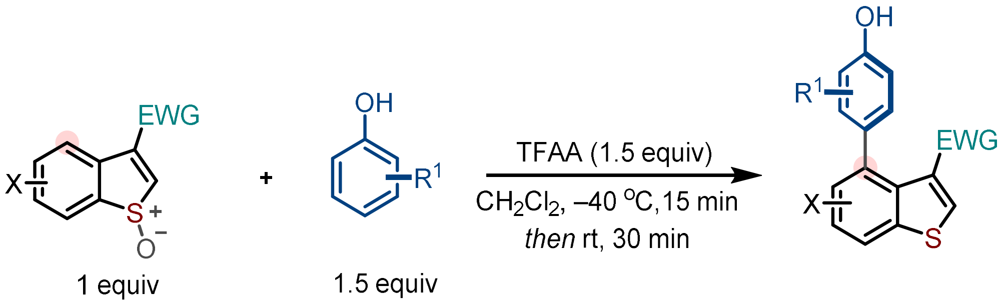

Sulfoxide (1 equiv) and phenol (1.5 equiv) were dissolved in CH_2Cl_2 (1 mL) in an oven-dried tube. TFAA (1.5 equiv) was then added at −40 °C. After 15 min at −40 °C, the mixture was warmed to room temperature and stirred for 2 h. Saturated aqueous $NaHCO_3$ was then added and the aqueous phase was extracted with CH_2Cl_2 (3 × 3 mL). The combined organic layers were dried over $MgSO_4$ and concentrated *in vacuo*. The crude product was purified by column chromatography on silica gel eluting with *n*-hexane in EtOAc.

General Procedure I. C4 C-H Arylation of dibenzothiophene *S*-oxides

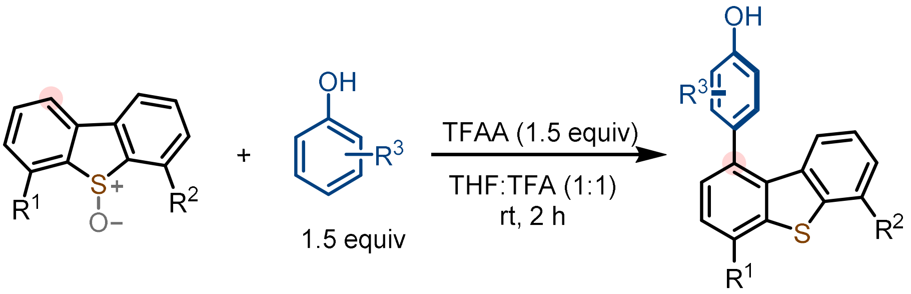

Sulfoxide (1 equiv) and phenol (1.5 equiv) were dissolved in THF/TFA (0.7/0.7 mL) in an oven-dried tube. TFAA (1.5 equiv) was then added at room temperature. The mixture was stirred for 2 h. Saturated aqueous $NaHCO_3$ was then added and the aqueous phase was extracted with CH_2Cl_2 (3 × 3 mL). The combined organic layers were dried over $MgSO_4$ and concentrated *in vacuo*. The crude product was purified by column chromatography on silica gel eluting with *n*-hexane in EtOAc.

Optimization of the C–H type C4-arylation in benzothiophenes

All optimization reactions were carried out on a 0.20 mmol scale. Optimization studies focused on the coupling of benzothiophene *S*-oxide **2a** – readily prepared from the parent benzothiophene in 85% isolated yield using H_2O_2/TFA/CH_2Cl_2 – and 2-bromophenol **3a**, in the presence of TFAA (Supplementary Table 1). During optimization studies it was observed on purging sulfoxide and phenol (both without any solvent) in a vial before the reaction resulted in lower yield than when subjected to reaction without any purging.

Supplementary Table 1. Screening of coupling conditions[a]

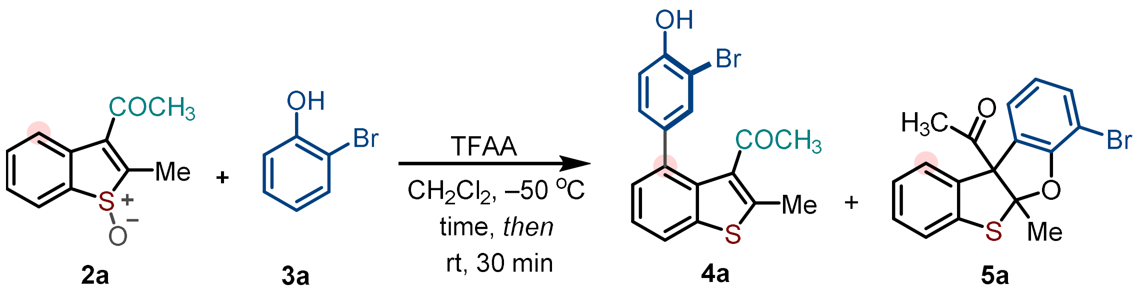

entry	ArOH (equiv)	TFAA (equiv)	Yield of sulfide byproduct (reduced **2a**)	Yield of **4a**[b]
1	1.0	1.0	15	37%
2	1.0	1.5	23	44%
3	1.5	1.5	14	57%
4[c]	1.5	1.5	8	46%
5	2.0	1.5	11	52%
6	2.5	1.5	7	69%
7	3.0	1.5	3	77%
8[d]	3.0	1.5	5	48%

[a] Reaction conditions: A solution of sulfoxide **2a** (1 equiv.) and phenol **3a** in CH_2Cl_2 was added to a solution of TFAA in CH_2Cl_2 at –50 °C. After 45 min, the reaction was warmed to room temperature and stirred for 30 min. [b] Yield determined by ^{1}H NMR using nitromethane as internal standard. [c] Alternative order of addition: TFAA added to a solution of sulfoxide **2a** and phenol **3a** in CH_2Cl_2 at –50 °C. [d] Reaction kept at –50 °C and quenched at –50 °C after 2 h. In all entries, by-product **5a** – the product of C3 arylation – was formed in approx. 20% NMR yield.

Compound characterization data

Benzothiophene substrate synthesis

2,3-Dimethylbenzo[b]thiophene, 1

3-Bromo-2-methylbenzothiophene (500 mg, 2.2 mmol, 1.0 equiv) was dissolved in THF (15 mL).

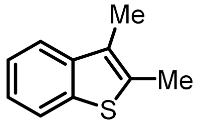

n-BuLi (1.5 mL, 2.4 mmol, 1.1 equiv, 1.6 M in hexane) was added slowly with stirring at −78 °C. The reaction mixture was stirred for 30 min and then methyl iodide (149 μL, 2.4 mmol, 1.2 equiv) was added. After stirring for 1 h, the mixture was warmed to room temperature and stirred for 2 h at room temperature. After completion of the reaction, H_2O was added. The mixture was extracted with CH_2Cl_2 (2 x 10 mL), dried over $MgSO_4$, filtered and the solvents were removed. The residue was purified by column chromatography on silica gel to give the title compound as a colorless oil (321 mg, 2.0 mmol, 90 %). ^{1}H NMR (400 MHz, $CDCl_3$) δ 7.74 (d, *J* = 8.0 Hz, 1H, Ar H), 7.59 (d, *J* = 8.0 Hz, 1H, Ar H), 7.38 – 7.22 (m, 2H, Ar H), 2.49 (s, 3H, CH_3), 2.30 (s, 3H, CH_3). ^{13}C NMR (101 MHz, $CDCl_3$) δ 141.2 (ArC), 138.2 (ArC), 133.8 (ArC), 127.2 (ArC), 123.9 (ArCH), 123.6 (ArCH), 122.1 (ArCH), 121.3 (ArCH), 13.9 (CH_3), 11.5 (CH_3).

The data are in accordance with the literature.[1]

1-(2-Methylbenzo[b]thiophen-3-yl)ethan-1-one, 1a

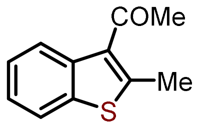

Prepared as described in General Procedure A: 2-methylbenzothiophene (1.26 g, 8.5 mmol) was used as the substrate. Purification by column chromatography on silica gel (gradient from hexane to 3% EtOAc in hexane), afforded the title compound (1.28 g, 6.7 mmol, 80%) as a white solid. ^{1}H NMR (400 MHz, $CDCl_3$) δ 8.19 (d, *J* = 8.2 Hz, 1H, Ar H), 7.75 (d, *J* = 8.0 Hz, 1H, Ar H), 7.44 – 7.39 (m, 1H, Ar H), 7.37 – 7.31 (m, 1H, Ar H), 2.80 (s, 3H, CH_3), 2.67 (s, 3H, CH_3). ^{13}C NMR (101 MHz, $CDCl_3$) δ 198.0 (C=O), 148.9 (ArC), 138.5 (ArC), 137.3 (ArC), 133.3 (ArC), 125.1 (ArCH), 124.6 (ArCH), 124.0 (ArCH), 121.9 (ArCH), 32.0 (CH_3), 17.3 (CH_3).

The data are in accordance with the literature.[2]

3-Formyl 2-methyl benzo[b]thiophene, 1c

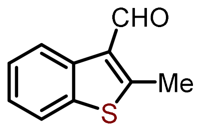

To a stirred solution of 2-methylbenzo[b]thiophene (1.26 g, 8.5 mmol, 1.0 equiv) in CH_2Cl_2 (20 mL) at 0° C, $SnCl_4$ (2 mL, 17 mmol, 2.0 equiv) was added over 5 min under nitrogen. After 15 min, dichloromethyl methyl ether (0.92 mL, 10.2 mmol, 1.2 equiv) was added. The reaction became a yellowish-colored suspension. The reaction was allowed to warm to room temperature and stirred for 16 h, after which it was poured onto ice water (50 mL). The aqueous mixture was acidified with 1.0 N HCl (25 mL) and stirred until the suspension dissolved. The organic phase was separated, dried ($MgSO_4$), and concentrated *in vacuo*. Purification by column chromatography on silica gel (2% EtOAc in hexane) gave the title compound (1.2 g, 6.8 mmol, 81%) as a white solid. ^{1}H NMR (400 MHz, $CDCl_3$) δ 10.37 (s, 1H, CHO), 8.59 (dt, *J* = 8.1, 1.0 Hz, 1H, Ar H), 7.76 (dt, *J* = 7.9, 1.0 Hz, 1H, Ar H), 7.50 – 7.42 (m, 1H, Ar H), 7.37 (ddd, *J* = 8.2, 7.2, 1.3 Hz, 1H, Ar H), 2.92 (s, 3H, CH_3). ^{13}C NMR (101 MHz, $CDCl_3$) δ 184.2 (C=O), 157.4 (ArC), 137.0 (ArC), 136.7 (ArC), 130.0 (ArC), 125.7 (ArCH), 124.9 (ArCH), 123.5 (ArCH), 121.2 (ArCH), 14.3 (CH_3).

The data are in accordance with the literature.[3]

(2-Methylbenzo[b]thiophen-3-yl)(phenyl)methanone, 1d

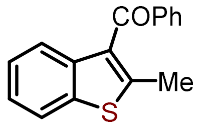

Prepared as described in General Procedure C: 2-methylbenzothiophene (666 mg, 4.5 mmol, 3.0 equiv) was used and benzoyl chloride (211 mg, 1.5 mmol, 1.0 equiv) to afford the title compound as an off-white solid (208 mg, 0.82 mmol, 55% yield). ^{1}H NMR (400 MHz, $CDCl_3$) δ 7.87 – 7.82 (m, 2H, Ar H), 7.79 (dd, *J* = 7.3, 1.7 Hz, 1H, Ar H), 7.64 – 7.57 (m, 1H, Ar H), 7.53 – 7.44 (m, 3H, Ar H), 7.33 – 7.25 (m, 2H, Ar H), 2.50 (s, 3H, CH_3). ^{13}C NMR (101 MHz, $CDCl_3$) δ 193.5 (C=O), 145.3 (ArC), 139.0 (ArC), 138.4 (ArC), 137.7 (ArC), 133.1 (ArCH), 132.3 (ArC), 129.5 (ArCH), 128.5 (ArCH), 124.6 (ArCH), 124.2 (ArCH), 123.2 (ArCH), 121.6, (ArCH), 15.4 (CH_3).

The data are in accordance with the literature.[4]

(4-Methoxyphenyl)(2-methylbenzo[b]thiophen-3-yl)methanone, 1e

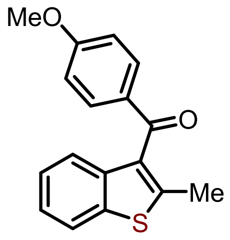

Prepared as described in General Procedure C: 2-methylbenzothiophene (666 mg, 4.5 mmol, 3.0 equiv) was used with 4-methoxy benzoyl chloride (256 mg, 1.5 mmol, 1.0 equiv) to afford the title compound as a colorless liquid (170 mg, 0.6 mmol, 40% yield). ^{1}H NMR (400 MHz, $CDCl_3$) δ 7.83 (d, J = 8.8 Hz, 2H), 7.79 – 7.72 (m, 1H), 7.53 – 7.46 (m, 1H), 7.30 – 7.22 (m, 2H), 6.92 (d, J = 8.9 Hz, 2H), 3.84 (s, 3H), 2.49 (s, 3H). ^{13}C NMR (101 MHz, $CDCl_3$) δ 192.6 (C=O), 164.2 (ArC), 144.5 (ArC), 139.6 (ArC), 138.4 (ArC), 133.0 (ArC), 132.6 (ArCH), 131.8 (ArCH), 124.9 (ArCH), 124.4 (ArCH), 123.5 (ArCH), 122.0 (ArC), 114.3 (ArCH), 55.8 (OCH_3), 15.9 (CH_3).

The data are in accordance with the literature. [5]

2-(4-Bromophenyl)benzo[b]thiophene-3-carbaldehyde, 1n

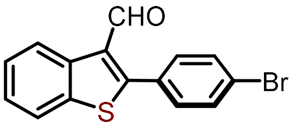

In an oven-dried, round bottom flask, flushed with N_2, benzo[b]thiophene-3-carboxaldehyde (2.03 g, 12.5 mmol, 1.0 equiv), 1-bromo-4-iodobenzene (3.54 g, 12.5 mmol, 1.0 equiv) and potassium carbonate (5.17 g, 37.5 mmol, 3.0 equiv) were dissolved in DMF (25 mL). N_2 was bubbled through the solution with the help of a long needle. PPh_3 (0.328 g, 1.25 mmol, 10 mol%) and $Pd(OAc)_2$ (0.14 g, 0.63 mmol, 5 mol%) were then added. The mixture was heated to 120 °C and stirred overnight. After cooling to room temperature, the reaction mixture was concentrated; the residue was treated with ethyl acetate, and filtered through Celite. The filtrate was washed with water (75 mL), brine (25 mL), dried with $MgSO_4$ and concentrated *in vacuo*. The residue was purified by column chromatography on silica gel (1% ethyl acetate/PE) to afford the title compound (1.58 g, 4.98 mmol, 40%) as a crystalline white solid. ^{1}H NMR (400 MHz, $CDCl_3$) δ 10.04 (s, 1H, CHO), 8.78 (dd, *J* = 8.1, 1.2 Hz, 1H, Ar H), 7.86 (dt, *J* = 7.9, 1.0 Hz, 1H, Ar H), 7.67 (d, *J* = 8.4 Hz, 2H, Ar H), 7.58 – 7.50 (m, 1H, Ar H), 7.50 – 7.44 (m, 3H, Ar H). ^{13}C NMR (101 MHz, $CDCl_3$) δ 186.3 (C=O), 159.0 (ArC), 138.0 (ArC), 137.1 (ArC), 132.3 (ArCH), 132.0 (ArCH), 130.6 (ArC), 130.5 (ArC), 126.6 (ArCH), 126.2 (ArCH), 125.4 (ArCH), 124.9 (ArC), 121.8 (ArCH). ν_{max} (neat)/cm^{-1} 733, 751, 845, 1316, 1347, 1394, 1663, 2836; HRMS (ESI): Calcd. for $C_{15}H_{20}BrOS$ ($M+H^+$), 316.9636; found 316.9634.

Methyl 2-methylbenzo[*b*]thiophene-3-carboxylate, 1s

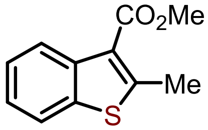

Prepared as described in General Procedure D: 2-methylbenzo[b]thiophene-3-carboxylic acid (85 mg, 0.44 mmol, 1.0 equiv) was used as the substrate and dissolved in *N*,*N*-dimethylformamide (DMF, 1 mL) K_2CO_3 (122 mg, 0.88 mmol, 2.0 equiv) and MeI (55 μL, 0.88 mmol, 2.0 equiv) were then added. The crude mixture was purified by column chromatography (10% EtOAc in hexane) to give the title compound as a colorless oil (89 mg, 0.43 mmol, 98%). ^{1}H NMR (400 MHz, $CDCl_3$) δ 8.41 (d, *J* = 8.0 Hz, 1H, ArH), 7.73 (d, *J* = 8.0 Hz, 1H, ArH), 7.42 (t, *J* = 7.4 Hz, 1H, ArH), 7.32 (t, *J* = 7.4 Hz, 1H, ArH), 3.97 (s, 3H, OCH_3), 2.84 (s, 3H, CH_3). ^{13}C NMR (101 MHz, $CDCl_3$) δ 164.4 ($COOCH_3$), 152.7 (ArC), 138.6 (ArC), 137.1 (ArC), 125.2 (ArCH), 124.5 (ArCH), 124.4 (ArCH), 122.6 (ArC), 121.6 (ArCH), 51.4 ($COOCH_3$), 17.0 (CH_3).

The data are in accordance with the literature.[6]

Dimethyl benzo[b]thiophene-2,3-dicarboxylate, 1u

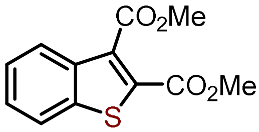

Oxalyl chloride (2.74 mL, 32.0 mmol, 1.6 equiv) was added dropwise to a solution of thiophenol (2.2 g, 20.0 mmol, 1.0 equiv) in anhydrous Et_2O (30 mL) at 0 °C, and the reaction stirred for 1.5 h with warming to room temperature. The reaction mixture was concentrated *in vacuo* and the residue was dissolved in anhydrous CH_2Cl_2 (40 mL). Aluminium chloride (3.20 g, 24.0 mmol, 1.2 equiv) was added portion-wise at 0 °C and the reaction mixture was then heated at reflux for 1 h. The reaction was then cooled to room temperature and poured into ice-cold water. The phases were separated, the organic layer was washed with aqueous $NaHCO_3$ (20 mL) and the aqueous layer was extracted with CH_2Cl_2 (3 x 25 mL). The organic phases were combined, dried with $MgSO_4$, filtered, and concentrated *in vacuo* to give the crude product, which was recrystallized from ethyl acetate to give benzo[b]thiophene-2,3-dione (983 mg, 5.96 mmol, 30%) as a red solid. ^{1}H NMR (400 MHz, $CDCl_3$) δ 7.83 (dd, *J* = 7.6, 1.4 Hz, 1H, ArH), 7.69 (td, *J* = 7.7, 1.5 Hz, 1H, ArH), 7.43 (d, *J* = 7.8 Hz, 1H, ArH), 7.38 (t, *J* = 7.6 Hz, 1H, ArH). ^{13}C NMR (101 MHz, $CDCl_3$) δ 185.9 (C=O), 182.0 (C=O), 142.2 (ArC), 138.5 (ArCH), 128.5 (ArCH), 127.8 (ArCH), 125.9 (ArCH), 124.7 (ArC).

The data are in accordance with the literature.[7]

A mixture of benzothiophene-2,3-dione (645 mg, 3.93 mmol, 1.0 equiv) and chloroacetic acid

(408 mg, 4.32 mmol, 1.1 equiv) and 2N aqueous sodium carbonate solution (7 mL) was heated at 65 °C for 3 h. Then, 8N aqueous sodium hydroxide solution (4 mL) was added to the reaction mixture, and the mixture was heated at 100 °C for 3.5 h. The reaction mixture was acidified with 6N hydrochloric acid and the precipitated solid benzo[b]thiophene-2,3-dicarboxylic acid (523 mg, 2.38 mmol, 60%) was collected by filtration and washed with water. ^{1}H NMR (500 MHz, Acetone-d_6) δ 8.36 – 8.31 (m, 1H, ArH), 8.12 – 8.07 (m, 1H, ArH), 7.63 – 7.56 (m, 2H, ArH). ^{13}C NMR (101 MHz, Acetone-d_6) δ 165.3 (COOH), 162.7 (COOH), 139.9 (ArC), 137.9 (ArC), 131.4 (ArC), 127.4 (ArCH), 125.8 (ArCH), 125.7 (ArCH), 122.5 (ArCH).

Following general procedure D: benzo[b]thiophene-2,3-dicarboxylic acid (222 mg, 0.99 mmol, 1.0 equiv) was dissolved in *N*,*N*-dimethylformamide (DMF, 2 mL) and K_2CO_3 (276 mg, 2.00 mmol, 2.0 equiv) was then added. The mixture was stirred at room temperature for 15 min and MeI (125 μL, 2.01 mmol, 2.0 equiv) was then added. The crude mixture was purified by column chromatography (10% EtOAc in hexane) to give the title compound (238 mg, 0.95 mmol, 95%) as a white solid. ^{1}H NMR (400 MHz, $CDCl_3$) δ 7.94 (dd, *J* = 7.6, 1.8 Hz, 1H, ArH), 7.88 – 7.84 (m, 1H, ArH), 7.54 – 7.44 (m, 2H, ArH), 4.03 (s, 3H, OCH_3), 3.95 (s, 3H, OCH_3). ^{13}C NMR (101 MHz, $CDCl_3$) δ 165.0 (C=O), 162.3 (C=O), 140.5 (ArC), 136.9 (ArC), 133.3 (ArC), 133.3 (ArC), 127.6 (ArCH), 125.8 (ArCH), 124.6 (ArCH), 122.7 (ArCH), 53.1 (OCH_3), 53.0 (OCH_3).

The data are in accordance with the literature.[8]

1-(2,5-Dimethylbenzo[b]thiophen-3-yl)ethan-1-one, 1w

Prepared as described in General Procedure B: 5-methylbenzothiophene (1.33 g, 9.00 mmol) was used as the substrate. 2,5-Dimethylbenzothiophene was obtained and used without purification for acetylation following General Procedure A. 2,5-Dimethylbenzothiophene (1.38 g, 8.51 mmol) was used as the substrate. Purification by column chromatography on silica gel (gradient from 2% EtOAc in hexane to 20% EtOAc in hexane), afforded the title compound (1.48 g, 7.22 mmol, 85%) as a white solid.

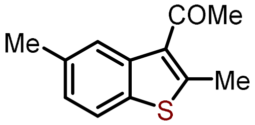

^{1}H NMR (400 MHz, $CDCl_3$) δ ^{1}H NMR (400 MHz, $CDCl_3$) δ 7.99 (s, 1H, ArH), 7.61 (d, *J* = 8.2 Hz, 1H, ArH), 7.16 (d, *J* = 8.2 Hz, 1H, ArH), 2.77 (s, 3H, CH_3), 2.64 (s, 3H, CH_3), 2.48 (s, 3H, CH_3). ^{13}C NMR (101 MHz, $CDCl_3$) δ 196.1 (C=O), 149.1 (ArC), 138.8 (ArC), 135.2 (ArC), 134.5 (ArC), 133.0 (ArC), 126.2 (ArCH), 124.0 (ArCH), 121.4 (ArCH), 32.0 (CH_3), 21.9 (CH_3), 17.2 (CH_3).

The data are in accordance with the literature.[9]

1-(5-Bromo-2-methylbenzo[b]thiophen-3-yl)ethan-1-one, 1x

Prepared as described in General Procedure B: 5-Bromobenzothiophene (1.92 g, 9.00 mmol) was used as the substrate. 5-Bromo-2-methylbenzo[b]thiophene was obtained and used without

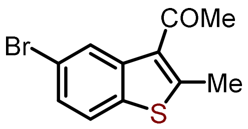

purification for acetylation following General Procedure A. 5-Bromo-2-methylbenzo[b]thiophene (1.93 g, 8.49 mmol) was used as the substrate. Purification by column chromatography on silica gel (gradient from 2% EtOAc in hexane to 20% EtOAc in hexane), afforded the title compound (1.59 g, 5.94 mmol, 70%) as a white solid. ^{1}H NMR (400 MHz, $CDCl_3$) δ 8.42 (d, *J* = 1.9 Hz, 1H, ArH), 7.59 (d, *J* = 8.5 Hz, 1H, ArH), 7.44 (dd, *J* = 8.5, 1.9 Hz, 1H, ArH), 2.81 (s, 3H, CH_3), 2.65 (s, 3H, CH_3). ^{13}C NMR (101 MHz, $CDCl_3$) δ 195.1 (C=O), 150.9 (ArC), 140.1 (ArC), 135.7 (ArC), 132.2 (ArC), 127.6 (ArCH), 126.9 (ArCH), 122.8 (ArCH), 119.7 (ArC), 31.9 (CH_3), 17.4 (CH_3).

The data are in accordance with the literature.[9]

1-(7-Bromo-2-methylbenzo[b]thiophen-3-yl)ethan-1-one, 1z

Prepared as described in General Procedure B: 7-bromobenzothiophene (1.92 g, 9.00 mmol) was used as the substrate. 7-Bromo-2-methylbenzo[b]thiophene was obtained and used without

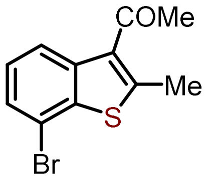

purification for acetylation following General Procedure A. 7-Bromo-2-methylbenzo[b]thiophene (1.93 g, 8.49 mmol) was used as the substrate. Purification by column chromatography on silica gel (gradient from 2% EtOAc in hexane to 20% EtOAc in hexane), afforded the title compound (2.1 g, 7.80 mmol, 90%) as a white solid. ^{1}H NMR (500 MHz, Acetone-d_6) δ 8.29 (dd, *J* = 8.2, 0.9 Hz, 1H, ArH), 7.58 (dd, *J* = 7.7, 0.9 Hz, 1H, ArH), 7.39 (t, *J* = 8.0 Hz, 1H, ArH), 2.86 (s, 3H, CH_3), 2.64 (s, 3H, CH_3). ^{13}C NMR (126 MHz, Acetone-d_6) δ 195.8 (C=O), 150.3 (ArC), 140.6 (ArC), 139.3 (ArC), 134.7 (ArC), 128.1 (ArCH), 127.8 (ArCH), 124.3 (ArCH), 115.1 (ArC), 32.0 (CH_3), 17.0 (CH_3). ν_{max} (neat)/cm^{-1} 723, 788, 1051, 1091, 1148, 1182, 1221, 1309, 1327, 1356, 1386, 1441, 1502, 1536, 1581, 1649, 2971; HRMS (ESI): Calcd. for $C_{11}H_9BrOS$ (M+H$^+$), 268.9636; found 268.9632.

Methyl benzo[*b*]thiophene-3-carboxylate, 1ab

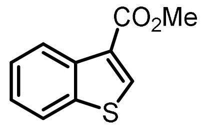

Prepared as described in General Procedure D: Benzo[b]thiophene-3-carboxylic acid (1.78 g, 10.0 mmol, 1.0 equiv) was used as the substrate dissolved in *N*,*N*-dimethylformamide (DMF, 20 mL), K_2CO_3 (2.76 g, 20.0 mmol, 2.0 equiv) and MeI (1.2 mL, 20.0 mmol, 2.0 equiv) were then added. The crude mixture was purified by column chromatography (5% EtOAc in hexane) to give the title compound as a colourless oil (1.90 g, 9.88 mmol, 99%); ^{1}H NMR (400 MHz, $CDCl_3$) δ = 8.60 (d, *J* = 8.0 Hz, 1H, ArH), 8.38 (s, 1H, ArH), 7.87 (d, *J* = 8.0 Hz, 1H, ArH) 7.49 (t, *J* = 7.6 Hz, 1H, ArH), 7.41 (t, *J* = 7.6 Hz, 1H, ArH), 3.96 (s, 3H, OCH_3). ^{13}C NMR (101 MHz, $CDCl_3$) δ 163.4 (C=O), 140.1 (ArC), 136.8 (ArC), 136.7 (ArCH), 127.2 (ArC), 125.5 (ArCH), 125.1 (ArCH), 124.8 (ArCH), 122.6 (ArCH), 51.9 (OCH_3).
The data are in accordance with the literature.[8]

Methyl 5-bromobenzo[b]thiophene-3-carboxylate, 1ah

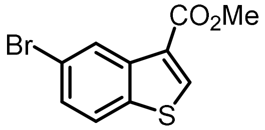

Following General Procedure D: 5-bromobenzo[b]thiophene-3-carboxylic acid (257 mg, 0.99 mmol) was dissolved in *N*,*N*-dimethylformamide (DMF, 2 mL) and K_2CO_3 (276 mg, 2.0 mmol, 2.0 equiv) was then added. The mixture was stirred at room temperature for 15 min and MeI (125 μL, 2.0 mmol, 2.0 equiv) was added. The crude mixture was purified by column chromatography (10% EtOAc in hexane) to give the title compound as an off white solid (266 mg, 0.98 mmol, 98%); ^{1}H NMR (400 MHz, $CDCl_3$) δ 8.75 (d, *J* = 1.9 Hz, 1H, ArH), 8.38 (s, 1H, ArH), 7.71 (dd, *J* = 8.6, 1.3 Hz, 1H, ArH), 7.50 (dd, *J* = 8.5, 1.9 Hz, 1H, ArH), 3.96 (s, 3H, OCH_3) ppm; ^{13}C NMR (100 MHz, $CDCl_3$) δ 162.9 (C=O), 138.7 (ArC), 138.4 (ArC), 138.1 (ArCH), 128.4 (ArCH), 127.7 (ArCH), 126.6 (ArC), 123.8 (ArCH), 120.1 (ArC), 52.0 (CH_3); HRMS (ESI): Calcd. for $C_{10}H_7O_2BrS$ ($M+H^+$), 270.9428; found 270.9424.

Methyl 5-phenylbenzo[b]thiophene-3-carboxylate, 1ai

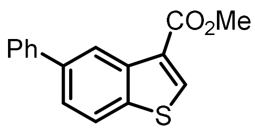

To an oven-dried vial was added 5-bromobenzo[b]thiophene-3-carboxylate (269 mg, 1.0 mmol, 1.0 equiv), palladium tetrakis(triphenylphosphine (92 mg, 0.08 mmol, 8 mol%), and Na_2CO_3 (466 mg, 4.4 mmol, 4.4 equiv) followed by toluene (3.0 mL) and H_2O (3.0 mL). A solution of phenylboronic acid (1.1 equiv) in EtOH (4 mL) was then added to the above solution. The reaction mixture was refluxed for 6 hours and then concentrated *in vacuo*. The biphasic mixture was acidified with an aqueous solution of HCl 1N until pH = 2. The aqueous layer was extracted with EtOAc (5 mL x 3) and the organic layer was washed with H_2O, and brine, dried over $MgSO_4$, filtered, and evaporated to dryness. The residue was purified by flash chromatography on silica gel (n-hexane/ EtOAc 9/1) to afford the title compound (227 mg, 0.85 mmol, 85%) as a colourless oil. ^{1}H NMR (400 MHz, $CDCl_3$) δ 8.84 (dd, *J* = 1.9, 0.7 Hz, 1H, ArH), 8.42 (s, 1H, ArH), 7.93 (dd, *J* = 8.4, 0.7 Hz, 1H, ArH), 7.73 – 7.70 (m, 2H, ArH), 7.67 (dd, *J* = 8.4, 1.8 Hz, 1H, ArH), 7.51 – 7.45 (m, 2H, ArH), 7.40 – 7.36 (m, 1H, ArH), 3.97 (s, 3H, OCH_3). ^{13}C NMR (101 MHz, $CDCl_3$) δ 163.4 (C=O), 141.3 (ArC), 139.2 (ArC), 139.1 (ArC), 137.5 (ArC), 137.4 (ArCH), 129.0 (ArCH), 127.7 (ArCH), 127.5 (ArCH), 127.3 (ArC), 124.8 (ArCH), 123.3 (ArCH), 122.8 (ArCH), 51.9 (OCH_3); HRMS (APCI): Calcd. for $C_{16}H_{12}O_2S$ (M+H$^+$), 269.0631; found 269.0636.

Benzothiophene *S*-oxides synthesis:

2,3-Dimethylbenzo[b]thiophene 1-oxide, 2

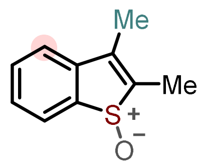

Prepared as described in General Procedure E, using 2,3-dimethylbenzo[b]thiophene (162 mg, 0.99 mmol). Purification by column chromatography on silica gel (gradient from 5% EtOAc in hexane to 50% EtOAc in hexane), afforded the title compound as a white solid (157 mg, 0.88 mmol, 88%); ^{1}H NMR (400 MHz, $CDCl_3$) δ 7.86 (d, *J* = 7.5 Hz, 1H, Ar H), 7.50 (t, *J* = 7.6 Hz, 1H, Ar H), 7.39 (t, *J* = 7.5 Hz, 1H, Ar H), 7.34 (d, *J* = 7.6 Hz, 1H, Ar H), 2.31 (s, 3H, CH_3), 2.14 (s, 3H, CH_3). ^{13}C NMR (101 MHz, $CDCl_3$) δ 143.7 (Ar C), 143.0 (Ar C), 140.1 (Ar C), 136.7 (Ar C), 132.1 (Ar CH), 127.8 (Ar CH), 126.0 (Ar CH), 121.8 (Ar CH), 11.7 (CH_3), 10.7 (CH_3). ν_{max} (neat)/cm^{-1} 764, 1003, 1049, 1140, 1200, 1271, 1296, 1461, 1576, 1627, 1681, 1719, 3005; HRMS (ESI): Calcd. for $C_{11}H_{10}O_2SNa$ (M+Na$^+$), 229.0299; found 229.0295.

1-(2-Methyl-1-oxidobenzo[b]thiophen-3-yl)ethan-1-one, 2a

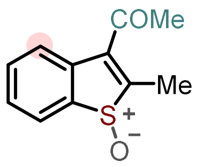

Prepared as described in General Procedure E, using 1-(2-methylbenzo[b]thiophen-3-yl)ethan-1-one (190 mg, 0.99 mmol). Purification by column chromatography on silica gel (gradient from 5% EtOAc in hexane to 50% EtOAc in hexane), afforded the title compound as a white solid (175 mg, 0.85 mmol, 85%); ^{1}H NMR (400 MHz, $CDCl_3$) δ 7.87 (d, *J* = 7.6 Hz, 1H, Ar H), 7.62 (d, *J* = 7.8 Hz, 1H, Ar H), 7.50 (t, *J* = 7.6 Hz, 1H, Ar H), 7.42 (t, *J* = 7.5 Hz, 1H, Ar H), 2.55 (s, 3H, CH_3), 2.51 (s, 3H, CH_3). ^{13}C NMR (101 MHz, $CDCl_3$) δ 197.5 (C=O), 151.8 (Ar C), 143.1 (Ar C), 137.7 (Ar C), 135.9 (Ar C), 132.4 (Ar CH), 128.3 (Ar CH), 126.5 (Ar CH), 124.3 (Ar CH), 31.4 (CH_3), 12.7 (CH_3). ν_{max} (neat)/cm^{-1} 710, 733, 1018, 1185, 1295, 1328, 1360, 1453, 1509, 1665, 2917, 3062; HRMS (ESI): Calcd. for $C_{11}H_{10}O_2SNa$ (M+Na$^+$), 229.0299; found 229.0295.

(2-Methyl-1-oxidobenzo[b]thiophen-3-yl)(phenyl)methanone, 2b

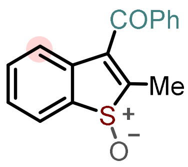

Prepared as described in General Procedure E, using (2-methylbenzo[b]thiophen-3-yl)(phenyl)methanone (252 mg, 0.99 mmol). Purification by column chromatography on silica gel (gradient from 1% EtOAc in hexane to 30% EtOAc in hexane), afforded the title compound as a white solid

(242 mg, 0.90 mmol, 90%); ^{1}H NMR (400 MHz, $CDCl_3$) δ 7.97-7.95 (m, 3H, ArH), 7.67 (t, *J* = 7.4 Hz, 1H, ArH), 7.52 (t, *J* = 7.3 Hz, 2H, ArH), 7.48 – 7.41 (m, 2H, ArH), 7.22-7.19 (m, 1H, ArH), 2.27 (s, 3H, CH_3). ^{13}C NMR (101 MHz, $CDCl_3$) δ 192.3 (C=O), 150.0 (Ar C), 143.7 (Ar C), 137.8 (Ar C), 137.2 (Ar C), 136.1 (Ar C), 134.9 (Ar CH), 132.5 (Ar CH), 129.8 (Ar CH), 129.3 (Ar CH), 128.5 (Ar CH), 126.7 (Ar CH), 123.8 (Ar CH), 12.4 (CH_3). ν_{max} (neat)/cm^{-1} 731, 1062, 1264, 1723, 2854, 2926; HRMS (ESI): Calcd. for $C_{16}H_{12}O_2SNa$ (M+Na$^+$), 291.0456; found 291.0453.

4-Methoxyphenyl)(2-methyl-1-oxidobenzo[b]thiophen-3-yl)methanone, 2c

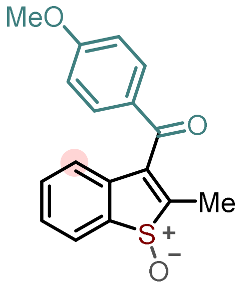

Prepared as described in General Procedure E, using (4-methoxyphenyl)(2-methylbenzo[b]thiophen-3-yl)methanone (282 mg, 0.99 mmol). Purification by column chromatography on silica gel (gradient from 5% EtOAc in hexane to 30% EtOAc in hexane), afforded the title compound as a white solid (238 mg, 0.80 mmol, 80%); ^{1}H NMR (400 MHz, $CDCl_3$) δ 7.93-7.91 (m, 3H, ArH), 7.44 – 7.38 (m, 2H, ArH), 7.20 – 7.15 (m, 1H, ArH), 6.95 (d, *J* = 8.9 Hz, 2H, ArH), 3.87 (s, 3H, OCH_3), 2.26 (s, 3H, CH_3). ^{13}C NMR (101 MHz, $CDCl_3$) δ 190.4 (C=O), 165.0 (ArC), 148.7 (ArC), 143.6 (ArC), 138.2 (ArC), 137.3 (ArC), 132.3 (ArCH), 132.2 (ArCH), 128.9 (ArC), 128.3 (ArCH), 126.5 (ArCH), 123.7 (ArCH), 114.5 (ArCH), 55.7 (OCH_3), 12.2 (CH_3). ν_{max} (neat)/cm^{-1} 763, 876, 1021, 1150, 1229, 1259, 1594, 2840; HRMS (ESI): Calcd. for $C_{17}H_{14}O_3S$ (M+H$^+$), 299.0742; found 299.0736.

2-Methylbenzo[b]thiophene-3-carbaldehyde 1-oxide, 2d

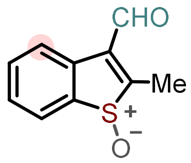

Prepared as described in General Procedure E, using 2-methylbenzo[b]thiophene-3-carbaldehyde (176 mg, 0.99 mmol). Quenching with aqueous $NaHCO_3$ solution carried out at 0 °C to avoid formation of side products. Purification by column chromatography on silica gel (gradient from 1% EtOAc in hexane to 30% EtOAc in hexane), afforded the title compound as a white solid (48 mg, 0.25 mmol, 25%); ^{1}H NMR (400 MHz, $CDCl_3$) δ 10.32 (s, 1H, CHO), 8.30 (d, *J* = 7.9 Hz, 1H, ArH), 7.92 (d, *J* = 7.4 Hz, 1H, ArH), 7.57 (td, *J* = 7.7, 1.3 Hz, 1H, ArH), 7.49 (td, *J* = 7.6, 1.2 Hz, 1H, ArH), 2.76 (s, 3H, CH_3). ^{13}C NMR (101 MHz, $CDCl_3$) δ 186.9 (C=O), 163.9 (ArC), 142.7 (ArC), 134.9 (ArC), 132.7 (ArCH), 132.6 (ArC), 128.8 (ArCH), 126.5 (ArCH), 125.4 (ArCH), 11.3 (CH_3). ν_{max} (neat)/cm^{-1}

753, 875, 950, 987, 1022, 1058, 1122, 1155, 1216, 1264, 1295, 1339, 1374, 1442, 1458, 1584, 1675, 1711, 2851, 2919; HRMS (ESI): Calcd. for $C_{10}H_8O_2SNa$ (M+Na$^+$), 215.0143; found 215.0143.

2-(4-Bromophenyl)benzo[b]thiophene-3-carbaldehyde 1-oxide, 2n

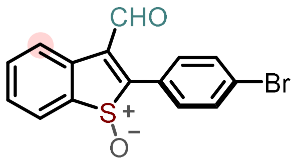

Prepared as described in General Procedure E, using 2-(4-bromophenyl)benzo[b]thiophene-3-carbaldehyde (317 mg, 0.99 mmol). Purification by column chromatography on silica gel (gradient from 1% EtOAc in hexane to 30% EtOAc in hexane), afforded the title compound as a yellow solid (100 mg, 0.30 mmol, 30%); ^{1}H NMR (400 MHz, $CDCl_3$) δ 10.02 (s, 1H, CHO), 8.41 (d, *J* = 8.4 Hz, 1H, ArH), 8.00 (d, *J* = 7.6 Hz, 1H, ArH), 7.73 (d, *J* = 8.6 Hz, 2H, ArH), 7.65 (t, *J* = 7.6 Hz, 1H, ArH), 7.59-7.54 (m, 3H, ArH). ^{13}C NMR (101 MHz, $CDCl_3$) δ 188.4 (C=O), 164.1 (ArC), 143.3 (ArC), 134.8 (ArC), 132.9 (ArCH), 132.4 (ArC), 132.2 (ArCH), 129.6 (ArCH), 127.1 (ArC), 126.7 (ArCH), 126.53 (ArCH), 126.50 (ArC). ν_{max} (neat)/cm^{-1} 755, 892, 1033, 1667, 2849, 2920; HRMS (ESI): Calcd. for $C_{23}H_{19}BrO_3SNa$ (M+Na$^+$) CH_3OH, 386.9666; found 386.9668.

Methyl 2-methylbenzo[*b*]thiophene-3-carboxylate 1-oxide, 2s

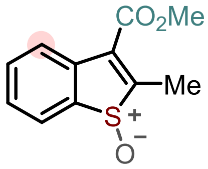

Prepared as described in General Procedure E, using methyl 2-methylbenzo[b]thiophene-3-carboxylate (51 mg, 0.25 mmol). Purification by column chromatography on silica gel (gradient from 1% EtOAc in hexane to 30% EtOAc in hexane), afforded the title compound as a white solid (50 mg, 0.22 mmol, 90%); ^{1}H NMR (400 MHz, $CDCl_3$) δ 8.02 (d, *J* = 7.7 Hz, 1H, ArH), 7.89 (d, *J* = 7.6 Hz, 1H, ArH), 7.55 (t, *J* = 7.7 Hz, 1H, ArH), 7.45 (t, *J* = 7.6 Hz, 1H, ArH), 3.98 (s, 3H, OCH_3), 2.66 (s, 3H, CH_3). ^{13}C NMR (101 MHz, $CDCl_3$) δ 163.7 (C=O), 158.7 (ArC), 142.9 (Ar C), 135.9 (ArC), 132.5 (ArCH), 128.3 (ArCH), 128.9 (ArC), 126.4 (ArCH), 125.3 (ArCH), 52.5 (OCH_3), 13.4 (CH_3). ν_{max} (neat)/cm^{-1} 758, 1022, 1210, 1437, 1706, 2953, 3100; HRMS (ESI): Calcd. for $C_{11}H_{10}O_3S$ Na (M+Na$^+$), 245.0248; found 245.0245.

Dimethyl benzo[b]thiophene-2,3-dicarboxylate 1-oxide, 2u

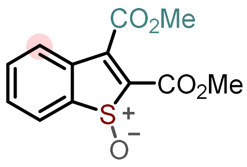

Prepared as described in General Procedure E, using dimethyl benzo[b]thiophene-2,3-dicarboxylate (250 mg, 0.99 mmol). Purification by column chromatography on silica gel (gradient from 5% EtOAc in hexane to 30% EtOAc in hexane), afforded the title compound as a light pink solid (231 mg, 0.87 mmol, 87%); ^{1}H NMR (400 MHz, $CDCl_3$) δ 8.02 – 7.95 (m, 1H, ArH), 7.71 – 7.56 (m, 3H, ArH), 4.03 (s, 3H, OCH_3), 3.98 (s, 3H, OCH_3). ^{13}C NMR (101 MHz, $CDCl_3$) δ 163.2 (C=O), 160.7 (C=O), 145.6 (ArC), 143.7 (ArC), 141.1 (ArC), 133.8 (ArC), 132.9 (ArCH), 132.1 (ArCH), 127.2 (ArCH), 126.0 (ArCH), 53.6 (OCH_3). ν_{max} (neat)/cm^{-1} 752, 1059, 1158, 1216, 1243, 1273, 1313, 1342, 1430, 1570, 1594, 1717, 1745, 2922, 2951; HRMS (ESI): Calcd. for $C_{12}H_{10}O_5SNa$ ($M+Na^+$), 289.0147; found 289.0142.

1-(2,5-Dimethyl-1-oxidobenzo[b]thiophen-3-yl)ethan-1-one, 2w

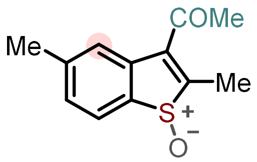

Prepared as described in General Procedure E, using 1-(2,5-Dimethylbenzo[b]thiophen-3-yl)ethan-1-one (204 mg, 0.99 mmol). Purification by column chromatography on silica gel (gradient from 5% EtOAc in hexane to 30% EtOAc in hexane), afforded the title compound as a white solid (202 mg, 0.92 mmol, 92%); ^{1}H NMR (400 MHz, $CDCl_3$) δ 7.77 (d, *J* = 7.8 Hz, 1H, ArH), 7.42 (s, 1H, ArH), 7.25 (overlapped with $CDCl_3$, d, *J* = 8.4 Hz, 1H, ArH), 2.57 (s, 3H, CH_3), 2.52 (s, 3H, CH_3), 2.42 (s, 3H, CH_3). ^{13}C NMR (101 MHz, $CDCl_3$) δ 197.9 (C=O), 151.8 (ArC), 143.3 (ArC), 140.3 (ArC), 138.0 (ArC), 136.4 (ArC), 129.1 (ArCH), 126.5 (ArCH), 124.9 (ArCH), 31.5 (CH_3), 21.9 (CH_3), 12.9 (CH_3). ν_{max} (neat)/cm^{-1} 804, 1028, 1061, 1124, 1154, 1181, 1215, 1298, 1317, 1353, 1372, 1411, 1445, 1587, 1675, 2919, 2972; HRMS (ESI): Calcd. for $C_{12}H_{12}O_2SNa$ ($M+Na^+$), 243.0456; found 243.0456.

1-(5-Bromo-2-methyl-1-oxidobenzo[b]thiophen-3-yl)ethan-1-one, 2x

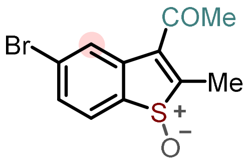

Prepared as described in General Procedure E, using 1-(5-bromo-2-methylbenzo[b]thiophen-3-yl)ethan-1-one (269 mg, 0.99 mmol). Purification by column chromatography on silica gel (gradient from 5% EtOAc in hexane to 30% EtOAc in hexane), afforded the title compound as

a white solid (254 mg, 0.89 mmol, 89%); ^{1}H NMR (400 MHz, $CDCl_3$) *δ* 7.87 (d, *J* = 1.7 Hz, 1H, ArH), 7.73 (d, *J* = 8.2 Hz, 1H, ArH), 7.57 (dd, *J* = 8.0, 1.7 Hz, 1H, ArH), 2.57 (two singlets overlapped, 6H, CH_3). ^{13}C NMR (101 MHz, $CDCl_3$) δ 196.8 (C=O), 154.4 (ArC), 141.7 (ArC), 137.8 (ArC), 136.4 (ArC), 131.3 (ArCH), 127.9 (ArCH), 127.6 (ArCH), 127.3 (ArC), 31.6 (CH_3), 13.2 (CH_3). ν_{max} (neat)/cm^{-1} 755, 823, 898, 1022, 1288, 1307, 1355, 1390, 1437, 1549, 1572, 16712998, 3063; HRMS (ESI): Calcd. for $C_{11}H_9BrO_2SNa$ (M+Na$^+$), 306.9404; found 306.9406.

1-(7-Bromo-2-methyl-1-oxidobenzo[b]thiophen-3-yl)ethan-1-one, 2z

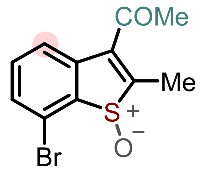

Prepared as described in General Procedure E, using 1-(7-bromo-2-methylbenzo[b]thiophen-3-yl)ethan-1-one (269 mg, 0.99 mmol). Purification by column chromatography on silica gel (gradient from 5% EtOAc in hexane to 30% EtOAc in hexane), afforded the title compound as a white solid (274 mg, 0.96 mmol, 96%); ^{1}H NMR (400 MHz, $CDCl_3$) δ 7.62 (d, *J* = 7.8 Hz, 1H, ArH), 7.53 (d, *J* = 8.0 Hz, 1H, ArH), 7.39 (t, *J* = 7.8 Hz, 1H, ArH), 2.58 (s, 3H, CH_3), 2.58 (s, 3H, CH_3). ^{13}C NMR (101 MHz, $CDCl_3$) δ 197.2 (C=O), 152.6 (ArC), 142.5 (ArC), 138.6 (ArC), 137.2 (ArC), 134.1 (ArCH), 132.1 (ArCH), 123.4 (ArCH), 122.3 (ArC), 31.6 (CH_3), 13.2 (CH_3). ν_{max} (neat)/cm^{-1} 721, 780, 1021, 1044, 1405, 1438, 1667, 2917; HRMS (ESI): Calcd. for $C_1H_9BrO_2SNa$ (M+Na$^+$), 306.9404; found 306.9399.

1-(3-Methyl-1-oxidobenzo[b]thiophen-2-yl)ethan-1-one, 2aa

Prepared as described in General Procedure E, using 1-(3-methylbenzo[b]thiophen-2-yl)ethan-1-one (190 mg, 0.99 mmol). Purification by column chromatography on silica gel (gradient from 5% EtOAc in hexane to 30% EtOAc in hexane), afforded the title compound as a white solid (169 mg, 0.82 mmol, 82%); ^{1}H NMR (400 MHz, $CDCl_3$) δ 7.99 – 7.94 (m, 1H, ArH), 7.68 – 7.63 (m, 3H, ArH), 2.71 (s, 3H, CH_3), 2.65 (s, 3H, CH_3). ^{13}C NMR (101 MHz, $CDCl_3$) δ 193.0 (C=O), 152.8 (ArC), 144.6 (ArC), 144.31 (ArC), 138.3 (ArC), 132.7 (ArCH), 131.9 (ArCH), 126.6 (ArCH), 125.1 (ArCH), 31.6 (CH_3), 14.1 (CH_3). ν_{max} (neat)/cm^{-1} 736, 1017, 1130, 1222, 1270, 1363, 1544, 1670, 3007, 3060; HRMS (ESI): Calcd. for $C_{11}H_{10}O_2SNa$ (M+Na$^+$), 229.0299; found 229.0294.

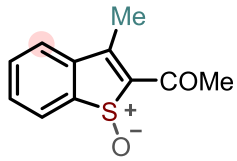

Methyl benzo[*b*]thiophene-3-carboxylate 1-oxide, 2ab

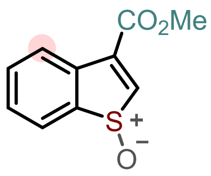

Prepared as described in General Procedure F, using methyl benzo[*b*]thiophene-3-carboxylate (192 mg, 0.99 mmol, 1.0 equiv), $BF_3·OEt_2$ (1.0 mL, 8.10 mmol, 8.1 equiv), *m*-CPBA (269 mg, 1.20 mmol, 1.2 equiv) and CH_2Cl_2 (4 mL). The crude mixture was purified by recrystallization using CH_2Cl_2 and hexane as solvents, to give the title compound as a white solid (162 mg, 0.78 mmol, 78%); ^1H NMR (500 MHz, $CDCl_3$) δ 8.26 (d, *J* = 7.8 Hz, 1H, ArH), 7.94 (d, *J* = 7.6 Hz, 1H, ArH), 7.76 (s, 1H, ArH), 7.61 (t, *J* = 7.6 Hz, 1H, ArH), 7.54 (t, *J* = 7.6 Hz, 1H, ArH), 3.96 (s, 3H, OCH_3). ^{13}C NMR (101 MHz, $CDCl_3$) δ 162.6 (C=O), 145.8 (ArC), 143.8 (ArCH), 135.9 (ArC), 134.2 (ArC), 132.4 (ArCH), 129.5 (ArCH), 126.5 (ArCH), 126.4 (ArCH), 52.93 (OCH_3). ν_{max} (neat)/cm^{-1} 770, 807, 940, 1018, 1048, 1137, 1206, 1400, 3058, 3089; HRMS (APCI): Calcd. for $C_{10}H_9O_3S$ ($M+H^+$), 209.0267; found 209.0264.

Methyl 5-bromobenzo[b]thiophene-3-carboxylate 1-oxide, 2ah

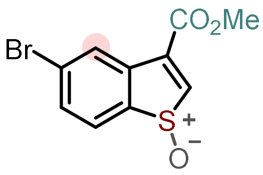

Prepared as described in General Procedure F, using methyl 5-bromobenzo[b]thiophene-3-carboxylate (136 mg, 0.50 mmol, 1.0 equiv), $BF_3·OEt_2$ (0.5 mL, 4.05 mmol, 8.0 equiv), *m*-CPBA (134 mg, 0.60 mmol, 1.2 equiv) and CH_2Cl_2 (4 mL). The crude mixture was purified by recrystallization using CH_2Cl_2 and hexane as solvents, to give the title compound (72 mg, 0.25 mmol, 50%) as a white solid; ^1H NMR (400 MHz, $CDCl_3$) δ 8.25 (dd, J = 7.9, 1.1 Hz, 1H, ArH), 7.94 (dd, J = 7.5, 1.2 Hz, 1H, Ar H), 7.62 – 7.58 (m, 1H, Ar H), 7.55 – 7.52 (m, 1H, Ar H), 3.96 (s, 3H, OCH_3). ^{13}C NMR (101 MHz, $CDCl_3$) δ 162.5 (C=O), 145.7 (ArC), 143.7 (ArC), 135.9 (ArC), 134.2 (ArC), 132.5 (ArCH), 129.5 (ArCH), 126.5 (ArCH), 126.4 (ArCH), 52.9 (OCH_3). ν_{max} (neat)/cm^{-1} 718, 744, 773, 1015, 1044, 1123, 1207, 1424, 1438, 1455, 1720, 2957, 3056, 3087; HRMS (APCI): Calcd. for $C_{10}H_7BrO_3S$ ($M+H^+$), 286.9378; found 286.9372.

Methyl 5-phenylbenzo[b]thiophene-3-carboxylate 1-oxide, 2ai

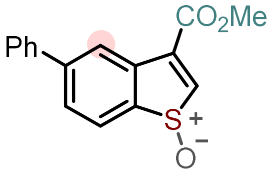

Prepared as described in General Procedure F, using methyl 5-phenylbenzo[b]thiophene-3-carboxylate (134 mg, 0.50 mmol, 1.0 equiv), $BF_3·OEt_2$ (0.5 mL, 4.05 mmol, 8.0 equiv), *m*-CPBA (134 mg, 0.60 mmol,

1.2 equiv) and CH_2Cl_2 (4 mL). The crude mixture was purified by recrystallization using CH_2Cl_2 and hexane as solvents, to give the title compound (113 mg, 0.80 mmol, 80%) as a white solid; ^{1}H NMR (400 MHz, $CDCl_3$) δ 8.49 (d, *J* = 1.6 Hz, 1H, ArH), 8.00 (d, *J* = 8.0 Hz, 1H, ArH), 7.81 (s, 1H, ArH), 7.75 (dd, *J* = 8.0, 1.7 Hz, 1H, ArH), 7.50-7.43 (m, 3H, ArH), 3.98 (s, 3H, OCH_3) ppm; ν_{max} (neat)/cm^{-1} 767, 932, 1024, 1090, 1169, 1216, 1455, 3078, 3060; HRMS (ESI): Calcd. for $C_{16}H_{13}OS$ (M+H$^+$), 285.0585; found 285.0579.

4,6-Dimethyldibenzo[*b*,*d*]thiophene 5-oxide, 2ak

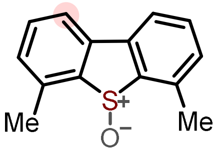

Prepared as described in General Procedure E, using 4,6-dimethyldibenzo[b,d]thiophene (212 mg, 0.99 mmol). Purification by column chromatography on silica gel [gradient from 1% EtOAc in hexane to 30% EtOAc in hexane], afforded the title compound as a light yellow solid (210 mg, 0.92 mmol, 92%); ^{1}H NMR (400 MHz, $CDCl_3$) δ 7.58 (d, *J* = 7.5 Hz, 2H, ArH), 7.44 (t, *J* = 7.6 Hz, 2H, ArH), 7.21 (d, *J* = 7.5 Hz, 2H, ArH), 2.73 (s, 6H, 2 x CH_3). ^{13}C NMR (101 MHz, $CDCl_3$) δ 142.8 (ArC), 139.2 (ArC), 137.7 (ArC), 132.8 (ArCH), 131.0 (ArCH), 119.6 (ArCH), 18.8 (CH_3). ν_{max} (neat)/cm^{-1} 778, 1002, 1282, 1379, 1442, 1473, 1685, 2943, 3013; HRMS (ESI): Calcd. for $C_{14}H_{12}OS$ (M+Na$^+$), 251.0507; found 251.0501.

4-Methyldibenzo[b,d]thiophene 5-oxide, 2ao

To a solution of dibenzo[b,d]thiophene (368 mg, 2.00 mmol) in THF (8 mL) at –78 °C under N_2 was

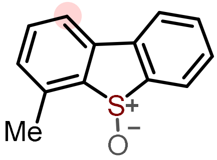

added *n*-BuLi (1.6 M in hexanes, 2.5 mL, 4.00 mmol), and the reaction mixture was warmed to room temperature and stirred for 5 h. The resulting deep orange solution was cooled to –78 °C and MeI (250 µL, 4.00 mmol) was added. The reaction mixture was warmed to room temperature, stirred for 30 min, and quenched with saturated aqueous NH_4Cl (10 mL). The product was extracted with CH_2Cl_2 (3 x 10 mL), the combined organic extracts were dried over $MgSO_4$, and the solvent was removed *in vacuo* to afford 4-methyldibenzo[b,d]thiophene (435 mg, 2.19 mmol, 99%). ^{1}H NMR (400 MHz, $CDCl_3$) δ 8.17-8.15 (m, 1H, ArH), 8.02 (d, J = 7.6 Hz, 1H, ArH), 7.90-7.88 (m, 1H, ArH), 7.49-7.45 (m, 2 H, ArH), 7.41 (t, J = 7.6 Hz, 1H, ArH), 7.28 (d, J = 7.2 Hz, 1H, ArH), 2.60 (s, 3H, CH_3).^{13}C NMR (101 MHz, $CDCl_3$) δ 139.7 (ArC), 139.4 (ArC), 136.4 (ArC), 135.5 (ArC), 132.4 (ArC), 127.1 (ArCH), 126.7 (ArCH), 124.9 (ArCH), 124.5 (ArCH), 123.0 (ArCH), 121.9 (ArCH), 119.2 (ArCH), 20.7 (CH_3).

The product was used without further purification for the next step. Prepared as described in General Procedure E, using 4-methyldibenzo[b,d]thiophene (198 mg, 0.99 mmol). Purification by column chromatography on silica gel (gradient from 1% EtOAc in hexane to 30% EtOAc in hexane), afforded the title compound as a white solid (188 mg, 0.88 mmol, 88%); ^{1}H NMR (400 MHz, $CDCl_3$) δ 8.00 – 7.95 (m, 1H, ArH), 7.77 (dd, J = 7.6, 1.4 Hz, 1H, ArH), 7.65 – 7.55 (m, 2H, ArH), 7.51 – 7.44 (m, 2H, ArH), 7.23 (d, J = 7.5 Hz, 1H, ArH), 2.75 (s, 3H, Me) ppm; ^{13}C NMR (101 MHz, $CDCl_3$) δ 144.9 (ArC), 143.3 (ArC), 139.5 (ArC), 137.5 (ArC), 137.1 (ArC), 132.9 (ArCH), 132.5 (ArCH), 131.2 (ArCH), 129.5 (ArCH), 127.5 (ArCH), 122.1 (ArCH), 119.5 (ArCH), 18.8 (CH_3). ν_{max} (neat)/cm^{-1} 759, 1007, 1019, 1050, 1444, 1481, 1575, 1590, 1707, 1817, 2851, 2919; HRMS (ESI): Calcd. for $C_{13}H_{10}OSNa$ (M+Na$^+$), 237.0350; found 237.0348.

2-Phenyldibenzo[b,d]thiophene 5-oxide, 2ap

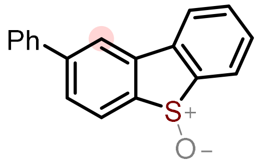

To an oven-dried vial was added 2-bromodibenzo[b,d]thiophene (658 mg, 2.5 mmol, 1 equiv) palladium tetrakis(triphenylphosphine (230 mg, 0.2 mmol, 8 mol%), and Na_2CO_3 (1.52 g, 11 mmol, 4.4 equiv) followed by toluene (7.5 mL) and H_2O (7.5 mL). A solution of phenylboronic acid (1.1 equiv) in EtOH (10 mL) was then added to the above solution. The reaction mixture was refluxed for 6 hours and then concentrated *in vacuo*. The biphasic mixture was acidified with an aqueous solution of HCl 1N until pH = 2. The aqueous layer was extracted with EtOAc (10 mL x 3) and the organic layer was washed with H_2O, and brine, dried over $MgSO_4$, filtered, and evaporated to dryness. The residue was purified by column chromatography on silica gel (n-hexane/ EtOAc 9/1) to afford the title compound (586 mg, 2.25 mmol, 90%) as a crystalline white solid. ^{1}H NMR (400 MHz, $CDCl_3$) δ 8.34 (d, *J* = 1.5 Hz, 1 H, ArH), 8.20-8.23 (m, 1 H, ArH), 7.88 (d, *J* = 8.0 Hz, 1 H, ArH), 7.85-7.87 (m, 1 H, ArH), 7.68-7.70 (m, 3 H, ArH), 7.44-7.50 (m, 4 H, ArH), 7.38 (t, *J* = 7.5 Hz, 1 H, ArH). ^{13}C NMR (101 MHz, $CDCl_3$) δ 141.6 (ArC), 140.3 (ArC), 138.9 (ArC), 138.3 (ArC), 136.8 (ArC), 135.8 (ArC), 129.4 (ArCH), 127.8 (ArCH), 127.6 (ArCH), 127.2 (ArCH), 126.6 (ArCH), 124.9 (ArCH), 123.4 (ArCH), 123.2 (ArCH), 122.0 (ArCH), 120.2 (ArCH).

Prepared as described in General Procedure E, using 2-phenyldibenzo[b,d]thiophene (260 mg, 0.99 mmol). Purification by column chromatography on silica gel (gradient from 1% EtOAc in hexane to 30% EtOAc in hexane), afforded the title compound as a white solid (240 mg, 0.87

mmol, 87%); ^{1}H NMR (400 MHz, $CDCl_3$) δ 7.96 (dd, *J* = 1.6, 0.6 Hz, 1H, ArH), 7.89 – 7.85 (m, 2H, ArH), 7.72 (dd, *J* = 8.0, 1.6 Hz, 1H, ArH), 7.69 – 7.62 (m, 3H, ArH), 7.57 – 7.44 (m, 4H, ArH). ^{13}C NMR (101 MHz, $CDCl_3$) δ 147.5 (ArC), 139.5 (ArC), 138.4 (ArC), 136.4 (ArC), 134.0 (ArCH), 132.5 (ArC), 131.7 (ArC), 130.6 (ArCH), 129.4 (ArCH), 129.3 (ArCH), 128.9 (ArCH), 127.5 (ArCH), 122.7 (ArCH), 122.4 (ArCH), 121.7 (ArCH), 120.4 (ArCH). ν_{max} (neat)/cm^{-1} 755, 787, 837, 1220, 1357, 1376, 1520, 3010; HRMS (APCI): Calcd. for $C_{18}H_{12}OS$ (M+H$^+$), 277.0682; found 277.0682.

4-Phenyldibenzo[b,d]thiophene 5-oxide, 2aq

Prepared as described in General Procedure E, using 4-phenyldibenzo[b,d]thiophene (260 mg, 0.99 mmol). Purification by column chromatography on silica gel (gradient from 1% EtOAc in hexane to 30% EtOAc in hexane), afforded the title compound as a white solid (254 mg, 0.92 mmol, 92%); ^{1}H NMR (400 MHz, $CDCl_3$) δ 7.83-7.75 (m, 5H, ArH), 7.68 – 7.62 (m, 2H, ArH), 7.54 – 7.46 (m, 5H, ArH). ^{13}C NMR (101 MHz, $CDCl_3$) δ 140.6 (ArC), 138.2 (ArC), 136.5 (ArC), 135.7 (ArC), 133.8 (ArCH), 133.7 (ArCH), 132.6 (ArC), 132.5 (ArCH), 131.3 (ArC), 130.5 (ArCH), 129.4 (ArCH), 129.1 (ArCH), 128.7 (ArCH), 122.1 (ArCH), 121.5 (ArCH), 120.4 (ArCH). ν_{max} (neat)/cm^{-1} 786, 824, 837, 1240, 1370, 1450, 1510, 2980; HRMS (APCI): Calcd. for $C_{18}H_{12}OS$ (M+H$^+$), 277.0687; found 277.0682.

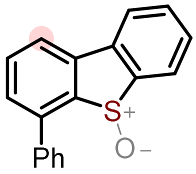

C–H type C4-arylation of benzothiophenes

1-(4-(3-Bromo-4-hydroxyphenyl)-2-methylbenzo[b]thiophen-3-yl)ethan-1-one, 4a

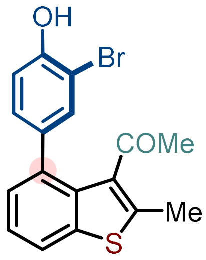

Prepared as described in General Procedure G, using 1-(2-methyl-1-oxidobenzo[b]thiophen-3-yl)ethan-1-one (103 mg, 0.49 mmol), 2-bromophenol (259 mg, 1.5 mmol), and TFAA (103 μL, 0.74 mmol, 1.5 equiv). Purification by column chromatography on silica gel (1% EtOAc in hexane), afforded the title compound as a white solid (136 mg, 0.38 mmol, 77%); ^{1}H NMR (400 MHz, $CDCl_3$) δ 7.75 (d, *J* = 7.9 Hz, 1H, Ar H), 7.52 (d, *J* = 2.2 Hz, 1H, Ar H), 7.37 (t, *J* = 7.7 Hz, 1H, Ar H), 7.29 – 7.23 (m, 2H, Ar H), 7.07 (d, *J* = 8.3 Hz, 1H, Ar H), 5.96 (br.s, 1H, OH), 2.52 (s, 3H, CH_3), 1.68 (s, 3H, CH_3). ^{13}C NMR (101 MHz, $CDCl_3$) δ 201.4 (C=O), 152.3 (ArC), 142.4 (ArC), 139.5 (ArC), 136.1 (ArC), 135.73 (ArC), 135.68 (ArC), 135.60 (ArC), 132.5 (ArCH), 129.9 (ArCH), 126.8 (ArCH), 124.6 (ArCH), 121.4 (ArCH), 116.4 (ArCH), 110.6 (ArC), 31.4 (CH_3), 14.9 (CH_3). ν_{max} (neat)/cm^{-1} 703, 753, 784, 1135, 1262, 1657, 2920, 3148; HRMS (ESI): Calcd. for $C_{17}H_{13}BrO_2SNa$ (M+Na$^+$), 382.9717; found 382.9711.

1-(4-Bromo-5a-methylbenzo[4,5]thieno[2,3-b]benzofuran-10b(5aH)-yl)ethan-1-one, 5a

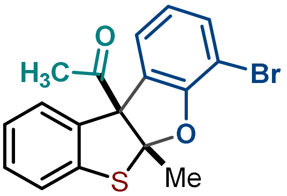

Prepared as described in General Procedure G, using 1-(2-methyl-1-oxidobenzo[b]thiophen-3-yl)ethan-1-one (103 mg, 0.49 mmol), 2-bromophenol (259 mg, 1.5 mmol), and TFAA (103 μL, 0.75 mmol, 1.5 equiv). Purification by column chromatography on silica gel (1% EtOAc in hexane), afforded **4a** in 77% yield and the title compound as a white solid (18 mg, 0.050 mmol, 10%); ^{1}H NMR (400 MHz, $CDCl_3$) δ 7.35 (dd, *J* = 8.0, 1.2 Hz, 1H, ArH), 7.29 (dd, *J* = 7.6, 1.2 Hz, 1H, ArH), 7.24 – 7.23 (m, 2H, ArH), 7.15 – 7.09 (m, 2H, ArH), 6.88 (t, *J* = 7.8 Hz, 1H, ArH), 2.14 (s, 3H, CH_3), 1.90 (s, 3H, CH_3). ^{13}C NMR (101 MHz, $CDCl_3$) δ 203.0 (C=O), 156.3 (ArC), 140.4 (ArC), 139.2 (ArC), 132.6 (ArCH), 129.5 (ArCH), 129.0 (ArC), 125.9 (ArCH), 125.3 (ArCH), 124.8 (ArCH), 123.3 (ArCH), 122.6 (ArCH), 109.6 (ArC), 103.4 (ArC), 80.3 (ArC), 28.7 (CH_3), 24.2 (CH_3). ν_{max} (neat)/cm^{-1} 732, 1264, 2927, 3054; HRMS (ESI): Calcd. for $C_{17}H_{13}BrO_2S$ (M+H$^+$), 360.9898; found 360.9892.

(4-(3-Bromo-4-hydroxyphenyl)-2-methylbenzo[b]thiophen-3-yl)(phenyl)methanone, 4b

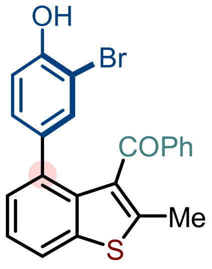

Prepared as described in General Procedure G, using (2-methyl-1-oxidobenzo[b]thiophen-3-yl)(phenyl)methanone (54 mg, 0.20 mmol), and 2-bromophenol (104 mg, 0.61 mmol). Purification by column chromatography on silica gel (3% EtOAc, 3% CH_2Cl_2 in hexane), afforded the title compound as a white solid (38 mg, 0.09 mmol, 45%) and corresponding minor C3 product (8.5 mg, 0.02 mmol, 10%); ^{1}H NMR (500 MHz, $CDCl_3$) δ 7.86 (dd, *J* = 8.3, 1.4 Hz, 2H, ArH), 7.81 (d, *J* = 2.1 Hz, 1H, ArH), 7.62 (t, *J* = 7.4 Hz, 1H, ArH), 7.57 (dd, *J* = 8.4, 2.1 Hz, 1H, ArH), 7.51 – 7.46 (m, 3H, ArH), 7.34 (d, *J* = 7.9 Hz, 1H, ArH), 7.27 – 7.25 (m, 1H, overlapped with $CDCl_3$, ArH), 7.15 (d, *J* = 8.3 Hz, 1H, ArH), 5.66 (s, 1H, OH), 2.48 (s, 3H, CH_3). ^{13}C NMR (126 MHz, $CDCl_3$) δ 193.6 (C=O), 152.3 (ArC), 146.0 (ArC), 139.9 (ArC), 138.7 (ArC), 137.2 (ArC), 134.5 (ArC), 133.5 (ArCH), 132.9 (ArC), 131.8 (ArCH), 129.9 (ArCH), 129.2 (ArCH), 128.9 (ArCH), 128.0 (ArC), 125.7 (ArCH), 124.4 (ArCH), 122.5 (ArCH), 116.5 (ArCH), 110.7 (ArC), 15.8 (CH_3) ppm. ν_{max} (neat)/cm^{-1} 733, 1288, 1501, 1714, 3057, 3379; HRMS (ESI): Calcd. for $C_{22}H_{15}BrO_2SNa$ (M+Na$^+$), 444.9874; found 444.9874.

(4-(3-Bromo-4-hydroxyphenyl)-2-methylbenzo[b]thiophen-3-yl)(4-methoxyphenyl)methanone, 4c

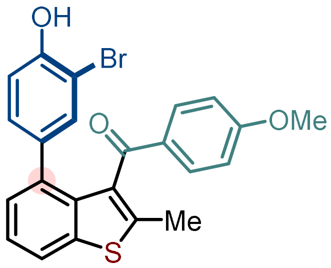

Prepared as described in General Procedure G, using (4-methoxyphenyl)(2-methyl-1-oxidobenzo[b]thiophen-3-yl)methanone (60 mg, 0.20 mmol), and 2-bromophenol (104 mg, 0.61 mmol). Purification by column chromatography on silica gel (3% EtOAc, 3% CH_2Cl_2 in hexane), afforded the title compound as a white solid (36 mg, 0.08 mmol, 40%) and corresponding minor C3 product (14 mg, 0.03 mmol, 16%); ^{1}H NMR (400 MHz, Acetone-d_6) δ 9.24 (s, 1H, OH), 7.87 (d, *J* = 2.2 Hz, 1H, ArH), 7.86 – 7.82 (m, 2H, ArH), 7.59 (dd, *J* = 8.4, 2.3 Hz, 1H, ArH), 7.46 – 7.35 (m, 3H, ArH), 7.20 (d, *J* = 8.3 Hz, 1H, ArH), 7.10 – 7.05 (m, 2H, ArH), 3.92 (s, 3H, OCH_3), 2.48 (s, 3H, CH_3). ^{13}C NMR (101 MHz, $CDCl_3$) δ 192.3 (C=O), 164.1 (ArC), 152.3 (ArC), 144.4 (ArC), 140.1 (ArC), 134.6 (ArC), 134.5 (ArC), 133.4 (ArC), 132.4 (ArCH), 131.77 (ArCH), 131.4 (ArC), 129.2 (ArCH), 125.6 (ArCH), 124.3 (ArCH), 122.4 (ArCH), 116.5 (ArCH), 114.1 (ArCH), 110.7 (ArC), 55.7 (OCH_3), 15.6 (CH_3). ν_{max} (neat)/cm^{-1} 691,

754, 1174, 1461, 1640, 2919, 3309; HRMS (ESI): Calcd. for $C_{23}H_{17}BrO_3SNa$ (M+Na$^+$), 474.9979; found 474.9970.

XRD: Single crystal analysis confirms the structure.

4-(3-Bromo-4-hydroxyphenyl)-2-methylbenzo[b]thiophene-3-carbaldehyde, 4d

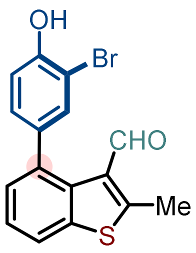

Prepared as described in General Procedure G, using 2-methylbenzo[b]thiophene-3-carbaldehyde 1-oxide (38 mg, 0.20 mmol), and 2-bromophenol (104 mg, 0.61 mmol). Purification by column chromatography on silica gel (5% EtOAc in hexane to 10% EtOAc in hexane), afforded the title compound as a white solid (43 mg, 0.124 mmol, 62%); ^{1}H NMR (400 MHz, $CDCl_3$) δ 9.29 (s, 1H, CHO), 7.75 (dd, *J* = 8.0, 1.1 Hz, 1H, ArH), 7.38 (t, *J* = 7.7 Hz, 1H, ArH), 7.29 (d, *J* = 6.5 Hz, 2H, ArH), 6.93 – 6.85 (m, 2H, ArH), 5.69 (s, 1H, OH), 2.81 (s, 3H, CH_3). ^{13}C NMR (101 MHz, $CDCl_3$) δ 187.5 (C=O), 153.9 (ArC), 152.4 (ArC), 138.2 (ArC), 136.9 (ArC), 135.9 (ArC), 135.6 (ArC), 132.1 (ArCH), 130.8 (ArC), 129.6 (ArCH), 127.6 (ArCH), 124.5 (ArCH), 121.6 (ArCH), 116.8 (ArCH), 110.9 (ArC), 17.1 (CH_3). ν_{max} (neat)/cm^{-1} 643, 709, 1040, 1136, 1205, 1252, 1649, 2850, 2919, 3221; HRMS (ESI): Calcd. for $C_{16}H_{11}BrO_2SNa$ (M+Na$^+$), 368.9561; found 368.9554.

1-(4-(4-Hydroxyphenyl)-2-methylbenzo[b]thiophen-3-yl)ethan-1-one, 4e

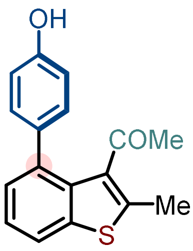

Prepared as described in General Procedure G, using 1-(2-methyl-1-oxidobenzo[b]thiophen-3-yl)ethan-1-one (41 mg, 0.20 mmol), and phenol (56.5 mg, 0.60 mmol). Purification by column chromatography on silica gel (7% EtOAc in hexane), afforded the title compound as a white solid (28 mg, 0.1 mmol, 50%) and corresponding minor C3 product (17 mg, 0.06 mmol, 31%); ^{1}H NMR (400 MHz, $CDCl_3$) δ 7.73 (dd, *J* = 7.9, 1.2 Hz, 1H, ArH), 7.38 (t, *J* = 7.6 Hz, 1H, ArH), 7.33-7.29 (m, 3H, ArH), 6.92 (d, *J* = 8.6 Hz, 2H, Ar H), 6.84 (br.s, 1H, OH), 2.53 (s, 3H, CH_3), 1.66 (s, 3H, CH_3). ^{13}C NMR (101 MHz, $CDCl_3$) δ 203.7 (C=O), 156.7 (ArC), 142.9 (ArC), 139.4 (ArC), 137.5 (ArC), 135.9 (ArC), 135.9 (ArC), 133.9 (ArC), 130.3 (ArCH), 126.8 (ArCH), 124.8 (ArCH), 120.8 (ArCH), 115.9 (ArCH), 31.2 (CH_3), 14.9 (CH_3). ν_{max} (neat)/cm^{-1} 755, 787, 825, 1182, 1212, 1326, 1561, 1658, 2850, 2919, 3401; HRMS (ESI): Calcd. for $C_{17}H_{13}O_2S$ (M-H$^+$), 281.0636; found 281.0635.

XRD: Single crystal analysis confirms the structure.

4-(4-Hydroxyphenyl)-2-methylbenzo[b]thiophene-3-carbaldehyde, 4f

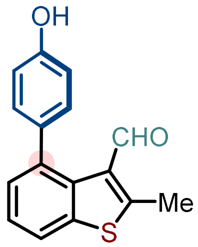

Prepared as described in General Procedure G, using 2-methylbenzo[b]thiophene-3-carbaldehyde 1-oxide (38 mg, 0.2 mmol), and phenol (94.1 mg, 0.99 mmol). Purification by column chromatography on silica gel (5% EtOAc in hexane), afforded the title compound as a white solid (21 mg, 0.08 mmol, 40%); ^{1}H NMR (400 MHz, $CDCl_3$) δ 9.29 (s, 1H, CHO), 7.75 (dd, *J* = 8.0, 1.1 Hz, 1H, ArH), 7.37 (d, *J* = 7.7 Hz, 1H, ArH), 7.31 – 7.26 (m, 3H, ArH), 6.92 – 6.85 (m, 2H, ArH), 5.69 (s, 1H, OH), 2.81 (s, 3H, CH_3). ^{13}C NMR (101 MHz, $CDCl_3$) δ 188.49 (CHO), 155.9 (C-OH), 153.6 (ArC), 138.1 (ArC), 137.3 (ArC), 137.2 (ArC), 134.5 (ArC), 131.0 (ArC), 130.0 (ArCH), 127.5 (ArCH), 124.5 (ArCH), 121.0 (ArCH), 116.2 (ArCH), 17.2 (CH_3). ν_{max} (neat)/cm^{-1} 655, 755, 1106, 1340, 1394, 1599, 2955, 3389; HRMS (ESI): Calcd. for $C_{16}H_{12}O_2S$ ($M+H^+$), 269.0636; found 269.0631.

1-(4-(4-Hydroxy-3-iodophenyl)-2-methylbenzo[b]thiophen-3-yl)ethan-1-one, 4g

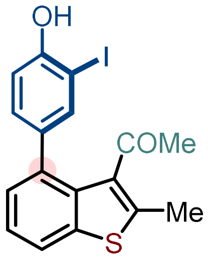

Prepared as described in General Procedure G, using 1-(2-methyl-1-oxidobenzo[b]thiophen-3-yl)ethan-1-one (41 mg, 0.20 mmol), and 2-iodophenol (132 mg, 0.60 mmol). Purification by column chromatography on silica gel (7% EtOAc in hexane), afforded the title compound as a white solid (49 mg, 0.12 mmol, 60%) and corresponding minor C3 product (13 mg, 0.03 mmol, 16%); ^{1}H NMR (400 MHz, $(CD_3)_2CO$) δ 7.88 (dd, *J* = 8.0, 1.1 Hz, 1H, ArH), 7.71 (d, *J* = 2.2 Hz, 1H, ArH), 7.43 (t, *J* = 7.7 Hz, 1H, ArH), 7.31 (dd, *J* = 7.4, 1.1 Hz, 1H, ArH), 7.24 (dd, *J* = 8.2, 2.2 Hz, 1H, ArH), 7.01 (d, *J* = 8.3 Hz, 1H, ArH), 2.48 (s, 3H, CH_3), 1.66 (s, 3H, CH_3). ^{13}C NMR (101 MHz, $(CD_3)_2CO$) δ 200.0 (C=O), 157.3 (ArC), 141.9 (ArC), 140.3 (ArCH), 140.1 (ArC), 137.4 (ArC), 136.6 (ArC), 136.4 (ArC), 136.0 (ArC), 131.7 (ArCH), 127.6 (ArCH), 125.4 (ArCH), 122.0 (ArCH), 115.7 (ArCH), 84.5 (ArC), 31.3 (CH_3), 14.6 (CH_3). δ ν_{max} (neat)/cm^{-1} 783, 801, 1208, 1283, 1659, 1678, 2918, 3052; HRMS (ESI): Calcd. for $C_{17}H_{13}IO_2SNa$ ($M+Na^+$), 430.9579; found 430.9573.

1-(4-(3,5-Dichloro-4-hydroxyphenyl)-2-methylbenzo[b]thiophen-3-yl)ethan-1-one, 4h

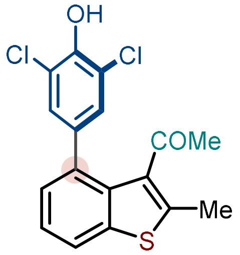

Prepared as described in General Procedure G, using 1-(2-methyl-1-oxidobenzo[b]thiophen-3-yl)ethan-1-one (41 mg, 0.20 mmol), and 2,6-dichlorophenol (98 mg, 0.60 mmol). Purification by column chromatography on silica gel (3% EtOAc, 3% CH_2Cl_2 in hexane), afforded the title compound as a white solid (36 mg, 0.10 mmol, 51%); ^{1}H NMR (400 MHz, $CDCl_3$) δ 7.77 (dd, *J* = 8.0, 1.1 Hz, 1H, ArH), 7.37 (t, *J* = 7.7 Hz, 1H, ArH), 7.31 (s, 2H, ArH), 7.28 – 7.25 (m, 1H, ArH), 5.96 (br.s, 1H, OH), 2.53 (s, 3H, CH_3), 1.78 (s, 3H, CH_3). ^{13}C NMR (101 MHz, Acetone-d_6) δ 200.1 (C=O), 149.7 (ArC), 142.2 (ArC), 140.2 (ArC), 137.1 (ArC), 136.3 (ArC), 135.5 (ArC), 135.3 (ArC), 129.8 (ArCH), 127.6 (ArCH), 125.4 (ArCH), 122.9 (ArC), 122.6 (ArCH), 31.5 (CH_3), 14.6 (CH_3). ν_{max} (neat)/cm^{-1} 648, 778, 1153, 1295, 1325, 1667, 2921, 3321; HRMS (ESI): Calcd. for $C_{17}H_{12}Cl_2O_2SNa$ (M+Na$^+$), 372.9833; found 372.9827.

1-(4-(3-Benzyl-4-hydroxyphenyl)-2-methylbenzo[b]thiophen-3-yl)ethan-1-one, 4i

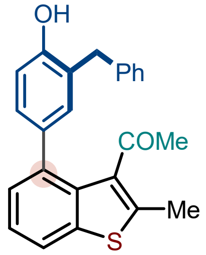

Prepared as described in General Procedure G, using 1-(2-methyl-1-oxidobenzo[b]thiophen-3-yl)ethan-1-one (41 mg, 0.20 mmol), and 2-benzylphenol (111 mg, 0.60 mmol). Purification by column chromatography on silica gel (5% EtOAc in hexane to 10% EtOAc in hexane), afforded the title compound as a white solid (41 mg, 0.11 mmol, 55%) and corresponding minor C3 product (10 mg, 0.03 mmol, 14%); ^{1}H NMR (500 MHz, $CDCl_3$) δ 7.71 (dd, *J* = 8.0, 1.1 Hz, 1H, ArH), 7.35 (t, *J* = 7.6 Hz, 1H, ArH), 7.28 – 7.24 (m, 5H, ArH), 7.20 (d, *J* = 2.3 Hz, 1H, ArH), 7.18 – 7.14 (m, 1H, ArH), 7.10 (dd, *J* = 8.2, 2.3 Hz, 1H, ArH), 6.84 (d, *J* = 8.2 Hz, 1H, ArH), 4.03 (s, 2H, CH_2), 2.51 (s, 3H, CH_3), 1.61 (s, 3H, CH_3). ^{13}C NMR (126 MHz, $CDCl_3$) δ 202.4 (C=O), 154.3 (ArC), 142.4 (ArC), 140.2 (ArC), 139.3 (ArC), 137.5 (ArC), 136.2 (ArC), 135.9 (ArC), 134.3 (ArC), 131.5 (ArCH), 128.9 (ArCH), 128.7 (ArCH), 128.6 (ArCH), 128.2 (ArC), 126.8 (ArCH), 126.4 (ArCH), 124.6 (ArCH), 120.8 (ArCH), 116.0 (ArCH), 36.5 (CH_2), 31.2 (CH_3), 14.9 (CH_3). ν_{max} (neat)/cm^{-1} 695, 723, 1111, 1267, 1401, 1664, 2919, 3398; HRMS (ESI): Calcd. for $C_{24}H_{20}O_2SNa$ (M+Na$^+$), 395.1082; found 395.1076.

1-(4-(3-Allyl-4-hydroxyphenyl)-2-methylbenzo[b]thiophen-3-yl)ethan-1-one, 4j

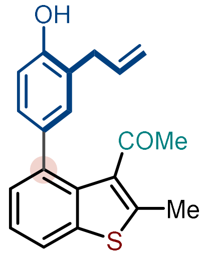

Prepared as described in General Procedure G, using 1-(2-methyl-1-oxidobenzo[b]thiophen-3-yl)ethan-1-one (41 mg, 0.20 mmol), and 2-allylphenol (81 mg, 0.60 mmol). Purification by column chromatography on silica gel (5% EtOAc in hexane to 20% EtOAc in hexane), afforded the title compound as a white solid (32 mg, 0.09 mmol, 50%) and corresponding minor C3 product (10 mg, 0.03 mmol, 16%); ^{1}H NMR (400 MHz, Acetone-d_6) δ 7.84 (dd, J = 8.0, 1.1 Hz, 1H, ArH), 7.40 (t, J = 7.7 Hz, 1H, ArH), 7.30 (dd, J = 7.4, 1.1 Hz, 1H, ArH), 7.09 (d, J = 7.9 Hz, 2H, ArH), 6.92 (d, J = 7.9 Hz, 1H, ArH), 6.09 – 5.99 (m, 1H, CH_2-C*H*=CH_2), 5.10 (dq, J = 17.1, 1.8 Hz, 1H, CH_2-CH=CH_2), 5.00 (ddt, J = 10.0, 2.3, 1.3 Hz, 1H, CH_2-CH=CH_2), 3.40 (d, J = 6.7 Hz, 2H, CH_2-CH=CH_2), 2.46 (s, 3H, CH_3), 1.54 (s, 3H, CH_3). ^{13}C NMR (101 MHz, Acetone-d_6) δ 200.1 (C=O), 155.6 (ArC), 141.6 (ArC), 140.0 (ArC), 138.5 (ArC), 137.7 (ArC), 137.6 (=CH), 136.6 (ArC), 134.2 (ArC), 131.6 (ArCH), 128.7 (ArCH), 127.7 (ArC), 127.4 (ArCH), 125.4 (ArCH), 121.4 (ArCH), 116.1 (ArCH), 115.8 (=CH_2), 34.9 (CH_2), 31.1 (CH_3), 14.9 (CH_3). ν_{max} (neat)/cm^{-1} 646, 750, 778, 1114, 1214, 1458, 1671, 2920, 3375; HRMS (ESI): Calcd. for $C_{20}H_{18}O_2SNa$ (M+Na$^+$), 345.0920; found 345.0920.

4-(3-Allyl-4-hydroxyphenyl)-2-methylbenzo[b]thiophene-3-carbaldehyde, 4k

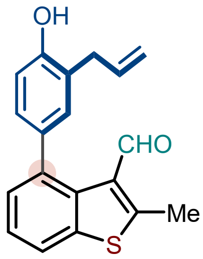

Prepared as described in General Procedure G, using 2-methylbenzo[b]thiophene-3-carbaldehyde 1-oxide (38 mg, 0.2 mmol), and 2-allylphenol (134 mg, 0.99 mmol). Purification by column chromatography on silica gel (5% EtOAc in hexane to 20% EtOAc in hexane), afforded the title compound as a white solid (31 mg, 0.10 mmol, 50%); ^{1}H NMR (400 MHz, $CDCl_3$) δ 9.27 (s, 1H, CHO), 7.74 (dd, J = 7.9, 1.2 Hz, 1H, ArH), 7.37 (t, J = 7.7 Hz, 1H, ArH), 7.29 (dd, J = 7.3, 1.2 Hz, 1H, ArH), 7.21 – 7.15 (m, 2H, ArH), 6.88 (d, J = 8.8 Hz, 1H, ArH), 6.05 – 5.97 (m, 1H, CH_2-C*H*=CH_2), 5.36 (s, 1H, OH), 5.22 – 5.16 (m, 2H, CH_2-CH=CH_2), 3.44 (dt, J = 6.4, 1.7 Hz, 2H, CH_2-CH=CH_2), 2.80 (s, 3H, CH_3). ^{13}C NMR (101 MHz, $CDCl_3$) δ 188.2 (C=O), 154.2 (ArC), 153.1 (ArC), 138.1 (ArC), 137.3 (ArC), 137.2 (ArC), 136.1 (=CH), 134.8 (ArC), 131.2 (ArC), 130.8 (ArCH), 128.1 (ArCH), 127.4 (ArCH), 126.4 (ArC), 124.4 (ArCH), 121.0 (ArCH), 116.9 (=CH_2), 116.5 (ArCH), 35.1 (CH_2), 17.1 (CH_3). ν_{max} (neat)/cm^{-1} 642, 780, 1107, 1155, 1217, 1453, 1654, 2921, 3256; HRMS (APCI): Calcd. for $C_{19}H_{17}O_2S$ (M+H$^+$), 309.0949; found 309.0944.

4-(3-Benzoyl-4-hydroxyphenyl)-2-methylbenzo[b]thiophene-3-carbaldehyde, 4l

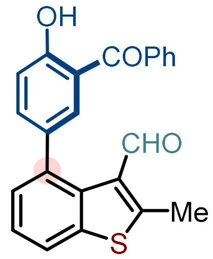

Prepared as described in General Procedure G, using 2-methylbenzo[b]thiophene-3-carbaldehyde 1-oxide (38 mg, 0.20 mmol), and 2-hydroxybenzophenone (198 mg, 0.99 mmol). Purification by column chromatography on silica gel (5% EtOAc in hexane to 20% EtOAc in hexane), afforded the title compound as a white solid (41 mg, 0.11 mmol, 55%); ^{1}H NMR (400 MHz, $CDCl_3$) δ 12.10 (s, 1H, OH), 9.50 (s, 1H, CHO), 7.76 (dd, *J* = 8.0, 1.1 Hz, 1H, ArH), 7.67 – 7.62 (m, 3H, ArH), 7.59 – 7.52 (m, 2H, ArH), 7.48 – 7.44 (m, 2H, ArH), 7.35 (t, *J* = 7.7 Hz, 1H, ArH), 7.23 (dd, *J* = 7.4, 1.1 Hz, 1H, ArH), 7.19 (d, *J* = 8.5 Hz, 1H, ArH), 2.82 (s, 3H, CH_3). ^{13}C NMR (101 MHz, $CDCl_3$) δ 201.6 (PhC=O), 187.2 (HC=O), 163.1 (ArC), 153.9 (ArC), 138.3 (ArC), 137.7 (ArC), 136.9 (ArC), 136.7 (ArCH), 135.9 (ArC), 133.1 (ArCH), 132.9 (ArC), 132.2 (ArCH), 130.8 (ArC), 129.2 (ArCH), 128.6 (ArCH), 127.7 (ArCH), 124.5 (ArCH), 121.5 (ArCH), 119.5 (ArCH), 119.4 (ArC), 17.1 (CH_3). ν_{max} (neat)/cm^{-1} 642, 661, 690, 711, 1175, 1287, 1448, 1503, 1576, 2865, 2977; HRMS (APCI): Calcd. for $C_{23}H_{16}O_3SNa$ (M+Na$^+$), 395.0718; found 395.0721.

4-(5-Bromo-4-hydroxy-2-methylphenyl)-2-methylbenzo[b]thiophene-3-carbaldehyde, 4m

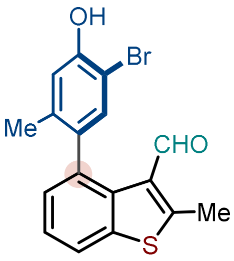

Prepared as described in General Procedure G, using 2-methylbenzo[b]thiophene-3-carbaldehyde 1-oxide (38 mg, 0.20 mmol), and 2-bromo-5-methylphenol (187 mg, 0.99 mmol). Purification by column chromatography on silica gel (5% EtOAc in hexane to 20% EtOAc in hexane), afforded the title compound as a white solid (43 mg, 0.12 mmol, 60%); ^{1}H NMR (400 MHz, $CDCl_3$) δ 9.20 (s, 1H, CHO), 7.79 (dd, *J* = 8.1, 1.1 Hz, 1H, ArH), 7.38 (t, *J* = 7.8 Hz, 1H, ArH), 7.36 (s, 1H, ArH), 7.19 (dd, *J* = 7.4, 1.1 Hz, 1H, ArH), 6.97 (s, 1H, ArH), 5.56 (br.s, 1H, OH), 2.82 (s, 3H, CH_3), 1.97 (s, 3H, CH_3). ^{13}C NMR (101 MHz, $CDCl_3$) δ 187.7 (C=O), 154.2 (ArC), 152.2 (ArC), 137.8 (ArC), 137.6 (ArC), 137.2 (ArC), 135.5 (ArC), 134.8 (ArC), 132.4 (ArCH), 131.1 (ArC), 127.9 (ArCH), 124.4 (ArCH), 121.6 (ArCH), 117.9 (ArCH), 107.7 (ArC), 19.9 (CH_3), 17.4 (CH_3). ν_{max} (neat)/cm^{-1} 644, 772, 1156, 1271, 1695, 2781, 2919, 3058; HRMS (APCI): Calcd. for $C_{17}H_{14}BrO_2S$ (M+H$^+$), 360.9898; found 360.9892.

2-(4-Bromophenyl)-4-(4-hydroxyphenyl)benzo[b]thiophene-3-carbaldehyde, 4n

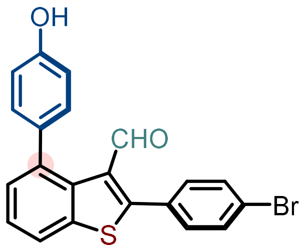

Prepared as described in General Procedure G, using 2-(4-bromophenyl)benzo[b]thiophene-3-carbaldehyde 1-oxide (67 mg, 0.20 mmol), and phenol (57 mg, 0.60 mmol). Purification by column chromatography on silica gel (10% EtOAc in hexane), afforded the title compound as a white solid (47 mg, 0.11 mmol, 58%); ^{1}H NMR (400 MHz, $CDCl_3$) δ 9.35 (s, 1H, CHO), 7.83 (dd, *J* = 8.0, 1.2 Hz, 1H, ArH), 7.56 (d, *J* = 8.6 Hz, 2H, ArH), 7.47 (d, *J* = 8.3 Hz, 3H, ArH), 7.37 (dd, *J* = 7.4, 1.2 Hz, 1H, ArH), 7.30 (d, *J* = 8.5 Hz, 2H, ArH), 6.89 (d, *J* = 8.5 Hz, 2H, ArH). ^{13}C NMR (101 MHz, $CDCl_3$) δ 186.7 (C=O), 156.0 (ArC), 151.8 (ArC), 139.5 (ArC), 138.5 (ArC), 137.0 (ArC), 134.3 (ArC), 132.1 (ArCH), 131.8 (ArC), 131.5 (ArCH), 131.0 (ArC), 130.1 (ArCH), 127.9 (ArCH), 125.5 (ArCH), 124.2 (ArC), 121.0 (ArCH), 116.1 (ArCH). ν_{max} (neat)/cm^{-1} 710, 750, 781, 814, 1084, 1102, 1179, 1265, 1474, 1778, 1896, 2854, 2922, 3422; HRMS (ESI): Calcd. for $C_{21}H_{13}BrO_2SNa$ (M+Na$^+$), 430.9717; found 430.9711.

XRD: Single crystal analysis confirms the structure.

1-(4-(3-Bromo-4-hydroxyphenyl)-2-(4-bromophenyl)benzo[b]thiophen-3-yl)ethan-1-one, 4o

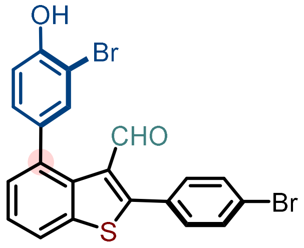

Prepared as described in General Procedure G, using 2-(4-bromophenyl)benzo[b]thiophene-3-carbaldehyde 1-oxide (67 mg, 0.20 mmol), and 2-bromophenol (104 mg, 0.60 mmol). Purification by column chromatography on silica gel (10% EtOAc in hexane), afforded the title compound as a light yellow solid (64 mg, 0.13 mmol, 65%); ^{1}H NMR (400 MHz, $CDCl_3$) δ 9.45 (s, 1H, CHO), 7.85 (d, *J* = 8.1 Hz, 1H, ArH), 7.60 – 7.54 (m, 3H, ArH), 7.47-7.45 (m, 3H, ArH), 7.35 (d, *J* = 7.3 Hz, 1H, ArH), 7.29 (dd, *J* = 8.2, 2.1 Hz, 1H, ArH), 7.10 (d, *J* = 8.3 Hz, 1H, ArH), 5.63 (br.s, 1H, OH). ^{13}C NMR (101 MHz, $CDCl_3$) δ 186.1 (C=O), 152.8 (ArC), 152.3 (ArC), 139.6 (ArC), 136.9 (ArC), 136.4 (ArC), 135.9 (ArC), 132.1 (ArCH), 132.0 (ArC), 131.6 (ArCH), 131.5 (ArC), 131.4 (ArC), 129.6 (ArCH), 128.1 (ArCH), 125.4 (ArCH), 124.3 (ArC), 121.5 (ArCH), 116.6 (ArCH), 110.8 (ArC). ν_{max} (neat)/cm^{-1} 711, 751, 785, 818, 1039, 1136, 1240, 1448, 1475 1666, 2852, 2921, 3292; HRMS (ESI): Calcd. for $C_{21}H_{12}Br_2O_2SNa$ (M+Na$^+$), 510.8802; found 510.8798.

1-(2-(4-Bromophenyl)-4-(4-hydroxy-3-nitrophenyl)benzo[b]thiophen-3-yl)ethan-1-one, 4p

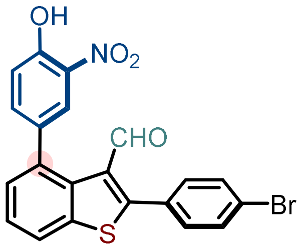

Prepared as described in General Procedure G, using 2-(4-bromophenyl)benzo[b]thiophene-3-carbaldehyde 1-oxide (67 mg, 0.20 mmol), and 2-nitrophenol (84 mg, 0.60 mmol). Purification by column chromatography on silica gel (10% EtOAc in hexane), afforded the title compound as a yellow solid (55 mg, 0.12 mmol, 60%); ^{1}H NMR (400 MHz, $CDCl_3$) δ 10.64 (s, 1H, OH), 9.60 (s, 1H, CHO), 8.13 (d, *J* = 2.2 Hz, 1H, ArH), 7.91 (dd, *J* = 8.1, 1.1 Hz, 1H, ArH), 7.66 – 7.60 (m, 3H, ArH), 7.52 (t, *J* = 7.7 Hz, 1H, ArH), 7.47 (d, *J* = 8.5 Hz, 2H, ArH), 7.39 (dd, *J* = 7.4, 1.2 Hz, 1H, ArH), 7.23 (d, *J* = 8.7 Hz, 1H, ArH). ^{13}C NMR (101 MHz, $CDCl_3$) δ 185.4 (C=O), 156.2 (ArC), 154.6 (ArC), 139.8 (ArC), 137.9 (ArCH), 136.3 (ArC), 135.1 (ArC), 135.1 (ArC), 133.5 (ArC), 132.1 (ArCH), 131.9 (ArCH), 131.6 (ArC), 131.0 (ArC), 128.7 (ArCH), 125.8 (ArCH), 124.8 (ArC), 124.4 (ArCH), 122.0 (ArCH), 120.2 (ArCH). ν_{max} (neat)/cm^{-1} 687, 789, 1183, 1352, 1395, 1570, 1678, 1782, 3274; HRMS (ESI): Calcd. for $C_{21}H_{12}BrNO_4SNa$ $(M+Na^+)$, 475.9568; found 475.9563.

4-(3-Acetyl-4-hydroxyphenyl)-2-(4-bromophenyl)benzo[b]thiophene-3-carbaldehyde, 4q

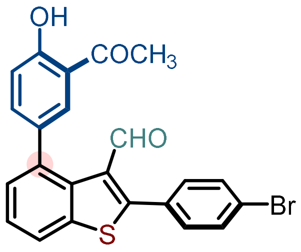

Prepared as described in General Procedure G, using 2-(4-bromophenyl)benzo[b]thiophene-3-carbaldehyde 1-oxide (67 mg, 0.20 mmol), and 2-acetylphenol (82 mg, 0.60 mmol). Purification by column chromatography on silica gel (5% EtOAc in hexane to 20% EtOAc in hexane), afforded the title compound as a white solid (50 mg, 0.11 mmol, 55%); ^{1}H NMR (400 MHz, $CDCl_3$) *δ* ^{1}H NMR (400 MHz, $CDCl_3$) δ 12.33 (s, 1H, OH), 9.49 (s, 1H, CHO), 7.88 (dd, *J* = 8.1, 1.1 Hz, 1H, ArH), 7.79 (d, *J* = 2.2 Hz, 1H, ArH), 7.59 – 7.45 (m, 6H, ArH), 7.38 (dd, *J* = 7.4, 1.1 Hz, 1H, ArH), 7.09 (d, *J* = 8.5 Hz, 1H, ArH), 2.62 (s, 3H, CH_3). ^{13}C NMR (101 MHz, $CDCl_3$) δ 204.6 (CH_3C=O), 186.3 (HC=O), 162.2 (ArC), 152.7 (ArC), 139.8 (ArC), 137.4 (ArC), 136.8 (ArCH), 136.4 (ArC), 132.9 (ArC), 132.1 (ArCH), 131.7 (ArCH), 131.6 (ArC), 131.5 (ArC), 130.6 (ArCH), 128.1 (ArCH), 125.6 (ArCH), 124.4 (ArC), 121.5 (ArCH), 119.7 (ArC), 119.3 (ArCH), 26.9 (CH_3). ν_{max} (neat)/cm^{-1} 638, 742, 776, 1181, 1615, 1638, 2770, 2850, 2919; HRMS (APCI): Calcd. for $C_{23}H_{16}BrO_3S$ $(M+H^+)$, 452.9983; found 452.9980.

Methyl 5-(2-(4-bromophenyl)-3-formylbenzo[b]thiophen-4-yl)-2-hydroxybenzoate, 4r

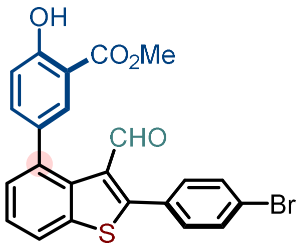

Prepared as described in General Procedure G, using 2-(4-bromophenyl)benzo[b]thiophene-3-carbaldehyde 1-oxide (67 mg, 0.20 mmol), and methyl salicylate (92 mg, 0.60 mmol). Purification by column chromatography on silica gel (5% EtOAc in hexane to 20% EtOAc in hexane), afforded the title compound as a white solid (64 mg, 0.14 mmol, 68%); ^{1}H NMR (400 MHz, $CDCl_3$) δ 10.84 (s, 1H, OH), 9.47 (s, 1H, CHO), 7.91 (d, *J* = 2.3 Hz, 1H, ArH), 7.86 (dd, *J* = 8.0, 1.1 Hz, 1H, ArH), 7.57 (d, *J* = 8.6 Hz, 2H, ArH), 7.53 – 7.44 (m, 4H, ArH), 7.36 (dd, *J* = 7.3, 1.1 Hz, 1H, ArH), 7.08 (d, *J* = 8.6 Hz, 1H, ArH), 3.94 (s, 3H, OCH_3). ^{13}C NMR (101 MHz, $CDCl_3$) δ 185.9 (HC=O), 170.5 (MeOC=O), 161.4 (ArC), 152.8 (ArC), 139.6 (ArC), 137.5 (ArC), 136.4 (ArC), 136.1 (ArCH), 133.2 (ArC), 132.1 (ArCH), 131.6 (ArCH), 131.5 (ArC), 129.8 (ArCH), 128.3 (ArCH), 125.5 (ArCH), 124.3 (ArC), 121.4 (ArCH), 118.4 (ArCH), 112.7 (ArC), 52.6 (OCH_3). ν_{max} (neat)/cm^{-1} 696, 733, 781, 792, 1010, 1102, 1208, 1242, 1436, 1676, 2849, 2919, 2959, 3090; HRMS (APCI): Calcd. for $C_{23}H_{16}BrO_4S$ ($M+H^+$), 466.9953; found 466.9958.

Methyl 4-(4-hydroxyphenyl)-2-methylbenzo[b]thiophene-3-carboxylate, 4s

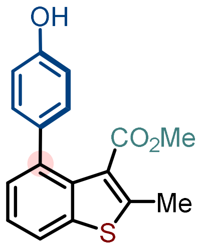

Prepared as described in General Procedure G, using methyl 2-methylbenzo[b]thiophene-3-carboxylate 1-oxide (45 mg, 0.20 mmol), and phenol (57 mg, 0.60 mmol). Purification by column chromatography on silica gel (5% EtOAc in hexane), afforded the title compound as a white solid (24 mg, 0.08 mmol, 40%) and corresponding minor C3 product (23 mg, 0.08 mmol, 38%); ^{1}H NMR (400 MHz, DMSO-d_6) δ 9.51 (s, 1H, OH), 7.89 (dd, *J* = 8.0, 1.1 Hz, 1H, ArH), 7.39 (t, *J* = 7.7 Hz, 1H, ArH), 7.21 (dd, *J* = 7.4, 1.1 Hz, 1H, ArH), 7.08 (d, *J* = 8.5 Hz, 2H, ArH), 6.81 (d, *J* = 8.5 Hz, 2H, ArH), 3.33 (s, 3H, OCH_3), 3.09 (s, 3H, CH_3). ^{13}C NMR (101 MHz, DMSO-d_6) δ 165.1 (C=O), 156.6 (ArC), 143.7 (ArC), 138.3 (ArC), 137.3 (ArC), 134.7 (ArC), 132.2 (ArC), 128.9 (ArCH), 126.8 (ArCH), 126.4 (ArC), 124.4 (ArCH), 120.7 (ArCH), 115.1 (ArCH), 51.2 (OCH_3), 14.5 (CH_3). ν_{max} (neat)/cm^{-1} 758, 830, 862, 1248, 1452, 1508, 1607, 1688, 2509, 3383; HRMS (ESI): Calcd. for $C_{17}H_{14}O_3SNa$ ($M+Na^+$), 321.0561; found 321.0554.

Methyl 4-(3-bromo-4-hydroxyphenyl)-2-methylbenzo[b]thiophene-3-carboxylate, 4t

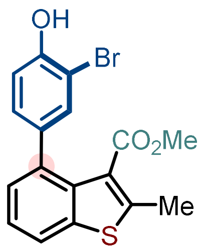

Prepared as described in General Procedure G, using methyl 2-methylbenzo[b]thiophene-3-carboxylate 1-oxide (45 mg, 0.20 mmol), and 2-bromophenol (104 mg, 0.60 mmol). Purification by column chromatography on silica gel (5% EtOAc in hexane), afforded the title compound as a white solid (31 mg, 0.082 mmol, 41%) and corresponding minor C3 product (11 mg, 0.03 mmol, 14%); ^{1}H NMR (400 MHz, $CDCl_3$) δ 7.73 (dd, *J* = 8.0, 1.1 Hz, 1H, ArH), 7.50 (d, *J* = 2.1 Hz, 1H, ArH), 7.35 (t, *J* = 7.7 Hz, 1H, ArH), 7.25 – 7.22 (m, 2H, ArH), 7.08 (d, *J* = 8.3 Hz, 1H, ArH), 5.62 (br.s, 1H, OH), 3.25 (s, 3H, OCH_3), 2.62 (s, 3H, CH_3). ^{13}C NMR (101 MHz, $CDCl_3$) δ 166.1 (C=O), 151.5 (ArC), 145.5 (ArC), 139.2 (ArC), 136.2 (ArC), 135.7 (ArC), 135.3 (ArC), 131.6 (ArCH), 129.2 (ArCH), 127.2 (ArCH), 126.2 (ArC), 124.3 (ArCH), 121.3 (ArCH), 116.0 (ArCH), 110.1 (ArC), 51.7 (OCH_3), 15.1 (CH_3). ν_{max} (neat)/cm^{-1} 609, 722, 755, 774, 977, 1039, 1284, 1689, 2849, 2920, 3314; HRMS (ESI): Calcd. for $C_{17}H_{13}BrO_3SNa$ (M+Na$^+$), 398.9666; found 398.9661.

Dimethyl 4-(3-bromo-4-hydroxyphenyl)benzo[b]thiophene-2,3-dicarboxylate, 4u

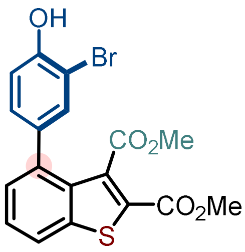

Prepared as described in General Procedure G, using dimethyl benzo[b]thiophene-2,3-dicarboxylate 1-oxide (53 mg, 0.20 mmol), and 2-bromophenol (104 mg, 0.60 mmol). Purification by column chromatography on silica gel (5% EtOAc in hexane to 20% EtOAc in hexane), afforded the title compound as a white solid (38 mg, 0.09 mmol, 45%); ^{1}H NMR (500 MHz, $CDCl_3$) δ 7.86 (dd, *J* = 8.2, 1.0 Hz, 1H, ArH), 7.50 (7.50 (t, *J* = 7.7 Hz, 1H, ArH), 7.47 (d, *J* = 2.1 Hz, 1H, ArH), 7.26 – 7.21 (m, 2H, ArH), 7.07 (d, *J* = 8.2 Hz, 1H, ArH), 5.66 (br.s, 1H, OH), 3.90 (s, 3H, OCH_3), 3.48 (s, 3H, OCH_3). ^{13}C NMR (126 MHz, $CDCl_3$) δ 165.84 (C=O), 161.95 (C=O), 152.1 (ArC), 141.9 (ArC), 138.3 (ArC), 135.5 (ArC), 134.4 (ArC), 133.3 (ArC), 132.9 (ArCH), 131.3 (ArC), 130.4 (ArCH), 127.8 (ArCH), 127.2 (ArCH), 122.2 (ArCH), 115.6 (ArCH), 109.5 (ArC), 53.0 (OCH_3), 52.8 (OCH_3). ν_{max} (neat)/cm^{-1} 752, 772, 795, 1191, 1717, 3007, 3367; HRMS (ESI): Calcd. for $C_{18}H_{13}BrO_5SNa$ (M+Na$^+$), 444.9544; found 444.9539.

Dimethyl 4-(3-acetyl-4-hydroxyphenyl)benzo[b]thiophene-2,3-dicarboxylate, 4v

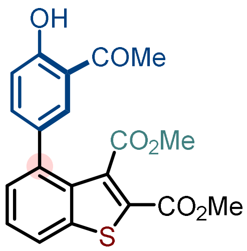

Prepared as described in General Procedure G, using dimethyl benzo[b]thiophene-2,3-dicarboxylate 1-oxide (53 mg, 0.20 mmol), and 2-acetylphenol (82 mg, 0.60 mmol). Purification by column chromatography on silica gel (5% EtOAc in hexane to 20% EtOAc in hexane), afforded the title compound as a white solid (31 mg, 0.08 mmol, 41%); ^{1}H NMR (400 MHz, $CDCl_3$) δ 12.36 (s, 1H, OH), 7.89 (d, *J* = 8.2 Hz, 1H, ArH), 7.70 (d, *J* = 2.2 Hz, 1H, ArH), 7.54 (t, *J* = 7.7 Hz, 1H, ArH), 7.50 (dd, *J* = 8.5, 2.2 Hz, 1H, ArH), 7.28 (d, *J* = 7.3 Hz, 1H, ArH), 7.05 (d, *J* = 8.5 Hz, 1H, ArH), 3.90 (s, 3H, OCH_3), 3.33 (s, 3H, OCH_3), 2.64 (s, 3H, CH_3).^{13}C NMR (101 MHz, $CDCl_3$) δ 205.0 (CH_3CO), 166.1 (COOMe), 162.1 (ArC), 161.9 (COOMe), 142.0 (ArC), 138.8 (ArC), 137.2 (ArCH), 135.6 (ArC), 134.5 (ArC), 132.2 (ArCH), 131.3 (ArC), 129.9 (ArC), 127.8 (ArCH), 127.3 (ArCH), 122.2 (ArCH), 118.7 (ArC), 118.2 (ArCH), 53.1 (OCH_3), 52.6 (OCH_3), 26.9 (CH_3). ν_{max} (neat)/cm^{-1} 771, 1103, 1368, 1695, 2780, 2853, 2921; HRMS (ESI): Calcd. for $C_{20}H_{16}O_6SNa$ (M+Na$^+$), 407.0565; found 407.0556.

1-(4-(3-Bromo-4-hydroxyphenyl)-2,5-dimethylbenzo[b]thiophen-3-yl)ethan-1-one, 4w

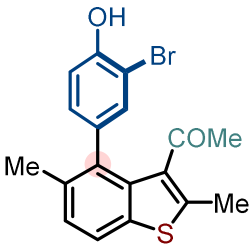

Prepared as described in General Procedure G, using 1 1-(2,5-dimethyl-1-oxidobenzo[b]thiophen-3-yl)ethan-1-one (44 mg, 0.19 mmol), and 2-bromophenol (173 mg, 0.99 mmol). Purification by column chromatography on silica gel (5% EtOAc in hexane to 20% EtOAc in hexane), afforded the title compound as a white solid (49 mg, 0.13 mmol, 65%); ^{1}H NMR (400 MHz, DMSO-d_6) δ 7.80 (d, *J* = 8.2 Hz, 1H, ArH), 7.27 (d, *J* = 8.3 Hz, 1H, ArH), 7.23 (d, *J* = 2.0 Hz, 1H, ArH), 7.00 (dd, *J* = 8.3, 2.0 Hz, 1H, ArH), 6.97 (d, *J* = 8.2 Hz, 1H, ArH), 2.35 (s, 3H, CH_3), 2.14 (s, 3H, CH_3), 1.69 (s, 3H, CH_3). ^{13}C NMR (101 MHz, DMSO-d6) δ 200.7 (C=O), 153.5 (ArC), 138.7 (ArC), 136.6 (ArC), 136.6 (ArC), 135.8 (ArC), 134.4 (ArC), 134.0 (ArC), 132.7 (ArC), 131.2 (ArC), 130.7 (ArCH), 126.9 (ArCH), 121.3 (ArCH), 115.9 (ArCH), 108.9 (ArCH), 31.4 (CH_3), 20.2 (CH_3), 14.1 (CH_3). ν_{max} (neat)/cm^{-1} 803, 1137, 1186, 1202, 1291, 1347, 1900, 2920, 3159; HRMS (APCI): Calcd. for $C_{18}H_{16}BrO_2S$ (M+H$^+$), 375.0054; found 375.0058.

XRD: Single crystal analysis confirms the structure.

1-(5-Bromo-4-(4-hydroxyphenyl)-2-methylbenzo[b]thiophen-3-yl)ethan-1-one, 4x

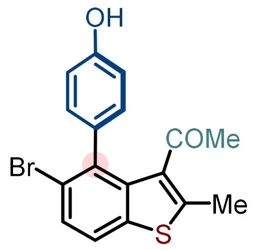

Prepared as described in General Procedure G, using 1-(5-bromo-2-methyl-1-oxidobenzo[b]thiophen-3-yl)ethan-1-one (57 mg, 0.20 mmol), and phenol (57 mg, 0.60 mmol). Purification by column chromatography on silica gel (5% EtOAc in hexane to 20% EtOAc in hexane), afforded the title compound as a white solid (46 mg, 0.13 mmol, 64%); ^{1}H NMR (400 MHz, Acetone-*d*$_6$) δ 7.80 (d, *J* = 8.6 Hz, 1H, ArH), 7.65 (d, *J* = 8.6 Hz, 1H, ArH), 7.10 (d, *J* = 8.4 Hz, 2H, ArH), 6.89 (d, *J* = 8.3 Hz, 2H, ArH), 5.62 (s, 1H, OH), 2.40 (s, 3H, CH_3), 1.64 (s, 3H, CH_3). ^{13}C NMR (101 MHz, Acetone-d$_6$) *δ* 200.8 (C=O), 158.5 (ArC), 141.2 (ArC), 139.2 (ArC), 138.9 (ArC), 138.4 (ArC), 137.8 (ArC), 133.1 (ArCH), 131.5 (ArC), 129.4 (ArCH), 123.4 (ArCH), 122.1 (ArC), 115.7 (ArCH), 31.6 (CH_3), 14.4 (CH_3). $ν_{max}$ (neat)/cm^{-1} 797, 852, 1014, 1191, 1263, 12881397, 1426, 1547, 1611, 1666, 2958, 3279; HRMS (ESI): Calcd. for $C_{17}H_{13}BrO_2S$ (M+H$^+$), 360.9898; found 360.9893.

1-(5-Bromo-4-(3-bromo-4-hydroxyphenyl)-2-methylbenzo[b]thiophen-3-yl)ethan-1-one, 4y

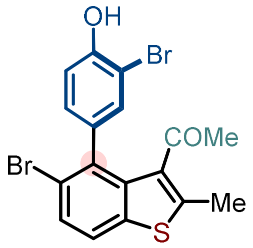

Prepared as described in General Procedure G, using 1-(5-bromo-2-methyl-1-oxidobenzo[b]thiophen-3-yl)ethan-1-one (57 mg, 0.20 mmol), and 2-bromophenol (104 mg, 0.60 mmol). Purification by column chromatography on silica gel (5% EtOAc in hexane to 20% EtOAc in hexane), afforded the title compound as a white solid (55 mg, 0.12 mmol, 62%); ^{1}H NMR (400 MHz, Acetone-*d*$_6$) δ 7.85 (d, *J* = 8.6 Hz, 1H, ArH), 7.67 (d, *J* = 8.6 Hz, 1H, ArH), 7.38 (d, *J* = 2.0 Hz, 1H, ArH), 7.10 (d, *J* = 2.1 Hz, 1H, ArH), 7.07 (d, *J* = 8.3 Hz, 1H, ArH), 2.43 (s, 3H, CH_3), 1.78 (s, 3H, CH_3). ^{13}C NMR (101 MHz, Acetone-d$_6$) δ 200.7 (C=O), 155.0 (ArC), 141.4 (ArC), 139.0 (ArC), 138.9 (ArC), 138.0 (ArC), 136.1 (ArC), 136.1 (ArCH), 133.0 (ArC), 132.2 (ArCH), 129.3 (ArCH), 123.9 (ArCH), 121.9 (ArC), 116.7 (ArCH), 109.8 (ArC), 31.9 (CH_3), 14.4 (CH_3). $ν_{max}$ (neat)/cm^{-1} 636, 794, 1040, 1170, 1286, 1393, 1681, 2852, 2920, 2956, 3054; HRMS (APCI): Calcd. for $C_{17}H_{13}Br_2O_2S$ (M+H$^+$), 438.9003; found 438.8998.

1-(7-Bromo-4-(3-bromo-4-hydroxyphenyl)-2-methylbenzo[b]thiophen-3-yl)ethan-1-one, 4z

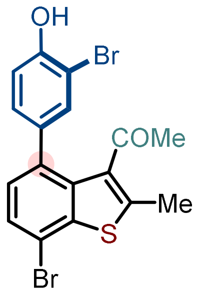

Prepared as described in General Procedure G, using 1-(7-bromo-2-methyl-1-oxidobenzo[b]thiophen-3-yl)ethan-1-one (57 mg, 0.20 mmol), and 2-bromophenol (104 mg, 0.60 mmol). Purification by column chromatography on silica gel (5% EtOAc in hexane to 20% EtOAc in hexane), afforded the title compound as a white solid (68 mg, 0.15 mmol, 77%); ^{1}H NMR (400 MHz, DMSO-d_6) δ 7.65 (d, J = 7.9 Hz, 1H, ArH), 7.40 (d, J = 2.2 Hz, 1H, ArH), 7.24 (d, J = 7.9 Hz, 1H, ArH), 7.14 (dd, J = 8.3, 2.2 Hz, 1H, ArH), 6.98 (d, J = 8.3 Hz, 1H, ArH), 2.47 (s, 3H, CH_3), 1.67 (s, 3H, CH_3). ^{13}C NMR (101 MHz, DMSO-d_6) δ 199.1 (C=O), 154.2 (ArC), 141.7 (ArC), 139.7 (ArC), 136.9 (ArC), 135.9 (ArC), 134.9 (ArC), 132.9 (ArCH), 132.3 (ArC), 129.2 (ArCH), 128.4 (ArCH), 127.2 (ArCH), 116.4 (ArCH), 113.6 (ArC), 109.5 (ArC), 31.0 (CH_3), 14.4 (CH_3). ν_{max} (neat)/cm^{-1} 811, 965, 1154, 1205, 1362, 1707, 1797, 1868, 2922, 3262; HRMS (ESI): Calcd. for $C_{17}H_{12}Br_2O_2SNa$ (M+Na$^+$), 462.8802; found 462.8796.

1-(4-(3-Bromo-4-hydroxyphenyl)-3-methylbenzo[b]thiophen-2-yl)ethan-1-one, 4aa Prepared as

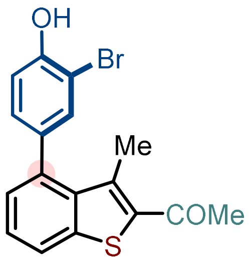

described in General Procedure G, using 1-(3-methyl-1-oxidobenzo[b]thiophen-2-yl)ethan-1-one (41 mg, 0.20 mmol), and 2-bromophenol (104 mg, 0.60 mmol). Purification by column chromatography on silica gel (5% EtOAc in hexane), afforded the title compound as a white solid (45 mg, 0.12 mmol, 62%) and corresponding minor C3 product (8.0 mg, 0.02 mmol, 11%); ^{1}H NMR (500 MHz, $CDCl_3$) δ 7.84 (dd, J = 8.1, 1.1 Hz, 1H, ArH), 7.47 – 7.43 (m, 2H, ArH), 7.21 – 7.18 (m, 2H, ArH), 7.08 (d, J = 8.3 Hz, 1H, ArH), 5.68 (br.s, 1H, OH), 2.63 (s, 3H, CH_3), 2.22 (s, 3H, CH_3). ^{13}C NMR (126 MHz, $CDCl_3$) δ 193.6 (C=O), 151.9 (ArC), 140.9 (ArC), 140.7 (ArC), 139.5 (ArC), 137.8 (ArC), 136.2 (ArC), 135.1 (ArC), 132.7 (ArCH), 130.3 (ArCH), 128.0 (ArCH), 126.6 (ArCH), 122.5 (ArCH), 115.6 (ArCH), 109.8 (ArC), 31.1 (CH_3), 17.9 (CH_3). ν_{max} (neat)/cm^{-1} 692, 728, 1031, 1099, 1239, 1259, 1492, 1649, 2920, 3216; HRMS (ESI): Calcd. for $C_{17}H_{13}BrO_2SNa$ (M+Na$^+$), 382.9717; found 382.9709.

Methyl 4-(4-hydroxyphenyl)benzo[*b*]thiophene-3-carboxylate, 4ab

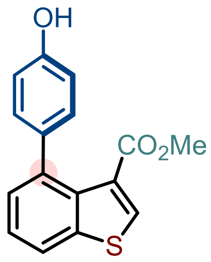

Prepared as described in General Procedure H, using benzo[b]thiophene-3-carboxylate 1-oxide (42 mg, 0.20 mmol), and phenol (28.2 mg, 0.30 mmol). Purification by column chromatography on silica gel (5% EtOAc in hexane to 20% EtOAc in hexane), afforded the title compound as a white solid (36 mg, 0.13 mmol, 64%) and corresponding minor C3 product (3.0 mg, 0.01 mmol, 5%); ^{1}H NMR (400 MHz, Acetone-d$_6$) δ 8.46 (s, 1H, OH), 8.12 (s, 1H, ArH), 7.98 (dd, *J* = 8.1, 1.1 Hz, 1H, ArH), 7.49 (t, *J* = 7.7 Hz, 1H, ArH), 7.34 (dd, *J* = 7.4, 1.1 Hz, 1H, ArH), 7.19 (d, *J* = 8.6 Hz, 2H, ArH), 6.92 (d, *J* = 8.5 Hz, 2H, ArH), 3.21 (s, 3H, OCH_3). ^{13}C NMR (101 MHz, Acetone-d$_6$) δ 165.8 (C=O), 157.6 (ArC), 142.1 (ArC), 139.7 (ArC), 134.8 (ArC), 134.5 (ArC), 132.7 (ArCH), 132.0 (ArC), 130.0 (ArCH), 127.8 (ArCH), 126.0 (ArCH), 122.1 (ArCH), 115.9 (ArCH), 51.8 (OCH_3). ν_{max} (neat)/cm^{-1} 758, 830, 862, 944, 1247, 1430, 1452, 1559, 1687, 2509, 3383; HRMS (ESI): Calcd. for $C_{16}H_{12}O_3SNa$ (M+Na$^+$), 307.0405; found 307.0409.

XRD: Single crystal analysis confirms the structure.

Methyl 4-(4-hydroxy-3-iodophenyl)benzo[b]thiophene-3-carboxylate, 4ac

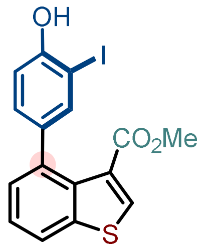

Prepared as described in General Procedure H, using benzo[b]thiophene-3-carboxylate 1-oxide (42 mg, 0.20 mmol), and 2-iodophenol (66 mg, 0.30 mmol). Purification by column chromatography on silica gel (5% EtOAc in hexane to 20% EtOAc in hexane), afforded the title compound as a white solid (57 mg, 0.14 mmol, 70%); ^{1}H NMR (400 MHz, Acetone-d$_6$) δ 8.17 (s, 1H, ArH), 8.01 (dd, *J* = 8.1, 1.1 Hz, 1H, ArH), 7.69 (d, *J* = 2.2 Hz, 1H, ArH), 7.53 – 7.47 (m, 1H, ArH), 7.36 (dd, *J* = 7.3, 1.1 Hz, 1H, ArH), 7.22 (dd, *J* = 8.3, 2.2 Hz, 1H, ArH), 7.03 (d, *J* = 8.3 Hz, 1H, ArH), 3.32 (s, 3H, OCH_3). ^{13}C NMR (101 MHz, Acetone-d$_6$) δ 165.5 (C=O), 156.7 (ArC), 142.1 (ArC), 139.4 (ArCH), 137.8 (ArC), 136.5 (ArC), 134.6 (ArC), 133.4 (ArCH), 131.5 (ArC), 130.1 (ArCH), 127.9 (ArCH), 126.0 (ArCH), 122.6 (ArCH), 115.5 (ArCH), 84.0 (ArC), 52.1 (OCH_3). ν_{max} (neat)/cm^{-1} 787, 1025, 1186, 1562, 1687, 2945, 3096, 3274; HRMS (ESI): Calcd. for $C_{16}H_1O_3ISNa$(M+ Na$^+$), 432.9371; found 432.9381.

Methyl 4-(3-bromo-4-hydroxyphenyl)benzo[b]thiophene-3-carboxylate, 4ad

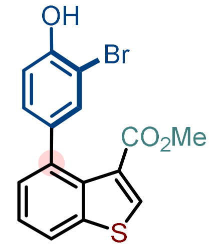

Prepared as described in General Procedure H, using benzo[b]thiophene-3-carboxylate 1-oxide (42 mg, 0.20 mmol), and 2-bromophenol (52 mg, 0.30 mmol). Purification by column chromatography on silica gel [(5% EtOAc in hexane to 20% EtOAc in hexane), afforded the title compound as a white solid (55 mg, 0.15 mmol, 76%); ^{1}H NMR (400 MHz, Acetone-d_6) δ 8.17 (s, 1H, ArH), 8.02 (dd, *J* = 8.1, 1.2 Hz, 1H, ArH), 7.51 (t, *J* = 7.7 Hz, 1H, ArH), 7.48 (d, *J* = 2.2 Hz, 1H, ArH), 7.37 (dd, *J* = 7.3, 1.1 Hz, 1H, ArH), 7.19 (dd, *J* = 8.3, 2.2 Hz, 1H, ArH), 7.09 (d, *J* = 8.3 Hz, 1H, ArH), 3.32 (s, 3H, OCH_3). ^{13}C NMR (101 MHz, Acetone-d_6) δ 165.5 (C=O), 154.1 (ArC), 142.1 (ArC), 137.9 (ArC), 136.1 (ArC), 134.6 (ArC), 133.4 (ArCH), 133.2 (ArCH), 131.4 (ArC), 129.3 (ArCH), 127.9 (ArCH), 126.0 (ArCH), 122.7 (ArCH), 116.9 (ArCH), 110.1 (ArC), 52.0 (OCH_3). ν_{max} (neat)/cm^{-1} 685, 811, 1290, 1347, 1498, 1693, 3097, 3341; HRMS (ESI): Calcd. for $C_{16}H_{11}O_3BrSNa$ (M+Na$^+$), 384.9510; found 384.9518.

Methyl 4-(3,5-dichloro-4-hydroxyphenyl)benzo[b]thiophene-3-carboxylate, 4ae

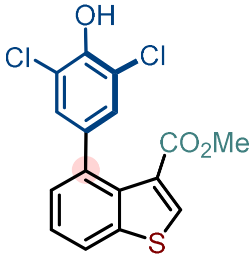

Prepared as described in General Procedure H, using benzo[b]thiophene-3-carboxylate 1-oxide (42 mg, 0.20 mmol), and 2,6-dichlorophenol (49 mg, 0.30 mmol). Purification by column chromatography on silica gel (5% EtOAc in hexane to 20% EtOAc in hexane), afforded the title compound as a white solid (35 mg, 0.09 mmol, 50%); ^{1}H NMR (400 MHz, $CDCl_3$) δ 8.02 (s, 1H, ArH), 7.89 (dd, *J* = 8.1, 1.1 Hz, 1H, ArH), 7.44 (t, *J* = 7.7 Hz, 1H, ArH), 7.34 – 7.31 (m, 3H, ArH), 5.92 (br.s, 1H, OH), 3.47 (s, 3H, OCH_3). ^{13}C NMR (101 MHz, $CDCl_3$) δ 165.0 (C=O), 147.0 (ArC), 141.6 (ArC), 135.9 (ArC), 135.7 (ArC), 133.6 (ArC), 133.5 (ArC), 129.9 (ArC), 127.9 (ArCH), 127.4 (ArCH), 125.2 (ArCH), 122.5 (ArCH), 121.0 (ArCH), 52.2 (OCH_3). ν_{max} (neat)/cm^{-1} 778, 796, 876, 947, 1219, 1295, 1487, 1919, 3407; HRMS (ESI): Calcd. for $C_{16}H_{10}O_3Cl_2SNa$ (M+Na$^+$), 374.9625; found 374.9634.

Methyl 4-(3-formyl-4-hydroxyphenyl)benzo[b]thiophene-3-carboxylate, 4af

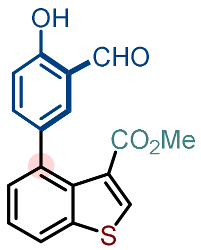

Prepared as described in General Procedure H, using benzo[b]thiophene-3-carboxylate 1-oxide (42 mg, 0.20 mmol), and salicyladehyde (37 mg, 0.30 mmol). Purification by column chromatography on silica gel (5% EtOAc in hexane to 20% EtOAc in hexane), afforded the title compound as a white solid (34 mg, 0.11 mmol, 54%) and corresponding minor C3 product (7.5 mg, 0.02 mmol, 12%); ^{1}H NMR (400 MHz, $CDCl_3$) δ 11.05 (s, 1H, OH), 9.92 (s, 1H, CHO), 8.02 (s, 1H, ArH), 7.90 (d, *J* = 8.2 Hz, 1H, ArH), 7.58 – 7.55 (m, 2H, ArH), 7.47 (t, *J* = 7.7 Hz, 1H, ArH), 7.35 (d, *J* = 7.2 Hz, 1H, ArH), 7.07 (d, *J* = 8.3 Hz, 1H, ArH), 3.32 (s, 3H, OCH_3). ^{13}C NMR (101 MHz, $CDCl_3$) δ 196.8 (CHO), 165.7 (C=O), 160.9 (ArC), 141.6 (ArC), 136.9 (ArCH), 136.7 (ArC), 134.6 (ArC), 133.8 (ArC), 133.5 (ArCH), 132.8 (ArCH), 130.0 (ArC), 127.4 (ArCH), 125.3 (ArCH), 122.2 (ArCH), 120.4 (ArC), 117.7 (ArCH), 52.1 (OCH_3). ν_{max} (neat)/cm^{-1} 761, 1074, 1158, 1584, 1653, 1712, 2850, 2920, 2950, 3093; HRMS (ESI): Calcd. for $C_{17}H_{12}O_4SNa$ (M+Na$^+$), 335.0354; found 335.0359.

Methyl 4-(4-hydroxy-3-nitrophenyl)benzo[b]thiophene-3-carboxylate, 4ag

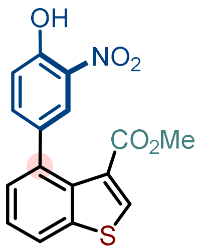

Prepared as described in General Procedure H, using benzo[b]thiophene-3-carboxylate 1-oxide (42 mg, 0.20 mmol), and 2-nitrophenol (42 mg, 0.30 mmol). Purification by column chromatography on silica gel (5% EtOAc in hexane), afforded the title compound as a yellow solid (36 mg, 0.11 mmol, 55%) and corresponding minor C3 product (10 mg, 0.03 mmol, 15%); ^{1}H NMR (400 MHz, Acetone-d$_6$) δ 8.29 (s, 1H, ArH), 8.08 (dd, *J* = 8.1, 1.1 Hz, 1H, ArH), 8.05 (d, *J* = 2.3 Hz, 1H, ArH), 7.66 (dd, *J* = 8.6, 2.3 Hz, 1H, ArH), 7.55 (t, *J* = 7.7 Hz, 1H, ArH), 7.44 (dd, *J* = 7.4, 1.1 Hz, 1H, ArH), 7.29 (d, *J* = 8.6 Hz, 1H, ArH), 3.37 (s, 3H, OCH_3). ^{13}C NMR (101 MHz, Acetone-d$_6$) δ 165.0 (C=O), 154.3 (ArC), 142.4 (ArC), 137.9 (ArCH), 136.6 (ArC), 135.4 (ArC), 134.9 (ArCH), 134.7 (ArC), 134.37 (ArC), 130.6 (ArC), 128.3 (ArCH), 126.1 (ArCH), 124.6 (ArCH), 123.4 (ArCH), 120.5 (ArCH), 52.1 (OCH_3). ν_{max} (neat)/cm^{-1} 760, 1218, 1315, 1454, 1531, 1719, 2854, 2951, 3270; HRMS (ESI): Calcd. for $C_{16}H_{11}O_5NSNa$ (M+Na$^+$), 352.0256; found 352.0261.

Methyl 5-bromo-4-(3-bromo-4-hydroxyphenyl)benzo[b]thiophene-3-carboxylate, 4ah

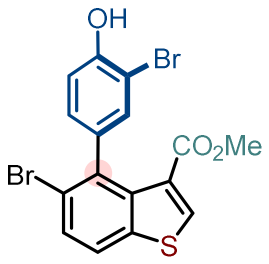

Prepared as described in General Procedure H, using methyl 5-bromobenzo[b]thiophene-3-carboxylate 1-oxide (57 mg, 0.20 mmol), and 2-bromophenol (52 mg, 0.30 mmol). Purification by column chromatography on silica gel (5% EtOAc in hexane to 20% EtOAc in hexane), afforded the title compound as a white solid (53 mg, 0.12 mmol, 60%); ^{1}H NMR (400 MHz, $CDCl_3$) δ 7.83 (s, 1H, ArH), 7.72 (d, *J* = 8.6 Hz, 1H, ArH), 7.68 (d, *J* = 8.7 Hz, 1H, ArH), 7.42 (d, *J* = 2.1 Hz, 1H, ArH), 7.20 (dd, *J* = 8.3, 2.1 Hz, 1H, ArH), 7.11 (d, *J* = 8.4 Hz, 1H, ArH), 5.64 (br.s, 1H, OH), 3.33 (s, 3H, OCH_3). ^{13}C NMR (101 MHz, $CDCl_3$) δ 165.2 (C=O), 152.0 (ArC), 139.9 (ArC), 136.4 (ArC), 136.1 (ArC), 133.8 (ArC), 133.2 (ArCH), 132.7 (ArCH), 131.0 (ArCH), 130.9 (ArC), 129.7 (ArCH), 123.4 (ArCH), 122.1 (ArC), 115.7 (ArCH), 109.7 (ArC), 52.4 (OCH_3). ν_{max} (neat)/cm^{-1} 735, 752, 773, 1043, 1326, 1438, 1501, 1543, 1571, 1730, 2850, 2920, 3385; HRMS (ESI): Calcd. for $C_{16}H_{10}Br_2O_3S(M+H^+)$, 440.8796; found 440.8791.

Methyl 4-(4-hydroxyphenyl)-5-phenylbenzo[b]thiophene-3-carboxylate, 4ai

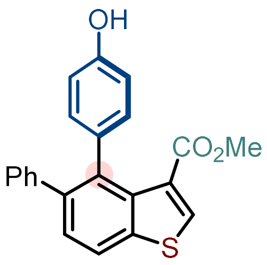

Prepared as described in General Procedure H, using methyl 5-phenylbenzo[b]thiophene-3-carboxylate 1-oxide (57 mg, 0.20 mmol), and phenol (28 mg, 0.30 mmol). Purification by column chromatography on silica gel (5% EtOAc in hexane to 20% EtOAc in hexane), afforded the title compound as a white solid (43 mg, 0.12 mmol, 60%) and corresponding minor C3 product (5.0 mg, 0.01 mmol, 7%); ^{1}H NMR (400 MHz, Acetone-d_6) δ 8.05 (d, *J* = 8.4 Hz, 1H, ArH), 8.02 (d, *J* = 0.5 Hz, 1H, ArH), 7.44 (dt, *J* = 8.1, 0.7 Hz, 2H, ArH), 7.20 – 7.17 (m, 2H, ArH), 7.09 – 7.07 (m, 2H, ArH), 6.87 – 6.85 (m, 2H, ArH), 6.70 – 6.67 (m, 2H, ArH), 3.18 (s, 3H, OCH_3). ^{13}C NMR (101 MHz, Acetone-d_6) δ 166.1 (C=O), 157.1 (ArC), 142.7 (ArC), 140.7 (ArC), 139.7 (ArC), 137.1 (ArC), 136.3 (ArC), 132.9 (ArC), 132.3 (ArCH), 131.8 (ArCH), 131.6 (ArC), 130.9 (ArCH), 128.7 (ArCH), 128.4 (ArCH), 127.0 (ArCH), 122.4 (ArCH), 115.3 (ArCH), 52.0 (OCH_3). ν_{max} (neat)/cm^{-1} 705, 750, 1208, 1224, 1443, 1470, 3080, 3450; HRMS (APCI): Calcd. for $C_{22}H_{16}O_3S(M+H^+)$, 361.0898; found 361.0893.

Methyl 4-(3-bromo-4-hydroxyphenyl)-5-phenylbenzo[b]thiophene-3-carboxylate, 4aj

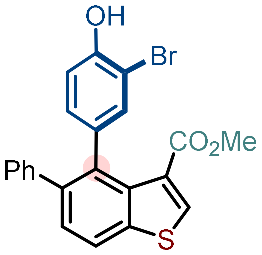

Prepared as described in General Procedure H, using methyl 5-phenylbenzo[b]thiophene-3-carboxylate 1-oxide (57 mg, 0.20 mmol), and 2-bromophenol (52 mg, 0.30 mmol). Purification by column chromatography on silica gel (5% EtOAc in hexane to 20% EtOAc in hexane), afforded the title compound as a white solid (62 mg, 0.14 mmol, 70%); ^{1}H NMR (400 MHz, $CDCl_3$) δ 7.91 (d, *J* = 8.3 Hz, 1H, ArH), 7.88 (s, 1H, ArH), 7.44 (d, *J* = 8.3 Hz, 2H, ArH), 7.23 – 7.21 (m, 3H, ArH), 7.06 – 7.03 (m, 2H, ArH), 6.91 (dd, *J* = 8.4, 2.0 Hz, 1H, ArH), 6.85 (d, *J* = 8.3 Hz, 1H, ArH), 3.30 (s, 3H, OCH_3). ^{13}C NMR (101 MHz, $CDCl_3$) δ 165.7 (C=O), 151.2 (ArC), 141.1 (ArC), 140.2 (ArC), 139.1 (ArC), 135.1 (ArC), 134.2 (ArC), 133.9 (ArC), 133.5 (ArCH), 132.2 (ArCH), 131.4 (ArCH), 131.2 (ArC), 130.2 (ArCH), 128.0 (ArCH), 127.9 (ArCH), 126.6 (ArCH), 122.1 (ArCH), 115.5 (ArCH), 109.5 (ArC), 52.3 (OCH_3). ν_{max} (neat)/cm^{-1} 679, 712, 1369, 1439, 1479, 1640, 3450; HRMS (APCI): Calcd. for $C_{22}H_{16}BrO_3S$(M+H$^+$), 440.9983; found 440.9979.

4-(4,6-Dimethyldibenzo[*b,d*]thiophen-1-yl)phenol, 4ak

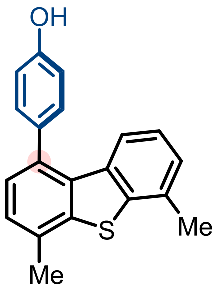

Prepared as described in General Procedure I, using 4,6-dimethyldibenzo[*b*,*d*]thiophene 5-oxide (46 mg, 0.20 mmol), and phenol (28 mg, 0.30 mmol). Purification by column chromatography on silica gel (5% EtOAc in hexane to 50% EtOAc in hexane), afforded the title compound as white solid (38 mg, 0.12 mmol, 62%); ^{1}H NMR (400 MHz, $CDCl_3$) δ 7.33 (d, *J* = 8.5 Hz, 2H, ArH), 7.28 (dd, *J* = 7.4, 0.9 Hz, 1H, ArH), 7.20 – 7.17 (m, 2H, ArH), 7.12 – 7.04 (m, 2H, ArH), 6.97 (d, *J* = 8.5 Hz, 2H, ArH), 4.95 (br.s, 1H, OH), 2.66 (s, 3H, CH_3), 2.61 (s, 3H, CH_3); ^{13}C NMR (101 MHz, $CDCl_3$) δ 155.2 (ArC), 139.9 (ArC), 139.6 (ArC), 136.9 (ArC), 136.3 (ArC), 134.1 (ArC), 133.5 (ArC), 131.9 (ArC), 131.1 (ArC), 130.7 (ArCH), 127.5 (ArCH), 126.4 (ArCH), 126.1 (ArCH), 124.3 (ArCH), 122.6 (ArCH), 115.6 (ArCH), 20.7 (CH_3), 20.6 (CH_3). ν_{max} (neat)/cm^{-1} 797, 1029, 1098, 1260, 1481, 1612, 1973, 2152, 2966, 3304; HRMS (ESI): Calcd. for $C_{20}H_{17}OS$ (M+H$^+$), 305.1000; found 305.0993.

2-Bromo-4-(4,6-dimethyldibenzo[*b,d*]thiophen-1-yl)phenol, 4al

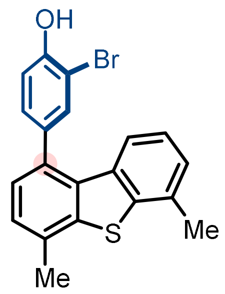

Prepared as described in General Procedure I, using 4,6-dimethyldibenzo[*b*,*d*]thiophene 5-oxide (46 mg, 0.20 mmol), 2-bromophenol (52 mg, 0.30 mmol). Purification by column chromatography on silica gel (5% EtOAc in hexane to 50% EtOAc in hexane), afforded the title compound as a white solid (28 mg, 0.07 mmol, 36%); ^{1}H NMR (400 MHz, $CDCl_3$) δ 7.58 (d, *J* = 2.0 Hz, 1H, ArH), 7.32 (dd, *J* = 8.3, 2.0 Hz, 1H, ArH), 7.28 (dd, *J* = 7.4, 1.0 Hz, 1H, ArH), 7.23 – 7.08 (m, 5H, ArH), 5.69 (br.s, 1H, OH), 2.66 (s, 3H, CH_3), 2.61 (s, 3H, CH_3). ^{13}C NMR (101 MHz, $CDCl_3$) δ 151.9 (ArC), 140.1 (ArC), 139.6 (ArC), 136.0 (ArC), 135.4 (ArC), 133.3 (ArC), 132.7 (ArCH), 132.1 (ArC), 131.6 (ArC), 130.5 (ArCH), 127.5 (ArCH), 126.6 (ArCH), 126.1 (ArCH), 124.4 (ArCH), 122.4 (ArCH), 116.2 (ArCH), 110.4 (ArC), 20.7 (CH_3), 20.6 (CH_3). ν_{max} (neat)/cm^{-1} 690, 705, 716, 731, 753, 798, 860, 1036, 1053, 1064, 1181, 1225, 1239, 1260, 1312, 1328, 1349, 1378, 1398, 1441, 1477, 1504, 1572, 1602, 2909, 2963, 3504; HRMS (ESI): Calcd. for $C_{20}H_{14}OBrS$ (M-H$^+$), 380.9949; found 380.9926.

XRD: Single crystal analysis confirms the structure.

2-Allyl-4-(4,6-dimethyldibenzo[b,d]thiophen-1-yl)phenol, 4am

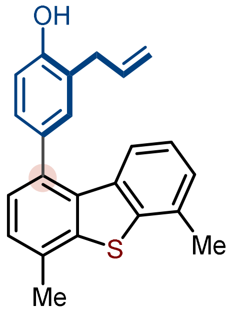

Prepared as described in General Procedure I, using 4,6-dimethyldibenzo[*b*,*d*]thiophene 5-oxide (46 mg, 0.20 mmol), and 2-allylphenol (40 mg, 0.30 mmol). Purification by column chromatography on silica gel (5% EtOAc in hexane to 50% EtOAc in hexane), afforded the title compound as a white solid (32 mg, 0.09 mmol, 46%); ^{1}H NMR (400 MHz, $CDCl_3$) δ 7.33 – 7.08 (m, 7H, ArH), 7.00 (d, *J* = 8.0 Hz, 1H, ArH), 6.11 (ddt, *J* = 16.5, 9.9, 6.3 Hz, 1H, CH_2-C*H*=CH_2), 5.30 – 5.16 (m, 3H, CH_2-CH=C*H₂*, OH peak is merged), 3.53 (d, *J* = 5.7 Hz, 2H, C*H₂*-CH=CH_2), 2.71 (s, 3H, CH_3), 2.65 (s, 3H, CH_3). ^{13}C NMR (101 MHz, $CDCl_3$) δ 153.8 (ArC), 139.9 (ArC), 139.6 (ArC), 137.1 (ArC), 136.4 (=CH), 136.3 (ArC), 134.2 (ArC), 133.4 (ArC), 131.9 (ArC), 131.6 (ArCH), 131.0 (ArC), 128.9 (ArCH), 127.4 (ArCH), 126.4 (ArCH), 126.1 (ArCH), 125.6 (ArC), 124.2 (ArCH), 122.7 (ArCH), 116.8 (=CH_2), 116.1 (ArCH), 35.2 (CH_2), 20.7 (CH_3), 20.6 (CH_3). ν_{max} (neat)/cm^{-1} 754, 798, 868, 915, 1025, 1054, 1064, 1093, 1157, 1195, 1261, 1350, 1379, 1399, 1418, 1436, 1481, 1506, 1608, 1638, 2908, 2963, 3526. HRMS (ESI): Calcd. for $C_{23}H_{20}OS$

(M+H$^+$), 345.1313; found 345.1308.

2,6-Dichloro-4-(4,6-dimethyldibenzo[b,d]thiophen-1-yl)phenol, 4an

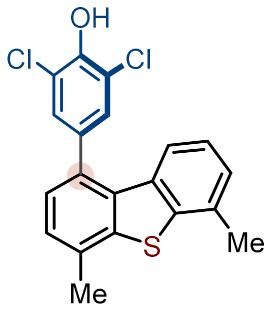

Prepared as described in General Procedure I, using 4,6-dimethyldibenzo[*b*,*d*]thiophene 5-oxide (46 mg, 0.20 mmol), and 2,6-dichlorophenol (49 mg, 0.30 mmol). Purification by column chromatography on silica gel (5% EtOAc in hexane to 50% EtOAc in hexane), afforded the title compound as a white solid (31 mg, 0.08 mmol, 42%); ^{1}H NMR (400 MHz, $CDCl_3$) δ 7.38 (s, 2H, 2 x ArH), 7.27 (d, *J* = 7.3 Hz, 1H, ArH), 7.22 (t, *J* = 7.3 Hz, 1H, ArH), 7.18 – 7.10 (m, 3H, ArH), 5.99 (s, 1H, OH), 2.66 (s, 3H, CH_3), 2.61 (s, 3H, CH_3). ^{13}C NMR (101 MHz, $CDCl_3$) δ 147.4 (ArC), 140.2 (ArC), 139.7 (ArC), 135.7 (ArC), 135.0 (ArC), 134.2 (ArC), 133.0 (ArC), 132.2 (ArC), 132.1 (ArC), 129.3 (ArCH), 127.4 (ArCH), 126.7 (ArCH), 126.2 (ArCH), 124.6 (ArCH), 122.3 (ArCH), 121.3 (ArC), 20.8 (CH_3), 20.6 (CH_3). ν_{max} (neat)/cm^{-1} 626, 668, 799, 1048, 1089, 1164, 1195, 1260, 1294, 1476, 1491, 1501, 1921; HRMS (ESI): Calcd. for $C_{20}H_{14}Cl_2OS$(M+H$^+$),373.0221, Found 373.0215.

XRD: Single crystal analysis confirms the structure.

4-(4-Methyldibenzo[*b,d*]thiophen-1-yl)phenol, 4ao

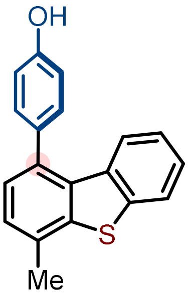

Prepared as described in General Procedure I, using 4-methyldibenzo[b,d]thiophene 5-oxide (42 mg, 0.20 mmol), and phenol (28 mg, 0.30 mmol). Purification by column chromatography on silica gel (5% EtOAc in hexane to 50% EtOAc in hexane), afforded the title compound as white solid (23 mg, 0.08 mmol, 40%); ^{1}H NMR (400 MHz, $CDCl_3$) δ 7.82 (dt, *J* = 8.0, 0.9 Hz, 1H, ArH), 7.34 – 7.20 (m, 5H, ArH), 7.16 (d, *J* = 7.4 Hz, 1H, ArH), 7.11 – 7.05 (m, 1H, ArH), 6.95 (d, *J* = 8.6 Hz, 2H, ArH), 5.00 (br.s, 1H, OH), 2.60 (s, 3H, CH_3). ^{13}C NMR (101 MHz, $CDCl_3$) δ 155.2 (ArC), 140.1 (ArC), 139.5 (ArC), 136.9 (ArC), 136.5 (ArC), 134.1 (ArC), 132.8 (ArC), 131.1 (ArC), 130.7 (ArCH), 127.4 (ArCH), 126.3 (ArCH), 126.1 (ArCH), 125.0 (ArCH), 123.9 (ArCH), 122.8 (ArCH), 115.6 (ArCH), 20.6 (CH_3). ν_{max} (neat)/cm^{-1} 779, 892, 938, 1240, 1480, 1775, 2920, 3233, 3595; HRMS (ESI): Calcd. for $C_{19}H_{13}OS$ (M-H$^+$), 289.0687; found 289.0691.

4-(2-Phenyldibenzo[b,d]thiophen-1-yl)phenol, 4ap

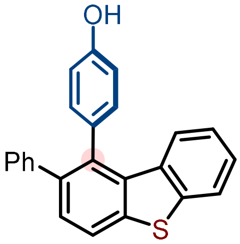

Prepared as described in General Procedure I, using 2-phenyldibenzo[b,d]thiophene 5-oxide (55 mg, 0.20 mmol), and phenol (28 mg, 0.30 mmol). Purification by column chromatography on silica gel (5% EtOAc in hexane to 50% EtOAc in hexane), afforded the title compound as white solid (30 mg, 0.08 mmol, 43%); ^{1}H NMR (400 MHz, $CDCl_3$) δ 7.90 (d, *J* = 8.2 Hz, 1H, ArH), 7.82 (d, *J* = 8.1 Hz, 1H, ArH), 7.48 (d, *J* = 8.2 Hz, 1H, ArH), 7.33 (ddd, *J* = 8.1, 7.1, 1.2 Hz, 1H, ArH), 7.20 – 7.03 (m, 8H, ArH), 6.85 – 6.78 (m, 3H, ArH), 4.80 (s, 1H, OH). ^{13}C NMR (126 MHz, Acetone-d_6) δ 156.8 (ArC), 141.6 (ArC), 139.8 (ArC), 139.1 (ArC), 138.6 (ArC), 137.7 (ArC), 136.0 (ArC), 133.8 (ArC), 131.3 (ArCH), 130.3 (ArC), 130.1 (ArCH), 128.5 (ArCH), 127.4 (ArCH), 126.2 (ArCH), 126.1 (ArCH), 125.1 (ArCH), 123.7 (ArCH), 122.6 (ArCH), 121.7 (ArCH), 115.4 (ArCH). ν_{max} (neat)/cm^{-1} 730, 746, 1068, 1098, 1132, 1287, 1330, 1363, 1512, 1546, 1671, 1889, 2105, 3397, 3528; HRMS (ESI): Calcd. for $C_{24}H_{15}OS$ (M-H), 351.0844; found 351.0859.

4-(4-Phenyldibenzo[b,d]thiophen-1-yl)phenol, 4aq

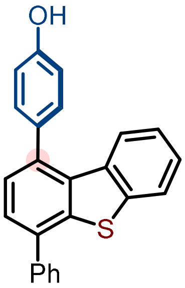

Prepared as described in General Procedure I, using 4-phenyldibenzo[b,d]thiophene 5-oxide (55 mg, 0.20 mmol), and phenol (28 mg, 0.30 mmol). Purification by column chromatography on silica gel (5% EtOAc in hexane to 50% EtOAc in hexane), afforded the title compound as white solid (32 mg, 0.09 mmol, 45%); ^{1}H NMR (400 MHz, $CDCl_3$) δ 7.80 – 7.74 (m, 3H, ArH), 7.54 (t, *J* = 7.6 Hz, 2H, ArH), 7.48 – 7.43 (m, 2H, ArH), 7.41 – 7.31 (m, 4H, ArH), 7.27 (d, *J* = 6.8 Hz, 1H, overlapped with $CDCl_3$, ArH), 7.14 – 7.08 (m, 1H, ArH), 7.04 – 6.99 (m, 2H, ArH), 5.02 (s, 1H, OH). ^{13}C NMR (126 MHz, Acetone-d_6) δ 158.3 (ArC) 141.4 (ArC), 140.4 (ArC), 139.7 (ArC), 136.8 (ArC), 136.6 (ArC), 134.3 (ArC), 133.0 (ArC), 131.0 (ArCH), 129.8 (ArCH), 129.2 (ArCH), 129.0 (ArCH), 128.8 (ArCH), 127.3 (ArCH), 127.0 (ArCH), 125.7 (ArCH), 124.7 (ArCH), 123.4 (ArCH), 116.5 (ArCH) ppm. ν_{max} (neat)/cm^{-1} 703, 735, 780, 819, 1137, 1227, 1315, 1471, 1512, 1814, 3032, 3302; HRMS (ESI): Calcd. for $C_{24}H_{15}OS$ (M-H), 351.0844; found 351.0849.

5. Control Experiments

5.1 The importance of the free OH in the phenol partner:

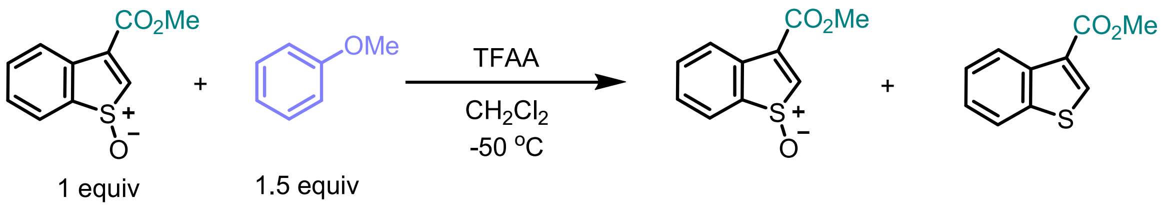

no C4 coupling; starting material recovered

Sulfoxide 2ab (41.6 mg, 0.20 mmol, 1.0 equiv) and anisole (32.4 mg, 0.30 mmol) were dissolved in CH_2Cl_2 (1 mL) in an oven-dried tube. TFAA (63 μL, 0.3 mmol, 1.5 equiv) was then added at −40 °C. After 15 min at −40 °C, the mixture was warmed to room temperature and stirred for 2 h. Saturated aqueous $NaHCO_3$ was then added and the aqueous phase was extracted with CH_2Cl_2 (3 × 3 mL). The combined organic layers were dried over $MgSO_4$ and concentrated *in vacuo*. The NMR analysis of the reaction mixture shows 60% (by NMR) of recovered sulfoxide and sulfide 6% (by NMR).

5.2 The importance of an electron-withdrawing group at C3:

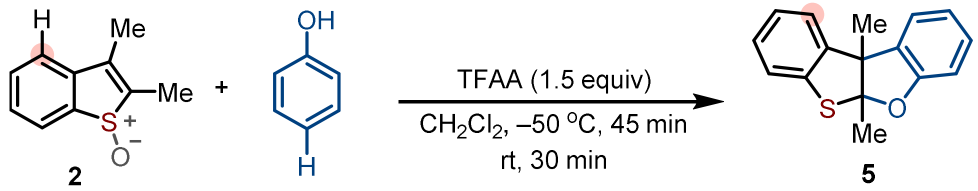

-no C4 coupling - only C3 coupling-

Prepared as described in General Procedure G, 2,3-dimethylbenzo[b]thiophene 1-oxide (35.6 mg, 0.20 mmol, 1.0 equiv), phenol (57 mg, 0.60 mmol, 3.0 equiv). Purification by column chromatography on silica gel (1% EtOAc in hexane), afforded the title compound as a white solid (33 mg, 0.13 mmol, 65%). ^{1}H NMR (400 MHz, $CDCl_3$) δ 7.28 (dt, *J* = 6.7, 1.4 Hz, 1H, ArH), 7.22 (dd, *J* = 7.4, 1.4 Hz, 1H, ArH), 7.18 – 7.09 (m, 4H, ArH), 6.95 (td, *J* = 7.5, 1.0 Hz, 1H, ArH), 6.85 (d, *J* = 8.0 Hz, 1H, ArH), 1.91 (s, 3H, CH_3), 1.60 (s, 3H, CH_3). ^{13}C NMR (101 MHz, $CDCl_3$) δ 157.2 (ArC), 143.7 (ArC), 138.5 (ArC), 132.9 (ArC), 128.7 (ArCH), 128.2 (ArCH), 125.2 (ArCH), 124.2 (ArCH), 123.3 (ArCH), 121.9 (ArCH), 111.7 (ArC), 110.5 (ArCH), 62.4 (ArC), 23.4 (CH_3), 21.6 (CH_3). ν_{max} (neat)/cm^{-1} 750, 880, 1034, 1068, 1110, 1185, 1455, 1594, 2926, 3340; HRMS (ESI): Calcd. for

$C_{16}H_{14}OSNa$ (M+Na$^+$), 277.0663; found 277.0655.

XRD: Single crystal analysis confirms the structure.

6. Selective manipulations of the products of C4-arylation

6.1. Benzofuran synthesis

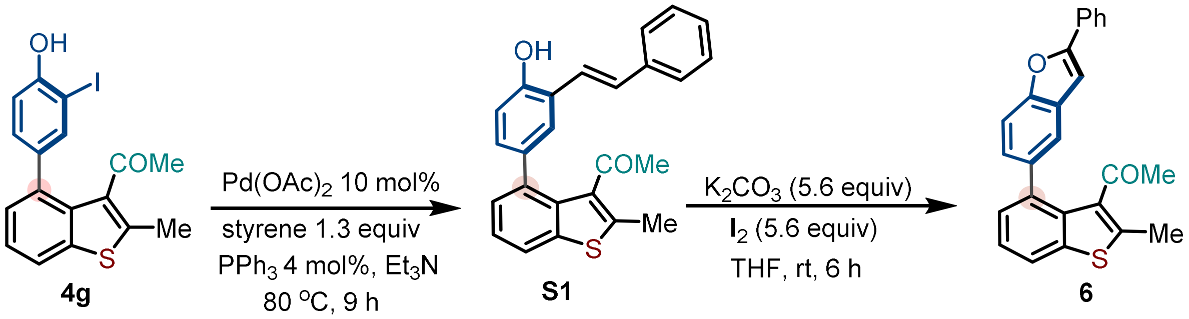

In a vial, **4g** (408 mg, 1.0 mmol, 1.0 equiv), styrene (149 µL, 1.3 mmol, 1.3 equiv), Et_3N (0.5 mL), $Pd(OAc)_2$ (22.5 mg, 0.1 mmol, 10 mol%) with PPh_3 (10.5 mg, 0.04 mmol, 4 mol%) were heated at 80 °C for 9 hours. The reaction mixture was cooled to room temperature. To the reaction mixture ice-cold H_2O (10 mL) was added. The reaction mixture was acidified using aqueous HCl (1 M) followed by extraction with EtOAc (3 × 15 mL). The combined extracts were dried over $MgSO_4$ and concentrated *in vacuo*. The residue was purified by column chromatography on silica gel (5% EtOAc in hexane) as eluent to give the Heck product **S1** as an off-white solid (231 mg, 0.60 mmol, 60%). ^{1}H NMR (400 MHz, $CDCl_3$) δ 7.78 – 7.73 (m, 1H, ArH), 7.63 (d, *J* = 2.2 Hz, 1H, ArH), 7.57 – 7.53 (m, 2H, ArH), 7.47 (d, *J* = 16.5 Hz, 1H, CH=), 7.41 – 7.32 (m, 4H, OH peak merged, ArH), 7.25 (d, *J* = 9.4 Hz, 1H, ArH), 7.21 – 7.13 (m, 2H out of which 1CH= another ArH), 7.02 (t, *J* = 4.1 Hz, 1H, ArH), 6.91 (d, *J* = 8.2 Hz, 1H, ArH), 2.56 (s, 3H, CH_3), 1.74 (s, 3H, CH_3). ^{13}C NMR (101 MHz, $CDCl_3$) δ 203.0 (C=O), 153.9 (ArC), 142.6 (ArC), 139.4 (ArC), 137.8 (ArC), 137.3 (ArC), 136.0 (ArC), 134.4 (ArC), 130.3 (ArCH), 129.4 (CH=), 128.7 (ArCH), 127.7 (ArCH), 127.3 (ArCH), 126.9 (ArCH), 126.8 (ArCH), 125.3 (ArC), 124.7 (ArCH), 123.0 (ArCH), 120.9 (ArCH), 116.3 (CH=), 31.5 (CH_3), 15.0 (CH_3). ν_{max} (neat)/cm^{-1} 700, 770, 1050, 1177, 1352, 1434, 1684, 2852, 2922; HRMS (ESI): Calcd. for $C_{25}H_{19}O_2S$ (M-H), 383.1106; found 383.1120.

To a solution of the Heck product (193 mg, 0.5 mmol, 1.0 equiv) in THF (5 mL) was added anhydrous K_2CO_3 (383 mg, 2.8 mmol, 5.6 equiv) and the solution stirred for 10 min. I_2 (705 mg, 2.8 mmol, 5.6 equiv) was added, and the mixture was stirred at ambient temperature until the

starting material was consumed. The mixture was poured into saturated aqueous $NaHCO_3$ (8 mL) and treated with saturated aqueous $NaHSO_3$ (8 mL) to remove the unreacted iodine. The mixture was extracted with EtOAc (3 × 10 mL), and the organic layer was dried over anhydrous $MgSO_4$. The organic layer was concentrated, and the crude material was purified by column chromatography (10% EtOAc in hexane) to give the desired benzofuran product (96 mg, 0.25 mmol, 50%). ^{1}H NMR (400 MHz, Acetone-d_6) δ 8.02 – 7.98 (m, 2H, ArH), 7.92 (dd, J = 7.9, 1.3 Hz, 1H, ArH), 7.66 – 7.61 (m, 2H, ArH), 7.56 – 7.51 (m, 2H, ArH), 7.50 – 7.41 (m, 3H, ArH), 7.37 – 7.34 (m, 2H, ArH), 2.48 (s, 3H, CH_3), 1.50 (s, 3H, CH_3). ^{13}C NMR (101 MHz, Acetone-d_6) δ 200.1 (C=O), 157.7 (ArC), 155.3 (ArC), 141.9 (ArC), 140.1 (ArC), 138.5 (ArC), 138.0 (ArC), 137.5 (ArC), 136.7 (ArC), 131.1 (ArC), 130.5 (ArC), 129.9 (ArCH), 129.8 (ArCH), 128.0 (ArCH), 126.7 (ArCH), 125.8 (ArCH), 125.4 (ArCH), 122.5 (ArCH), 122.0 (ArCH), 112.1 (ArCH), 102.7 (ArCH), 31.1 (CH_3), 14.6 (CH_3). ν_{max} (neat)/cm^{-1} 691, 751, 768, 1204, 1522, 1917, 2918; HRMS (ESI): Calcd. for $C_{25}H_{19}O_2S$ (M+H$^+$), 383.1106; found 383.1101.

6.2. Orthgonal cross-coupling

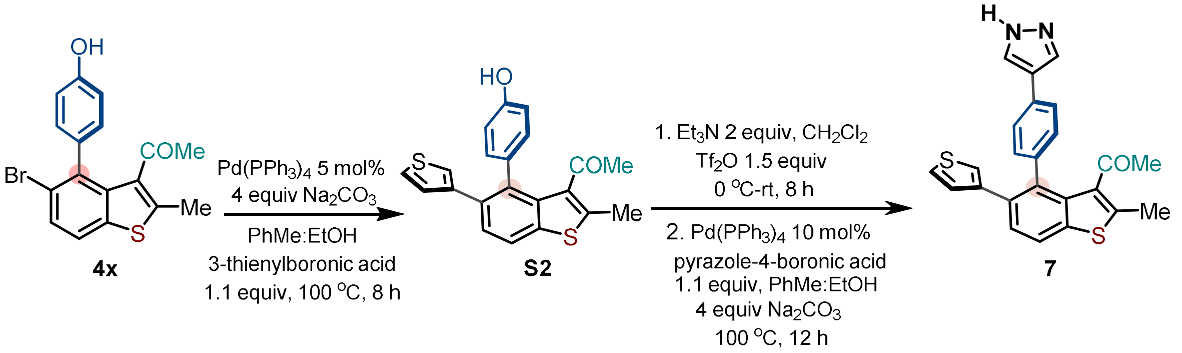

4x **S2** **7**

To an oven-dried vial was added **4x** (361 mg, 1.0 mmol, 1.0 equiv), palladium tetrakis(triphenylphosphine) (58 mg, 0.05 mmol, 5 mol%), and Na_2CO_3 (424 mg, 4.0 mmol, 4.0 equiv) followed by toluene (3.0 mL) and H_2O (3.0 mL). A solution of 3-thienylboronic acid (141 mg, 1.1 mmol, 1.1 equiv) in EtOH (4.0 mL) was then added to the above solution. The reaction mixture was heated at reflux for 6 hours and then concentrated *in vacuo*. The biphasic mixture was acidified with an aqueous solution of HCl 1N until pH = 2. The aqueous layer was extracted with EtOAc (10 mL x 3) and the organic layer was washed with H_2O, and brine, dried over $MgSO_4$, filtered, and evaporated to dryness. The residue was purified by column chromatography on silica

gel (n-hexane/EtOAc 3/1) to afford 1-(4-(4-hydroxyphenyl)-2-methyl-5-(thiophen-3-yl)benzo[b]thiophen-3-yl)ethan-1-one **S2** (294 mg, 0.81 mmol, 81%) as an off-white solid.

^{1}H NMR (400 MHz, Acetone-d_6) δ 7.88 (d, *J* = 8.3 Hz, 1H, ArH), 7.46 (d, *J* = 8.3 Hz, 1H, ArH), 7.22 (dd, *J* = 5.1, 3.0 Hz, 1H, ArH), 7.06 (d, *J* = 2.7 Hz, 1H, ArH), 6.92 (d, *J* = 8.0 Hz, 2H, ArH), 6.74 (d, *J* = 8.0 Hz, 2H, ArH), 6.66 (d, *J* = 5.0 Hz, 1H, ArH), 2.40 (s, 3H, CH_3), 1.55 (s, 3H, CH_3).

^{13}C NMR (101 MHz, Acetone-d_6) δ 201.2 (C=O), 157.9 (ArC), 143.1 (ArC), 140.1 (ArC), 138.8 (ArC), 138.7 (ArC), 138.2 (ArC), 135.9 (ArC), 134.3 (ArC), 133.4 (ArCH), 131.5 (ArC), 130.0 (ArCH), 127.6 (ArCH), 125.1 (ArCH), 123.9 (ArCH), 121.9 (ArCH), 115.7 (ArCH), 31.5 (CH_3), 14.5 (CH_3). ν_{max} (neat)/cm^{-1} 651, 773, 785, 1100, 1164, 1181, 1199, 1299, 1512, 1736, 2851, 3370; HRMS (ESI): Calcd. for $C_{21}H_{16}O_2S_2$ (M+Na$^+$), 387.0489; found 387.0481.

1-(4-(4-Hydroxyphenyl)-2-methyl-5-(thiophen-3-yl)benzo[b]thiophen-3-yl)ethan-1-one **S2** (291 mg, 0.80 mmol, 1.0 equiv) was dissolved in CH_2Cl_2 (8 mL) and the mixture was cooled to 0 °C. To this solution, triethylamine was added (134 µL, 0.96 mmol, 1.2 equiv) followed by the dropwise addition of triflic anhydride (162 µL, 0.96 mmol, 1.2 equiv). The reaction was monitored by TLC and stopped after the consumption of starting material completely. The solvent was evaporated *in vacuo* and the triflate product was used without further purification for the next step.

To an oven-dried vial was added the product from the above step (0.8 mmol, 1.0 equiv), palladium tetrakis(triphenylphosphine (92 mg, 0.08 mmol, 10 mol%), and Na_2CO_3 (339 mg, 3.2 mmol, 4.0 equiv) followed by toluene (2.5 mL) and H_2O (2.5 mL). A solution of pyrazole-4-boronic acid (99 mg, 0.88 mmol, 1.1 equiv) in EtOH (3.2 mL) was then added to the above solution. The reaction mixture was heated at reflux for 6 hours and then concentrated *in vacuo*. The biphasic mixture was acidified with an aqueous solution of HCl 1N until pH = 2. The aqueous layer was extracted with EtOAc (10 mL x 3) and the organic layer was washed with H_2O, and brine, dried over $MgSO_4$, filtered, and evaporated to dryness. The residue was purified by column chromatography on silica gel (n-hexane/EtOAc 2/1) to afford **7** (265 mg, 0.64 mmol, 80%) as a white solid. ^{1}H NMR (400 MHz, DMSO-d_6) δ 13.00 (s, 1H, ArH), 8.26 (s, 1H, ArH), 8.00 (d, *J* = 8.3 Hz, 1H, ArH), 7.56 – 7.51 (m, 2H, ArH), 7.46 (d, *J* = 8.3 Hz, 1H, ArH), 7.30 (dd, *J* = 5.0, 3.0 Hz, 1H, ArH), 7.20 (dd, *J* = 3.0, 1.3 Hz, 1H, ArH), 7.05 – 7.00 (m, 2H, ArH), 6.60 (dd, *J* = 5.0, 1.3 Hz, 1H, ArH), 2.38 (s, 3H, CH_3), 1.54 (s, 3H, CH_3). ^{13}C NMR (101 MHz, DMSO-d_6) δ 200.9 (C=O), 141.5 (ArC),

139.8 (ArC), 137.4 (ArC), 137.1 (ArC), 136.5 (ArC), 136.4 (ArC), 134.4 (ArC), 132.9 (ArC), 132.1 (ArC), 131.5 (ArCH), 129.0 (ArCH), 126.8 (ArCH), 125.1 (ArCH), 124.4 (ArCH), 123.6 (ArCH), 121.7 (ArCH), 120.7 (ArC), 31.3 (CH_3), 14.4 (CH_3). ν_{max} (neat)/cm^{-1} 787, 818, 834, 1048, 1125, 1174, 1659; HRMS (APCI): Calcd. for $C_{24}H_{18}N_2OS_2$ (M+H$^+$), 415.0939; found 415.0939.

6.3. Removal of C3 formyl group

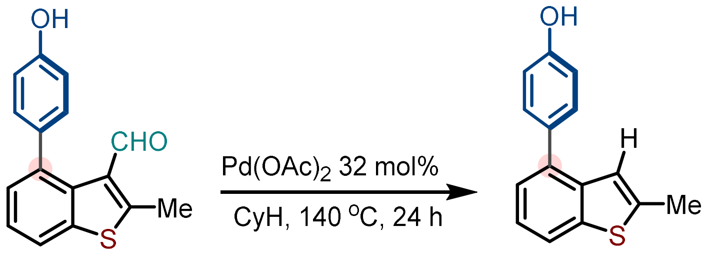

4c **8**

To an oven-dried vial under nitrogen, molecular sieves (30 mg, 4Å), **4c** (0.1 mmol, 27 mg, 1.0 equiv), and palladium acetate (7.2 mg, 0.03 mmol, 32 mol%) and cyclohexane (0.25 mL) were added. The vial was then placed in a preheated oil bath at 140 °C for 24 h. The reaction mixture was cooled to room temperature and filtered through celite with ethyl acetate (20 mL). The filtrate was concentrated and purified by column chromatography (5% EtOAc in hexane) to give the deformylated C4-arylated product as an off-white solid (13.2 mg, 0.05 mmol, 55%).

^{1}H NMR (400 MHz, $CDCl_3$) δ 7.71 (d, *J* = 7.7, 1H, ArH), 7.43 (d, *J* = 8.7 Hz, 2H, ArH), 7.29 (t, *J* = 7.6 Hz, 1H, ArH), 7.24 (dd, *J* = 7.4, 1.3 Hz, 1H, ArH), 7.08 (s, 1H, ArH), 6.94 (d, *J* = 8.5 Hz, 2H, ArH), 4.81 (s, 1H, OH), 2.56 (d, *J* = 1.2 Hz, 3H, CH_3). ^{13}C NMR (101 MHz, $CDCl_3$) δ 155.0 (ArC), 140.8 (ArC), 140.4 (ArC), 138.8 (ArC), 136.5 (ArC), 134.1 (ArC), 130.5 (ArCH), 124.6 (ArCH), 123.7 (ArCH), 121.2 (ArCH), 120.9 (ArCH), 115.4 (ArCH), 16.4 (CH_3). ν_{max} (neat)/cm^{-1} 754, 823, 1000, 1550, 2800, 3400; HRMS (ESI): Calcd. for $C_{15}H_{11}OS$ (M-H), 239.0531; found 239.0540.

6.4. Reduction of C3 formyl group

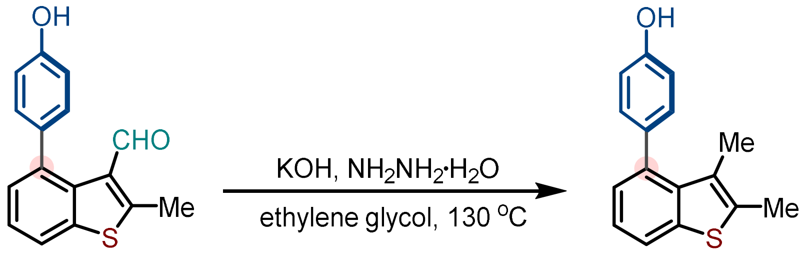

In a vial, a mixture of **4c** (116 mg, 0.433 mmol), 99% hydrazine hydrate (0.25 ml), and ethylene glycol (0.25 ml) was stirred at 130 °C for 4 h. The resulting clear solution was cooled to 60 °C and finely ground potassium hydroxide (88 mg) was added, and the temperature of the mixture was raised slowly to 130 °C. After the mixture had been stirred at 130 °C for 2 h, it was poured into water. Extraction of the product with ether (10 mL x 3) afforded the title compound (77 mg, 0.30 mmol, 70%).

^{1}H NMR (400 MHz, $CDCl_3$) δ 7.74 (dd, *J* = 8.1, 1.4 Hz, 1H, ArH), 7.23 – 7.19 (m, 3H, ArH), 7.11 (d, *J* = 7.2 Hz, 1H, ArH), 6.86 (dd, *J* = 8.1, 1.3 Hz, 2H, ArH), 4.79 (s, 1H, OH), 2.42 (s, 3H, CH_3), 1.72 (s, 3H, CH_3). ^{13}C NMR (101 MHz, $CDCl_3$) δ 154.8 (ArC), 138.8 (ArC), 137.6 (ArC), 134.7 (ArC), 134.2 (ArC), 131.1 (ArCH), 130.1 (ArC), 128.3 (ArC), 126.9 (ArCH), 122.6 (ArCH), 121.3 (ArCH), 114.4 (ArCH), 15.1 (CH_3), 14.3 (CH_3). ν_{max} (neat)/cm^{-1} 751, 784, 1152, 1171, 1222, 1259, 2920, 3420; HRMS (ESI): Calcd. for $C_{16}H_{13}OS$ (M-H), 253.0687; found 253.0680.

6.5. Deacylative C3-alkylation

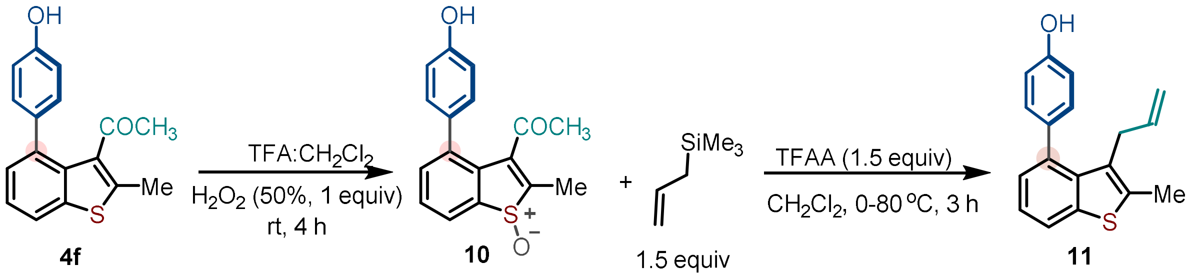

To an oven-dried vial under nitrogen was added the **4f** (282 mg, 1.0 mmol, 1.0 equiv), CH_2Cl_2 (2.2 mL), and TFA (2.2 mL). H_2O_2 (50% aq, 57 µL, 1.0 equiv) was added to this mixture at room temperature. The reaction was monitored by TLC (5% EtOAc in $CHCl_3$) and more H_2O_2 was added until complete consumption of the starting material was observed. The reaction was then

quenched with $NaHCO_3$ at 0 °C and the aqueous layer was extracted with CH_2Cl_2. The combined organic layers were dried with $MgSO_4$ and concentrated *in vacuo* to give desired benzo[b]thiophene *S*-oxide as an off-white solid (209 mg, 0.70 mmol, 70%).

^{1}H NMR (400 MHz, DMSO-d_6) δ 9.73 (s, 1H, OH), 8.00 (dd, *J* = 7.3, 1.3 Hz, 1H, ArH), 7.61 – 7.49 (m, 2H, ArH), 7.09 (s, 2H, ArH), 6.79 (d, *J* = 8.2 Hz, 2H, ArH), 2.18 (s, 3H, CH_3), 1.65 (s, 3H, CH_3). ^{13}C NMR (101 MHz, DMSO-d_6) δ 199.1 (C=O), 157.8 (ArC), 147.6 (ArC), 145.3 (ArC), 141.1 (ArC), 138.4 (ArC), 134.1 (ArCH), 132.9 (ArC), 130.3 (ArCH), 129.8 (ArC), 128.4 (ArCH), 125.3 (ArCH), 115.4 (ArCH), 29.5 (CH_3), 11.0 (CH_3). ν_{max} (neat)/cm^{-1} 721, 798, 991, 1201, 1699, 2852, 2922, 3157; HRMS (ESI): Calcd. for $C_{18}H_{16}OS$ (M-H$^+$), 279.0844; found 279.0849.

Benzo[b]thiophene *S*-oxide **10** (125 mg, 0.42 mmol, 1.0 equiv) was dissolved in MeCN (4 mL) followed by addition of allyl silane (98.7 µL, 0.63 mmol, 1.5 equiv) and TFAA (119.7 µL, 0.84 mmol, 2.0 equiv) at 0 °C. The reaction was heated at reflux for 3 hours before quenching with saturated aqueous $NaHCO_3$ solution. The aqueous layer was extracted with EtOAc and the combined organic extracts were dried with $MgSO_4$. The solvent was removed in vacuo before purification by column chromatography (n-hexane) to give the deacylative C3-alkylated product as a white solid (70 mg, 0.25 mmol, 60%).

^{1}H NMR (400 MHz, $CDCl_3$) δ 7.75 (dd, *J* = 8.0, 1.2 Hz, 1H, ArH), 7.25 – 7.18 (m, 3H, ArH), 7.08 (dd, *J* = 7.3, 1.2 Hz, 1H, ArH), 6.83 (d, *J* = 8.5 Hz, 2H, ArH), 5.48 (ddt, *J* = 17.0, 10.4, 5.3 Hz, 1H, CH_2-C*H*=CH_2), 4.84 – 4.78 (m, 2H, CH_2-CH=C*H₂*, overlapped with OH peak), 4.61 – 4.54 (m, 1H, CH_2-CH=CH_2), 2.99 (dt, *J* = 5.4, 1.9 Hz, 2H, C*H₂*-CH=CH_2), 2.40 (s, 3H, CH_3). ^{13}C NMR (101 MHz, $CDCl_3$) δ 155.0 (ArC), 139.2 (ArC), 137.5 (ArC), 137.4 (ArC), 136.5 (ArC), 136.1 (=CH), 134.4 (ArC), 130.9 (ArCH), 129.8 (ArC), 127.3 (ArCH), 122.5 (ArCH), 121.4 (ArCH), 114.5 (=CH_2), 114.4 (ArCH), 31.3 (CH_2), 14.2 (CH_3). ν_{max} (neat)/cm^{-1} 756, 786, 817, 833, 1151, 1230, 1441, 1508, 2918, 3251; HRMS (ESI): Calcd. for $C_{18}H_{16}OS$ (M-H$^+$), 279.0844; found 279.0849.

7. X-ray Structures

7.1 X-ray structure of **2ab** – **CCDC 2236439**

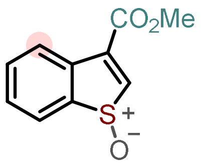

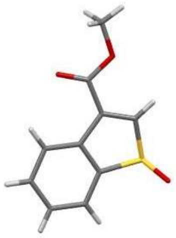

Crystal data and structure refinement of **2ab**

<table>
<tr><td rowspan="2">Cell:</td><td>a = 3.8868(4)</td><td>b = 24.834(3)</td><td>c = 9.3604(5)</td></tr>
<tr><td>alpha = 90</td><td>beta = 96.972(7)</td><td>gamma = 90</td></tr>
<tr><td>Temperature</td><td colspan="3">100 K</td></tr>
<tr><td></td><td>Calculated</td><td colspan="2">Reported</td></tr>
<tr><td>Volume</td><td>896.83(15)</td><td colspan="2">896.83(14)</td></tr>
<tr><td>Space group</td><td>P 21/c</td><td colspan="2">P1 21/c 1</td></tr>
<tr><td>Hall group</td><td>-P 2ybc</td><td colspan="2">-P 2ybc</td></tr>
<tr><td>Moiety formula</td><td>C10 H8 O3 S</td><td colspan="2">C10 H8 O3 S</td></tr>
<tr><td>Sum formula</td><td>C10 H8 O3 S</td><td colspan="2">C10 H8 O3 S</td></tr>
<tr><td>Mr</td><td>208.22</td><td colspan="2">208.22</td></tr>
<tr><td>Dx, g cm^{-3}</td><td>1.542</td><td colspan="2">1.542</td></tr>
<tr><td>Z</td><td>4</td><td colspan="2">4</td></tr>
<tr><td>Mu (mm^{-1})</td><td>3.027</td><td colspan="2">3.027</td></tr>
<tr><td>F000</td><td>432.0</td><td colspan="2">432.0</td></tr>
<tr><td>F000’</td><td>434.62</td><td colspan="2"></td></tr>
<tr><td>h, k, lmax</td><td>4,31,11</td><td colspan="2">4,30,11</td></tr>
<tr><td>Nref</td><td>1861</td><td colspan="2">1765</td></tr>
<tr><td>Tmin, Tmax</td><td>0.957,0.973</td><td colspan="2">0.728,1.000</td></tr>
<tr><td>Tmin’</td><td>0.847</td><td colspan="2">Wavelength 1.54184</td></tr>
<tr><td colspan="4">Correction method = # Reported T Limits: Tmin = 0.995 Tmax = 1.000 AbsCorr = MULTI-SCAN</td></tr>
</table>

Data completeness	0.948	Theta (max)	75.862
R (reflections)	0.0574(1216)	wR2 (reflections)	0.1618(1765)
S	1.043	Npar	128

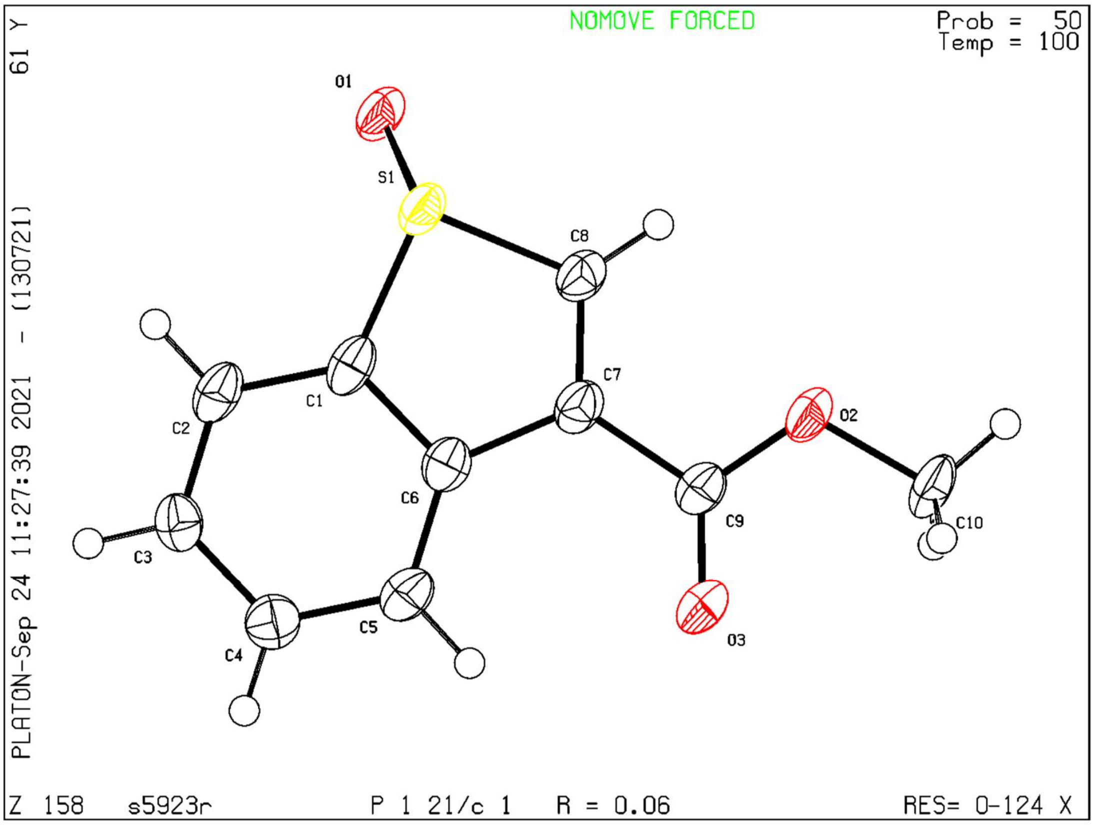

7.2 X-ray structure of **2ak- CCDC 2236443**

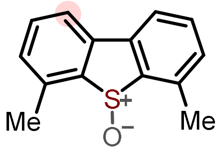

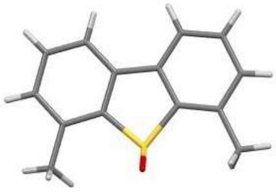

Crystal data and structure refinement of **2aj**

Cell:	a = 7.5541(2)	b = 7.6255(2)	c = 18.6721(5)
	alpha = 90	beta = 91.552(2)	gamma = 90
Temperature	100 K		
	Calculated	Reported	
Volume	1075.19(5)	1075.19(5)	
Space group	P 21/n	P1 21/n 1	
Hall group	-P 2yn	-P 2yn	
Moiety formula	C14 H12 O S	C14 H12 O S	
Sum formula	C14 H12 O S	C14 H12 O S	
Mr	228.30	228.30	
Dx, g cm^{-3}	1.410	1.410	
Z	4	4	
Mu (mm^{-1})	2.433	2.433	
F000	480.0	480.0	
F000’	482.50		
h, k, lmax	9,9,23	9,9,23	
Nref	2249	2142	
Tmin, Tmax	0.552,0.729	0.748,1.000	
Tmin’	0.378	Wavelength 1.54184	
Correction method = # Reported T Limits: Tmin = 0.748 Tmax = 1.000 AbsCorr = MULTI-SCAN			
Data completeness	0.952	Theta (max)	75.712

R (reflections)	0.0326(2015)	wR2 (reflections)	0.0879(2142)
S	1.071	Npar	147

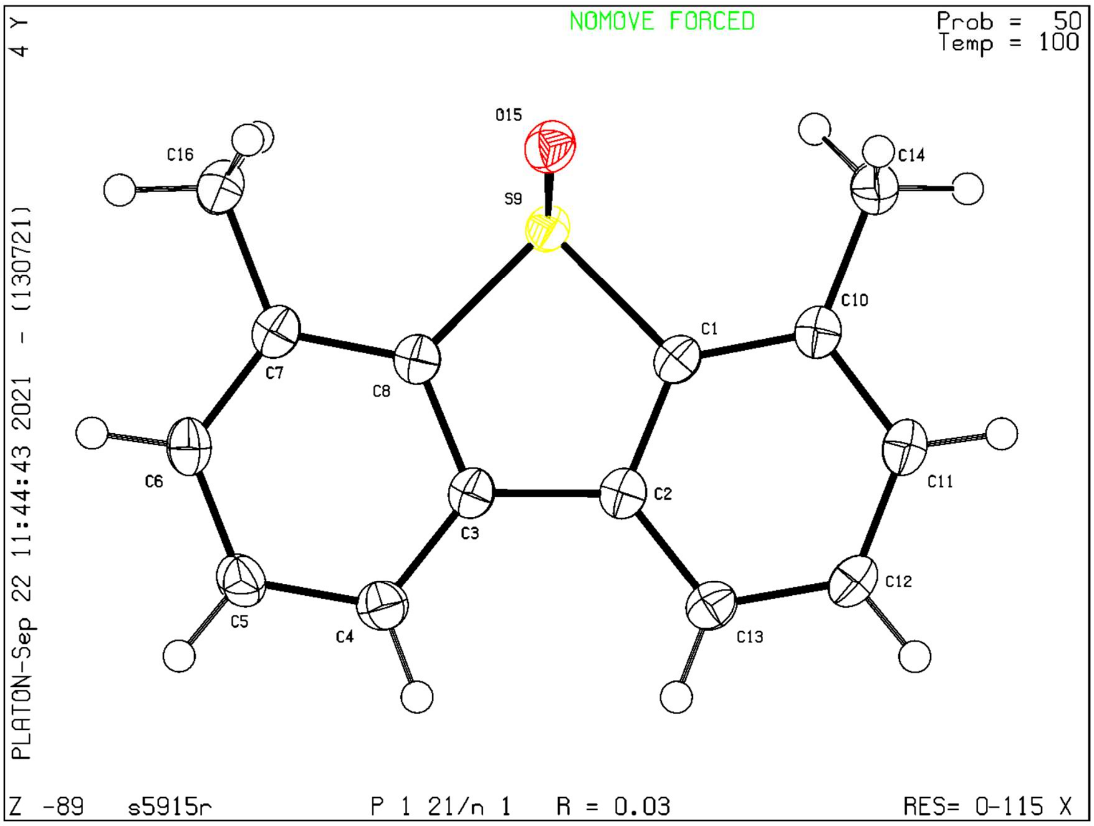

7.3 X-ray structure of **4c- CCDC 2236442**

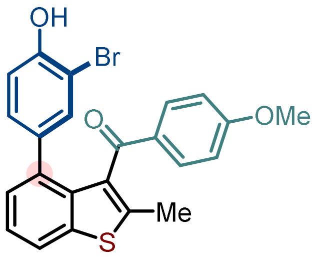

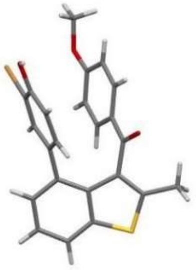

Crystal data and structure refinement of **4c**

Cell:	a = 10.76491(14)	b = 12.06541(15)	c = 14.4500(2)
	alpha = 90	beta = 99.5080(13)	gamma = 90
Temperature	100 K		
	Calculated	Reported	
Volume	1851.03(4)	1851.03(4)	
Space group	P 21/c	P1 21/c 1	
Hall group	-P 2ybc	-P 2ybc	
Moiety formula	C23 H17 Br O3 S	C23 H17 Br O3 S	
Sum formula	C23 H17 Br O3 S	C23 H17 Br O3 S	
Mr	453.33	453.33	
Dx, g cm^{-3}	1.627	1.627	
Z	4	4	
Mu (mm^{-1})	4.270	4.270	
F000	920.0	920.0	
F000'	920.81		
h, k, lmax	13,15,18	13,15,17	
Nref	3860	3741	
Tmin, Tmax	0.774,0.975	0.915,1.000	
Tmin'	0.581	Wavelength 1.54184	
Correction method = # Reported T Limits: Tmin = 0.915 Tmax = 1.000 AbsCorr = MULTI-SCAN			

Data completeness	0.969	Theta (max)	75.762
R (reflections)	0.0281(3373)	wR2 (reflections)	0.0758(3741)
S	1.058	Npar	256

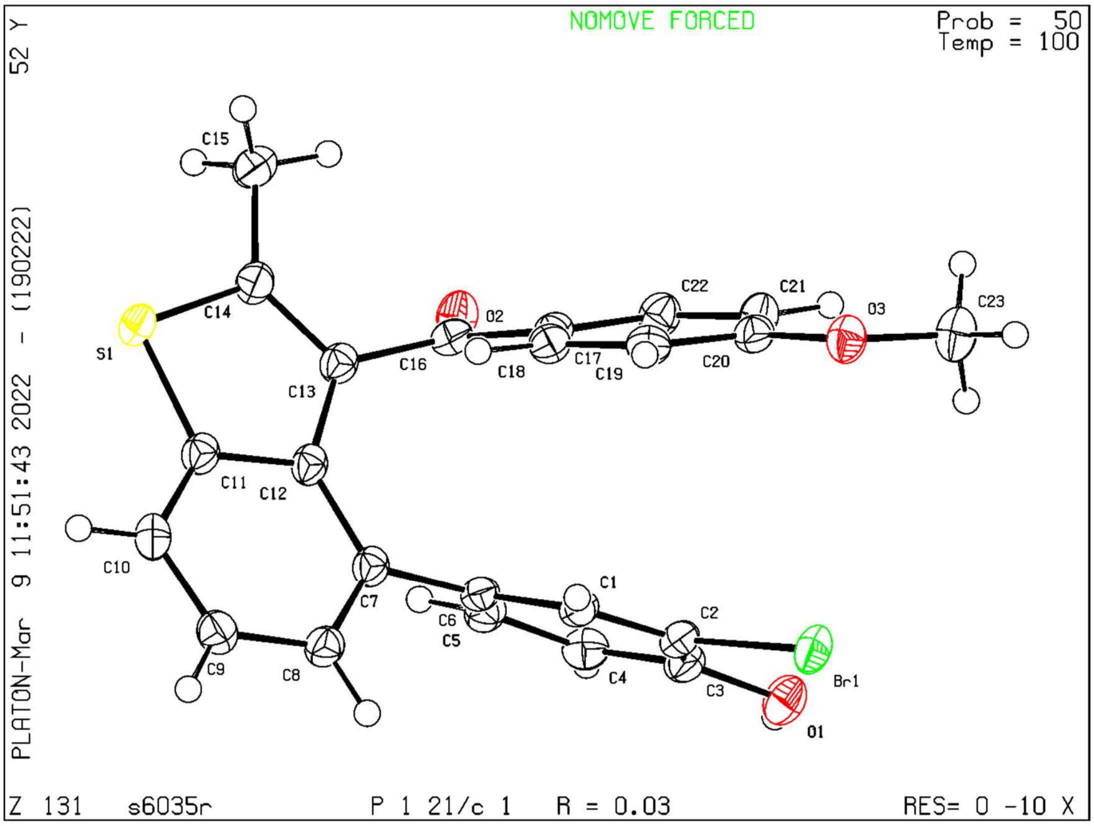

7.4 X-ray structure of **4e- CCDC 2236438**

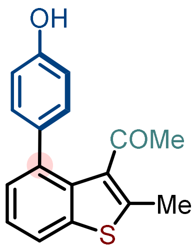

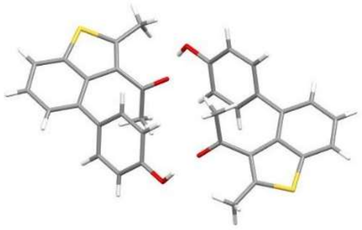

Crystal data and structure refinement of **4e**

Cell:	a = 18.968(2)	b = 7.0846(5)	c = 22.869(2)
	alpha = 90	beta = 112.408(11)	gamma = 90
Temperature	100 K		
	Calculated	Reported	
Volume	2841.1(5)	2841.2(5)	
Space group	P 21/n	P1 21/n 1	
Hall group	-P 2yn	-P 2yn	
Moiety formula	1.058(C17 H14 O2 S), [illegible]	2(C17 H14 O2 S)	
Sum formula	C19.43 H16 O2.29 S1.14	C34 H28 O4 S2	
Mr	322.68	564.68	
Dx, g cm^{-3}	1.320	1.320	
Z	7	4	
Mu (mm^{-1})	0.226	0.226	
F000	1184.0	1184.0	
F000'	1185.49		
h, k, lmax	23,8,28	23,8,28	
Nref	5809	5796	
Tmin, Tmax	0.926,0.953	0.739,1.000	
Tmin'	0.895	Wavelength 0.71073	
Correction method = # Reported T Limits: Tmin = 0.739 Tmax = 1.000 AbsCorr = MULTI-SCAN			
Data completeness	0.998	Theta (max)	26.371

R (reflections)	0.1396(4680)	wR2 (reflections)	0.3497(5796)
S	1.135	Npar	664

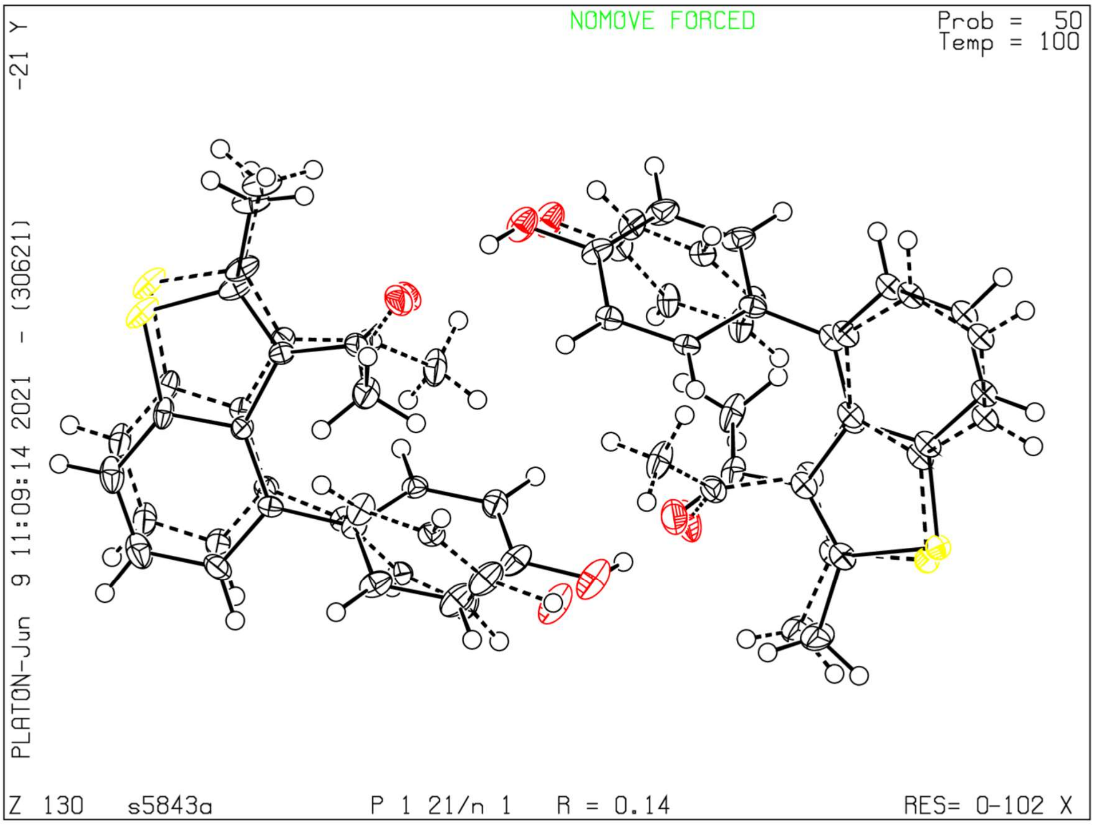

7.5 X-ray structure of **4n** – **CCDC 2236441**

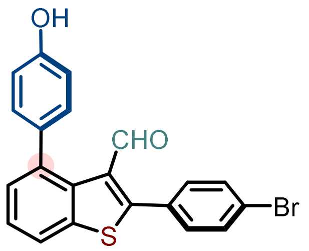

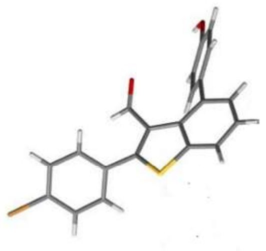

Crystal data and structure refinement of **4n**

Cell:	a = 19.9193(12)	b = 14.0776(9)	c = 20.0140(11)
	alpha = 90	beta = 114.924(7)	gamma = 90
Temperature	100 K		
	Calculated	Reported	
Volume	5089.6(6)	5089.6(6)	
Space group	P 21/c	P1 21/c 1	
Hall group	-P 2ybc	-P 2ybc	
Moiety formula	C21 H13 Br O2 S	C21 H13 Br O2 S	
Sum formula	C21 H13 Br O2 S	C21 H13 Br O2 S	
Mr	409.27	409.28	
Dx, g cm^{-3}	1.602	1.602	
Z	12	12	
Mu (mm^{-1})	4.544	4.544	
F000	2472.0	2472.0	
F000’	2473.44		
h, k, lmax	25,17,25	25,17,24	
Nref	10673	10283	
Tmin, Tmax	0.897,0.991	0.760,1.000	
Tmin’	0.601	Wavelength 1.54184	
Correction method = # Reported T Limits: Tmin = 0.760 Tmax = 1.000 AbsCorr = MULTI-SCAN			
Data completeness	0.963	Theta (max)	76.360

R (reflections)	0.0749(5781)	wR2 (reflections)	0.2398(10283)
S	1.026	Npar	679

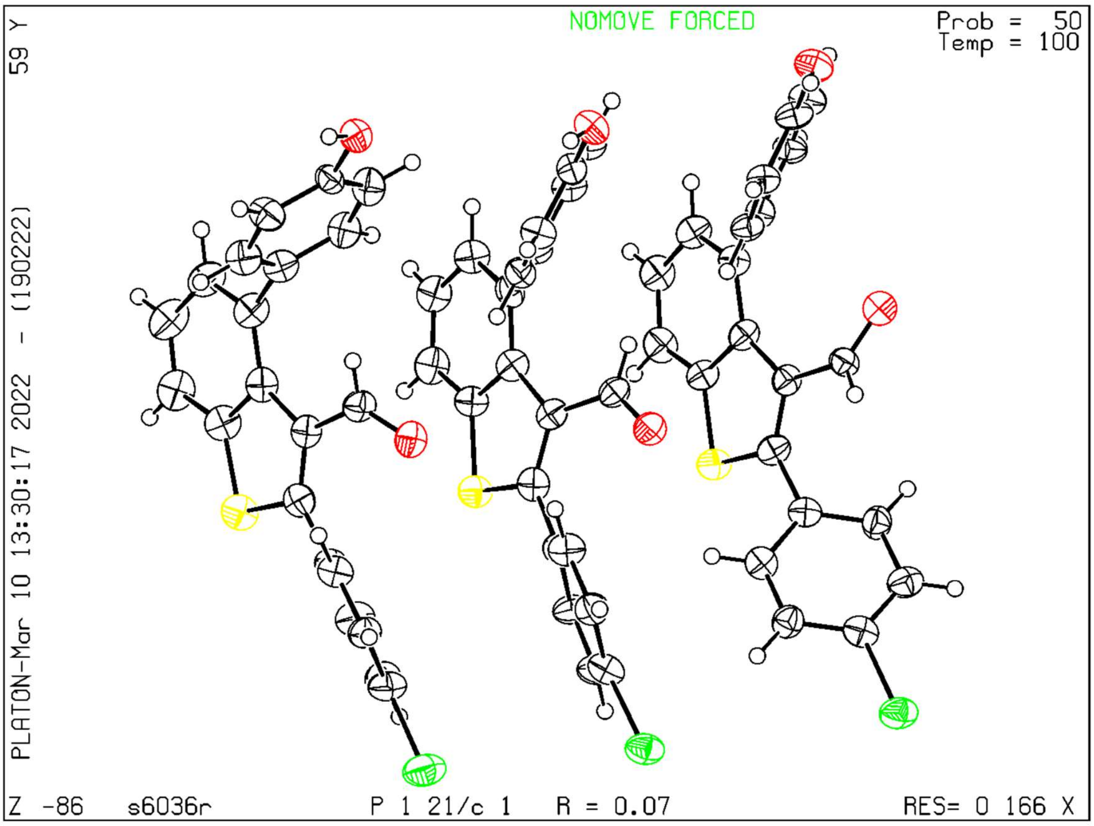

7.6 X-ray structure of **4w** – **CCDC 2236444**

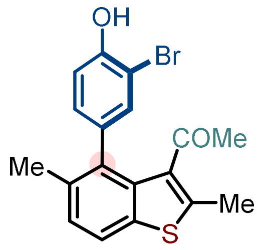

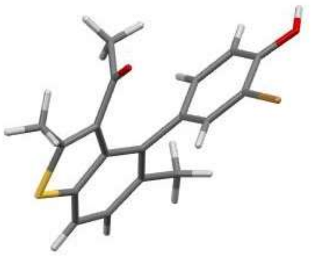

Crystal data and structure refinement of **4w**

Cell:	a = 13.1708(6)	b = 7.2818(3)	c = 16.9434(9)
	alpha = 90	beta = 105.203(5)	gamma = 90
Temperature	100 K		

	Calculated	Reported
Volume	1568.12(13)	1568.12(13)
Space group	P 21/c	P1 21/c 1
Hall group	-P 2ybc	-P 2ybc
Moiety formula	C18 H15 Br O2 S	C18 H15 Br O2 S
Sum formula	C18 H15 Br O2 S	C18 H15 Br O2 S
Mr	375.26	375.27
Dx, g cm^{-3}	1.589	1.590
Z	4	4
Mu (mm^{-1})	2.758	2.758
F000	760.0	760.0
F000’	759.64	
h, k, lmax	19,10,24	17,10,21
Nref	4953	3890
Tmin, Tmax	0.725,0.738	0.995,1.000
Tmin’	0.711	Wavelength 0.71073
Correction method = # Reported T Limits: Tmin = 0.995 Tmax = 1.000 AbsCorr = MULTI-SCAN		

Data completeness	0.785	Theta (max)	30.889

R (reflections)	0.0384(2821)	wR2 (reflections)	0.0803(3890)
S	1.018	Npar	202

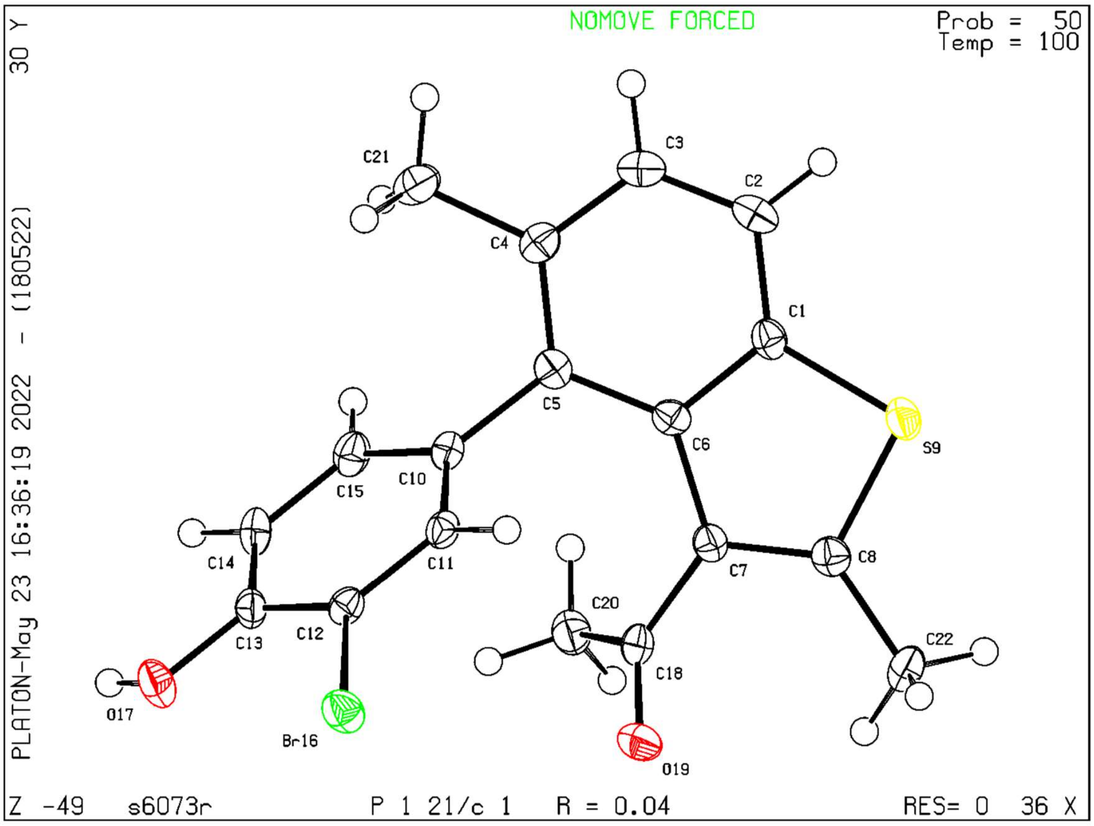

7.7 X-ray structure of **4ab** – **CCDC 2236447**

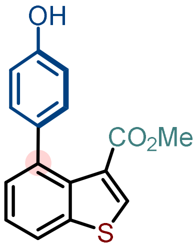

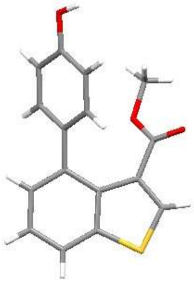

Crystal data and structure refinement of **4ab**

Cell:	a = 9.3616(7)	b = 10.9636(5)	c = 12.6924(7)
	alpha = 90	beta = 90	gamma = 90
Temperature	150 K		
	Calculated	Reported	
Volume	1302.71(13)	1302.71(13)	
Space group	P 21 21 21	P 21 21 21	
Hall group	P 2ac 2ab	P 2ac 2ab	
Moiety formula	$C_{16}H_{12}O_3S$	$C_{16}H_{12}O_3S$	
Sum formula	$C_{16}H_{12}O_3S$	$C_{16}H_{12}O_3S$	
Mr	284.32	284.32	
Dx, g cm^{-3}	1.450	1.450	
Z	4	4	
Mu (mm^{-1})	2.250	2.250	
F000	592.0	592.0	
F000’	595.03		
h, k, lmax	11,13,15	11,13,15	
Nref	2717[1570]	2626	
Tmin, Tmax	0.850, 0.874	0.880, 1.000	
Tmin’	0.799	Wavelength 1.54184	
Correction method = # Reported T Limits: Tmin = 0.880 Tmax = 1.000 AbsCorr = MULTI-SCAN			

Data completeness	1.67/0.97	Theta (max)	76.213
R (reflections)	0.0436(2364)	wR2 (reflections)	0.1083(2626)
S	1.067	Npar	183

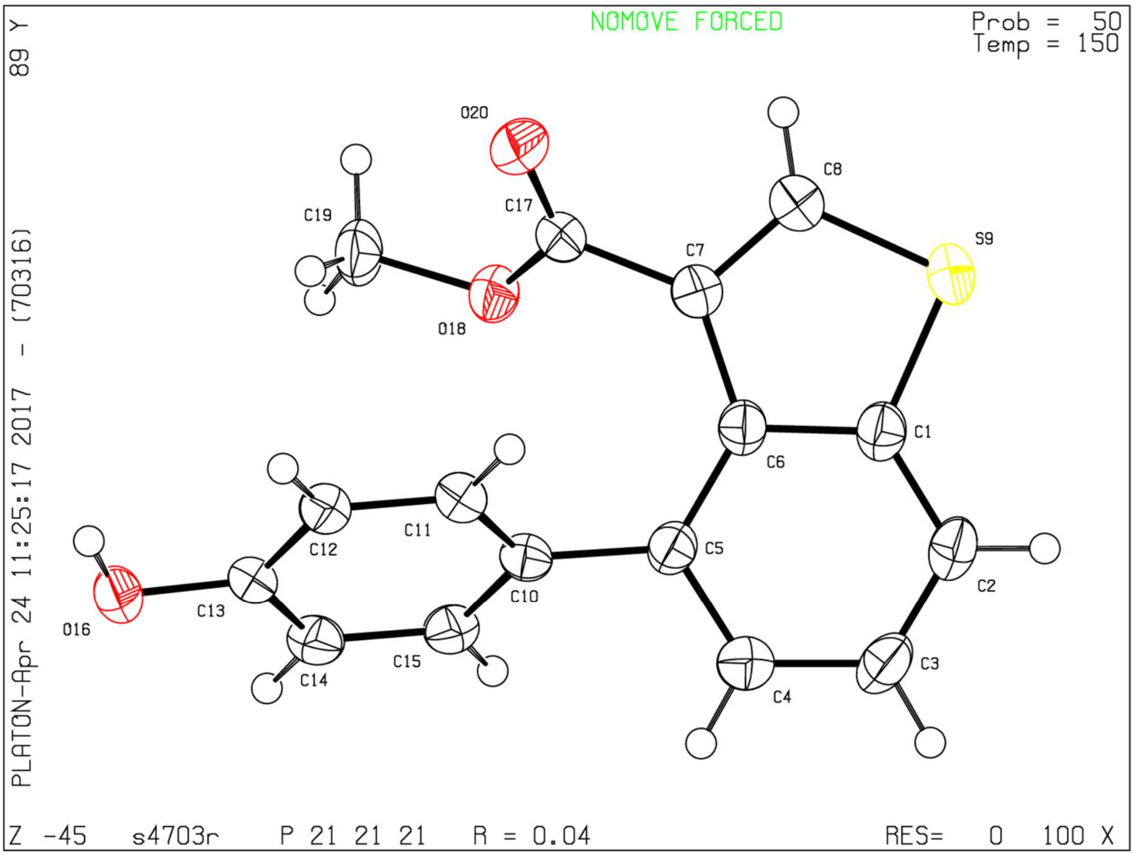

7.8 X-ray structure of **4al** – **CCDC 2236446**

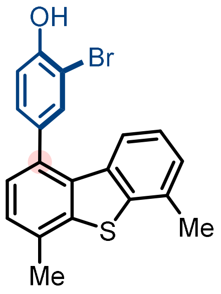

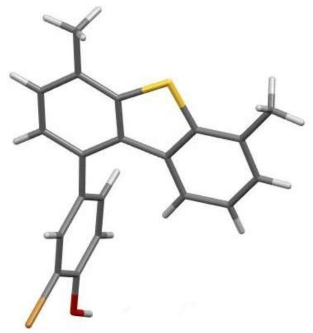

Crystal data and structure refinement of **4al**

Cell:	a = 22.9498(5)	b = 11.8086(3)	c = 13.1427(3)
	alpha = 90	beta = 97.367(2)	gamma = 90

Temperature	100 K	
	Calculated	Reported
Volume	3532.34(14)	3532.35(16)
Space group	P 21/c	P1 21/c 1
Hall group	-P 2ybc	-P 2ybc
Moiety formula	2(C20 H15 Br O S), C H2 Cl2	C20 H15 Br O S, 0.5(C H2Cl2)
Sum formula	C41 H32 Br2 Cl2 O2 S2	C20.50 H16 Br Cl O S
Mr	851.49	425.75
Dx, g cm^{-3}	1.601	1.601
Z	4	8
Mu (mm^{-1})	5.700	5.700
F000	1720.0	1720.0
F000’	1723.48	
h, k, lmax	28,14,16	28,14,15
Nref	7350	7033
Tmin, Tmax	0.432,0.843	0.599,1.000
Tmin’	0.216	Wavelength 1.54184
Correction method = # Reported T Limits: Tmin = 0.599 Tmax = 1.000 AbsCorr = MULTI-SCAN		

Data completeness	0.957	Theta (max)	75.761

R (reflections)	0.0547(5865)	wR2 (reflections)	0.1647(7033)
S	1.046	Npar	446

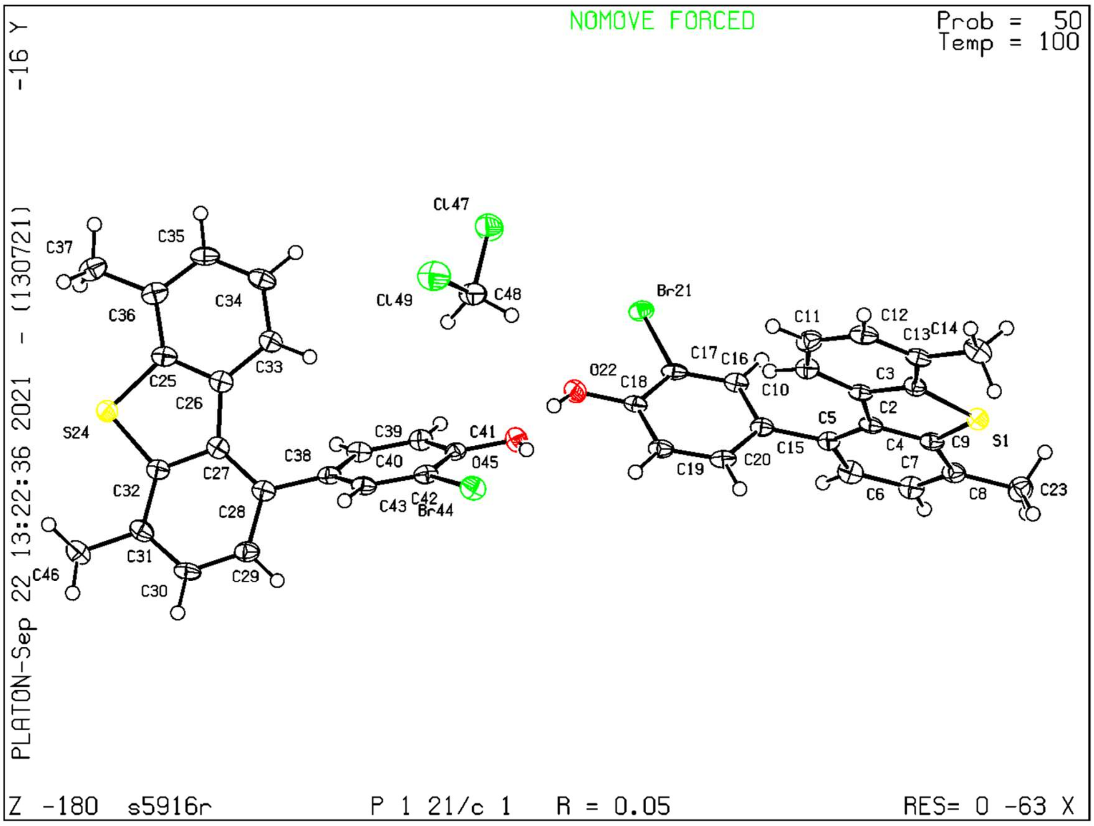

7.9 X-ray structure of **4an** – **CCDC 2236445**

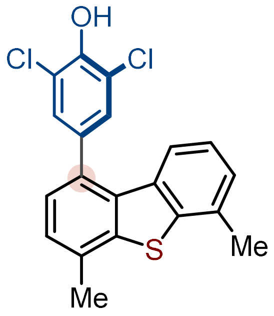

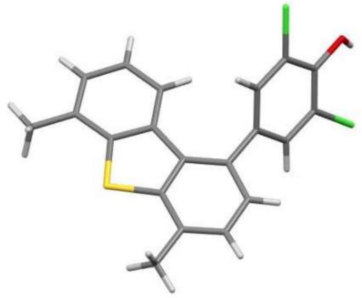

Crystal data and structure refinement of **4an**

Cell:	a = 20.4215(8)	b = 4.16241(14)	c = 19.9981(7)
	alpha = 90	beta = 102.526(4)	gamma = 90

Temperature	100 K	
	Calculated	Reported
Volume	1659.43(11)	1659.43(11)
Space group	C 2	C 1 2 1
Hall group	C 2y	C 2y
Moiety formula	C20 H14 Cl2 O S	C20 H14 Cl2 O S
Sum formula	C20 H14 Cl2 O S	C20 H14 Cl2 O S
Mr	373.27	373.27
Dx, g cm^{-3}	1.494	1.494
Z	4	4
Mu (mm^{-1})	4.715	4.715
F000	768.0	768.0
F000’	773.87	
h, k, lmax	25,5,25	25,5,25
Nref	3468[1981]	3392
Tmin, Tmax	0.668,0.954	0.581,1.000
Tmin’	0.371	Wavelength 1.54184

Correction method = # Reported T Limits: Tmin = 0.581 Tmax = 1.000 AbsCorr = MULTI-SCAN			
Data completeness	1.71/0.98	Theta (max)	75.888
R (reflections)	0.0731(3205)	wR2 (reflections)	0.2116(3392)
S	1.074	Npar	221

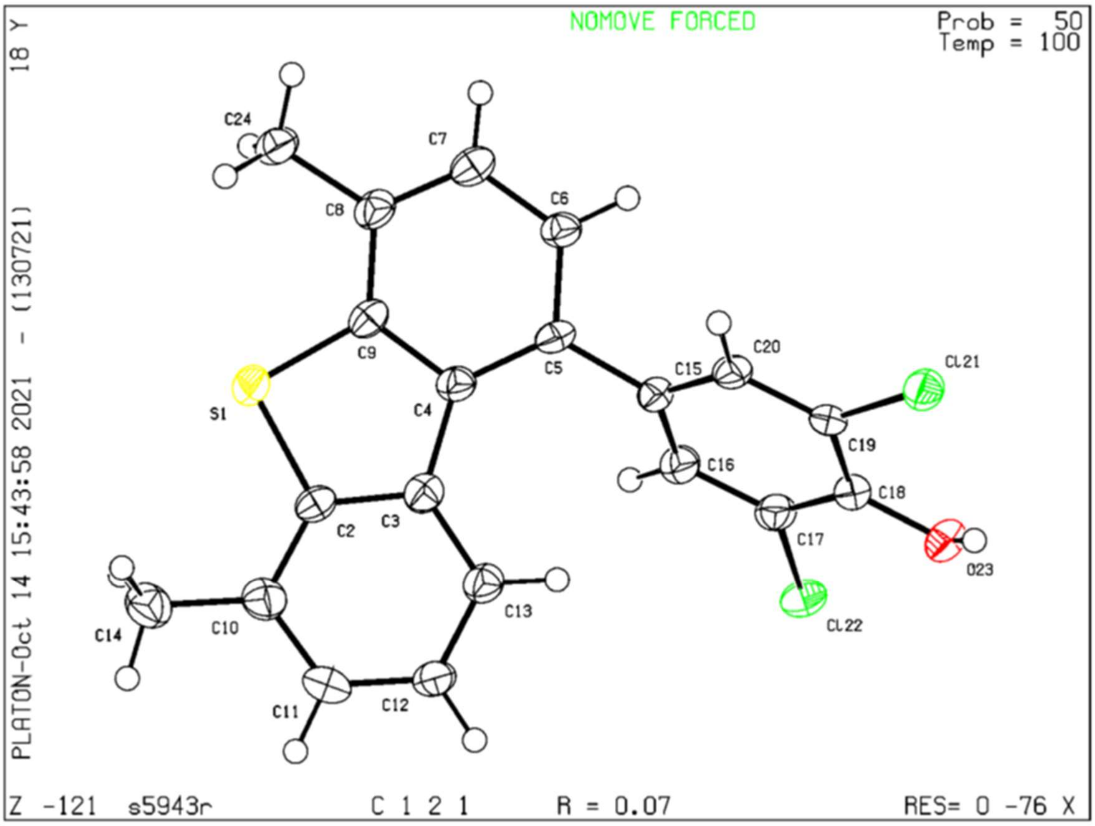

7.10 X-ray structure of **5** – **CCDC 2236440**

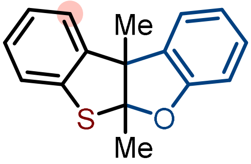

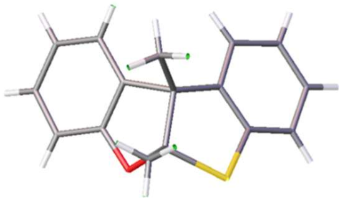

Crystal data and structure refinement of **5**

Cell:	a = 12.6565(6)	b = 15.2195(9)	c = 6.5541(3)
	alpha = 90	beta = 90	gamma = 90
Temperature	100 K		
	Calculated	Reported	
Volume	1262.49(11)	1262.49(11)	
Space group	P n m a	P n m a	
Hall group	-P 2ac 2n	-P 2ac 2n	
Moiety formula	C16 H14 O S	C16 H14 O S	
Sum formula	C16 H14 O S	C16 H14 O S	
Mr	254.33	254.33	
Dx, g cm^{-3}	1.338	1.338	
Z	4	4	
Mu (mm^{-1})	2.130	2.130	
F000	536.0	536.0	
F000’	538.64		
h, k, lmax	15,19,8	15,19,7	
Nref	1356	1319	
Tmin, Tmax	0.580,0.810	0.395,1.000	
Tmin’	0.474	Wavelength 1.54184	
Correction method = # Reported T Limits: Tmin = 0.395 Tmax = 1.000 AbsCorr = MULTI-SCAN			
Data completeness	0.973	Theta (max)	75.585
R (reflections)	0.0813(1133)	wR2 (reflections)	0.2576(1319)

S	1.103	Npar	165

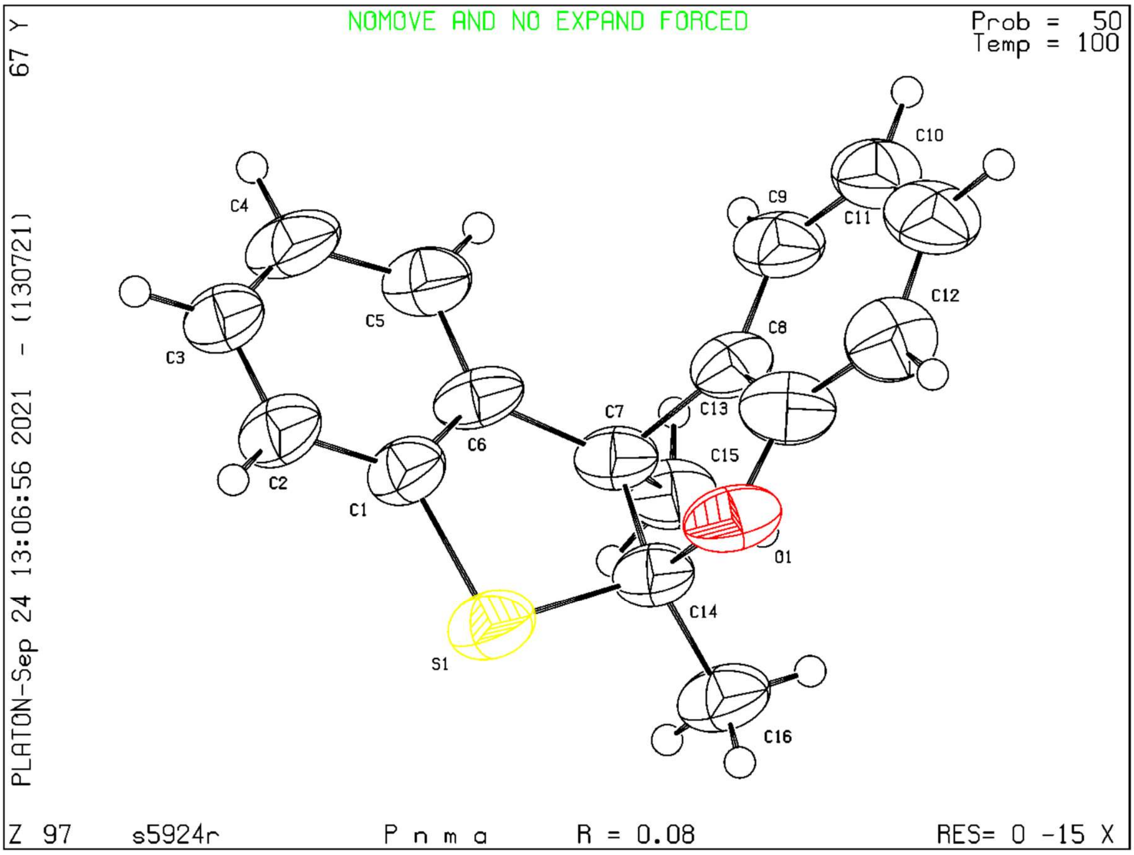

8. Computational Details

8.1. General Information

The hybrid meta-GGA M06-2X[10] density functional with Grimme's zero-damped D3[11] correction, in conjunction with Pople's double-ζ 6-31+G(d,p)[12] basis set, was used to optimize all stationary point structures. To these optimized structures, single-point energy corrections (M06-2X-D3/def2-QZVPP)[13] were applied. For both optimization and single point calculations the integral equation formalism variant of the polarizable continuum model (IEF-PCM), with the SMD solvation model was applied to account for solvent effects (solvent=dichloromethane).[14] *Gaussian 16*[15] version C.01 was employed for all density functional theory (DFT) calculations, using an "ultrafine" pruned (99,590) grid for numerical integration of the exchange-correlation functional and its derivatives. Conformational analyses of all stationary points were performed manually. Quasiclassical *ab-initio* molecular dynamics simulations were performed using *Jprogdyn,*[16] a Java program interfaced with *Gaussian 16* to calculate molecular trajectories based DFT computed frequency calculations (see *section **8.4***). Population analysis was performed using *NBO 6.0*[17] interfaced with *Gaussian 16* and the computed NBO charges were used to calculate Fukui functions.[18] The resulting NBO charges were used to compute Fukui functions. Molecular graphics were generated using *PyMol.*[19] Vibrational frequency calculations were performed to verify that stationary points were either minima or first-order saddle points on the potential energy surface (PES), and to calculate thermal corrections to Gibbs free energies (G). Intrinsic reaction coordinate (IRC)[20] calculations were performed to ensure that the transition structures connected to their corresponding starting and final geometries. The computed thermochemistry data were corrected following Grimme's quasi-harmonic (QHA) model for entropy[21] with a frequency cut-off value of 100.0 cm^{-1} using the *GoodVibes*[22] program at 298.15 K (25˚C), unless otherwise stated. Also, *GoodVibes* applied (i) 1 M standard concentration corrections to all individual calculations to account for reactions in solution (i.e. change in standard concentration from 1 atm to 1 M)[23] and (ii) multi-conformational corrections (G_{conf}) to all final Boltzmann weighted G to include the entropic stabilization created by multiple accessible low-lying conformers.[24] G values of all the energy profiles correspond to the Boltzmann weighted G of all the conformers found in each step. Boltzmann weighted G (G_{av}) were calculated as:

$$G_{av} = \sum_i G_i \times p_i \qquad (1)$$

where G_i is the relative Gibbs free energy of the corresponding conformers of a certain reaction step and pi is the probability of each conformer calculated as:

$$p_i = \frac{e^{\frac{-G_i}{RT}}}{\sum_i \left(e^{\frac{-G_i}{RT}} \right)} \tag{2}$$

8.2. Potential energy surfaces

The potential energy surface (PES) for the reaction between the reaction of 1-(2-methyl-1-oxidobenzo[*b*]thiophen-3-yl)ethan-1-one (**2a**) and phenol presented in the main manuscript (**Figure S1**) was studied with the trifluoroacetate counterion present (**Figure S2A**) and absent (**Figure S2B**). In both cases, the PESs were found to be very similar, the major difference being the absence of a transition state connecting intermediates **G** and **F** when the counterion is not considered explicitly. The predicted ΔΔG for the selectivity determining step (**TS-II** vs **TS-IV**) was found to be 1.2 kcal/mol for the explicit counterion and 0.7 kcal/mol for the implicit counterion model. The latter was found to be in good agreement with the experimentally determined selectivity of 0.3 kcal/mol (1.7:1 rr), suggesting that inclusion of the counterion is not necessary to rationalize selectivity in this transformation.

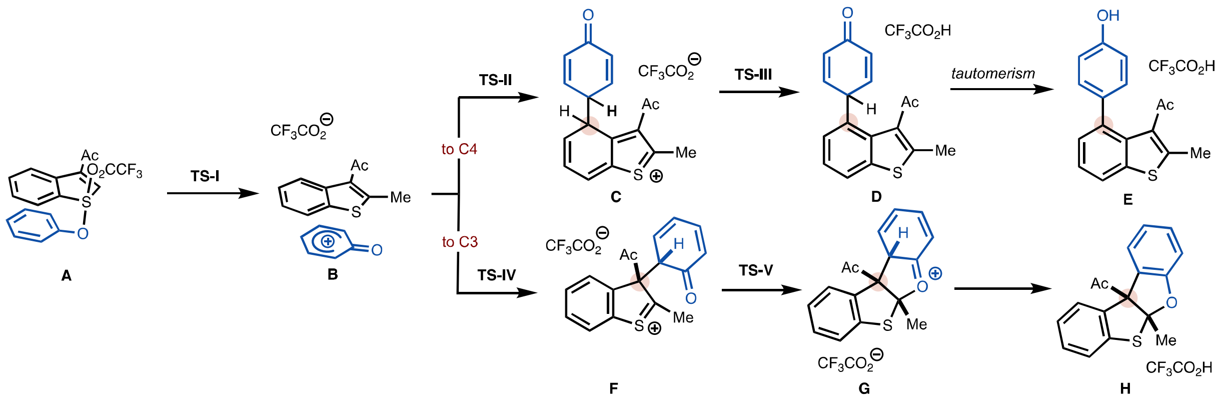

***Figure S1**: Proposed reaction mechanism*

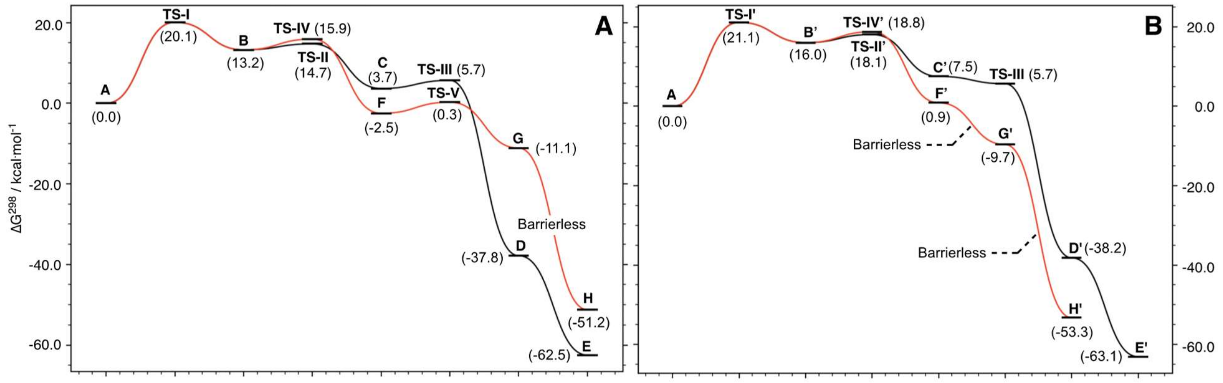

Figure S2A, B: Potential energy surfaces for the reaction between 2-methyl-3-acyl-benzothiophene S-oxide and phenol computed at the M06-2X-D3/Def2-QZVPP(SMD=CH_2Cl_2)//M06-2X-D3/6-31+G(d,p)(SMD=CH_2Cl_2) level of theory: A) in the presence

of explicit molecules of trifluoroacetate or trifluoroacetic acid; B) in the absence of explicit molecules of trifluoroacetate or trifluoroacetic acid (the stationary points that were computed with implicit molecules have been indicated by the prime superscript)

The PES was also modelled for the reaction between methyl benzo[*b*]thiophene-3-carboxylate 1-oxide (**2b**) and phenol (**Figure S3**). The trifluoroacetate counteranion was omitted from the calculations since this has little effect (as shown above). In this example, the selectivity between recombination at the C4 (*via* **2-H-3-COOMe_TS-II**) vs C3 (*via* **2-H-3-COOMe_TS-IV**) of the benzothiophene moiety was found to increase to 2.7 kcal/mol in favor of the C4 product (*via* intermediate **2-H-3-COOMe_C**). Experimentally the reaction of **2b** with phenol leads to the exclusive formation of the C4-arylated product, which is in good agreement with the theoretical model.

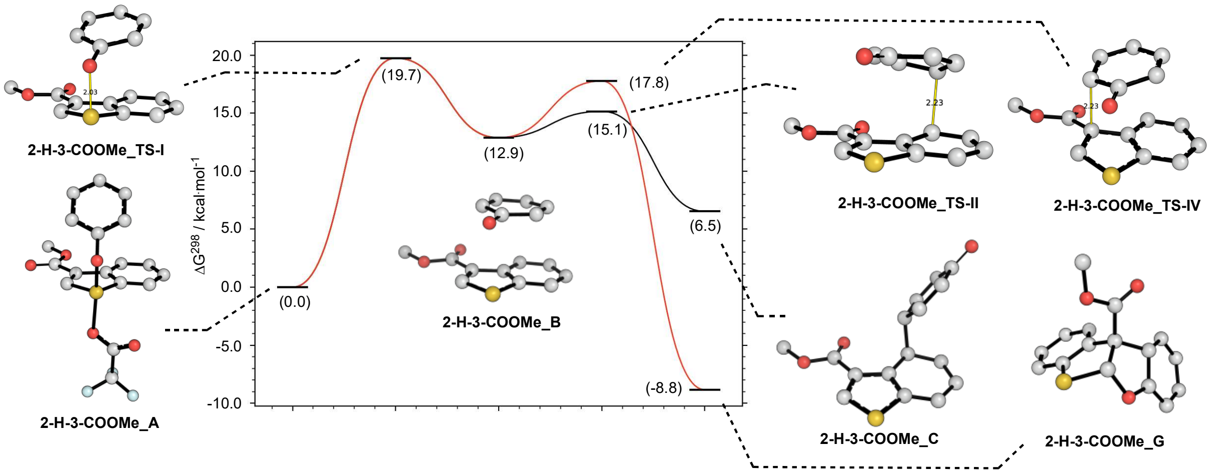

Figure S3: Potential energy surfaces for the reaction between methyl benzo[*b*]thiophene-3-carboxylate 1-oxide and phenol computed at the M06-2X-D3/Def2-QZVPP(SMD=CH_2Cl_2)//M06-2X-D3/6-31+G(d,p)(SMD=CH_2Cl_2) level of theory

The potential energy surface for the reaction between 2,3-dimethylbenzo[*b*]thiophene 1-oxide and phenol was also considered: experimentally, this reaction leads to the exclusive formation of the C3-arylated product. The intrinsic reaction coordinate (IRC) calculation from the TS for the cleavage of the S-O bond (**2-Me-3-Me_TS-I**) bypasses a π-stacked complex and instead evolves directly to the formation of the C3-arylated intermediate **2-Me-3-Me_G,** which results in the observed regioisomer. Attempts at identifying a transition state leading to the direct arylation at the C4 position lead to the identification of a new TS corresponding instead to a formal concerted asynchronous [3,3]-sigmatropic rearrangement (**2-Me-3-Me_TS-IV**) between the C4 and the C3-arylated intermediates as confirmed by IRC calculations (**Figure S4**).

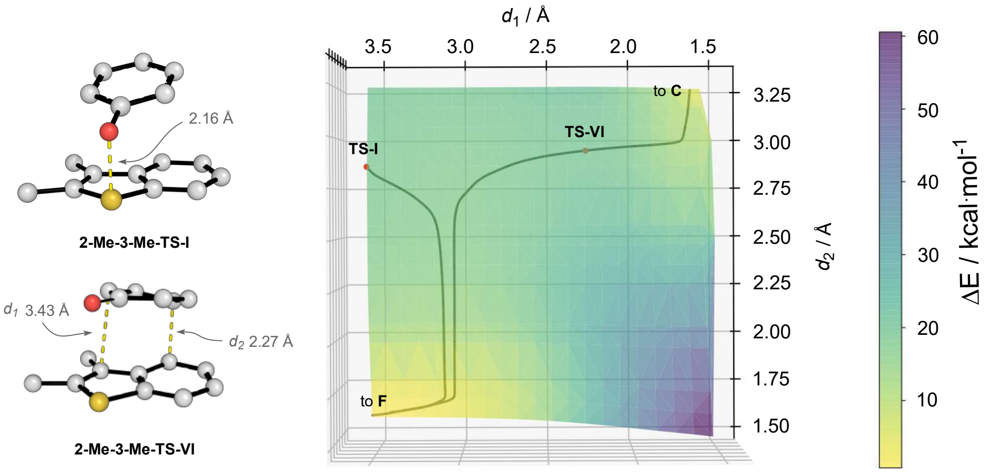

Figure S4: 2D potential energy surfaces for the reaction between 2,3-dimethylbenzo[*b*]thiophene 1-oxide and phenol computed at the M06-2X-D3/6-31+G(d,p)(SMD=CH_2Cl_2) level of theory; IRC paths are indicated by the back lines.

8.3. Fukui functions

Fukui functions were calculated for a series of benzothiophenes using population analysis data computed using NBO.6 interfaced with Gaussian 16 version C.01. Only NBO charges of the heavy atoms were used in the calculation of the Fukui functions, with the partial charges of the hydrogen atoms beings summed into the charges of the heavy atoms that they are directly connected to.

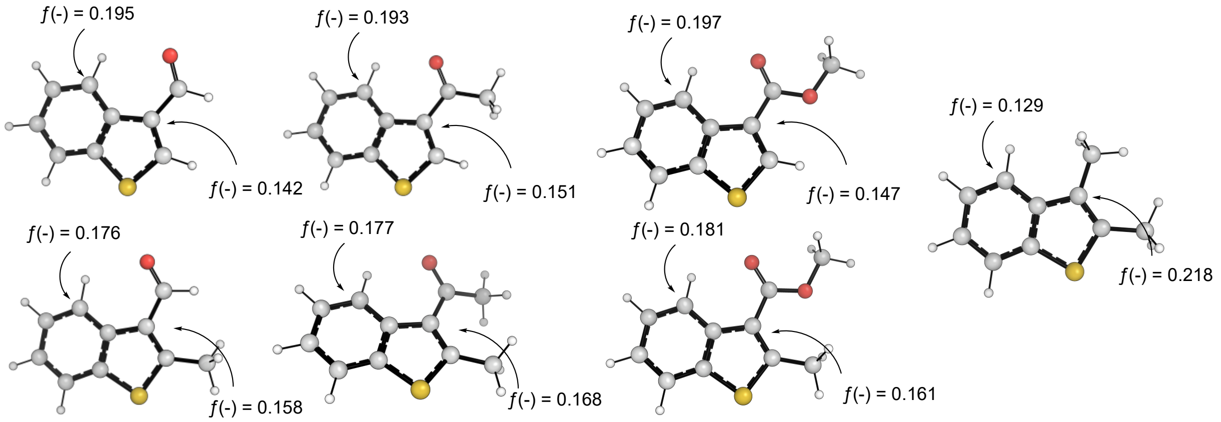

Figure S5: ƒ(-) Fukui functions computed for a series of bezothiophenes computed at the M06-2X-D3/6-31+G(d,p)(SMD=CH_2Cl_2) level of theory;

8.4. Dynamics simulations

Due to the shallow nature of the potential energy surface of the reaction between 2,3-dimethylbenzo[*b*]thiophene 1-oxide and phenol upon crossing of **2-Me-3-Me_TS-I**, we surmised that dynamic effects could potentially play an important role in this transformation. Thus, the *Jprogdyn* package was used to initiate and propagate 100 quasiclassical dynamics trajectories starting in the region of **2-Me-3-Me_TS-I**. Experimentally, optimal reaction conditions involve slow warming from -50°C to room temperature upon addition of phenol, making it challenging for us to assign a single reaction temperature in these simulations, used in allocating the excess thermal energy across the vibrational normal modes. However, it was possible to obtain full conversion of starting material in 14h in the reaction between benzothiophene S-oxide **2a** and 2-bromophenol **3a** when the temperature was maintained constant at -50°C. Therefore, for our simulations the temperature was set to 233 K together with quasiclassical partitioning of energy, 1 fs time steps and a total of 500 fs simulation time. The level of theory chosen was M06-2X-D3/6-31+G(d,p)(SMD=CH_2Cl_2).

**Outcome**	**no. of events**
**C3-arylation**	39
**C4-arylation**	6
**Stable "B"**	50
**TS-I recrossing**	3
**Error Termination**	2

Supplementary Table 2: Tabulated results of dynamic simulations

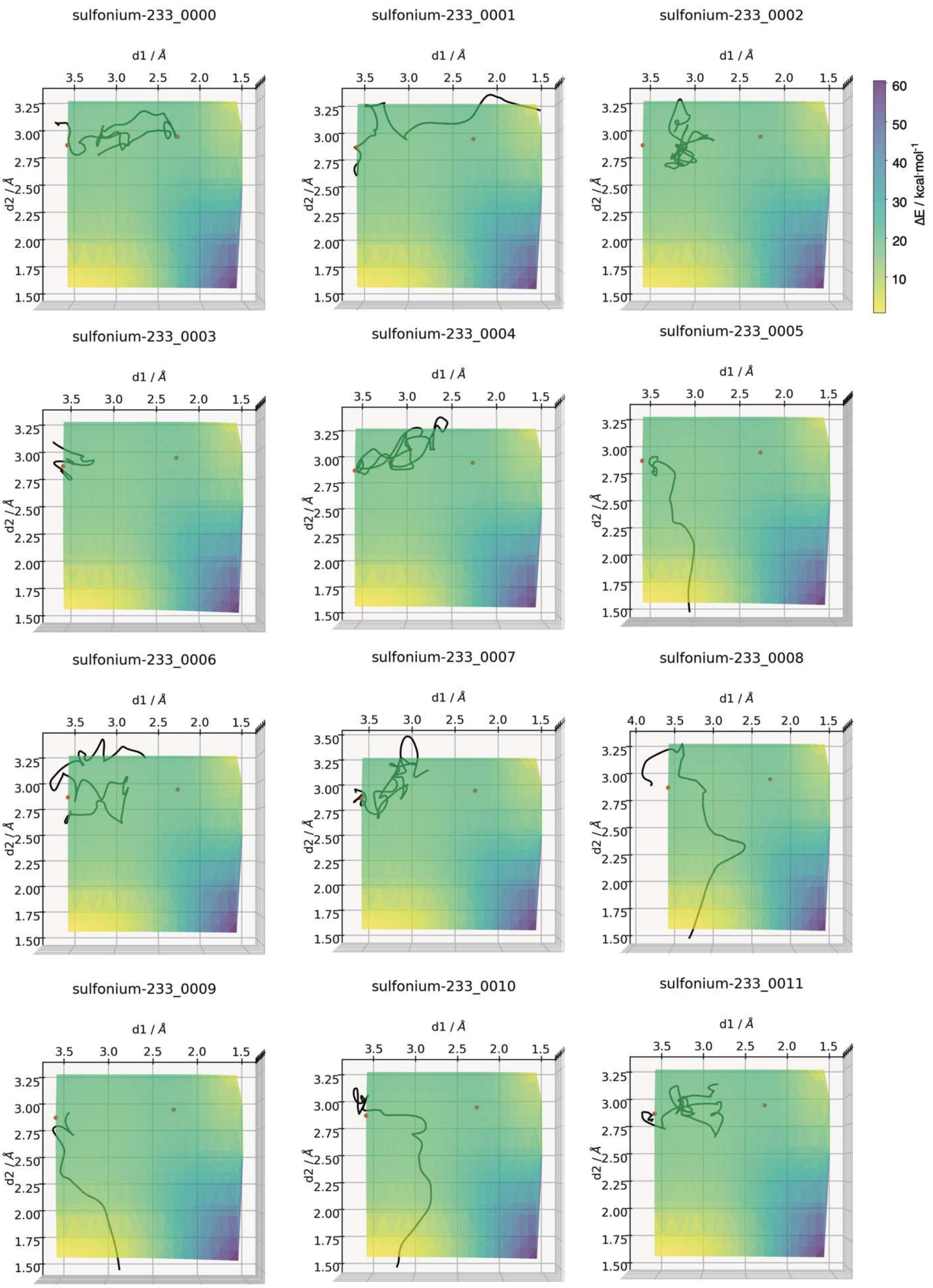
sulfonium-233_0000
sulfonium-233_0001
sulfonium-233_0002
sulfonium-233_0003
sulfonium-233_0004
sulfonium-233_0005
sulfonium-233_0006
sulfonium-233_0007
sulfonium-233_0008
sulfonium-233_0009
sulfonium-233_0010
sulfonium-233_0011
d1 / Å
d2 / Å
ΔE / kcal·mol-1

sulfonium-233_0012

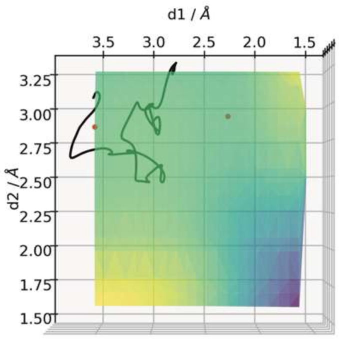

sulfonium-233_0013

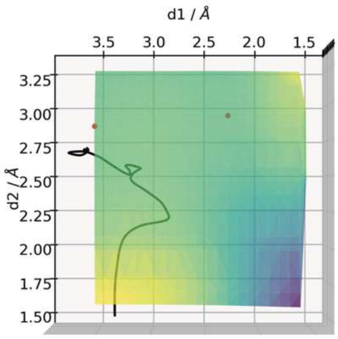

sulfonium-233_0014

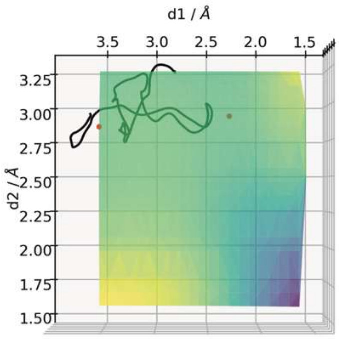

sulfonium-233_0015

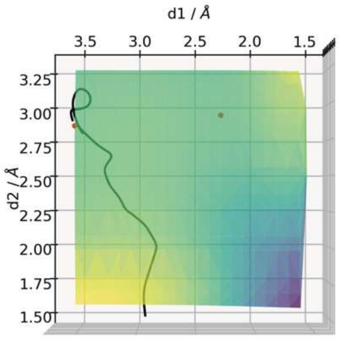

sulfonium-233_0016

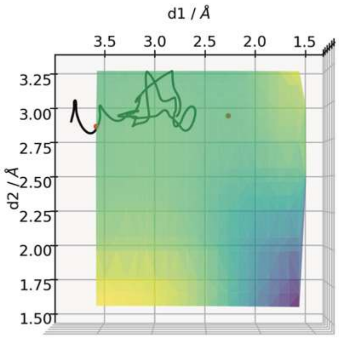

sulfonium-233_0017

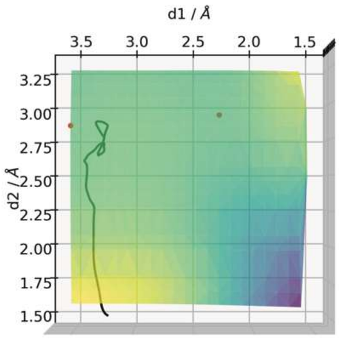

sulfonium-233_0018

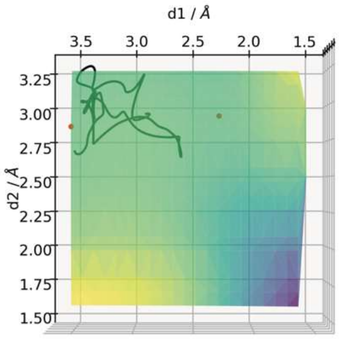

sulfonium-233_0019

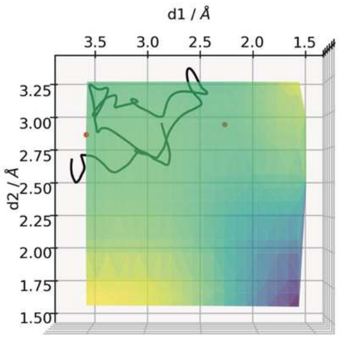

sulfonium-233_0020

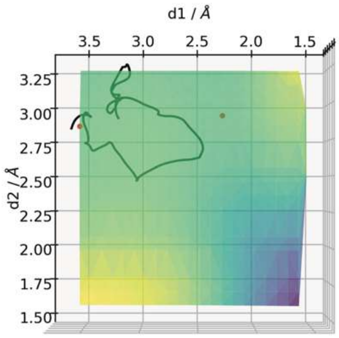

sulfonium-233_0021

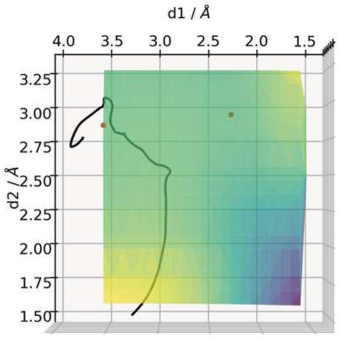

sulfonium-233_0022

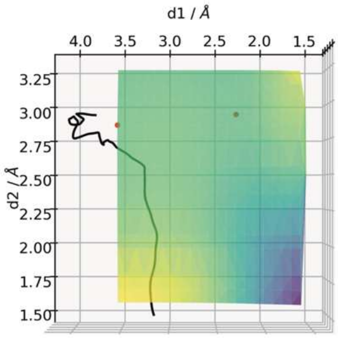

sulfonium-233_0023

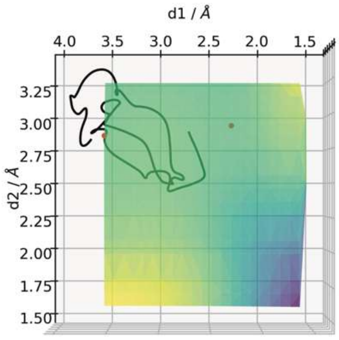

sulfonium-233_0024

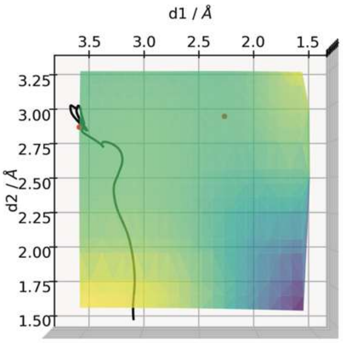

sulfonium-233_0025

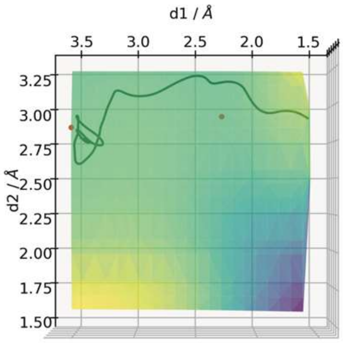

sulfonium-233_0026

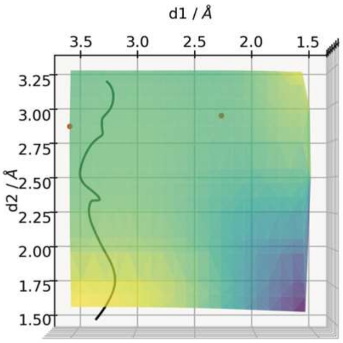

sulfonium-233_0027

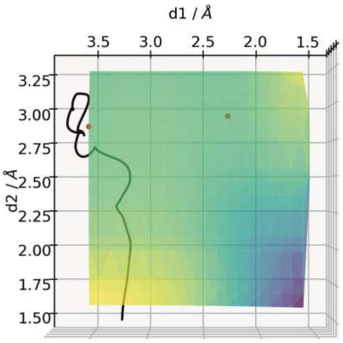

sulfonium-233_0028

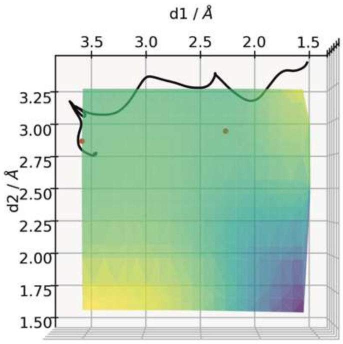

sulfonium-233_0029

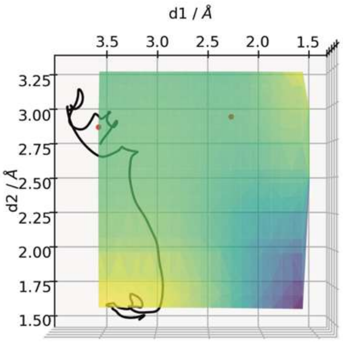

sulfonium-233_0030

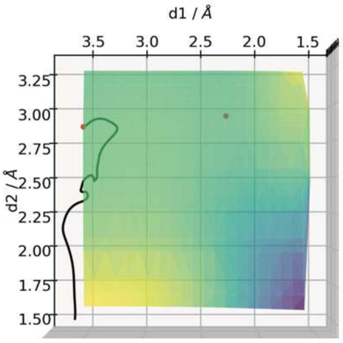

sulfonium-233_0031

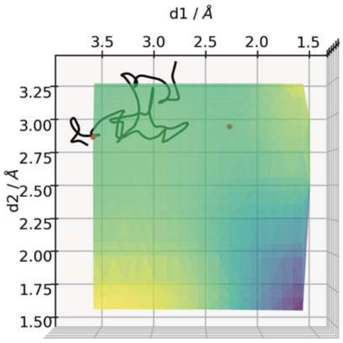

sulfonium-233_0032

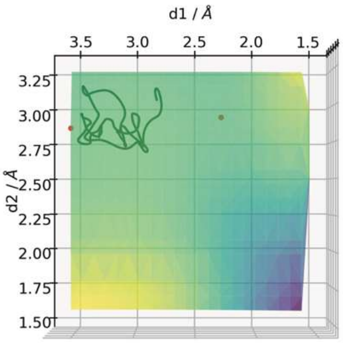

sulfonium-233_0033

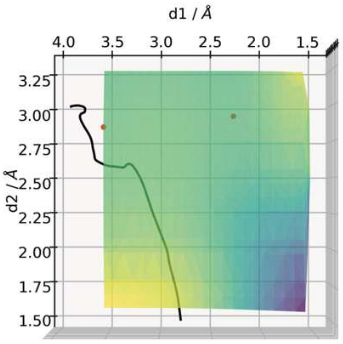

sulfonium-233_0034

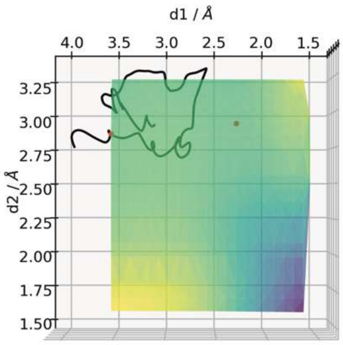

sulfonium-233_0035

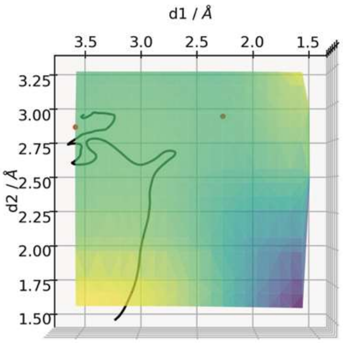

sulfonium-233_0036

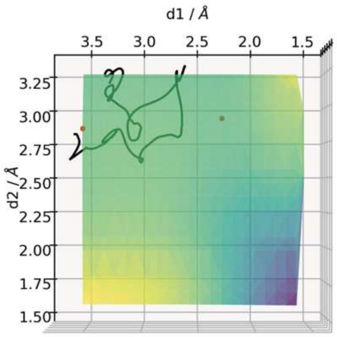

sulfonium-233_0037

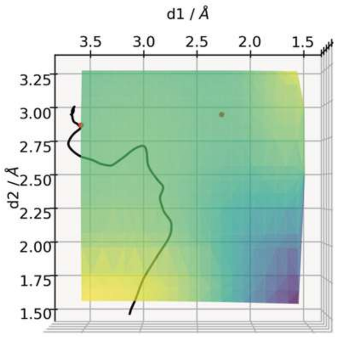

sulfonium-233_0038

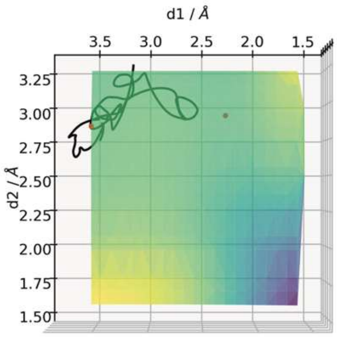

sulfonium-233_0039

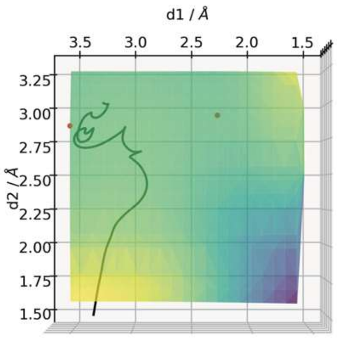

sulfonium-233_0040

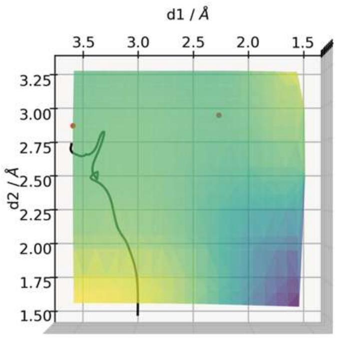

sulfonium-233_0041

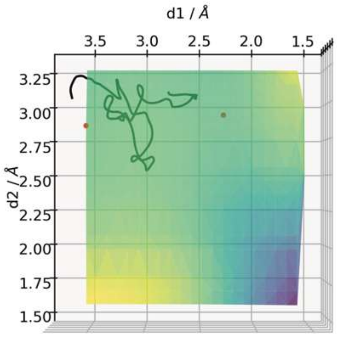

sulfonium-233_0042

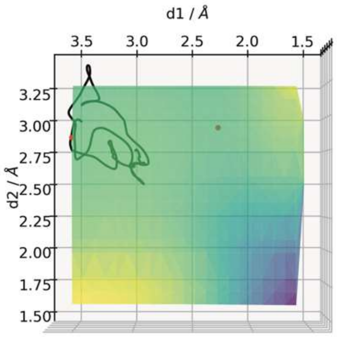

sulfonium-233_0043

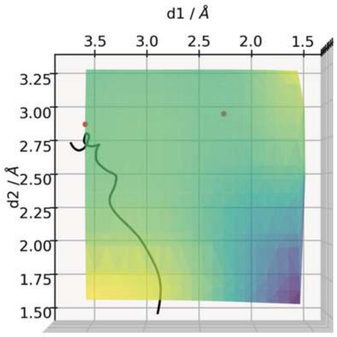

sulfonium-233_0044

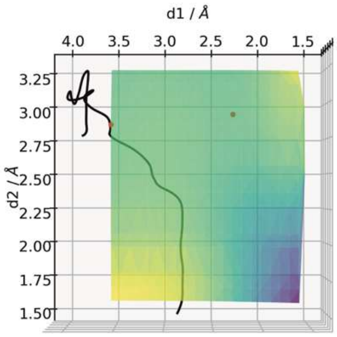

sulfonium-233_0045

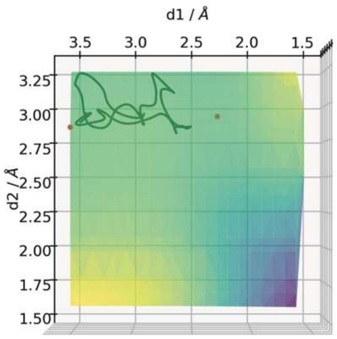

sulfonium-233_0046

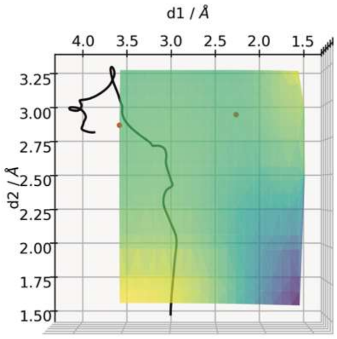

sulfonium-233_0047

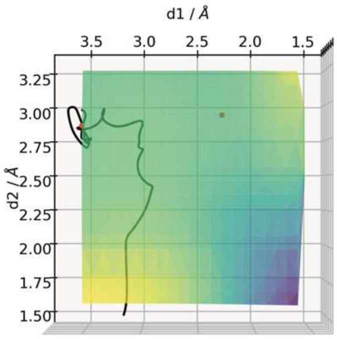

sulfonium-233_0048

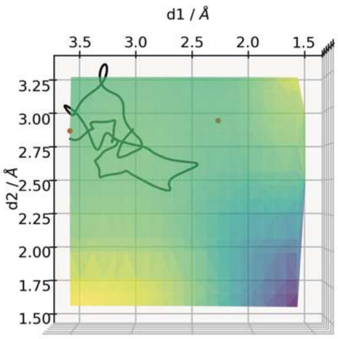

sulfonium-233_0049

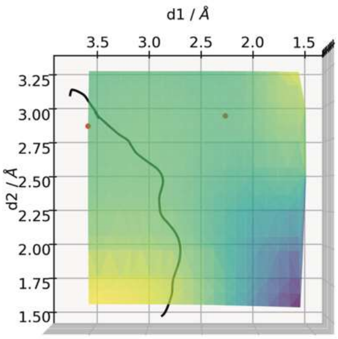

sulfonium-233_0050

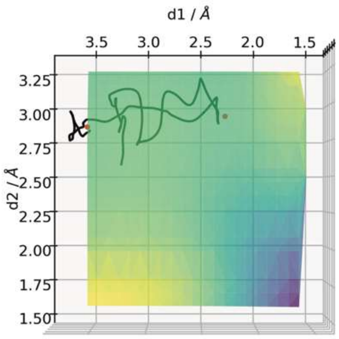

sulfonium-233_0051

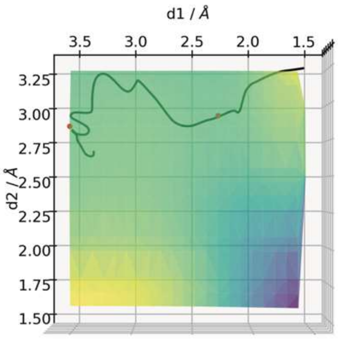

sulfonium-233_0052

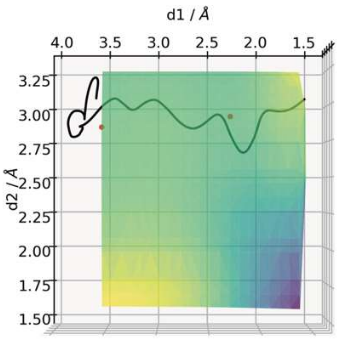

sulfonium-233_0053

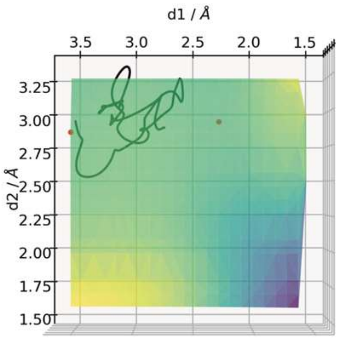

sulfonium-233_0054

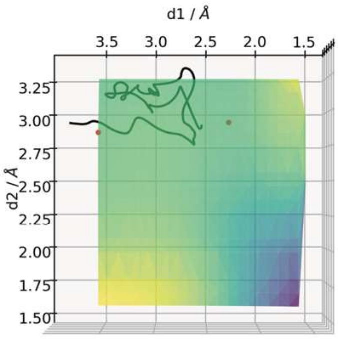

sulfonium-233_0055

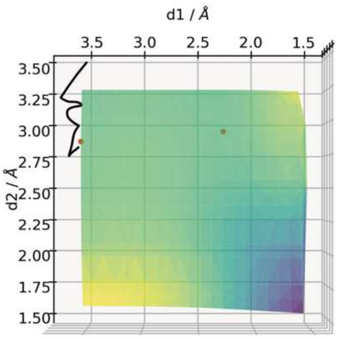

sulfonium-233_0056

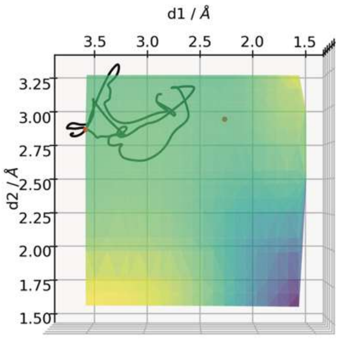

sulfonium-233_0057

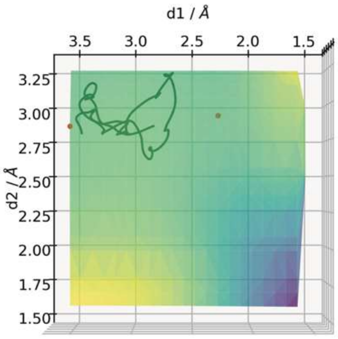

sulfonium-233_0058

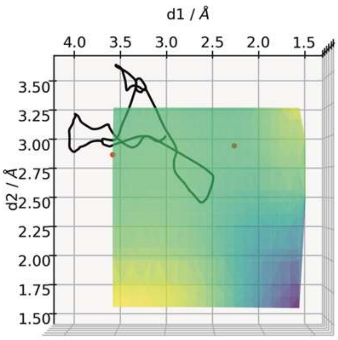

sulfonium-233_0059

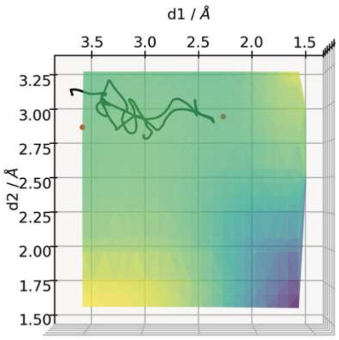

sulfonium-233_0061

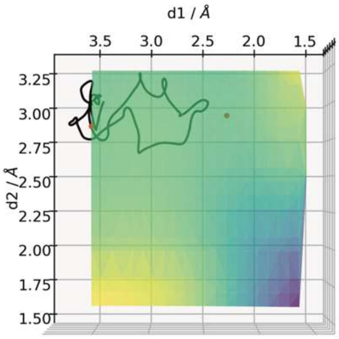

sulfonium-233_0062

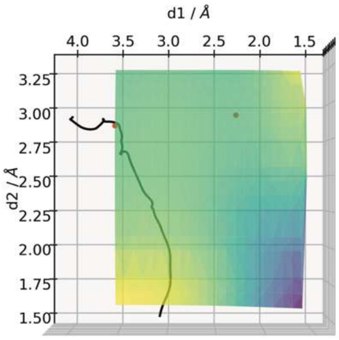

sulfonium-233_0063

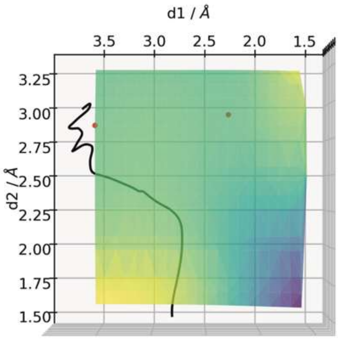

sulfonium-233_0064

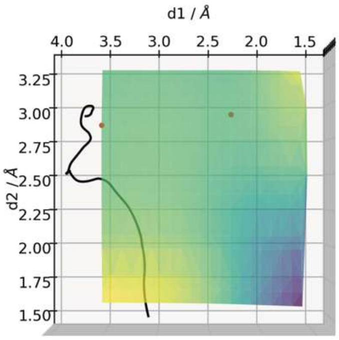

sulfonium-233_0065

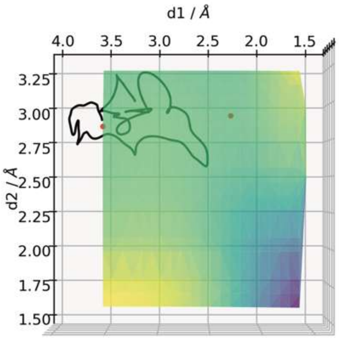

sulfonium-233_0066

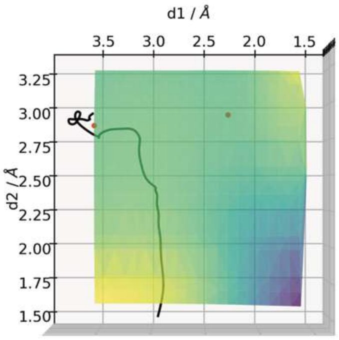

sulfonium-233_0067

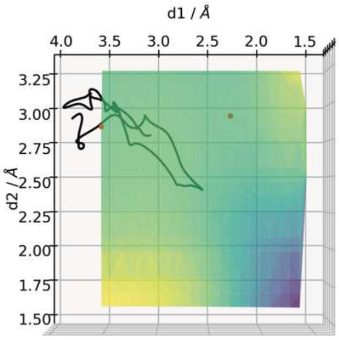

sulfonium-233_0068

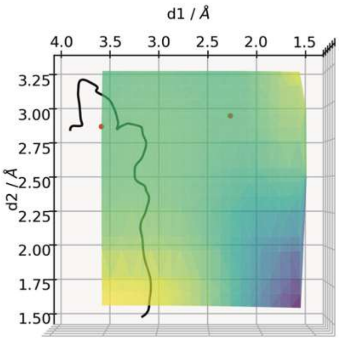

sulfonium-233_0069

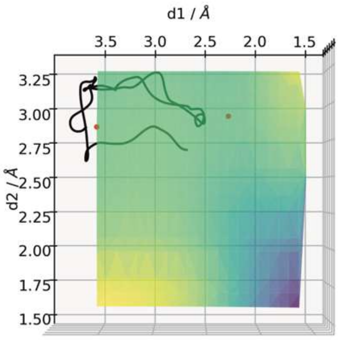

sulfonium-233_0070

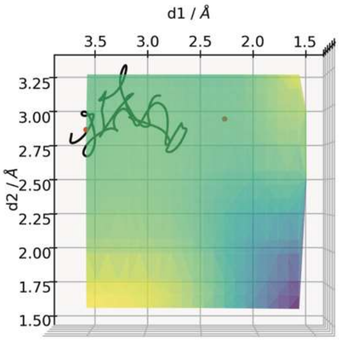

sulfonium-233_0071

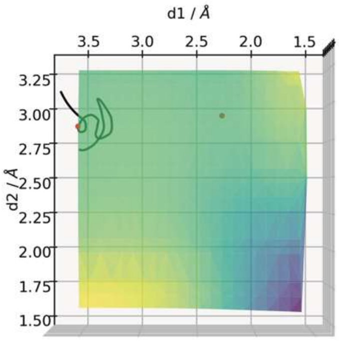

sulfonium-233_0072

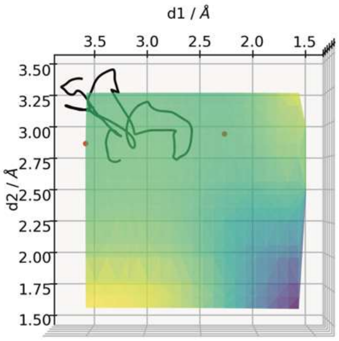

sulfonium-233_0073

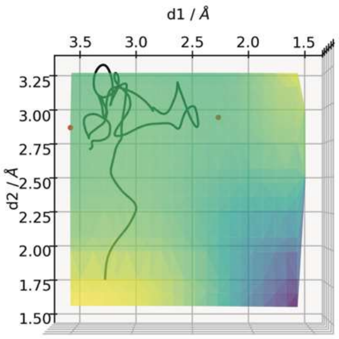

sulfonium-233_0074

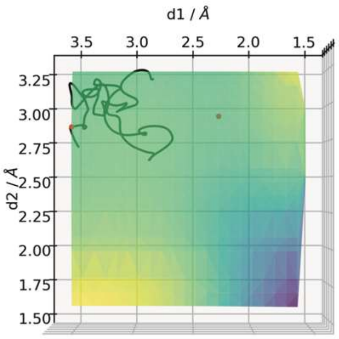

sulfonium-233_0075

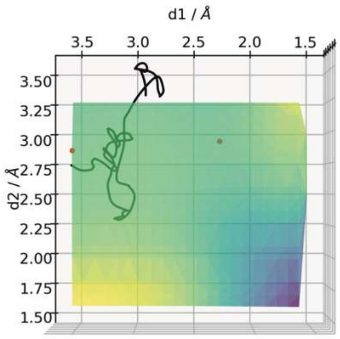

sulfonium-233_0076

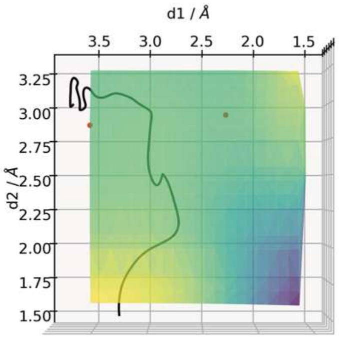

sulfonium-233_0077

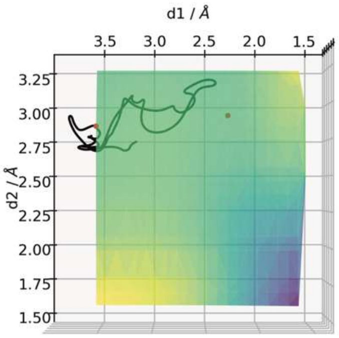

sulfonium-233_0078

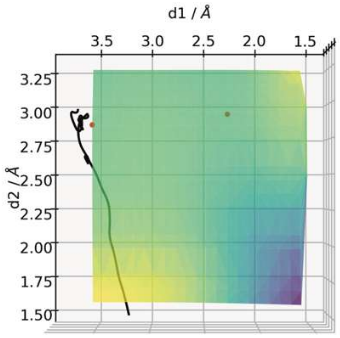

sulfonium-233_0079

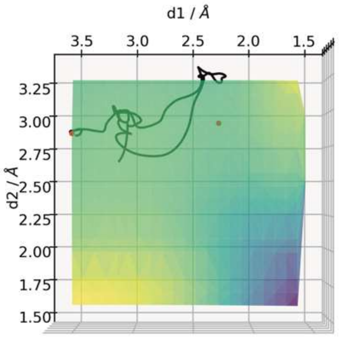

sulfonium-233_0080

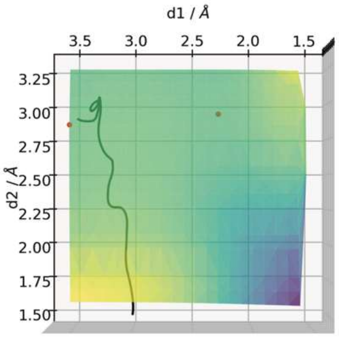

sulfonium-233_0081

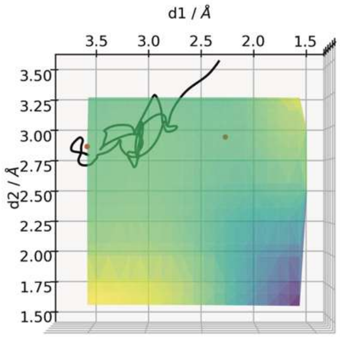

sulfonium-233_0082

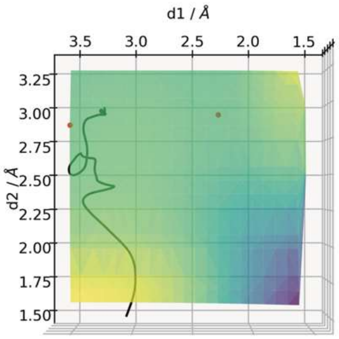

sulfonium-233_0083

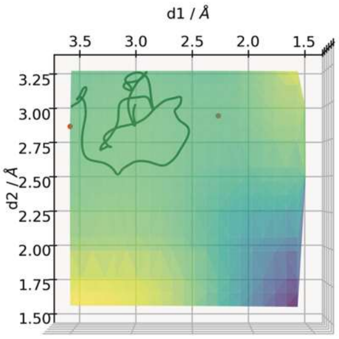

sulfonium-233_0084

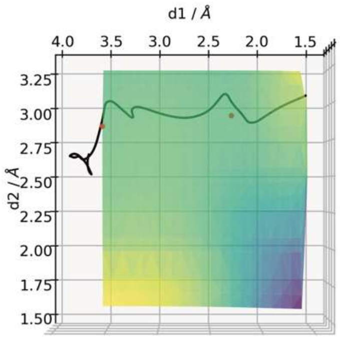

sulfonium-233_0085

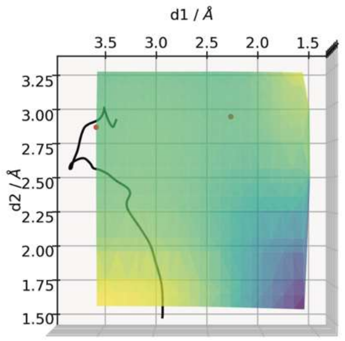

sulfonium-233_0086

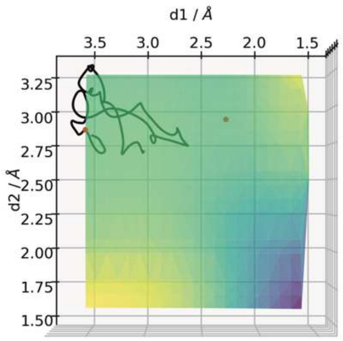

sulfonium-233_0087

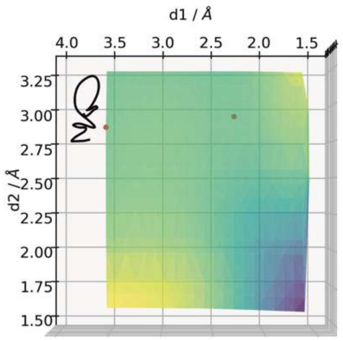

sulfonium-233_0088

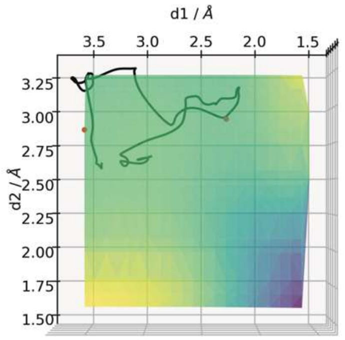

sulfonium-233_0089

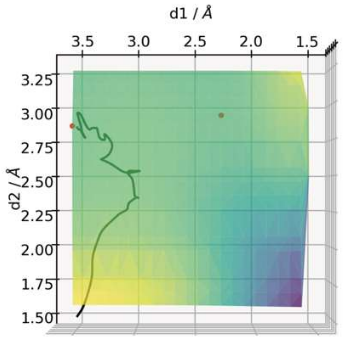

sulfonium-233_0090

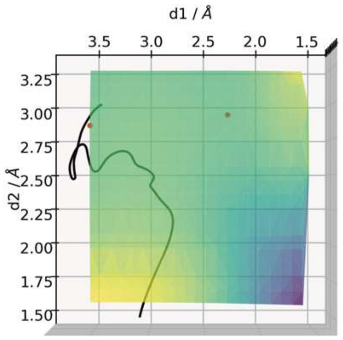

sulfonium-233_0091

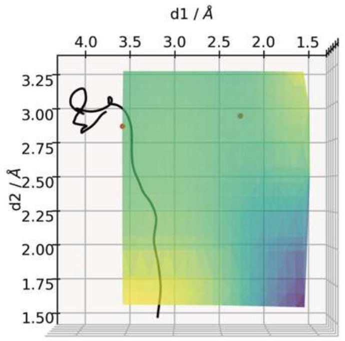

sulfonium-233_0092

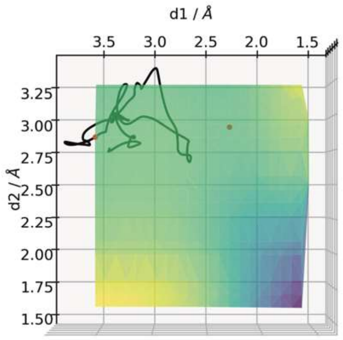

sulfonium-233_0093

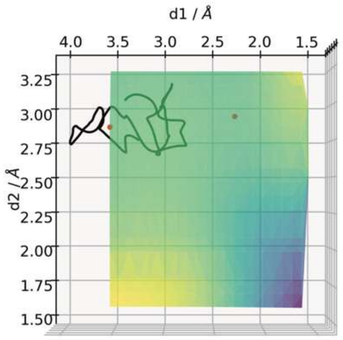

sulfonium-233_0094

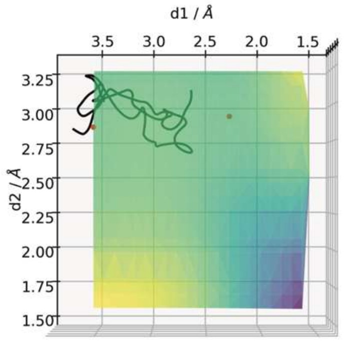

sulfonium-233_0095

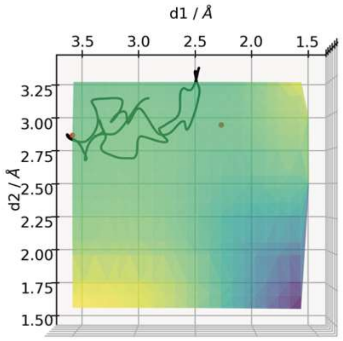

sulfonium-233_0096

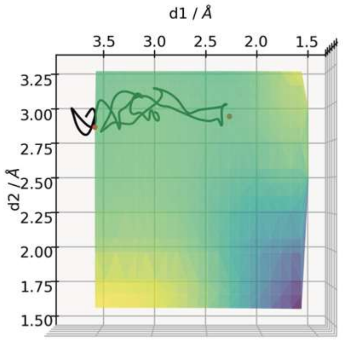

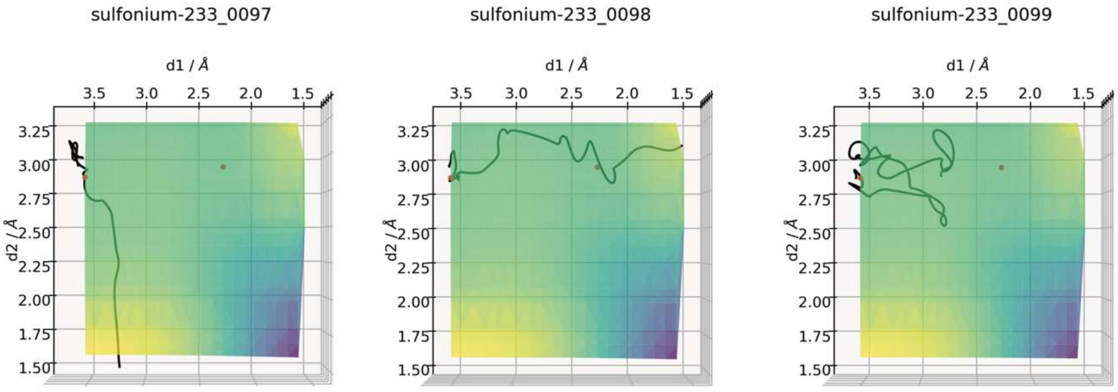

Figure S6: Dynamic trajectories for the reaction between 2,3-dimethylbenzo[*b*]thiophene 1-oxide and phenol

8.5. Thermochemical Data

Legend:
E_T = adiabatic triplet energy
E_{SPC} = energy obtained in the single-point energy corrections
E = energy obtained in the geometry optimizations
ZPE = zero-point energy
H_{SPC} = enthalpy corrected with E_{SPC}
T·S = temperature times entropy with no correction
T·qh-S = temperature times entropy with quasi-harmonic S correction
G(T) = Gibbs free energy corrected only with E_{SPC}
qh-G(T) = Gibbs free energy with E_{SPC} and quasi-harmonic S correction
ν_{imag} = imaginary frequencies

Name	E_{SPC}	E	ZPE	H_{SPC}	T·S	T·gh-S	G(T)	qh-G(T)	ν_{imag} / cm^{-1}
**2-H-3-COOMe-A1-1**	-1767.710396	-1767.053476	0.273715	-1767.410441	0.082245	0.075747	-1767.492686	-1767.486188	
**2-H-3-COOMe-A1-2**	-1767.709118	-1767.052438	0.273613	-1767.409205	0.083373	0.076314	-1767.492578	-1767.485520	
**2-H-3-COOMe-A1-3**	-1767.709061	-1767.052411	0.273522	-1767.409174	0.084887	0.077042	-1767.494061	-1767.486216	
**2-H-3-COOMe-A1-4**	-1767.707254	-1767.050306	0.273550	-1767.407391	0.082568	0.075850	-1767.489958	-1767.483241	
**2-H-3-COOMe-A1-5**	-1767.706204	-1767.049299	0.273653	-1767.406190	0.084738	0.076976	-1767.490928	-1767.483166	
**2-H-3-COOMe-A1-6**	-1767.707577	-1767.050448	0.273642	-1767.407579	0.083946	0.076596	-1767.491525	-1767.484174	
**2-H-3-COOMe-A2-1**	-1241.238319	-1240.828084	0.246691	-1240.973116	0.062847	0.059451	-1241.035964	-1241.032568	
**2-H-3-COOMe-A2-2**	-1241.237277	-1240.827222	0.246823	-1240.972067	0.062256	0.059079	-1241.034322	-1241.031145	
**2-H-3-COOMe-A2-3**	-1241.237276	-1240.827096	0.246745	-1240.972104	0.061933	0.059019	-1241.034037	-1241.031123	
**2-H-3-COOMe-A2-5**	-1241.232109	-1240.821840	0.246249	-1240.967147	0.064090	0.060299	-1241.031237	-1241.027446	
**2-H-3-COOMe-A2-6**	-1241.233259	-1240.822800	0.246127	-1240.968371	0.064347	0.060452	-1241.032718	-1241.028823	
**2-H-3-COOMe-B-1**	-1241.228501	-1240.830249	0.244994	-1240.964325	0.062306	0.060227	-1241.026631	-1241.024552	
**2-H-3-COOMe-B-2**	-1241.230254	-1240.831675	0.245211	-1240.965926	0.061909	0.060011	-1241.027835	-1241.025937	
**2-H-3-COOMe-C-1**	-1241.239913	-1240.841566	0.246474	-1240.974878	0.062189	0.059258	-1241.037067	-1241.034137	
**2-H-3-COOMe-C-2**	-1241.240766	-1240.842201	0.246595	-1240.975673	0.061376	0.058938	-1241.037049	-1241.034611	
**2-H-3-COOMe-C-3**	-1241.242254	-1240.843296	0.247170	-1240.976665	0.061365	0.059040	-1241.038030	-1241.035705	
**2-H-3-COOMe-C-4**	-1241.240410	-1240.841805	0.246744	-1240.975090	0.062082	0.059372	-1241.037171	-1241.034462	
**2-H-3-COOMe-C-5**	-1241.235378	-1240.836676	0.246723	-1240.970054	0.062390	0.059378	-1241.032444	-1241.029432	
**2-H-3-COOMe-C-6**	-1241.236120	-1240.837606	0.246659	-1240.971076	0.061360	0.058818	-1241.032436	-1241.029894	
**2-H-3-COOMe-E-1**	-1241.267360	-1240.872439	0.249140	-1241.000793	0.058574	0.056612	-1241.059366	-1241.057405	
**2-H-3-COOMe-E-2**	-1241.269767	-1240.850420	0.248812	-1241.003252	0.060004	0.057456	-1241.063256	-1241.060707	

**2-H-3-COOMe-TS1-1**	-1241.220646	-1240.818562	0.245608	-1240.956973	0.060416	0.058109	-1241.017389	-1241.015082	-276.55
**2-H-3-COOMe-TS1-2**	-1241.219440	-1240.817557	0.245805	-1240.955752	0.059179	0.057523	-1241.014931	-1241.013274	-273.22
**2-H-3-COOMe-TS2-1**	-1241.225706	-1240.827370	0.245351	-1240.962063	0.060263	0.058286	-1241.022327	-1241.020349	-151.66
**2-H-3-COOMe-TS2-2**	-1241.227638	-1240.828971	0.245328	-1240.963999	0.060349	0.058419	-1241.024348	-1241.022418	-144.51
**2-H-3-COOMe-TS3-1**	-1241.222858	-1240.824367	0.245518	-1240.959221	0.059275	0.057952	-1241.018496	-1241.017173	-203.90
**2-H-3-COOMe-TS3-2**	-1241.223569	-1240.824963	0.245451	-1240.959940	0.059593	0.058086	-1241.019533	-1241.018026	-212.95
**2-Me-3-Me-A1-1**	-1618.446946	-1617.861156	0.285406	-1618.136307	0.079430	0.073560	-1618.215737	-1618.209867	
**2-Me-3-Me-A1-2**	-1618.449017	-1617.863161	0.285071	-1618.138622	0.080096	0.073785	-1618.218718	-1618.212407	
**2-Me-3-Me-A1-3**	-1618.450844	-1617.865301	0.285724	-1618.140144	0.078148	0.072770	-1618.218292	-1618.212914	
**2-Me-3-Me-A2-1**	-1091.982891	-1091.644055	0.258636	-1091.707033	0.057896	0.055948	-1091.764929	-1091.762981	
**2-Me-3-Me-A2-2**	-1091.978335	-1091.638730	0.257822	-1091.702803	0.060395	0.057620	-1091.763198	-1091.760423	
**2-Me-3-Me-C-1**	-1091.971841	-1091.643512	0.258569	-1091.695800	0.059035	0.056675	-1091.754835	-1091.752475	
**2-Me-3-Me-C2**	-1091.976685	-1091.648785	0.258962	-1091.700591	0.057589	0.055737	-1091.758179	-1091.756328	
**2-Me-3-Me-C3**	-1091.978362	-1091.650317	0.258270	-1091.702520	0.059377	0.056980	-1091.761897	-1091.759500	
**2-Me-3-Me-D-1**	-1091.989264	-1091.662337	0.259355	-1091.713100	0.056392	0.054840	-1091.769491	-1091.767940	
**2-Me-3-Me-E-1**	-1092.007058	-1091.684157	0.261226	-1091.730022	0.053499	0.052510	-1091.783521	-1091.782533	
**2-Me-3-Me-TS1-1**	-1091.960101	-1091.630303	0.257266	-1091.685987	0.055917	0.054948	-1091.741903	-1091.740935	-212.44
**2-Me-3-Me-TS2-1**	-1091.958117	-1091.630551	0.257055	-1091.683874	0.056940	0.055659	-1091.740814	-1091.739533	-168.94
**A_1**	-1731.785890	-1731.150197	0.294976	-1731.463985	0.083015	0.076809	-1731.547000	-1731.540795	
**A_10**	-1731.766355	-1731.129198	0.294183	-1731.444623	0.087008	0.078962	-1731.531631	-1731.523585	
**A_11**	-1731.769357	-1731.132547	0.294417	-1731.447646	0.085727	0.078129	-1731.533373	-1731.525775	
**A_12**	-1731.771449	-1731.134316	0.294415	-1731.449823	0.083428	0.077213	-1731.533250	-1731.527036	
**A_13**	-1731.773345	-1731.136463	0.294574	-1731.451688	0.083331	0.077048	-1731.535019	-1731.528736	
**A_2**	-1731.785451	-1731.149531	0.294736	-1731.463615	0.083392	0.077187	-1731.547008	-1731.540802	
**A_3**	-1731.785698	-1731.149749	0.294883	-1731.463692	0.083972	0.077475	-1731.547664	-1731.541167	
**A_4**	-1731.784090	-1731.148433	0.295088	-1731.462073	0.082896	0.076691	-1731.544969	-1731.538764	
**A_5**	-1731.782267	-1731.145918	0.294617	-1731.460325	0.086400	0.078711	-1731.546726	-1731.539037	
**A_6**	-1731.782124	-1731.145736	0.294728	-1731.460237	0.084531	0.077753	-1731.544768	-1731.537990	
**A_7**	-1731.781861	-1731.145856	0.294524	-1731.460090	0.084540	0.077765	-1731.544630	-1731.537855	
**A_8**	-1731.778136	-1731.141808	0.294837	-1731.456173	0.084877	0.077875	-1731.541049	-1731.534048	
**A_9**	-1731.771593	-1731.134605	0.294855	-1731.449540	0.084911	0.077670	-1731.534451	-1731.527210	
**B_1-notfa**	-1205.301531	-1204.924037	0.266783	-1205.015053	0.062874	0.060783	-1205.077928	-1205.075836	
**B_1**	-1731.754614	-1731.128826	0.292979	-1731.433723	0.084873	0.078397	-1731.518596	-1731.512121	
**B_10**	-1731.759387	-1731.134217	0.293211	-1731.438395	0.085371	0.078739	-1731.523766	-1731.517134	
**B_11**	-1731.759259	-1731.134207	0.293323	-1731.438218	0.082852	0.077742	-1731.521070	-1731.515960	
**B_12**	-1731.759012	-1731.133374	0.293655	-1731.437596	0.084531	0.078299	-1731.522127	-1731.515895	

**B_13**	-1731.756121	-1731.130323	0.292844	-1731.435242	0.086088	0.079131	-1731.521329	-1731.514373
**B_14**	-1731.758610	-1731.133632	0.293814	-1731.437406	0.082267	0.076915	-1731.519672	-1731.514320
**B_15**	-1731.759510	-1731.133968	0.293989	-1731.437967	0.083353	0.077616	-1731.521321	-1731.515583
**B_16**	-1731.761301	-1731.136222	0.293743	-1731.440049	0.083869	0.077943	-1731.523918	-1731.517992
**B_17**	-1731.760317	-1731.134825	0.293324	-1731.439144	0.084377	0.078329	-1731.523521	-1731.517473
**B_18**	-1731.759795	-1731.134010	0.293654	-1731.438302	0.086478	0.079166	-1731.524780	-1731.517468
**B_19**	-1731.754017	-1731.128412	0.293633	-1731.432794	0.084029	0.077814	-1731.516824	-1731.510608
**B_2-notfa**	-1205.299952	-1204.922399	0.266527	-1205.013627	0.063108	0.060986	-1205.076735	-1205.074612
**B_2**	-1731.755618	-1731.130348	0.293351	-1731.434669	0.082729	0.077468	-1731.517398	-1731.512136
**B_20**	-1731.759873	-1731.134532	0.293702	-1731.438629	0.082446	0.077356	-1731.521075	-1731.515985
**B_21**	-1731.763763	-1731.138487	0.293325	-1731.442685	0.083837	0.078106	-1731.526523	-1731.520791
**B_22**	-1731.755689	-1731.129890	0.293295	-1731.434597	0.085028	0.078286	-1731.519626	-1731.512883
**B_3-notfa**	-1205.294909	-1204.917340	0.266158	-1205.008732	0.063882	0.061613	-1205.072614	-1205.070345
**B_3**	-1731.757688	-1731.132713	0.293239	-1731.436819	0.083213	0.077611	-1731.520031	-1731.514429
**B_4**	-1731.758471	-1731.133245	0.293344	-1731.437467	0.083116	0.077715	-1731.520583	-1731.515182
**B_5**	-1731.760120	-1731.134729	0.293244	-1731.439128	0.083585	0.077935	-1731.522713	-1731.517063
**B_6**	-1731.758865	-1731.133435	0.293010	-1731.437935	0.084364	0.078445	-1731.522299	-1731.516381
**B_7**	-1731.760315	-1731.134801	0.293488	-1731.439047	0.084558	0.078304	-1731.523605	-1731.517351
**B_8**	-1731.759333	-1731.134222	0.293319	-1731.438045	0.085411	0.078772	-1731.523456	-1731.516817
**B_9**	-1731.758119	-1731.133196	0.293623	-1731.437113	0.081388	0.076864	-1731.518501	-1731.513977
**C_1-notfa**	-1205.312201	-1204.934332	0.267964	-1205.025056	0.062431	0.060071	-1205.087487	-1205.085127
**C_1**	-1731.776066	-1731.150343	0.295077	-1731.453993	0.082355	0.076550	-1731.536348	-1731.530543
**C_10**	-1731.774549	-1731.149386	0.294868	-1731.452634	0.084236	0.077366	-1731.536870	-1731.530001
**C_11**	-1731.780414	-1731.155322	0.295324	-1731.458244	0.082111	0.076312	-1731.540355	-1731.534556
**C_12**	-1731.778465	-1731.152983	0.294860	-1731.456495	0.082683	0.076684	-1731.539178	-1731.533179
**C_13**	-1731.777837	-1731.152535	0.294956	-1731.455845	0.082807	0.076668	-1731.538652	-1731.532513
**C_14**	-1731.774354	-1731.148807	0.295048	-1731.452206	0.083649	0.077111	-1731.535855	-1731.529316
**C_15**	-1731.777092	-1731.152214	0.295188	-1731.455099	0.081374	0.075866	-1731.536472	-1731.530965
**C_16**	-1731.771578	-1731.146198	0.295681	-1731.449148	0.082452	0.076222	-1731.531600	-1731.525369
**C_17**	-1731.780007	-1731.155071	0.295142	-1731.458006	0.081673	0.076101	-1731.539679	-1731.534107
**C_18**	-1731.774751	-1731.149586	0.295831	-1731.452380	0.080708	0.075400	-1731.533088	-1731.527780
**C_19**	-1731.780010	-1731.154817	0.294929	-1731.458178	0.082546	0.076492	-1731.540724	-1731.534670
**C_2-notfa**	-1205.316082	-1204.938502	0.267723	-1205.029169	0.062331	0.059914	-1205.091500	-1205.089083
**C_2**	-1731.777842	-1731.152482	0.295049	-1731.455814	0.083074	0.076691	-1731.538887	-1731.532505
**C_20**	-1731.776772	-1731.151886	0.294884	-1731.454735	0.083167	0.077035	-1731.537901	-1731.531770
**C_21**	-1731.776409	-1731.151563	0.295235	-1731.454365	0.081363	0.075834	-1731.535728	-1731.530199

**C_22**	-1731.776620	-1731.151421	0.294982	-1731.454475	0.083684	0.077196	-1731.538159	-1731.531671
**C_23**	-1731.775450	-1731.150207	0.295493	-1731.453210	0.082258	0.076143	-1731.535468	-1731.529353
**C_24**	-1731.776525	-1731.151169	0.295247	-1731.454277	0.082459	0.076503	-1731.536736	-1731.530780
**C_25**	-1731.773992	-1731.148927	0.295132	-1731.451906	0.083020	0.076513	-1731.534926	-1731.528419
**C_26**	-1731.774676	-1731.149413	0.295157	-1731.452603	0.082962	0.076706	-1731.535565	-1731.529310
**C_27**	-1731.776314	-1731.150834	0.294647	-1731.454495	0.084118	0.077314	-1731.538613	-1731.531809
**C_28**	-1731.778149	-1731.152037	0.295058	-1731.456081	0.082830	0.076634	-1731.538912	-1731.532716
**C_29**	-1731.774898	-1731.149723	0.294985	-1731.452765	0.083893	0.077225	-1731.536658	-1731.529990
**C_3-notfa**	-1205.316005	-1204.937983	0.268253	-1205.028699	0.062759	0.059908	-1205.091458	-1205.088607
**C_3**	-1731.773447	-1731.148046	0.295565	-1731.451094	0.081169	0.075827	-1731.532263	-1731.526922
**C_30**	-1731.770365	-1731.145357	0.295087	-1731.448393	0.081657	0.076055	-1731.530050	-1731.524448
**C_31**	-1731.777001	-1731.152147	0.295186	-1731.455019	0.080753	0.075707	-1731.535772	-1731.530726
**C_32**	-1731.773066	-1731.148039	0.295247	-1731.450866	0.081640	0.076157	-1731.532506	-1731.527024
**C_33**	-1731.774347	-1731.149898	0.294780	-1731.452635	0.081337	0.075964	-1731.533972	-1731.528599
**C_34**	-1731.774995	-1731.149732	0.294889	-1731.453125	0.082496	0.076421	-1731.535621	-1731.529546
**C_35**	-1731.776462	-1731.151150	0.295049	-1731.454322	0.082993	0.076812	-1731.537315	-1731.531134
**C_36**	-1731.772514	-1731.147113	0.295314	-1731.450348	0.081155	0.076009	-1731.531503	-1731.526357
**C_37**	-1731.774327	-1731.149182	0.294921	-1731.452488	0.081858	0.076315	-1731.534346	-1731.528804
**C_38**	-1731.774309	-1731.149176	0.294921	-1731.452497	0.081641	0.076217	-1731.534138	-1731.528714
**C_39**	-1731.779472	-1731.154407	0.295061	-1731.457470	0.082304	0.076476	-1731.539775	-1731.533947
**C_4-notfa**	-1205.312896	-1204.934634	0.267348	-1205.026019	0.064326	0.061193	-1205.090346	-1205.087212
**C_4**	-1731.779659	-1731.154695	0.295400	-1731.457451	0.082312	0.076283	-1731.539762	-1731.533734
**C_40**	-1731.774241	-1731.148654	0.294800	-1731.452305	0.083290	0.077128	-1731.535595	-1731.529433
**C_41**	-1731.772270	-1731.146595	0.294827	-1731.450186	0.085100	0.077932	-1731.535287	-1731.528119
**C_42**	-1731.775400	-1731.149902	0.294971	-1731.453351	0.084034	0.077366	-1731.537385	-1731.530717
**C_5-notfa**	-1205.309768	-1204.932004	0.267902	-1205.022780	0.062255	0.059782	-1205.085036	-1205.082562
**C_5**	-1731.778161	-1731.152377	0.294736	-1731.456252	0.083244	0.077056	-1731.539496	-1731.533308
**C_6-notfa**	-1205.308690	-1204.930524	0.269026	-1205.020899	0.060876	0.058959	-1205.081775	-1205.079858
**C_6**	-1731.779154	-1731.153912	0.294982	-1731.457219	0.082699	0.076613	-1731.539918	-1731.533831
**C_7**	-1731.778300	-1731.153092	0.294979	-1731.456368	0.081830	0.076253	-1731.538198	-1731.532621
**C_8**	-1731.778356	-1731.152793	0.295154	-1731.456122	0.083004	0.076772	-1731.539126	-1731.532894
**C_9**	-1731.778680	-1731.153150	0.294961	-1731.456741	0.083285	0.076823	-1731.540026	-1731.533565
**D_1-notfa**	-1204.932031	-1204.554484	0.256492	-1204.656199	0.063972	0.061039	-1204.720172	-1204.717239
**D_1**	-1731.844999	-1731.217623	0.295302	-1731.522611	0.085392	0.078330	-1731.608003	-1731.600941
**D_2-notfa**	-1204.931873	-1204.554485	0.256782	-1204.655872	0.063498	0.060620	-1204.719370	-1204.716492
**D_2**	-1731.844536	-1731.217284	0.295681	-1731.521914	0.084531	0.077737	-1731.606445	-1731.599651

**D_3**	-1731.844801	-1731.217517	0.295883	-1731.522089	0.084324	0.077607	-1731.606413	-1731.599696
**D_4**	-1731.844704	-1731.217439	0.295706	-1731.522078	0.084408	0.077645	-1731.606486	-1731.599724
**D_5**	-1731.844798	-1731.217518	0.295914	-1731.522054	0.084292	0.077576	-1731.606346	-1731.599629
**D_6**	-1731.844720	-1731.217413	0.295691	-1731.522082	0.085095	0.077979	-1731.607177	-1731.600060
**D_7**	-1731.844459	-1731.217230	0.295819	-1731.521781	0.084246	0.077578	-1731.606027	-1731.599359
**D_8**	-1731.843462	-1731.216177	0.295469	-1731.520901	0.086372	0.078585	-1731.607272	-1731.599486
**D_9**	-1731.844728	-1731.217440	0.295368	-1731.522229	0.087494	0.079185	-1731.609723	-1731.601414
**E_1-notfa**	-1204.973304	-1204.595245	0.257248	-1204.697011	0.061686	0.059780	-1204.758697	-1204.756791
**E_1**	-1731.887173	-1731.260347	0.296606	-1731.563900	0.081135	0.075871	-1731.645036	-1731.639771
**E_10**	-1731.885387	-1731.257726	0.296696	-1731.562058	0.080625	0.075726	-1731.642683	-1731.637784
**E_11**	-1731.885474	-1731.258557	0.296705	-1731.562217	0.080142	0.075481	-1731.642359	-1731.637698
**E_12**	-1731.882873	-1731.254595	0.296108	-1731.559903	0.083292	0.076980	-1731.643194	-1731.636882
**E_13**	-1731.883003	-1731.255073	0.296228	-1731.559977	0.083222	0.076925	-1731.643199	-1731.636902
**E_14**	-1731.882749	-1731.255028	0.296069	-1731.559717	0.084179	0.077397	-1731.643895	-1731.637114
**E_15**	-1731.882823	-1731.254650	0.296398	-1731.559717	0.082049	0.076252	-1731.641767	-1731.635970
**E_16**	-1731.884469	-1731.257542	0.296592	-1731.561333	0.079835	0.075379	-1731.641169	-1731.636713
**E_2-notfa**	-1204.973098	-1204.595019	0.257392	-1204.696715	0.061552	0.059672	-1204.758267	-1204.756387
**E_2**	-1731.886353	-1731.259188	0.296301	-1731.563282	0.082103	0.076372	-1731.645386	-1731.639654
**E_3-notfa**	-1204.969673	-1204.591487	0.257287	-1204.693281	0.062333	0.060001	-1204.755614	-1204.753282
**E_3**	-1731.886880	-1731.260029	0.296526	-1731.563655	0.081316	0.075976	-1731.644971	-1731.639631
**E_4-notfa**	-1204.969452	-1204.591235	0.257176	-1204.693133	0.062375	0.060063	-1204.755508	-1204.753197
**E_4**	-1731.886355	-1731.258604	0.296534	-1731.563160	0.081844	0.076231	-1731.645004	-1731.639391
**E_5**	-1731.886277	-1731.259041	0.296510	-1731.563188	0.081231	0.075939	-1731.644419	-1731.639128
**E_6**	-1731.886083	-1731.258299	0.296541	-1731.562872	0.081754	0.076208	-1731.644626	-1731.639080
**E_7**	-1731.885613	-1731.258889	0.295976	-1731.562708	0.083186	0.076904	-1731.645895	-1731.639613
**E_8**	-1731.885910	-1731.258945	0.296510	-1731.562616	0.081045	0.075986	-1731.643661	-1731.638602
**E_9**	-1731.886213	-1731.258350	0.296157	-1731.563210	0.083117	0.076887	-1731.646327	-1731.640097
**F_1-notfa**	-1205.327237	-1204.949830	0.267446	-1205.040742	0.061299	0.059468	-1205.102041	-1205.100210
**F_1**	-1731.781811	-1731.155602	0.295725	-1731.459369	0.082211	0.076205	-1731.541579	-1731.535574
**F_10**	-1731.787417	-1731.161627	0.294181	-1731.465949	0.084508	0.077613	-1731.550456	-1731.543562
**F_2-notfa**	-1205.321812	-1204.944382	0.267986	-1205.034868	0.061369	0.059279	-1205.096238	-1205.094147
**F_2**	-1731.784910	-1731.159914	0.295489	-1731.462748	0.082091	0.076165	-1731.544839	-1731.538913
**F_3**	-1731.790063	-1731.164246	0.294389	-1731.468622	0.083697	0.077291	-1731.552319	-1731.545913
**F_4**	-1731.785548	-1731.160878	0.295973	-1731.463066	0.081863	0.075825	-1731.544929	-1731.538891
**F_5**	-1731.787606	-1731.162312	0.293922	-1731.466485	0.084117	0.077320	-1731.550602	-1731.543804
**F_6**	-1731.784912	-1731.159117	0.294946	-1731.463042	0.081970	0.076305	-1731.545012	-1731.539347

**F_7**	-1731.777814	-1731.152518	0.294191	-1731.456227	0.085361	0.078244	-1731.541588	-1731.534471	
**F_8**	-1731.773444	-1731.148038	0.295241	-1731.451155	0.084898	0.077650	-1731.536053	-1731.528805	
**F_9**	-1731.778632	-1731.153277	0.293996	-1731.457297	0.084917	0.078084	-1731.542214	-1731.535381	
**G_1-notfa**	-1205.346982	-1204.946996	0.269497	-1205.059258	0.059418	0.057726	-1205.118676	-1205.116984	
**G_1**	-1731.805203	-1731.184240	0.296918	-1731.482549	0.079005	0.073776	-1731.561555	-1731.556325	
**G_10**	-1731.807314	-1731.186546	0.296209	-1731.484964	0.080408	0.074727	-1731.565371	-1731.559691	
**G_11**	-1731.800653	-1731.179547	0.297134	-1731.477684	0.080073	0.074373	-1731.557756	-1731.552057	
**G_12**	-1731.801244	-1731.180123	0.296796	-1731.478398	0.080734	0.074649	-1731.559132	-1731.553047	
**G_13**	-1731.796239	-1731.174509	0.297274	-1731.472931	0.082915	0.075655	-1731.555846	-1731.548586	
**G_14**	-1731.801879	-1731.180411	0.296523	-1731.479135	0.082130	0.075674	-1731.561265	-1731.554809	
**G_15**	-1731.801541	-1731.180097	0.296919	-1731.478525	0.081919	0.075469	-1731.560444	-1731.553994	
**G_2-notfa**	-1205.340757	-1204.967293	0.271547	-1205.051803	0.057048	0.055783	-1205.108852	-1205.107587	
**G_2**	-1731.804771	-1731.183924	0.296962	-1731.482049	0.078507	0.073602	-1731.560557	-1731.555652	
**G_3**	-1731.803934	-1731.183316	0.296954	-1731.481157	0.079295	0.073972	-1731.560452	-1731.555129	
**G_4**	-1731.802628	-1731.181366	0.296546	-1731.480063	0.080294	0.074431	-1731.560356	-1731.554493	
**G_5**	-1731.805258	-1731.184511	0.296630	-1731.482747	0.079422	0.074115	-1731.562169	-1731.556862	
**G_6**	-1731.800673	-1731.179696	0.296751	-1731.478056	0.079732	0.074249	-1731.557788	-1731.552304	
**G_7**	-1731.798230	-1731.177134	0.297241	-1731.475237	0.079129	0.073721	-1731.554366	-1731.548958	
**G_8**	-1731.798019	-1731.175862	0.297624	-1731.474656	0.080505	0.074317	-1731.555161	-1731.548973	
**G_9**	-1731.803905	-1731.181981	0.296764	-1731.481102	0.080382	0.074657	-1731.561484	-1731.555759	
**H_1-notfa**	-1204.960984	-1204.588165	0.258540	-1204.684680	0.058281	0.056767	-1204.742962	-1204.741448	
**H_1**	-1731.872987	-1731.250573	0.298310	-1731.549176	0.079078	0.073635	-1731.628254	-1731.622811	
**H_2-notfa**	-1204.959531	-1204.586747	0.258665	-1204.683061	0.058886	0.056947	-1204.741946	-1204.740008	
**H_2**	-1731.870642	-1731.247716	0.297792	-1731.547241	0.079575	0.074065	-1731.626816	-1731.621306	
**H_3**	-1731.872422	-1731.250142	0.298036	-1731.548824	0.078273	0.073416	-1731.627097	-1731.622240	
**H_4**	-1731.871022	-1731.248134	0.297946	-1731.547489	0.080003	0.074177	-1731.627492	-1731.621666	
**H_5**	-1731.872265	-1731.250032	0.298157	-1731.548518	0.078607	0.073559	-1731.627126	-1731.622078	
**H_6**	-1731.866911	-1731.244525	0.297752	-1731.543279	0.079887	0.074289	-1731.623166	-1731.617567	
**H_7**	-1731.867632	-1731.245365	0.297708	-1731.544105	0.078737	0.073744	-1731.622841	-1731.617848	
**H_8**	-1731.869389	-1731.246662	0.297840	-1731.545926	0.079842	0.074115	-1731.625769	-1731.620041	
**TFA-anion**	-526.438895	-526.190256	0.025655	-526.406295	0.034725	0.034181	-526.441021	-526.440476	
**TFAH**	-526.895846	-526.644630	0.038100	-526.850463	0.035674	0.034872	-526.886137	-526.885335	
**TS-III_1**	-1731.772262	-1731.146907	0.292346	-1731.453693	0.080857	0.075258	-1731.534551	-1731.528952	-107.74
**TS-III_2**	-1731.775335	-1731.150027	0.292488	-1731.456632	0.080638	0.075252	-1731.537270	-1731.531884	-91.00
**TS-III_3**	-1731.772260	-1731.146907	0.292338	-1731.453701	0.080815	0.075236	-1731.534516	-1731.528937	-107.97
**TS-III_4**	-1731.775634	-1731.150307	0.292676	-1731.456725	0.080975	0.075427	-1731.537701	-1731.532152	-77.91

**TS-III_5**	-1731.775284	-1731.149905	0.292943	-1731.455991	0.082661	0.075890	-1731.538652	-1731.531881	-103.35
**TS-II_1-notfa**	-1205.299450	-1204.921838	0.266833	-1205.013769	0.061044	0.059010	-1205.074813	-1205.072779	-138.33
**TS-II_1**	-1731.756046	-1731.131029	0.292963	-1731.436066	0.083222	0.076934	-1731.519288	-1731.512999	-131.28
**TS-II_10**	-1731.758379	-1731.132706	0.293975	-1731.437594	0.083069	0.076833	-1731.520663	-1731.514426	-202.94
**TS-II_11**	-1731.748433	-1731.123084	0.293871	-1731.427905	0.080789	0.075548	-1731.508694	-1731.503454	-166.33
**TS-II_12**	-1731.762549	-1731.137385	0.293532	-1731.442171	0.081932	0.076381	-1731.524103	-1731.518552	-115.07
**TS-II_13**	-1731.753124	-1731.127730	0.293116	-1731.433103	0.081418	0.076174	-1731.514522	-1731.509277	-143.49
**TS-II_2-notfa**	-1205.297319	-1204.919654	0.267541	-1205.011233	0.059632	0.058260	-1205.070865	-1205.069493	-153.9
**TS-II_2**	-1731.754402	-1731.128765	0.293380	-1731.434098	0.082234	0.076250	-1731.516332	-1731.510349	-115.88
**TS-II_3**	-1731.758010	-1731.132879	0.293696	-1731.437521	0.080888	0.075793	-1731.518409	-1731.513314	-118.25
**TS-II_4**	-1731.755383	-1731.130394	0.293809	-1731.434813	0.082188	0.076265	-1731.517001	-1731.511078	-132.05
**TS-II_5**	-1731.759698	-1731.134043	0.293823	-1731.439115	0.081811	0.076131	-1731.520926	-1731.515246	-218.04
**TS-II_6**	-1731.757890	-1731.132091	0.293816	-1731.437268	0.081671	0.076162	-1731.518938	-1731.513430	-145.99
**TS-II_7**	-1731.757894	-1731.132198	0.293516	-1731.437543	0.081638	0.076136	-1731.519181	-1731.513680	-154.57
**TS-II_8**	-1731.755841	-1731.130064	0.293660	-1731.435326	0.082201	0.076370	-1731.517527	-1731.511696	-156.41
**TS-II_9**	-1731.757002	-1731.131320	0.293506	-1731.436539	0.083061	0.076830	-1731.519600	-1731.513369	-248.68
**TS-IV_1-notfa**	-1205.297924	-1204.920383	0.267964	-1205.011634	0.058664	0.057793	-1205.070298	-1205.069427	-174.03
**TS-IV_1**	-1731.757186	-1731.132171	0.294214	-1731.436601	0.079184	0.074747	-1731.515785	-1731.511348	-112.62
**TS-IV_10**	-1731.754563	-1731.129955	0.293693	-1731.434397	0.080061	0.075224	-1731.514458	-1731.509620	-184.94
**TS-IV_11**	-1731.758874	-1731.133492	0.293484	-1731.438619	0.082141	0.076401	-1731.520761	-1731.515021	-179.04
**TS-IV_12**	-1731.757432	-1731.131722	0.293802	-1731.437094	0.079691	0.075167	-1731.516785	-1731.512260	-172.51
**TS-IV_13**	-1731.757454	-1731.131706	0.293769	-1731.437066	0.079834	0.075382	-1731.516900	-1731.512448	-183.13
**TS-IV_14**	-1731.755036	-1731.129209	0.293411	-1731.434881	0.080765	0.075818	-1731.515646	-1731.510699	-186.34
**TS-IV_15**	-1731.752645	-1731.126952	0.293172	-1731.432586	0.081543	0.076255	-1731.514129	-1731.508840	-182.24
**TS-IV_16**	-1731.759779	-1731.134406	0.293783	-1731.439386	0.080735	0.075748	-1731.520121	-1731.515134	-177.91
**TS-IV_17**	-1731.754598	-1731.128972	0.293317	-1731.434534	0.081335	0.076122	-1731.515869	-1731.510655	-177.21
**TS-IV_18**	-1731.759931	-1731.134612	0.293532	-1731.439734	0.081204	0.075928	-1731.520938	-1731.515662	-193.66
**TS-IV_2-notfa**	-1205.298331	-1204.920832	0.266758	-1205.012820	0.059980	0.058791	-1205.072800	-1205.071611	-171.69
**TS-IV_2**	-1731.755295	-1731.129568	0.293261	-1731.435232	0.080878	0.076009	-1731.516110	-1731.511241	-198.18
**TS-IV_3**	-1731.756385	-1731.131616	0.294392	-1731.435540	0.079115	0.075044	-1731.514655	-1731.510585	-191.66
**TS-IV_4**	-1731.757169	-1731.132329	0.293403	-1731.437004	0.083148	0.076892	-1731.520152	-1731.513896	-167.63
**TS-IV_5**	-1731.759145	-1731.133473	0.294154	-1731.438345	0.080580	0.075596	-1731.518925	-1731.513941	-227.52
**TS-IV_6**	-1731.754287	-1731.128634	0.294501	-1731.433259	0.080519	0.075416	-1731.513778	-1731.508675	-220.42
**TS-IV_7**	-1731.756735	-1731.131521	0.294653	-1731.435854	0.078399	0.074387	-1731.514253	-1731.510241	-184.04
**TS-IV_8**	-1731.756983	-1731.131805	0.293589	-1731.436850	0.079317	0.075150	-1731.516167	-1731.512001	-162.00
**TS-IV_9**	-1731.759774	-1731.134133	0.293519	-1731.439598	0.080136	0.075493	-1731.519734	-1731.515092	-222.14

**TS-I_1-notfa**	-1205.293597	-1204.913077	0.266643	-1205.008078	0.061499	0.059280	-1205.069577	-1205.067359	-255.68
**TS-I_1**	-1731.752422	-1731.125558	0.292999	-1731.432451	0.082084	0.076435	-1731.514535	-1731.508886	-138.55
**TS-I_2-notfa**	-1205.293171	-1204.912685	0.266470	-1205.007802	0.061147	0.059285	-1205.068949	-1205.067087	-254.10
**TS-I_2**	-1731.752093	-1731.125244	0.294119	-1731.431441	0.081210	0.075483	-1731.512651	-1731.506923	-134.21
**TS-I_3-notfa**	-1205.291687	-1204.911579	0.267120	-1205.005958	0.060520	0.058679	-1205.066478	-1205.064637	-239.04
**TS-I_4**	-1731.752360	-1731.124614	0.293190	-1731.432198	0.083620	0.077282	-1731.515818	-1731.509480	-199.93
**TS-I_5**	-1731.753086	-1731.125914	0.293919	-1731.432699	0.080563	0.075360	-1731.513262	-1731.508059	-170.67
**TS-I_6**	-1731.749785	-1731.122098	0.293549	-1731.429510	0.081311	0.076047	-1731.510821	-1731.505558	-240.11
**TS-V_1**	-1731.784518	-1731.159936	0.293743	-1731.464335	0.081437	0.075516	-1731.545772	-1731.539851	-202.20
**TS-V_2**	-1731.777927	-1731.153093	0.293678	-1731.457506	0.084237	0.077154	-1731.541743	-1731.534660	-115.37
**TS-V_3**	-1731.785262	-1731.160890	0.294199	-1731.464895	0.079969	0.074643	-1731.544864	-1731.539538	-200.80
**TS-V_4**	-1731.785115	-1731.160803	0.293978	-1731.464893	0.081338	0.075367	-1731.546231	-1731.540260	-204.39
**TS-V_5**	-1731.783014	-1731.159314	0.295371	-1731.461959	0.079173	0.073897	-1731.541132	-1731.535855	-219.47
**TS-V_6**	-1731.786536	-1731.162002	0.294441	-1731.465866	0.081060	0.075170	-1731.546926	-1731.541036	-216.33
**TS-V_7**	-1731.779765	-1731.156264	0.295151	-1731.458766	0.079498	0.074268	-1731.538264	-1731.533034	-247.21
**TS-V_8**	-1731.778810	-1731.153949	0.296003	-1731.456903	0.081560	0.074963	-1731.538464	-1731.531866	-214.20
**TS-V_9**	-1731.782963	-1731.158408	0.294202	-1731.462452	0.079662	0.074727	-1731.542114	-1731.537179	-88.41

Table S 8.5: Compiled thermochemical data in atomic units for structures computed at the M06-2X-D3/Def2-QZVPP(SMD=CH_2Cl_2)//M06-2X-D3/6-31+G(d,p)(CPCM=CH_2Cl_2) level of theory.

7.9. XYZ Coordinates for Computed Structures

40
2-H-3-COOMe-A1-1 Eopt -1767.053476
C -0.482332 0.044203 1.074481
C 0.138614 -1.000335 0.376148
C 0.512978 -2.197501 0.950919
C 0.238886 -2.355901 2.315108
C -0.378803 -1.336083 3.036327
C -0.742428 -0.127615 2.430723
C -0.757498 1.198945 0.196383
C -0.375852 1.030514 -1.074493
H 1.000834 -2.975582 0.373812
H 0.516311 -3.280570 2.810197
H -0.580210 -1.476579 4.093454
H -1.215457 0.661310 3.002082
S 0.302510 -0.598469 -1.355043
O -1.095855 -1.356920 -1.967556
C -2.322912 -1.314157 -1.309485
C -2.659332 -2.350688 -0.441863
C -3.208863 -0.274821 -1.583166
C -3.906347 -2.329859 0.180733
H -1.953741 -3.156769 -0.267538
C -4.453669 -0.266765 -0.955099
H -2.923563 0.508568 -2.278982
C -4.800318 -1.287334 -0.068682
H -4.178273 -3.132359 0.859191
H -5.151879 0.538350 -1.161755
H -5.768843 -1.274419 0.421114
C -1.427337 2.448292 0.669741
O -1.781962 2.625110 1.813314
C 3.120999 -0.381369 -0.561716
O 2.189832 0.472261 -0.664692
O 3.087675 -1.597924 -0.721514
C 4.471833 0.269092 -0.154835
F 4.380061 0.844627 1.058957
F 5.473990 -0.615012 -0.101375
F 4.832595 1.232662 -1.021485
H -0.454456 1.707948 -1.915403
O -1.592752 3.325377 -0.313996
C -2.234836 4.559739 0.049703
H -2.276696 5.145286 -0.866465
H -3.239881 4.359997 0.426155
H -1.646326 5.076460 0.809960
40
2-H-3-COOMe-A1-2 Eopt -1767.052438
C -0.536682 0.369624 0.883929
C 0.109270 -0.831553 0.555449
C 0.440749 -1.809160 1.470717
C 0.093160 -1.569765 2.805716
C -0.554144 -0.389858 3.164995
C -0.871864 0.589898 2.217121
C -0.753485 1.211586 -0.313098
C -0.304686 0.673573 -1.452406
H 0.952227 -2.716770 1.168553
H 0.335224 -2.309778 3.561076
H -0.815702 -0.219492 4.204383
H -1.367601 1.503635 2.518565
S 0.362337 -0.960288 -1.206382
O -1.021243 -1.862743 -1.635944
C -2.269937 -1.641296 -1.060285
C -3.135609 -0.712428 -1.633121
C -2.645882 -2.405558 0.042280
C -4.399844 -0.533962 -1.072395
H -2.821614 -0.143401 -2.503106
C -3.911827 -2.216668 0.593851
H -1.954336 -3.135666 0.450578
C -4.786010 -1.277880 0.043290
H -5.082047 0.187071 -1.511838
H -4.213813 -2.806784 1.453466
H -5.770008 -1.132640 0.477923
C -1.423937 2.549682 -0.374384
O -1.720419 3.087410 -1.417120
C 3.145326 -0.525919 -0.390126
O 2.229169 0.261108 -0.774856
O 3.099251 -1.733776 -0.177073
C 4.493702 0.210432 -0.158878
F 4.377963 1.127736 0.820199
F 5.481007 -0.621531 0.189694
F 4.891297 0.863997 -1.265350
H -0.335351 1.080281 -2.455683
O -1.650599 3.061904 0.828109
C -2.293017 4.349765 0.850820
H -3.272401 4.284484 0.373643
H -2.393646 4.604321 1.903898
H -1.671702 5.083233 0.333990
40
2-H-3-COOMe-A1-3 Eopt -1767.052411
C -0.530212 0.359997 0.891508
C 0.121910 -0.834782 0.551564
C 0.462849 -1.817693 1.457680
C 0.119867 -1.590769 2.795973
C -0.530477 -0.416325 3.167325
C -0.858138 0.568841 2.228509
C -0.748650 1.212987 -0.297759
C -0.296759 0.687734 -1.441854
H 0.978780 -2.719622 1.146281
H 0.369155 -2.335371 3.544442
H -0.786762 -0.254418 4.209387
H -1.356283 1.477802 2.539831
S 0.377242 -0.945381 -1.211059
O -0.999996 -1.851044 -1.652108
C -2.251948 -1.638948 -1.079860
C -3.120919 -0.714064 -1.654093
C -2.627527 -2.408122 0.019365
C -4.388706 -0.545069 -1.098498
H -2.806531 -0.141729 -2.521816
C -3.896941 -2.228434 0.566022
H -1.933176 -3.134854 0.428960
C -4.774794 -1.294072 0.013826
H -5.073976 0.172193 -1.539343
H -4.198788 -2.822404 1.423022
H -5.761582 -1.156205 0.444492
C -1.425649 2.548180 -0.350602
O -1.580380 3.164920 -1.380096
C 3.158967 -0.499286 -0.387623
O 2.237358 0.284319 -0.765965
O 3.122521 -1.709647 -0.187088
C 4.501469 0.245551 -0.149074
F 4.373840 1.167629 0.823846
F 5.490684 -0.579129 0.211284
F 4.903679 0.895014 -1.256484
H -0.330900 1.103464 -2.441352
O -1.838878 2.958223 0.841471
C -2.508425 4.231956 0.869818
H -3.404921 4.194109 0.248561
H -2.769369 4.398468 1.912977
H -1.835838 5.012774 0.510596
40
2-H-3-COOMe-A1-4 Eopt -1767.050306
C -0.511387 0.118365 1.070562
C -0.677175 -1.249163 0.819464
C -1.068914 -2.173922 1.767203
C -1.331985 -1.686653 3.052358
C -1.184210 -0.329967 3.332946
C -0.767517 0.582432 2.356113
C -0.055177 0.833752 -0.141146
C 0.040887 0.066654 -1.234226
H -1.173362 -3.228044 1.528953
H -1.645950 -2.373401 3.831035
H -1.386108 0.031718 4.336065
H -0.639645 1.628604 2.601589
S -0.346568 -1.627748 -0.886567
O -1.877342 -1.806918 -1.526479
C -2.894483 -0.889030 -1.226720
C -3.767364 -1.181249 -0.183810
C -3.039757 0.243274 -2.020721
C -4.811273 -0.295542 0.079163
H -3.632697 -2.087004 0.398194
C -4.089672 1.118971 -1.746070
H -2.349357 0.427461 -2.838070
C -4.969288 0.854383 -0.695576
H -5.501898 -0.509017 0.888689
H -4.218604 2.006393 -2.357367
H -5.783126 1.541105 -0.485535
C 0.364435 2.268232 -0.241549
O 0.754859 2.763729 -1.274268
C 2.770054 -0.567607 0.142972
O 2.081931 -1.541993 -0.254193
O 2.476962 0.383776 0.873133
C 4.212287 -0.576721 -0.445215
F 5.016980 0.341585 0.107707
F 4.814231 -1.771195 -0.291956
F 4.182376 -0.324056 -1.772399
H 0.385926 0.332514 -2.225787
O 0.254582 2.913389 0.910495
C 0.690729 4.284186 0.903908
H 0.091415 4.863453 0.199205
H 0.541123 4.640755 1.921095
H 1.746015 4.334182 0.629396
40
2-H-3-COOMe-A1-5 Eopt -1767.049299
C 0.773252 1.514110 0.938991
C 0.426964 0.227123 1.376172
C 0.837181 -0.326632 2.570548
C 1.656881 0.464634 3.384273
C 2.025360 1.745611 2.980332
C 1.591525 2.282888 1.763353
C 0.169461 1.825759 -0.374679
C -0.570588 0.838261 -0.890781
H 0.549212 -1.332106 2.857379
H 2.008851 0.069588 4.331317
H 2.664614 2.344787 3.620718
H 1.890253 3.280440 1.468426
S -0.657480 -0.573306 0.204991
O -2.068443 -0.065420 1.044057

C -3.247655 -0.517233 0.455082
C -3.658441 -1.834039 0.648427
C -4.004976 0.384401 -0.288058
C -4.862642 -2.251849 0.083569
H -3.046267 -2.511074 1.237349
C -5.210693 -0.044657 -0.841727
H -3.651003 1.403417 -0.415188
C -5.638652 -1.360372 -0.658979
H -5.195241 -3.274903 0.228723
H -5.813744 0.650759 -1.417145
H -6.576625 -1.690883 -1.093853
C 0.310261 3.091073 -1.163913
O -0.325468 3.313143 -2.169329
C 1.732805 -2.112640 -0.330877
O 1.220071 -1.076102 -0.858826
O 1.360314 -2.753643 0.644563
C 3.023145 -2.563378 -1.066947
F 3.531958 -3.691226 -0.561217
F 2.791976 -2.775125 -2.374026
F 3.976743 -1.617535 -0.981066
H -1.093630 0.804616 -1.838423
O 1.204503 3.920963 -0.641809
C 1.406188 5.155183 -1.354205
H 0.473335 5.720598 -1.389695
H 2.163092 5.695233 -0.788962
H 1.756675 4.946398 -2.366454
40
2-H-3-COOMe-A1-6 Eopt -1767.050448
C 0.539175 1.608959 1.137314
C 0.493676 0.250682 1.479184
C 1.061341 -0.281504 2.617867
C 1.723352 0.612410 3.468438
C 1.793903 1.968094 3.154584
C 1.206738 2.481354 1.992770
C -0.156391 1.880559 -0.135885
C -0.686778 0.800495 -0.720492
H 1.010773 -1.342670 2.835306
H 2.189522 0.238552 4.373887
H 2.316417 2.644470 3.823365
H 1.269198 3.536686 1.757863
S -0.437047 -0.674719 0.268459
O -1.895294 -0.534853 1.168956
C -2.980258 -1.148504 0.547666
C -3.104510 -2.535637 0.575007
C -3.936901 -0.339872 -0.060503
C -4.220550 -3.122028 -0.020509
H -2.342174 -3.137848 1.061065
C -5.052127 -0.938735 -0.645344
H -3.803872 0.738090 -0.059740
C -5.193526 -2.327106 -0.628447
H -4.330593 -4.201794 -0.003831
H -5.809137 -0.318607 -1.115278
H -6.061770 -2.789422 -1.087338
C -0.262331 3.245206 -0.735875
O 0.180919 4.242434 -0.212151
C 2.188475 -1.644939 -0.444411
O 1.455696 -0.697472 -0.871561
O 2.006338 -2.402189 0.501057
C 3.485594 -1.800013 -1.283427
F 4.256569 -2.802730 -0.851507
F 3.201430 -2.037742 -2.576380
F 4.223632 -0.676386 -1.239920
H -1.223456 0.713964 -1.656703
O -0.901055 3.228846 -1.900556
C -1.044395 4.501978 -2.553665
H -1.577721 4.294215 -3.479053
H -1.618236 5.183189 -1.922608
H -0.060649 4.925861 -2.763603
33
2-H-3-COOMe-A2-1 Eopt -1240.828084
C 0.244429 1.458071 -0.162211
C -0.895806 1.496240 0.654859
C -2.006717 2.277045 0.400467
C -1.978830 3.044337 -0.771342
C -0.863574 3.020967 -1.604315
C 0.264047 2.240487 -1.308523
C 1.274910 0.556903 0.394558
C 0.925635 -0.053223 1.536101
H -2.864233 2.289815 1.066252
H -2.832428 3.664628 -1.021884
H -0.858084 3.628700 -2.503153
H 1.132472 2.249772 -1.955574
H 1.485652 -0.741058 2.157070
S -0.700596 0.433343 2.058560
O -1.645047 -0.887972 1.772658
C -1.555852 -1.538769 0.522894
C -0.634835 -2.569202 0.378743
C -2.437037 -1.166749 -0.484877
C -0.594291 -3.250630 -0.836324
H 0.019731 -2.836417 1.202509
C -2.380594 -1.858787 -1.694226
H -3.154741 -0.369970 -0.319407
C -1.460197 -2.892436 -1.870769
H 0.112511 -4.063020 -0.969995
H -3.061691 -1.590165 -2.494921
H -1.422636 -3.426385 -2.814805
C 2.606577 0.331931 -0.259693
O 2.951904 0.909152 -1.263691
O 3.326499 -0.567026 0.392057
C 4.623194 -0.857238 -0.166323
H 5.058100 -1.607228 0.490628
H 4.509439 -1.247367 -1.179184
H 5.232238 0.048303 -0.176805
33
2-H-3-COOMe-A2-2 Eopt -1240.827222
C 0.753017 1.174947 -0.017281
C -0.425874 1.660363 0.572063
C -1.141586 2.745350 0.104272
C -0.654182 3.368329 -1.051699
C 0.510589 2.909854 -1.659719
C 1.232508 1.820814 -1.149179
C 1.297266 0.027380 0.744199
C 0.564921 -0.330280 1.809068
H -2.041649 3.095056 0.600422
H -1.186685 4.217883 -1.464876
H 0.880189 3.411113 -2.548233
H 2.146218 1.495807 -1.629586
H 0.741941 -1.120988 2.528341
S -0.850916 0.714953 2.008588
O -2.135341 -0.225306 1.576932
C -2.082340 -0.946158 0.363890
C -1.548465 -2.229174 0.374830
C -2.628008 -0.364793 -0.774311
C -1.559183 -2.955798 -0.814693
H -1.148172 -2.649204 1.292332
C -2.628160 -1.106154 -1.955060
H -3.053179 0.632487 -0.729526
C -2.091647 -2.394114 -1.976154
H -1.153753 -3.962168 -0.829103
H -3.053388 -0.675363 -2.855544
H -2.095146 -2.965444 -2.898837
C 2.543682 -0.758599 0.442140
O 2.963426 -1.614509 1.184042
O 3.089228 -0.409889 -0.710999
C 4.281752 -1.129317 -1.086103
H 4.054326 -2.191870 -1.185845
H 4.583690 -0.707242 -2.041992
H 5.056007 -0.977585 -0.332341
33
2-H-3-COOMe-A2-3 Eopt -1240.827096
C 0.814019 1.103331 -0.015120
C -0.319710 1.630991 0.623612
C -0.992653 2.763269 0.207632
C -0.506450 3.394015 -0.944782
C 0.612823 2.892619 -1.602697
C 1.290904 1.752739 -1.145943
C 1.307671 -0.104401 0.684155
C 0.582477 -0.463809 1.753197
H -1.862256 3.140712 0.737244
H -1.005790 4.281436 -1.317873
H 0.979833 3.398450 -2.489688
H 2.169616 1.392644 -1.665632
H 0.736940 -1.285574 2.442422
S -0.757537 0.658934 2.038710
O -2.115017 -0.177099 1.618527
C -2.153576 -0.844272 0.374810
C -1.680743 -2.148831 0.301846
C -2.726630 -0.186284 -0.706673
C -1.783516 -2.817443 -0.917123
H -1.257027 -2.629783 1.178249
C -2.820851 -0.871157 -1.917491
H -3.101181 0.825908 -0.595102
C -2.346633 -2.179159 -2.023252
H -1.426119 -3.838769 -0.997308
H -3.270169 -0.380400 -2.774613
H -2.423119 -2.706097 -2.968889
C 2.500180 -0.943441 0.316309
O 2.688625 -2.038840 0.789366
O 3.288623 -0.339432 -0.556723
C 4.463670 -1.075111 -0.955660
H 4.167694 -2.008840 -1.436430
H 4.985093 -0.427158 -1.656594
H 5.083335 -1.280839 -0.081419
33
2-H-3-COOMe-A2-5 Eopt -1240.821840
C 1.859286 0.750242 0.041560
C 0.714106 1.524379 0.287480
C 0.649377 2.896273 0.147274
C 1.817210 3.535655 -0.287636
C 2.971581 2.799461 -0.536181
C 3.011428 1.407298 -0.371120
C 1.595667 -0.688286 0.273270
C 0.338299 -0.971199 0.645018
H -0.257840 3.453710 0.358204
H 1.816870 4.611584 -0.422705
H 3.869837 3.312623 -0.863458
H 3.924324 0.859218 -0.564613
H -0.104665 -1.928532 0.892342
S -0.628575 0.503600 0.823049
O -1.578219 0.533087 -0.525463
C -2.879687 0.002634 -0.342961
C -3.881991 0.834075 0.138058
C -3.097773 -1.317337 -0.712044

C -5.168489 0.309015 0.248451
H -3.662437 1.862925 0.407697
C -4.391925 -1.824195 -0.597113
H -2.278171 -1.920629 -1.091282
C -5.421656 -1.014349 -0.117011
H -5.972682 0.937529 0.616770
H -4.592061 -2.850999 -0.885049
H -6.426602 -1.414485 -0.029288
C 2.571440 -1.826251 0.141631
O 2.240240 -2.980492 0.273150
O 3.797978 -1.408056 -0.121536
C 4.799936 -2.437706 -0.250094
H 4.534478 -3.110389 -1.066983
H 5.726992 -1.911694 -0.467118
H 4.878314 -2.993029 0.685982
33
2-H-3-COOMe-A2-6 Eopt -1240.822800
C -1.954339 -0.860686 0.007944
C -0.819139 -1.636015 0.288721
C -0.767902 -3.011777 0.183121
C -1.938486 -3.648591 -0.248994
C -3.083532 -2.908104 -0.528635
C -3.110773 -1.511653 -0.400229
C -1.691567 0.578832 0.213075
C -0.443218 0.869186 0.607790
H 0.129718 -3.574511 0.418811
H -1.948037 -4.727631 -0.356147
H -3.983267 -3.421679 -0.851228
H -4.012392 -0.951523 -0.614853
H 0.007957 1.824477 0.845830
S 0.518990 -0.609980 0.823717
O 1.483173 -0.658974 -0.514744
C 2.772339 -0.099169 -0.333320
C 3.783986 -0.895471 0.186152
C 2.971128 1.212176 -0.742459
C 5.058977 -0.342839 0.295253
H 3.580649 -1.919336 0.485466
C 4.254103 1.746774 -0.628559
H 2.145799 1.787087 -1.152045
C 5.292441 0.972623 -0.109666
H 5.869883 -0.944084 0.693111
H 4.439046 2.767114 -0.947962
H 6.288703 1.394122 -0.023005
C -2.731241 1.639736 -0.002472
O -3.853589 1.393881 -0.377135
O -2.261125 2.848004 0.263313
C -3.188441 3.936574 0.082342
H -2.630273 4.835050 0.336031
H -3.523287 3.968575 -0.955638
H -4.042027 3.809145 0.750306
33
2-H-3-COOMe-B-1 Eopt -1240.830249
C -0.065035 0.223401 -1.022442
C -1.264748 0.971419 -0.966989
C -2.487214 0.464680 -1.443731
C -2.499158 -0.807036 -2.000164
C -1.320880 -1.563012 -2.059928
C -0.105663 -1.064839 -1.573721
C 1.030767 0.970680 -0.425912
C 0.646909 2.211566 -0.003541
H -3.392707 1.061777 -1.388977
H -3.427233 -1.217994 -2.382435
H -1.343271 -2.555802 -2.498885
H 0.797411 -1.657866 -1.653015
H 1.278542 2.956063 0.466351
S -1.015619 2.537592 -0.267806
O -1.152339 1.196680 2.399770
C -0.983322 0.052375 2.002733
C 0.367851 -0.569445 2.021552
C -2.119924 -0.743509 1.481688
C 0.543723 -1.814097 1.501938
H 1.170389 0.005247 2.476357
C -1.900126 -1.989360 0.987579
H -3.105810 -0.291187 1.536416
C -0.569700 -2.492115 0.931986
H 1.518597 -2.289030 1.487622
H -2.710080 -2.594847 0.596515
H -0.407477 -3.473542 0.493019
C 2.443924 0.544239 -0.255860
O 3.289928 1.223552 0.284419
O 2.672327 -0.672239 -0.751628
C 4.014270 -1.167113 -0.605040
H 4.277473 -1.228362 0.452905
H 4.010291 -2.156717 -1.058234
H 4.714410 -0.510147 -1.124117
33
2-H-3-COOMe-B-2 Eopt -1240.831675
C -0.171444 0.346981 -1.010525
C -1.124171 1.326766 -0.650525
C -2.472528 1.236126 -1.043651
C -2.857097 0.152249 -1.820510
C -1.923853 -0.831193 -2.179527
C -0.584009 -0.748947 -1.779283
C 1.131439 0.665507 -0.456441
C 1.131353 1.842808 0.235991
H -3.185829 2.003001 -0.756830
H -3.888539 0.063019 -2.144201
H -2.240905 -1.672015 -2.789046
H 0.133765 -1.501758 -2.084585
H 1.971665 2.305009 0.739250
S -0.408708 2.602542 0.279328
O -0.635362 0.773608 2.655555
C -0.820196 -0.255372 2.021055
C 0.305624 -1.178159 1.718345
C -2.173509 -0.607294 1.527238
C 0.088108 -2.276595 0.947538
H 1.273349 -0.934767 2.149180
C -2.347905 -1.724070 0.774825
H -2.989888 0.050134 1.811546
C -1.215611 -2.512405 0.425899
H 0.891030 -2.959315 0.693817
H -3.323949 -2.006628 0.396755
H -1.366803 -3.379231 -0.212566
C 2.334985 -0.182286 -0.631352
O 2.329008 -1.250729 -1.208164
O 3.416763 0.355765 -0.071296
C 4.630341 -0.404779 -0.188215
H 5.392210 0.184800 0.317952
H 4.513311 -1.376390 0.295786
H 4.887480 -0.541725 -1.240229
33
2-H-3-COOMe-C-1 Eopt -1240.841566
C -0.919068 -0.547497 -0.342859
C -2.166078 -0.552248 0.319377
C -3.301582 0.021552 -0.229198
C -3.237927 0.671737 -1.490442
C -2.036810 0.797096 -2.117933
C -0.759786 0.305932 -1.548696
C 0.040841 -1.292956 0.371903
C -0.468303 -1.791849 1.561918
H -4.251098 -0.032400 0.297911
H -4.143877 1.076981 -1.924709
H -1.966806 1.333031 -3.060982
H -0.176355 -0.199647 -2.328204
H 0.078838 -2.392170 2.281613
S -2.099179 -1.423429 1.827062
C 1.428669 -1.644825 -0.039524
O 2.294967 -1.968932 0.738806
C 0.105875 1.622190 -1.165679
C -0.453696 2.266854 0.065840
C 1.559533 1.284660 -1.075609
C 0.257384 2.425015 1.189078
H -1.490150 2.597476 0.023027
C 2.279041 1.454094 0.041107
H 2.023376 0.899658 -1.980702
C 1.668726 1.992035 1.275964
H -0.169050 2.884422 2.076168
H 3.338373 1.216040 0.082509
H -0.029454 2.294794 -2.022911
O 2.307848 2.093812 2.315670
O 1.571697 -1.607147 -1.362571
C 2.895033 -1.880550 -1.856670
H 2.830839 -1.770527 -2.937632
H 3.605038 -1.165153 -1.434897
H 3.189979 -2.896936 -1.590881
33
2-H-3-COOMe-C-2 Eopt -1240.842201
C -0.940531 -0.597428 -0.430136
C -2.033930 -0.919652 0.399573
C -3.316366 -0.462766 0.138428
C -3.564110 0.382599 -0.976430
C -2.520656 0.807561 -1.741388
C -1.110038 0.452912 -1.463138
C 0.228103 -1.275846 -0.030847
C 0.020243 -2.043254 1.105201
H -4.144382 -0.758533 0.778357
H -4.579914 0.694309 -1.187931
H -2.690677 1.495530 -2.565829
H -0.592166 0.190707 -2.394248
H 0.761946 -2.657351 1.604764
S -1.573438 -2.004097 1.685080
C 1.500057 -1.294393 -0.803466
O 1.551423 -1.070847 -1.993888
C -0.392778 1.816701 -0.958009
C -0.842959 2.163308 0.428350
C 1.090431 1.714612 -1.116053
C 0.004938 2.287512 1.457053
H -1.911284 2.303292 0.582623
C 1.942091 1.847858 -0.090730
H 1.458509 1.529223 -2.121768
C 1.461669 2.103326 1.283002
H -0.341487 2.533303 2.456757
H 3.017928 1.785224 -0.229629
H -0.754847 2.580692 -1.658215
O 2.234624 2.172890 2.231020
O 2.539115 -1.618326 -0.046919
C 3.807061 -1.703308 -0.723149
H 4.056855 -0.737534 -1.166622
H 4.527941 -1.969843 0.046643
H 3.765941 -2.471649 -1.497441
33
2-H-3-COOMe-C-3 Eopt -1240.843296

C 0.862297 0.686934 0.377587
C 0.994334 2.056428 0.080920
C 0.064093 2.995313 0.506991
C -1.062499 2.596027 1.269618
C -1.260125 1.274798 1.537692
C -0.368019 0.200406 1.048650
C 1.980999 -0.049605 -0.070523
C 2.909483 0.755253 -0.711201
H 0.198846 4.048425 0.271431
H -1.760843 3.348685 1.615387
H -2.143843 0.959729 2.087739
H -0.092754 -0.462001 1.883823
H 3.853322 0.429369 -1.134379
S 2.473168 2.392729 -0.780542
C 2.188189 -1.497435 0.192244
O 1.424128 -2.167490 0.856289
C -1.217659 -0.736627 0.072431
C -2.263541 -1.475989 0.845435
C -1.740159 0.030335 -1.102847
C -3.554122 -1.490961 0.489961
H -1.919937 -2.035538 1.713590
C -3.031065 0.022556 -1.456942
H -1.014770 0.595034 -1.686993
C -4.037670 -0.737520 -0.686116
H -4.295549 -2.055860 1.047595
H -3.390585 0.569826 -2.323481
H -0.492461 -1.477895 -0.287662
O -5.220344 -0.740897 -1.007145
O 3.303510 -1.954010 -0.360152
C 3.599364 -3.343605 -0.124375
H 2.796300 -3.967068 -0.521006
H 4.531267 -3.534160 -0.652337
H 3.718567 -3.520887 0.945890
33
2-H-3-COOMe-C-4 Eopt -
1240.841805
C -1.071365 -0.519308 0.353635
C -1.370079 -1.888816 0.205862
C -0.580402 -2.884543 0.763514
C 0.578156 -2.549614 1.510264
C 0.942856 -1.243568 1.633258
C 0.202689 -0.123585 1.008284
C -2.073513 0.289640 -0.226861
C -3.075015 -0.466942 -0.814845
H -0.851360 -3.931031 0.642920
H 1.165992 -3.341439 1.958805
H 1.851091 -0.983004 2.171928
H 0.002094 0.643108 1.771726
H -3.950054 -0.070854 -1.319890
S -2.856392 -2.139707 -0.669804
C -2.179019 1.775184 -0.227785
O -2.976314 2.384615 -0.902511
C 1.178869 0.590844 -0.034263
C 2.310270 1.256785 0.684542
C 1.602850 -0.355935 -1.114545
C 3.594008 1.054360 0.364281
H 2.040236 1.943664 1.484580
C 2.885854 -0.565939 -1.432651
H 0.811620 -0.869320 -1.658988
C 3.980594 0.119989 -0.714378
H 4.401007 1.565235 0.881468
H 3.174427 -1.244856 -2.229713
H 0.566086 1.380854 -0.488786
O 5.155679 -0.078342 -0.998791
O -1.313305 2.339658 0.612336
C -1.334946 3.779365 0.666343
H -0.559132 4.054351 1.378193
H -1.117003 4.190855 -0.320772
H -2.312078 4.122803 1.009174
33
2-H-3-COOMe-C-5 Eopt -
1240.836676
C 1.348803 -0.058908 -0.254051
C 2.692375 -0.042751 0.160103
C 3.486259 -1.181115 0.117159
C 2.956446 -2.407125 -0.360822
C 1.646859 -2.477833 -0.731395
C 0.705524 -1.340904 -0.631817
C 0.779728 1.231299 -0.226188
C 1.671454 2.182344 0.237662
H 4.525772 -1.138346 0.434489
H 3.600937 -3.276408 -0.412694
H 1.227473 -3.423298 -1.067750
H 0.218022 -1.191434 -1.607136
H 1.475510 3.242894 0.352079
S 3.206255 1.560890 0.617187
C -0.598278 1.530243 -0.698832
O -1.221791 0.780992 -1.420095
C -0.483670 -1.810550 0.363768
C -0.893416 -0.770373 1.362523
C -1.611614 -2.350895 -0.461095
C -2.155757 -0.344816 1.488588
H -0.121611 -0.399414 2.033824
C -2.870430 -1.911475 -0.352009
H -1.358870 -3.129641 -1.178422
C -3.230219 -0.843849 0.602364
H -2.441596 0.381597 2.244406
H -3.671521 -2.309612 -0.967808
H -0.065002 -2.639032 0.951954
O -4.370431 -0.399744 0.671805
O -1.042565 2.696652 -0.251617
C -2.369644 3.063131 -0.674977
H -3.087870 2.317514 -0.327996
H -2.564130 4.028884 -0.213532
H -2.405423 3.140312 -1.763024
33
2-H-3-COOMe-C-6 Eopt -
1240.837606
C -1.370544 -0.102124 0.209738
C -2.707748 -0.346196 -0.157530
C -3.283890 -1.604011 -0.053635
C -2.528949 -2.702331 0.432437
C -1.217468 -2.525931 0.753532
C -0.499673 -1.238575 0.602480
C -1.059427 1.272347 0.137114
C -2.131140 2.024473 -0.314103
H -4.324845 -1.753891 -0.329635
H -3.004911 -3.670636 0.528434
H -0.622238 -3.371835 1.091144
H -0.041586 -0.982695 1.569702
H -2.133080 3.100018 -0.457233
S -3.529319 1.118383 -0.628315
C 0.224289 1.934694 0.499992
O 0.504450 3.064500 0.171513
C 0.735538 -1.520070 -0.392575
C 1.043494 -0.418588 -1.360879
C 1.910968 -1.967718 0.420330
C 2.281786 0.053562 -1.552379
H 0.223231 -0.057127 -1.977025
C 3.144517 -1.476070 0.252911
H 1.721897 -2.735373 1.168959
C 3.428411 -0.438008 -0.758707
H 2.492624 0.810203 -2.302826
H 3.983555 -1.821964 0.849602
H 0.419632 -2.368769 -1.018080
O 4.561919 -0.006023 -0.934200
O 0.998548 1.146798 1.242674
C 2.276420 1.697316 1.610439
H 2.796256 0.901183 2.140163
H 2.826637 1.995040 0.714842
H 2.133091 2.562903 2.260103
33
2-H-3-COOMe-E-1 Eopt -
1240.872439
C -1.008604 -0.152004 -0.466436
C -2.054237 -0.238224 0.455862
C -3.270481 -0.826045 0.124278
C -3.431078 -1.328904 -1.167114
C -2.398656 -1.239688 -2.101837
C -1.184191 -0.646538 -1.755195
C 0.224291 0.550777 0.066191
C 0.062398 0.549151 1.614085
H -4.074528 -0.894416 0.850176
H -4.372011 -1.794418 -1.442933
H -2.538803 -1.629810 -3.104404
H -0.391465 -0.559741 -2.492735
H 0.578599 1.357758 2.135106
S -1.678421 0.499425 2.030907
O 0.707051 -0.711055 2.034950
C 1.442141 -1.181508 1.073396
C 1.534136 -0.255454 -0.070927
C 2.061847 -2.431257 1.093853
C 2.005164 -0.851722 -1.341885
H 2.367069 0.423505 0.226019
C 2.586102 -2.856931 -0.107963
H 2.043475 -3.055416 1.979076
C 2.547569 -2.090501 -1.325941
H 2.000264 -0.234771 -2.235871
H 3.032393 -3.846821 -0.148570
H 2.964716 -2.528114 -2.225276
C 0.325237 1.986715 -0.468802
O -0.582496 2.555108 -1.021318
O 1.517908 2.518549 -0.222210
C 1.698636 3.885652 -0.645333
H 0.986286 4.530000 -0.127602
H 2.720090 4.140398 -0.371407
H 1.557316 3.959123 -1.724849
33
2-H-3-COOMe-E-2 Eopt -
1240.850420
C 0.581329 0.662261 -0.458392
C 1.266236 1.446927 0.473906
C 1.832674 2.664918 0.112219
C 1.713280 3.087235 -1.212234
C 1.043379 2.305838 -2.153837
C 0.477538 1.086386 -1.779978
C 0.061121 -0.652290 0.104337
C 0.147844 -0.510342 1.649993
H 2.355112 3.272892 0.842928
H 2.148997 4.035436 -1.508806
H 0.961988 2.642377 -3.181277
H -0.026927 0.475816 -2.522392
H 0.310430 -1.441809 2.192949
S 1.368677 0.725271 2.092505
O -1.193063 -0.024125 2.035446
C -2.024387 -0.146432 1.049150
C -1.450132 -0.887214 -0.090307
C -3.314478 0.390271 1.037561

C -2.156614 -0.718500 -1.383257
H -1.644198 -1.954307 0.160183
C -3.946528 0.398478 -0.186028
H -3.737110 0.855233 1.919038
C -3.373644 -0.131145 -1.397608
H -1.727116 -1.183417 -2.264810
H -4.928839 0.857077 -0.251324
H -3.944842 -0.057784 -2.314917
C 0.866338 -1.827699 -0.445622
O 0.396442 -2.644475 -1.203393
O 2.115130 -1.805759 -0.014852
C 2.978106 -2.844753 -0.518979
H 3.037830 -2.774307 -1.605423
H 3.947008 -2.660232 -0.063171
H 2.588394 -3.820079 -0.226205
33
2-H-3-COOMe-TS1-1 Eopt -1240.818562
C -0.182925 -0.764012 0.900720
C -1.198231 -1.433265 0.186655
C -2.517433 -1.494544 0.619487
C -2.818649 -0.878410 1.838861
C -1.827396 -0.215619 2.559541
C -0.500620 -0.148678 2.103693
C 1.097148 -0.840416 0.178082
C 1.020637 -1.522730 -0.986893
H -3.282435 -2.000110 0.038259
H -3.833899 -0.915921 2.218215
H -2.082077 0.261036 3.500711
H 0.259407 0.368577 2.676496
H 1.800885 -1.719826 -1.711055
S -0.616890 -1.958845 -1.363712
O -0.995298 -0.238629 -2.378477
C -0.950155 0.820197 -1.606318
C 0.281300 1.514857 -1.417744
C -2.130209 1.258841 -0.942091
C 0.319910 2.612088 -0.572301
H 1.157565 1.182547 -1.966499
C -2.066561 2.348053 -0.098855
H -3.058342 0.728514 -1.131126
C -0.839412 3.006020 0.104926
H 1.247703 3.154650 -0.424779
H -2.957391 2.697582 0.412504
H -0.797735 3.851917 0.784572
C 2.364031 -0.231909 0.687457
O 2.439707 0.344652 1.748593
O 3.367109 -0.392209 -0.164352
C 4.632468 0.161845 0.243277
H 5.320305 -0.061547 -0.569331
H 4.537906 1.240082 0.384303
H 4.962798 -0.310409 1.170051
33
2-H-3-COOMe-TS1-2 Eopt -1240.817557
C -0.048599 -0.438452 1.053132
C -1.285924 -1.080574 0.833536
C -2.465637 -0.700978 1.461308
C -2.392336 0.355100 2.375635
C -1.179858 0.997951 2.611978
C 0.002226 0.613754 1.958327
C 1.001589 -1.044742 0.213871
C 0.555801 -2.064878 -0.554415
H -3.405263 -1.204138 1.254231
H -3.288176 0.672654 2.897944
H -1.141504 1.818204 3.321659
H 0.932373 1.129420 2.160669
H 1.117539 -2.662329 -1.262013
S -1.164572 -2.236247 -0.458596
O -1.527269 -0.942389 -1.988004
C -1.140962 0.279359 -1.713639
C 0.189386 0.693215 -2.021267
C -2.047028 1.183064 -1.089688
C 0.593341 1.979208 -1.698226
H 0.849344 -0.004239 -2.528706
C -1.621043 2.455047 -0.771323
H -3.061504 0.848749 -0.895755
C -0.297433 2.842983 -1.052434
H 1.599372 2.309661 -1.934623
H -2.301236 3.158520 -0.302759
H 0.030342 3.842732 -0.783589
C 2.446103 -0.654224 0.136100
O 3.231983 -1.204783 -0.599917
O 2.738512 0.356450 0.941374
C 4.103696 0.815988 0.902805
H 4.349253 1.159601 -0.103765
H 4.151940 1.637858 1.613997
H 4.774917 0.008113 1.198424
33
2-H-3-COOMe-TS2-1 Eopt -1240.827370
C -0.114418 0.076504 -0.998249
C -1.304920 0.822887 -1.088260
C -2.517747 0.250808 -1.500043
C -2.549266 -1.101898 -1.840526
C -1.401762 -1.874924 -1.712332
C -0.183338 -1.326424 -1.224196
C 0.977766 0.900470 -0.561061
C 0.594644 2.202099 -0.348859
H -3.418373 0.854981 -1.565926
H -3.474213 -1.549676 -2.185235
H -1.430035 -2.932923 -1.956037
H 0.729093 -1.906049 -1.309173
H 1.237530 3.012002 -0.023552
S -1.057708 2.489941 -0.660348
O -0.951251 1.416695 2.592495
C -0.837919 0.267352 2.194821
C 0.489796 -0.401315 2.122687
C -2.027995 -0.517019 1.773335
C 0.615262 -1.578895 1.477050
H 1.323765 0.099279 2.606673
C -1.870733 -1.701338 1.152029
H -3.002965 -0.096576 2.002179
C -0.538593 -2.170331 0.828658
H 1.571926 -2.083205 1.386618
H -2.721600 -2.293598 0.832978
H -0.436135 -3.182447 0.448873
C 2.394614 0.508051 -0.350012
O 3.228590 1.238220 0.138142
O 2.641559 -0.736554 -0.761330
C 3.986593 -1.207385 -0.566530
H 4.243949 -1.178219 0.494239
H 3.992783 -2.231014 -0.936481
H 4.684504 -0.590351 -1.135115
33
2-H-3-COOMe-TS2-2 Eopt -1240.828971
C -0.225502 0.316528 -0.972974
C -1.173038 1.309480 -0.667090
C -2.516168 1.202598 -1.056678
C -2.925842 0.078527 -1.775615
C -2.022002 -0.942645 -2.043793
C -0.674437 -0.884766 -1.588276
C 1.075519 0.673815 -0.487933
C 1.077409 1.891048 0.149103
H -3.223939 1.990924 -0.815689
H -3.954853 -0.002685 -2.106627
H -2.347255 -1.827378 -2.583392
H 0.052343 -1.596857 -1.965876
H 1.931165 2.386085 0.596201
S -0.455985 2.642350 0.188307
O -0.479431 0.599825 2.918381
C -0.691585 -0.362713 2.196296
C 0.410131 -1.256424 1.749756
C -2.067859 -0.685128 1.736237
C 0.190712 -2.156510 0.770048
H 1.372338 -1.140840 2.241127
C -2.258648 -1.605715 0.772550
H -2.886241 -0.156333 2.216043
C -1.113270 -2.228522 0.137837
H 0.979251 -2.802114 0.398937
H -3.250821 -1.856122 0.412485
H -1.299074 -3.062174 -0.532830
C 2.290149 -0.152532 -0.682756
O 2.298756 -1.196048 -1.303991
O 3.361625 0.379164 -0.101290
C 4.590238 -0.351054 -0.256702
H 4.844247 -0.435510 -1.314953
H 5.342182 0.230924 0.272409
H 4.493971 -1.344981 0.184380
33
2-H-3-COOMe-TS3-1 Eopt -1240.824367
C -0.228091 0.010839 -0.966369
C -1.446119 0.714092 -1.056726
C -2.618680 0.128116 -1.528549
C -2.550559 -1.200135 -1.946480
C -1.342952 -1.902777 -1.898439
C -0.174861 -1.313199 -1.407635
C 0.785930 0.823862 -0.288627
C 0.331705 2.135728 -0.081517
H -3.549156 0.685151 -1.575608
H -3.443854 -1.685963 -2.324294
H -1.309438 -2.929605 -2.248204
H 0.754283 -1.869048 -1.379263
H 0.922246 2.950109 0.325284
S -1.276899 2.360295 -0.481824
O -1.427444 1.345831 2.166668
C -0.961619 0.237674 1.924868
C 0.526681 0.070506 1.809357
C -1.808926 -0.941883 1.719655
C 1.060161 -1.236036 1.701148
H 1.126767 0.883720 2.210795
C -1.235329 -2.146744 1.517236
H -2.883527 -0.794386 1.754688
C 0.208019 -2.294739 1.517703
H 2.133120 -1.391152 1.753445
H -1.842182 -3.034337 1.370229
H 0.616816 -3.296729 1.422765
C 2.270894 0.601632 -0.292296
O 3.043722 1.318075 0.302128
O 2.610973 -0.460561 -1.005837
C 4.021268 -0.754326 -1.064064
H 4.409297 -0.918563 -0.057203
H 4.102418 -1.659026 -1.662665
H 4.550456 0.072686 -1.540034
33
2-H-3-COOMe-TS3-2 Eopt -1240.824963

C 0.433444 -0.306236 -0.950034
C 1.436489 -1.212234 -0.555876
C 2.773469 -1.048850 -0.910659
C 3.095194 0.046794 -1.711151
C 2.104417 0.932831 -2.145595
C 0.767892 0.772577 -1.769949
C -0.840693 -0.632214 -0.307908
C -0.770105 -1.861092 0.369883
H 3.534775 -1.749993 -0.584146
H 4.126367 0.201328 -2.010532
H 2.377429 1.764777 -2.786883
H 0.003888 1.459398 -2.114050
H -1.595234 -2.367322 0.859073
S 0.774042 -2.504763 0.423829
O 0.784771 -0.701007 2.609714
C 0.638440 0.326809 1.957138
C -0.733045 0.708067 1.477313
C 1.762332 1.200601 1.604739
C -0.919652 1.980789 0.884479
H -1.560214 0.199356 1.965581
C 1.527411 2.352337 0.941219
H 2.754196 0.884546 1.911850
C 0.176577 2.748839 0.588843
H -1.920247 2.334759 0.657973
H 2.343093 3.017390 0.676693
H 0.035345 3.719666 0.122303
C -2.153252 -0.096455 -0.791935
O -2.253635 0.641959 -1.743653
O -3.163784 -0.517415 -0.041739
C -4.473696 -0.061641 -0.434412
H -4.698776 -0.407854 -1.444368
H -5.159621 -0.501939 0.286048
H -4.515833 1.028262 -0.391945
40
2-Me-3-Me-A1-1 Eopt -1617.861156
C -0.689884 2.283861 0.084683
C -0.463854 1.254640 -0.833993
C -0.909426 1.267036 -2.139506
C -1.639159 2.391851 -2.545176
C -1.890810 3.432934 -1.652574
C -1.421075 3.390188 -0.334665
C -0.107701 2.028143 1.418984
C 0.530444 0.849576 1.512210
H -0.716941 0.439269 -2.813253
H -2.015671 2.444564 -3.561260
H -2.463542 4.293362 -1.983999
H -1.627040 4.207944 0.349285
S 0.514328 -0.021211 -0.073481
O 1.987815 0.604897 -0.682993
C 3.082652 -0.239122 -0.524872
C 3.115575 -1.491250 -1.135456
C 4.159175 0.238275 0.218323
C 4.256114 -2.280109 -0.989552
H 2.267690 -1.837897 -1.719657
C 5.298048 -0.555659 0.344809
H 4.093583 1.219114 0.679505
C 5.347006 -1.815621 -0.253603
H 4.292014 -3.257086 -1.461349
H 6.145169 -0.189532 0.916594
H 6.233490 -2.433037 -0.149032
C -2.047846 -1.467658 -0.173887
O -1.487282 -0.769199 0.721048
O -1.693624 -1.699769 -1.326853
C -3.384732 -2.088429 0.319021
F -3.975052 -2.850742 -0.608378
F -3.193947 -2.858467 1.406783
F -4.263501 -1.128797 0.666002
C 1.194592 0.140010 2.641203
H 0.665470 -0.792690 2.865619
H 2.233126 -0.111090 2.399755
H 1.190423 0.764340 3.536068
C -0.278379 3.024105 2.521029
H 0.119676 3.996135 2.211108
H -1.343196 3.158023 2.740137
H 0.229300 2.715955 3.435027
40
2-Me-3-Me-A1-2 Eopt -1617.863161
C 0.572505 0.953444 0.787716
C 0.571864 1.081393 -0.603008
C 0.876541 2.249108 -1.273985
C 1.215804 3.357521 -0.487965
C 1.230193 3.259582 0.902990
C 0.904553 2.063025 1.553790
C 0.176930 -0.393928 1.250024
C -0.071617 -1.256978 0.248761
H 0.857909 2.308291 -2.357822
H 1.462337 4.298030 -0.969131
H 1.490926 4.131156 1.495068
H 0.903781 2.007608 2.638044
S 0.178415 -0.479679 -1.346337
O 1.701485 -1.051468 -1.780080
C 2.790645 -0.911235 -0.915773
C 3.628912 0.188745 -1.075463
C 3.044122 -1.896854 0.033277
C 4.742753 0.309940 -0.246501
H 3.408563 0.927039 -1.839727
C 4.162745 -1.763272 0.855496
H 2.381711 -2.752530 0.118182
C 5.006366 -0.659938 0.722055
H 5.404858 1.162346 -0.361001
H 4.373295 -2.525797 1.598672
H 5.873706 -0.560367 1.367011
C -2.856380 0.322064 0.001917
O -2.216117 0.114580 -1.059900
O -2.507080 0.808527 1.079486
C -4.333675 -0.169051 -0.093234
F -5.090690 0.215078 0.944950
F -4.945327 0.271839 -1.208733
F -4.383844 -1.520056 -0.132804
C -0.558390 -2.665592 0.229195
H -0.532474 -3.085962 1.236036
H -1.588919 -2.709177 -0.140724
H 0.061157 -3.293323 -0.420628
C 0.052209 -0.682879 2.707429
H -0.710620 -0.026150 3.139029
H -0.226554 -1.718993 2.899195
H 1.001650 -0.472730 3.212105
40
2-Me-3-Me-A1-3 Eopt -1617.865301
C 0.656231 0.533471 1.204842
C 0.185617 0.813954 -0.081630
C -0.008884 2.089476 -0.571890
C 0.299005 3.150683 0.289165
C 0.771934 2.904090 1.577671
C 0.950465 1.598065 2.049375
C 0.766005 -0.912882 1.488689
C 0.404784 -1.692341 0.455284
H -0.388238 2.259373 -1.573714
H 0.161398 4.171235 -0.052343
H 1.001738 3.740367 2.230379
H 1.310737 1.419887 3.058006
S -0.035881 -0.702832 -0.982664
O 1.444146 -0.771602 -1.828700
C 2.647929 -0.401974 -1.236309
C 3.110535 0.899928 -1.416615
C 3.391469 -1.347375 -0.533418
C 4.336632 1.264614 -0.863384
H 2.514147 1.606477 -1.985045
C 4.617116 -0.970196 0.014722
H 3.015826 -2.360083 -0.423938
C 5.087225 0.334235 -0.142508
H 4.704842 2.276778 -0.998779
H 5.204557 -1.700493 0.562597
H 6.040622 0.623000 0.288673
C -2.905769 0.020708 -0.601880
O -2.124150 -0.724311 0.053522
O -2.677268 0.752601 -1.563538
C -4.364337 -0.038341 -0.065467
F -4.423752 0.327140 1.229475
F -5.201263 0.759018 -0.740337
F -4.858820 -1.289353 -0.141825
C 0.331462 -3.169641 0.280279
H 0.785459 -3.676066 1.134085
H -0.711408 -3.493697 0.193789
H 0.859165 -3.486339 -0.626593
C 1.249007 -1.388638 2.819702
H 0.610347 -0.990476 3.615123
H 1.253051 -2.476475 2.888526
H 2.265974 -1.019961 2.995986
33
2-Me-3-Me-A2-1 Eopt -1091.644055
C 1.510778 0.052421 0.614555
C 1.355951 -0.289607 -0.733825
C 1.802996 -1.472987 -1.291023
C 2.424246 -2.379728 -0.422541
C 2.587266 -2.069241 0.925983
C 2.143682 -0.849372 1.456984
C 0.973608 1.394767 0.940729
C 0.417079 2.027186 -0.113117
H 1.674363 -1.696413 -2.345498
H 2.784977 -3.326451 -0.809491
H 3.076269 -2.783096 1.580923
H 2.295567 -0.617707 2.506588
S 0.512412 1.011444 -1.581412
O -1.035870 0.454939 -1.808715
C -1.669541 -0.234597 -0.757968
C -2.379517 0.483021 0.198161
C -1.605646 -1.624288 -0.755159
C -3.039160 -0.226311 1.201667
H -2.429676 1.565947 0.150242
C -2.271127 -2.316981 0.254342
H -1.057830 -2.144222 -1.534428
C -2.979533 -1.619756 1.234299
H -3.602763 0.314694 1.954886
H -2.236971 -3.401500 0.270790
H -3.494078 -2.164067 2.019720
C -0.210046 3.373118 -0.270836
H -0.441192 3.791760 0.710067
H 0.463609 4.058634 -0.794950
H -1.142473 3.310907 -0.841679
C 1.080822 1.935604 2.325443
H 0.550476 1.270819 3.016391
H 2.131311 1.959181 2.633859
H 0.665638 2.939417 2.408728

33
2-Me-3-Me-A2-2 Eopt -1091.638730
C -2.532114 -0.025767 -0.059634
C -1.423247 -0.819784 0.252847
C -1.432816 -2.201541 0.258236
C -2.637755 -2.820846 -0.096970
C -3.760664 -2.057269 -0.409602
C -3.723255 -0.656027 -0.387977
C -2.252714 1.427957 0.011945
C -0.985293 1.721331 0.364455
H -0.554311 -2.783583 0.518071
H -2.693246 -3.903671 -0.119571
H -4.687301 -2.557450 -0.672088
H -4.609695 -0.076581 -0.625925
S -0.064971 0.220945 0.694038
O 0.922162 0.132986 -0.622405
C 2.290499 -0.097078 -0.356411
C 2.698656 -1.293532 0.217138
C 3.180055 0.891572 -0.751822
C 4.064433 -1.500657 0.405918
H 1.972137 -2.050473 0.499771
C 4.543387 0.661618 -0.568783
H 2.809065 1.807972 -1.199340
C 4.984153 -0.527677 0.012280
H 4.406706 -2.428137 0.853172
H 5.258257 1.417541 -0.877052
H 6.045980 -0.698340 0.157428
C -0.237805 3.000214 0.531746
H -0.883495 3.841942 0.278115
H 0.110465 3.125840 1.561431
H 0.632569 3.028184 -0.132577
C -3.326121 2.416456 -0.295372
H -3.686069 2.259035 -1.317733
H -4.174297 2.259980 0.379592
H -2.980934 3.444923 -0.196624
33
2-Me-3-Me-C-1 Eopt -1091.643512
C 0.984101 0.273762 0.345852
C 2.058434 1.002381 -0.235489
C 2.071339 2.373426 -0.292478
C 0.978483 3.120634 0.255975
C -0.104789 2.476677 0.751329
C -0.281945 0.996364 0.663999
C 1.285867 -1.077252 0.531204
C 2.564821 -1.388403 0.039172
H 2.925086 2.905687 -0.704689
H 1.044752 4.202046 0.279020
H -0.921638 3.053877 1.172271
H -0.725291 0.605928 1.588254
S 3.394691 -0.050549 -0.623332
C -1.389472 0.753436 -0.487710
C -1.504657 -0.691818 -0.865519
C -2.693145 1.360924 -0.058899
C -2.634391 -1.399239 -0.740721
H -0.621297 -1.161900 -1.290133
C -3.820992 0.653579 0.084089
H -2.709385 2.429639 0.134589
C -3.867034 -0.794440 -0.199569
H -2.689098 -2.443159 -1.036390
H -4.749434 1.120440 0.399992
H -1.013536 1.300787 -1.364569
O -4.882655 -1.455353 -0.015947
C 3.188351 -2.737482 0.090029
H 3.294690 -3.046489 1.136625
H 2.529707 -3.463505 -0.399022
H 4.168237 -2.764418 -0.387767
C 0.428253 -2.111585 1.196155
H -0.447339 -1.659588 1.665670
H 0.078843 -2.858440 0.475788
H 1.002542 -2.635093 1.966731
33
2-Me-3-Me-C2 Eopt -1091.648785
C -0.847129 -0.331907 0.601021
C -1.742172 -0.615011 -0.470121
C -2.091345 -1.893888 -0.823345
C -1.524333 -3.013021 -0.127158
C -0.580244 -2.807569 0.820755
C -0.049613 -1.457744 1.166897
C -0.810664 1.023951 0.931726
C -1.616386 1.781284 0.061855
H -2.814371 -2.072020 -1.615202
H -1.853491 -4.012787 -0.385393
H -0.112367 -3.651333 1.321244
H 0.045701 -1.364144 2.256501
S -2.437417 0.850910 -1.110633
C 1.452389 -1.408396 0.605174
C 1.467557 -1.361739 -0.891910
C 2.238315 -0.315687 1.256373
C 2.104725 -0.410490 -1.586664
H 0.930627 -2.149532 -1.416718
C 2.892125 0.626958 0.566047
H 2.277474 -0.330456 2.343736
C 2.850106 0.670003 -0.909434
H 2.102998 -0.398001 -2.672898
H 3.472962 1.399685 1.061826
H 1.893293 -2.362770 0.929125
O 3.410182 1.558435 -1.542345
C -1.791625 3.255652 0.135908
H -2.272955 3.514747 1.086627
H -0.809965 3.741142 0.123067
H -2.396301 3.646226 -0.683369
C -0.108553 1.665569 2.092127
H 0.276995 0.915456 2.784966
H 0.726593 2.289130 1.760818
H -0.808874 2.304829 2.639065
33
2-Me-3-Me-C3 Eopt -1091.650317
C -1.078459 0.051014 -0.531517
C -1.601405 1.275930 -0.040870
C -0.960673 2.477217 -0.224603
C 0.284627 2.528468 -0.930911
C 0.857676 1.386237 -1.381191
C 0.271175 0.035243 -1.161101
C -1.933458 -1.033855 -0.318032
C -3.095000 -0.640001 0.362489
H -1.395627 3.399373 0.153251
H 0.759121 3.490680 -1.084510
H 1.817518 1.423671 -1.890730
H 0.205850 -0.483598 -2.131080
S -3.149170 1.032141 0.722656
C 1.281602 -0.828796 -0.295885
C 2.536953 -1.097655 -1.065119
C 1.505871 -0.208826 1.049480
C 3.758640 -0.845818 -0.579020
H 2.416134 -1.533116 -2.055502
C 2.725439 0.048606 1.537993
H 0.619711 0.021427 1.639526
C 3.946129 -0.249675 0.760328
H 4.658064 -1.067977 -1.146100
H 2.867825 0.487748 2.521127
H 0.775850 -1.793472 -0.143619
O 5.063260 -0.010438 1.204530
C -4.213547 -1.542686 0.748531
H -4.685321 -1.945402 -0.155340
H -3.821049 -2.389547 1.321040
H -4.973312 -1.032919 1.342303
C -1.694764 -2.448237 -0.756491
H -0.970971 -2.486386 -1.575079
H -1.313705 -3.059908 0.068494
H -2.624295 -2.904324 -1.106989
33
2-Me-3-Me-D-1 Eopt -1091.662337
C 0.812238 -0.589758 0.434759
C 1.981628 -0.298346 -0.268269
C 2.887061 -1.267797 -0.684762
C 2.586807 -2.592768 -0.375800
C 1.425136 -2.912248 0.333006
C 0.537537 -1.918873 0.748273
C 0.006516 0.629876 0.822287
C 0.674347 1.798839 0.142700
H 3.786736 -1.007219 -1.232963
H 3.263610 -3.380496 -0.689483
H 1.208939 -3.949781 0.566159
H -0.355117 -2.182063 1.307610
S 2.140993 1.442082 -0.550775
O -0.761171 0.986973 -1.796626
C -1.593913 0.415507 -1.099065
C -1.492461 0.550640 0.415606
C -2.689903 -0.371765 -1.650743
C -2.330540 -0.408036 1.206150
H -1.929756 1.544705 0.626253
C -3.457333 -1.125490 -0.832512
H -2.807101 -0.380475 -2.729409
C -3.271051 -1.159232 0.613237
H -2.213244 -0.417141 2.286009
H -4.239744 -1.747585 -1.258770
H -3.914118 -1.804113 1.202839
C 0.177308 3.190148 0.214137
H -0.023592 3.450802 1.259839
H 0.881161 3.904533 -0.213339
H -0.771885 3.257547 -0.330282
C 0.147040 0.883745 2.352715
H -0.137066 -0.026820 2.883048
H 1.183276 1.116851 2.611109
H -0.501122 1.705503 2.666469
33
2-Me-3-Me-E-1 Eopt -1091.684157
C 0.919398 -0.542915 0.442879
C 1.906774 -0.021435 -0.398488
C 2.924811 -0.818017 -0.914808
C 2.951776 -2.168605 -0.567055
C 1.982452 -2.702646 0.282933
C 0.968155 -1.890328 0.793408
C -0.080942 0.485869 0.952037
C 0.066642 1.712614 -0.006101
H 3.679458 -0.399891 -1.573611
H 3.736322 -2.805622 -0.963251
H 2.015498 -3.753458 0.551372
H 0.229303 -2.312293 1.469065
S 1.738908 1.720452 -0.687558
O -0.859033 1.369915 -1.132907
C -1.680630 0.437595 -0.771053

C -1.548539 0.088888 0.655008
C -2.570052 -0.205386 -1.639240
C -2.148898 -1.205847 1.049969
H -2.190800 0.840455 1.170270
C -3.170428 -1.341141 -1.145349
H -2.694296 0.124908 -2.663663
C -2.955824 -1.857526 0.182228
H -2.003377 -1.552396 2.069050
H -3.827427 -1.901838 -1.804823
H -3.458178 -2.774719 0.467253
C -0.353528 3.071155 0.508413
H -1.385423 3.039704 0.871490
H 0.302215 3.381884 1.323941
H -0.290455 3.807349 -0.296707
C 0.163913 0.817102 2.421600
H 1.183213 1.188526 2.562785
H -0.546157 1.566091 2.783175
H 0.049351 -0.091528 3.019545
33
2-Me-3-Me-TS1-1 Eopt -1091.630303
C -0.105771 -0.784088 1.007033
C -0.103035 -1.554681 -0.169758
C -1.227806 -2.222600 -0.634644
C -2.393142 -2.132627 0.138910
C -2.410343 -1.383044 1.312853
C -1.267387 -0.699246 1.759309
C 1.190339 -0.130684 1.248241
C 2.115312 -0.424677 0.282458
H -1.210022 -2.794500 -1.557280
H -3.287939 -2.652498 -0.186158
H -3.324100 -1.325593 1.895666
H -1.294807 -0.115907 2.674674
S 1.416135 -1.367596 -0.993437
O 0.791353 0.367470 -2.117213
C -0.041705 1.061696 -1.417133
C 0.440663 2.030569 -0.475908
C -1.451204 0.836353 -1.544594
C -0.463743 2.735380 0.304414
H 1.509329 2.221403 -0.433284
C -2.325680 1.536517 -0.748683
H -1.790372 0.119059 -2.285362
C -1.830541 2.465966 0.192071
H -0.112921 3.483164 1.007693
H -3.395703 1.378115 -0.834837
H -2.532707 3.004895 0.821500
C 3.521028 0.056604 0.118699
H 3.850192 0.573750 1.020919
H 4.205309 -0.776585 -0.063565
H 3.597024 0.749720 -0.726725
C 1.421182 0.680615 2.475832
H 0.590744 1.380748 2.619247
H 1.444015 0.020933 3.351340
H 2.353958 1.242758 2.434275
33
2-Me-3-Me-TS2-1 Eopt -1091.630551
C -0.036721 -0.666110 0.925983
C -0.033067 -1.513441 -0.205557
C -1.170521 -2.201235 -0.617186
C -2.362186 -2.052713 0.115780
C -2.412182 -1.187551 1.192534
C -1.272441 -0.417058 1.577830
C 1.238273 -0.072952 1.169801
C 2.174555 -0.469331 0.227895
H -1.140928 -2.856539 -1.483583
H -3.244459 -2.608158 -0.181817
H -3.338523 -1.056825 1.744024
H -1.288999 0.101791 2.532365
S 1.540039 -1.562536 -0.941214
O 0.810956 1.298962 -2.315686
C -0.089473 1.480800 -1.505997
C -1.450868 0.944340 -1.742980
C 0.136205 2.251210 -0.256250
C -2.368659 0.974526 -0.757076
H -1.658536 0.538555 -2.728779
C -0.811279 2.268862 0.708979
H 1.069818 2.800534 -0.174784
C -2.003002 1.474507 0.552556
H -3.367091 0.575298 -0.899956
H -0.665362 2.813389 1.636258
H -2.791054 1.586088 1.291340
C 3.603005 -0.037888 0.171328
H 4.168290 -0.590423 -0.581176
H 3.659607 1.029170 -0.072744
H 4.078955 -0.185417 1.145842
C 1.583807 0.794101 2.342536
H 2.113126 0.207802 3.102409
H 2.235139 1.621151 2.046410
H 0.689075 1.212048 2.808031
42
A_1 Eopt -1731.150197
C -0.518809 -0.165586 1.140068
C 0.043061 -0.995312 0.160863
C 0.367582 -2.322400 0.361344
C 0.107905 -2.853816 1.629679
C -0.439333 -2.052651 2.630459
C -0.750478 -0.707867 2.401441
C -0.730635 1.214919 0.655910
C -0.333771 1.432260 -0.610048
H 0.811212 -2.920295 -0.427426
H 0.346245 -3.892315 1.833379
H -0.621626 -2.475346 3.613327
H -1.156866 -0.103966 3.205042
S 0.234883 -0.109658 -1.366873
O -1.224374 -0.536545 -2.147692
C -2.444333 -0.607780 -1.481101
C -3.271906 0.512980 -1.448803
C -2.841400 -1.820562 -0.921932
C -4.518053 0.415904 -0.830714
H -2.945612 1.440440 -1.910089
C -4.087162 -1.902950 -0.301403
H -2.182810 -2.681162 -0.981358
C -4.924002 -0.786915 -0.250800
H -5.170449 1.283372 -0.804504
H -4.404395 -2.843971 0.137009
H -5.893447 -0.856708 0.232458
C -1.421832 2.298580 1.434929
O -1.017214 3.443694 1.375402
C -2.649249 1.926440 2.224223
H -3.092297 0.986470 1.887168
H -2.374993 1.828659 3.280734
H -3.373736 2.739611 2.139671
C 3.065804 -0.295964 -0.596144
O 2.177902 0.594208 -0.436256
O 2.976702 -1.406848 -1.110167
C 4.438884 0.130532 -0.007838
F 4.369684 0.225711 1.334309
F 5.414478 -0.738646 -0.294626
F 4.824780 1.333057 -0.468626
C -0.340983 2.630579 -1.488433
H -0.977188 3.409468 -1.066259
H 0.677919 3.025027 -1.575408
H -0.692423 2.375710 -2.493760
42
A_10 Eopt -1731.129198
C 1.121348 -1.002527 -0.351836
C 1.163122 -0.136215 -1.456104
C 0.048989 0.468577 -2.011555
C -1.181464 0.207566 -1.397503
C -1.256149 -0.647801 -0.300463
C -0.112547 -1.270680 0.223059
C 2.456638 -1.547063 -0.021034
C 3.456581 -1.120591 -0.814572
H 0.124666 1.122928 -2.874730
H -2.096772 0.654648 -1.770390
H -2.230818 -0.840163 0.145102
H -0.207044 -1.959180 1.055686
S 2.815664 0.039371 -2.035113
O 3.348619 1.507046 -1.475259
C 3.016129 1.891827 -0.161174
C 3.882999 1.564470 0.875449
C 1.858048 2.636043 0.035701
C 3.567030 1.998826 2.162260
H 4.788679 0.999924 0.676528
C 1.555202 3.057181 1.329318
H 1.219379 2.885041 -0.805471
C 2.403696 2.735130 2.389582
H 4.233319 1.760343 2.984954
H 0.655914 3.638495 1.504551
H 2.161116 3.065033 3.394655
C 2.744068 -2.462744 1.143379
O 3.485858 -3.410101 0.982624
C 2.133297 -2.122173 2.473578
H 1.807314 -1.081309 2.526667
H 1.266999 -2.771595 2.643077
H 2.867502 -2.332786 3.254318
C -4.812789 -0.245111 0.056594
O -4.284580 0.356372 -0.896518
O -4.333536 -1.007720 0.912538
C -6.347759 0.023991 0.171900
F -7.002184 -0.367874 -0.943601
F -6.938641 -0.603824 1.202183
F -6.608368 1.341388 0.323813
C 4.919216 -1.400593 -0.849920
H 5.246757 -1.787562 0.116426
H 5.142423 -2.149990 -1.616337
H 5.487869 -0.494435 -1.081800
42
A_11 Eopt -1731.132547
C -0.692596 -0.068998 -0.999026
C -1.696663 -0.878279 -1.555995
C -1.503479 -2.184458 -1.967826
C -0.224157 -2.720704 -1.778704
C 0.791305 -1.941042 -1.230714
C 0.577903 -0.607145 -0.848852
C -1.179251 1.299634 -0.714417
C -2.467495 1.522885 -1.034005
H -2.305644 -2.770200 -2.405989
H -0.026228 -3.745342 -2.074200
H 1.789857 -2.344108 -1.095061
H 1.415571 -0.029735 -0.462480
S -3.207057 0.016330 -1.684725
O -4.161471 -0.506695 -0.432166
C -3.574888 -0.687915 0.836038

C -3.571469 0.373477 1.734163
C -3.073686 -1.944375 1.158248
C -3.034828 0.162660 3.003768
H -3.992078 1.333897 1.453355
C -2.537260 -2.135943 2.430417
H -3.114439 -2.750053 0.432356
C -2.512446 -1.084653 3.347860
H -3.026637 0.976644 3.721468
H -2.140758 -3.108774 2.701893
H -2.090591 -1.239578 4.335723
C -0.367383 2.383095 -0.044229
O -0.437066 3.518398 -0.470482
C 0.442031 1.997396 1.155943
H -0.003222 1.150451 1.685741
H 1.450170 1.704651 0.830144
H 0.528241 2.863491 1.814962
C 4.034091 -0.129605 0.040717
O 3.680325 -1.295839 -0.204003
O 3.377559 0.923686 0.131209
C 5.572296 0.010124 0.270653
F 6.271118 -0.411547 -0.805375
F 5.976170 1.265624 0.522928
F 5.983402 -0.744361 1.313319
C -3.349865 2.718930 -0.927951
H -2.951991 3.407296 -0.180298
H -3.395614 3.241836 -1.888712
H -4.367679 2.433906 -0.644136
42
A_12 Eopt -
1731.134316
C -2.666089 -0.802629 0.324468
C -3.086357 0.122622 -0.640315
C -4.175933 0.959028 -0.481604
C -4.867257 0.868037 0.733362
C -4.466376 -0.040461 1.710578
C -3.369586 -0.892082 1.515759
C -1.489401 -1.580198 -0.113126
C -1.026671 -1.276144 -1.335845
H -4.481205 1.658983 -1.253175
H -5.724654 1.508968 0.907066
H -5.019120 -0.098096 2.642542
H -3.082274 -1.607042 2.280284
S -2.020482 0.052108 -2.047502
O -1.041865 1.382797 -1.926478
C -0.575745 1.763848 -0.650798
C 0.575056 1.176233 -0.140708
C -1.276703 2.761231 0.019464
C 1.029327 1.606850 1.106943
H 1.119120 0.413290 -0.693310
C -0.804377 3.180011 1.261313
H -2.160778 3.200254 -0.431931
C 0.341602 2.598103 1.807275
H 1.925848 1.163253 1.530986
H -1.331836 3.961098 1.799187
H 0.704169 2.924088 2.776942
C -0.917424 -2.656742 0.787277
O -1.588951 -3.647669 0.987524
C 0.407112 -2.371083 1.410838
H 0.310523 -1.468426 2.028756
H 0.726074 -3.212678 2.027103
H 1.158572 -2.142317 0.642923
C 3.491687 -0.897840 0.248711
O 3.697419 -1.447798 1.340367
O 2.707069 -1.177308 -0.680728
C 4.405037 0.337270 -0.044828
F 5.528951 -0.053723 -0.690651
F 3.814547 1.258235 -0.829631
F 4.806210 0.980804 1.066236
C 0.069670 -1.822411 -2.183073
H 0.682800 -2.511571 -1.600409
H -0.348443 -2.349260 -3.047083
H 0.726727 -1.024143 -2.543062
42
A_13 Eopt -
1731.136463
C -2.025942 -1.399058 0.258126
C -2.814868 -0.738816 -0.695410
C -4.177545 -0.538462 -0.575846
C -4.784418 -1.010455 0.593960
C -4.028083 -1.668752 1.560272
C -2.651383 -1.880617 1.400545
C -0.611884 -1.517303 -0.168336
C -0.327549 -0.946538 -1.352479
H -4.751172 -0.034014 -1.347435
H -5.849764 -0.868633 0.738879
H -4.514638 -2.038501 2.456995
H -2.095268 -2.416826 2.161194
S -1.819707 -0.221653 -2.057366
O -1.650479 1.412884 -1.883813
C -1.514205 1.947552 -0.583737
C -0.263209 1.971543 0.015375
C -2.653910 2.473429 0.016992
C -0.159432 2.545830 1.283333
H 0.618069 1.566394 -0.475509
C -2.527807 3.047582 1.279773
H -3.607034 2.437756 -0.501096
C -1.283454 3.078090 1.914191
H 0.810696 2.572663 1.769953
H -3.401612 3.469248 1.766060
H -1.191181 3.522023 2.900469
C 0.442361 -2.250723 0.632594
O 1.122220 -3.087897 0.077177
C 0.563884 -1.935680 2.095954
H 0.106537 -0.977037 2.348455
H 0.079608 -2.735303 2.668217
H 1.624590 -1.917320 2.348893
C 3.012295 0.587416 -0.084300
O 2.169725 -0.075046 0.550112
O 2.877415 1.502944 -0.913025
C 4.482485 0.131633 0.175229
F 4.739031 -1.018479 -0.492150
F 5.405415 1.021380 -0.225924
F 4.726910 -0.118984 1.476162
C 0.919271 -0.830832 -2.154456
H 1.722063 -1.359588 -1.640848
H 0.777106 -1.267012 -3.148654
H 1.214408 0.219229 -2.264794
42
A_2 Eopt -
1731.149531
C -0.525650 -0.006822 1.154994
C -0.008058 -0.958372 0.268813
C 0.243136 -2.271890 0.612387
C -0.051718 -2.649453 1.928032
C -0.571556 -1.723612 2.831869
C -0.809978 -0.396130 2.459348
C -0.700808 1.317916 0.527928
C -0.330003 1.383724 -0.759130
H 0.656348 -2.975320 -0.102476
H 0.133100 -3.670939 2.243188
H -0.790473 -2.034773 3.848297
H -1.200844 0.317779 3.176939
S 0.192653 -0.247746 -1.348935
O -1.275684 -0.736373 -2.072961
C -2.482722 -0.769608 -1.382323
C -2.874514 -1.957861 -0.768555
C -3.303315 0.355768 -1.378008
C -4.106938 -2.009073 -0.120176
H -2.220291 -2.823015 -0.807681
C -4.536776 0.290216 -0.730209
H -2.979758 1.262020 -1.881624
C -4.936299 -0.886179 -0.094997
H -4.419609 -2.929994 0.362051
H -5.185093 1.161325 -0.725363
H -5.895522 -0.930990 0.410975
C -1.209634 2.481375 1.341620
O -0.646126 2.756463 2.381854
C -2.456265 3.172514 0.875663
H -2.446209 3.356151 -0.201242
H -3.296150 2.495719 1.080127
H -2.597830 4.102678 1.427518
C 3.025758 -0.417536 -0.576824
O 2.166263 0.512523 -0.529329
O 2.895183 -1.587047 -0.925343
C 4.425467 0.052198 -0.092848
F 4.387283 0.415833 1.202865
F 5.358227 -0.899442 -0.211499
F 4.852720 1.120959 -0.789549
C -0.242278 2.493445 -1.746202
H -0.479347 3.449164 -1.276150
H 0.776677 2.552602 -2.142868
H -0.925694 2.327133 -2.587155
42
A_3 Eopt -
1731.149749
C -0.553199 0.145134 1.110594
C -0.084210 -0.908358 0.315195
C 0.108631 -2.194817 0.777440
C -0.201230 -2.439161 2.120947
C -0.689139 -1.415995 2.932343
C -0.868745 -0.118541 2.439569
C -0.668488 1.410162 0.359441
C -0.340613 1.325746 -0.937533
H 0.483900 -2.979054 0.128639
H -0.062662 -3.435469 2.527170
H -0.934164 -1.626416 3.968433
H -1.254511 0.664371 3.085682
S 0.107621 -0.371920 -1.370063
O -1.384997 -0.875016 -2.019205
C -2.568085 -0.838482 -1.283659
C -3.347173 0.315328 -1.280396
C -2.971017 -1.992899 -0.615160
C -4.547964 0.314801 -0.570769
H -3.019179 1.195844 -1.823683
C -4.172638 -1.980255 0.089798
H -2.348139 -2.880869 -0.655857
C -4.958008 -0.826199 0.119124
H -5.160533 1.210894 -0.559384
H -4.494394 -2.874221 0.614926
H -5.891478 -0.818923 0.673009
C -1.070924 2.723183 0.984320
O -2.055812 3.307362 0.579442
C -0.158376 3.280680 2.037262
H 0.731651 3.667678 1.524605
H -0.652545 4.094088 2.569962
H 0.184435 2.510372 2.731839
C 2.958439 -0.572500 -0.584426
O 2.145829 0.396959 -0.630513

O 2.780447 -1.759349 -0.845425
C 4.366343 -0.137397 -0.090950
F 4.312048 0.285474 1.187792
F 5.263375 -1.128485 -0.142673
F 4.853561 0.882109 -0.820924
C -0.277893 2.333679 -2.027914
H -0.634483 3.300012 -1.667079
H 0.752150 2.439006 -2.384808
H -0.900830 2.027224 -2.875951
42
A_4 Eopt -
1731.148433
C -0.575371 -0.648162 0.922942
C -0.614616 -1.375877 -0.272059
C -0.983160 -2.703791 -0.360913
C -1.352555 -3.340033 0.829604
C -1.348518 -2.635679 2.031887
C -0.961443 -1.291300 2.092988
C -0.120776 0.743650 0.717146
C 0.127033 1.070003 -0.563013
H -0.991852 -3.230708 -1.309972
H -1.645785 -4.384076 0.810388
H -1.646979 -3.139395 2.945732
H -0.965634 -0.770502 3.043841
S -0.225371 -0.330374 -1.647719
O -1.754645 0.081139 -2.210969
C -2.812501 0.330763 -1.329320
C -3.686162 -0.712007 -1.034519
C -2.999980 1.613805 -0.825409
C -4.768509 -0.462124 -0.192963
H -3.517758 -1.694057 -1.464492
C -4.087360 1.849193 0.015846
H -2.311873 2.410749 -1.088805
C -4.965513 0.813971 0.337557
H -5.457524 -1.266240 0.045117
H -4.244385 2.845221 0.417472
H -5.807815 1.003368 0.995434
C 0.130277 1.727482 1.829868
O -0.229231 2.881923 1.697828
C 0.848401 1.243239 3.059242
H 1.525214 2.032760 3.394484
H 0.112357 1.075032 3.853757
H 1.400895 0.321466 2.869438
C 2.790874 -0.597067 -0.142656
O 2.149528 -0.700838 -1.220618
O 2.445439 -0.793664 1.023623
C 4.240230 -0.066802 -0.353136
F 5.038174 -0.279886 0.702718
F 4.844316 -0.614118 -1.421527
F 4.213875 1.269796 -0.561452
C 0.649658 2.296032 -1.222974
H 0.882087 3.054170 -0.474950
H 1.551788 2.056747 -1.795125
H -0.092862 2.709059 -1.916093
42
A_5 Eopt -
1731.145918
C 0.763336 1.825787 0.818866
C 0.428102 0.572386 1.344806
C 0.785709 0.141546 2.605770
C 1.534594 1.029037 3.388141
C 1.880495 2.286842 2.897195
C 1.497827 2.698405 1.616043
C 0.233348 2.031080 -0.542038
C -0.472996 1.004436 -1.036065
H 0.509177 -0.842481 2.968615
H 1.844853 0.730235 4.383763
H 2.455829 2.964067 3.520178
H 1.766262 3.687274 1.257214
S -0.586502 -0.328257 0.192288
O -2.041336 0.206552 0.922335
C -3.183659 -0.456587 0.482678
C -3.352957 -1.817816 0.726662
C -4.163895 0.299149 -0.154808
C -4.535000 -2.431940 0.314579
H -2.577481 -2.382247 1.237246
C -5.346053 -0.325449 -0.550183
H -3.992530 1.357766 -0.326044
C -5.531398 -1.689621 -0.320887
H -4.678208 -3.491896 0.500045
H -6.119956 0.255583 -1.042078
H -6.451148 -2.173253 -0.634340
C 0.507517 3.262071 -1.368849
O -0.416740 3.944797 -1.762463
C 1.938676 3.524119 -1.737391
H 2.199706 2.821160 -2.539003
H 2.052266 4.545711 -2.102450
H 2.619376 3.327449 -0.906082
C 1.882036 -1.846192 -0.136022
O 1.359599 -0.876201 -0.766807
O 1.489637 -2.424389 0.871391
C 3.220524 -2.304151 -0.776782
F 3.750920 -3.363571 -0.157616
F 3.056686 -2.634135 -2.070236
F 4.133633 -1.315412 -0.734334
C -1.098324 0.760174 -2.362459
H -0.653548 -0.125847 -2.828000
H -2.175561 0.588045 -2.264024
H -0.942722 1.622854 -3.013170
42
A_6 Eopt -
1731.145736
C 0.954806 1.738049 0.885724
C 0.322166 0.533719 1.220632
C 0.404713 -0.067826 2.459901
C 1.176455 0.582612 3.429879
C 1.836734 1.770883 3.124553
C 1.738273 2.355204 1.856943
C 0.711443 2.138143 -0.516631
C -0.040287 1.279522 -1.224980
H -0.091686 -1.009623 2.667082
H 1.269122 0.146886 4.418948
H 2.446692 2.253334 3.881522
H 2.279112 3.269902 1.641074
S -0.589427 -0.093724 -0.174302
O -2.067126 0.672299 0.219351
C -3.195501 -0.134786 0.130046
C -3.267778 -1.359151 0.790379
C -4.268893 0.359500 -0.606467
C -4.443281 -2.104289 0.699857
H -2.425015 -1.725953 1.370751
C -5.442977 -0.388353 -0.674227
H -4.174999 1.317710 -1.107865
C -5.531558 -1.621845 -0.027132
H -4.506555 -3.061076 1.208577
H -6.286434 -0.007837 -1.242013
H -6.445099 -2.204498 -0.088544
C 1.184747 3.426457 -1.131861
O 1.596946 3.434297 -2.275733
C 1.083031 4.687454 -0.315200
H 0.368215 4.596351 0.504858
H 2.069452 4.923080 0.099537
H 0.796823 5.505531 -0.979829
C 1.572902 -2.029217 -0.206613
O 1.378919 -0.924638 -0.802156
O 0.895466 -2.579455 0.654844
C 2.877132 -2.726752 -0.679265
F 3.950444 -1.948583 -0.450087
F 3.088853 -3.891966 -0.058642
F 2.843402 -2.974991 -2.000818
C -0.545063 1.268104 -2.621277
H -0.363852 2.231361 -3.098484
H -0.027147 0.491796 -3.195740
H -1.618134 1.047883 -2.641144
42
A_7 Eopt -
1731.145856
C 0.352186 1.993218 0.508977
C 0.511171 0.835932 1.280755
C 1.137715 0.806200 2.509780
C 1.649626 2.019320 2.986306
C 1.515268 3.188178 2.238461
C 0.867781 3.189193 0.998464
C -0.365416 1.745413 -0.756040
C -0.711105 0.466400 -0.970336
H 1.243948 -0.115925 3.070480
H 2.161141 2.041951 3.942689
H 1.923392 4.117611 2.622321
H 0.777092 4.104743 0.423839
S -0.283409 -0.542830 0.482936
O -1.752749 -0.249699 1.346547
C -2.844586 -0.950830 0.853378
C -3.920189 -0.208812 0.369371
C -2.867211 -2.344440 0.863018
C -5.044123 -0.879224 -0.110182
H -3.861778 0.875776 0.375600
C -3.992588 -3.002479 0.366285
H -2.023671 -2.902717 1.259276
C -5.079614 -2.274885 -0.119366
H -5.889783 -0.309430 -0.482865
H -4.019740 -4.087749 0.368337
H -5.953599 -2.793831 -0.499949
C -0.613622 2.865191 -1.733554
O 0.307830 3.595535 -2.040069
C -2.022576 3.084022 -2.201455
H -2.529742 2.146009 -2.438314
H -2.571931 3.544609 -1.370317
H -2.033010 3.759547 -3.057715
C 2.427155 -1.407656 0.031053
O 1.579630 -0.752268 -0.655330
O 2.353773 -1.795293 1.190185
C 3.708310 -1.733715 -0.783005
F 4.277459 -0.616790 -1.266826
F 4.628419 -2.369220 -0.051042
F 3.418175 -2.519885 -1.836477
C -1.263795 -0.255140 -2.150957
H -1.250548 0.389585 -3.031512
H -0.640695 -1.129906 -2.364677
H -2.287194 -0.603432 -1.971451
42
A_8 Eopt -
1731.141808
C -1.486330 0.961940 1.117189
C -0.221545 0.373829 0.948454
C 0.514862 -0.165104 1.988903
C -0.061587 -0.132594 3.263217
C -1.309112 0.453706 3.460246
C -2.025846 1.015579 2.396852

C -2.035229 1.503236 -0.144354
C -1.232597 1.336874 -1.210096
H 1.488201 -0.604431 1.806089
H 0.479017 -0.557345 4.102125
H -1.733506 0.488005 4.458551
H -2.980556 1.495208 2.581408
S 0.294649 0.492636 -0.744438
O 0.022157 -0.988688 -1.438366
C -1.046972 -1.771216 -0.978978
C -2.284886 -1.671653 -1.604191
C -0.802445 -2.671468 0.053395
C -3.316965 -2.502812 -1.167496
H -2.430901 -0.974434 -2.423299
C -1.844799 -3.491419 0.480279
H 0.187098 -2.725081 0.496372
C -3.100281 -3.403643 -0.124591
H -4.288588 -2.445028 -1.647685
H -1.672893 -4.201546 1.282719
H -3.908515 -4.045340 0.211759
C -3.401558 2.114711 -0.296109
O -3.543320 3.095694 -0.999386
C -4.562258 1.444789 0.388659
H -4.345626 0.410006 0.661899
H -4.810954 2.005436 1.296781
H -5.426360 1.488779 -0.278067
C 3.272709 0.297353 0.089800
O 2.586023 -0.721146 -0.157125
O 2.927696 1.402055 0.529969
C 4.783486 0.114295 -0.241216
F 4.968973 -0.058195 -1.567549
F 5.542680 1.157364 0.124617
F 5.296914 -0.974911 0.365325
C -1.380256 1.692596 -2.648558
H -2.428768 1.874111 -2.889106
H -0.813684 2.603156 -2.871509
H -0.996255 0.890691 -3.287744
42
A_9 Eopt -
1731.134605
C -1.585288 -1.373260 -0.455543
C -0.618396 -1.282408 0.559083
C 0.662778 -1.792316 0.464078
C 1.007192 -2.403099 -0.749253
C 0.071384 -2.508623 -1.774119
C -1.234187 -2.011960 -1.636081
C -2.884320 -0.800011 -0.040099
C -2.908161 -0.297401 1.208162
H 1.383445 -1.704853 1.272150
H 2.012302 -2.793300 -0.868525
H 0.349737 -2.997981 -2.701944
H -1.949730 -2.139102 -2.440657
S -1.283627 -0.465797 1.971580
O -0.699547 1.082012 1.945654
C -0.565938 1.742637 0.706581
C -1.634497 2.485510 0.217276
C 0.662890 1.669213 0.060454
C -1.460892 3.176482 -0.981719
H -2.567623 2.541833 0.768754
C 0.815634 2.366424 -1.137348
H 1.475734 1.101990 0.510055
C -0.243428 3.110090 -1.660572
H -2.278202 3.769658 -1.379300
H 1.767661 2.330813 -1.657080
H -0.115738 3.649062 -2.594056
C -4.101573 -0.705102 -0.925358
O -5.193349 -0.966089 -0.462710
C -3.909364 -0.241645 -2.342468
H -2.964453 0.287673 -2.482815
H -3.926427 -1.114551 -3.005252
H -4.750426 0.400447 -2.613206
C 3.630164 -0.689754 0.871094
O 3.810159 -1.912266 0.740910
O 2.970027 -0.043370 1.705666
C 4.294988 0.159426 -0.257629
F 3.637718 -0.022468 -1.428838
F 4.288054 1.481815 -0.019801
F 5.576282 -0.189224 -0.479840
C -3.966002 0.365296 2.020060
H -4.773635 0.708221 1.371776
H -4.380922 -0.339404 2.748315
H -3.558421 1.221863 2.567043
35
B_1-notfa Eopt -
1204.924037
C 0.082844 -0.050414 -1.078534
C 0.568206 -1.354494 -0.837347
C 1.881575 -1.746103 -1.149438
C 2.724297 -0.810293 -1.734811
C 2.263542 0.487923 -1.982910
C 0.956387 0.880278 -1.658380
C -1.302601 0.097243 -0.636582
C -1.816426 -1.077073 -0.123945
H 2.218792 -2.759019 -0.949234
H 3.741276 -1.086243 -1.992031
H 2.928026 1.212348 -2.444163
H 0.618093 1.884266 -1.880552
S -0.652640 -2.358469 -0.143459
O 0.007516 -1.119280 2.469475
C 0.611521 -0.152371 2.022888
C -0.072409 1.151435 1.826573
C 2.043979 -0.257992 1.657763
C 0.600678 2.189082 1.259561
H -1.098546 1.230980 2.175875
C 2.680046 0.806791 1.105019
H 2.539261 -1.201265 1.868673
C 1.944870 1.996654 0.838317
H 0.123928 3.147335 1.089627
H 3.725628 0.757779 0.822340
H 2.457982 2.823288 0.353563
C -2.039422 1.389107 -0.774997
O -1.413263 2.402669 -1.048234
C -3.531024 1.443791 -0.591318
H -4.031980 0.681078 -1.192817
H -3.794337 1.274197 0.457747
H -3.874861 2.435171 -0.887777
C -3.156307 -1.403555 0.465203
H -3.398553 -0.715676 1.279003
H -3.944454 -1.339147 -0.289311
H -3.160573 -2.416755 0.872098
42
B_1 Eopt -
1731.128826
C -0.361605 -0.074388 0.492966
C 0.181281 -0.425997 -0.764574
C 0.785367 -1.668526 -0.999465
C 0.849700 -2.579932 0.052365
C 0.308232 -2.254811 1.297625
C -0.301075 -1.010815 1.528466
C -0.999199 1.234095 0.438766
C -0.884055 1.830358 -0.801936
H 1.210170 -1.901336 -1.970820
H 1.323447 -3.543608 -0.101068
H 0.365312 -2.970966 2.111870
H -0.692274 -0.787517 2.514502
S -0.029835 0.839076 -1.917204
O -2.701707 -0.092641 -2.320706
C -2.823135 -0.742854 -1.284389
C -2.389379 -2.149079 -1.217220
C -3.394026 -0.121085 -0.065268
C -2.480016 -2.827639 -0.044500
H -2.015394 -2.596882 -2.132917
C -3.486630 -0.851112 1.089621
H -3.771672 0.893857 -0.154216
C -2.972025 -2.164218 1.118704
H -2.158409 -3.860324 0.035120
H -3.907951 -0.418765 1.991239
H -3.010215 -2.719655 2.052163
C -1.600145 1.977133 1.588625
O -2.536223 2.736043 1.396301
C -1.011495 1.797437 2.962896
H 0.046019 1.526758 2.927170
H -1.148971 2.725122 3.521606
H -1.560061 1.005344 3.486714
C 3.095334 0.754750 -0.683840
O 2.576578 1.785126 -0.219252
O 3.201469 0.334530 -1.847900
C 3.739826 -0.153216 0.415385
F 2.913736 -0.350825 1.464324
F 4.099086 -1.373624 -0.021698
F 4.860763 0.417161 0.917428
C -1.390527 3.160985 -1.257597
H -1.282553 3.903024 -0.464047
H -0.847826 3.502541 -2.141592
H -2.454040 3.091802 -1.508272
42
B_10 Eopt -
1731.134217
C 2.028994 0.376662 1.006876
C 2.982144 1.008214 0.178852
C 4.321120 0.602675 0.135132
C 4.720974 -0.442774 0.965289
C 3.795171 -1.070280 1.802948
C 2.450594 -0.678462 1.827488
C 0.700189 0.950056 0.829506
C 0.685855 1.976812 -0.092224
H 5.031469 1.098720 -0.519630
H 5.755295 -0.769962 0.954502
H 4.119265 -1.881429 2.447957
H 1.746074 -1.169860 2.487594
S 2.257315 2.277806 -0.753337
O 1.627902 0.249641 -2.719344
C 1.592638 -0.707394 -1.953343
C 2.792284 -1.533115 -1.705091
C 0.332542 -1.047247 -1.244721
C 2.726897 -2.544046 -0.804126
H 3.687991 -1.297903 -2.271709
C 0.310721 -2.086506 -0.355860
H -0.558139 -0.467795 -1.472723
C 1.507235 -2.784174 -0.094227
H 3.588829 -3.165828 -0.588129
H -0.599334 -2.335851 0.176782
H 1.495035 -3.580404 0.646349
C -0.454600 0.515444 1.684249
O -0.434393 -0.603968 2.169565
C -1.533649 1.502452 2.027311
H -2.110790 1.782335 1.143305
H -2.200571 1.046467 2.760945
H -1.085259 2.411624 2.443308

C -3.457578 0.357804 -0.436986
O -2.350941 -0.217438 -0.436123
O -3.751825 1.551872 -0.598014
C -4.650176 -0.593436 -0.111324
F -4.685150 -0.863640 1.214657
F -5.850314 -0.083600 -0.431416
F -4.553111 -1.780522 -0.741158
C -0.459740 2.794189 -0.603569
H -0.751959 3.553253 0.129425
H -0.186464 3.305126 -1.529738
H -1.326961 2.154380 -0.799331
42
B_11 Eopt -
1731.134207
C -1.193124 -0.049792 -1.009153
C -2.547585 -0.431252 -1.119286
C -2.938854 -1.722815 -1.518487
C -1.947599 -2.646601 -1.815557
C -0.596692 -2.290311 -1.696640
C -0.206131 -1.004262 -1.296614
C -1.057211 1.334597 -0.571019
C -2.275746 1.949742 -0.388399
H -3.990311 -1.982662 -1.599234
H -2.220236 -3.649307 -2.127398
H 0.175943 -3.018259 -1.927296
H 0.847975 -0.754265 -1.244973
S -3.600600 0.882992 -0.728839
O -3.094952 0.236176 2.212087
C -2.125051 -0.479129 2.000410
C -2.307297 -1.900439 1.611172
C -0.742093 0.047998 2.117612
C -1.226505 -2.663076 1.313491
H -3.325258 -2.279090 1.596322
C 0.311077 -0.740344 1.784505
H -0.631664 1.067400 2.477661
C 0.068548 -2.065200 1.312353
H -1.327654 -3.702610 1.022042
H 1.335841 -0.387070 1.804953
H 0.933180 -2.646996 1.004508
C 0.282624 1.966280 -0.370150
O 1.260353 1.251588 -0.221050
C 0.425117 3.464918 -0.373774
H 0.036966 3.884480 0.560043
H 1.485802 3.706104 -0.453992
H -0.126342 3.914381 -1.203546
C 3.240768 -0.852014 -0.555180
O 2.765112 -1.679981 0.247776
O 3.175650 -0.784700 -1.791411
C 4.120378 0.241470 0.127029
F 5.374561 -0.243023 0.326615
F 4.262831 1.362544 -0.595437
F 3.666835 0.609556 1.340344
C -2.622419 3.327700 0.092568
H -2.317859 4.087464 -0.631955
H -3.699269 3.422389 0.247615
H -2.128098 3.538971 1.044471
42
B_12 Eopt -
1731.133374
C 1.276720 0.032453 0.985206
C 1.657391 -1.318924 1.136589
C 0.733920 -2.342258 1.421923
C -0.601349 -1.999434 1.568582
C -1.002019 -0.665050 1.415632
C -0.083212 0.351569 1.121054
C 2.424201 0.884159 0.677936
C 3.605978 0.173947 0.636586
H 1.065705 -3.370554 1.532708
H -1.338832 -2.763868 1.788944
H -2.049031 -0.409731 1.546131
H -0.417877 1.376981 1.029412
S 3.364010 -1.512524 0.953417
O 2.937438 -1.390851 -2.039831
C 1.791918 -0.966030 -1.974726
C 0.655729 -1.886267 -1.721482
C 1.504455 0.482900 -2.140094
C -0.600971 -1.390124 -1.593239
H 0.885645 -2.946415 -1.662993
C 0.235573 0.939825 -1.988843
H 2.342011 1.125853 -2.396993
C -0.800394 0.019055 -1.648858
H -1.465482 -2.015178 -1.397210
H -0.005151 1.992363 -2.085420
H -1.814781 0.379850 -1.493374
C 2.277386 2.353812 0.461912
O 1.158156 2.830623 0.343187
C 3.488109 3.245540 0.394221
H 4.062894 3.041797 -0.514657
H 3.146253 4.280769 0.373297
H 4.146146 3.089323 1.252556
C -4.151504 -0.729512 0.044419
O -4.497956 -1.423192 1.009860
O -3.554383 -1.015997 -1.012653
C -4.503511 0.791408 0.137604
F -3.407191 1.561489 -0.066211
F -5.398628 1.150176 -0.807559
F -5.016453 1.165764 1.319107
C 5.013906 0.593878 0.332924
H 5.391550 1.287416 1.088224
H 5.675727 -0.274426 0.313086
H 5.071506 1.078998 -0.645012
42
B_13 Eopt -
1731.130323
C 1.197191 -0.092457 -1.001084
C -0.045115 -0.394901 -0.396474
C -0.681076 -1.641124 -0.556140
C -0.060546 -2.597321 -1.350495
C 1.173288 -2.320648 -1.948262
C 1.811373 -1.080419 -1.780052
C 1.643932 1.258019 -0.667470
C 0.732904 1.920012 0.131070
H -1.638464 -1.814242 -0.072727
H -0.532178 -3.563321 -1.496360
H 1.651466 -3.074492 -2.566998
H 2.753977 -0.880903 -2.273679
S -0.644500 0.943913 0.512628
O 1.179546 0.163344 2.675079
C 1.869064 -0.597968 2.005980
C 1.424516 -1.986047 1.744950
C 3.160095 -0.151906 1.430938
C 2.175671 -2.800027 0.956219
H 0.502903 -2.305958 2.222881
C 3.870830 -0.993416 0.632492
H 3.503599 0.843764 1.698267
C 3.347434 -2.283629 0.339684
H 1.872102 -3.819610 0.746825
H 4.809089 -0.688336 0.183722
H 3.912248 -2.932733 -0.324454
C 2.927493 1.813180 -1.185632
O 3.747913 1.056950 -1.686794
C 3.205896 3.289922 -1.108470
H 3.340920 3.602187 -0.068338
H 4.121419 3.495357 -1.664149
H 2.377891 3.867207 -1.528507
C -3.801968 0.170640 0.937899
O -4.183137 1.158379 1.577930
O -3.023625 -0.760724 1.232517
C -4.367332 0.036663 -0.513381
F -5.092676 -1.094422 -0.659547
F -5.158601 1.052605 -0.891351
F -3.368748 -0.033306 -1.423814
C 0.769797 3.283975 0.754898
H 0.764725 4.068978 -0.005318
H -0.099499 3.433437 1.398844
H 1.665752 3.403264 1.370490
42
B_14 Eopt -
1731.133632
C 0.491033 -0.558210 -0.292943
C 0.668297 -1.101144 0.999091
C 1.279898 -2.344692 1.221810
C 1.711609 -3.074232 0.119118
C 1.545658 -2.555405 -1.167703
C 0.943121 -1.304660 -1.387554
C -0.130704 0.763599 -0.237809
C -0.451855 1.135897 1.055655
H 1.395864 -2.730466 2.230330
H 2.179147 -4.042507 0.262027
H 1.877254 -3.132994 -2.025475
H 0.790330 -0.942461 -2.395533
S 0.018982 -0.059525 2.208871
O 2.519545 1.474837 2.003236
C 2.881537 1.029269 0.919448
C 3.627855 -0.244433 0.834465
C 2.575091 1.750504 -0.340714
C 3.959541 -0.753141 -0.380194
H 3.897272 -0.723152 1.771318
C 2.924814 1.201560 -1.539074
H 2.106198 2.726595 -0.249970
C 3.543620 -0.074130 -1.561858
H 4.499572 -1.688869 -0.472840
H 2.702636 1.701832 -2.475111
H 3.781196 -0.518754 -2.524862
C -0.388060 1.567717 -1.473001
O -0.166734 1.062927 -2.563781
C -0.852018 2.994097 -1.374970
H -0.197244 3.579200 -0.722468
H -0.849488 3.421210 -2.378643
H -1.866900 3.020639 -0.968581
C -3.358744 0.337393 0.275606
O -3.540007 0.292961 1.503395
O -3.386124 1.287244 -0.528093
C -3.073004 -1.059363 -0.366635
F -2.512879 -0.991989 -1.585987
F -2.256159 -1.820648 0.390268
F -4.223263 -1.760708 -0.508748
C -1.108968 2.367148 1.598616
H -2.072982 2.523610 1.108024
H -1.287063 2.263367 2.670782
H -0.474574 3.244873 1.443097
42
B_15 Eopt -
1731.133968
C -1.510835 0.072006 -1.077910
C -0.705560 -1.060085 -0.811014
C 0.675654 -1.097265 -1.090426
C 1.251849 0.021149 -1.670657

C 0.475610 1.159017 -1.931091
C -0.894669 1.198523 -1.638705
C -2.902542 -0.151324 -0.687016
C -3.106169 -1.418752 -0.186282
H 1.281720 -1.969152 -0.857379
H 2.309749 0.014642 -1.911321
H 0.940738 2.031481 -2.380704
H -1.475561 2.083286 -1.864924
S -1.643818 -2.353202 -0.160520
O -1.583977 -0.920403 2.487124
C -1.122366 0.116957 2.030185
C 0.321152 0.218078 1.700464
C -1.987058 1.297996 1.785616
C 0.819903 1.373391 1.179868
H 0.960537 -0.637966 1.901104
C -1.454135 2.416903 1.234729
H -3.030457 1.219962 2.079039
C -0.068175 2.435122 0.882363
H 1.874424 1.453513 0.939605
H -2.060972 3.291084 1.028299
H 0.331952 3.334355 0.420710
C -3.940502 0.909638 -0.836590
O -3.595154 2.051091 -1.107480
C -5.400107 0.585422 -0.662588
H -5.615024 0.328989 0.379497
H -5.979731 1.466663 -0.939205
H -5.694398 -0.262956 -1.286017
C 3.718776 -1.004657 0.242659
O 3.681618 -1.954812 -0.553515
O 3.096888 -0.784672 1.299442
C 4.720798 0.141861 -0.115064
F 5.720534 0.220111 0.789160
F 5.298737 0.008147 -1.319459
F 4.113393 1.352290 -0.115458
C -4.326998 -2.090196 0.370101
H -5.109978 -2.178887 -0.386561
H -4.083743 -3.094463 0.723191
H -4.724896 -1.526603 1.218366
42
B_16 Eopt -
1731.136222
C 2.174944 0.385955 0.960806
C 3.036494 1.006796 0.030508
C 4.353873 0.580901 -0.181048
C 4.831170 -0.483403 0.578043
C 3.999607 -1.104515 1.514658
C 2.677459 -0.686980 1.711854
C 0.839806 0.981426 0.938511
C 0.746551 2.026428 0.036245
H 4.986689 1.075890 -0.911696
H 5.849571 -0.830230 0.437864
H 4.382448 -1.930896 2.106088
H 2.054150 -1.173644 2.450239
S 2.238084 2.305494 -0.791240
O 1.148473 0.308227 -2.621057
C 1.249308 -0.658838 -1.875184
C 2.483939 -1.473171 -1.850344
C 0.129427 -1.028274 -0.968809
C 2.585993 -2.499743 -0.972082
H 3.266989 -1.213221 -2.556102
C 0.273975 -2.084740 -0.111278
H -0.799131 -0.460807 -1.051913
C 1.506239 -2.767846 -0.069650
H 3.478511 -3.113423 -0.919302
H -0.538875 -2.355541 0.552723
H 1.628572 -3.577593 0.645718
C -0.241876 0.521636 1.867370
O -0.066503 -0.500670 2.512482
C -1.493791 1.333655 2.039106
H -2.040618 1.408212 1.095517
H -2.119539 0.843305 2.785502
H -1.244793 2.347535 2.369562
C -3.220828 -0.383971 -0.308453
O -2.681096 -1.295558 0.336973
O -2.723621 0.524540 -1.005792
C -4.772860 -0.304202 -0.183099
F -5.372259 0.072278 -1.327376
F -5.341889 -1.462204 0.189766
F -5.114513 0.614525 0.751811
C -0.415256 2.884503 -0.364078
H -0.753296 3.504395 0.470454
H -0.136749 3.546133 -1.187095
H -1.248400 2.254568 -0.694845
42
B_17 Eopt -
1731.134825
C -1.480019 0.027707 -1.097144
C -0.470626 -0.906468 -0.774489
C 0.888359 -0.690517 -1.066818
C 1.238978 0.485827 -1.714400
C 0.254233 1.427243 -2.040766
C -1.097350 1.215068 -1.736947
C -2.797991 -0.436468 -0.667755
C -2.747251 -1.680595 -0.075852
H 1.637115 -1.424726 -0.785930
H 2.279355 0.682232 -1.952760
H 0.540063 2.345590 -2.545134
H -1.841094 1.952252 -2.011789
S -1.132481 -2.308512 -0.015780
O -1.189277 -0.758047 2.523901
C -1.023496 0.338705 2.005824
C 0.333462 0.800188 1.620828
C -2.179772 1.234076 1.743692
C 0.484979 2.014406 1.022354
H 1.180947 0.148747 1.838563
C -1.982684 2.423312 1.120408
H -3.154412 0.897023 2.086313
C -0.663760 2.783295 0.710444
H 1.474026 2.357377 0.739169
H -2.806830 3.091906 0.900243
H -0.529031 3.725677 0.185090
C -4.027972 0.378046 -0.890559
O -3.916993 1.556237 -1.198190
C -5.391464 -0.245430 -0.758660
H -5.607783 -0.476442 0.289126
H -6.130462 0.468305 -1.124237
H -5.456408 -1.175249 -1.329803
C 3.592076 0.017559 0.776224
O 3.050113 -0.973349 1.300368
O 3.436456 1.234543 0.970381
C 4.655940 -0.364052 -0.302925
F 4.167375 -1.250242 -1.198346
F 5.111808 0.681951 -1.012020
F 5.734374 -0.948384 0.262867
C -3.813994 -2.527883 0.552968
H -4.545762 -2.859906 -0.187710
H -3.375616 -3.415103 1.014802
H -4.338699 -1.970616 1.333773
42
B_18 Eopt -
1731.134010
C -1.241256 0.092133 -0.937739
C -1.858221 -1.109047 -1.347753
C -1.126982 -2.248845 -1.734396
C 0.256380 -2.185401 -1.697566
C 0.891108 -1.014581 -1.257190
C 0.162121 0.124134 -0.880548
C -2.226271 1.109987 -0.582264
C -3.521615 0.664710 -0.750185
H -1.640669 -3.150103 -2.055884
H 0.847171 -3.048038 -1.986551
H 1.975076 -0.976097 -1.204484
H 0.694583 1.013213 -0.566895
S -3.579615 -0.968030 -1.328045
O -3.439335 -1.314683 1.767268
C -2.226696 -1.164292 1.817526
C -1.311301 -2.286649 1.489011
C -1.628731 0.140854 2.202088
C 0.027943 -2.083108 1.470024
H -1.769828 -3.250056 1.284545
C -0.285589 0.311112 2.138013
H -2.312530 0.920344 2.527297
C 0.540645 -0.768907 1.692287
H 0.722865 -2.879978 1.228042
H 0.181904 1.260780 2.373465
H 1.614940 -0.608266 1.598690
C -1.821245 2.466909 -0.109091
O -0.645813 2.686590 0.141208
C -2.843749 3.558889 0.057797
H -3.544349 3.311081 0.861020
H -2.317755 4.479117 0.313787
H -3.418366 3.705969 -0.860439
C 3.736048 0.095154 0.476679
O 2.900354 1.016882 0.484003
O 3.673058 -1.060809 0.933808
C 5.049504 0.437953 -0.292264
F 5.469237 1.696606 -0.069297
F 6.077530 -0.372782 0.007609
F 4.848743 0.329154 -1.627701
C -4.846656 1.318979 -0.489274
H -4.991746 2.191059 -1.131238
H -5.663020 0.619880 -0.682494
H -4.922745 1.639024 0.553147
42
B_19 Eopt -
1731.128412
C 1.123570 -0.097744 -0.956453
C 0.441645 -1.167347 -0.334877
C 0.726467 -2.514601 -0.617441
C 1.709744 -2.797032 -1.557488
C 2.399316 -1.752090 -2.181292
C 2.120811 -0.406755 -1.890284
C 0.648969 1.194795 -0.463303
C -0.377104 1.061393 0.454027
H 0.177094 -3.309771 -0.121875
H 1.944402 -3.827860 -1.800111
H 3.164855 -1.979534 -2.917165
H 2.652231 0.382744 -2.405376
S -0.763034 -0.598436 0.760253
O 1.398898 -0.212777 2.717750
C 2.330336 -0.267777 1.923246
C 2.879334 -1.573107 1.491840
C 2.929777 0.968512 1.364261
C 3.872899 -1.615791 0.566088
H 2.464448 -2.461219 1.959382
C 3.917014 0.879192 0.430839
H 2.565186 1.915425 1.753473
C 4.336026 -0.402588 -0.015678

H 4.290377 -2.557455 0.227763
H 4.362617 1.764939 -0.006832
H 5.100138 -0.457159 -0.787197
C 1.232298 2.480489 -0.945551
O 2.234703 2.453227 -1.646279
C 0.602187 3.796783 -0.581739
H 0.604437 3.949310 0.501407
H 1.173915 4.591419 -1.062030
H -0.435859 3.832829 -0.924839
C -3.971461 -0.712404 0.829662
O -3.747017 0.103317 1.742407
O -4.222482 -1.927340 0.846909
C -3.976353 -0.090023 -0.607115
F -3.467516 -0.915794 -1.542196
F -5.240237 0.197987 -0.996910
F -3.278915 1.059693 -0.697233
C -1.151370 2.072842 1.244189
H -1.583482 2.836861 0.596359
H -1.971556 1.582074 1.772924
H -0.497680 2.563501 1.973235
35
B_2-notfa Eopt -
1204.922399
C 0.064836 0.134639 -1.073298
C 0.587778 -1.179466 -1.066391
C 1.913613 -1.464047 -1.428880
C 2.730907 -0.410306 -1.821944
C 2.236665 0.898096 -1.825386
C 0.916114 1.181335 -1.449068
C -1.305977 0.159169 -0.578603
C -1.780703 -1.096259 -0.265788
H 2.285457 -2.484213 -1.411115
H 3.757349 -0.604931 -2.113343
H 2.883280 1.715891 -2.128451
H 0.566701 2.207064 -1.476606
S -0.599470 -2.327399 -0.551686
O 0.046665 -1.582379 2.214381
C 0.609881 -0.524737 1.961861
C -0.139599 0.759059 1.988854
C 2.046611 -0.495877 1.610431
C 0.496055 1.918391 1.655904
H -1.176306 0.720568 2.312176
C 2.635882 0.681809 1.280986
H 2.583814 -1.438814 1.647263
C 1.851787 1.871093 1.244125
H -0.025569 2.869484 1.667036
H 3.685486 0.734440 1.014034
H 2.336629 2.797888 0.947375
C -2.198815 1.356186 -0.479251
O -3.076631 1.386256 0.367315
C -2.014774 2.494395 -1.447724
H -1.284381 3.201809 -1.037339
H -1.654524 2.153206 -2.420920
H -2.967032 3.016246 -1.555013
C -3.115764 -1.499231 0.272749
H -3.151468 -1.319482 1.351961
H -3.909343 -0.907757 -0.187846
H -3.305829 -2.559529 0.092739
42
B_2 Eopt -
1731.130348
C 0.739475 0.436044 0.622672
C 1.010309 1.521206 -0.240655
C 1.807035 2.604074 0.145131
C 2.327710 2.612230 1.438887
C 2.066696 1.551439 2.307115
C 1.283132 0.456408 1.908253
C -0.020973 -0.600391 -0.063815
C -0.352329 -0.250671 -1.360259
H 2.003435 3.422907 -0.540737
H 2.939802 3.445893 1.765928
H 2.473716 1.567864 3.313541
H 1.094394 -0.344549 2.613722
S 0.263014 1.296591 -1.782033
O 2.490334 -0.341415 -2.488806
C 2.868728 -0.628607 -1.354677
C 3.836585 0.218394 -0.638355
C 2.342387 -1.834503 -0.668340
C 4.201198 -0.100420 0.629489
H 4.237443 1.074510 -1.172204
C 2.761483 -2.131470 0.604849
H 1.684886 -2.485757 -1.237958
C 3.628093 -1.243559 1.266681
H 4.908214 0.508989 1.181898
H 2.399500 -3.016266 1.118360
H 3.922474 -1.464701 2.289350
C -0.557432 -1.858203 0.546437
O -0.663857 -2.863225 -0.136132
C -0.974579 -1.844708 1.992625
H -1.284943 -0.851065 2.323307
H -1.785398 -2.562864 2.126923
H -0.127940 -2.170858 2.609147
C -2.925650 1.196661 -0.207409
O -3.109096 1.289618 -1.433827
O -2.153092 1.789724 0.563101
C -3.799263 0.083611 0.460584
F -3.303463 -1.146012 0.164850
F -3.849553 0.155875 1.802498
F -5.074904 0.088768 0.028267
C -1.113975 -1.029851 -2.381876
H -1.926291 -1.585028 -1.909901
H -1.533529 -0.364985 -3.138542
H -0.448802 -1.750951 -2.869557
42
B_20 Eopt -
1731.134532
C -1.916325 0.237142 -0.943268
C -0.622895 0.435142 -1.480807
C -0.191846 1.679721 -1.975332
C -1.090373 2.739607 -1.957658
C -2.372415 2.563843 -1.426485
C -2.793104 1.328187 -0.912146
C -2.100698 -1.138403 -0.482451
C -0.988393 -1.918162 -0.729033
H 0.822453 1.791335 -2.343146
H -0.786022 3.708969 -2.337870
H -3.065383 3.400088 -1.411067
H -3.794350 1.214267 -0.516988
S 0.301840 -1.018857 -1.450017
O 0.909230 -0.463638 1.271112
C 0.042865 0.381717 1.474263
C 0.255897 1.791987 1.076891
C -1.245128 0.009381 2.104740
C -0.744016 2.699171 1.262844
H 1.235875 2.047324 0.677680
C -2.218755 0.947361 2.255504
H -1.349302 -1.015019 2.451887
C -1.990798 2.268822 1.780015
H -0.617520 3.738217 0.979561
H -3.175704 0.702120 2.701293
H -2.791097 2.997870 1.879960
C -3.368024 -1.591297 0.158403
O -4.187511 -0.758740 0.521381
C -3.644627 -3.058473 0.348996
H -2.949815 -3.490337 1.075500
H -4.664334 -3.169732 0.718952
H -3.529166 -3.603639 -0.591833
C 3.223478 0.774001 -0.133685
O 3.433699 1.673262 0.699026
O 2.624023 0.799134 -1.224400
C 3.869244 -0.598516 0.232508
F 3.883660 -0.845825 1.553688
F 3.275801 -1.651979 -0.353531
F 5.165056 -0.618002 -0.172656
C -0.688700 -3.352710 -0.404932
H -1.364615 -4.030091 -0.932057
H 0.334077 -3.602996 -0.695508
H -0.783025 -3.535694 0.669253
42
B_21 Eopt -
1731.138487
C -0.984352 0.013815 -0.960246
C -2.000120 -0.847033 -1.431588
C -1.737464 -2.158458 -1.858434
C -0.427342 -2.619444 -1.812042
C 0.591352 -1.794783 -1.320424
C 0.323957 -0.487628 -0.887541
C -1.530950 1.287080 -0.516424
C -2.895491 1.368280 -0.693291
H -2.541313 -2.792691 -2.220577
H -0.199047 -3.628251 -2.138890
H 1.611121 -2.162600 -1.254205
H 1.138143 0.124577 -0.518395
S -3.549525 -0.073519 -1.392562
O -3.651478 -0.690945 1.608739
C -2.456186 -0.926296 1.716692
C -1.915553 -2.270947 1.404123
C -1.506686 0.133465 2.154933
C -0.579165 -2.485386 1.450578
H -2.633756 -3.045372 1.151346
C -0.172035 -0.122819 2.177262
H -1.935437 1.088095 2.448279
C 0.299095 -1.395747 1.747435
H -0.149741 -3.453352 1.215916
H 0.560531 0.630204 2.448597
H 1.376452 -1.557063 1.696991
C -0.756168 2.447611 0.023905
O -1.302138 3.235452 0.780969
C 0.677934 2.633233 -0.387673
H 0.857117 2.298347 -1.412469
H 0.935903 3.688438 -0.281145
H 1.328168 2.053753 0.283272
C 3.366579 -0.315561 0.645998
O 2.728268 0.510850 1.328199
O 3.289794 -1.554348 0.593154
C 4.386763 0.351568 -0.326792
F 5.132781 -0.523606 -1.017398
F 3.740586 1.115044 -1.238044
F 5.240348 1.167322 0.322170
C -3.832347 2.485892 -0.363435
H -3.372405 3.450584 -0.585490
H -4.764641 2.392458 -0.924917
H -4.068622 2.471209 0.705412
42
B_22 Eopt -
1731.129890
C -0.426791 -0.073008 0.542867
C 0.199158 -0.397155 -0.681797

C 0.848979 -1.624620 -0.896465
C 0.881624 -2.543687 0.146918
C 0.267089 -2.241101 1.365058
C -0.390614 -1.016810 1.575332
C -1.048964 1.246794 0.490160
C -0.858323 1.863027 -0.733281
H 1.335905 -1.829563 -1.844387
H 1.387797 -3.493499 0.011437
H 0.305028 -2.960314 2.178035
H -0.833767 -0.793625 2.537495
S 0.049408 0.882874 -1.821042
O -2.625856 -0.087704 -2.397709
C -2.763973 -0.777874 -1.391026
C -2.244204 -2.159900 -1.345710
C -3.443485 -0.242638 -0.189037
C -2.345046 -2.883096 -0.199900
H -1.803723 -2.553100 -2.257023
C -3.521923 -1.002097 0.940637
H -3.894149 0.742466 -0.271160
C -2.910128 -2.281136 0.960103
H -1.963677 -3.896257 -0.136706
H -4.003236 -0.629760 1.837198
H -2.943877 -2.859360 1.879757
C -1.744565 1.836300 1.671490
O -2.076848 1.105963 2.594333
C -2.014946 3.314972 1.737080
H -2.819372 3.585739 1.045413
H -2.326349 3.560296 2.753034
H -1.127097 3.892434 1.467598
C 3.172171 0.740216 -0.657768
O 2.735147 1.829252 -0.246518
O 3.206528 0.238189 -1.794012
C 3.785337 -0.135445 0.484311
F 2.929565 -0.285234 1.517975
F 4.138937 -1.374638 0.098992
F 4.900215 0.437728 0.994040
C -1.335020 3.184914 -1.256380
H -0.838036 4.011543 -0.741987
H -1.119789 3.274680 -2.323173
H -2.414650 3.290091 -1.122215
35
B_3-notfa Eopt -
1204.917340
C -0.094805 -0.065735 -1.063073
C 0.383226 -1.374705 -0.795307
C 1.642501 -1.825225 -1.237258
C 2.434437 -0.952215 -1.966415
C 1.982309 0.347530 -2.234870
C 0.727341 0.792087 -1.801862
C -1.440894 0.123876 -0.527212
C -1.924056 -0.997506 0.095641
H 1.970360 -2.838732 -1.025270
H 3.406192 -1.274531 -2.324497
H 2.605062 1.020754 -2.815921
H 0.399375 1.788453 -2.077204
S -0.778815 -2.307336 0.068490
O 0.184293 -1.013157 2.457961
C 0.850097 -0.128473 1.932302
C 0.324426 1.251270 1.810684
C 2.206820 -0.414221 1.410459
C 1.089304 2.212616 1.227216
H -0.657126 1.445678 2.234045
C 2.934611 0.579778 0.827497
H 2.586669 -1.422757 1.546518
C 2.363101 1.868135 0.693781
H 0.740726 3.235642 1.140725
H 3.930405 0.396507 0.439947
H 2.944415 2.645586 0.204434
C -2.322032 1.326101 -0.693294
O -3.508811 1.157273 -0.913483
C -1.774362 2.714463 -0.500152
H -1.875330 2.955533 0.565715
H -0.722279 2.813105 -0.760552
H -2.377504 3.422262 -1.071100
C -3.240549 -1.222200 0.769316
H -3.638236 -0.281440 1.153323
H -3.966141 -1.626360 0.056680
H -3.138254 -1.928707 1.596764
42
B_3 Eopt -
1731.132713
C 0.526730 -0.567412 -0.042563
C 0.927735 -0.732101 1.301520
C 1.563814 -1.897393 1.758027
C 1.783052 -2.927912 0.849490
C 1.390119 -2.781623 -0.484228
C 0.766708 -1.608964 -0.942471
C -0.091722 0.735168 -0.259609
C -0.158802 1.484787 0.896223
H 1.859624 -1.996474 2.798280
H 2.264383 -3.842521 1.178699
H 1.559768 -3.593461 -1.185234
H 0.445761 -1.525534 -1.974052
S 0.529134 0.653753 2.244179
O 2.944705 1.889274 1.231331
C 3.111683 1.145610 0.268454
C 3.821682 -0.141144 0.422940
C 2.599038 1.510218 -1.072449
C 3.944167 -0.977772 -0.640752
H 4.239992 -0.360746 1.400676
C 2.745126 0.641114 -2.113080
H 2.149965 2.493476 -1.181058
C 3.346616 -0.621510 -1.882674
H 4.453748 -1.930955 -0.553452
H 2.368731 0.878386 -3.100723
H 3.418424 -1.321195 -2.711493
C -0.649970 1.129158 -1.588654
O -0.218657 0.593604 -2.598922
C -1.756858 2.145799 -1.666538
H -1.330590 3.155353 -1.650314
H -2.280530 2.007096 -2.613998
H -2.451789 2.051431 -0.827072
C -2.981583 -0.194803 1.012891
O -3.270759 0.996139 1.227461
O -2.303233 -1.011163 1.656800
C -3.536897 -0.778859 -0.329621
F -4.461215 -0.003074 -0.923092
F -2.538605 -0.939065 -1.233992
F -4.099468 -1.993318 -0.170175
C -0.686542 2.865051 1.134226
H -1.779916 2.832450 1.170116
H -0.322384 3.258301 2.086065
H -0.368780 3.546151 0.341877
42
B_4 Eopt -
1731.133245
C 1.779562 -0.328820 -0.981994
C 0.428505 -0.368404 -1.404243
C -0.199815 -1.558586 -1.798041
C 0.555719 -2.729931 -1.802573
C 1.890693 -2.710589 -1.394769
C 2.506928 -1.523863 -0.971521
C 2.150174 1.008035 -0.531905
C 1.124312 1.917039 -0.680476
H -1.248801 -1.546916 -2.079815
H 0.093510 -3.662060 -2.109935
H 2.467768 -3.630402 -1.396158
H 3.544984 -1.548549 -0.660672
S -0.307889 1.197255 -1.323345
O -0.784414 0.684754 1.402295
C 0.050399 -0.194518 1.602368
C -0.274258 -1.611861 1.352015
C 1.411631 0.152132 2.080858
C 0.685681 -2.559652 1.532260
H -1.290707 -1.831786 1.028044
C 2.338246 -0.836633 2.252616
H 1.608185 1.195409 2.313522
C 1.994299 -2.170094 1.924683
H 0.479753 -3.608376 1.347849
H 3.340441 -0.612542 2.602955
H 2.753701 -2.941268 2.028790
C 3.490877 1.441639 -0.033920
O 3.569303 2.346322 0.781782
C 4.731423 0.772397 -0.564921
H 4.596953 0.392345 -1.579787
H 5.552693 1.490705 -0.532269
H 4.993374 -0.066997 0.090212
C -3.213741 -0.515908 -0.592851
O -2.970191 -0.066050 -1.727815
O -3.054416 -1.654207 -0.116667
C -3.879414 0.518850 0.367702
F -5.201140 0.628007 0.079499
F -3.802751 0.181025 1.665092
F -3.363761 1.754053 0.250947
C 1.094933 3.371361 -0.331926
H 2.053804 3.842775 -0.555930
H 0.303320 3.887439 -0.879804
H 0.906932 3.494258 0.739758
42
B_5 Eopt -
1731.134729
C 1.764577 -0.293138 -1.004194
C 0.400928 -0.348436 -1.375785
C -0.215357 -1.532202 -1.817754
C 0.566942 -2.677974 -1.921270
C 1.918636 -2.641454 -1.567230
C 2.525214 -1.464874 -1.101557
C 2.143127 1.043705 -0.549017
C 1.090391 1.933511 -0.626193
H -1.273465 -1.527762 -2.064269
H 0.118084 -3.603825 -2.265248
H 2.519957 -3.541961 -1.651612
H 3.576331 -1.457200 -0.843206
S -0.360673 1.193119 -1.209182
O -0.688918 0.574520 1.568423
C 0.139570 -0.326677 1.657850
C -0.219347 -1.714408 1.290449
C 1.520456 -0.048844 2.120381
C 0.730757 -2.689023 1.340093
H -1.252799 -1.889471 0.993370
C 2.439108 -1.052772 2.138940
H 1.739244 0.961248 2.456143
C 2.061729 -2.348562 1.690264
H 0.499250 -3.713032 1.068588
H 3.460328 -0.879485 2.457869
H 2.818207 -3.129540 1.677064
C 3.526985 1.357845 -0.091101
O 4.304067 0.441751 0.138854

C 3.970920 2.786277 0.075323
H 3.451476 3.254395 0.917196
H 5.043477 2.790000 0.272181
H 3.755375 3.372306 -0.822041
C -3.234601 -0.505032 -0.551613
O -3.021322 -0.076034 -1.700834
O -3.048658 -1.629862 -0.054715
C -3.885806 0.547844 0.399595
F -5.196721 0.704175 0.089828
F -3.841023 0.209348 1.697768
F -3.323450 1.765063 0.291277
C 0.982445 3.378700 -0.236177
H 1.629245 4.007139 -0.853142
H -0.044567 3.730051 -0.357621
H 1.259969 3.520027 0.811921
42
B_6 Eopt -
1731.133435
C -1.491766 0.072350 -1.096179
C -0.480870 -0.885792 -0.847888
C 0.876586 -0.638588 -1.112789
C 1.225325 0.590214 -1.659962
C 0.242005 1.555848 -1.904458
C -1.107716 1.313322 -1.620450
C -2.796464 -0.414197 -0.662628
C -2.746369 -1.693928 -0.160185
H 1.629324 -1.388759 -0.890226
H 2.265433 0.808932 -1.878236
H 0.527566 2.516814 -2.321393
H -1.837895 2.087779 -1.825313
S -1.140080 -2.343343 -0.191314
O -1.146126 -1.013153 2.436241
C -1.012308 0.131125 2.021793
C 0.327955 0.666048 1.684827
C -2.198418 1.011059 1.842985
C 0.442088 1.938088 1.211448
H 1.194344 0.020242 1.836500
C -2.032927 2.271883 1.361635
H -3.163779 0.608016 2.137050
C -0.727684 2.710254 0.998379
H 1.419710 2.335166 0.960662
H -2.876731 2.939361 1.222504
H -0.618646 3.709375 0.583148
C -4.098798 0.306404 -0.786948
O -4.988149 0.098534 0.023151
C -4.307083 1.263807 -1.931654
H -3.724346 0.990647 -2.813817
H -5.371620 1.289667 -2.171177
H -4.009444 2.270446 -1.614798
C 3.598080 0.032030 0.766651
O 3.082317 -0.983659 1.269482
O 3.420434 1.240067 0.995482
C 4.657106 -0.300077 -0.333396
F 4.174683 -1.169524 -1.248309
F 5.087080 0.773157 -1.017677
F 5.751719 -0.880256 0.204808
C -3.847201 -2.543021 0.390845
H -4.773788 -2.377669 -0.161913
H -3.584103 -3.602227 0.344411
H -4.033136 -2.279992 1.437245
42
B_7 Eopt -
1731.134801
C -1.474717 0.030358 -1.110474
C -0.465954 -0.906892 -0.795038
C 0.894172 -0.689341 -1.080144
C 1.247982 0.492135 -1.716613
C 0.264468 1.435869 -2.038653
C -1.088317 1.222525 -1.739905
C -2.793316 -0.434856 -0.681062
C -2.743078 -1.688484 -0.106885
H 1.641389 -1.426666 -0.803158
H 2.289195 0.689644 -1.950317
H 0.551711 2.357774 -2.535667
H -1.830085 1.961729 -2.013111
S -1.129425 -2.317778 -0.054864
O -1.199588 -0.780217 2.504321
C -1.033590 0.320209 1.994478
C 0.323969 0.787188 1.618130
C -2.190473 1.215975 1.734284
C 0.475196 2.004550 1.026275
H 1.172059 0.136749 1.836521
C -1.993590 2.408444 1.117033
H -3.165430 0.876386 2.073673
C -0.674048 2.772251 0.712414
H 1.464596 2.351539 0.749337
H -2.817993 3.077186 0.897794
H -0.539324 3.716908 0.191147
C -4.019508 0.392027 -0.874571
O -3.906127 1.551365 -1.247136
C -5.384951 -0.186055 -0.616538
H -5.511467 -0.418347 0.445341
H -6.129008 0.554308 -0.911840
H -5.536553 -1.108060 -1.183944
C 3.585417 0.019555 0.790474
O 3.042809 -0.974909 1.307169
O 3.427429 1.235286 0.990633
C 4.654754 -0.354427 -0.285781
F 4.174708 -1.242564 -1.183763
F 5.106689 0.695117 -0.992155
F 5.734877 -0.932717 0.283165
C -3.807942 -2.553620 0.500887
H -4.551484 -2.848961 -0.243250
H -3.369793 -3.462796 0.917964
H -4.318502 -2.028136 1.312348
42
B_8 Eopt -
1731.134222
C 2.416418 0.828630 -0.077401
C 1.941617 1.324011 1.156559
C 1.480754 2.640691 1.318015
C 1.512426 3.485627 0.218713
C 1.988042 3.017511 -1.015447
C 2.435903 1.705572 -1.175283
C 2.814110 -0.574161 0.020931
C 2.659016 -1.072436 1.297339
H 1.119976 2.986814 2.282044
H 1.163692 4.508683 0.314432
H 2.012203 3.689612 -1.868241
H 2.804352 1.365840 -2.134548
S 2.036955 0.117180 2.394065
O -0.493934 -1.094920 1.239136
C -0.534771 -0.501990 0.174981
C -1.026412 0.900994 0.098683
C -0.010854 -1.122191 -1.072053
C -0.898423 1.605435 -1.056496
H -1.460018 1.319331 1.002569
C 0.114128 -0.376309 -2.200074
H 0.264901 -2.172209 -1.020823
C -0.290911 0.985552 -2.177574
H -1.229870 2.634821 -1.131461
H 0.524588 -0.792751 -3.112270
H -0.172297 1.570036 -3.086815
C 3.331006 -1.328641 -1.155692
O 3.235588 -0.839726 -2.273081
C 3.979658 -2.675679 -0.977844
H 3.248754 -3.414307 -0.635495
H 4.380303 -2.990959 -1.941751
H 4.784773 -2.629999 -0.239227
C -3.995211 -0.903002 -0.821044
O -2.780352 -0.972179 -1.111730
O -4.999419 -1.266414 -1.443501
C -4.276578 -0.183484 0.535222
F -4.201417 1.161471 0.377564
F -5.491716 -0.441962 1.040663
F -3.380382 -0.503882 1.488158
C 2.882145 -2.441990 1.868696
H 3.931432 -2.737610 1.798890
H 2.593826 -2.467834 2.921520
H 2.276274 -3.183910 1.340687
42
B_9 Eopt -
1731.133196
C 1.752735 0.355828 1.100437
C 2.711634 1.141568 0.415720
C 4.084135 0.854721 0.430901
C 4.521405 -0.242487 1.162816
C 3.594581 -1.038508 1.842781
C 2.224274 -0.753591 1.817807
C 0.397589 0.867849 0.885986
C 0.381450 1.986244 0.073981
H 4.785394 1.484562 -0.108803
H 5.578522 -0.483573 1.196899
H 3.938951 -1.896708 2.411936
H 1.554025 -1.394293 2.374801
S 1.967718 2.455394 -0.426858
O 1.478689 0.621225 -2.597149
C 1.588765 -0.427482 -1.972480
C 2.911603 -1.066698 -1.795357
C 0.392210 -1.078332 -1.377887
C 3.010422 -2.207526 -1.067699
H 3.761876 -0.592545 -2.276279
C 0.542488 -2.239503 -0.672295
H -0.583344 -0.623337 -1.563856
C 1.838014 -2.763551 -0.471882
H 3.964405 -2.699054 -0.911782
H -0.329270 -2.726495 -0.249384
H 1.947445 -3.666159 0.124706
C -0.866801 0.398316 1.539048
O -1.875553 1.078242 1.440259
C -0.881867 -0.865237 2.353723
H -0.303701 -0.712993 3.272588
H -1.916950 -1.093387 2.607454
H -0.450356 -1.703063 1.802182
C -2.929592 -0.614694 -0.663075
O -2.436018 -1.669577 -0.225122
O -2.538666 0.151371 -1.564153
C -4.294763 -0.218061 -0.023560
F -4.438299 -0.642546 1.242645
F -4.526143 1.104033 -0.024936
F -5.307903 -0.783324 -0.730980
C -0.775479 2.793777 -0.420293
H -1.221624 3.359467 0.402542
H -0.456278 3.488101 -1.201222
H -1.545916 2.123820 -0.815322
35
C_1-notfa Eopt -
1204.934332

C -0.969899 -0.024430 0.520865
C -2.108508 0.108038 -0.317215
C -3.085902 -0.857404 -0.425602
C -2.982508 -2.075176 0.308389
C -1.877285 -2.302264 1.059850
C -0.731227 -1.360072 1.147551
C -0.177338 1.139222 0.509394
C -0.693679 2.123659 -0.355121
H -3.951099 -0.690093 -1.063262
H -3.781679 -2.804084 0.243256
H -1.762341 -3.238627 1.599706
H -0.452840 -1.270329 2.204406
S -2.145981 1.653597 -1.111767
C 1.065304 1.472631 1.297770
O 1.952887 2.097000 0.750798
C 1.107446 1.178823 2.771001
H 0.607203 2.016807 3.273033
H 0.581522 0.268436 3.056610
H 2.145570 1.140050 3.104492
C 0.518667 -2.089620 0.453100
C 0.365591 -2.074597 -1.036827
C 1.808538 -1.518072 0.947853
C 1.277394 -1.533306 -1.854517
H -0.540567 -2.520749 -1.443656
C 2.732800 -0.996334 0.132180
H 1.978754 -1.557404 2.022110
C 2.518749 -0.926139 -1.329740
H 1.146159 -1.523713 -2.932704
H 3.667982 -0.592112 0.509024
H 0.457860 -3.127762 0.806425
O 3.338670 -0.398830 -2.071432
C -0.130918 3.472914 -0.642440
H 0.214324 3.943962 0.281419
H 0.737863 3.370408 -1.300356
H -0.868824 4.115063 -1.126109
42
C_1 Eopt -
1731.150343
C 0.164410 0.379772 -0.333406
C -0.046428 0.875971 -1.648379
C 0.536071 0.307575 -2.754372
C 1.408655 -0.819141 -2.615506
C 1.707445 -1.291277 -1.382582
C 1.185740 -0.686811 -0.126194
C -0.609659 1.051378 0.617062
C -1.398817 2.066464 0.036119
H 0.327952 0.697744 -3.747603
H 1.828814 -1.270242 -3.506677
H 2.395350 -2.125318 -1.268630
H 0.757588 -1.492657 0.487124
S -1.188111 2.191095 -1.648725
C -0.691879 0.772377 2.093340
O -0.850692 1.705173 2.858165
C -0.571465 -0.643481 2.595251
H -1.163719 -0.726368 3.507902
H -0.927215 -1.372583 1.866504
H 0.473700 -0.858030 2.849260
C 2.410677 -0.124681 0.713783
C 3.272685 -1.251479 1.190598
C 3.148395 0.940473 -0.038254
C 4.595362 -1.293392 0.988595
H 2.770745 -2.051363 1.732707
C 4.470187 0.899687 -0.246565
H 2.562252 1.780787 -0.407414
C 5.294370 -0.223582 0.246012
H 5.206329 -2.112082 1.357696
H 4.989602 1.688296 -0.783222
H 1.961454 0.346846 1.600721
O 6.503242 -0.266951 0.046896
C -3.071731 -1.800447 -0.210766
C -3.435964 -0.350088 0.247331
O -3.304613 -0.134908 1.465689
O -3.750485 0.400133 -0.691304
F -1.820366 -1.822718 -0.743887
F -3.894750 -2.273757 -1.163045
F -3.071347 -2.703149 0.784730
C -2.373416 2.947488 0.728973
H -1.841250 3.666044 1.359436
H -3.004216 3.479943 0.015858
H -2.996905 2.319679 1.373964
42
C_10 Eopt -
1731.149386
C 0.299496 -1.691967 0.205114
C 0.204422 -2.265919 -1.090395
C 1.299908 -2.692260 -1.806359
C 2.615909 -2.552046 -1.272274
C 2.782378 -1.933513 -0.078316
C 1.663454 -1.342463 0.700728
C -0.965300 -1.505327 0.795728
C -2.006785 -1.876671 -0.080673
H 1.168723 -3.156456 -2.780923
H 3.461024 -2.923987 -1.839361
H 3.781003 -1.774463 0.321692
H 1.777504 -1.629925 1.751927
S -1.450396 -2.487361 -1.567269
C -1.291653 -1.016214 2.178448
O -2.447451 -0.759335 2.461534
C -0.217624 -0.933599 3.231724
H 0.336847 -1.874700 3.298645
H 0.486135 -0.124667 3.015294
H -0.699440 -0.725446 4.187511
C 1.892841 0.242343 0.677865
C 3.053748 0.599091 1.547333
C 1.971953 0.781728 -0.714947
C 4.064424 1.373355 1.130847
H 3.038395 0.214537 2.566318
C 2.982142 1.556393 -1.135037
H 1.126224 0.558303 -1.364707
C 4.110691 1.900072 -0.249346
H 4.887470 1.644763 1.785824
H 3.006259 1.968166 -2.140042
H 0.972289 0.673248 1.103872
O 5.047989 2.591989 -0.636673
C -2.643936 2.443649 -0.527495
C -1.447215 1.457112 -0.348375
O -0.911779 1.545912 0.773767
O -1.195306 0.727368 -1.322033
F -3.231808 2.371733 -1.733071
F -2.254941 3.725615 -0.362119
F -3.610822 2.215158 0.387381
C -3.478457 -1.787312 0.127060
H -3.763479 -0.736225 0.238032
H -4.021345 -2.222685 -0.713863
H -3.763130 -2.291457 1.053821
42
C_11 Eopt -
1731.155322
C 0.339207 -1.699419 0.206020
C 0.143869 -2.330553 -1.044264
C 1.188481 -2.750648 -1.840321
C 2.537419 -2.558839 -1.424062
C 2.788416 -1.913941 -0.255891
C 1.728041 -1.363274 0.621948
C -0.876048 -1.462117 0.883034
C -1.981277 -1.878062 0.125688
H 0.988247 -3.242464 -2.789506
H 3.342536 -2.916465 -2.055032
H 3.814802 -1.728374 0.052899
H 1.882355 -1.724213 1.648810
S -1.542325 -2.579286 -1.368359
C -0.915031 -0.852251 2.252626
O 0.119612 -0.821725 2.901608
C -2.194526 -0.321314 2.831595
H -2.764395 0.248274 2.096954
H -2.807157 -1.153662 3.195220
H -1.939913 0.319519 3.676831
C 1.945008 0.213126 0.718338
C 3.158781 0.520572 1.532280
C 1.930428 0.842056 -0.638544
C 4.140833 1.321890 1.098099
H 3.204838 0.077034 2.525633
C 2.913714 1.640775 -1.077652
H 1.047856 0.646080 -1.248168
C 4.097291 1.932666 -0.247177
H 5.004367 1.557735 1.713639
H 2.876540 2.108535 -2.057515
H 1.061199 0.591299 1.249051
O 5.008459 2.650361 -0.651038
C -2.685905 2.358498 -0.631327
C -1.478791 1.384804 -0.453892
O -0.932448 1.489207 0.661921
O -1.241141 0.635336 -1.415599
F -3.242940 2.320320 -1.852056
F -2.336128 3.639787 -0.402923
F -3.670789 2.065955 0.250772
C -3.441452 -1.776332 0.421478
H -3.753477 -0.727131 0.371459
H -4.026471 -2.336844 -0.310007
H -3.672016 -2.156564 1.418351
42
C_12 Eopt -
1731.152983
C 0.153275 -0.026337 -0.363313
C -0.180956 -1.031771 -1.297268
C 0.305509 -2.318573 -1.203007
C 1.245178 -2.663607 -0.188904
C 1.685755 -1.709046 0.667638
C 1.240599 -0.291936 0.619286
C -0.606655 1.144318 -0.552120
C -1.491913 1.030548 -1.634218
H -0.034720 -3.083985 -1.896036
H 1.599156 -3.685510 -0.123204
H 2.431006 -1.954687 1.420359
H 0.880157 -0.034267 1.624948
S -1.408826 -0.492735 -2.404587
C -0.637193 2.362935 0.324472
O -0.879959 3.447632 -0.173689
C -0.432339 2.196424 1.806858
H -0.921895 3.027972 2.316982
H -0.831532 1.238832 2.154245
H 0.639307 2.242920 2.037041
C 2.496514 0.634953 0.343764
C 3.435256 0.600332 1.509414
C 3.138667 0.313651 -0.971409
C 4.742497 0.339005 1.386718
H 3.004240 0.814522 2.486084
C 4.444107 0.044605 -1.097590

H 2.493475 0.317615 -1.848856
C 5.346171 0.035661 0.072511
H 5.409605 0.336623 2.243957
H 4.892938 -0.176017 -2.061776
H 2.095085 1.657759 0.275802
O 6.541276 -0.213700 -0.041435
C -3.501669 -0.421581 0.950412
C -2.297736 -1.418536 1.042977
O -1.381694 -1.048135 1.803079
O -2.435764 -2.448171 0.365757
F -3.160196 0.855099 1.219510
F -4.463099 -0.757129 1.839414
F -4.086052 -0.409779 -0.262259
C -2.474121 2.039691 -2.122320
H -1.948543 2.845533 -2.643477
H -3.200199 1.588429 -2.800813
H -2.999295 2.488877 -1.274973
42
C_13 Eopt -
1731.152535
C 0.172560 0.659262 -0.092331
C 0.136280 1.676888 -1.070937
C 0.777925 1.560195 -2.285815
C 1.506795 0.380286 -2.603289
C 1.632136 -0.599446 -1.670777
C 1.095867 -0.487612 -0.292173
C -0.697746 0.923430 0.987966
C -1.348881 2.160740 0.841497
H 0.705001 2.357261 -3.022038
H 1.956676 0.283992 -3.584337
H 2.218077 -1.488423 -1.892292
H 0.579502 -1.416887 -0.019162
S -0.901000 2.983215 -0.583069
C -0.972009 -0.099022 2.046721
O -0.168911 -1.002909 2.219308
C -2.246795 -0.045445 2.841127
H -2.390217 -1.014735 3.319669
H -2.168453 0.722944 3.617556
H -3.106413 0.191709 2.209591
C 2.338222 -0.393058 0.702826
C 3.060520 -1.701970 0.746379
C 3.217020 0.782050 0.410129
C 4.384828 -1.812792 0.578266
H 2.452099 -2.582723 0.944735
C 4.541540 0.677154 0.240233
H 2.737876 1.758629 0.356425
C 5.226159 -0.629951 0.307278
H 4.891864 -2.771666 0.635508
H 5.164882 1.545311 0.046060
H 1.874342 -0.245544 1.687084
O 6.438568 -0.727341 0.144813
C -3.314375 -1.722621 -0.488667
C -2.455866 -0.653004 -1.236481
O -1.394449 -1.120553 -1.692915
O -2.916784 0.498828 -1.245134
F -2.660356 -2.175496 0.607198
F -3.565034 -2.801696 -1.257484
F -4.504843 -1.269243 -0.060964
C -2.347714 2.823778 1.731266
H -2.044298 2.758264 2.777887
H -2.467161 3.875731 1.465525
H -3.321865 2.335263 1.620265
42
C_14 Eopt -
1731.148807
C 0.531030 0.681791 0.074867
C 0.538709 1.527320 1.221065
C 0.561908 2.892500 1.135324
C 0.636840 3.533177 -0.147101
C 0.762723 2.783242 -1.264752
C 0.864876 1.296116 -1.243229
C 0.318771 -0.656322 0.408424
C 0.211183 -0.846767 1.802638
H 0.500382 3.503259 2.032644
H 0.601725 4.615246 -0.196762
H 0.860100 3.256088 -2.238314
H 0.253967 0.880082 -2.052545
S 0.324839 0.603974 2.681359
C 0.020951 -1.806287 -0.509373
O 0.395931 -2.926040 -0.216842
C -0.820853 -1.565641 -1.739096
H -1.284612 -0.578476 -1.756508
H -0.205840 -1.696559 -2.635217
H -1.600671 -2.331322 -1.756144
C 2.384262 0.929651 -1.628033
C 3.309716 1.268954 -0.500383
C 2.479493 -0.489183 -2.087641
C 4.081706 0.358585 0.107328
H 3.323925 2.305669 -0.168999
C 3.252466 -1.403585 -1.488343
H 1.883664 -0.755298 -2.957998
C 4.082058 -1.057124 -0.315022
H 4.736804 0.622762 0.932535
H 3.304094 -2.430424 -1.838910
H 2.612688 1.577093 -2.485809
O 4.751759 -1.901552 0.269324
C -4.042186 -0.093708 -0.600448
C -2.846434 -0.100330 0.407671
O -2.563457 -1.206588 0.899766
O -2.345645 1.025981 0.578004
F -4.474409 -1.317615 -0.944684
F -3.729234 0.539968 -1.752423
F -5.105503 0.561358 -0.083337
C -0.011922 -2.127450 2.522488
H 0.877853 -2.757891 2.432889
H -0.231565 -1.958845 3.578130
H -0.848106 -2.652422 2.052953
42
C_15 Eopt -
1731.152214
C 0.430994 0.774602 0.130628
C 0.590048 1.833749 1.062075
C 0.641153 3.150405 0.681519
C 0.557056 3.502529 -0.702882
C 0.501362 2.527253 -1.640828
C 0.564151 1.075764 -1.321265
C 0.251890 -0.466477 0.759645
C 0.256961 -0.355310 2.157588
H 0.714670 3.938296 1.427233
H 0.540343 4.550377 -0.978909
H 0.466249 2.783293 -2.696727
H -0.213039 0.545206 -1.883097
S 0.510610 1.243629 2.699403
C -0.150844 -1.704254 0.013697
O -0.798256 -1.576579 -1.010673
C 0.240031 -3.059060 0.531247
H 1.225262 -3.042310 1.004841
H -0.496486 -3.387806 1.272745
H 0.227822 -3.765551 -0.300448
C 1.969700 0.533268 -1.871320
C 3.094195 1.026681 -1.012964
C 1.946401 -0.950930 -2.046958
C 3.943123 0.207532 -0.378367
H 3.189703 2.105136 -0.899064
C 2.803163 -1.774486 -1.427423
H 1.189301 -1.346919 -2.719380
C 3.833573 -1.260045 -0.503575
H 4.741261 0.587187 0.253227
H 2.774836 -2.849888 -1.580520
H 2.061064 0.980222 -2.871467
O 4.570867 -2.015265 0.122472
C -3.625445 -0.420087 -0.751137
C -2.699825 0.300153 0.279261
O -2.620350 -0.258283 1.387026
O -2.203204 1.363574 -0.139148
F -3.724602 -1.745780 -0.558083
F -3.254657 -0.236875 -2.030177
F -4.887535 0.073313 -0.649508
C -0.005088 -1.416078 3.170611
H 0.762265 -2.193967 3.122673
H -0.021349 -1.004394 4.180949
H -0.978560 -1.871714 2.962683
42
C_16 Eopt -
1731.146198
C 0.434008 -1.370567 0.675904
C 0.863594 -0.751105 1.880060
C 0.056836 -0.623487 2.987830
C -1.281450 -1.121066 2.972610
C -1.775352 -1.662977 1.833290
C -1.012400 -1.728547 0.558220
C 1.488401 -1.524886 -0.243712
C 2.702392 -1.000994 0.252031
H 0.441705 -0.161764 3.894353
H -1.885307 -1.043053 3.868895
H -2.805804 -2.008621 1.791623
H -1.115361 -2.740720 0.148614
S 2.553363 -0.340157 1.815935
C 1.467620 -2.199488 -1.585974
O 2.377836 -1.995668 -2.366484
C 0.376363 -3.181246 -1.926432
H 0.240703 -3.909691 -1.120995
H -0.574395 -2.668500 -2.099849
H 0.660347 -3.698033 -2.843855
C -1.775832 -0.771441 -0.471232
C -3.006027 -1.444584 -0.991733
C -2.023480 0.580814 0.121468
C -4.209990 -0.858634 -0.999232
H -2.878729 -2.444641 -1.404138
C -3.226321 1.170524 0.118784
H -1.162300 1.087963 0.549023
C -4.410421 0.498144 -0.446868
H -5.084868 -1.351999 -1.412925
H -3.376226 2.162759 0.534666
H -1.074674 -0.616514 -1.305187
O -5.516027 1.030745 -0.451222
C 0.906982 2.881060 -0.289468
C 1.327406 1.677485 -1.194886
O 0.367523 1.028934 -1.650783
O 2.553590 1.509565 -1.281993
F 0.742427 2.457786 0.993210
F -0.259652 3.442860 -0.652134
F 1.816396 3.867931 -0.247179
C 4.045946 -1.008616 -0.386553
H 4.295347 -2.020615 -0.718159
H 4.009141 -0.363240 -1.267122
H 4.811039 -0.648937 0.304248
42

C_17 Eopt -
1731.155071
C -0.345059 -1.489476 -0.363175
C -0.795827 -1.395161 -1.700338
C 0.029796 -1.615929 -2.783458
C 1.396387 -1.968613 -2.591532
C 1.894774 -2.044191 -1.330371
C 1.100218 -1.748509 -0.114091
C -1.394520 -1.337994 0.568766
C -2.631854 -1.131475 -0.062811
H -0.362621 -1.540979 -3.794828
H 2.019526 -2.160694 -3.456939
H 2.944921 -2.280162 -1.171263
H 1.175263 -2.599056 0.579422
S -2.507957 -1.101120 -1.769585
C -1.169559 -1.455052 2.049261
O -0.137021 -1.982337 2.434329
C -2.188942 -0.939399 3.019285
H -2.536746 0.050576 2.712526
H -3.044019 -1.622518 3.062359
H -1.725204 -0.893605 4.005621
C 1.812635 -0.547064 0.647295
C 3.042863 -1.025882 1.346808
C 2.040095 0.605877 -0.277304
C 4.236197 -0.433475 1.206614
H 2.921346 -1.875522 2.016610
C 3.232242 1.198538 -0.427056
H 1.172787 0.962795 -0.827759
C 4.421153 0.723838 0.305943
H 5.113447 -0.774545 1.748968
H 3.370583 2.047021 -1.091120
H 1.093767 -0.211722 1.406070
O 5.518153 1.258136 0.170561
C -1.085254 2.674193 -0.415001
C -1.442473 1.861404 0.868944
O -0.456059 1.389721 1.463844
O -2.661141 1.743669 1.079725
F -0.932850 1.820971 -1.463653
F 0.067761 3.357076 -0.310991
F -2.029245 3.555958 -0.777771
C -3.996497 -1.005480 0.529506
H -4.217848 -1.881386 1.146270
H -4.043259 -0.107391 1.149572
H -4.755322 -0.931360 -0.251789
42
C_18 Eopt -
1731.149586
C 0.279858 -1.629298 0.295842
C 0.206393 -2.285979 -0.961181
C 1.308937 -2.807927 -1.598980
C 2.609200 -2.678259 -1.024638
C 2.759741 -1.964695 0.117736
C 1.636876 -1.266086 0.795306
C -0.994506 -1.379933 0.840551
C -2.019745 -1.749360 -0.055927
H 1.195239 -3.338993 -2.540971
H 3.457682 -3.128421 -1.526080
H 3.750140 -1.802379 0.536561
H 1.714076 -1.425996 1.875473
S -1.439375 -2.461856 -1.488601
C -1.344472 -0.826507 2.193475
O -2.517103 -0.763960 2.518763
C -0.268835 -0.441166 3.172740
H 0.391967 0.325708 2.765433
H -0.755111 -0.051027 4.067359
H 0.324867 -1.320572 3.445323
C 1.906796 0.305265 0.586843
C 2.994444 0.749246 1.510020
C 2.133624 0.661946 -0.848296
C 4.057496 1.454009 1.100628
H 2.878232 0.491468 2.562023
C 3.193653 1.371356 -1.261003
H 1.352914 0.366222 -1.545915
C 4.240317 1.813387 -0.320954
H 4.823266 1.791089 1.793421
H 3.321410 1.652218 -2.302591
H 0.961546 0.800460 0.871824
O 5.221132 2.448425 -0.697778
C -2.738691 2.236693 -0.622816
C -1.411934 1.468912 -0.328580
O -0.968502 1.640397 0.824358
O -0.963568 0.816021 -1.285564
F -3.470949 1.672105 -1.599545
F -2.470906 3.501419 -1.022039
F -3.539822 2.334844 0.453111
C -3.490005 -1.559629 0.083433
H -3.868940 -2.151555 0.921086
H -3.700384 -0.512151 0.320375
H -4.012360 -1.840597 -0.832865
42
C_19 Eopt -
1731.154817
C -0.724578 -1.701187 0.042166
C -1.078366 -2.059494 -1.281500
C -0.258432 -2.808805 -2.097499
C 1.003633 -3.272572 -1.624379
C 1.417428 -2.925164 -0.379216
C 0.638804 -2.049002 0.529909
C -1.757404 -1.010745 0.707679
C -2.886133 -0.841120 -0.112679
H -0.574568 -3.063730 -3.106278
H 1.619444 -3.883674 -2.273515
H 2.393360 -3.244507 -0.020778
H 0.551129 -2.527220 1.515273
S -2.682151 -1.512475 -1.672752
C -1.638293 -0.578082 2.141457
O -0.762257 -1.085691 2.824493
C -2.595694 0.419665 2.722722
H -3.528576 -0.085336 2.997531
H -2.142636 0.833022 3.624697
H -2.809025 1.217137 2.009700
C 1.501058 -0.740201 0.799574
C 2.696425 -1.058063 1.635893
C 1.812970 -0.022175 -0.474246
C 3.936994 -0.673612 1.307385
H 2.511229 -1.599300 2.562327
C 3.051278 0.361369 -0.811199
H 0.970193 0.208395 -1.125720
C 4.208379 0.063111 0.055857
H 4.791030 -0.883592 1.944869
H 3.252838 0.909049 -1.727474
H 0.846020 -0.077610 1.382086
O 5.346869 0.404012 -0.251411
C -0.048874 3.184523 -0.681610
C -0.896976 2.179668 0.161045
O -0.331927 1.701871 1.161286
O -2.036414 1.989344 -0.301098
F -0.677491 4.371303 -0.814635
F 0.156582 2.718342 -1.934906
F 1.161710 3.443752 -0.165218
C -4.198176 -0.200333 0.186205
H -4.599348 -0.560378 1.135718
H -4.044390 0.881696 0.249133
H -4.923916 -0.407899 -0.602260
35
C_2-notfa Eopt -
1204.938502
C -0.948901 0.198691 0.546453
C -1.983440 0.593064 -0.333412
C -3.168488 -0.105254 -0.449116
C -3.378486 -1.296274 0.304748
C -2.373975 -1.781057 1.078926
C -1.036674 -1.145225 1.178570
C 0.106315 1.130687 0.579927
C -0.101697 2.195016 -0.309755
H -3.956056 0.254833 -1.107187
H -4.331022 -1.807386 0.232443
H -2.502957 -2.713057 1.623147
H -0.715724 -1.121312 2.226111
S -1.593444 2.084618 -1.139194
C 1.231646 1.050465 1.575672
O 0.993671 0.566121 2.667539
C 2.594383 1.567599 1.217270
H 2.824823 1.402216 0.161813
H 2.638173 2.643974 1.416610
H 3.332155 1.072462 1.850884
C -0.004247 -2.148161 0.441179
C -0.199698 -2.090844 -1.043182
C 1.398889 -1.888588 0.886247
C 0.772892 -1.734159 -1.891705
H -1.191412 -2.334732 -1.420310
C 2.380710 -1.555168 0.038032
H 1.599519 -1.995285 1.949792
C 2.126619 -1.390631 -1.408631
H 0.607383 -1.683051 -2.964024
H 3.399317 -1.390070 0.378299
H -0.290606 -3.143431 0.807864
O 2.999536 -0.992650 -2.171695
C 0.772453 3.374953 -0.574063
H 1.014588 3.884024 0.363387
H 1.708000 3.051260 -1.040955
H 0.283470 4.087233 -1.240480
42
C_2 Eopt -
1731.152482
C 0.118747 0.557456 -0.065367
C -0.016839 1.602938 -1.008008
C 0.604746 1.580529 -2.236738
C 1.435786 0.480958 -2.604084
C 1.669438 -0.510188 -1.707290
C 1.128198 -0.503474 -0.324671
C -0.753561 0.707286 1.032673
C -1.503581 1.899511 0.942683
H 0.447420 2.389426 -2.946251
H 1.880268 0.463031 -3.592118
H 2.337293 -1.329241 -1.962360
H 0.696890 -1.484572 -0.083281
S -1.157115 2.793897 -0.462812
C -0.930837 -0.371528 2.059452
O -0.088869 -1.254645 2.133270
C -2.155971 -0.399930 2.926274
H -2.191749 -1.366714 3.429754
H -2.104966 0.393000 3.679159
H -3.054873 -0.254693 2.319381
C 2.362840 -0.361370 0.676535
C 3.194012 -1.604169 0.641936
C 3.132691 0.900812 0.446955
C 4.520569 -1.590159 0.458114

H 2.665520 -2.543558 0.795351
C 4.458884 0.920140 0.260946
H 2.569490 1.832931 0.456226
C 5.253246 -0.325396 0.245511
H 5.109403 -2.502866 0.458012
H 5.003578 1.848438 0.114383
H 1.892638 -0.309555 1.667006
O 6.466274 -0.309119 0.063420
C -2.773401 -1.772294 -0.718037
C -3.137255 -0.256686 -0.854926
O -3.835930 0.199935 0.068128
O -2.684740 0.267133 -1.886011
F -1.500478 -2.030480 -1.088858
F -3.563274 -2.531558 -1.509092
F -2.907601 -2.248728 0.532516
C -2.522435 2.455845 1.877974
H -2.151356 2.447146 2.905178
H -2.781860 3.480912 1.606869
H -3.427229 1.842763 1.816540
42
C_20 Eopt -1731.151886
C 0.305667 0.478259 -0.015600
C -0.172162 1.052643 1.183376
C -0.521325 2.383713 1.278551
C -0.296427 3.265613 0.180929
C 0.292196 2.793678 -0.943884
C 0.767386 1.392478 -1.098087
C 0.394160 -0.924517 0.069536
C 0.041705 -1.401625 1.338367
H -0.973719 2.767831 2.188737
H -0.610666 4.299405 0.261871
H 0.496058 3.460743 -1.777690
H 0.466744 1.027032 -2.086114
S -0.448843 -0.156957 2.403529
C 0.596648 -1.893759 -1.057857
O 1.175000 -2.946492 -0.861314
C -0.047030 -1.571215 -2.383112
H 0.702150 -1.160762 -3.068746
H -0.411860 -2.505260 -2.816863
H -0.865841 -0.854173 -2.272808
C 2.372122 1.441047 -1.106948
C 2.896680 1.716607 0.269293
C 2.931580 0.204126 -1.732950
C 3.708206 0.874538 0.922470
H 2.570216 2.639799 0.744927
C 3.748885 -0.638609 -1.088850
H 2.647456 0.012792 -2.765457
C 4.162836 -0.389455 0.307807
H 4.063598 1.085290 1.927091
H 4.140636 -1.533397 -1.564080
H 2.618697 2.288805 -1.760967
O 4.864987 -1.185927 0.921183
C -3.577158 -0.499338 -0.300188
C -2.885956 0.895897 -0.428782
O -3.169137 1.726232 0.448416
O -2.104200 0.958501 -1.398874
F -4.514988 -0.558422 0.657221
F -2.666273 -1.460025 -0.008452
F -4.169446 -0.876986 -1.450937
C 0.005813 -2.817961 1.799826
H -0.507009 -2.911468 2.758447
H -0.501513 -3.439059 1.055949
H 1.026743 -3.198603 1.900066
42
C_21 Eopt -1731.151563
C 0.352906 0.720287 0.091101
C 0.322926 1.667201 1.142951
C 0.371074 3.024481 0.920161
C 0.468680 3.530766 -0.408972
C 0.597773 2.665019 -1.445800
C 0.692556 1.191906 -1.278732
C 0.150925 -0.593040 0.552766
C -0.009775 -0.641814 1.946983
H 0.305964 3.720333 1.753214
H 0.440167 4.601869 -0.570711
H 0.705240 3.038932 -2.460951
H 0.049225 0.700318 -2.015968
S 0.085548 0.893516 2.681265
C -0.035714 -1.756977 -0.377482
O -0.494352 -1.545538 -1.485750
C 0.341745 -3.143461 0.061811
H -0.472345 -3.573391 0.655726
H 0.483515 -3.761298 -0.826322
H 1.248654 -3.139679 0.672785
C 2.204129 0.780693 -1.640865
C 3.140804 1.210689 -0.553642
C 2.300573 -0.670551 -1.986085
C 3.919985 0.354762 0.120753
H 3.154764 2.270823 -0.307071
C 3.090423 -1.528527 -1.325646
H 1.691442 -1.011151 -2.819670
C 3.919286 -1.090219 -0.185455
H 4.580026 0.686614 0.917171
H 3.152275 -2.577525 -1.602354
H 2.430139 1.350668 -2.553100
O 4.585088 -1.886322 0.469543
C -3.532356 -0.524180 -0.646710
C -2.720734 0.710231 -0.137603
O -2.703642 0.795039 1.103190
O -2.216979 1.430129 -1.017651
F -4.845423 -0.392805 -0.341806
F -3.123574 -1.670321 -0.058097
F -3.468808 -0.723355 -1.971130
C -0.307631 -1.821902 2.811028
H -0.458624 -1.522722 3.849374
H -1.217977 -2.316297 2.456826
H 0.512662 -2.544064 2.772091
42
C_22 Eopt -1731.151421
C -0.319767 0.423576 0.095242
C 0.306995 0.919682 -1.080366
C 0.751019 2.213015 -1.196263
C 0.554182 3.138815 -0.124247
C -0.147152 2.754711 0.968728
C -0.780860 1.414779 1.106909
C -0.509760 -0.963114 0.048025
C -0.062927 -1.517937 -1.164371
H 1.282729 2.533415 -2.088909
H 0.965761 4.137853 -0.207005
H -0.336540 3.458231 1.775325
H -0.625015 1.048179 2.126972
S 0.601750 -0.359326 -2.226829
C -0.921227 -1.876625 1.168216
O -1.531546 -2.898481 0.912242
C -0.479282 -1.545489 2.569420
H 0.417551 -0.921888 2.565062
H -1.288838 -1.031769 3.098839
H -0.285624 -2.485737 3.090548
C -2.369555 1.635054 0.950102
C -2.725566 1.903346 -0.480514
C -3.114109 0.488241 1.554171
C -3.534823 1.107150 -1.191559
H -2.276245 2.778942 -0.945209
C -3.925325 -0.311851 0.850629
H -2.969426 0.326347 2.619852
C -4.155821 -0.092404 -0.592914
H -3.766265 1.309190 -2.233482
H -4.449925 -1.144559 1.310449
H -2.583696 2.530762 1.548541
O -4.847575 -0.856832 -1.256443
C 3.805935 0.012428 0.061415
C 2.568344 -0.452676 0.895484
O 2.053291 0.470038 1.560353
O 2.266314 -1.651563 0.790986
F 3.518469 1.085672 -0.710177
F 4.290999 -0.929916 -0.762043
F 4.823120 0.386092 0.867347
C -0.060839 -2.952584 -1.557745
H 0.510067 -3.114505 -2.473310
H 0.372196 -3.549088 -0.749165
H -1.088686 -3.296777 -1.704645
42
C_23 Eopt -1731.150207
C -0.586155 0.701560 0.270073
C -0.057093 1.501386 -0.773421
C -0.115709 2.874634 -0.762587
C -0.767812 3.558423 0.309579
C -1.399409 2.841369 1.270697
C -1.471563 1.357343 1.269799
C -0.255647 -0.658728 0.129888
C 0.471624 -0.898735 -1.050172
H 0.351545 3.451985 -1.556824
H -0.759358 4.641955 0.327580
H -1.938151 3.346535 2.068129
H -1.277246 0.969121 2.274976
S 0.785448 0.526378 -1.940445
C -0.471371 -1.655155 1.235298
O -0.738019 -1.243369 2.350497
C -0.373920 -3.126989 0.960998
H -0.941803 -3.401033 0.066887
H 0.673587 -3.403723 0.808638
H -0.760695 -3.663313 1.828330
C -3.009212 0.980308 0.947987
C -3.329293 1.288066 -0.483673
C -3.317961 -0.425483 1.350286
C -3.739130 0.357617 -1.355401
H -3.195785 2.318048 -0.809289
C -3.738208 -1.357680 0.484033
H -3.184727 -0.669572 2.400365
C -3.925499 -1.050149 -0.947296
H -3.950033 0.598622 -2.393420
H -3.962182 -2.372532 0.801558
H -3.586893 1.649844 1.599686
O -4.236890 -1.917008 -1.757854
C 3.983911 -0.006995 -0.017341
C 2.746175 -0.153536 0.927959
O 2.213015 0.909954 1.281851
O 2.470706 -1.342672 1.179810
F 4.214062 1.251107 -0.428806
F 3.847733 -0.756643 -1.135270
F 5.115499 -0.428960 0.587743
C 1.043154 -2.170074 -1.581752
H 1.536928 -2.005664 -2.541151

H 1.781276 -2.551327 -0.868980
H 0.258925 -2.918866 -1.716510
42
C_24 Eopt -1731.151169
C 0.271616 -0.315053 0.073827
C -0.388143 -0.676753 -1.132591
C -0.981226 -1.903266 -1.314727
C -0.917645 -2.892385 -0.285413
C -0.201152 -2.642857 0.837377
C 0.584495 -1.396900 1.047938
C 0.637552 1.035266 0.089132
C 0.307821 1.687645 -1.113717
H -1.527834 -2.119008 -2.230076
H -1.445886 -3.828939 -0.420342
H -0.118331 -3.397801 1.615115
H 0.447076 -1.059615 2.080191
S -0.470631 0.668779 -2.235705
C 1.134226 1.839961 1.257017
O 1.884688 2.778818 1.063864
C 0.591338 1.516597 2.623167
H 0.589991 2.434034 3.215305
H -0.415067 1.093510 2.553196
H 1.246065 0.795935 3.124143
C 2.138850 -1.800016 0.907433
C 2.484335 -2.065911 -0.526241
C 3.004079 -0.769184 1.557913
C 3.394727 -1.351339 -1.200774
H 1.940691 -2.866583 -1.024360
C 3.918241 -0.053011 0.891052
H 2.865076 -0.624029 2.626626
C 4.145053 -0.254364 -0.555610
H 3.617677 -1.548287 -2.245550
H 4.532430 0.695006 1.384280
H 2.233628 -2.732429 1.479861
O 4.934776 0.441125 -1.184820
C -3.792036 -0.049639 0.063824
C -2.588882 0.492113 0.903115
O -2.092478 -0.320442 1.706697
O -2.302302 1.674318 0.650928
F -3.971902 -1.378263 0.168197
F -3.652594 0.213833 -1.253224
F -4.950531 0.528729 0.449083
C 0.521477 3.120652 -1.453409
H 1.592514 3.320347 -1.551721
H 0.015385 3.390199 -2.381548
H 0.146107 3.748628 -0.639581
42
C_25 Eopt -1731.148927
C -0.343598 -0.271545 -0.120875
C 0.216466 -0.822408 1.061427
C 0.689720 -2.112049 1.121946
C 0.577475 -2.971929 -0.013233
C -0.092340 -2.540990 -1.110667
C -0.757142 -1.213663 -1.193395
C -0.445414 1.129694 -0.060289
C -0.066255 1.623996 1.202888
H 1.184561 -2.474200 2.019962
H 1.017856 -3.961222 0.027476
H -0.237600 -3.203665 -1.960112
H -0.604563 -0.766429 -2.180235
S 0.431602 0.400468 2.283276
C -0.635954 1.952115 -1.300720
O -1.114690 1.429359 -2.292500
C -0.141408 3.369979 -1.343567
H -0.194473 3.714891 -2.376704
H -0.760967 4.018515 -0.717871
H 0.891466 3.408873 -0.985533
C -2.341028 -1.505092 -1.076363
C -2.702152 -1.955119 0.307329
C -3.148945 -0.345529 -1.565041
C -3.593911 -1.314972 1.074624
H -2.199920 -2.842514 0.686971
C -4.048425 0.292103 -0.802729
H -2.985989 -0.042643 -2.593680
C -4.301391 -0.110869 0.592333
H -3.833663 -1.655332 2.078109
H -4.633578 1.123013 -1.186852
H -2.508607 -2.338768 -1.772638
O -5.077467 0.507954 1.314711
C 3.725312 -0.373325 -0.065230
C 2.611482 0.472818 -0.763428
O 1.977030 -0.109044 -1.660956
O 2.516000 1.626275 -0.305858
F 3.781707 -1.649891 -0.481726
F 3.554398 -0.416302 1.275355
F 4.949285 0.156721 -0.275944
C -0.031481 3.029110 1.699042
H -0.943788 3.560843 1.420562
H 0.076218 3.058395 2.784738
H 0.825857 3.547714 1.256744
42
C_26 Eopt -1731.149413
C 0.322349 -1.656515 0.200132
C 0.200527 -2.220110 -1.097746
C 1.281003 -2.636748 -1.841426
C 2.607288 -2.505124 -1.331518
C 2.798234 -1.907883 -0.130512
C 1.696936 -1.326686 0.681109
C -0.931593 -1.462089 0.810432
C -1.990359 -1.822249 -0.049612
H 1.130019 -3.087575 -2.819390
H 3.440546 -2.869796 -1.920478
H 3.805000 -1.761676 0.253363
H 1.824353 -1.646257 1.722170
S -1.463155 -2.435299 -1.545404
C -1.231987 -0.975889 2.199923
O -2.369349 -0.648415 2.483808
C -0.155969 -0.982120 3.254466
H -0.627034 -0.783404 4.217670
H 0.353745 -1.949834 3.288197
H 0.585684 -0.200283 3.065438
C 1.940789 0.254768 0.702623
C 3.141221 0.571650 1.534123
C 1.967099 0.836906 -0.675193
C 4.145689 1.341943 1.096466
H 3.162280 0.158855 2.541959
C 2.972580 1.605942 -1.116302
H 1.087822 0.648814 -1.291539
C 4.143850 1.904419 -0.270814
H 4.999479 1.583564 1.723178
H 2.960687 2.047992 -2.108624
H 1.045098 0.681025 1.179681
O 5.077391 2.589695 -0.678451
C -2.756268 2.311770 -0.494398
C -1.461198 1.447323 -0.374674
O -1.210618 0.750602 -1.374411
O -0.840864 1.604632 0.692649
F -3.362707 2.520038 0.687360
F -3.680487 1.775127 -1.312184
F -2.460376 3.533338 -0.997532
C -3.456708 -1.720422 0.187427
H -4.020192 -2.165808 -0.634482
H -3.725233 -2.207226 1.128364
H -3.733015 -0.665570 0.286353
42
C_27 Eopt -1731.150834
C -0.917321 1.708239 0.383608
C -1.337341 2.585749 -0.641382
C -2.653513 2.684252 -1.047587
C -3.661085 1.891923 -0.430592
C -3.308664 0.994827 0.527577
C -1.910844 0.762585 0.959463
C 0.455167 1.851267 0.683675
C 1.069890 2.813276 -0.126062
H -2.931457 3.378020 -1.837133
H -4.691320 2.003983 -0.747055
H -4.064749 0.355600 0.977678
H -1.853580 0.809045 2.056004
S -0.014449 3.546642 -1.231979
C 1.118583 1.049853 1.765873
O 0.408711 0.512997 2.601037
C 2.613877 0.923317 1.839648
H 2.847198 0.051612 2.453420
H 3.065244 0.821505 0.852056
H 3.030946 1.811822 2.327343
C -1.544040 -0.749680 0.608365
C -2.303558 -1.689757 1.485678
C -1.686313 -1.019589 -0.854998
C -3.006918 -2.724975 1.007549
H -2.234023 -1.516879 2.558476
C -2.397053 -2.047463 -1.339132
H -1.144644 -0.356369 -1.526185
C -3.115500 -2.974905 -0.444389
H -3.522163 -3.420834 1.663565
H -2.466571 -2.244569 -2.405134
H -0.477446 -0.866558 0.848735
O -3.779158 -3.909690 -0.884617
C 3.285649 -1.866073 -0.615495
C 1.820607 -1.508138 -0.206669
O 1.360657 -0.467164 -0.713292
O 1.314135 -2.324900 0.580373
F 4.071320 -2.067139 0.463546
F 3.888942 -0.919275 -1.355857
F 3.327251 -3.006180 -1.338185
C 2.491280 3.270135 -0.155857
H 2.602851 4.162482 -0.774373
H 2.846643 3.497333 0.851379
H 3.124455 2.481867 -0.576304
42
C_28 Eopt -1731.152037
C -1.386976 0.462439 0.770409
C -1.323095 -0.859274 1.261212
C -0.362828 -1.282121 2.159134
C 0.634176 -0.374304 2.623258
C 0.643040 0.903101 2.166487
C -0.343128 1.440637 1.193564
C -2.500440 0.660233 -0.075566
C -3.249900 -0.518106 -0.235837
H -0.366478 -2.306816 2.516996
H 1.382013 -0.725684 3.324408
H 1.417924 1.592485 2.494843
H -0.865549 2.282548 1.677682
S -2.610525 -1.833770 0.633839

C -2.864301 2.005407 -0.624933
O -2.479825 2.999254 -0.027919
C -3.656973 2.123982 -1.895242
H -4.715824 1.927746 -1.699460
H -3.555706 3.143407 -2.269693
H -3.309744 1.408325 -2.645156
C 0.446032 2.087931 -0.015766
C 1.103208 3.361468 0.415380
C 1.374820 1.093430 -0.643214
C 2.404860 3.610021 0.223938
H 0.460332 4.105898 0.882344
C 2.679404 1.338176 -0.829078
H 0.934455 0.146622 -0.961950
C 3.293301 2.613261 -0.410658
H 2.859660 4.550409 0.522184
H 3.336575 0.608384 -1.293155
H -0.312555 2.356303 -0.761263
O 4.487371 2.840437 -0.579507
C 2.131835 -2.563118 -0.667569
C 0.574134 -2.610063 -0.560506
O -0.038969 -1.840453 -1.319795
O 0.168452 -3.391347 0.320635
F 2.580602 -1.856158 -1.718413
F 2.676038 -3.791258 -0.763789
F 2.665835 -1.991232 0.437682
C -4.501569 -0.740089 -1.021980
H -4.872322 -1.757397 -0.885607
H -5.280422 -0.042475 -0.700448
H -4.316060 -0.582791 -2.087984
42
C_29 Eopt -1731.149723
C 0.426233 0.512678 0.155402
C 0.336869 1.169017 1.411831
C 0.145889 2.523070 1.527106
C 0.068011 3.340683 0.357366
C 0.274008 2.785318 -0.860834
C 0.637128 1.355372 -1.054337
C 0.473853 -0.881871 0.292900
C 0.476372 -1.281294 1.641545
H 0.026568 2.980035 2.506369
H -0.148975 4.397053 0.464871
H 0.256066 3.401090 -1.756399
H 0.099042 0.962331 -1.922224
S 0.372220 0.027269 2.725012
C 0.355832 -1.911125 -0.791722
O 0.906878 -2.989855 -0.668694
C -0.543754 -1.618194 -1.967616
H 0.065084 -1.384362 -2.847402
H -1.107864 -2.527145 -2.191366
H -1.228938 -0.790556 -1.773858
C 2.196305 1.323334 -1.453673
C 3.059800 1.643178 -0.271964
C 2.535053 0.027924 -2.118045
C 3.977541 0.795489 0.211659
H 2.904115 2.607861 0.207216
C 3.454120 -0.822437 -1.643349
H 1.993903 -0.202598 -3.033066
C 4.212991 -0.522767 -0.411192
H 4.583494 1.041845 1.078937
H 3.680075 -1.760376 -2.142586
H 2.303282 2.121852 -2.200700
O 5.013512 -1.322993 0.060625
C -4.172790 -0.198662 -0.457966
C -2.805562 0.272895 0.131887
O -2.206794 1.075104 -0.614233
O -2.487739 -0.224894 1.221269
F -4.934400 0.835963 -0.867095
F -4.919204 -0.911289 0.400447
F -3.977624 -0.983820 -1.543812
C 0.512195 -2.671944 2.173419
H -0.246847 -3.278184 1.669878
H 1.484802 -3.123936 1.958360
H 0.334310 -2.691776 3.249675
35
C_3-notfa Eopt -1204.937983
C 0.883266 0.375649 0.481188
C 1.253569 1.698258 0.162184
C 0.473670 2.794415 0.485589
C -0.756104 2.625530 1.176389
C -1.187676 1.370358 1.470711
C -0.455125 0.139836 1.088579
C 1.878297 -0.563688 0.117342
C 2.985608 0.055116 -0.484426
H 0.805247 3.796760 0.223752
H -1.336712 3.499391 1.447026
H -2.142714 1.228718 1.971372
H -0.335250 -0.497730 1.976849
S 2.815251 1.753356 -0.597880
C 1.752885 -2.024637 0.441876
O 0.918379 -2.361217 1.266462
C 2.615596 -3.043444 -0.243867
H 2.632488 -2.881153 -1.324800
H 3.643638 -2.982049 0.126250
H 2.218320 -4.033605 -0.019025
C -1.389051 -0.708750 0.115512
C -2.582265 -1.226063 0.854999
C -1.716571 0.059376 -1.127974
C -3.837355 -1.052643 0.422521
H -2.381567 -1.781717 1.769281
C -2.970606 0.240137 -1.560183
H -0.878080 0.463387 -1.694060
C -4.127736 -0.299599 -0.815676
H -4.689837 -1.457970 0.959815
H -3.189131 0.787777 -2.472409
H -0.782079 -1.578712 -0.165459
O -5.276627 -0.128890 -1.207124
C 4.261472 -0.540730 -0.982247
H 4.767394 -1.075888 -0.173529
H 4.064360 -1.247304 -1.792616
H 4.934642 0.232539 -1.356184
42
C_3 Eopt -1731.148046
C -0.945923 -1.473992 0.275322
C -1.254241 -2.066875 -0.983323
C -0.507998 -3.078139 -1.533279
C 0.654016 -3.573923 -0.856793
C 1.055490 -2.990446 0.295344
C 0.371451 -1.808958 0.894335
C -1.966265 -0.629892 0.725397
C -2.999538 -0.502986 -0.232266
H -0.803261 -3.526257 -2.479179
H 1.205175 -4.398593 -1.293155
H 1.963529 -3.323233 0.792394
H 0.249519 -1.979877 1.971081
S -2.750789 -1.450597 -1.617900
C -2.130399 0.045935 2.061867
O -3.265222 0.202177 2.476725
C -0.953818 0.516030 2.871153
H -0.222106 -0.276588 3.045455
H -0.468049 1.341886 2.342725
H -1.325562 0.874744 3.831788
C 1.383482 -0.581037 0.758015
C 2.487285 -0.715456 1.759748
C 1.849101 -0.381873 -0.652535
C 3.782675 -0.636029 1.431452
H 2.187054 -0.865269 2.795799
C 3.145017 -0.302187 -0.982151
H 1.074086 -0.258962 -1.408302
C 4.210404 -0.433745 0.030594
H 4.569140 -0.714972 2.176587
H 3.462285 -0.130793 -2.006864
H 0.798952 0.306993 1.031413
O 5.397993 -0.379703 -0.271914
C 0.489859 2.952660 -0.684401
C -0.765149 2.020459 -0.662650
O -0.819501 1.167470 -1.564553
O -1.528520 2.257038 0.293252
F 1.273735 2.786273 -1.760937
F 1.271692 2.731144 0.400460
F 0.150051 4.256398 -0.648380
C -4.196923 0.376047 -0.165544
H -3.877774 1.360175 0.191187
H -4.669703 0.472613 -1.144726
H -4.919121 -0.026053 0.550129
42
C_30 Eopt -1731.145357
C -0.431343 -1.520412 0.190636
C -0.728466 -2.016655 -1.105092
C 0.162554 -2.754395 -1.855133
C 1.459008 -3.058861 -1.348638
C 1.831932 -2.555742 -0.145940
C 0.980308 -1.646786 0.664196
C -1.568368 -0.970943 0.821472
C -2.703352 -1.021832 -0.015913
H -0.130725 -3.132894 -2.831387
H 2.129774 -3.672589 -1.937959
H 2.833008 -2.738446 0.238065
H 1.000996 -1.985482 1.706584
S -2.388177 -1.733236 -1.532168
C -1.709437 -0.465815 2.233850
O -2.786741 -0.031672 2.596688
C -0.579076 -0.600305 3.218026
H 0.301202 -0.038049 2.902836
H -0.921993 -0.205998 4.175102
H -0.309231 -1.654735 3.341366
C 1.740663 -0.236227 0.674227
C 2.939962 -0.309601 1.563297
C 2.032280 0.257031 -0.708398
C 4.159773 0.088096 1.179076
H 2.775981 -0.682325 2.573484
C 3.250522 0.655286 -1.098138
H 1.193283 0.310634 -1.399937
C 4.409123 0.594461 -0.186269
H 5.011322 0.056295 1.852718
H 3.433031 1.038890 -2.097720
H 1.025269 0.477018 1.116275
O 5.530274 0.940322 -0.545522
C -1.349839 2.303726 -0.909981
C -0.353289 2.598075 0.257450
O -0.510994 1.835396 1.235216
O 0.467307 3.497665 0.042110
F -2.612110 2.108397 -0.482311
F -0.991998 1.163970 -1.558345
F -1.400965 3.267495 -1.840932

C -4.099124 -0.578306 0.260681
H -4.734605 -0.717744 -0.616072
H -4.505737 -1.150762 1.099929
H -4.106845 0.470748 0.561313
42
C_31 Eopt -
1731.152147
C -0.388922 -1.542299 0.158215
C -0.842303 -2.045690 -1.082179
C -0.020146 -2.725283 -1.959256
C 1.342446 -2.969060 -1.630742
C 1.840684 -2.493185 -0.459540
C 1.051131 -1.693363 0.506462
C -1.434443 -0.976014 0.921009
C -2.668472 -1.060308 0.260612
H -0.414929 -3.095491 -2.902247
H 1.964297 -3.521927 -2.324737
H 2.888374 -2.649606 -0.211311
H 1.103933 -2.177713 1.493548
S -2.551158 -1.799535 -1.278421
C -1.214371 -0.446676 2.309787
O -0.209935 -0.800208 2.906332
C -2.216471 0.468416 2.947748
H -3.085440 -0.105640 3.286108
H -1.743954 0.936961 3.811925
H -2.540276 1.234721 2.241735
C 1.794278 -0.303996 0.709787
C 3.027845 -0.487800 1.531322
C 2.024492 0.378312 -0.601167
C 4.229365 -0.030617 1.154060
H 2.902422 -0.993438 2.487376
C 3.224297 0.832716 -0.987137
H 1.154100 0.514121 -1.240977
C 4.419502 0.657890 -0.139459
H 5.109374 -0.144190 1.780704
H 3.364635 1.347470 -1.933225
H 1.097744 0.326473 1.279880
O 5.524357 1.058662 -0.493962
C -1.480496 2.189130 -0.988770
C -0.388712 2.625306 0.042189
O -0.359367 1.890285 1.051045
O 0.298736 3.597668 -0.291739
F -2.662082 1.907395 -0.399825
F -1.102699 1.054983 -1.632739
F -1.732875 3.099090 -1.940666
C -4.031509 -0.662811 0.726527
H -4.784674 -0.892992 -0.029047
H -4.284586 -1.205491 1.642622
H -4.073080 0.406818 0.941240
42
C_32 Eopt -
1731.148039
C 0.300505 0.597328 0.034661
C 0.081518 1.410051 1.184754
C -0.057367 2.772385 1.118488
C 0.068280 3.449230 -0.138739
C 0.406390 2.746606 -1.244508
C 0.693779 1.284966 -1.230717
C 0.206393 -0.764554 0.327801
C -0.029862 -0.997578 1.701289
H -0.287473 3.346405 2.012832
H -0.098949 4.519126 -0.180026
H 0.545762 3.252275 -2.196377
H 0.230939 0.819493 -2.108152
S -0.162979 0.435538 2.607459
C 0.210005 -1.936010 -0.618878
O 0.728306 -2.978120 -0.266171
C -0.495543 -1.833045 -1.946832
H -1.249196 -2.625505 -1.969994
H -1.002827 -0.881127 -2.104161
H 0.219975 -2.018119 -2.753555
C 2.275662 1.119494 -1.461607
C 3.039165 1.548473 -0.246703
C 2.584710 -0.269106 -1.918448
C 3.868096 0.732660 0.417530
H 2.886428 2.569882 0.097138
C 3.416267 -1.088119 -1.262449
H 2.096497 -0.596399 -2.833824
C 4.096193 -0.659573 -0.021975
H 4.406583 1.060030 1.302287
H 3.623536 -2.095379 -1.612496
H 2.503929 1.806229 -2.288669
O 4.825794 -1.418839 0.605500
C -3.256207 0.371280 -0.274268
C -3.219484 -1.179618 -0.486197
O -3.819455 -1.549772 -1.506585
O -2.562311 -1.807211 0.364710
F -4.467683 0.904062 -0.513801
F -2.394970 0.991051 -1.125876
F -2.901228 0.759094 0.963135
C -0.198080 -2.311193 2.374300
H -0.915292 -2.905630 1.800685
H 0.756261 -2.845989 2.380487
H -0.554785 -2.192288 3.398711
42
C_33 Eopt -
1731.149898
C 0.327833 0.719645 0.029839
C 0.196360 1.595714 1.137851
C 0.206700 2.964595 1.006215
C 0.395707 3.563147 -0.278126
C 0.652035 2.775147 -1.351124
C 0.763685 1.295314 -1.272148
C 0.111321 -0.622380 0.378373
C -0.124074 -0.775209 1.757963
H 0.052971 3.601854 1.873893
H 0.348368 4.641816 -0.369513
H 0.844577 3.218905 -2.324599
H 0.220439 0.834114 -2.103923
S -0.111829 0.709234 2.602871
C -0.006281 -1.714729 -0.652391
O -0.314536 -1.402278 -1.788053
C 0.280519 -3.138672 -0.278801
H 0.446146 -3.709312 -1.193859
H 1.152196 -3.212854 0.377876
H -0.589919 -3.550874 0.241369
C 2.318524 0.938279 -1.518811
C 3.137808 1.329168 -0.326611
C 2.478863 -0.490643 -1.926192
C 3.857584 0.449883 0.382275
H 3.113167 2.377027 -0.032556
C 3.208524 -1.371471 -1.227956
H 1.965309 -0.795624 -2.834197
C 3.906930 -0.980062 0.012467
H 4.431992 0.749948 1.254117
H 3.316092 -2.404472 -1.547669
H 2.603736 1.564823 -2.374915
O 4.511833 -1.798909 0.696870
C -3.191936 0.345488 -0.318450
C -3.216273 -1.202732 -0.557739
O -3.783529 -1.521540 -1.611937
O -2.660851 -1.881372 0.327265
F -4.407476 0.904876 -0.482991
F -2.366006 0.969691 -1.192410
F -2.774187 0.700534 0.911747
C -0.421294 -2.015638 2.526312
H -1.320733 -2.473922 2.102804
H 0.407733 -2.723958 2.445492
H -0.592348 -1.797385 3.581779
42
C_34 Eopt -
1731.149732
C -0.246908 -0.234868 -0.062314
C 0.492359 -0.434328 1.129882
C 1.162955 -1.606961 1.402271
C 1.137347 -2.682899 0.468124
C 0.375318 -2.575640 -0.650260
C -0.497407 -1.408906 -0.940713
C -0.736194 1.078165 -0.172096
C -0.377973 1.865346 0.934268
H 1.741019 -1.707996 2.317753
H 1.728178 -3.569597 0.665708
H 0.324499 -3.399052 -1.358275
H -0.394558 -1.123658 -1.992824
S 0.533847 1.010254 2.095854
C -1.433567 1.590261 -1.400562
O -1.176676 1.082569 -2.476985
C -2.444172 2.697574 -1.282563
H -3.045846 2.598423 -0.374857
H -1.930730 3.664474 -1.251895
H -3.084542 2.675563 -2.165786
C -2.013081 -1.920050 -0.751042
C -2.326254 -2.122652 0.700268
C -2.977180 -1.024655 -1.460624
C -3.318323 -1.479686 1.330174
H -1.693443 -2.817314 1.249463
C -3.980695 -0.390830 -0.838245
H -2.830865 -0.909111 -2.531522
C -4.193894 -0.528227 0.615920
H -3.518582 -1.631758 2.387028
H -4.676614 0.245842 -1.377822
H -2.035187 -2.893829 -1.259471
O -5.064770 0.107746 1.201542
C 3.965675 -0.059741 -0.167940
C 2.708185 0.490205 -0.921142
O 2.436100 1.662076 -0.608362
O 2.160239 -0.310011 -1.698787
F 3.784542 -0.035362 1.175029
F 5.063646 0.684698 -0.415035
F 4.283329 -1.327889 -0.481204
C -0.636235 3.315749 1.172268
H -1.709056 3.504445 1.270925
H -0.138372 3.661455 2.079565
H -0.260001 3.898854 0.325935
42
C_35 Eopt -
1731.151150
C 0.260813 -0.322848 0.046391
C -0.404906 -0.673281 -1.159809
C -1.000199 -1.897653 -1.350814
C -0.933027 -2.896482 -0.331653
C -0.214248 -2.656612 0.791904
C 0.570796 -1.412382 1.013565
C 0.633100 1.026039 0.068520
C 0.301409 1.687838 -1.128547
H -1.550635 -2.104600 -2.265841
H -1.460989 -3.832073 -0.474056
H -0.130137 -3.418431 1.562782

H 0.426304 -1.082174 2.047628
S -0.488006 0.680306 -2.253032
C 1.145164 1.820913 1.235650
O 1.907406 2.750022 1.041009
C 0.609025 1.507022 2.607827
H -0.332925 0.955032 2.558475
H 1.352118 0.934067 3.172182
H 0.462195 2.455629 3.130391
C 2.124383 -1.812779 0.882414
C 2.488979 -2.046885 -0.551670
C 2.977974 -0.795901 1.568142
C 3.413805 -1.322195 -1.195140
H 1.947266 -2.832221 -1.076013
C 3.907432 -0.070859 0.933060
H 2.813228 -0.669224 2.635604
C 4.160535 -0.245304 -0.512625
H 3.651912 -1.495456 -2.240772
H 4.512640 0.667549 1.451222
H 2.213912 -2.757549 1.435326
O 4.966810 0.457178 -1.112387
C -3.787953 -0.034892 0.091398
C -2.576758 0.481718 0.934938
O -2.284822 1.667367 0.706906
O -2.079698 -0.351672 1.716735
F -3.648708 0.247614 -1.221912
F -4.939490 0.549182 0.488298
F -3.981129 -1.363021 0.175419
C 0.521793 3.121920 -1.459713
H 1.593287 3.314959 -1.566069
H 0.009707 3.401591 -2.381505
H 0.157877 3.746609 -0.638225

42

C_36 Eopt -1731.147113

C 0.158952 0.697065 1.093740
C -1.209435 0.721170 1.455415
C -1.763584 -0.174269 2.343097
C -0.958788 -1.186871 2.939502
C 0.347264 -1.293188 2.593599
C 1.018211 -0.412422 1.600623
C 0.504285 1.792028 0.279445
C -0.607179 2.589059 -0.036171
H -2.816328 -0.104864 2.606381
H -1.410144 -1.874082 3.645128
H 0.952221 -2.088841 3.021418
H 1.882118 0.048525 2.106056
S -2.041044 2.058386 0.719009
C 1.872856 2.190906 -0.195981
O 1.993989 2.736729 -1.276579
C 3.051379 1.994313 0.723403
H 3.432500 0.969908 0.648385
H 3.847566 2.671771 0.410386
H 2.779011 2.186404 1.765159
C 1.641545 -1.380814 0.479572
C 1.712990 -0.788643 -0.893001
C 2.942472 -1.926618 0.980224
C 2.834017 -0.799000 -1.629089
H 0.775570 -0.415086 -1.305030
C 4.068743 -1.914803 0.254839
H 2.953657 -2.352075 1.981819
C 4.101555 -1.341051 -1.106001
H 2.849969 -0.406994 -2.642430
H 5.000057 -2.321863 0.638160
H 0.913198 -2.199122 0.387810
O 5.136410 -1.320309 -1.766307
C -3.389824 -1.262411 -1.075334
C -1.840343 -1.080583 -1.008576
O -1.281860 -1.899308 -0.253829
O -1.385969 -0.127682 -1.662761
F -3.954882 -0.878481 0.097271
F -3.752378 -2.544496 -1.269109
F -3.983391 -0.540428 -2.037783
C -0.654549 3.786690 -0.920714
H -1.580266 4.347470 -0.780406
H 0.202290 4.437556 -0.732375
H -0.592824 3.458988 -1.963763

42

C_37 Eopt -1731.149182

C 0.104862 0.591069 1.123406
C -1.261772 0.722507 1.450713
C -1.919427 -0.177977 2.264649
C -1.230610 -1.302667 2.796057
C 0.070792 -1.506810 2.466434
C 0.859979 -0.596850 1.601704
C 0.587334 1.696352 0.394287
C -0.422243 2.617828 0.104473
H -2.967109 -0.028501 2.514760
H -1.764431 -1.998145 3.432656
H 0.593220 -2.387612 2.834103
H 1.664227 -0.184900 2.237515
S -1.933560 2.177456 0.775363
C 2.046917 1.870762 0.085742
O 2.858309 1.433853 0.884214
C 2.472357 2.564675 -1.176654
H 2.460630 3.648890 -1.019579
H 3.493137 2.260446 -1.414564
H 1.799777 2.330848 -2.006286
C 1.611226 -1.473032 0.497069
C 1.660070 -0.884513 -0.878308
C 2.943486 -1.902978 1.027291
C 2.788542 -0.831935 -1.600488
H 0.708284 -0.563191 -1.299591
C 4.077726 -1.811160 0.321695
H 2.960659 -2.309850 2.037499
C 4.086570 -1.256608 -1.046021
H 2.793039 -0.453497 -2.619355
H 5.033004 -2.130741 0.728043
H 0.979238 -2.365387 0.377246
O 5.127612 -1.155723 -1.690818
C -3.405111 -1.123430 -1.167053
C -1.852723 -0.970659 -1.100871
O -1.303215 -1.826059 -0.381548
O -1.385958 0.001127 -1.718693
F -3.959386 -0.735000 0.009495
F -3.793319 -2.396385 -1.367615
F -3.986306 -0.383462 -2.123559
C -0.326289 3.892632 -0.666073
H -1.254644 4.462871 -0.605786
H 0.489423 4.510855 -0.281481
H -0.124497 3.673425 -1.719422

42

C_38 Eopt -1731.149176

C 0.107626 0.595848 1.119523
C -1.258529 0.742303 1.442175
C -1.928063 -0.148269 2.257441
C -1.252672 -1.278564 2.793918
C 0.047000 -1.498425 2.467233
C 0.849159 -0.598311 1.603963
C 0.603372 1.693847 0.387922
C -0.395917 2.624672 0.092114
H -2.974831 0.013851 2.503790
H -1.795433 -1.966544 3.431115
H 0.558695 -2.384045 2.838551
H 1.651606 -0.191298 2.245531
S -1.913446 2.201707 0.760244
C 2.065500 1.853557 0.083076
O 2.870481 1.413586 0.886332
C 2.500688 2.538140 -1.181190
H 1.828842 2.306223 -2.011914
H 2.497907 3.623010 -1.028444
H 3.519607 2.224254 -1.414666
C 1.602068 -1.480155 0.505454
C 1.649338 -0.900022 -0.873562
C 2.934815 -1.906743 1.036840
C 2.776872 -0.852542 -1.597535
H 0.697435 -0.579591 -1.295224
C 4.068088 -1.820555 0.328946
H 2.953432 -2.307252 2.049508
C 4.075543 -1.273941 -1.041910
H 2.780247 -0.480123 -2.618633
H 5.023764 -2.137988 0.736085
H 0.970893 -2.373708 0.391516
O 5.116053 -1.175664 -1.688061
C -3.413899 -1.120072 -1.161371
C -1.861385 -0.970659 -1.093265
O -1.391549 -0.001147 -1.712310
O -1.315034 -1.826118 -0.371482
F -3.805212 -2.392979 -1.355922
F -3.991571 -0.383603 -2.122677
F -3.968780 -0.724010 0.012323
C -0.285022 3.897406 -0.680054
H -0.077876 3.676285 -1.731823
H -1.209210 4.475045 -0.626439
H 0.533033 4.509528 -0.290506

42

C_39 Eopt -1731.154407

C -0.408175 -1.533644 0.348819
C -0.794215 -2.266364 -0.800075
C 0.070223 -3.088044 -1.488010
C 1.416654 -3.253229 -1.046474
C 1.852124 -2.566855 0.038783
C 1.012055 -1.607946 0.801012
C -1.496177 -0.852418 0.931171
C -2.699953 -1.086598 0.238939
H -0.272477 -3.633082 -2.364167
H 2.073312 -3.921605 -1.590771
H 2.883520 -2.667724 0.369293
H 1.005331 -1.912549 1.859385
S -2.491161 -2.084413 -1.124225
C -1.365749 -0.036843 2.180927
O -0.422110 -0.255169 2.925298
C -2.363143 1.045125 2.483009
H -2.670506 1.570066 1.574336
H -3.252699 0.605252 2.946912
H -1.909803 1.741079 3.190488
C 1.748856 -0.203827 0.793293
C 2.977494 -0.254059 1.644806
C 1.983475 0.269198 -0.607932
C 4.182523 0.131488 1.206204
H 2.845805 -0.601036 2.668457
C 3.190474 0.649581 -1.049615
H 1.109326 0.314142 -1.260206
C 4.381130 0.606170 -0.179750
H 5.059215 0.114148 1.847560
H 3.339111 1.006230 -2.065062

H 1.053448 0.492994 1.274968
O 5.489406 0.944559 -0.584747
C -0.744321 2.660210 -0.665546
C -1.590479 1.521820 -1.322317
O -0.921374 0.701895 -1.975780
O -2.802134 1.563229 -1.049905
F -1.447787 3.774292 -0.409228
F 0.317464 3.028140 -1.401483
F -0.252152 2.237332 0.531019
C -4.086882 -0.654383 0.581777
H -4.321002 -0.926546 1.614812
H -4.171998 0.428063 0.463548
H -4.815539 -1.129119 -0.077762
35
C_4-notfa Eopt -1204.934634
C -0.906111 -0.407067 0.473756
C -1.397607 -1.697732 0.158647
C -0.694415 -2.850882 0.437391
C 0.589609 -2.784713 1.052502
C 1.135258 -1.576200 1.338458
C 0.456038 -0.281501 1.070563
C -1.807742 0.602791 0.097479
C -2.959301 0.089067 -0.514784
H -1.113146 -3.822636 0.187491
H 1.120150 -3.705088 1.265957
H 2.127078 -1.518045 1.780379
H 0.358051 0.215949 2.050980
S -2.964535 -1.617646 -0.595430
C -1.654682 2.095563 0.254380
O -1.865557 2.807810 -0.707416
C -1.365480 2.655643 1.617895
H -2.322340 2.694581 2.154301
H -0.693755 2.029904 2.208593
H -0.969579 3.667911 1.527055
C 1.395727 0.657773 0.217610
C 2.621737 1.027814 0.993897
C 1.673947 0.050112 -1.124946
C 3.861782 0.857091 0.520301
H 2.461227 1.468921 1.976154
C 2.913459 -0.134057 -1.595184
H 0.811588 -0.238324 -1.724713
C 4.102895 0.254937 -0.808339
H 4.738724 1.152245 1.089128
H 3.095833 -0.571649 -2.572431
H 0.826322 1.586203 0.059984
O 5.237963 0.085668 -1.237313
C -4.103814 0.866155 -1.068036
H -4.347061 1.702808 -0.407826
H -3.820075 1.283114 -2.039979
H -4.987510 0.239864 -1.199905
42
C_4 Eopt -1731.154695
C -0.919790 -1.535225 0.264137
C -1.253465 -2.141695 -0.972322
C -0.492741 -3.139261 -1.539173
C 0.697221 -3.596430 -0.895513
C 1.112281 -2.994829 0.247006
C 0.401027 -1.851396 0.871632
C -1.919383 -0.657129 0.722460
C -2.964179 -0.530205 -0.214072
H -0.798356 -3.595983 -2.477737
H 1.264417 -4.401855 -1.346973
H 2.045632 -3.297761 0.715998
H 0.276378 -2.027314 1.948445
S -2.762103 -1.530018 -1.579360
C -1.846657 0.004965 2.064857
O -0.830639 -0.135692 2.730113
C -3.022727 0.753484 2.621465
H -3.239126 1.624095 1.998993
H -3.908218 0.111308 2.654807
H -2.769367 1.077975 3.631240
C 1.367690 -0.581895 0.787011
C 2.486028 -0.721280 1.769484
C 1.808727 -0.319130 -0.619597
C 3.774956 -0.594180 1.428444
H 2.201626 -0.910824 2.802989
C 3.097733 -0.196394 -0.963883
H 1.021645 -0.196012 -1.363245
C 4.179010 -0.333761 0.030905
H 4.572890 -0.674024 2.161245
H 3.397202 0.016138 -1.986322
H 0.744911 0.259029 1.121891
O 5.361227 -0.239210 -0.285107
C 0.466946 2.959379 -0.684256
C -0.798359 2.042884 -0.635247
O -0.898301 1.207234 -1.549325
O -1.530865 2.276298 0.345847
F 1.216612 2.791392 -1.785419
F 1.276886 2.729127 0.374493
F 0.137747 4.266673 -0.635946
C -4.143553 0.379237 -0.201980
H -3.771928 1.406236 -0.114392
H -4.722346 0.284531 -1.122354
H -4.798881 0.168157 0.646643
42
C_40 Eopt -1731.148654
C 0.316501 -1.592431 -0.087809
C 0.763878 -1.971114 1.204197
C -0.065086 -2.545933 2.137742
C -1.443179 -2.780298 1.837141
C -1.942741 -2.400385 0.636901
C -1.133815 -1.724606 -0.413337
C 1.363323 -1.105772 -0.884412
C 2.593523 -1.088833 -0.192222
H 0.323320 -2.835482 3.111173
H -2.073058 -3.246699 2.585493
H -2.997020 -2.545309 0.412509
H -1.236739 -2.313425 -1.337899
S 2.467322 -1.686247 1.392800
C 1.306665 -0.711328 -2.332025
O 2.067701 0.140538 -2.749679
C 0.365314 -1.441175 -3.257814
H 0.313523 -2.506133 -3.013764
H -0.644902 -1.021965 -3.194532
H 0.720203 -1.308137 -4.280808
C -1.801621 -0.321525 -0.729917
C -3.077157 -0.510141 -1.490523
C -1.936363 0.499326 0.517421
C -4.236339 0.041987 -1.112399
H -3.022693 -1.111371 -2.396724
C -3.098674 1.047359 0.897603
H -1.027715 0.650669 1.104108
C -4.335582 0.860194 0.116060
H -5.148127 -0.086200 -1.688846
H -3.174661 1.651435 1.797396
H -1.094589 0.203471 -1.388810
O -5.402926 1.353695 0.465886
C 1.011529 2.697919 0.223567
C 1.728448 1.707897 1.198050
O 0.932333 1.033379 1.881113
O 2.966826 1.700693 1.136141
F 1.830536 3.597878 -0.341575
F 0.029629 3.393599 0.831675
F 0.422480 2.021174 -0.793548
C 3.919787 -0.648134 -0.704607
H 4.090906 -1.048693 -1.707079
H 3.924380 0.443942 -0.767055
H 4.724289 -0.964475 -0.038325
42
C_41 Eopt -1731.146595
C 1.536280 0.899171 0.545514
C 1.611211 2.010875 -0.321287
C 0.540601 2.861728 -0.538145
C -0.708574 2.618662 0.086783
C -0.877899 1.487068 0.826524
C 0.186764 0.470912 0.997034
C 2.785523 0.262390 0.700806
C 3.778263 0.845265 -0.099966
H 0.657683 3.731647 -1.180100
H -1.523028 3.318044 -0.059791
H -1.839591 1.241278 1.267568
H 0.173046 0.089422 2.023288
S 3.210380 2.198718 -0.978330
C 3.155077 -0.804619 1.691254
O 4.036062 -1.599353 1.423076
C 2.492315 -0.787994 3.046073
H 1.576053 -1.386462 3.025919
H 3.173864 -1.243553 3.766579
H 2.233824 0.227120 3.357644
C -0.276892 -0.802656 0.085116
C -0.137411 -0.476532 -1.367226
C 0.460135 -2.026822 0.513821
C 0.698589 -1.136922 -2.180673
H -0.756656 0.335231 -1.741806
C 1.289570 -2.697792 -0.296169
H 0.283830 -2.371197 1.530417
C 1.525455 -2.256518 -1.687955
H 0.794892 -0.882684 -3.232493
H 1.815674 -3.590356 0.030353
H -1.341140 -0.911733 0.335136
O 2.353572 -2.811389 -2.403110
C -5.322736 -0.092918 0.019642
C -3.769921 -0.074421 0.165243
O -3.375526 -0.462310 1.283602
O -3.131026 0.326200 -0.821524
F -5.825509 -1.325737 0.237681
F -5.758841 0.294930 -1.188898
F -5.905547 0.725532 0.922924
C 5.206056 0.438985 -0.233162
H 5.785497 1.194585 -0.766482
H 5.644821 0.272405 0.754243
H 5.265143 -0.508102 -0.778280
42
C_42 Eopt -1731.149902
C -1.555491 -0.961543 0.536934
C -1.625850 -2.087272 -0.304919
C -0.548478 -2.939365 -0.502100
C 0.691419 -2.686907 0.131234
C 0.847738 -1.551316 0.871617
C -0.221705 -0.540999 1.037502
C -2.804131 -0.310589 0.665323
C -3.796833 -0.916085 -0.110214
H -0.657603 -3.817027 -1.134917

H 1.510667 -3.383079 -0.003286
H 1.804180 -1.304891 1.324362
H -0.260079 -0.203102 2.079419
S -3.223591 -2.291204 -0.958267
C -3.039932 0.799209 1.650093
O -2.397321 0.794995 2.685414
C -4.052841 1.868679 1.354314
H -5.045080 1.524549 1.666953
H -3.800175 2.756961 1.935808
H -4.090554 2.108757 0.288815
C 0.273203 0.761921 0.201720
C 0.171333 0.504371 -1.267657
C -0.445533 1.984445 0.664488
C -0.592183 1.244955 -2.083509
H 0.760610 -0.324120 -1.653804
C -1.189646 2.742943 -0.152734
H -0.328164 2.250447 1.712576
C -1.374188 2.388542 -1.575138
H -0.658806 1.039849 -3.148242
H -1.686366 3.644201 0.196112
H 1.333416 0.850154 0.481907
O -2.134909 3.024459 -2.299472
C 5.296645 0.101767 -0.009302
C 3.752989 0.013243 0.196113
O 3.382618 0.380143 1.329180
O 3.098184 -0.421824 -0.765650
F 5.774960 1.323723 0.299165
F 5.691384 -0.162955 -1.264587
F 5.939201 -0.776879 0.792661
C -5.239417 -0.550427 -0.242498
H -5.782458 -1.294324 -0.827808
H -5.703372 -0.479003 0.745115
H -5.339828 0.419270 -0.739272
35
C_5-notfa Eopt -
1204.932004
C 1.095083 0.435970 0.265316
C 2.403917 0.719925 -0.176153
C 2.888225 2.009916 -0.286663
C 2.068008 3.119012 0.060401
C 0.785933 2.904257 0.456808
C 0.160962 1.561825 0.541041
C 0.853065 -0.949682 0.369016
C 1.964715 -1.709057 -0.012694
H 3.906024 2.184289 -0.627798
H 2.475399 4.120594 -0.007017
H 0.142258 3.746440 0.700748
H -0.190769 1.428693 1.576631
S 3.300038 -0.741021 -0.477242
C -0.441317 -1.490315 0.912349
O -1.054555 -0.804171 1.710740
C -0.954079 -2.829107 0.464984
H -0.721852 -3.022664 -0.584827
H -0.493387 -3.617038 1.071454
H -2.033157 -2.858227 0.627228
C -1.174082 1.577431 -0.356366
C -1.272820 0.447971 -1.335807
C -2.380902 1.739915 0.516950
C -2.358610 -0.328411 -1.438711
H -0.434144 0.307586 -2.014625
C -3.464483 0.960609 0.421519
H -2.345505 2.547854 1.245726
C -3.522254 -0.154834 -0.541981
H -2.429633 -1.115744 -2.184255
H -4.329614 1.101091 1.062489
H -1.100502 2.486535 -0.972018
O -4.491245 -0.903600 -0.606499
C 2.123505 -3.193162 -0.026746
H 1.816952 -3.613930 0.934630
H 1.495475 -3.631411 -0.808573
H 3.159058 -3.479161 -0.217415
42
C_5 Eopt -
1731.152377
C -0.084219 0.400798 0.270265
C 0.510224 -0.238523 1.388113
C 0.292447 -1.564975 1.684172
C -0.583683 -2.350396 0.875931
C -1.238881 -1.773249 -0.161341
C -1.110661 -0.336123 -0.519696
C 0.386385 1.709481 0.100993
C 1.310497 2.074912 1.093904
H 0.802053 -2.030753 2.524480
H -0.714759 -3.400525 1.108951
H -1.930256 -2.359984 -0.761676
H -0.818004 -0.296739 -1.579223
S 1.614058 0.825787 2.213617
C 0.117005 2.634904 -1.050508
O 0.062653 3.835015 -0.851122
C -0.004838 2.046038 -2.429540
H 0.619876 1.152807 -2.529198
H -1.050888 1.774852 -2.621136
H 0.282182 2.807452 -3.156974
C -2.528083 0.359514 -0.420296
C -3.446438 -0.176110 -1.474118
C -3.078460 0.282689 0.971208
C -4.667367 -0.654287 -1.203804
H -3.077452 -0.150814 -2.498115
C -4.295866 -0.201690 1.246932
H -2.440812 0.651742 1.773551
C -5.183804 -0.707314 0.179658
H -5.325088 -1.022703 -1.985818
H -4.680090 -0.242165 2.262034
H -2.349526 1.421474 -0.650205
O -6.298344 -1.152743 0.430749
C 3.312702 -1.711313 -0.453049
C 2.486074 -0.593181 -1.168577
O 1.466009 -0.990022 -1.763930
O 2.976929 0.541591 -1.050920
F 3.673292 -1.360054 0.799002
F 4.457901 -1.966734 -1.123586
F 2.659922 -2.882208 -0.346132
C 2.043270 3.364894 1.219225
H 1.350272 4.158484 1.513191
H 2.845953 3.296619 1.954944
H 2.465783 3.641481 0.248733
35
C_6-notfa Eopt -
1204.930524
C 1.031377 0.481049 0.279082
C 2.245391 1.037856 -0.188089
C 2.481325 2.396661 -0.209271
C 1.482647 3.308153 0.239900
C 0.273211 2.835974 0.634039
C -0.103064 1.398185 0.582045
C 1.081361 -0.923900 0.337134
C 2.309218 -1.429159 -0.123416
H 3.439727 2.781709 -0.548609
H 1.701611 4.369167 0.248463
H -0.501994 3.531096 0.945879
H -0.543707 1.118698 1.547841
S 3.403094 -0.200326 -0.583406
C 0.017496 -1.865147 0.836660
O -0.064659 -2.972674 0.341254
C -0.869870 -1.439782 1.975089
H -0.318585 -0.851156 2.713770
H -1.702552 -0.834145 1.598193
H -1.286786 -2.334700 2.439307
C -1.309779 1.307025 -0.492433
C -1.373770 0.009887 -1.241139
C -2.583628 1.667171 0.211362
C -2.467984 -0.760396 -1.274756
H -0.496541 -0.270244 -1.819859
C -3.672248 0.888151 0.197960
H -2.598692 2.607095 0.758640
C -3.691654 -0.397636 -0.529406
H -2.500890 -1.678999 -1.853619
H -4.580683 1.168696 0.723106
H -1.093428 2.081225 -1.242309
O -4.677057 -1.125311 -0.521744
C 2.732649 -2.853550 -0.228141
H 2.455650 -3.395434 0.679894
H 2.206674 -3.329084 -1.061636
H 3.808355 -2.938082 -0.390379
42
C_6 Eopt -
1731.153912
C -0.117060 0.398521 0.373382
C 0.299722 -0.286387 1.539073
C -0.058254 -1.592308 1.790951
C -0.892948 -2.301803 0.875011
C -1.388702 -1.663425 -0.214948
C -1.140547 -0.227410 -0.502227
C 0.575355 1.608098 0.185961
C 1.476090 1.862755 1.230732
H 0.320290 -2.105456 2.671886
H -1.125131 -3.341715 1.072443
H -2.059743 -2.184662 -0.893755
H -0.822590 -0.101630 -1.547609
S 1.456413 0.654293 2.441128
C 0.492971 2.355495 -1.111428
O -0.537871 2.295681 -1.762688
C 1.695146 3.097786 -1.616303
H 1.775534 4.067479 -1.113698
H 2.602402 2.517850 -1.426295
H 1.564732 3.268243 -2.685974
C -2.535206 0.534152 -0.393703
C -3.423790 0.142706 -1.530738
C -3.160389 0.377714 0.956343
C -4.686778 -0.270695 -1.362111
H -2.998606 0.230393 -2.529046
C -4.421610 -0.038148 1.131213
H -2.548063 0.638262 1.818654
C -5.280330 -0.397195 -0.015785
H -5.323313 -0.527449 -2.204040
H -4.863902 -0.128216 2.119212
H -2.266322 1.588937 -0.537822
O -6.433895 -0.783751 0.144012
C 3.054141 -1.875697 -0.638177
C 2.667549 -0.411996 -1.024476
O 1.695136 -0.296840 -1.791854
O 3.384783 0.444393 -0.475671
F 2.877161 -2.090928 0.686928
F 4.351332 -2.137195 -0.896473
F 2.336066 -2.815374 -1.274750
C 2.424052 3.000689 1.390853
H 1.945059 3.946303 1.128799
H 2.798074 3.063828 2.414325

H 3.276232 2.837633 0.721408
42
C_7 Eopt -1731.153092
C 0.045493 0.411609 -0.228187
C -0.180393 0.691151 -1.597297
C 0.377033 -0.061412 -2.607842
C 1.236872 -1.157439 -2.299417
C 1.558826 -1.414815 -1.006440
C 1.092213 -0.582393 0.131942
C -0.792245 1.164858 0.615715
C -1.602354 2.059720 -0.114149
H 0.144574 0.156708 -3.647438
H 1.629811 -1.762328 -3.108034
H 2.247003 -2.221730 -0.766395
H 0.727924 -1.223652 0.947480
S -1.347325 1.963699 -1.793755
C -0.887635 0.893806 2.087549
O 0.054015 0.355514 2.648831
C -2.158308 1.205213 2.822351
H -2.222197 2.279194 3.024962
H -3.024920 0.904943 2.225349
H -2.141815 0.668364 3.771806
C 2.371905 0.149864 0.745346
C 3.280478 -0.852277 1.383408
C 3.044765 1.045108 -0.246674
C 4.592918 -0.917225 1.124659
H 2.823485 -1.530748 2.101884
C 4.356380 0.983107 -0.510742
H 2.424164 1.787295 -0.746515
C 5.230096 -0.007017 0.151611
H 5.239672 -1.637381 1.617506
H 4.830512 1.656786 -1.218755
H 1.952372 0.769510 1.548696
O 6.429948 -0.070122 -0.095754
C -2.477248 -1.894876 0.178447
C -3.198757 -0.726655 -0.572949
O -3.058810 -0.758969 -1.805296
O -3.832031 0.045887 0.170464
F -1.359040 -2.315082 -0.448090
F -3.287969 -2.970763 0.278649
F -2.108512 -1.574824 1.435690
C -2.618129 3.028293 0.386834
H -2.232612 3.588002 1.241557
H -2.909098 3.732123 -0.395039
H -3.508424 2.469544 0.695829
42
C_8 Eopt -1731.152793
C -0.181460 0.536057 0.018990
C -0.049104 1.786974 0.668186
C -0.677150 2.073454 1.858720
C -1.519878 1.105499 2.481499
C -1.749126 -0.079856 1.864550
C -1.180831 -0.438343 0.538821
C 0.640151 0.442493 -1.117421
C 1.371139 1.623879 -1.340262
H -0.520163 3.033504 2.344192
H -1.974499 1.338774 3.437054
H -2.418483 -0.807954 2.316190
H -0.704034 -1.422709 0.640660
S 1.072208 2.821355 -0.166794
C 0.839850 -0.752082 -2.004377
O 1.116446 -0.580422 -3.178119
C 0.744155 -2.136449 -1.416544
H 1.017162 -2.157312 -0.358255
H -0.281906 -2.508967 -1.524267
H 1.399610 -2.794568 -1.990390
C -2.375828 -0.622159 -0.489451
C -3.210698 -1.804996 -0.109784
C -3.152517 0.646970 -0.664499
C -4.540722 -1.745110 0.030872
H -2.681621 -2.744785 0.039733
C -4.481742 0.712889 -0.518832
H -2.586562 1.537294 -0.935420
C -5.277623 -0.478140 -0.156646
H -5.129798 -2.620070 0.290165
H -5.028181 1.640687 -0.661615
H -1.899078 -0.852751 -1.453812
O -6.494343 -0.417744 -0.017285
C 3.530401 -1.194358 0.669650
C 2.426382 -0.447078 1.487263
O 1.428529 -1.133357 1.780863
O 2.687420 0.749892 1.683158
F 4.773998 -0.906551 1.092613
F 3.476125 -0.826348 -0.636956
F 3.413203 -2.532728 0.690428
C 2.346959 1.900563 -2.431513
H 1.812111 2.045712 -3.375038
H 2.939700 2.790632 -2.214771
H 3.011944 1.043289 -2.564751
42
C_9 Eopt -1731.153150
C 0.170204 0.932383 -0.178546
C 0.182660 2.070281 -1.017870
C 0.904221 2.125755 -2.188628
C 1.691436 1.012375 -2.605749
C 1.764350 -0.086436 -1.813972
C 1.072566 -0.203800 -0.506721
C -0.747297 1.056359 0.888429
C -1.431250 2.281889 0.844401
H 0.861195 3.011215 -2.818587
H 2.226380 1.066410 -3.546483
H 2.386985 -0.928999 -2.106450
H 0.455255 -1.115069 -0.519328
S -0.937567 3.275987 -0.454616
C -1.094012 -0.103320 1.772763
O -0.607873 -1.194787 1.513814
C -2.030050 0.052830 2.934870
H -1.700311 0.854475 3.601379
H -3.034190 0.288581 2.571607
H -2.056378 -0.892884 3.476964
C 2.155855 -0.439955 0.624090
C 2.764387 -1.799504 0.487563
C 3.151549 0.678153 0.664362
C 4.086980 -2.006233 0.444205
H 2.070238 -2.636966 0.442860
C 4.474725 0.477049 0.616457
H 2.753180 1.688964 0.746562
C 5.043183 -0.881934 0.502186
H 4.509126 -3.003992 0.365817
H 5.182120 1.300392 0.657682
H 1.590079 -0.431107 1.564820
O 6.255093 -1.067072 0.455648
C -2.877932 -2.148489 -0.597898
C -2.645920 -0.619336 -0.814924
O -1.799075 -0.349148 -1.685396
O -3.352863 0.117357 -0.103516
F -3.816322 -2.603245 -1.464429
F -3.318800 -2.452880 0.634609
F -1.774663 -2.888051 -0.811547
C -2.550198 2.787115 1.690374
H -2.295064 2.754189 2.751167
H -2.809236 3.813569 1.424870
H -3.425254 2.151129 1.516523
34
D_1-notfa Eopt -1204.554484
C 0.972024 0.539912 0.025169
C 1.864501 1.634508 -0.065444
C 1.436407 2.963659 -0.082620
C 0.074435 3.207587 -0.007464
C -0.829421 2.142745 0.111341
C -0.410525 0.816740 0.149116
C 1.700912 -0.722334 0.010950
C 3.052615 -0.567833 -0.066629
H 2.151007 3.777819 -0.154093
H -0.296007 4.227491 -0.027283
H -1.892432 2.357687 0.187750
S 3.524957 1.109971 -0.144993
C 1.085682 -2.093530 -0.043962
O 0.425270 -2.427212 -1.011575
C 1.287235 -2.995794 1.140917
H 2.325428 -2.980261 1.482609
H 0.677361 -2.603999 1.965471
H 0.970609 -4.012912 0.905270
C -1.471114 -0.272705 0.312648
C -2.478771 0.092465 1.363078
C -2.084698 -0.606017 -1.017306
C -3.800929 0.090799 1.149490
H -2.080096 0.355413 2.341000
C -3.405914 -0.586057 -1.241470
H -1.390598 -0.867356 -1.812489
C -4.364452 -0.245110 -0.173149
H -4.509414 0.341497 1.933954
H -3.824662 -0.823566 -2.215342
H -0.977990 -1.180995 0.683192
O -5.576253 -0.240019 -0.373455
C 4.116647 -1.621489 -0.135295
H 3.691256 -2.591671 -0.401334
H 4.864699 -1.367172 -0.891154
H 4.631596 -1.718923 0.826335
42
D_1 Eopt -1731.217623
C -3.217097 -0.353439 -0.207902
C -4.274643 -1.007113 -0.884359
C -4.205679 -2.339118 -1.298016
C -3.043292 -3.043257 -1.028946
C -1.993043 -2.431207 -0.331547
C -2.063074 -1.111599 0.103529
C -3.568743 1.030642 0.084656
C -4.822089 1.371682 -0.331962
H -5.038237 -2.805552 -1.815394
H -2.949129 -4.077317 -1.344397
H -1.099254 -3.009208 -0.109474
H 4.095666 -1.357255 0.438802
S -5.640818 0.048985 -1.119678
C -2.635656 2.068291 0.639729
O -1.608951 2.340279 0.041836
C -2.981475 2.700562 1.958273
H -4.050326 2.906492 2.048099
H -2.731598 1.971849 2.741088
H -2.393819 3.606866 2.112520
C -0.878047 -0.560343 0.900361
C -0.428609 -1.528500 1.949688
C 0.215242 -0.122104 -0.024120

C 0.841101 -1.942556 2.073507
H -1.191582 -1.885852 2.637667
C 1.483803 -0.548660 0.084006
H -0.068266 0.572907 -0.810068
C 1.869500 -1.480549 1.142781
H 1.153386 -2.631353 2.852080
H 2.251039 -0.219524 -0.610343
H -1.209430 0.329486 1.453170
O 3.040589 -1.889007 1.264385
C 6.230016 0.805730 -0.819053
C 5.071879 0.280126 0.057615
O 4.886316 -0.991812 -0.145434
O 4.464667 1.019459 0.795011
F 7.359996 0.125004 -0.582342
F 6.466621 2.095594 -0.587175
F 5.936217 0.664168 -2.120827
C -5.520777 2.695703 -0.242416
H -4.805681 3.512135 -0.120648
H -6.096646 2.891269 -1.150787
H -6.215401 2.711309 0.604312
34
D_2-notfa Eopt -
1204.554485
C 0.966623 0.510864 0.069124
C 1.813891 1.644877 0.072995
C 1.334512 2.948952 0.203776
C -0.035722 3.129123 0.322230
C -0.899689 2.028139 0.267588
C -0.429692 0.725499 0.126348
C 1.742296 -0.715165 -0.073384
C 3.082175 -0.496009 -0.215073
H 2.014775 3.795109 0.206787
H -0.443446 4.128973 0.431066
H -1.973946 2.193697 0.312848
S 3.487660 1.198885 -0.133982
C 1.249943 -2.121071 0.077007
O 1.443707 -2.952745 -0.791878
C 0.647362 -2.500880 1.407706
H 0.243398 -1.641358 1.946157
H -0.112969 -3.274927 1.279367
H 1.464633 -2.915796 2.011340
C -1.467977 -0.385002 -0.052791
C -2.363037 -0.494069 1.147188
C -2.214764 -0.179369 -1.340409
C -3.698787 -0.418369 1.078157
H -1.871580 -0.633347 2.108223
C -3.550208 -0.105377 -1.414753
H -1.605573 -0.090447 -2.237677
C -4.393868 -0.219548 -0.209363
H -4.323743 -0.497684 1.963149
H -4.064624 0.042217 -2.360090
H -0.948217 -1.345786 -0.159669
O -5.618181 -0.152190 -0.272835
C 4.184853 -1.494095 -0.386994
H 4.472092 -1.564324 -1.441225
H 5.069627 -1.204270 0.185947
H 3.865351 -2.485736 -0.060926
42
D_2 Eopt -
1731.217284
C -3.222853 -0.453356 -0.154162
C -4.102217 -1.377184 -0.767917
C -3.819766 -2.739924 -0.872180
C -2.614446 -3.196116 -0.359749
C -1.701077 -2.294903 0.201558
C -1.972479 -0.933229 0.298104
C -3.782987 0.892137 -0.178354
C -4.985661 0.966097 -0.821403
H -4.522352 -3.419398 -1.344774
H -2.364499 -4.250537 -0.417547
H -0.740016 -2.662966 0.554224
H 4.169688 -0.850751 1.000356
S -5.532178 -0.591645 -1.384065
C -3.273702 2.098011 0.547382
O -3.080662 3.150424 -0.035305
C -3.161397 2.002880 2.050222
H -3.078981 0.973549 2.404278
H -2.328157 2.609764 2.412731
H -4.091396 2.419673 2.456900
C -0.846989 -0.026946 0.804560
C -0.450217 -0.377525 2.204407
C 0.293218 -0.041756 -0.166845
C 0.810901 -0.666741 2.558697
H -1.244722 -0.396603 2.947082
C 1.557286 -0.325274 0.182638
H 0.049676 0.196004 -1.199905
C 1.887845 -0.655599 1.569047
H 1.081911 -0.919914 3.578788
H 2.360974 -0.324105 -0.547269
H -1.200829 1.012772 0.822636
O 3.050526 -0.930790 1.919536
C 6.441240 0.399213 -1.019385
C 5.181488 0.367973 -0.126013
O 5.009057 -0.819692 0.377731
O 4.504847 1.356040 0.032114
F 7.539872 0.139777 -0.295367
F 6.596719 1.589698 -1.595434
F 6.365561 -0.525414 -1.987287
C -5.858763 2.160035 -1.052771
H -5.729317 2.530739 -2.074854
H -6.913448 1.906925 -0.915917
H -5.599739 2.970011 -0.368539
42
D_3 Eopt -
1731.217517
C 3.004124 0.558305 0.027904
C 3.646585 1.816460 0.117931
C 2.973220 2.989107 0.463206
C 1.616903 2.904294 0.739994
C 0.968027 1.663554 0.713133
C 1.637239 0.488964 0.381495
C 3.949895 -0.480789 -0.360279
C 5.229527 -0.024484 -0.498198
H 3.498591 3.937352 0.518315
H 1.060104 3.797221 1.005071
H -0.085336 1.609098 0.980670
H -4.151650 -1.184346 1.023963
S 5.353307 1.693283 -0.221034
C 3.633748 -1.887522 -0.762824
O 4.189808 -2.834974 -0.236021
C 2.718876 -2.075937 -1.949026
H 2.131175 -2.990639 -1.841029
H 2.073575 -1.213103 -2.125444
H 3.369052 -2.186814 -2.826109
C 0.859536 -0.823848 0.518166
C 0.522677 -1.068019 1.957544
C -0.328039 -0.844859 -0.391230
C -0.728684 -1.275822 2.394367
H 1.357499 -1.066977 2.654896
C -1.582746 -1.041225 0.041415
H -0.135951 -0.680589 -1.449078
C -1.855574 -1.255315 1.463741
H -0.956283 -1.448294 3.441515
H -2.423915 -1.047851 -0.645004
H 1.506542 -1.659441 0.220252
O -3.014401 -1.420903 1.889015
C -6.229334 0.505263 -0.880204
C -4.977203 0.229443 -0.018721
O -4.992229 -0.992124 0.430951
O -4.141832 1.083971 0.158062
F -7.348722 0.333480 -0.163192
F -6.281909 -0.336552 -1.923227
F -6.226799 1.750101 -1.353155
C 6.463947 -0.782765 -0.875848
H 6.207776 -1.735673 -1.342332
H 7.081758 -0.205976 -1.569260
H 7.063918 -0.996352 0.014848
42
D_4 Eopt -
1731.217439
C 3.037032 0.538237 0.207901
C 3.617032 1.813888 0.407497
C 2.979516 2.835169 1.113474
C 1.717084 2.581261 1.628583
C 1.098225 1.345767 1.401096
C 1.724243 0.327605 0.688277
C 3.929245 -0.323900 -0.556951
C 5.083384 0.295662 -0.941431
H 3.457108 3.800687 1.248642
H 1.193698 3.350854 2.186599
H 0.088253 1.181982 1.770499
H -4.161328 -1.363936 0.555419
S 5.179566 1.937001 -0.358005
C 3.773185 -1.792469 -0.804331
O 3.804177 -2.248869 -1.933438
C 3.730178 -2.692935 0.406648
H 3.110955 -3.571869 0.212167
H 4.760807 -3.028831 0.578256
H 3.400206 -2.170676 1.307075
C 0.901631 -0.929558 0.390463
C 0.498369 -1.630855 1.649890
C -0.250480 -0.582536 -0.501827
C -0.775770 -1.923253 1.951081
H 1.297747 -1.897936 2.337382
C -1.526867 -0.870222 -0.204556
H -0.004627 -0.070049 -1.429202
C -1.862465 -1.554710 1.044149
H -1.051875 -2.430623 2.870173
H -2.338958 -0.603756 -0.873751
H 1.517886 -1.633819 -0.184482
O -3.038685 -1.829739 1.346472
C -6.313430 0.698955 -0.827596
C -5.042545 0.249322 -0.073216
O -5.007279 -1.047738 0.028415
O -4.239254 1.056254 0.330458
F -7.417684 0.296265 -0.182913
F -6.346857 0.169605 -2.059591
F -6.362839 2.024403 -0.945576
C 6.229679 -0.255665 -1.730906
H 6.186076 0.104273 -2.764025
H 7.186198 0.057771 -1.304027
H 6.196048 -1.346385 -1.754652
42
D_5 Eopt -
1731.217518
C -3.001751 0.557745 -0.031228
C -3.641053 1.817387 -0.124149
C -2.965235 2.987178 -0.474265

C -1.609619 2.898051 -0.752816
C -0.963775 1.655865 -0.722508
C -1.635346 0.483964 -0.386182
C -3.949843 -0.477415 0.362086
C -5.227840 -0.017229 0.500920
H -3.488278 3.936591 -0.531555
H -1.050933 3.788631 -1.021828
H 0.089214 1.598260 -0.990866
H 4.152608 -1.191475 -1.016946
S -5.347668 1.700027 0.217566
C -3.636221 -1.884320 0.766669
O -4.191172 -2.831823 0.238861
C -2.723470 -2.071627 1.954633
H -2.138548 -2.988614 1.850910
H -2.075751 -1.210004 2.128442
H -3.375035 -2.176867 2.831413
C -0.859589 -0.830349 -0.518394
C -0.521177 -1.079388 -1.956618
C 0.326792 -0.850121 0.392539
C 0.730501 -1.289766 -2.391288
H -1.355189 -1.079577 -2.654953
C 1.581783 -1.048737 -0.038101
H 0.133566 -0.682597 1.449664
C 1.856346 -1.266818 -1.459470
H 0.959228 -1.465514 -3.437662
H 2.422204 -1.054045 0.649223
H -1.507439 -1.664644 -0.218718
O 3.015771 -1.433396 -1.882666
C 6.224300 0.512683 0.880067
C 4.972906 0.229055 0.019978
O 4.992029 -0.994508 -0.424382
O 4.134948 1.080203 -0.160797
F 6.277819 -0.322854 1.928205
F 6.218950 1.760356 1.345643
F 7.344594 0.338878 0.164870
C -6.463446 -0.770418 0.884436
H -7.066253 -0.198863 1.595329
H -7.078034 -0.964964 -0.000648
H -6.209111 -1.732417 1.332687
42
D_6 Eopt -
1731.217413
C -2.925589 0.546378 -0.228260
C -3.419646 1.848570 -0.482560
C -2.689731 2.815437 -1.175437
C -1.419774 2.478914 -1.619387
C -0.885650 1.215641 -1.336420
C -1.605325 0.250328 -0.638004
C -3.903039 -0.243837 0.510091
C -5.036038 0.450655 0.823308
H -3.102911 3.803334 -1.353894
H -0.825005 3.204894 -2.164164
H 0.131585 0.988039 -1.648550
H 4.150083 -1.531745 -0.385932
S -5.007316 2.079899 0.201310
C -3.846549 -1.711276 0.802567
O -3.965068 -2.132203 1.939623
C -3.793839 -2.647878 -0.380118
H -3.241334 -3.556407 -0.128915
H -4.832421 -2.925662 -0.600582
H -3.383829 -2.173730 -1.273755
C -0.873479 -1.047698 -0.279813
C -0.471598 -1.799909 -1.510099
C 0.268501 -0.746301 0.641549
C 0.795059 -2.155088 -1.772932
H -1.263818 -2.045991 -2.213278
C 1.538147 -1.093010 0.380790
H 0.021336 -0.213942 1.557282
C 1.875947 -1.802315 -0.853548
H 1.070520 -2.693349 -2.674453
H 2.343161 -0.860227 1.071230
H -1.550346 -1.697459 0.291021
O 3.050288 -2.108859 -1.132882
C 6.114821 0.845928 0.743283
C 4.900700 0.205578 0.035224
O 4.964888 -1.093060 0.101462
O 4.044676 0.889518 -0.473476
F 6.109121 0.544011 2.050679
F 6.098522 2.172292 0.625667
F 7.266375 0.394958 0.228113
C -6.247928 -0.014361 1.569612
H -6.213659 0.339175 2.605282
H -7.162806 0.370248 1.111167
H -6.297859 -1.104612 1.589559
42
D_7 Eopt -
1731.217230
C -3.212211 0.474507 -0.122083
C -4.059194 1.471493 -0.662953
C -3.731639 2.827980 -0.662254
C -2.513624 3.203301 -0.115690
C -1.632522 2.232067 0.375812
C -1.948513 0.876596 0.367693
C -3.815661 -0.845750 -0.252591
C -5.018134 -0.830148 -0.899704
H -4.409460 3.564321 -1.082762
H -2.228444 4.250046 -0.092830
H -0.661080 2.541323 0.755446
H 4.157804 0.709448 1.089805
S -5.512684 0.783947 -1.338594
C -3.344588 -2.121757 0.372528
O -3.170372 -3.125060 -0.296160
C -3.247666 -2.156274 1.878735
H -3.137542 -1.162875 2.317869
H -4.195723 -2.574339 2.240101
H -2.438712 -2.817998 2.197524
C -0.853958 -0.101530 0.806573
C 0.295602 -0.035460 -0.151804
C -0.462227 0.125193 2.233319
C 1.559763 0.207107 0.227179
H 0.057982 -0.194381 -1.201236
C 0.799192 0.371285 2.617708
H -1.261413 0.091093 2.970265
C 1.881790 0.434245 1.636285
H 2.369468 0.249756 -0.494949
H 1.065836 0.537805 3.656606
H -1.237148 -1.128635 0.737028
O 3.042155 0.683076 2.013184
C 6.446618 -0.270140 -1.057742
C 5.220891 -0.377482 -0.123408
O 4.990963 0.763157 0.459412
O 4.605743 -1.411810 -0.018982
F 6.199601 0.594887 -2.054089
F 6.746819 -1.448806 -1.599954
F 7.523789 0.171811 -0.395216
C -5.927456 -1.974229 -1.224812
H -6.974084 -1.701089 -1.066377
H -5.808757 -2.265263 -2.273664
H -5.694371 -2.844324 -0.608360
42
D_8 Eopt -
1731.216177
C -3.440932 0.481938 -0.143117
C -4.321109 1.564273 -0.384859
C -3.889300 2.888250 -0.476465
C -2.536776 3.145278 -0.310256
C -1.651314 2.099814 -0.020039
C -2.077110 0.778717 0.082417
C -4.170274 -0.779459 -0.101031
C -5.518062 -0.631612 -0.258906
H -4.594287 3.691666 -0.666307
H -2.164969 4.162375 -0.379674
H -0.601411 2.326305 0.153292
H 4.026576 0.113360 0.596848
S -5.976440 1.031869 -0.515456
C -3.593196 -2.161183 -0.057707
O -3.914912 -2.950344 0.812649
C -2.723613 -2.580258 -1.217869
H -3.389569 -3.057616 -1.948101
H -2.248430 -1.731249 -1.713321
H -1.980768 -3.314175 -0.897033
C -1.041128 -0.259250 0.523555
C 0.076410 -0.364827 -0.466361
C -0.584011 0.033730 1.920321
C 1.368653 -0.215124 -0.137322
H -0.203446 -0.567041 -1.497855
C 0.706562 0.179520 2.256329
H -1.363629 0.124625 2.673386
C 1.757270 0.066708 1.246086
H 2.157419 -0.295829 -0.879595
H 1.020260 0.388782 3.274148
H -1.517739 -1.247655 0.572002
O 2.950004 0.210405 1.572643
C 7.006591 -0.120669 -0.782631
C 5.964962 -0.030275 0.355398
O 4.763026 0.049713 -0.140128
O 6.306560 -0.033375 1.512430
F 6.752133 -1.164285 -1.585269
F 8.239915 -0.260295 -0.300751
F 6.978266 0.987336 -1.538654
C -6.584227 -1.682543 -0.272723
H -7.119070 -1.693021 0.682570
H -7.312625 -1.491491 -1.065361
H -6.151896 -2.673129 -0.425314
42
D_9 Eopt -
1731.217440
C -3.111461 0.545745 -0.022808
C -3.839680 1.759182 -0.059526
C -3.258561 3.001129 0.200311
C -1.908385 3.035677 0.515736
C -1.174960 1.846086 0.605857
C -1.752152 0.603704 0.360403
C -3.974374 -0.586850 -0.334565
C -5.275518 -0.233923 -0.547663
H -3.849532 3.910904 0.161885
H -1.422093 3.984471 0.717980
H -0.127512 1.888109 0.896613
H 4.155333 -0.922572 1.146789
S -5.522026 1.489513 -0.432170
C -3.555003 -2.005166 -0.572927
O -4.046801 -2.921509 0.061350
C -2.614900 -2.261330 -1.725033
H -3.244946 -2.474355 -2.598172
H -2.002650 -1.390591 -1.967931
H -1.992330 -3.136901 -1.527324
C -0.885765 -0.633360 0.609551
C 0.306378 -0.653053 -0.294786

C -0.541253 -0.737426 2.063882
C 1.567130 -0.766072 0.150427
H 0.112161 -0.562693 -1.361222
C 0.716828 -0.856825 2.514273
H -1.376699 -0.716174 2.760098
C 1.843422 -0.878360 1.583332
H 2.410120 -0.776674 -0.533537
H 0.949152 -0.936410 3.571527
H -1.471815 -1.534320 0.382805
O 3.004164 -0.994308 2.019401
C 6.414091 0.294115 -0.909343
C 5.118819 0.250548 -0.068693
O 5.022524 -0.883747 0.561466
O 4.349353 1.181919 -0.060738
F 7.493798 0.124971 -0.133703
F 6.539510 1.456203 -1.547520
F 6.415255 -0.685729 -1.825914
C -6.443714 -1.105793 -0.887802
H -7.043457 -0.662189 -1.687206
H -6.110808 -2.095200 -1.206285
H -7.089147 -1.233624 -0.012653
34
E_1-notfa Eopt -
1204.595245
C 1.153880 0.536206 0.003579
C 2.439835 1.111743 -0.088573
C 2.649780 2.492646 -0.105868
C 1.538016 3.318779 -0.034745
C 0.247032 2.772744 -0.000724
C 0.026977 1.396020 -0.008417
C 1.229075 -0.918587 0.050837
C 2.505175 -1.395229 -0.062618
H 3.652119 2.903761 -0.172961
H 1.665331 4.396731 -0.039384
H -0.612736 3.437050 -0.009237
S 3.682252 -0.110875 -0.173831
C 0.116640 -1.867882 0.366008
O -0.049080 -2.877114 -0.298991
C -0.695681 -1.627970 1.617064
H -0.439831 -2.428729 2.320417
H -0.482985 -0.665792 2.083852
H -1.763039 -1.705903 1.388407
C -1.363787 0.878347 -0.089511
C -2.343426 1.299368 0.820447
C -1.734529 -0.022767 -1.094293
C -3.645569 0.819179 0.748776
H -2.074934 1.995541 1.610818
C -3.036414 -0.510362 -1.180097
H -0.999186 -0.346666 -1.826737
C -3.991817 -0.093067 -0.251860
H -4.400088 1.135938 1.462200
H -3.310019 -1.210195 -1.965679
O -5.277444 -0.537407 -0.276401
C 2.991417 -2.811465 -0.063413
H 2.270130 -3.468866 0.424967
H 3.123406 -3.166331 -1.090884
H 3.952534 -2.896570 0.450407
H -5.404174 -1.156027 -1.011322
42
E_1 Eopt -
1731.260347
C -0.420055 1.584433 0.089503
C 0.471603 2.647090 -0.166610
C 0.200925 3.650080 -1.100266
C -0.999119 3.583210 -1.792016
C -1.882054 2.513617 -1.586434
C -1.607307 1.493188 -0.677771
C 0.098197 0.709716 1.133602
C 1.326069 1.088176 1.603844
H 0.906863 4.455912 -1.275109
H -1.245628 4.350047 -2.519533
H -2.792170 2.452824 -2.176565
H 1.202611 -1.772955 1.129609
S 1.904832 2.535571 0.823533
C -0.626273 -0.419863 1.772583
O -0.099077 -1.532534 1.856596
C -1.941824 -0.171293 2.445166
H -1.748774 -0.208662 3.524670
H -2.358564 0.803678 2.194960
H -2.648415 -0.968957 2.198214
C -2.533314 0.334973 -0.573543
C -3.898096 0.525513 -0.316241
C -2.064618 -0.971257 -0.756853
C -4.766611 -0.554489 -0.215184
H -4.279769 1.532913 -0.171165
C -2.925581 -2.062494 -0.660685
H -1.013555 -1.136696 -0.981886
C -4.277307 -1.853287 -0.382854
H -5.821232 -0.408587 -0.002886
H -2.550222 -3.072839 -0.805037
O -5.166963 -2.876065 -0.269523
C 3.657462 -1.547133 -1.021058
C 2.201390 -1.484687 -0.510973
O 2.135095 -1.917902 0.717246
O 1.312033 -1.064322 -1.209592
F 4.430083 -0.705171 -0.316820
F 3.735175 -1.205133 -2.304750
F 4.167135 -2.778021 -0.883052
C 2.156555 0.500367 2.703648
H 1.638292 -0.325610 3.193540
H 3.104355 0.120494 2.309657
H 2.385239 1.260698 3.456333
H -4.716821 -3.725055 -0.394686
42
E_10 Eopt -
1731.257726
C 2.844732 0.151092 -0.019063
C 4.223468 -0.109200 -0.161458
C 5.173714 0.909997 -0.259650
C 4.723184 2.221507 -0.221644
C 3.351709 2.504220 -0.142235
C 2.394868 1.493628 -0.069572
C 2.087403 -1.087275 0.100079
C 2.858022 -2.211258 -0.016295
H 6.229638 0.681299 -0.364158
H 5.435472 3.037754 -0.289310
H 3.016642 3.537078 -0.179164
H -1.621007 -1.790843 -0.248188
S 4.547776 -1.824890 -0.203827
C 0.657438 -1.197445 0.476991
O -0.084741 -1.947626 -0.160648
C 0.171300 -0.501635 1.715362
H -0.093729 -1.287447 2.432513
H 0.932368 0.143231 2.153496
H -0.736439 0.071269 1.502549
C 0.947872 1.833471 -0.105356
C 0.393679 2.737472 0.811550
C 0.103372 1.255067 -1.060734
C -0.964318 3.031464 0.799065
H 1.031498 3.195027 1.563377
C -1.260114 1.541677 -1.086667
H 0.514021 0.571929 -1.800574
C -1.795731 2.426739 -0.148674
H -1.395783 3.719959 1.518966
H -1.903032 1.087718 -1.837144
O -3.117305 2.745691 -0.115978
C -4.715368 -0.747226 -0.211598
C -3.332491 -1.114383 0.370650
O -2.575996 -1.625912 -0.562139
O -3.070647 -0.916496 1.530098
F -5.522775 -0.269136 0.729590
F -5.302485 -1.810148 -0.773002
F -4.585148 0.193164 -1.165980
C 2.448940 -3.649974 0.039450
H 2.318223 -4.043088 -0.974129
H 3.207915 -4.254462 0.542405
H 1.500754 -3.765831 0.567155
H -3.587865 2.296827 -0.833894
42
E_11 Eopt -
1731.258557
C -2.808869 -0.307573 -0.106036
C -4.208743 -0.258883 -0.275164
C -4.984505 -1.401289 -0.490718
C -4.336354 -2.626142 -0.529647
C -2.954079 -2.707754 -0.304513
C -2.178610 -1.576686 -0.062463
C -2.253538 1.032041 0.057425
C -3.207152 2.014546 0.071563
H -6.059476 -1.329637 -0.623790
H -4.906385 -3.533203 -0.704217
H -2.473519 -3.681939 -0.279792
H 1.447346 0.848320 -0.938223
S -4.807013 1.377051 -0.177788
C -0.809023 1.381292 0.024513
O -0.095894 0.894490 -0.852769
C -0.239008 2.336433 1.032109
H 0.035204 3.268567 0.524540
H -0.934768 2.553641 1.842488
H 0.683198 1.900024 1.428673
C -0.740612 -1.716305 0.280342
C -0.245441 -1.177388 1.472277
C 0.153571 -2.342736 -0.594776
C 1.115539 -1.208237 1.763486
H -0.929358 -0.705010 2.174664
C 1.515906 -2.379199 -0.318471
H -0.215073 -2.766311 -1.525108
C 1.997476 -1.786153 0.849578
H 1.496048 -0.762831 2.679482
H 2.219200 -2.826239 -1.014533
O 3.350159 -1.764739 1.030056
C 4.686659 0.554813 -0.529924
C 3.222771 0.859467 -0.143609
O 2.429918 0.683184 -1.161331
O 2.948319 1.248382 0.966504
F 5.203119 1.611509 -1.183301
F 4.794336 -0.506453 -1.331128
F 5.431981 0.343831 0.555065
C -3.045760 3.499957 0.210566
H -2.150754 3.852052 -0.307219
H -3.904680 4.025888 -0.212245
H -2.966414 3.780237 1.266183
H 3.565326 -1.328126 1.869087
42
E_12 Eopt -
1731.254595
C 2.564700 -0.257743 -0.023360
C 3.735079 -1.009808 -0.255542

C 4.954205 -0.419576 -0.596891
C 4.992579 0.961650 -0.718067
C 3.827262 1.724905 -0.556754
C 2.601652 1.141754 -0.240064
C 1.453938 -1.121533 0.353591
C 1.768690 -2.455250 0.330998
H 5.840330 -1.023723 -0.764692
H 5.924496 1.454642 -0.976185
H 3.865846 2.797811 -0.723195
H -2.200185 -0.795508 1.016951
S 3.440644 -2.721106 -0.071132
C 0.146624 -0.696382 0.896476
O -0.880928 -1.259504 0.503371
C 0.084364 0.292609 2.025805
H -0.311409 -0.245045 2.895781
H 1.062571 0.701440 2.275005
H -0.609504 1.104968 1.785137
C 1.373335 1.977109 -0.195382
C 1.315147 3.129175 0.600730
C 0.248105 1.637282 -0.956635
C 0.162077 3.901853 0.663380
H 2.177425 3.408541 1.200674
C -0.913928 2.404304 -0.905577
H 0.277426 0.764336 -1.604900
C -0.959301 3.533540 -0.085515
H 0.112544 4.785450 1.292120
H -1.779878 2.131654 -1.503884
O -2.064626 4.317858 0.018407
C -3.820546 -1.240518 -0.769865
C -4.125240 -0.551751 0.584276
O -3.095656 -0.435161 1.379525
O -5.240984 -0.171065 0.830014
F -3.309064 -2.464590 -0.574753
F -2.917965 -0.529474 -1.464157
F -4.916460 -1.358993 -1.511969
C 0.905104 -3.638928 0.639949
H 1.504438 -4.476740 1.003717
H 0.156426 -3.389727 1.394854
H 0.374039 -3.962487 -0.260914
H -2.778991 3.966416 -0.534299
42
E_13 Eopt -
1731.255073
C 2.119680 -0.749022 -0.043614
C 2.872905 -1.889013 -0.392311
C 4.078434 -1.809103 -1.093174
C 4.534127 -0.549998 -1.455011
C 3.775132 0.593524 -1.168301
C 2.557868 0.520218 -0.493236
C 0.931233 -1.117912 0.714805
C 0.783715 -2.471252 0.870980
H 4.638394 -2.703786 -1.346840
H 5.470477 -0.450318 -1.994839
H 4.120272 1.563463 -1.515283
H -1.743305 -1.156490 0.235837
S 2.103345 -3.356476 0.157553
C 0.029093 -0.191817 1.441898
O -1.197183 -0.307567 1.351134
C 0.601008 0.797500 2.412336
H 1.687500 0.855781 2.358814
H 0.155080 1.782822 2.245561
H 0.308583 0.458217 3.413401
C 1.737204 1.746445 -0.317270
C 2.279748 2.897551 0.270529
C 0.409565 1.788130 -0.759976
C 1.516617 4.046569 0.438509
H 3.308225 2.883852 0.621805
C -0.365142 2.934990 -0.602745
H -0.025049 0.917560 -1.246650
C 0.187701 4.063333 0.005218
H 1.933573 4.933348 0.905693
H -1.391986 2.958787 -0.957670
O -0.518670 5.209326 0.195604
C -3.943965 -0.240951 -0.336677
C -3.245274 -1.433223 -1.035554
O -2.071795 -1.736841 -0.546975
O -3.784320 -1.999579 -1.951007
F -5.130002 0.015047 -0.877897
F -3.189736 0.865239 -0.433226
F -4.121686 -0.498083 0.967588
C -0.275638 -3.245812 1.591765
H -0.939569 -3.734406 0.871274
H 0.172397 -4.020980 2.219394
H -0.882457 -2.595039 2.223735
H -1.421168 5.107322 -0.142323
42
E_14 Eopt -
1731.255028
C 2.783191 -0.028163 -0.151063
C 4.102447 -0.486439 -0.348839
C 5.133829 0.346853 -0.789828
C 4.830172 1.677887 -1.031032
C 3.541707 2.171872 -0.778527
C 2.514668 1.354223 -0.311784
C 1.907772 -1.118150 0.263048
C 2.563141 -2.309330 0.429084
H 6.138205 -0.037216 -0.938121
H 5.603803 2.352569 -1.383814
H 3.342056 3.231831 -0.909655
H -1.630268 -0.289684 -0.593830
S 4.250761 -2.184563 0.026438
C 0.428862 -1.049721 0.299545
O -0.159562 -0.500086 -0.634705
C -0.339104 -1.655259 1.438655
H -1.081017 -0.926796 1.782905
H -0.883895 -2.534560 1.077275
H 0.306967 -1.936538 2.269820
C 1.210275 1.956301 0.067386
C 0.694104 1.775561 1.357518
C 0.470533 2.713732 -0.844403
C -0.533838 2.315159 1.724063
H 1.261759 1.202489 2.088226
C -0.763680 3.256089 -0.493276
H 0.848817 2.859385 -1.852699
C -1.270026 3.049778 0.791552
H -0.933051 2.174056 2.723925
H -1.338716 3.827272 -1.218088
O -2.471955 3.546885 1.192336
C -4.845983 -0.890821 -0.445451
C -3.335236 -1.200570 -0.530198
O -2.646139 -0.095678 -0.566561
O -2.931349 -2.338328 -0.542801
F -5.131885 -0.264258 0.705857
F -5.570605 -2.006386 -0.500419
F -5.231621 -0.091779 -1.449277
C 2.021536 -3.649108 0.831603
H 1.948926 -3.721367 1.921886
H 1.028849 -3.817906 0.408572
H 2.674793 -4.452824 0.484857
H -2.906575 4.002401 0.455797
42
E_15 Eopt -
1731.254650
C -1.744559 -1.386994 0.029179
C -1.946301 -2.760575 -0.216760
C -3.142362 -3.264258 -0.733156
C -4.156978 -2.360943 -1.013749
C -3.958081 -0.984465 -0.834006
C -2.758277 -0.471352 -0.344296
C -0.428747 -1.145389 0.606144
C 0.329471 -2.280241 0.726840
H -3.271730 -4.327488 -0.909454
H -5.102077 -2.718656 -1.409871
H -4.742290 -0.290427 -1.123114
H 1.886532 0.024114 -0.252070
S -0.533846 -3.698520 0.200073
C 0.046427 0.121929 1.212886
O 1.171033 0.561755 0.955390
C -0.781007 0.800456 2.263153
H -1.774451 0.364126 2.360053
H -0.242120 0.681001 3.210776
H -0.850820 1.870823 2.046450
C -2.546651 0.998188 -0.287866
C -1.434878 1.580929 -0.913387
C -3.455676 1.836241 0.367628
C -1.219497 2.953575 -0.865699
H -0.732400 0.952648 -1.456612
C -3.252436 3.212253 0.423894
H -4.322931 1.405123 0.861024
C -2.127562 3.770897 -0.189318
H -0.358479 3.402457 -1.351092
H -3.959466 3.851893 0.946806
O -1.871052 5.106043 -0.163318
C 4.240739 0.662852 -0.064843
C 3.596748 -0.098851 -1.250120
O 2.315204 -0.319386 -1.120816
O 4.268722 -0.434336 -2.191073
F 3.684608 1.875292 0.071878
F 5.548336 0.818994 -0.238976
F 4.045839 -0.004571 1.083648
C 1.707996 -2.454529 1.284691
H 2.007700 -1.584926 1.872227
H 1.758482 -3.340284 1.923653
H 2.431353 -2.585079 0.472437
H -2.576299 5.572610 0.309907
42
E_16 Eopt -
1731.257542
C -2.840376 -0.275946 -0.047751
C -4.242159 -0.192924 -0.193325
C -5.057526 -1.319241 -0.331940
C -4.449527 -2.564734 -0.320883
C -3.063989 -2.677639 -0.133736
C -2.247436 -1.561359 0.028987
C -2.246552 1.054504 0.044261
C -3.171059 2.063128 0.010638
H -6.132337 -1.218638 -0.446249
H -5.051408 -3.460962 -0.433231
H -2.610255 -3.663588 -0.083560
H 1.469758 1.031889 -0.561562
S -4.792092 1.462217 -0.184623
C -0.793812 1.374957 0.037481
O -0.060977 0.891293 -0.825449
C -0.231451 2.294684 1.086806
H 0.497668 1.722440 1.672347
H 0.305449 3.119768 0.608133
H -0.996466 2.682058 1.758413

C -0.795987 -1.734073 0.291675
C -0.208700 -1.197890 1.444342
C 0.020578 -2.387831 -0.634560
C 1.166659 -1.255327 1.640999
H -0.834424 -0.706442 2.186955
C 1.399322 -2.449763 -0.453109
H -0.419010 -2.810753 -1.533739
C 1.971691 -1.855992 0.671895
H 1.630146 -0.817347 2.520104
H 2.033565 -2.920578 -1.199909
O 3.323939 -1.797641 0.852319
C 4.711545 0.705996 -0.167432
C 3.325653 0.486114 -0.812618
O 2.409673 1.174409 -0.188268
O 3.209407 -0.203930 -1.794826
F 5.526650 -0.317575 -0.428709
F 4.649135 0.869350 1.154864
F 5.269200 1.815236 -0.687597
C -2.964254 3.548078 0.066361
H -3.735319 4.067494 -0.507706
H -3.019277 3.905004 1.100408
H -1.994083 3.831328 -0.347280
H 3.772558 -2.301742 0.155067
34
E_2-notfa Eopt -
1204.595019
C -1.152336 0.538201 0.005662
C -2.435510 1.120745 -0.082150
C -2.638560 2.502702 -0.096478
C -1.522468 3.323058 -0.027423
C -0.234279 2.770245 0.002172
C -0.021052 1.392417 -0.007992
C -1.235322 -0.916260 0.050242
C -2.514408 -1.385692 -0.061226
H -3.638990 2.918928 -0.160344
H -1.644227 4.401658 -0.030438
H 0.628796 3.430188 -0.008655
S -3.684690 -0.094889 -0.166828
C -0.128404 -1.873290 0.360996
O 0.025993 -2.884589 -0.303363
C 0.692324 -1.638661 1.607781
H 0.437380 -2.439487 2.311324
H 1.757771 -1.721127 1.371956
H 0.486540 -0.676186 2.077040
C 1.367201 0.868778 -0.094234
C 1.731878 -0.033631 -1.103286
C 2.350076 1.284706 0.811018
C 3.029655 -0.524309 -1.195926
H 0.991462 -0.352942 -1.832666
C 3.652542 0.799705 0.732127
H 2.088692 1.980376 1.604181
C 3.991203 -0.111569 -0.271228
H 3.309079 -1.223073 -1.978276
H 4.404013 1.121405 1.448797
O 5.245450 -0.623610 -0.397209
C -3.008529 -2.799188 -0.064331
H -3.971830 -2.879147 0.446181
H -3.138821 -3.152778 -1.092441
H -2.292526 -3.460906 0.425972
H 5.822088 -0.270824 0.296851
42
E_2 Eopt -
1731.259188
C -1.252126 -1.605235 -0.150413
C -1.037127 -2.924471 -0.597744
C -1.779502 -3.497387 -1.633001
C -2.756518 -2.718687 -2.236053
C -2.944057 -1.383076 -1.852008
C -2.188510 -0.795811 -0.838737
C -0.375697 -1.279190 0.967469
C 0.494000 -2.288734 1.287968
H -1.595727 -4.517707 -1.954455
H -3.356838 -3.134729 -3.038965
H -3.666330 -0.770947 -2.385028
H 1.762311 0.380831 1.393307
S 0.248253 -3.699168 0.295282
C -0.490386 -0.098236 1.857454
O 0.512276 0.538079 2.196173
C -1.813319 0.239997 2.477408
H -1.721404 0.025736 3.548873
H -2.013506 1.309721 2.362799
H -2.632619 -0.347634 2.064848
C -2.321143 0.655825 -0.554088
C -1.188306 1.478569 -0.545842
C -3.570074 1.240348 -0.304193
C -1.285948 2.839019 -0.269016
H -0.209449 1.058504 -0.764314
C -3.683272 2.597611 -0.026545
H -4.462017 0.619032 -0.302472
C -2.537020 3.397969 -0.002016
H -0.392810 3.458732 -0.262330
H -4.648784 3.048720 0.180574
O -2.700370 4.717549 0.283929
C 4.000339 0.980989 -0.921445
C 2.747417 1.220162 -0.050630
O 2.618011 0.264832 0.827431
O 2.030954 2.173312 -0.236301
F 5.091035 0.802670 -0.164923
F 4.223238 2.008835 -1.737507
F 3.840383 -0.120462 -1.670788
C 1.532703 -2.353945 2.364259
H 2.529006 -2.205316 1.934829
H 1.371016 -1.580859 3.117726
H 1.519993 -3.329553 2.857211
H -1.842484 5.168306 0.275020
34
E_3-notfa Eopt -
1204.591487
C -1.152983 0.554393 0.022358
C -2.438660 1.124553 -0.105757
C -2.649438 2.504925 -0.167020
C -1.539984 3.333607 -0.101180
C -0.248430 2.789987 -0.030919
C -0.026664 1.414856 0.003099
C -1.227172 -0.896112 0.124228
C -2.498628 -1.378483 0.015570
H -3.651105 2.912716 -0.260437
H -1.667983 4.410928 -0.136931
H 0.610931 3.454866 -0.043661
S -3.676358 -0.102064 -0.162534
C -0.084446 -1.794094 0.509134
O 0.524080 -1.583959 1.542241
C 0.246943 -2.968155 -0.376993
H 0.032769 -2.755904 -1.427226
H 1.297925 -3.233362 -0.244618
H -0.362569 -3.826977 -0.073473
C 1.364263 0.896035 -0.056680
C 1.742833 -0.000441 -1.064942
C 2.332859 1.308159 0.862992
C 3.042518 -0.487249 -1.146766
H 1.009334 -0.316755 -1.803711
C 3.638264 0.826263 0.795853
H 2.060013 1.998059 1.657020
C 3.991759 -0.076853 -0.208313
H 3.334003 -1.180113 -1.930315
H 4.379975 1.143935 1.524494
O 5.250100 -0.584214 -0.324203
C -2.979010 -2.798973 0.079367
H -2.452156 -3.353214 0.860529
H -4.047566 -2.842039 0.302251
H -2.813865 -3.307588 -0.876203
H 5.814448 -0.240645 0.384339
42
E_3 Eopt -
1731.260029
C 0.427034 1.587582 0.088503
C -0.459330 2.653095 -0.174205
C -0.181353 3.652135 -1.109988
C 1.020575 3.577985 -1.797641
C 1.898016 2.505068 -1.586086
C 1.616229 1.488587 -0.675094
C -0.098819 0.718180 1.133342
C -1.327400 1.102667 1.597024
H -0.883524 4.460103 -1.290159
H 1.272451 4.341182 -2.527150
H 2.808729 2.437669 -2.174534
H -1.214463 -1.763075 1.138244
S -1.897126 2.549954 0.810231
C 0.618325 -0.411681 1.779829
O 0.084612 -1.520515 1.870329
C 1.935123 -0.167225 2.451919
H 1.741270 -0.197076 3.531490
H 2.636120 -0.971245 2.209808
H 2.358153 0.803623 2.196245
C 2.534716 0.324659 -0.568520
C 2.055144 -0.980632 -0.747499
C 3.899776 0.505303 -0.317911
C 2.906575 -2.076420 -0.654487
H 1.001931 -1.137358 -0.968741
C 4.761973 -0.583282 -0.220232
H 4.291164 1.509374 -0.175746
C 4.261901 -1.877987 -0.384471
H 2.534766 -3.086825 -0.794321
H 5.818596 -0.431917 -0.013016
O 5.052736 -2.981270 -0.295158
C -3.664132 -1.553162 -1.020047
C -2.209849 -1.481600 -0.506157
O -2.145148 -1.911067 0.723472
O -1.321002 -1.057766 -1.203261
F -3.740058 -1.216016 -2.305148
F -4.442485 -0.711963 -0.320885
F -4.168654 -2.785698 -0.878883
C -2.166060 0.520904 2.693886
H -2.402255 1.285801 3.439570
H -3.109976 0.137117 2.294360
H -1.650940 -0.301298 3.193368
H 5.972532 -2.722371 -0.134923
34
E_4-notfa Eopt -
1204.591235
C -1.152649 0.553580 0.020581
C -2.437321 1.124897 -0.113161
C -2.646747 2.505305 -0.177292
C -1.536907 3.333146 -0.107891
C -0.246216 2.788458 -0.031797
C -0.025573 1.413133 0.004763
C -1.229077 -0.896707 0.124551
C -2.500807 -1.377701 0.012637

H -3.647737 2.913763 -0.274968
H -1.663757 4.410543 -0.145251
H 0.613684 3.452701 -0.041227
S -3.676293 -0.100309 -0.172048
C -0.089036 -1.795745 0.514580
O 0.516514 -1.586024 1.549363
C 0.243294 -2.971393 -0.369353
H -0.370320 -3.828202 -0.068482
H 0.034785 -2.759232 -1.420771
H 1.292671 -3.239901 -0.231016
C 1.366068 0.895328 -0.048277
C 1.749637 -0.002893 -1.049862
C 2.332764 1.315769 0.873221
C 3.054231 -0.484364 -1.126474
H 1.020439 -0.326590 -1.789658
C 3.638499 0.841117 0.811462
H 2.053677 2.008112 1.662966
C 3.999455 -0.063743 -0.189196
H 3.339272 -1.179605 -1.912436
H 4.384324 1.157743 1.534021
O 5.289636 -0.498894 -0.206805
C -2.983395 -2.797404 0.078036
H -2.459747 -3.350622 0.862072
H -4.052669 -2.838349 0.297838
H -2.816231 -3.308427 -0.875897
H 5.425765 -1.112350 -0.944057
42
E_4 Eopt -
1731.258604
C 1.299407 1.611487 0.009471
C 0.933584 2.947922 -0.251482
C 1.746870 3.822921 -0.975129
C 2.956881 3.338596 -1.450043
C 3.317046 1.998252 -1.250961
C 2.498584 1.111213 -0.553862
C 0.289109 0.938751 0.816501
C -0.798093 1.724465 1.091684
H 1.440368 4.847853 -1.158857
H 3.616666 3.993463 -2.010340
H 4.239825 1.623662 -1.685033
H -1.574184 -1.052328 0.418250
S -0.626326 3.329549 0.433542
C 0.436768 -0.383642 1.476522
O -0.456417 -1.231814 1.403355
C 1.619875 -0.640005 2.361248
H 2.376313 0.140517 2.285632
H 1.239025 -0.671453 3.389181
H 2.052514 -1.618285 2.130621
C 2.866686 -0.325881 -0.469946
C 1.970423 -1.315985 -0.889678
C 4.122168 -0.724241 0.009824
C 2.300126 -2.666869 -0.810144
H 1.002363 -1.030067 -1.294937
C 4.464445 -2.068248 0.095300
H 4.831129 0.029266 0.343073
C 3.547807 -3.043125 -0.309847
H 1.593869 -3.424641 -1.139816
H 5.431863 -2.377358 0.478847
O 3.928830 -4.343775 -0.200679
C -4.573527 -0.922585 -0.883927
C -3.493712 -1.168585 0.192665
O -2.307427 -0.931723 -0.295731
O -3.790349 -1.515557 1.309067
F -4.580957 0.368023 -1.251189
F -5.786257 -1.230828 -0.430009
F -4.338064 -1.655359 -1.980291
C -2.017590 1.426717 1.907223
H -2.152534 2.182195 2.686957
H -2.910790 1.444995 1.273362
H -1.952840 0.446511 2.382294
H 3.216893 -4.927623 -0.503105
42
E_5 Eopt -
1731.259041
C 2.881585 -0.048909 0.070979
C 4.195276 -0.560007 0.081285
C 5.318972 0.258751 0.220107
C 5.114285 1.625399 0.342451
C 3.821189 2.164058 0.267523
C 2.698887 1.356340 0.101160
C 1.900538 -1.121600 -0.046928
C 2.467186 -2.361012 -0.191274
H 6.319449 -0.162112 0.228187
H 5.965381 2.288908 0.458620
H 3.688186 3.242024 0.294584
H -1.790971 -1.606762 -0.263719
S 4.205154 -2.295612 -0.113128
C 0.439473 -1.017835 0.166755
O -0.333717 -1.625081 -0.582820
C -0.089432 -0.316660 1.385937
H -0.853549 0.415133 1.100028
H -0.564112 -1.080975 2.013010
H 0.696530 0.173772 1.958511
C 1.354087 1.950001 -0.105309
C 0.800720 2.860231 0.803070
C 0.589233 1.565419 -1.214902
C -0.509496 3.307236 0.657953
H 1.382531 3.179806 1.663887
C -0.716610 2.011164 -1.375554
H 1.016352 0.895778 -1.957567
C -1.283629 2.846663 -0.410486
H -0.956536 3.976509 1.386635
H -1.308932 1.685695 -2.225340
O -2.583013 3.242716 -0.477066
C -4.949626 -0.853840 0.269979
C -3.508768 -0.657483 -0.252436
O -2.781842 -1.699665 0.025962
O -3.187972 0.355879 -0.826608
F -4.951574 -1.017808 1.600312
F -5.509279 -1.942015 -0.275690
F -5.713271 0.197224 -0.020276
C 1.803885 -3.694468 -0.348983
H 0.856394 -3.726794 0.193227
H 1.589702 -3.885783 -1.405159
H 2.445709 -4.497424 0.020637
H -3.075664 2.623933 -1.038309
42
E_6 Eopt -
1731.258299
C -1.291791 1.613383 0.011149
C -0.920832 2.949415 -0.245087
C -1.730425 3.830397 -0.965560
C -2.942215 3.352758 -1.442535
C -3.308050 2.013367 -1.247545
C -2.493645 1.120419 -0.553153
C -0.283489 0.933936 0.815178
C 0.806345 1.715002 1.093384
H -1.419475 4.854629 -1.145666
H -3.599092 4.012177 -2.000907
H -4.232284 1.644073 -1.683037
H 1.578698 -1.061031 0.408246
S 0.640156 3.323107 0.441492
C -0.433544 -0.391151 1.469451
O 0.457912 -1.240411 1.392102
C -1.616608 -0.649574 2.353937
H -1.234627 -0.689845 3.381107
H -2.370384 0.134079 2.284612
H -2.052224 -1.624874 2.116440
C -2.869967 -0.314786 -0.472663
C -4.124757 -0.705803 0.008245
C -1.979770 -1.310965 -0.898566
C -4.475319 -2.050328 0.089950
H -4.829232 0.049842 0.346080
C -2.316871 -2.657888 -0.823295
H -1.011304 -1.028216 -1.305176
C -3.565540 -3.028788 -0.320225
H -5.448319 -2.343317 0.476848
H -1.625992 -3.427234 -1.153667
O -3.848244 -4.357391 -0.256778
C 4.582562 -0.934718 -0.882737
C 3.498470 -1.179712 0.189788
O 2.314432 -0.939770 -0.302489
O 3.790609 -1.527831 1.307019
F 5.792882 -1.247590 -0.425407
F 4.595188 0.356989 -1.246506
F 4.348759 -1.663731 -1.981729
C 2.024607 1.410643 1.908374
H 2.918215 1.429359 1.275142
H 2.160862 2.162682 2.691209
H 1.957194 0.428653 2.379314
H -4.738613 -4.492118 0.100978
42
E_7 Eopt -
1731.258889
C -0.309613 0.879948 0.911610
C -1.536655 1.180035 1.539527
C -2.059853 0.409898 2.581874
C -1.325211 -0.686401 3.007085
C -0.073686 -0.969286 2.439823
C 0.467326 -0.191336 1.418345
C -0.035261 1.811076 -0.175096
C -0.977009 2.798583 -0.291480
H -3.010640 0.662756 3.040504
H -1.705969 -1.314119 3.806622
H 0.513209 -1.796367 2.829738
H 0.143685 -0.644476 -1.507553
S -2.270111 2.603262 0.851760
C 0.970708 1.606548 -1.246369
O 1.083209 0.504882 -1.789209
C 1.822996 2.745340 -1.720354
H 1.930644 3.521503 -0.961138
H 2.799283 2.352644 -2.013298
H 1.358061 3.184126 -2.610988
C 1.845626 -0.463824 0.936290
C 2.810226 0.553541 0.924619
C 2.220250 -1.739965 0.506188
C 4.106979 0.311355 0.486612
H 2.545120 1.549743 1.273259
C 3.514782 -1.995886 0.059420
H 1.485380 -2.540418 0.499677
C 4.458309 -0.967182 0.046701
H 4.854561 1.098744 0.483506
H 3.790673 -2.989611 -0.284888
O 5.737859 -1.155428 -0.378039
C -2.593714 -2.314429 -0.878391
C -1.721427 -1.109394 -1.292859
O -0.463971 -1.454628 -1.326948
O -2.215219 -0.036901 -1.539955

F -2.211503 -2.796028 0.312269
F -2.483352 -3.308791 -1.772896
F -3.878387 -1.974103 -0.795417
C -1.072266 3.907815 -1.297646
H -1.009834 3.513486 -2.316473
H -2.020738 4.440244 -1.202495
H -0.263267 4.630592 -1.157673
H 5.860721 -2.072748 -0.664746
42
E_8 Eopt -
1731.258945
C 2.864782 0.210945 -0.020027
C 4.253187 0.010892 -0.151361
C 5.155954 1.075002 -0.223750
C 4.645258 2.364466 -0.170090
C 3.261032 2.586662 -0.105837
C 2.353526 1.531396 -0.061968
C 2.154992 -1.056481 0.084878
C 2.969095 -2.146661 -0.032742
H 6.222603 0.897387 -0.318500
H 5.321813 3.212127 -0.215227
H 2.879943 3.603739 -0.134907
H -1.637426 -1.694619 -0.188097
S 4.646181 -1.690792 -0.213325
C 0.731099 -1.204364 0.488218
O -0.035222 -1.918022 -0.158224
C 0.291253 -0.573829 1.777475
H 0.134809 -1.397926 2.485021
H 1.037867 0.106511 2.186369
H -0.667049 -0.063486 1.645627
C 0.888354 1.771432 -0.136210
C 0.217324 2.571819 0.794939
C 0.138776 1.152664 -1.146928
C -1.169387 2.699506 0.762192
H 0.779597 3.064240 1.584187
C -1.243602 1.280712 -1.199318
H 0.644864 0.550855 -1.897875
C -1.901104 2.030727 -0.223023
H -1.685604 3.293643 1.511813
H -1.823004 0.793343 -1.977655
O -3.260688 2.066528 -0.284967
C -4.727714 -0.643646 -0.196530
C -3.305827 -0.866285 0.363511
O -2.586608 -1.543368 -0.494344
O -2.997032 -0.481324 1.462587
F -5.402339 0.237907 0.537716
F -5.404641 -1.805983 -0.168060
F -4.708094 -0.221273 -1.462851
C 2.617176 -3.599757 0.028363
H 2.658996 -4.040699 -0.973017
H 3.315724 -4.146485 0.667670
H 1.605167 -3.736778 0.412857
H -3.620171 2.553259 0.472516
42
E_9 Eopt -
1731.258350
C 2.817717 -0.197137 0.079096
C 4.072463 -0.838339 0.140279
C 5.259827 -0.145023 0.387064
C 5.182275 1.227995 0.568168
C 3.954599 1.895807 0.450250
C 2.769190 1.215456 0.178890
C 1.749430 -1.159769 -0.153138
C 2.197997 -2.442363 -0.322980
H 6.211497 -0.664912 0.433263
H 6.084755 1.796812 0.768443
H 3.926671 2.978833 0.531649
H -1.908647 -1.049508 -0.835145
S 3.925457 -2.556303 -0.137370
C 0.295674 -0.901597 -0.057852
O -0.458144 -1.357032 -0.924390
C -0.262110 -0.227242 1.162779
H -0.834860 -0.989024 1.705988
H 0.519148 0.161958 1.814114
H -0.947544 0.578965 0.879164
C 1.511558 1.973239 -0.051181
C 1.044311 2.900389 0.890367
C 0.763048 1.776257 -1.218023
C -0.150699 3.582020 0.694771
H 1.610416 3.067203 1.803128
C -0.436555 2.452392 -1.427437
H 1.121164 1.086555 -1.978754
C -0.900326 3.347929 -0.461949
H -0.521650 4.286602 1.432648
H -1.009847 2.285959 -2.336086
O -2.070047 4.027470 -0.597111
C -4.981323 -0.752572 0.261275
C -3.529875 -1.276955 0.201584
O -2.901431 -0.757785 -0.815218
O -3.108602 -2.048343 1.028894
F -4.989700 0.573977 0.461688
F -5.661363 -1.325864 1.251409
F -5.629200 -0.993573 -0.886450
C 1.416868 -3.691999 -0.587561
H 1.322644 -3.855690 -1.665925
H 1.912893 -4.562882 -0.152593
H 0.409788 -3.618868 -0.172075
H -2.519994 3.762570 -1.413528
35
F_1-notfa Eopt -
1204.949830
C -0.819082 -0.603871 -0.283482
C -1.946388 -0.510018 0.536369
C -2.869860 -1.537819 0.689079
C -2.631827 -2.715204 -0.016218
C -1.508661 -2.838041 -0.839494
C -0.600792 -1.788973 -0.983012
C 0.008950 0.654044 -0.316810
C -0.571303 1.577635 0.716529
H -3.734495 -1.433653 1.336099
H -3.325053 -3.544066 0.080491
H -1.337493 -3.765084 -1.376719
H 0.259798 -1.897368 -1.635550
S -2.019425 1.057970 1.347755
O 0.981483 0.161062 2.198395
C 1.753413 -0.141726 1.295239
C 2.909665 -1.008985 1.491271
C 1.528253 0.433007 -0.099682
C 3.580106 -1.492132 0.422311
H 3.143466 -1.307032 2.508015
C 2.240834 -0.278764 -1.212490
H 1.993871 1.435207 -0.061172
C 3.227579 -1.148407 -0.950379
H 4.403772 -2.183639 0.577516
H 1.994981 -0.010687 -2.236959
H 3.783558 -1.610550 -1.759141
C -0.177028 1.476291 -1.686976
O 0.653802 2.303294 -1.955666
C -1.366107 1.160466 -2.536877
H -2.278974 1.111913 -1.935193
H -1.458330 1.922165 -3.311099
H -1.227215 0.177378 -2.999500
C -0.004860 2.895392 1.076116
H 0.248263 3.455807 0.169765
H -0.683758 3.477619 1.699727
H 0.928788 2.732264 1.628838
42
F_1 Eopt -
1731.155602
C 1.132948 0.786279 0.542208
C -0.175810 0.867572 0.065732
C -0.936946 2.028609 0.120766
C -0.340452 3.156310 0.681279
C 0.964710 3.100118 1.175749
C 1.703725 1.918120 1.118966
C 1.739992 -0.590748 0.420394
C 0.762961 -1.396455 -0.404807
H -1.949545 2.037837 -0.266851
H -0.901075 4.083832 0.735708
H 1.412534 3.985652 1.615232
H 2.708370 1.888259 1.526575
S -0.706143 -0.669811 -0.637146
O 2.121162 -0.440464 -2.314994
C 3.108825 -0.163557 -1.645312
C 4.266877 0.538383 -2.190650
C 3.167992 -0.609616 -0.185266
C 5.241160 0.982556 -1.367502
H 4.265544 0.746036 -3.255687
C 4.225119 0.059318 0.645384
H 3.485019 -1.666017 -0.260236
C 5.212125 0.765026 0.074178
H 6.072987 1.546579 -1.780632
H 4.208760 -0.093058 1.722317
H 6.002820 1.196198 0.678999
C 1.735725 -1.302106 1.851599
O 1.096468 -0.801382 2.739859
C 2.542202 -2.558586 2.043086
H 2.564902 -3.196597 1.159148
H 3.574984 -2.279895 2.280415
H 2.127907 -3.103660 2.892069
C 0.971584 -2.801153 -0.845118
H 0.648214 -3.479235 -0.044529
H 0.377102 -3.021544 -1.733719
H 2.024924 -2.996033 -1.056051
C -5.302099 -0.143356 -0.162751
C -3.757715 0.070298 -0.182918
O -3.148545 -0.227244 0.857811
O -3.334168 0.519974 -1.266259
F -5.693643 -0.991791 -1.137428
F -5.960909 1.015748 -0.375554
F -5.765674 -0.644014 0.992999
42
F_10 Eopt -
1731.161627
C -0.674166 -0.592164 0.181822
C 0.353938 -0.469918 -0.753580
C 1.293192 -1.465514 -0.993880
C 1.182088 -2.639118 -0.252836
C 0.164186 -2.787596 0.694144
C -0.762888 -1.771219 0.921618
C -1.542777 0.637076 0.284522
C -1.085659 1.571078 -0.807567
H 2.083544 -1.332302 -1.726053
H 1.898325 -3.439055 -0.408866
H 0.097040 -3.705541 1.268707
H -1.532897 -1.897787 1.675375
S 0.301463 1.094431 -1.567821
O -2.815625 0.270263 -2.159627

C -3.416071 -0.168037 -1.183948
C -4.487026 -1.153749 -1.290234
C -3.064249 0.358072 0.202953
C -4.999454 -1.723268 -0.177371
H -4.795530 -1.447554 -2.288272
C -3.619028 -0.426709 1.354428
H -3.568744 1.338352 0.262263
C -4.551604 -1.371765 1.164979
H -5.765528 -2.488075 -0.274092
H -3.295870 -0.155626 2.356093
H -4.989319 -1.892245 2.010270
C -1.249506 1.479763 1.612078
O -2.129576 2.188374 2.032852
C 0.108913 1.349331 2.217790
H 0.194003 0.370400 2.702050
H 0.901481 1.388393 1.459067
H 0.246150 2.137024 2.959347
C -1.675337 2.909136 -1.056638
H -1.337495 3.595306 -0.268857
H -1.360721 3.309878 -2.021159
H -2.766126 2.868084 -1.016612
C 4.520675 -0.206885 -0.124321
C 3.315592 0.496377 0.581191
O 3.125976 0.107019 1.743001
O 2.734248 1.362524 -0.102900
F 5.694108 0.243191 0.374994
F 4.567732 -0.004400 -1.453073
F 4.514068 -1.542670 0.060119
35
F_2-notfa Eopt -
1204.944382
C 0.823067 -0.573523 0.347049
C 2.012946 -0.488978 -0.374973
C 2.971898 -1.494270 -0.393625
C 2.702440 -2.640872 0.350690
C 1.517237 -2.751604 1.082576
C 0.576638 -1.720696 1.094722
C -0.025744 0.669437 0.261021
C 0.602873 1.540882 -0.787131
H 3.888396 -1.395857 -0.965552
H 3.421306 -3.453431 0.357835
H 1.323019 -3.653345 1.653992
H -0.331325 -1.818194 1.681339
S 2.111196 1.035051 -1.266277
O -0.804634 -0.120780 -2.242661
C -1.623595 -0.357602 -1.363108
C -2.725468 -1.299628 -1.526543
C -1.522173 0.392895 -0.034208
C -3.462671 -1.671112 -0.457137
H -2.865342 -1.737123 -2.509701
C -2.299962 -0.203074 1.103587
H -2.018112 1.355042 -0.259591
C -3.234233 -1.139222 0.882021
H -4.245793 -2.414055 -0.581581
H -2.141519 0.190992 2.105570
H -3.834199 -1.523128 1.700263
C 0.158946 1.504648 1.632512
O 1.047904 1.175959 2.370497
C -0.744349 2.669347 1.928804
H -1.675093 2.677949 1.363696
H -0.956732 2.662888 3.000040
H -0.182817 3.583870 1.704165
C 0.006013 2.779205 -1.335183
H -0.564463 3.318935 -0.576420
H 0.754160 3.434996 -1.782383
H -0.699949 2.473213 -2.120369
42
F_2 Eopt -
1731.159914
C 1.035510 1.023103 0.319732
C 0.400215 1.812086 -0.636486
C 0.381596 3.200287 -0.597610
C 1.041364 3.818274 0.462573
C 1.680934 3.052792 1.440601
C 1.676106 1.658734 1.381211
C 0.894330 -0.462183 0.082745
C 0.165642 -0.610386 -1.233249
H -0.129647 3.780225 -1.359114
H 1.050973 4.901318 0.528808
H 2.183281 3.547106 2.265769
H 2.157230 1.083277 2.164048
S -0.353457 0.824496 -1.886076
O 2.541014 -0.484793 -2.197734
C 3.103184 -0.733224 -1.138327
C 4.551032 -0.633203 -0.973700
C 2.272684 -1.209921 0.053401
C 5.112076 -0.777059 0.245749
H 5.131416 -0.378816 -1.854662
C 2.997742 -1.230193 1.369066
H 2.066595 -2.270189 -0.179389
C 4.326656 -1.066570 1.439347
H 6.186257 -0.657855 0.358549
H 2.426877 -1.448557 2.268534
H 4.839726 -1.141494 2.392288
C 0.071657 -1.134318 1.280533
O -0.173778 -0.464532 2.247949
C -0.152292 -2.621668 1.257811
H -0.176229 -3.054624 0.260411
H 0.665926 -3.086307 1.821518
H -1.090730 -2.825867 1.773609
C -0.124753 -1.875637 -1.954510
H -1.067720 -2.277718 -1.567629
H -0.239987 -1.689671 -3.023844
H 0.667642 -2.611199 -1.804796
C -4.420541 -0.271023 0.175443
C -2.883971 -0.001686 0.161152
O -2.530618 1.179465 0.255315
O -2.212214 -1.049649 0.028944
F -4.763761 -1.156128 1.134022
F -4.830374 -0.799314 -0.999514
F -5.159928 0.829363 0.381539
42
F_3 Eopt -
1731.164246
C 0.974035 0.754011 0.300190
C -0.173549 0.881534 -0.485713
C -0.908973 2.058361 -0.568506
C -0.454267 3.147977 0.171192
C 0.692179 3.045849 0.964716
C 1.408448 1.851748 1.040478
C 1.572072 -0.627844 0.270860
C 0.832400 -1.378719 -0.803220
H -1.804448 2.108514 -1.177112
H -1.000516 4.084600 0.128960
H 1.030353 3.906218 1.533203
H 2.288961 1.781890 1.671510
S -0.524023 -0.617742 -1.360375
O 2.692122 -0.138649 -2.219741
C 3.464643 -0.034552 -1.274506
C 4.749347 0.654270 -1.377429
C 3.106000 -0.671218 0.065782
C 5.467695 0.925918 -0.266478
H 5.049966 0.998159 -2.361843
C 3.912722 -0.192748 1.238315
H 3.383972 -1.734768 -0.052671
C 5.035548 0.520036 1.065632
H 6.395320 1.485294 -0.353192
H 3.604378 -0.507161 2.231544
H 5.641761 0.807798 1.918196
C 1.205665 -1.461139 1.586591
O 1.896538 -2.410842 1.859452
C 0.013818 -1.024116 2.374888
H 0.239561 -0.075060 2.873423
H -0.851375 -0.851244 1.721128
H -0.214633 -1.787105 3.119460
C 1.193154 -2.746143 -1.244272
H 1.395381 -3.384859 -0.378049
H 0.414445 -3.194804 -1.862231
H 2.116988 -2.684504 -1.832783
C -5.029866 -0.064769 0.107650
C -3.509462 -0.159280 -0.227243
O -2.782040 -0.623931 0.666792
O -3.230000 0.260379 -1.366937
F -5.776701 -0.736973 -0.792563
F -5.454395 1.217003 0.079980
F -5.349835 -0.551119 1.316621
42
F_4 Eopt -
1731.160878
C 1.106858 0.957412 0.299766
C 0.570469 1.754402 -0.711623
C 0.653619 3.141727 -0.722206
C 1.315280 3.749816 0.342270
C 1.861955 2.976974 1.369525
C 1.759643 1.585233 1.359556
C 0.889946 -0.523269 0.090036
C 0.166356 -0.664300 -1.224525
H 0.219898 3.727828 -1.526050
H 1.401828 4.831031 0.371076
H 2.370290 3.464679 2.194985
H 2.174454 1.003553 2.173800
S -0.195783 0.779097 -1.964150
O 2.596947 -0.645182 -2.210628
C 3.106129 -0.764912 -1.102842
C 4.495531 -0.405367 -0.832675
C 2.255198 -1.323738 0.040624
C 5.014009 -0.558649 0.403777
H 5.069565 0.001844 -1.658726
C 2.947213 -1.422922 1.369243
H 2.017421 -2.354826 -0.263126
C 4.238491 -1.094952 1.517324
H 6.046237 -0.275627 0.591280
H 2.385646 -1.826675 2.207505
H 4.729620 -1.219360 2.476843
C 0.074290 -1.151089 1.311021
O 0.087888 -0.547296 2.350987
C -0.517555 -2.523876 1.172644
H -1.394604 -2.489784 0.520256
H 0.206617 -3.228018 0.751616
H -0.820406 -2.858428 2.165675
C -0.261566 -1.924893 -1.867538
H -1.312139 -2.096196 -1.578939
H -0.224342 -1.833731 -2.955345
H 0.340774 -2.775694 -1.547982
C -4.444055 0.137234 0.293068
C -3.125104 -0.593846 -0.101282
O -3.256884 -1.639643 -0.759747
O -2.099582 -0.029170 0.328175

F -4.385044 1.457232 0.029036
F -4.678408 0.017459 1.618187
F -5.530494 -0.335994 -0.336527
42
F_5 Eopt -1731.162312
C 1.423505 1.087903 0.362408
C 0.883545 1.969409 -0.577096
C 1.073176 3.345924 -0.525668
C 1.849554 3.846077 0.517548
C 2.399960 2.985253 1.471308
C 2.187603 1.608142 1.405645
C 1.042629 -0.351810 0.123300
C 0.282559 -0.385652 -1.177366
H 0.635619 4.005960 -1.267787
H 2.024621 4.914571 0.587893
H 2.998518 3.392531 2.279573
H 2.606222 0.956546 2.165562
S -0.069878 1.114996 -1.795637
O 2.611009 -0.656195 -2.140760
C 3.124136 -1.050989 -1.099123
C 4.557793 -1.292656 -0.969357
C 2.238248 -1.338096 0.108492
C 5.097774 -1.537180 0.244354
H 5.161526 -1.194224 -1.865601
C 2.961702 -1.485395 1.414882
H 1.816034 -2.339377 -0.089278
C 4.296041 -1.614645 1.459373
H 6.173001 -1.666746 0.334240
H 2.365221 -1.565481 2.318947
H 4.801744 -1.783667 2.404259
C -0.035308 -0.862514 1.195496
O -0.005423 -2.021011 1.517117
C -1.001434 0.147898 1.730583
H -0.474846 0.826571 2.410176
H -1.424740 0.749245 0.918185
H -1.794554 -0.374831 2.263925
C -0.298746 -1.608608 -1.752267
H -1.217636 -1.824848 -1.172013
H -0.582003 -1.471674 -2.796621
H 0.381814 -2.457649 -1.651825
C -4.753147 -0.186963 0.260494
C -3.393679 -0.609860 -0.377046
O -2.935112 -1.689619 0.036342
O -2.921086 0.224969 -1.169978
F -5.549393 0.482273 -0.592194
F -4.531609 0.641077 1.310768
F -5.470449 -1.219954 0.731764
42
F_6 Eopt -1731.159117
C 0.924476 -0.713228 0.859715
C -0.162134 -1.472544 0.419081
C -1.155933 -1.947819 1.266437
C -1.051024 -1.621509 2.616728
C 0.022508 -0.858959 3.084508
C 1.017051 -0.407965 2.216514
C 1.903708 -0.362584 -0.234311
C 1.328897 -0.928139 -1.512886
H -1.992304 -2.523809 0.885811
H -1.816370 -1.962356 3.306340
H 0.089108 -0.615743 4.139970
H 1.856132 0.162052 2.601273
S -0.080824 -1.771516 -1.321935
O 0.281055 1.360024 -1.763159
C 0.932043 1.871587 -0.859333
C 0.568471 3.145354 -0.246652
C 2.192652 1.161900 -0.369819
C 1.277345 3.625947 0.797082
H -0.310006 3.649313 -0.636490
C 2.854351 1.766638 0.832463
H 2.920338 1.266739 -1.191077
C 2.437958 2.932867 1.346193
H 0.976277 4.560808 1.262055
H 3.726893 1.260277 1.236691
H 2.958903 3.382739 2.184976
C 3.286073 -1.127247 -0.071793
O 4.283277 -0.613581 -0.514030
C 3.265708 -2.460933 0.610867
H 3.199777 -2.303010 1.693100
H 2.392911 -3.051013 0.315302
H 4.187354 -2.994712 0.378472
C 2.020870 -0.873692 -2.823368
H 2.784256 -1.663450 -2.847659
H 1.326228 -1.044985 -3.646901
H 2.524759 0.084353 -2.964530
C -3.443035 0.774333 -0.264035
C -3.535268 -0.783762 -0.311673
O -4.236730 -1.295224 0.572667
O -2.833447 -1.301361 -1.205169
F -3.374229 1.337539 -1.484043
F -2.325114 1.154447 0.400984
F -4.477615 1.354316 0.369376
42
F_7 Eopt -1731.152518
C 1.228306 -0.395828 0.196108
C 0.832506 -1.410801 -0.678678
C -0.495492 -1.676253 -0.993337
C -1.466759 -0.875232 -0.394771
C -1.097161 0.146718 0.484958
C 0.242099 0.391407 0.788909
C 2.719436 -0.340496 0.426692
C 3.337480 -1.343613 -0.516557
H -0.766493 -2.476091 -1.675137
H -2.523254 -1.041823 -0.597449
H -1.884270 0.743174 0.934811
H 0.504893 1.176912 1.489705
S 2.224793 -2.308368 -1.298600
O 3.445759 0.490084 -2.064577
C 3.318611 1.401674 -1.249711
C 3.163295 2.797609 -1.627862
C 3.354325 1.058593 0.231188
C 2.834551 3.713612 -0.688679
H 3.242005 3.045845 -2.680920
C 2.871043 2.128960 1.160548
H 4.429321 0.958938 0.466881
C 2.668782 3.379989 0.720406
H 2.667583 4.744591 -0.988708
H 2.771648 1.881596 2.214142
H 2.379915 4.167513 1.408084
C 3.114296 -0.881660 1.876933
O 4.113700 -0.450612 2.392853
C 2.212785 -1.898273 2.506104
H 1.325120 -1.389080 2.897580
H 1.866116 -2.638612 1.778855
H 2.742727 -2.384073 3.325489
C 4.784245 -1.663048 -0.572603
H 5.024122 -2.326752 0.269528
H 5.039070 -2.180831 -1.498535
H 5.396229 -0.764779 -0.476308
C -6.497830 0.303118 0.029344
C -4.998421 -0.138385 0.024858
O -4.232404 0.553194 0.715860
O -4.792079 -1.144371 -0.678820
F -7.284731 -0.645290 0.586476
F -6.966244 0.499448 -1.222085
F -6.732116 1.439448 0.707160
42
F_8 Eopt -1731.148038
C -1.195234 0.032070 -0.492868
C -0.197652 -0.590037 0.258470
C 1.135470 -0.201760 0.241304
C 1.475897 0.872710 -0.579374
C 0.500817 1.503600 -1.355483
C -0.831652 1.087136 -1.325841
C -2.561816 -0.595272 -0.333311
C -2.428745 -1.629310 0.761987
H 1.903754 -0.694908 0.828796
H 2.511197 1.206350 -0.598252
H 0.780411 2.332881 -1.997495
H -1.563395 1.582454 -1.953824
S -0.852504 -1.924600 1.217177
O -3.006393 0.145897 2.242295
C -3.481025 0.921184 1.416917
C -3.901454 2.276445 1.738638
C -3.682937 0.425160 -0.010780
C -4.223079 3.132196 0.742568
H -3.864198 2.581194 2.779045
C -3.910624 1.496244 -1.035309
H -4.643194 -0.118921 0.053973
C -4.205441 2.751032 -0.664162
H -4.483927 4.158253 0.987094
H -3.900764 1.216418 -2.085922
H -4.428882 3.507987 -1.408159
C -2.902196 -1.478074 -1.625971
O -1.984040 -1.864925 -2.300106
C -4.338774 -1.794193 -1.934834
H -4.911994 -2.056940 -1.043741
H -4.814585 -0.917172 -2.387059
H -4.360944 -2.615191 -2.652083
C -3.511839 -2.536305 1.219623
H -3.578980 -3.375992 0.512851
H -3.292174 -2.941537 2.208721
H -4.477872 -2.028150 1.238851
C 6.427687 0.002277 0.026456
C 4.959277 0.509062 0.197876
O 4.732053 1.647573 -0.243805
O 4.213948 -0.329983 0.736256
F 6.491998 -0.974160 -0.908969
F 6.919951 -0.528935 1.164803
F 7.294826 0.954583 -0.356964
42
F_9 Eopt -1731.153277
C 1.152223 0.086802 0.401460
C 0.170727 -0.476099 -0.418885
C -1.132413 -0.001773 -0.496058
C -1.455323 1.104139 0.288902
C -0.492735 1.689735 1.115929
C 0.807571 1.186763 1.183976
C 2.477018 -0.634105 0.345097
C 2.350196 -1.679253 -0.735311
H -1.890161 -0.453859 -1.128508
H -2.468198 1.498472 0.242852
H -0.758579 2.550730 1.720870
H 1.533448 1.648263 1.844980

S 0.803970 -1.852973 -1.328401
O 3.137114 0.107596 -2.204586
C 3.613569 0.825788 -1.329499
C 4.132663 2.162029 -1.584621
C 3.698815 0.283452 0.089566
C 4.470691 2.960917 -0.547470
H 4.162050 2.501905 -2.614408
C 3.982106 1.292886 1.159825
H 4.590387 -0.368441 0.081093
C 4.380964 2.534074 0.843765
H 4.809234 3.974176 -0.746128
H 3.934669 0.966225 2.195459
H 4.646425 3.243186 1.620530
C 2.762898 -1.493483 1.661826
O 3.909286 -1.733886 1.942605
C 1.583795 -1.954901 2.459396
H 1.183641 -1.105551 3.024062
H 0.779649 -2.321679 1.814254
H 1.906056 -2.733106 3.151145
C 3.413620 -2.646256 -1.097955
H 3.394992 -3.470941 -0.371822
H 3.243947 -3.064520 -2.091416
H 4.402705 -2.187149 -1.057423
C -6.240360 -0.024592 0.059711
C -4.900543 0.662195 -0.357933
O -4.724685 1.809130 0.084845
O -4.182193 -0.066042 -1.067500
F -6.008111 -1.077485 0.878367
F -6.912590 -0.511501 -1.004387
F -7.090728 0.785161 0.712200
35
G_1-notfa Eopt -
1204.946996
C -0.879227 0.617238 0.302796
C -1.795307 0.455185 -0.741080
C -2.750528 1.426654 -1.024042
C -2.784617 2.575573 -0.233868
C -1.883128 2.745431 0.817578
C -0.929297 1.764884 1.090613
C 0.064096 -0.554925 0.494014
C -0.050580 -1.421354 -0.811569
H -3.451890 1.294682 -1.840871
H -3.521889 3.343331 -0.443183
H -1.922097 3.640841 1.427739
H -0.243467 1.898702 1.921767
S -1.640942 -1.089330 -1.598655
O 1.004813 -0.834570 -1.686564
C 1.819156 -0.104802 -0.993983
C 1.558733 -0.181969 0.453618
C 2.814793 0.705049 -1.550327
C 2.163847 0.896617 1.269564
H 2.108150 -1.089710 0.794033
C 3.402127 1.606147 -0.691619
H 3.030626 0.678906 -2.610739
C 3.074198 1.721944 0.707042
H 1.946379 0.923247 2.332713
H 4.141855 2.291687 -1.094410
H 3.581547 2.475368 1.297054
C -0.264060 -1.392450 1.753075
O 0.649048 -1.834682 2.415824
C -1.710535 -1.611807 2.087060
H -2.113137 -0.693404 2.527784
H -2.297346 -1.822008 1.187503
H -1.797283 -2.426793 2.804373
C 0.249457 -2.901199 -0.690108
H -0.511007 -3.391810 -0.078830
H 0.249588 -3.357233 -1.681825
H 1.229375 -3.059813 -0.231305
42
G_1 Eopt -
1731.184240
C 1.086313 -0.954102 -0.497579
C 2.074594 -1.912365 -0.249960
C 2.289226 -2.982310 -1.113688
C 1.481238 -3.091486 -2.245879
C 0.482056 -2.151075 -2.499062
C 0.279475 -1.080360 -1.626867
C 0.978802 0.129787 0.558834
C 2.292134 0.022037 1.424622
H 3.064906 -3.714732 -0.912958
H 1.633058 -3.919961 -2.930825
H -0.152168 -2.254349 -3.373517
H -0.533231 -0.379324 -1.791497
S 2.959705 -1.649515 1.263921
O 3.233478 0.940617 0.723928
C 2.587871 1.723573 -0.084886
C 1.130408 1.559845 0.006728
C 3.201716 2.608583 -0.974165
C 0.350920 2.097452 -1.130401
H 0.836378 2.235135 0.848311
C 2.376351 3.167875 -1.926709
H 4.273755 2.764677 -0.964017
C 0.967335 2.906813 -2.025078
H -0.717064 1.896010 -1.147052
H 2.822937 3.824152 -2.668975
H 0.408759 3.370682 -2.829762
C -0.267804 0.025312 1.481470
O -0.637189 1.021291 2.063980
C -0.877115 -1.327903 1.668602
H -0.104005 -2.047856 1.964860
H -1.655596 -1.275285 2.430440
H -1.303598 -1.674745 0.720587
C 2.240511 0.480070 2.866652
H 1.865735 1.505391 2.927913
H 1.585547 -0.171102 3.449499
H 3.246120 0.444061 3.293018
C -4.443100 0.218443 0.142146
C -3.121905 -0.290615 -0.514381
O -3.129954 -1.472149 -0.885643
O -2.213057 0.565889 -0.520324
F -5.517046 -0.530440 -0.157676
F -4.328189 0.207166 1.491022
F -4.744452 1.484036 -0.206310
42
G_10 Eopt -
1731.186546
C 2.526127 -0.017028 -0.048365
C 2.713494 0.819199 -1.153282
C 3.862894 0.739480 -1.935436
C 4.841268 -0.191950 -1.586966
C 4.673403 -1.022753 -0.477781
C 3.516005 -0.935156 0.296533
C 1.236150 0.222389 0.714141
C 0.339020 1.127342 -0.204600
H 3.996810 1.386543 -2.796794
H 5.742141 -0.266382 -2.187923
H 5.444781 -1.738493 -0.213660
H 3.394780 -1.572061 1.168721
S 1.399469 1.982846 -1.401952
O -0.454437 0.136712 -0.977399
C -0.368544 -1.039634 -0.439960
C -0.821491 -2.214545 -1.056877
C 0.339944 -1.023803 0.846695
C -0.408244 -3.389335 -0.480863
H -1.396454 -2.178799 -1.973611
C 0.822632 -2.336806 1.327503
H -0.478173 -0.757808 1.559791
C 0.416738 -3.462799 0.700475
H -0.697607 -4.323941 -0.953553
H 1.412422 -2.367796 2.238676
H 0.699094 -4.440550 1.073572
C 1.498784 0.875555 2.090048
O 0.917052 0.456597 3.067251
C 2.505657 1.988106 2.155512
H 3.506892 1.544437 2.205820
H 2.472632 2.622472 1.265193
H 2.332791 2.579498 3.055206
C -0.633982 2.057147 0.487649
H -0.089910 2.847670 1.011107
H -1.295659 2.518287 -0.250203
H -1.244434 1.502928 1.206328
C -4.067486 0.199132 -0.626332
C -3.445196 0.276423 0.803981
O -3.887294 1.212427 1.483281
O -2.579173 -0.587093 1.055240
F -5.410338 0.085686 -0.580835
F -3.617304 -0.832931 -1.360683
F -3.799450 1.318748 -1.333690
42
G_11 Eopt -
1731.179547
C -0.978414 0.899745 -0.524345
C -1.981247 1.858248 -0.693651
C -1.919727 2.809063 -1.707410
C -0.819729 2.791387 -2.565983
C 0.186988 1.838118 -2.410367
C 0.113993 0.889747 -1.387855
C -1.194990 -0.032807 0.656275
C -2.720302 0.101278 1.014311
H -2.708796 3.544503 -1.829681
H -0.753863 3.525504 -3.363121
H 1.039038 1.831473 -3.082676
H 0.916151 0.167478 -1.262966
S -3.290386 1.732564 0.495087
O -3.356232 -0.903529 0.111916
C -2.468755 -1.735037 -0.336450
C -2.727071 -2.712295 -1.299327
C -1.134649 -1.523205 0.261046
C -1.618742 -3.329370 -1.840252
H -3.732699 -2.893444 -1.659344
C -0.005281 -2.132108 -0.482400
H -1.185920 -2.129226 1.196079
C -0.265467 -3.031782 -1.460176
H -1.773768 -4.059460 -2.630210
H 1.005757 -1.883163 -0.169127
H 0.543798 -3.532199 -1.979510
C -0.331867 0.397805 1.875624
O -0.155367 1.582540 2.046190
C 0.165295 -0.647696 2.833871
H -0.673351 -1.162142 3.315654
H 0.772525 -1.387024 2.307022
H 0.774650 -0.158876 3.594499
C -3.171105 -0.244584 2.418600
H -2.715712 0.434040 3.143454
H -4.258012 -0.153291 2.485444
H -2.895782 -1.275535 2.661966
C 4.504432 0.170642 0.227406
C 3.084658 -0.388731 -0.091345

O 2.996004 -0.910125 -1.217701
O 2.229788 -0.245564 0.802220
F 5.439876 -0.801581 0.162332
F 4.609333 0.725223 1.444575
F 4.869735 1.115609 -0.664163
42
G_12 Eopt -
1731.180123
C 0.638781 -0.833056 -0.494282
C 1.392708 -1.991621 -0.700396
C 1.041831 -2.922198 -1.673985
C -0.097642 -2.680789 -2.443510
C -0.862408 -1.530560 -2.244049
C -0.493349 -0.601979 -1.270529
C 1.129927 0.046582 0.638499
C 2.585695 -0.444799 0.971927
H 1.636830 -3.816518 -1.830546
H -0.388724 -3.400436 -3.202242
H -1.759011 -1.350675 -2.828355
H -1.110357 0.278945 -1.120288
S 2.766802 -2.155066 0.410419
O 3.431654 0.402189 0.083870
C 2.751174 1.409424 -0.370849
C 3.215622 2.294095 -1.346940
C 1.409667 1.501872 0.223406
C 2.267911 3.133190 -1.892766
H 4.234378 2.245688 -1.712024
C 0.441038 2.349220 -0.506623
H 1.569937 2.066366 1.173216
C 0.885593 3.157621 -1.496369
H 2.574661 3.799396 -2.694529
H -0.587945 2.375672 -0.159636
H 0.210147 3.835260 -2.005383
C 0.224814 -0.003938 1.896118
O 0.106211 0.995817 2.570984
C -0.439181 -1.306481 2.221670
H -1.197943 -1.528066 1.463229
H 0.288763 -2.125446 2.194598
H -0.907306 -1.243283 3.203912
C 3.088448 -0.241021 2.384954
H 2.512922 -0.855341 3.081833
H 4.140373 -0.530746 2.446041
H 2.996621 0.810504 2.673454
C -3.656239 0.434837 0.640580
C -3.720574 -0.107260 -0.826207
O -3.368150 0.743860 -1.665292
O -4.099692 -1.281134 -0.945061
F -2.415228 0.889902 0.945271
F -3.965215 -0.473620 1.579699
F -4.493474 1.478724 0.822820
42
G_13 Eopt -
1731.174509
C 2.702934 0.854125 -0.411472
C 3.006186 1.589259 0.737480
C 3.181977 2.968934 0.693620
C 3.059068 3.615067 -0.536776
C 2.768425 2.892491 -1.694260
C 2.593030 1.509005 -1.634968
C 2.586776 -0.643315 -0.184934
C 2.401562 -0.817609 1.365121
H 3.407287 3.530860 1.594589
H 3.190577 4.691283 -0.588552
H 2.678173 3.404743 -2.646401
H 2.385426 0.952415 -2.544206
S 3.164988 0.593169 2.194729
O 0.929007 -0.685814 1.531977
C 0.323428 -0.840328 0.393574
C -1.042416 -0.642929 0.195708
C 1.252735 -1.233025 -0.686425
C -1.461815 -0.612219 -1.117048
H -1.731505 -0.436584 1.006884
C 0.731892 -1.051032 -2.063297
H 1.304675 -2.338359 -0.565284
C -0.581793 -0.793295 -2.245512
H -2.518706 -0.410014 -1.303942
H 1.405170 -1.228200 -2.897527
H -1.000174 -0.720087 -3.242985
C 3.870401 -1.366903 -0.667544
O 4.928745 -0.790672 -0.550553
C 3.761034 -2.748876 -1.245451
H 3.267994 -3.430434 -0.543801
H 3.158121 -2.727755 -2.160037
H 4.760301 -3.118727 -1.475365
C 2.798243 -2.139753 1.988176
H 3.872882 -2.305470 1.880089
H 2.550122 -2.131677 3.052374
H 2.254967 -2.962966 1.514308
C -6.280546 0.235691 0.306520
C -4.813935 0.003126 -0.174698
O -4.640608 0.010307 -1.405651
O -4.014167 -0.174056 0.762734
F -6.358832 1.239865 1.204603
F -6.768394 -0.865541 0.920423
F -7.138556 0.535861 -0.681775
42
G_14 Eopt -
1731.180411
C 2.680106 0.842609 -0.397435
C 2.935204 1.565403 0.772076
C 3.101811 2.947147 0.749313
C 3.018572 3.606390 -0.477241
C 2.776709 2.895912 -1.653932
C 2.610072 1.511050 -1.617519
C 2.562319 -0.657396 -0.197927
C 2.383648 -0.874854 1.348000
H 3.289311 3.500739 1.663960
H 3.142766 4.684146 -0.511201
H 2.717153 3.419599 -2.602174
H 2.438538 0.961061 -2.538657
S 3.065149 0.555980 2.224419
O 0.910390 -0.821942 1.520241
C 0.307479 -0.957233 0.374867
C -1.064141 -0.803783 0.183698
C 1.246157 -1.263989 -0.716250
C -1.483109 -0.736944 -1.129139
H -1.761787 -0.654453 1.000040
C 0.725335 -1.056604 -2.086339
H 1.370170 -2.370374 -0.657082
C -0.598103 -0.843267 -2.261964
H -2.546499 -0.566007 -1.309909
H 1.406318 -1.189467 -2.921960
H -1.019945 -0.754852 -3.256739
C 3.786410 -1.416951 -0.759059
O 3.612571 -2.452461 -1.365734
C 5.143059 -0.815577 -0.540392
H 5.289938 -0.011943 -1.271423
H 5.231144 -0.371002 0.455432
H 5.906729 -1.579490 -0.688811
C 2.854977 -2.194108 1.921673
H 3.941300 -2.280233 1.837016
H 2.578034 -2.256613 2.976779
H 2.386993 -3.028455 1.390110
C -6.252402 0.317733 0.306396
C -4.825486 -0.095352 -0.173988
O -4.657051 -0.109619 -1.405704
O -4.052655 -0.377421 0.760138
F -6.209625 1.242234 1.287200
F -6.914953 -0.747677 0.812656
F -7.027675 0.824564 -0.666148
42
G_15 Eopt -
1731.180097
C -2.294576 1.022533 0.236300
C -3.163829 1.490043 -0.753892
C -3.195528 2.833585 -1.116454
C -2.339407 3.718693 -0.460728
C -1.474017 3.267225 0.536866
C -1.451491 1.917761 0.890798
C -2.418577 -0.460182 0.534017
C -3.244105 -1.077823 -0.651654
H -3.868098 3.185625 -1.892312
H -2.349265 4.769096 -0.733963
H -0.814210 3.965418 1.041005
H -0.786903 1.575899 1.679145
S -4.216182 0.234814 -1.434095
O -2.192368 -1.471978 -1.620996
C -1.021623 -1.477635 -1.051915
C 0.180482 -1.645999 -1.735012
C -1.100523 -1.244478 0.398820
C 1.323775 -1.364515 -1.013343
H 0.199296 -1.884410 -2.791694
C 0.168801 -0.852057 1.052974
H -1.296822 -2.256114 0.825126
C 1.329946 -0.954268 0.365497
H 2.294949 -1.415122 -1.500278
H 0.145867 -0.593340 2.107647
H 2.292230 -0.735029 0.823776
C -3.094937 -0.726424 1.898149
O -2.650827 -1.593278 2.620261
C -4.272818 0.124432 2.271264
H -3.902294 1.087795 2.640504
H -4.913182 0.329623 1.408240
H -4.839059 -0.367837 3.062308
C -4.063276 -2.315192 -0.351692
H -4.870262 -2.076401 0.345401
H -4.499612 -2.701705 -1.275793
H -3.429105 -3.092247 0.087055
C 6.256084 0.333998 -0.005883
C 4.822869 -0.249659 0.204000
O 4.371324 -0.172962 1.358346
O 4.330380 -0.715756 -0.841158
F 6.256563 1.318844 -0.930754
F 7.112063 -0.610488 -0.453039
F 6.802909 0.852009 1.105910
35
G_2-notfa Eopt -
1204.967293
C -0.915060 0.544573 0.382518
C -1.895092 0.378525 -0.599077
C -2.912016 1.311990 -0.776299
C -2.941831 2.427984 0.060630
C -1.975044 2.601749 1.051596
C -0.960293 1.658077 1.217237
C 0.093500 -0.588265 0.459618
C -0.046942 -1.370961 -0.894363
H -3.664226 1.177680 -1.547379
H -3.727576 3.166288 -0.064981

H -2.010832 3.471254 1.699585
H -0.222302 1.790200 2.003312
S -1.714630 -1.116950 -1.534553
O 0.890712 -0.632852 -1.793822
C 1.700210 0.102995 -1.102889
C 1.552973 -0.101083 0.352650
C 2.602966 1.013874 -1.660027
C 2.131885 0.968486 1.201083
H 2.221491 -0.971715 0.543138
C 3.192991 1.888567 -0.776212
H 2.747247 1.077713 -2.731979
C 2.949698 1.889001 0.643824
H 1.963430 0.923677 2.273235
H 3.862502 2.646491 -1.173865
H 3.438474 2.640994 1.252276
C -0.225265 -1.524897 1.655875
O -1.388185 -1.708968 1.935677
C 0.902220 -2.172965 2.407547
H 1.545923 -2.753634 1.738650
H 1.523287 -1.405096 2.882977
H 0.485887 -2.827920 3.172935
C 0.378097 -2.824106 -0.925549
H -0.260187 -3.420854 -0.269693
H 0.292939 -3.210003 -1.944396
H 1.419834 -2.920572 -0.604794
42
G_2 Eopt -
1731.183924
C 0.578610 -0.847793 -0.499419
C 1.275557 -2.049889 -0.656460
C 0.908715 -2.988828 -1.616093
C -0.186378 -2.708139 -2.433478
C -0.890458 -1.511648 -2.290880
C -0.511814 -0.578331 -1.324514
C 1.100056 0.042856 0.613282
C 2.533754 -0.510044 0.966984
H 1.462157 -3.915889 -1.728413
H -0.487758 -3.429058 -3.187020
H -1.744305 -1.304171 -2.927972
H -1.083651 0.334466 -1.187314
S 2.617104 -2.250182 0.484583
O 3.420158 0.252774 0.043474
C 2.800255 1.291545 -0.427084
C 1.460674 1.466907 0.152203
C 3.325366 2.140606 -1.403521
C 0.545792 2.352261 -0.601956
H 1.643132 2.046116 1.090744
C 2.433564 3.030040 -1.965397
H 4.344542 2.033525 -1.754811
C 1.050673 3.129265 -1.590397
H -0.486582 2.413658 -0.266668
H 2.789285 3.675220 -2.764349
H 0.420112 3.837754 -2.115026
C 0.226894 0.079693 1.899677
O 0.351840 1.022207 2.649487
C -0.623945 -1.115340 2.195987
H -1.352222 -1.271336 1.395498
H 0.010847 -2.010176 2.236476
H -1.135702 -0.973922 3.147385
C 3.075147 -0.282126 2.363001
H 3.048364 0.781337 2.615730
H 2.485374 -0.835567 3.096723
H 4.110716 -0.628714 2.407600
C -3.920278 0.153785 -0.211981
C -3.126244 0.795852 0.972067
O -3.670898 0.652350 2.074052
O -2.039392 1.316830 0.647090
F -5.248435 0.344722 -0.116077
F -3.552361 0.613838 -1.423071
F -3.724456 -1.186078 -0.236058
42
G_3 Eopt -
1731.183316
C 0.777981 -0.909350 -0.521797
C 1.554550 -2.068946 -0.435006
C 1.437384 -3.098729 -1.364125
C 0.512053 -2.955632 -2.398347
C -0.276138 -1.807876 -2.492793
C -0.148396 -0.781943 -1.555200
C 1.018644 0.096717 0.588389
C 2.368331 -0.337539 1.278613
H 2.050544 -3.991118 -1.286986
H 0.406558 -3.748503 -3.132396
H -1.001955 -1.711937 -3.293983
H -0.800017 0.085603 -1.595199
S 2.636545 -2.099070 0.969585
O 3.396478 0.406471 0.499935
C 2.845016 1.358618 -0.189037
C 1.407291 1.499579 0.083194
C 3.526952 2.146634 -1.117906
C 0.635224 2.263345 -0.922208
H 1.363931 2.164468 0.980296
C 2.736549 2.926432 -1.936286
H 4.600288 2.068898 -1.242270
C 1.303491 2.977787 -1.859763
H -0.445560 2.298446 -0.802523
H 3.224339 3.516856 -2.707356
H 0.768607 3.601363 -2.566775
C -0.110808 0.179983 1.652668
O -0.203724 1.187622 2.317644
C -0.956424 -1.036952 1.861266
H -1.503186 -1.265471 0.942211
H -0.315571 -1.899588 2.083901
H -1.651225 -0.862609 2.682955
C 2.582662 0.029277 2.732167
H 3.598111 -0.246160 3.027565
H 2.452772 1.105675 2.877238
H 1.871636 -0.501813 3.368439
C -4.055735 -0.139495 -0.042993
C -3.313577 1.218998 0.175638
O -4.037492 2.107052 0.644794
O -2.104409 1.212826 -0.132362
F -5.231720 0.020155 -0.681190
F -3.349892 -1.033678 -0.759455
F -4.338510 -0.731610 1.139821
42
G_4 Eopt -
1731.181366
C 1.086511 0.958339 -0.427565
C 1.228996 1.768294 0.701976
C 0.595558 3.004148 0.794437
C -0.178964 3.434223 -0.282910
C -0.310553 2.645700 -1.426861
C 0.322598 1.405445 -1.502591
C 1.850906 -0.349689 -0.366765
C 2.323861 -0.521378 1.125041
H 0.697967 3.618055 1.683632
H -0.687607 4.391313 -0.223764
H -0.918736 2.990603 -2.256647
H 0.215652 0.794525 -2.394675
S 2.315957 1.098451 1.932836
O 1.234765 -1.318591 1.740031
C 0.477001 -1.850573 0.826890
C 0.984306 -1.611396 -0.534006
C -0.683198 -2.571612 1.094270
C -0.007519 -1.807864 -1.617375
H 1.702962 -2.449008 -0.695464
C -1.486492 -2.843489 0.005179
H -0.976354 -2.811101 2.109302
C -1.170973 -2.436819 -1.340982
H 0.272033 -1.541118 -2.632095
H -2.419037 -3.370849 0.165925
H -1.890498 -2.648573 -2.123141
C 3.054118 -0.365566 -1.338565
O 3.310468 -1.379039 -1.953337
C 3.852326 0.897287 -1.478242
H 3.960108 1.413049 -0.519137
H 4.829408 0.663202 -1.901672
H 3.319316 1.575087 -2.154920
C 3.607995 -1.289233 1.361380
H 3.764435 -1.420485 2.434924
H 3.547835 -2.277767 0.895025
H 4.462950 -0.749690 0.946581
C -3.034688 0.618017 -0.092037
C -4.135958 -0.377691 0.395752
O -3.917040 -1.550408 0.030764
O -5.044097 0.116994 1.077270
F -1.854275 0.368209 0.522677
F -2.809195 0.506804 -1.418721
F -3.321362 1.909323 0.140573
42
G_5 Eopt -
1731.184511
C 0.774197 -0.837090 -0.549085
C 1.724565 -1.782669 -0.948120
C 1.480419 -2.669414 -1.992505
C 0.251886 -2.598839 -2.650089
C -0.700332 -1.650512 -2.275219
C -0.440491 -0.767231 -1.226934
C 1.198242 0.026293 0.625173
C 2.760352 -0.140799 0.728887
H 2.229234 -3.394920 -2.294326
H 0.044167 -3.284053 -3.465955
H -1.651077 -1.597041 -2.795893
H -1.186273 -0.038448 -0.930411
S 3.220376 -1.731216 0.002634
O 3.265378 0.930029 -0.177667
C 2.330394 1.800009 -0.414251
C 1.102567 1.536853 0.349656
C 2.450160 2.854885 -1.321076
C -0.120306 2.211205 -0.138734
H 1.280372 2.033317 1.335030
C 1.278978 3.516265 -1.628014
H 3.389824 3.070104 -1.815724
C -0.002208 3.193918 -1.063914
H -1.063974 1.945587 0.334881
H 1.320079 4.312377 -2.366952
H -0.870819 3.751308 -1.395368
C 0.544920 -0.335761 1.988732
O 0.494412 0.521659 2.843377
C 0.102828 -1.748791 2.191331
H 0.868109 -2.450519 1.840834
H -0.110613 -1.913014 3.248192
H -0.811921 -1.914011 1.608043
C 3.420228 0.084166 2.073180
H 4.505783 0.043536 1.953910
H 3.144998 1.063179 2.475831
H 3.112991 -0.688650 2.781014

C -4.108854 0.200350 0.005679
C -2.990773 -0.495220 0.846702
O -2.123093 0.309202 1.248046
O -3.094325 -1.720963 0.983463
F -4.764417 1.136848 0.722837
F -3.577285 0.842297 -1.062726
F -5.036799 -0.639634 -0.475821
42
G_6 Eopt -
1731.179696
C 1.167799 -1.002839 -0.167661
C 2.193179 -1.756727 -0.749385
C 1.915633 -2.888232 -1.510365
C 0.583275 -3.270061 -1.674256
C -0.446168 -2.533278 -1.088593
C -0.158952 -1.397139 -0.329068
C 1.659563 0.173701 0.668063
C 3.155832 0.393784 0.265245
H 2.716381 -3.461252 -1.967419
H 0.351190 -4.150923 -2.264763
H -1.478455 -2.842140 -1.216337
H -0.969953 -0.846287 0.144270
S 3.821329 -1.148323 -0.402648
O 3.048879 1.344459 -0.880254
C 1.877318 1.894785 -0.914390
C 1.035608 1.525965 0.242321
C 1.442959 2.725334 -1.949606
C -0.418539 1.767334 0.054524
H 1.325371 2.280624 1.011374
C 0.094141 3.006175 -1.968072
H 2.118781 3.036462 -2.737189
C -0.839093 2.516915 -0.991194
H -1.125932 1.419392 0.804083
H -0.295025 3.604231 -2.787846
H -1.893715 2.746010 -1.093342
C 1.493855 -0.213874 2.156597
O 2.337465 -0.916447 2.671088
C 0.259840 0.238282 2.875626
H 0.263288 -0.174268 3.884833
H 0.229518 1.332806 2.925993
H -0.642664 -0.087132 2.343058
C 4.081621 1.052273 1.265826
H 5.019691 1.313235 0.770003
H 3.624731 1.968923 1.652506
H 4.287093 0.375516 2.094008
C -4.602005 -0.391877 0.002442
C -3.521249 0.540831 0.630613
O -3.568974 1.732149 0.289500
O -2.718113 -0.065839 1.369387
F -5.600080 0.267199 -0.606769
F -4.041374 -1.195036 -0.932588
F -5.171136 -1.203798 0.913731
42
G_7 Eopt -
1731.177134
C 1.107666 -0.849473 -0.662162
C 2.122940 -1.808450 -0.639243
C 2.319398 -2.682236 -1.704401
C 1.461115 -2.593168 -2.801270
C 0.427433 -1.655941 -2.823986
C 0.242674 -0.781326 -1.751401
C 1.011559 -0.006228 0.595807
C 2.361684 -0.227984 1.366237
H 3.116316 -3.418947 -1.681798
H 1.598477 -3.268485 -3.640078
H -0.245475 -1.609374 -3.674038
H -0.592279 -0.082988 -1.748632
S 3.056945 -1.818795 0.868569
O 3.247696 0.829361 0.800006
C 2.549937 1.718404 0.167037
C 3.104526 2.751240 -0.591590
C 1.095546 1.510085 0.311941
C 2.224135 3.444196 -1.394549
H 4.174162 2.921787 -0.610828
C 0.254874 2.215237 -0.686471
H 0.869179 2.051785 1.258780
C 0.815038 3.171808 -1.464116
H 2.622667 4.222130 -2.040385
H -0.811681 1.999258 -0.706874
H 0.209297 3.743990 -2.157307
C -0.178400 -0.490935 1.470732
O -0.364856 -1.684460 1.546322
C -1.013328 0.517301 2.199952
H -0.387278 1.167767 2.820728
H -1.543336 1.136587 1.465462
H -1.737160 -0.004931 2.825520
C 2.359193 -0.028976 2.867431
H 1.725367 -0.776416 3.349608
H 3.376708 -0.131416 3.252518
H 1.993258 0.971905 3.117879
C -4.186413 -0.191387 0.411269
C -3.664365 0.451623 -0.911649
O -4.452249 0.461124 -1.863350
O -2.495068 0.882394 -0.800873
F -4.270146 0.732700 1.397874
F -3.354939 -1.152875 0.858909
F -5.403226 -0.747828 0.305227
42
G_8 Eopt -
1731.175862
C -1.267890 1.014742 0.279208
C -1.854580 1.817385 -0.702355
C -1.325355 3.059406 -1.038623
C -0.185055 3.499294 -0.365383
C 0.408003 2.711015 0.620701
C -0.134194 1.467458 0.948235
C -1.998372 -0.291775 0.539023
C -2.921961 -0.511980 -0.711159
H -1.783412 3.669812 -1.810489
H 0.245674 4.462518 -0.620111
H 1.298490 3.059304 1.133491
H 0.330299 0.865866 1.723879
S -3.307335 1.102774 -1.425675
O -2.020405 -1.214339 -1.660860
C -0.975747 -1.680726 -1.041773
C 0.128725 -2.233407 -1.680418
C -1.079661 -1.526479 0.425855
C 1.247140 -2.420555 -0.892490
H 0.140040 -2.385968 -2.752901
C 0.200383 -1.666831 1.163630
H -1.669773 -2.419293 0.729551
C 1.290004 -2.136357 0.520860
H 2.164299 -2.777765 -1.349898
H 0.203979 -1.473600 2.232542
H 2.226981 -2.297546 1.041873
C -2.853964 -0.195241 1.827714
O -3.373510 0.866564 2.089716
C -3.017066 -1.410394 2.695490
H -3.373069 -2.270748 2.119327
H -2.048556 -1.683562 3.131028
H -3.722040 -1.183882 3.495437
C -4.152752 -1.378264 -0.544695
H -4.854976 -0.913709 0.151707
H -4.647172 -1.500829 -1.511560
H -3.876221 -2.369125 -0.171817
C 4.301261 0.485489 0.298073
C 3.790361 -0.633595 -0.663336
O 4.271431 -1.757670 -0.433091
O 2.928276 -0.264643 -1.479494
F 3.595714 0.458094 1.456979
F 4.169508 1.726694 -0.198463
F 5.594866 0.339631 0.637843
42
G_9 Eopt -
1731.181981
C 1.260501 -0.999595 0.235308
C 1.807681 -1.716609 -0.833097
C 1.283567 -2.943269 -1.229625
C 0.190540 -3.455335 -0.530173
C -0.363003 -2.753052 0.541012
C 0.171959 -1.524112 0.928224
C 1.973381 0.302563 0.550766
C 2.876695 0.615722 -0.696232
H 1.709918 -3.486010 -2.067385
H -0.235490 -4.407219 -0.831066
H -1.217415 -3.158142 1.072894
H -0.261400 -0.985896 1.766147
S 3.215406 -0.932342 -1.574325
O 1.979856 1.417657 -1.562226
C 0.941453 1.832063 -0.891983
C -0.164718 2.447659 -1.464614
C 1.053970 1.537440 0.547044
C -1.275614 2.560210 -0.650537
H -0.187903 2.699873 -2.517795
C -0.210838 1.615567 1.314084
H 1.660194 2.379119 0.952165
C -1.305890 2.148070 0.730333
H -2.196114 2.958240 -1.065402
H -0.197607 1.330889 2.362081
H -2.235737 2.266672 1.274847
C 2.807949 0.228215 1.850816
O 2.828106 1.184295 2.596746
C 3.552992 -1.042620 2.132881
H 2.843481 -1.783428 2.519715
H 3.995187 -1.460444 1.223372
H 4.320971 -0.854238 2.883445
C 4.126792 1.434406 -0.455494
H 4.829592 0.875520 0.167241
H 4.607063 1.665395 -1.409473
H 3.875471 2.373917 0.046264
C -4.241947 -0.565149 0.223948
C -3.756941 0.669937 -0.599293
O -4.267456 1.747965 -0.246343
O -2.882965 0.416743 -1.446598
F -3.515416 -0.675459 1.364864
F -4.108710 -1.733250 -0.426976
F -5.530119 -0.478260 0.602034
34
H_1-notfa Eopt -
1204.588165
C 1.341136 -0.249077 0.355781
C 1.885195 -0.167513 -0.929879
C 3.109311 -0.760141 -1.235132
C 3.785326 -1.454214 -0.231486
C 3.249081 -1.547187 1.054169
C 2.025696 -0.943438 1.349826
C -0.024396 0.377907 0.491031
C -0.243430 1.359602 -0.727508

H 3.528720 -0.683844 -2.233579
H 4.739978 -1.920280 -0.456034
H 3.785996 -2.084290 1.829498
H 1.613321 -1.006233 2.352518
S 0.877815 0.717393 -2.078678
O -1.594977 1.190956 -1.142180
C -2.012313 -0.044397 -0.717973
C -1.157891 -0.598743 0.229101
C -3.169128 -0.676561 -1.144800
C -1.444414 -1.837393 0.784853
C -3.454745 -1.921165 -0.574404
H -3.815303 -0.223625 -1.889212
C -2.606602 -2.499190 0.374630
H -0.779328 -2.282829 1.520117
H -4.350667 -2.450432 -0.884218
H -2.849694 -3.470296 0.793349
C -0.222621 1.152977 1.815928
O 0.736816 1.523084 2.458728
C -1.634544 1.448885 2.246373
H -2.068894 0.542963 2.684448
H -1.624846 2.242847 2.994040
H -2.264808 1.730995 1.397444
C 0.017398 2.830569 -0.487577
H -0.133437 3.386379 -1.416162
H -0.666863 3.225423 0.270049
H 1.047720 2.977076 -0.153389
42
H_1 Eopt -
1731.250573
C 0.246093 -0.551541 -0.383347
C 0.016830 -1.527450 0.591805
C -0.959858 -2.504393 0.413572
C -1.698464 -2.504157 -0.770980
C -1.462332 -1.547220 -1.759192
C -0.486960 -0.566698 -1.566021
C 1.352412 0.417628 -0.045035
C 1.653802 0.312364 1.508589
H -1.148022 -3.248046 1.181727
H -2.468336 -3.255744 -0.918070
H -2.047179 -1.552538 -2.673472
H -0.319070 0.193184 -2.325018
S 1.070744 -1.392695 2.000849
O 3.067965 0.373561 1.643439
C 3.625708 0.001569 0.447572
C 2.703970 -0.001334 -0.592772
C 4.963379 -0.310942 0.264097
C 3.104217 -0.336707 -1.878549
H -1.532834 1.444096 0.102300
C 5.361510 -0.636272 -1.035976
H 5.660541 -0.306298 1.095192
C 4.448813 -0.653355 -2.095200
H 2.388666 -0.353084 -2.696564
H 6.400854 -0.889299 -1.221489
H 4.785318 -0.919735 -3.091761
C 0.992439 1.876548 -0.379653
O -0.174672 2.240491 -0.359253
C 2.097434 2.841270 -0.668511
H 2.528207 2.609020 -1.649081
H 1.707792 3.859064 -0.665194
H 2.903274 2.733539 0.065849
C 1.036274 1.354066 2.416538
H 1.315441 1.141366 3.451274
H 1.394637 2.355953 2.158963
H -0.053599 1.326353 2.339427
C -4.509927 0.137439 0.089092
C -3.266600 0.870514 -0.461024
O -3.236377 1.267212 -1.597706
O -2.358384 1.009201 0.470768
F -5.301389 1.011040 0.732701
F -5.214620 -0.402460 -0.904883
F -4.189357 -0.831513 0.950440
34
H_2-notfa Eopt -
1204.586747
C 1.272222 -0.348092 0.278008
C 1.777828 -0.273482 -1.024136
C 2.952561 -0.935494 -1.376752
C 3.614881 -1.689194 -0.406671
C 3.113578 -1.777797 0.893115
C 1.937931 -1.107330 1.236520
C -0.037396 0.373196 0.482910
C -0.256844 1.371125 -0.726455
H 3.344807 -0.868668 -2.386873
H 4.530890 -2.209060 -0.670440
H 3.637182 -2.366377 1.639522
H 1.543782 -1.175992 2.247886
S 0.784798 0.687451 -2.122331
O -1.628778 1.266567 -1.083836
C -2.102007 0.067206 -0.619653
C -1.240499 -0.527511 0.292261
C -3.311691 -0.499507 -0.990031
C -1.573794 -1.741909 0.873866
C -3.643538 -1.720272 -0.395323
H -3.962595 -0.014783 -1.709890
C -2.788794 -2.338501 0.522313
H -0.904692 -2.219124 1.585030
H -4.581523 -2.198949 -0.659831
H -3.069489 -3.289529 0.963155
C -0.117292 1.144751 1.821993
O -1.169131 1.211011 2.422466
C 1.124862 1.824439 2.344223
H 0.845695 2.795734 2.759023
H 1.530513 1.213018 3.158210
H 1.901371 1.938723 1.587095
C 0.052248 2.838510 -0.514171
H -0.553923 3.239359 0.304912
H 1.111553 2.985784 -0.295499
H -0.192606 3.389190 -1.426132
42
H_2 Eopt -
1731.247716
C -1.816300 1.349687 -0.380450
C -2.582257 1.847802 0.677883
C -3.243257 3.070530 0.574477
C -3.136247 3.790183 -0.615748
C -2.376210 3.299977 -1.679422
C -1.713560 2.077229 -1.562692
C -1.240395 -0.022010 -0.128936
C -1.246385 -0.284320 1.433636
H -3.829153 3.456262 1.403094
H -3.646013 4.744291 -0.708679
H -2.294162 3.871839 -2.598047
H -1.113786 1.699793 -2.386127
S -2.628901 0.788811 2.089774
O -1.607156 -1.649159 1.603718
C -2.298139 -2.043355 0.486389
C -2.152441 -1.152994 -0.571514
C -3.041837 -3.208227 0.387015
C -2.772540 -1.404299 -1.786911
H 2.429509 0.721256 -0.964497
C -3.655018 -3.460614 -0.844087
H -3.143307 -3.884285 1.229257
C -3.529401 -2.573145 -1.917168
H -2.672731 -0.708394 -2.615826
H -4.249026 -4.361650 -0.962123
H -4.026286 -2.791192 -2.856809
C 0.194899 -0.193791 -0.645132
O 0.892390 0.801770 -0.793949
C 0.701998 -1.577634 -0.899481
H 0.163183 -2.000799 -1.754194
H 1.771246 -1.567259 -1.109143
H 0.492992 -2.222325 -0.037939
C 0.036185 -0.012688 2.187839
H 0.843861 -0.659483 1.831356
H 0.332251 1.031644 2.056946
H -0.120061 -0.202595 3.252453
C 5.452952 -0.030543 -0.021348
C 3.909922 0.007297 0.013746
O 3.285103 -0.540577 0.887601
O 3.445815 0.687078 -1.002035
F 5.958128 1.206606 0.073130
F 5.935580 -0.756638 0.983726
F 5.888834 -0.562800 -1.171008
42
H_3 Eopt -
1731.250142
C 2.525582 -0.745631 -0.139435
C 3.517627 0.097896 -0.648811
C 4.626644 -0.421184 -1.314397
C 4.724171 -1.803578 -1.477224
C 3.732046 -2.652842 -0.983843
C 2.627058 -2.123228 -0.315444
C 1.361199 -0.023797 0.494832
C 1.791547 1.475821 0.786088
H 5.398784 0.236222 -1.701853
H 5.584015 -2.218834 -1.993871
H 3.817824 -3.726111 -1.118814
H 1.849995 -2.782004 0.065766
S 3.198719 1.815368 -0.395502
O 0.678689 2.281627 0.422507
C -0.098312 1.572627 -0.455642
C 0.224971 0.220782 -0.478221
C -1.112566 2.114008 -1.228480
C -0.470862 -0.650125 -1.303812
H -1.682967 -0.147229 1.407513
C -1.818653 1.228303 -2.048066
H -1.343491 3.173177 -1.192983
C -1.504003 -0.133385 -2.092872
H -0.224416 -1.708091 -1.333263
H -2.623970 1.610817 -2.668183
H -2.065454 -0.795490 -2.744481
C 0.874406 -0.670834 1.802492
O -0.308285 -0.637697 2.109203
C 1.865000 -1.319603 2.723734
H 1.585273 -1.098465 3.755979
H 1.792805 -2.404005 2.578239
H 2.893225 -1.018842 2.523698
C 2.189656 1.848475 2.199495
H 1.370596 1.640363 2.896306
H 3.084258 1.304088 2.507570
H 2.406770 2.919055 2.235594
C -4.582049 -0.297832 -0.079591
C -3.239274 -0.788863 0.506494
O -2.950259 -1.957200 0.433063
O -2.571574 0.178680 1.074879
F -5.585570 -0.673196 0.729777
F -4.643116 1.026751 -0.220090
F -4.795363 -0.855321 -1.275264

42
H_4 Eopt -
1731.248134
C -1.526695 1.322856 -0.361557
C -2.765502 1.868271 -0.013356
C -3.203876 3.070754 -0.565563
C -2.384763 3.720119 -1.489089
C -1.148562 3.179889 -1.848987
C -0.716938 1.978890 -1.284794
C -1.246223 -0.020838 0.265866
C -2.218178 -0.210568 1.501889
H -4.163109 3.494005 -0.283743
H -2.714395 4.657690 -1.926282
H -0.517467 3.695696 -2.565469
H 0.247597 1.561474 -1.560345
S -3.682165 0.890436 1.135948
O -2.646680 -1.566573 1.467216
C -2.500446 -2.022997 0.182123
C -1.701381 -1.189089 -0.591568
C -3.047645 -3.198474 -0.306506
C -1.421483 -1.514451 -1.910948
H 2.421987 0.718702 1.098461
C -2.756830 -3.523553 -1.635093
H -3.673198 -3.828811 0.316374
C -1.958137 -2.696500 -2.430824
H -0.800703 -0.865451 -2.523282
H -3.168780 -4.435127 -2.056936
H -1.756097 -2.972114 -3.460617
C 0.206239 -0.186780 0.733161
O 0.876912 0.812999 0.957243
C 0.743092 -1.569588 0.929638
H 1.650932 -1.545305 1.534312
H -0.005911 -2.212753 1.402720
H 0.968370 -2.000986 -0.052281
C -1.662659 0.094495 2.876065
H -0.832370 -0.577568 3.117033
H -1.314793 1.129832 2.917921
H -2.446371 -0.042450 3.625047
C 5.358166 -0.040685 -0.074567
C 3.818046 0.047202 -0.015839
O 3.127015 -0.402120 -0.895770
O 3.436270 0.655534 1.077361
F 5.843788 -0.651121 1.014104
F 5.756624 -0.720390 -1.146935
F 5.896639 1.185263 -0.130147
42
H_5 Eopt -
1731.250032
C -2.721380 0.199530 0.463268
C -3.476441 0.238533 -0.712759
C -4.767977 0.762807 -0.716940
C -5.292679 1.262495 0.475591
C -4.542541 1.236655 1.652667
C -3.251558 0.705658 1.647119
C -1.323023 -0.346869 0.291346
C -1.242104 -1.104686 -1.101489
H -5.355292 0.783582 -1.629784
H -6.297922 1.672561 0.483377
H -4.961759 1.627494 2.574030
H -2.664009 0.683927 2.562256
S -2.634299 -0.395701 -2.127179
O -0.005378 -0.726149 -1.688756
C 0.418398 0.431823 -1.099184
C -0.290023 0.736596 0.055609
C 1.457898 1.221125 -1.570586
C 0.020190 1.877709 0.784431
H 1.609454 -0.521286 1.524732
C 1.773427 2.359137 -0.823285
H 1.995546 0.958370 -2.475731
C 1.063322 2.691265 0.335366
H -0.535394 2.129510 1.684013
H 2.583613 3.000787 -1.156837
H 1.327398 3.585065 0.890968
C -0.918596 -1.293626 1.436111
O 0.217142 -1.283473 1.886413
C -1.934284 -2.244280 2.002217
H -2.227733 -1.869448 2.989628
H -2.829714 -2.328933 1.387544
H -1.463260 -3.220375 2.140642
C -1.303972 -2.617904 -1.103216
H -1.182602 -2.974443 -2.129324
H -0.491008 -3.032744 -0.497634
H -2.265494 -2.970915 -0.726714
C 4.496769 0.018807 0.116669
C 3.130741 -0.650757 0.386105
O 2.783065 -1.592158 -0.281233
O 2.510025 -0.100100 1.395642
F 4.598457 1.237778 0.648624
F 5.474062 -0.740673 0.637959
F 4.715384 0.116118 -1.197369
42
H_6 Eopt -
1731.244525
C 2.147275 0.896440 -0.431844
C 1.673316 1.778889 0.542535
C 1.776377 3.159217 0.390076
C 2.360618 3.656332 -0.775191
C 2.834065 2.786768 -1.759289
C 2.730527 1.405522 -1.589295
C 1.885202 -0.559387 -0.135976
C 1.590664 -0.709963 1.406652
H 1.410686 3.833115 1.158714
H 2.449952 4.729687 -0.911023
H 3.291452 3.184691 -2.659414
H 3.109000 0.730161 -2.350660
S 0.915499 0.968566 1.925180
O 0.553540 -1.668604 1.529264
C -0.133762 -1.691913 0.339504
C 0.558543 -1.054446 -0.684789
C -1.369178 -2.290569 0.153402
C 0.007422 -0.979685 -1.956288
H -1.060496 1.015827 0.808589
C -1.915598 -2.215007 -1.132084
H -1.889771 -2.774545 0.972737
C -1.243376 -1.565782 -2.172902
H 0.532883 -0.472043 -2.760816
H -2.886825 -2.663316 -1.319605
H -1.700265 -1.512168 -3.155716
C 3.063789 -1.488170 -0.521407
O 4.180004 -1.032327 -0.647656
C 2.771378 -2.954072 -0.690571
H 3.708323 -3.511840 -0.687919
H 2.106344 -3.322862 0.097065
H 2.256708 -3.108638 -1.645635
C 2.743442 -1.096743 2.304264
H 2.406613 -1.131141 3.342914
H 3.124705 -2.084816 2.028084
H 3.549581 -0.363720 2.216143
C -4.112036 0.569849 -0.184976
C -2.875310 0.491674 0.736529
O -2.949239 -0.029001 1.817644
O -1.836054 1.091255 0.199399
F -4.863903 1.621827 0.173104
F -3.793438 0.711669 -1.471682
F -4.854107 -0.530136 -0.052049
42
H_7 Eopt -
1731.245365
C 2.025452 0.895501 -0.449308
C 1.569500 1.792710 0.517960
C 1.612470 3.169814 0.316079
C 2.117684 3.647834 -0.892815
C 2.577303 2.762867 -1.869979
C 2.533890 1.385929 -1.649816
C 1.821959 -0.560684 -0.109006
C 1.542939 -0.686567 1.442326
H 1.259866 3.855964 1.079942
H 2.156832 4.718410 -1.068450
H 2.975199 3.145795 -2.804207
H 2.900191 0.698796 -2.406824
S 0.951617 1.011609 1.981034
O 0.470524 -1.606598 1.592226
C -0.193485 -1.689415 0.392401
C 0.514968 -1.109019 -0.651314
C -1.424103 -2.302879 0.215120
C -0.000411 -1.130550 -1.940308
H -1.249832 0.823278 1.634415
C -1.934838 -2.323497 -1.086921
H -1.963083 -2.735202 1.051666
C -1.236044 -1.747151 -2.152724
H 0.540930 -0.670063 -2.762525
H -2.898992 -2.789544 -1.267559
H -1.663134 -1.768990 -3.150020
C 3.031186 -1.452742 -0.487805
O 4.131101 -0.961373 -0.622540
C 2.785806 -2.928456 -0.650557
H 2.308529 -3.103329 -1.621537
H 3.737538 -3.459564 -0.613815
H 2.104625 -3.311476 0.116002
C 2.692821 -1.116626 2.327272
H 3.031416 -2.120510 2.053297
H 3.525425 -0.415525 2.228279
H 2.367190 -1.133270 3.370122
C -3.993623 0.468777 -0.032721
C -2.513236 0.822492 0.226385
O -1.807664 1.263582 -0.639367
O -2.209394 0.625000 1.493348
F -4.778882 1.450748 0.436955
F -4.225349 0.339441 -1.336481
F -4.353160 -0.665237 0.576162
42
H_8 Eopt -
1731.246662
C 2.298846 -1.089933 -0.460405
C 3.241956 -1.341750 0.540993
C 4.295012 -2.228886 0.325466
C 4.398190 -2.858061 -0.915842
C 3.464421 -2.608877 -1.923235
C 2.412040 -1.720005 -1.696818
C 1.264852 -0.055283 -0.091126
C 1.269947 0.125520 1.485637
H 5.022176 -2.426485 1.106957
H 5.214371 -3.551399 -1.094290
H 3.554293 -3.106058 -2.883560
H 1.683947 -1.522135 -2.480527
S 2.971620 -0.427803 2.026389
O 1.141658 1.519843 1.721151
C 1.544661 2.185546 0.592996

C 1.648746 1.349461 -0.510366
C 1.806224 3.545138 0.530351
C 2.030862 1.861597 -1.741395
H -2.419415 0.265157 -1.070165
C 2.183717 4.056380 -0.714617
H 1.723368 4.174646 1.409748
C 2.298944 3.230556 -1.837016
H 2.120724 1.213605 -2.609284
H 2.399497 5.116507 -0.806059
H 2.601489 3.654960 -2.788716
C -0.156899 -0.410749 -0.541377
O -0.908878 0.495232 -0.881119
C -0.620728 -1.837680 -0.520777
H -0.751254 -2.166951 -1.558269
H 0.086296 -2.504456 -0.028451
H -1.593659 -1.886970 -0.022727
C 0.200265 -0.584479 2.289256
H -0.797297 -0.260746 1.972105
H 0.287829 -1.667319 2.177471
H 0.323844 -0.334459 3.346098
C -5.456815 -0.303332 -0.043079
C -3.919477 -0.185531 0.030525
O -3.318496 -0.398527 1.054816
O -3.431385 0.156309 -1.132009
F -5.965662 -0.669428 1.130386
F -6.004025 0.868490 -0.391695
F -5.818528 -1.211695 -0.959836
7
TFA-anion Eopt -
526.190256
C -0.519576 0.011674 -0.006814
C 1.045703 0.007057 -0.019179
O 1.521139 -1.146110 -0.010044
O 1.578395 1.136004 -0.007914
F -1.044320 -0.863163 -0.891267
F -1.069457 1.209785 -0.286999
F -0.992116 -0.350127 1.211557
8
TFAH Eopt -
526.644630
C 0.592349 -0.000569 -0.003093
C -0.943092 0.155147 -0.009427
O -1.486347 1.225107 -0.003376
O -1.511160 -1.039993 -0.005332
F 1.182518 1.181498 -0.143230
F 0.996380 -0.800639 -0.995397
F 0.995332 -0.543414 1.153424
H -2.483561 -0.945382 0.011610
42
TS-III_1 Eopt -
1731.146907
C 1.227467 -1.060031 0.136721
C 1.833966 -2.248728 0.604741
C 1.263279 -3.052885 1.579686
C 0.027615 -2.695303 2.155497
C -0.554749 -1.509673 1.796048
C -0.024455 -0.609903 0.772555
C 2.003024 -0.455053 -0.890112
C 3.180086 -1.141801 -1.148887
H 1.765064 -3.962507 1.900490
H -0.427522 -3.339983 2.898076
H -1.486036 -1.194015 2.261658
H -0.898817 -0.623358 -0.016640
S 3.347516 -2.560992 -0.192147
C 1.531741 0.681818 -1.755859
O 0.408403 0.622851 -2.216469
C 2.466417 1.822400 -2.050614
H 3.152123 2.008449 -1.219558
H 3.060919 1.578854 -2.938425
H 1.879149 2.715453 -2.271275
C -0.051914 0.875405 1.303870
C 1.147375 1.137725 2.160466
C -0.312466 1.926588 0.274790
C 1.887222 2.250344 2.059967
H 1.398088 0.373229 2.895394
C 0.393799 3.064203 0.206028
H -1.149930 1.748279 -0.395694
C 1.556582 3.305069 1.079275
H 2.747659 2.427657 2.699109
H 0.154425 3.841099 -0.514713
H -0.946100 0.895708 1.946704
O 2.226575 4.331343 0.994494
C -4.644317 -0.231035 -0.626673
C -3.210075 -0.157023 -0.028064
O -2.329588 -0.571881 -0.822620
O -3.102774 0.280788 1.122830
F -4.733746 0.462369 -1.777570
F -5.585014 0.250268 0.196749
F -4.982833 -1.505102 -0.910579
C 4.238946 -0.816257 -2.152931
H 4.783130 0.083798 -1.849604
H 4.955852 -1.633099 -2.251285
H 3.787890 -0.631475 -3.132172
42
TS-III_2 Eopt -
1731.150027
C -1.169447 -1.046253 -0.206635
C -1.653660 -2.314531 -0.612586
C -1.028652 -3.090206 -1.575080
C 0.141165 -2.624765 -2.210594
C 0.610691 -1.376675 -1.906643
C 0.031294 -0.510017 -0.879759
C -1.989638 -0.485844 0.808032
C -3.079639 -1.288529 1.120873
H -1.439006 -4.060485 -1.844529
H 0.633515 -3.242445 -2.952359
H 1.491865 -0.986973 -2.410900
H 0.895954 -0.510633 -0.089491
S -3.107200 -2.757519 0.231706
C -1.780021 0.762256 1.627962
O -2.668628 1.588406 1.701342
C -0.540065 0.819422 2.473596
H -0.735772 0.190440 3.353056
H 0.336187 0.402968 1.969038
H -0.352661 1.842608 2.803113
C -0.024005 0.982397 -1.373231
C -1.291497 1.213879 -2.136649
C 0.258353 2.013304 -0.327149
C -2.087894 2.270319 -1.927917
H -1.548419 0.467009 -2.887427
C -0.512597 3.093168 -0.144277
H 1.166605 1.873842 0.254779
C -1.751246 3.298137 -0.921314
H -3.002838 2.420981 -2.493800
H -0.270713 3.851023 0.595799
H 0.820595 1.055206 -2.074914
O -2.464588 4.280923 -0.746854
C 4.607321 -0.188493 0.621540
C 3.151968 -0.034256 0.095197
O 2.249457 -0.423132 0.878492
O 3.060860 0.438885 -1.043635
F 5.283539 0.971335 0.534359
F 5.282642 -1.091904 -0.117923
F 4.678668 -0.592620 1.896793
C -4.158448 -1.006269 2.114501
H -4.863694 -0.281355 1.696340
H -4.704977 -1.914031 2.376430
H -3.735791 -0.572056 3.024552
42
TS-III_3 Eopt -
1731.146907
C -1.226494 -1.060391 -0.135855
C -1.832637 -2.249528 -0.603162
C -1.261395 -3.054325 -1.577267
C -0.025615 -2.696898 -2.152922
C 0.556284 -1.510767 -1.794330
C 0.025404 -0.610375 -0.771733
C -2.002688 -0.454464 0.889929
C -3.179865 -1.141039 1.148644
H -1.762865 -3.964289 -1.897585
H 0.429923 -3.342067 -2.894831
H 1.487536 -1.195133 -2.260030
H 0.899779 -0.622328 0.017524
S -3.346604 -2.561196 0.193210
C -1.531681 0.682555 1.755603
O -0.408550 0.623661 2.216720
C -2.466547 1.823099 2.050146
H -3.154078 2.007377 1.220163
H -3.058980 1.580628 2.939655
H -1.879399 2.716882 2.268139
C 0.051764 0.874548 -1.304262
C 0.310867 1.926587 -0.275702
C -1.147751 1.134907 -2.161138
C -0.396979 3.063242 -0.207470
H 1.148469 1.749648 0.394979
C -1.889175 2.246520 -2.061138
H -1.397494 0.369628 -2.895580
C -1.560058 3.302111 -1.080873
H -0.158743 3.840780 0.512954
H -2.749922 2.422279 -2.700293
H 0.946129 0.895299 -1.946828
O -2.231443 4.327510 -0.996569
C 4.645050 -0.228926 0.626356
C 3.210606 -0.154545 0.028271
O 2.330198 -0.568342 0.823481
O 3.103152 0.281766 -1.123171
F 4.733919 0.459289 1.780336
F 5.584955 0.257238 -0.195109
F 4.985172 -1.503928 0.904372
C -4.239134 -0.814688 2.151987
H -4.782007 0.086164 1.848736
H -4.957101 -1.630710 2.249418
H -3.788568 -0.630903 3.131654
42
TS-III_4 Eopt -
1731.150307
C -1.195168 -1.039598 -0.196655
C -1.730271 -2.290961 -0.591550
C -1.149164 -3.089452 -1.562632
C 0.026620 -2.666539 -2.217690
C 0.545356 -1.436042 -1.923605
C 0.011617 -0.545301 -0.891744
C -1.977767 -0.449227 0.830259
C -3.088907 -1.213987 1.164703
H -1.597309 -4.045369 -1.822975
H 0.485760 -3.303322 -2.964567
H 1.433591 -1.079595 -2.440021
H 0.889161 -0.560447 -0.120143

S -3.182890 -2.682011 0.279958
C -1.712856 0.794120 1.641244
O -2.576193 1.645021 1.732314
C -0.452773 0.818923 2.457749
H -0.646494 0.200199 3.344892
H 0.399869 0.375407 1.936218
H -0.228368 1.837766 2.777590
C -0.012531 0.943775 -1.401325
C 0.305155 1.977203 -0.367735
C -1.282628 1.198168 -2.153051
C -0.439609 3.075260 -0.184950
H 1.215902 1.822426 0.206416
C -2.053060 2.273700 -1.943940
H -1.564807 0.451343 -2.894881
C -1.682399 3.301498 -0.949295
H -0.172454 3.833235 0.546272
H -2.970658 2.440385 -2.500952
H 0.826342 0.987995 -2.112414
O -2.371916 4.301020 -0.774044
C 4.600437 -0.205363 0.613088
C 3.168103 -0.095363 0.017610
O 2.274942 -0.476090 0.818800
O 3.065629 0.328851 -1.137218
F 5.548330 0.269886 -0.204418
F 4.912561 -1.490288 0.877082
F 4.703373 0.468304 1.774400
C -4.137766 -0.894840 2.178866
H -4.815921 -0.133809 1.780781
H -4.719984 -1.780405 2.439478
H -3.680807 -0.491002 3.086491
42
TS-III_5 Eopt -
1731.149905
C 0.003215 1.198757 0.208037
C 0.239148 2.266488 1.100479
C -0.456303 2.425100 2.287005
C -1.460049 1.501737 2.655674
C -1.768207 0.481755 1.801361
C -1.049477 0.223177 0.550928
C 0.901390 1.227807 -0.899894
C 1.773837 2.310199 -0.842252
H -0.225927 3.258194 2.946679
H -1.990179 1.630616 3.591817
H -2.568861 -0.212460 2.050164
H -0.461438 -0.727671 0.840820
S 1.536481 3.283810 0.552563
C 1.024407 0.101004 -1.877796
O 0.690002 -1.015830 -1.517930
C 1.549275 0.339318 -3.265855
H 1.220198 -0.484618 -3.900790
H 1.206631 1.293593 -3.672155
H 2.644386 0.343037 -3.249246
C -2.028993 -0.215865 -0.587976
C -3.086447 0.826226 -0.792625
C -2.563106 -1.588869 -0.325127
C -4.396720 0.548929 -0.778140
H -2.739413 1.845385 -0.959405
C -3.872506 -1.872149 -0.316050
H -1.820622 -2.366068 -0.150114
C -4.888418 -0.824673 -0.544560
H -5.148784 1.317486 -0.932211
H -4.240077 -2.879315 -0.141277
H -1.438733 -0.278917 -1.508386
O -6.088555 -1.081285 -0.536164
C 2.677182 -2.412127 0.449838
C 1.602754 -1.554152 1.180254
O 0.484337 -2.099719 1.329920
O 1.995894 -0.435318 1.537840
F 3.399478 -1.686851 -0.425409
F 2.173593 -3.454263 -0.224953
F 3.558432 -2.917904 1.345097
C 2.896050 2.681850 -1.759398
H 2.523011 2.898839 -2.763456
H 3.423561 3.563812 -1.391909
H 3.613408 1.858435 -1.826907
35
TS-II_1-notfa Eopt -
1204.921838
C -0.181364 0.103898 -1.042625
C -0.667743 1.394782 -0.767488
C -2.000523 1.766462 -0.987021
C -2.889376 0.824469 -1.506903
C -2.453658 -0.472213 -1.746022
C -1.118812 -0.876117 -1.466242
C 1.222014 -0.023621 -0.724245
C 1.757199 1.164348 -0.241555
H -2.333829 2.777607 -0.769696
H -3.917891 1.103658 -1.705437
H -3.145544 -1.214022 -2.134269
H -0.772244 -1.841563 -1.813268
S 0.586969 2.425566 -0.164829
O 0.268761 0.784538 2.750959
C -0.387357 -0.086883 2.199359
C 0.233725 -1.367386 1.767795
C -1.839614 0.095271 1.942153
C -0.451777 -2.214405 0.969973
H 1.238158 -1.576448 2.125460
C -2.501452 -0.781529 1.161419
H -2.322523 0.945974 2.414049
C -1.775906 -1.854914 0.518725
H -0.015768 -3.140997 0.612235
H -3.559416 -0.669469 0.950426
H -2.344365 -2.602280 -0.027395
C 1.970484 -1.296436 -0.949970
O 1.420711 -2.206814 -1.552489
C 3.370859 -1.469396 -0.430854
H 4.067707 -0.858264 -1.013237
H 3.451933 -1.168096 0.616523
H 3.646902 -2.518699 -0.539224
C 3.145431 1.513170 0.200343
H 3.415348 0.950829 1.098795
H 3.872833 1.287317 -0.582444
H 3.218636 2.576622 0.435935
42
TS-II_1 Eopt -
1731.131029
C 0.535344 0.545490 0.038519
C 0.908676 0.715244 -1.308961
C 1.493020 1.892587 -1.782319
C 1.714468 2.946639 -0.888679
C 1.404354 2.788990 0.453929
C 0.859985 1.573149 0.953723
C -0.073332 -0.739593 0.268399
C -0.126892 -1.508220 -0.888724
H 1.756212 1.996139 -2.831311
H 2.145657 3.874488 -1.247295
H 1.593099 3.597581 1.153976
H 0.498527 1.522141 1.974458
S 0.529657 -0.689056 -2.246435
O 3.200477 -2.042414 -1.023046
C 3.250932 -1.203197 -0.133331
C 3.897129 0.112875 -0.364926
C 2.680700 -1.466446 1.211916
C 3.792459 1.088416 0.561046
H 4.447543 0.235908 -1.293065
C 2.588146 -0.462391 2.113947
H 2.367909 -2.484452 1.427053
C 2.989181 0.867135 1.738513
H 4.246337 2.062307 0.413476
H 2.157044 -0.618241 3.095440
H 2.962674 1.643491 2.497197
C -0.666376 -1.106167 1.588729
O -0.301335 -0.511654 2.592886
C -1.740278 -2.156987 1.666506
H -1.288748 -3.154528 1.630998
H -2.260666 -2.042553 2.618655
H -2.444131 -2.068845 0.833290
C -2.930370 0.182335 -1.040227
O -3.169700 -1.015579 -1.275374
O -2.276241 1.034701 -1.662913
C -3.536031 0.734192 0.293708
F -4.348144 -0.129812 0.927882
F -2.557840 1.053306 1.175327
F -4.252689 1.860544 0.098033
C -0.633927 -2.899024 -1.101126
H -1.728065 -2.889311 -1.102441
H -0.292693 -3.290687 -2.061792
H -0.275905 -3.565468 -0.313128
42
TS-II_10 Eopt -
1731.132706
C -2.053568 0.883353 0.754664
C -2.764712 1.686026 -0.161153
C -2.449860 3.022198 -0.389308
C -1.362517 3.588909 0.286771
C -0.602699 2.813854 1.150054
C -0.896835 1.444060 1.368778
C -2.523793 -0.474465 0.773543
C -3.533867 -0.686843 -0.157140
H -3.026690 3.615283 -1.093165
H -1.111576 4.630830 0.122120
H 0.252638 3.246084 1.660537
H -0.407429 0.920107 2.181109
S -3.985756 0.760618 -0.985325
O -0.997902 -2.524927 -1.977932
C -0.472227 -1.561662 -1.435433
C -0.550369 -0.204020 -2.028348
C 0.279940 -1.709470 -0.165189
C -0.008210 0.856155 -1.399838
H -1.049008 -0.116755 -2.989457
C 0.806750 -0.636378 0.455265
H 0.407241 -2.718579 0.216886
C 0.656056 0.688807 -0.117153
H -0.047665 1.851920 -1.830638
H 1.371657 -0.732733 1.377766
H 1.340893 1.453123 0.228015
C -2.028370 -1.464679 1.778931
O -1.464426 -1.051463 2.780373
C -2.238407 -2.941140 1.574990
H -2.122274 -3.241218 0.530926
H -1.524868 -3.477501 2.202591
H -3.250818 -3.208473 1.897818
C 3.720706 0.635077 -0.127856
O 3.495832 1.344529 0.866511
O 3.071711 0.482698 -1.182204
C 4.982938 -0.276309 -0.038540
F 5.687308 -0.295159 -1.185063
F 5.835063 0.077403 0.935974

F 4.616633 -1.555341 0.211288
C -4.263894 -1.938453 -0.525083
H -4.658267 -2.444638 0.358612
H -5.095073 -1.721061 -1.198568
H -3.581784 -2.625196 -1.040860
42
TS-II_11 Eopt -
1731.123084
C -1.530293 0.477076 -1.111777
C -2.602987 1.087800 -0.419181
C -3.931582 0.736421 -0.638472
C -4.238207 -0.264036 -1.579578
C -3.215465 -0.970675 -2.173712
C -1.846085 -0.697018 -1.866113
C -0.275721 1.099293 -0.816551
C -0.400296 2.087384 0.152346
H -4.729876 1.250761 -0.109977
H -5.274203 -0.502689 -1.791382
H -3.434010 -1.792413 -2.849163
H -1.091726 -1.167614 -2.484337
S -2.032185 2.326654 0.657570
O -2.425540 -0.318213 3.207570
C -2.174748 -0.979153 2.210373
C -3.252773 -1.693337 1.477193
C -0.792474 -1.109326 1.691763
C -2.989141 -2.319713 0.313822
H -4.234539 -1.713553 1.941295
C -0.564998 -1.721361 0.514771
H 0.019488 -0.683551 2.273500
C -1.668838 -2.239414 -0.279758
H -3.764561 -2.860550 -0.218298
H 0.450302 -1.784282 0.139046
H -1.431563 -2.951827 -1.064784
C 1.035509 0.895837 -1.514750
O 1.852538 1.800118 -1.505687
C 1.304994 -0.358072 -2.303413
H 0.767923 -0.298372 -3.257798
H 2.374120 -0.406128 -2.513749
H 0.992170 -1.262587 -1.779860
C 3.176312 0.133355 0.826785
O 2.002150 -0.282349 0.849591
O 3.692998 1.155978 1.300954
C 4.196308 -0.784137 0.077157
F 3.649907 -1.902358 -0.436436
F 4.788823 -0.140755 -0.950858
F 5.189963 -1.188567 0.898423
C 0.669738 2.919496 0.774421
H 0.965840 3.720208 0.089791
H 0.321261 3.363968 1.709707
H 1.553431 2.300496 0.963350
42
TS-II_12 Eopt -
1731.137385
C -1.033945 -0.034668 -0.931349
C -2.062250 -0.861325 -1.425617
C -1.837219 -2.179642 -1.838030
C -0.546990 -2.705653 -1.754073
C 0.478005 -1.939819 -1.211722
C 0.244278 -0.621220 -0.737021
C -1.528174 1.265733 -0.557545
C -2.890623 1.394246 -0.774640
H -2.654701 -2.781309 -2.225617
H -0.354397 -3.719064 -2.087805
H 1.480454 -2.343068 -1.100806
H 1.098310 -0.019124 -0.446248
S -3.585878 -0.030723 -1.451345
O -3.733056 -0.521477 1.911671
C -2.543736 -0.805419 1.902189
C -2.087936 -2.178107 1.564685
C -1.505626 0.207596 2.232435
C -0.776289 -2.427575 1.384944
H -2.852155 -2.945864 1.484881
C -0.200642 -0.071095 2.024451
H -1.851881 1.157115 2.631039
C 0.178247 -1.340323 1.449461
H -0.415014 -3.419378 1.134335
H 0.589827 0.649303 2.212068
H 1.241988 -1.563567 1.373929
C -0.721973 2.423327 -0.055249
O -1.259702 3.280470 0.627269
C 0.731155 2.526971 -0.429423
H 0.924280 2.134805 -1.431479
H 1.031111 3.574507 -0.367672
H 1.338838 1.955928 0.286021
C 3.402906 -0.351351 0.538408
O 2.775725 0.442835 1.266479
O 3.209994 -1.557151 0.305576
C 4.572405 0.317830 -0.244500
F 5.349390 -0.551576 -0.907696
F 4.081608 1.180996 -1.164440
F 5.381087 1.031707 0.560723
C -3.780076 2.565146 -0.509897
H -3.291770 3.492968 -0.815397
H -4.729756 2.463505 -1.039210
H -3.982640 2.639346 0.563187
42
TS-II_13 Eopt -
1731.127730
C -0.637433 0.541168 -0.385385
C -0.898280 1.309402 0.769665
C -1.589771 2.519250 0.724975
C -2.043511 3.001205 -0.511088
C -1.847161 2.248884 -1.655883
C -1.198643 0.980613 -1.609252
C 0.063940 -0.670426 -0.064077
C 0.342498 -0.776684 1.294914
H -1.761175 3.090636 1.633102
H -2.559070 3.953526 -0.560960
H -2.209403 2.608711 -2.614279
H -0.929442 0.508209 -2.547042
S -0.234314 0.567579 2.186277
O -2.832326 -1.453483 2.103959
C -3.067536 -1.140031 0.944975
C -3.873458 0.062774 0.625456
C -2.558641 -1.953849 -0.191526
C -3.965641 0.493162 -0.648948
H -4.373828 0.557202 1.452890
C -2.670694 -1.489715 -1.457937
H -2.110146 -2.913728 0.048714
C -3.222407 -0.182373 -1.688193
H -4.539642 1.375749 -0.909602
H -2.282028 -2.051090 -2.302001
H -3.372445 0.137242 -2.715032
C 0.534824 -1.716406 -1.023497
O 0.665525 -2.869700 -0.648685
C 0.853349 -1.325745 -2.443565
H 1.229345 -0.302981 -2.516942
H 1.589859 -2.027964 -2.837846
H -0.057269 -1.411550 -3.049879
C 2.907005 1.036865 0.676161
O 3.158499 0.568879 1.800701
O 2.177440 1.972166 0.308925
C 3.647417 0.271798 -0.471008
F 3.230958 -1.016635 -0.547003
F 3.465376 0.798953 -1.694991
F 4.980818 0.225781 -0.269235
C 1.054789 -1.870003 2.019362
H 1.916443 -2.208582 1.440747
H 1.394593 -1.527825 2.998235
H 0.381892 -2.723935 2.150452
35
TS-II_2-notfa Eopt -
1204.919654
C 0.141592 0.190646 -1.022426
C 0.658232 -1.117579 -1.125099
C 1.985729 -1.378504 -1.470596
C 2.847789 -0.305172 -1.727252
C 2.391617 0.993343 -1.575409
C 1.059555 1.270984 -1.149203
C -1.242764 0.185133 -0.638168
C -1.738401 -1.100838 -0.462548
H 2.342605 -2.401595 -1.551081
H 3.872989 -0.496332 -2.022884
H 3.062012 1.829844 -1.748667
H 0.707964 2.294013 -1.217577
S -0.556233 -2.308955 -0.780903
O -0.134853 -1.591205 2.477093
C 0.452112 -0.565574 2.165049
C -0.258427 0.740648 2.098443
C 1.900485 -0.579346 1.840645
C 0.352603 1.813319 1.551120
H -1.265793 0.777640 2.503672
C 2.481270 0.511103 1.305150
H 2.448106 -1.490589 2.062446
C 1.675091 1.673338 0.987559
H -0.154105 2.769915 1.464147
H 3.536201 0.527327 1.053527
H 2.191296 2.577862 0.679987
C -2.138255 1.375315 -0.494323
O -3.117960 1.313282 0.228524
C -1.826436 2.627175 -1.271300
H -1.118215 3.237705 -0.698272
H -1.386800 2.406885 -2.247061
H -2.747753 3.199210 -1.390373
C -3.107830 -1.530037 -0.050745
H -3.235800 -1.362410 1.023566
H -3.866548 -0.937151 -0.566131
H -3.267691 -2.589368 -0.261159
42
TS-II_2 Eopt -
1731.128765
C -0.342838 -0.091232 0.427190
C 0.150280 -0.429118 -0.847849
C 0.776171 -1.652933 -1.117851
C 0.908715 -2.584415 -0.085834
C 0.387658 -2.299311 1.169730
C -0.279243 -1.074912 1.440269
C -0.924727 1.232105 0.442488
C -0.858648 1.841783 -0.806702
H 1.170720 -1.859780 -2.107772
H 1.410549 -3.527447 -0.271807
H 0.480201 -3.025195 1.972499
H -0.555377 -0.827089 2.457386
S -0.098208 0.849291 -1.980152
O -3.149420 -0.112824 -2.181142
C -3.049468 -0.781659 -1.160425
C -2.495078 -2.156855 -1.203088
C -3.482705 -0.249796 0.156183

C -2.238544 -2.817528 -0.053761
H -2.320296 -2.588731 -2.184227
C -3.199745 -0.936177 1.288442
H -4.036179 0.685251 0.154463
C -2.436080 -2.149762 1.206442
H -1.823055 -3.819293 -0.055218
H -3.478644 -0.559944 2.265811
H -2.256841 -2.707744 2.120575
C -1.449802 1.846272 1.695163
O -1.585477 1.147351 2.690442
C -1.794422 3.309308 1.749479
H -2.665235 3.521945 1.121546
H -2.028370 3.565845 2.783102
H -0.962247 3.922696 1.394316
C 3.075512 0.721750 -0.724366
O 2.579270 1.799382 -0.352380
O 3.190170 0.206190 -1.849090
C 3.666871 -0.113429 0.459535
F 2.780052 -0.261154 1.467187
F 4.063242 -1.352326 0.116590
F 4.749869 0.498291 0.993396
C -1.343375 3.180839 -1.273693
H -0.782543 3.986703 -0.792675
H -1.214626 3.279431 -2.353429
H -2.404751 3.311032 -1.047459
42
TS-II_3 Eopt -
1731.132879
C -1.148843 -0.133204 -0.969398
C -2.482807 -0.511185 -1.204246
C -2.848750 -1.814540 -1.569070
C -1.854522 -2.781929 -1.706408
C -0.531748 -2.452151 -1.429248
C -0.158028 -1.148608 -1.009270
C -1.035830 1.265260 -0.616721
C -2.271261 1.895714 -0.586998
H -3.890284 -2.063994 -1.751635
H -2.118142 -3.789895 -2.006581
H 0.248567 -3.203792 -1.512505
H 0.895751 -0.900343 -0.948768
S -3.562744 0.828557 -0.997861
O -3.215144 0.402669 2.311028
C -2.246701 -0.317089 2.109355
C -2.423890 -1.753856 1.769360
C -0.859441 0.204737 2.203886
C -1.365745 -2.489887 1.375646
H -3.425347 -2.163036 1.867393
C 0.175669 -0.550182 1.777444
H -0.733084 1.199556 2.622793
C -0.077585 -1.854759 1.209754
H -1.465965 -3.539438 1.120916
H 1.203175 -0.202545 1.785039
H 0.794711 -2.453006 0.963232
C 0.288842 1.910310 -0.365147
O 1.291187 1.213828 -0.337390
C 0.395991 3.397443 -0.162706
H -0.163363 3.714423 0.722177
H 1.449894 3.645773 -0.033915
H -0.004208 3.934595 -1.027561
C 3.270564 -0.915812 -0.397527
O 2.792228 -1.578393 0.545152
O 3.173739 -1.062845 -1.625217
C 4.190738 0.257238 0.061041
F 5.452003 -0.206482 0.263752
F 4.295375 1.251025 -0.834363
F 3.806346 0.814645 1.223399
C -2.655579 3.300323 -0.232031
H -2.173005 4.024274 -0.892120
H -3.736112 3.435496 -0.311405
H -2.364729 3.523314 0.798733
42
TS-II_4 Eopt -
1731.130394
C 1.941881 0.875958 0.648262
C 2.789743 0.923514 -0.477877
C 4.112696 0.484887 -0.432249
C 4.631126 -0.011494 0.775182
C 3.801020 -0.153857 1.870636
C 2.420659 0.195203 1.805582
C 0.655147 1.453424 0.388639
C 0.531089 1.880853 -0.923576
H 4.745978 0.549731 -1.313043
H 5.670981 -0.314048 0.827177
H 4.181178 -0.582943 2.793026
H 1.833926 0.224747 2.715797
S 1.973126 1.622355 -1.843921
O 1.352648 -1.880449 -2.674221
C 1.411854 -1.981638 -1.457214
C 2.646146 -2.470034 -0.789795
C 0.254774 -1.633471 -0.596839
C 2.739329 -2.456818 0.554452
H 3.433302 -2.860867 -1.428017
C 0.389336 -1.599582 0.743679
H -0.687445 -1.385684 -1.075715
C 1.673851 -1.887551 1.354377
H 3.624706 -2.826896 1.060379
H -0.442768 -1.299752 1.371475
H 1.696132 -2.068932 2.424849
C -0.375944 1.601890 1.463893
O -0.295195 0.895731 2.458340
C -1.418371 2.677617 1.356645
H -2.162169 2.407505 0.600660
H -1.911742 2.775875 2.324983
H -0.963027 3.630737 1.069911
C -3.344836 0.264207 -0.261574
O -2.179486 -0.065430 0.034326
O -3.795508 1.320869 -0.731598
C -4.390351 -0.853751 0.042432
F -4.488973 -1.075224 1.372496
F -5.628791 -0.575977 -0.394200
F -4.038306 -2.031538 -0.515649
C -0.639604 2.493245 -1.621547
H -0.789094 3.524432 -1.285062
H -0.484148 2.507834 -2.702413
H -1.548156 1.923845 -1.398509
42
TS-II_5 Eopt -
1731.134043
C -2.110507 0.965446 0.455704
C -2.132412 1.801086 -0.679614
C -1.666380 3.111308 -0.653680
C -1.123559 3.621330 0.534618
C -1.026758 2.810966 1.651848
C -1.450565 1.451751 1.627070
C -2.592665 -0.354715 0.172564
C -2.916216 -0.512242 -1.170237
H -1.708241 3.731564 -1.544734
H -0.770617 4.645830 0.567018
H -0.588925 3.196202 2.567681
H -1.576520 0.926443 2.566890
S -2.731199 0.948661 -2.072420
O 0.103913 -2.483090 -1.315897
C 0.318299 -1.576824 -0.523335
C 0.679606 -0.212656 -0.984665
C 0.196326 -1.781318 0.940224
C 0.769081 0.810553 -0.114783
H 0.867722 -0.087717 -2.047353
C 0.270413 -0.745560 1.796841
H 0.067654 -2.804074 1.282965
C 0.514850 0.598683 1.306915
H 1.035975 1.810297 -0.444478
H 0.186868 -0.892112 2.868844
H 0.970905 1.291586 2.004097
C -2.809137 -1.362928 1.256610
O -2.917462 -0.969097 2.407614
C -2.913265 -2.829549 0.934587
H -2.255244 -3.126820 0.115152
H -2.671636 -3.395176 1.836154
H -3.945956 -3.062662 0.650832
C 3.386121 -0.675560 0.586334
O 3.078391 -1.832671 0.255815
O 3.203218 -0.047685 1.645985
C 4.165057 0.123429 -0.508914
F 3.969565 1.454125 -0.437430
F 5.497035 -0.075771 -0.375499
F 3.846639 -0.243501 -1.763420
C -3.387999 -1.720174 -1.912774
H -4.226758 -2.198375 -1.403029
H -3.701872 -1.458869 -2.924960
H -2.569531 -2.446177 -1.989231
42
TS-II_6 Eopt -
1731.132091
C 1.441817 0.050898 -1.098678
C 0.550747 1.096322 -0.800798
C -0.824413 1.016371 -1.059654
C -1.333790 -0.139317 -1.653684
C -0.490324 -1.211891 -1.909671
C 0.893383 -1.168227 -1.582307
C 2.797820 0.373994 -0.724713
C 2.902174 1.646937 -0.183729
H -1.482969 1.844901 -0.814513
H -2.388340 -0.207714 -1.894584
H -0.890917 -2.122742 -2.344838
H 1.550023 -1.946656 -1.950961
S 1.381307 2.455167 -0.115560
O 1.653405 0.615049 2.789771
C 1.298420 -0.366622 2.152092
C -0.131915 -0.605449 1.836329
C 2.289026 -1.369661 1.673990
C -0.485295 -1.595734 0.991756
H -0.877320 0.032433 2.302653
C 1.911196 -2.330978 0.806175
H 3.302378 -1.286160 2.057101
C 0.543918 -2.365591 0.328997
H -1.530956 -1.776722 0.750472
H 2.615301 -3.060225 0.418581
H 0.240937 -3.228222 -0.256683
C 3.925457 -0.577254 -0.951961
O 3.764478 -1.515721 -1.718273
C 5.230397 -0.406636 -0.222103
H 5.072764 -0.177527 0.834553
H 5.802250 -1.329909 -0.324311
H 5.805533 0.412876 -0.664812
C -3.738642 -0.499197 0.594638
O -3.113918 0.096837 1.490016
O -3.678939 -1.675464 0.196156
C -4.777246 0.410014 -0.138445

F -5.791715 0.753262 0.684691
F -4.220891 1.566475 -0.561792
F -5.334515 -0.161193 -1.219487
C 4.096410 2.403712 0.311549
H 4.886770 2.419275 -0.442526
H 3.829573 3.436037 0.546068
H 4.493088 1.945015 1.221989
42
TS-II_7 Eopt -1731.132198
C 1.730786 -0.365919 -0.966425
C 0.373433 -0.377212 -1.339244
C -0.293347 -1.541719 -1.743173
C 0.415766 -2.746155 -1.781669
C 1.738172 -2.780684 -1.367098
C 2.404004 -1.614065 -0.895008
C 2.182768 0.959444 -0.609586
C 1.161688 1.897982 -0.722089
H -1.344319 -1.488534 -2.013813
H -0.081621 -3.651744 -2.110654
H 2.285977 -3.718670 -1.371012
H 3.473533 -1.640821 -0.729610
S -0.322686 1.208387 -1.250997
O -0.406143 0.847898 2.014303
C 0.332297 -0.124625 1.936791
C -0.205154 -1.463799 1.584265
C 1.790445 -0.009380 2.198340
C 0.642835 -2.481955 1.329132
H -1.286296 -1.565683 1.522958
C 2.610262 -1.042054 1.907029
H 2.145907 0.921936 2.630741
C 2.065203 -2.239181 1.308945
H 0.276744 -3.466696 1.059876
H 3.682251 -0.979823 2.057791
H 2.730654 -3.087680 1.181308
C 3.596445 1.234314 -0.224366
O 4.385957 0.302565 -0.146585
C 4.062278 2.636509 0.059209
H 3.505193 3.076132 0.891309
H 5.122287 2.597801 0.311759
H 3.919553 3.275986 -0.816548
C -3.253725 -0.482419 -0.540782
O -3.053041 -0.070365 -1.699493
O -2.944258 -1.553450 0.008291
C -4.059671 0.518859 0.345521
F -5.359899 0.541653 -0.029682
F -4.043938 0.216322 1.654318
F -3.607569 1.782464 0.234151
C 1.138193 3.367854 -0.428528
H 1.862297 3.908448 -1.042220
H 0.147544 3.781128 -0.629148
H 1.369745 3.552145 0.624411
42
TS-II_8 Eopt -1731.130064
C 1.474805 -0.104557 -1.061631
C 0.580403 0.977154 -0.939305
C -0.786918 0.863128 -1.210253
C -1.296983 -0.368588 -1.632094
C -0.459832 -1.469938 -1.701714
C 0.917355 -1.384996 -1.344558
C 2.817370 0.273472 -0.708501
C 2.909065 1.607857 -0.336724
H -1.444311 1.721727 -1.104348
H -2.348041 -0.466865 -1.877193
H -0.858760 -2.437081 -1.993045
H 1.554847 -2.227508 -1.587767
S 1.395631 2.421707 -0.428063
O 1.602366 1.045236 2.746687
C 1.264267 -0.014352 2.238210
C -0.157798 -0.304994 1.931672
C 2.271069 -1.062355 1.912136
C -0.488685 -1.399469 1.218568
H -0.915960 0.381525 2.297716
C 1.912600 -2.139496 1.184061
H 3.279984 -0.912122 2.285836
C 0.556131 -2.250323 0.686035
H -1.527748 -1.619663 0.982841
H 2.630238 -2.908918 0.916035
H 0.260334 -3.199174 0.247968
C 4.041995 -0.581857 -0.773336
O 5.005279 -0.318512 -0.072622
C 4.084710 -1.743614 -1.732293
H 3.510105 -1.551388 -2.641366
H 5.127800 -1.946688 -1.980512
H 3.676819 -2.633619 -1.238529
C -3.754475 -0.438471 0.629056
O -3.167962 0.266224 1.469647
O -3.651354 -1.647288 0.356466
C -4.784583 0.364676 -0.228948
F -5.747508 0.915021 0.539098
F -4.186881 1.387302 -0.884103
F -5.409335 -0.370901 -1.163649
C 4.108811 2.378501 0.107790
H 4.970027 2.143494 -0.521566
H 3.918922 3.453263 0.076743
H 4.366693 2.096963 1.133632
42
TS-II_9 Eopt -1731.131320
C -2.092640 0.938368 0.409631
C -2.091410 1.775275 -0.729591
C -1.633563 3.086344 -0.698254
C -1.113505 3.605845 0.497909
C -1.025061 2.797723 1.614211
C -1.429126 1.429752 1.581204
C -2.578889 -0.374098 0.113060
C -2.863720 -0.530378 -1.242002
H -1.663799 3.703388 -1.592089
H -0.768743 4.632892 0.532990
H -0.598722 3.183195 2.535273
H -1.550615 0.920961 2.530879
S -2.654555 0.924886 -2.139423
O 0.134833 -2.556146 -1.257293
C 0.319211 -1.625923 -0.486713
C 0.684437 -0.273906 -0.979494
C 0.174207 -1.791509 0.980688
C 0.770865 0.771883 -0.137105
H 0.882545 -0.177699 -2.043424
C 0.269318 -0.737072 1.810896
H 0.026254 -2.803677 1.345277
C 0.507239 0.598922 1.289350
H 1.043189 1.761710 -0.491435
H 0.198064 -0.857681 2.887299
H 0.978800 1.301205 1.966565
C -2.917826 -1.456023 1.089415
O -3.010062 -2.612813 0.715715
C -3.219656 -1.079321 2.519038
H -3.677950 -0.090667 2.596989
H -3.883299 -1.836968 2.939026
H -2.292799 -1.082544 3.103407
C 3.400355 -0.663940 0.630051
O 3.103404 -1.831573 0.331596
O 3.203529 -0.004065 1.668377
C 4.186304 0.112765 -0.476294
F 3.913084 1.432472 -0.492489
F 5.519481 0.001894 -0.270245
F 3.951537 -0.341487 -1.719758
C -3.321591 -1.754561 -1.956920
H -4.164571 -2.211057 -1.431921
H -3.606085 -1.530395 -2.986492
H -2.509632 -2.490848 -1.960802
35
TS-IV_1-notfa Eopt -1204.920383
C 0.360743 -0.127869 -1.028322
C 0.884414 -1.395623 -0.709275
C 2.228570 -1.724870 -0.870369
C 3.076375 -0.746900 -1.389792
C 2.574116 0.506644 -1.743274
C 1.223847 0.831534 -1.568693
C -1.049549 -0.014289 -0.626454
C -1.553661 -1.246636 -0.145971
H 2.603293 -2.709083 -0.606384
H 4.128643 -0.970618 -1.529714
H 3.243135 1.251101 -2.163629
H 0.853423 1.804720 -1.859001
S -0.354100 -2.440685 -0.068768
O -0.131981 -1.085141 2.450895
C 0.283005 -0.028331 1.987568
C -0.687110 0.987005 1.463862
C 1.717876 0.279858 1.916292
C -0.211626 2.252412 1.084138
H -1.731827 0.830677 1.726984
C 2.118588 1.479758 1.445642
H 2.407726 -0.482822 2.263654
C 1.147560 2.469874 1.027109
H -0.905845 3.039302 0.811110
H 3.171980 1.733329 1.386096
H 1.515653 3.440910 0.707821
C -1.909694 1.123426 -1.121087
O -1.379476 2.007102 -1.768266
C -3.379011 1.168798 -0.816295
H -3.902377 0.409428 -1.406369
H -3.581014 0.975681 0.239895
H -3.752858 2.154381 -1.094656
C -2.910374 -1.602343 0.368850
H -3.195513 -0.923188 1.178659
H -3.663351 -1.532462 -0.420233
H -2.918232 -2.620781 0.761450
42
TS-IV_1 Eopt -1731.132171
C -0.463210 0.595461 -0.315796
C -0.485270 1.209167 0.953418
C -0.885552 2.527967 1.150849
C -1.256514 3.271214 0.028075
C -1.214776 2.694518 -1.240620
C -0.825280 1.359987 -1.428919
C -0.119130 -0.827230 -0.214017
C 0.218770 -1.187320 1.102704
H -0.897923 2.966723 2.143972
H -1.565776 4.304159 0.147865
H -1.486581 3.288298 -2.107940
H -0.779941 0.938018 -2.423325
S 0.023866 0.101828 2.190603
O -2.594203 -0.963671 2.181476
C -2.875991 -0.667140 1.021778

C -3.585917 0.575712 0.693112
C -2.486896 -1.573905 -0.093985
C -3.875192 0.858475 -0.597366
H -3.860642 1.224622 1.518641
C -2.884439 -1.272282 -1.394948
H -2.123801 -2.558170 0.188838
C -3.502012 -0.057650 -1.646986
H -4.395909 1.771394 -0.867330
H -2.677495 -1.959473 -2.207936
H -3.774137 0.194577 -2.668231
C 0.146318 -1.665525 -1.437846
O 0.059004 -1.132662 -2.530048
C 0.446623 -3.132552 -1.319721
H -0.315158 -3.648868 -0.727476
H 0.473205 -3.551305 -2.326493
H 1.418954 -3.270886 -0.841485
C 3.140831 -0.532039 0.274609
O 2.926618 -1.479954 -0.503550
O 3.332795 -0.505756 1.501389
C 3.191611 0.879662 -0.395523
F 2.718949 0.902305 -1.652125
F 2.487769 1.799182 0.297675
F 4.464211 1.337053 -0.452646
C 0.698321 -2.486703 1.659689
H 1.672103 -2.726170 1.221564
H 0.813505 -2.420758 2.743148
H -0.008099 -3.290834 1.435250
42
TS-IV_10 Eopt -
1731.129955
C -1.908232 0.114347 -1.039795
C -2.851954 1.053588 -0.571641
C -4.226896 0.834585 -0.604389
C -4.681922 -0.368025 -1.142228
C -3.771494 -1.306018 -1.631868
C -2.391741 -1.080078 -1.586112
C -0.532567 0.558583 -0.761301
C -0.511705 1.848501 -0.182991
H -4.921283 1.577097 -0.222995
H -5.747045 -0.569796 -1.182813
H -4.136422 -2.235615 -2.057299
H -1.726281 -1.837753 -1.977757
S -2.067687 2.463692 0.088182
O -1.479879 1.015538 2.481090
C -1.526820 -0.094025 1.960998
C -2.760337 -0.895985 1.976839
C -0.312654 -0.661834 1.288510
C -2.754918 -2.139632 1.453810
H -3.638027 -0.445053 2.429380
C -0.349752 -2.000249 0.859956
H 0.643949 -0.164585 1.470411
C -1.537021 -2.696084 0.901101
H -3.650307 -2.752872 1.458233
H 0.570944 -2.455662 0.512528
H -1.558977 -3.728146 0.561254
C 0.725034 0.070180 -1.453467
O 1.722139 0.764486 -1.406093
C 0.711082 -1.198879 -2.254924
H 0.047297 -1.075905 -3.118235
H 1.727668 -1.390371 -2.598048
H 0.359465 -2.049499 -1.668085
C 3.094873 -0.522653 0.669033
O 2.639467 -1.627759 0.327046
O 2.707791 0.285219 1.535223
C 4.397406 -0.090584 -0.071029
F 4.531060 -0.640008 -1.289300
F 4.505405 1.239347 -0.227319
F 5.483252 -0.480877 0.644462
C 0.659684 2.665835 0.242608
H 1.167022 3.071948 -0.638362
H 0.343676 3.487226 0.889344
H 1.387327 2.034453 0.764167
42
TS-IV_11 Eopt -
1731.133492
C -1.473528 -0.373459 -0.981314
C -2.698608 -1.070784 -1.000138
C -2.781345 -2.449108 -1.184326
C -1.590753 -3.151253 -1.373102
C -0.367338 -2.478688 -1.371818
C -0.290399 -1.094912 -1.172032
C -1.669083 1.031623 -0.613699
C -3.035969 1.359476 -0.508884
H -3.739814 -2.958730 -1.183054
H -1.621252 -4.225327 -1.523148
H 0.552793 -3.035774 -1.520789
H 0.688130 -0.625385 -1.156869
S -4.040717 0.006243 -0.717459
O -3.389180 -0.047862 2.124096
C -2.200719 -0.309787 1.971199
C -1.689044 -1.685168 2.042586
C -1.225775 0.793314 1.687158
C -0.367099 -1.912319 1.888827
H -2.410162 -2.475318 2.226224
C 0.143151 0.504144 1.634739
H -1.589087 1.809114 1.823018
C 0.551285 -0.813109 1.665736
H 0.035825 -2.918991 1.933615
H 0.886493 1.284103 1.500806
H 1.611230 -1.029832 1.562589
C -0.660988 2.149948 -0.759202
O -0.873019 3.208639 -0.195678
C 0.566545 1.923849 -1.587230
H 0.350774 1.343061 -2.487557
H 0.995496 2.893293 -1.844600
H 1.301089 1.365199 -0.988644
C 3.475489 0.176306 -0.032355
O 3.088130 0.995224 0.820035
O 2.835631 -0.546610 -0.817345
C 5.026647 0.011167 -0.080705
F 5.461222 -0.684124 0.996464
F 5.467677 -0.646708 -1.165140
F 5.668534 1.194728 -0.057375
C -3.656890 2.681483 -0.209326
H -3.244427 3.452697 -0.864699
H -4.740999 2.640754 -0.329439
H -3.427471 2.974717 0.820792
42
TS-IV_12 Eopt -
1731.131722
C -1.222077 -0.328681 -0.957074
C -2.418975 -1.071610 -1.016844
C -2.442601 -2.451310 -1.209576
C -1.220448 -3.106769 -1.364117
C -0.023984 -2.388254 -1.329592
C -0.010300 -1.004121 -1.126132
C -1.482332 1.064423 -0.582465
C -2.862178 1.341544 -0.520817
H -3.380196 -2.997910 -1.239217
H -1.205837 -4.180879 -1.516237
H 0.926662 -2.898338 -1.449129
H 0.945149 -0.494681 -1.124599
S -3.808092 -0.045903 -0.774499
O -3.264980 -0.134810 2.060463
C -2.060790 -0.344047 1.955148
C -1.495430 -1.697996 2.020878
C -1.123775 0.804657 1.738045
C -0.158194 -1.865167 1.938134
H -2.191221 -2.522001 2.143752
C 0.257257 0.574911 1.763702
H -1.536546 1.801759 1.872419
C 0.724590 -0.723643 1.808281
H 0.287449 -2.853935 1.978408
H 0.950490 1.408645 1.712751
H 1.793926 -0.913088 1.776507
C -0.509809 2.216973 -0.674097
O -0.763967 3.252344 -0.085214
C 0.739313 2.056207 -1.487351
H 0.565757 1.483659 -2.401517
H 1.137094 3.045332 -1.717828
H 1.485869 1.513213 -0.893278
C 3.405459 -0.680337 -0.674186
O 3.041713 -1.655770 0.010057
O 3.165855 -0.368568 -1.851602
C 4.257157 0.347424 0.137306
F 5.277758 -0.235852 0.794777
F 4.784697 1.332693 -0.604742
F 3.487639 0.947473 1.081762
C -3.539599 2.636759 -0.226434
H -3.115771 3.437640 -0.837057
H -4.613557 2.566825 -0.408514
H -3.379327 2.908027 0.822863
42
TS-IV_13 Eopt -
1731.131706
C -1.014903 -0.285621 -0.937103
C -2.016801 -1.257798 -1.134506
C -1.729986 -2.604660 -1.347118
C -0.389115 -2.986284 -1.378979
C 0.623202 -2.039620 -1.207505
C 0.322051 -0.691736 -0.982821
C -1.598480 1.008481 -0.570960
C -3.005738 0.989627 -0.652029
H -2.523426 -3.332770 -1.484321
H -0.133454 -4.027815 -1.542740
H 1.667965 -2.336585 -1.241204
H 1.131269 0.018403 -0.854509
S -3.608386 -0.552470 -1.026835
O -3.353838 -0.572108 1.862897
C -2.127844 -0.548460 1.873508
C -1.324265 -1.773399 1.984143
C -1.408537 0.762124 1.751466
C 0.021571 -1.683755 2.034333
H -1.859931 -2.716397 2.028416
C -0.017015 0.794716 1.915036
H -2.012713 1.660505 1.854131
C 0.680140 -0.393049 2.003010
H 0.643140 -2.570325 2.108136
H 0.508857 1.744368 1.935478
H 1.764024 -0.385331 2.078577
C -0.894766 2.345352 -0.569164
O -1.404380 3.281311 0.019336
C 0.410140 2.486907 -1.295758
H 0.447366 1.883524 -2.205389
H 0.569812 3.541758 -1.523440
H 1.216035 2.155126 -0.629417
C 3.630282 -0.883937 -0.212122
O 3.294118 -1.451365 0.843941

O 3.778479 -1.313656 -1.366732
C 3.945781 0.637851 -0.033762
F 5.205153 0.817636 0.421016
F 3.839644 1.345946 -1.170998
F 3.128395 1.235245 0.862691
C -3.964669 2.103910 -0.405258
H -3.663275 2.998426 -0.955708
H -4.977820 1.820177 -0.695132
H -3.963726 2.359651 0.660115
42
TS-IV_14 Eopt -
1731.129209
C -1.244842 -0.130372 -1.007082
C -1.283756 -1.534945 -0.896916
C -0.152284 -2.335761 -1.047893
C 1.056072 -1.706532 -1.345924
C 1.108161 -0.319845 -1.498193
C -0.027137 0.478791 -1.322987
C -2.524539 0.467675 -0.618816
C -3.509990 -0.511146 -0.382546
H -0.207585 -3.413810 -0.931860
H 1.968225 -2.285584 -1.450276
H 2.052109 0.151325 -1.754586
H 0.052977 1.552625 -1.438720
S -2.887999 -2.086805 -0.493199
O -2.404356 -1.345143 2.245586
C -1.546619 -0.505637 1.991117
C -0.113446 -0.822752 2.004702
C -1.959983 0.888700 1.627668
C 0.791878 0.148085 1.752721
H 0.168867 -1.848184 2.223274
C -0.975504 1.874370 1.479291
H -3.002144 1.149163 1.796036
C 0.354415 1.502913 1.491504
H 1.855529 -0.078031 1.734391
H -1.258658 2.910535 1.323485
H 1.116822 2.264469 1.350092
C -2.962344 1.889929 -0.874173
O -3.898070 2.346759 -0.243759
C -2.241332 2.700038 -1.913249
H -1.904078 2.088398 -2.752684
H -2.905747 3.494451 -2.255967
H -1.361817 3.165168 -1.449910
C 4.039787 -0.891585 -0.066481
O 3.560438 -1.289204 1.011685
O 4.256379 -1.473373 -1.140419
C 4.445802 0.619631 -0.025422
F 5.508908 0.818997 0.783471
F 4.770378 1.134577 -1.222766
F 3.447909 1.394637 0.463383
C -4.940920 -0.317279 -0.011440
H -5.411934 0.419365 -0.666609
H -5.490546 -1.258740 -0.065570
H -5.004428 0.065847 1.013269
42
TS-IV_15 Eopt -
1731.126952
C -1.581164 -0.383394 1.056454
C -0.756479 0.709752 1.394504
C 0.523971 0.554754 1.920975
C 0.978198 -0.744726 2.152990
C 0.163048 -1.839367 1.865589
C -1.112204 -1.674907 1.308527
C -2.775701 0.060404 0.334220
C -2.878803 1.465937 0.303892
H 1.167540 1.403824 2.128171
H 1.985788 -0.890935 2.526571
H 0.528920 -2.843483 2.056941
H -1.712570 -2.548028 1.082717
S -1.524509 2.222012 0.991238
O -0.753608 1.834250 -1.740942
C -0.669526 0.612791 -1.661950
C 0.604474 -0.063195 -1.383858
C -1.897209 -0.227312 -1.831130
C 0.650334 -1.412761 -1.352694
H 1.474285 0.566301 -1.223529
C -1.765890 -1.621681 -1.883829
H -2.782540 0.274740 -2.213238
C -0.542147 -2.193625 -1.599673
H 1.580145 -1.934901 -1.150796
H -2.626795 -2.243112 -2.110140
H -0.446531 -3.275937 -1.611857
C -4.012603 -0.758779 0.056645
O -4.784728 -0.393479 -0.810611
C -4.269261 -1.994366 0.870553
H -3.915634 -1.893569 1.898760
H -5.339180 -2.206547 0.849711
H -3.744632 -2.837768 0.405099
C 3.940350 -0.093987 0.882769
O 3.592677 0.878077 1.574041
O 3.999661 -1.310105 1.138262
C 4.425759 0.253977 -0.562918
F 4.132965 1.507317 -0.951370
F 5.768089 0.123861 -0.663734
F 3.893858 -0.567938 -1.494338
C -3.964586 2.302860 -0.282311
H -4.943957 1.946197 0.045794
H -3.845137 3.351222 -0.002709
H -3.937145 2.226453 -1.374876
42
TS-IV_16 Eopt -
1731.134406
C -1.427192 -0.184789 -1.021895
C -2.812120 -0.445489 -1.041188
C -3.337749 -1.686563 -1.395591
C -2.441094 -2.690316 -1.761199
C -1.065402 -2.446586 -1.763098
C -0.543130 -1.203284 -1.390363
C -1.144355 1.138259 -0.462702
C -2.321670 1.865642 -0.213277
H -4.408248 -1.866492 -1.388783
H -2.819582 -3.666598 -2.045336
H -0.380241 -3.238793 -2.048821
H 0.532873 -1.061640 -1.370715
S -3.721657 0.950829 -0.522798
O -3.056098 0.283125 2.216111
C -2.023682 -0.323170 1.950793
C -1.994632 -1.785399 1.807449
C -0.745695 0.433779 1.750112
C -0.821610 -2.406584 1.560583
H -2.935670 -2.314278 1.921280
C 0.449239 -0.275973 1.589180
H -0.749156 1.482891 2.034808
C 0.408225 -1.648837 1.448176
H -0.771805 -3.485277 1.452986
H 1.398627 0.249149 1.545672
H 1.346090 -2.177742 1.294527
C 0.158802 1.897054 -0.579792
O 0.402349 2.784213 0.217215
C 1.082883 1.552808 -1.708236
H 0.527625 1.326273 -2.622836
H 1.770154 2.385183 -1.866814
H 1.665453 0.660004 -1.442835
C 3.409585 -1.011538 0.121615
O 3.568129 -1.515850 1.244965
O 2.625026 -1.303697 -0.801715
C 4.297732 0.245254 -0.144214
F 5.538130 0.125361 0.359313
F 4.433610 0.540400 -1.448349
F 3.746108 1.336151 0.441570
C -2.455628 3.254793 0.311305
H -1.775311 3.931064 -0.211656
H -3.480265 3.616552 0.208768
H -2.184965 3.272525 1.373161
42
TS-IV_17 Eopt -
1731.128972
C -1.391950 -0.098190 1.116423
C -0.522347 1.009942 1.043198
C 0.805223 0.959701 1.460427
C 1.267398 -0.241501 2.000487
C 0.416399 -1.342175 2.114372
C -0.910607 -1.286954 1.671325
C -2.663173 0.185425 0.445353
C -2.752558 1.528533 0.034730
H 1.471395 1.811869 1.360478
H 2.298719 -0.308480 2.332742
H 0.789953 -2.265469 2.546064
H -1.540624 -2.163353 1.764603
S -1.312693 2.383291 0.317152
O -0.852749 1.213564 -2.238428
C -0.796693 0.052094 -1.846727
C 0.480379 -0.603989 -1.530254
C -2.064191 -0.722940 -1.665308
C 0.476130 -1.907233 -1.168996
H 1.385340 -0.000527 -1.594353
C -1.992091 -2.088143 -1.365773
H -2.973256 -0.274569 -2.057817
C -0.760591 -2.648494 -1.086997
H 1.400558 -2.424459 -0.935647
H -2.894666 -2.689777 -1.329629
H -0.705569 -3.704353 -0.835268
C -3.929065 -0.629175 0.539150
O -4.786647 -0.496835 -0.314852
C -4.110210 -1.571345 1.694896
H -3.619933 -1.213563 2.602343
H -5.179094 -1.711676 1.862900
H -3.678006 -2.543532 1.426698
C 3.638882 1.092300 0.030899
O 2.935549 1.420978 -0.942587
O 3.875678 1.663904 1.106677
C 4.347121 -0.286822 -0.167312
F 5.348352 -0.195434 -1.070364
F 4.885645 -0.792050 0.955031
F 3.496040 -1.226163 -0.638373
C -3.893982 2.218126 -0.633501
H -4.830165 2.004417 -0.112149
H -3.734289 3.297246 -0.667030
H -3.996697 1.848539 -1.659853
42
TS-IV_18 Eopt -
1731.134612
C -0.941373 -0.202618 -0.911596
C -1.912145 -1.120058 -1.358250
C -1.601042 -2.434181 -1.701861
C -0.267190 -2.831382 -1.608091
C 0.714010 -1.932236 -1.182479
C 0.389407 -0.619335 -0.825255

C -1.553801 1.049989 -0.465810
C -2.938484 1.075865 -0.730712
H -2.369363 -3.125880 -2.032666
H 0.006978 -3.847621 -1.870784
H 1.748656 -2.255879 -1.117843
H 1.164158 0.055770 -0.480165
S -3.500548 -0.395616 -1.366086
O -3.620513 -0.741558 1.534777
C -2.404921 -0.758387 1.692688
C -1.646915 -2.014678 1.772874
C -1.648269 0.534339 1.793369
C -0.315723 -1.974728 1.989814
H -2.204391 -2.939310 1.660584
C -0.281503 0.503188 2.106572
H -2.244515 1.432162 1.936632
C 0.374091 -0.708593 2.148751
H 0.269040 -2.887093 2.052768
H 0.275784 1.422812 2.255429
H 1.446666 -0.725308 2.332052
C -0.837313 2.365568 -0.247129
O -1.384459 3.232245 0.409832
C 0.513787 2.564867 -0.863771
H 0.596211 2.072984 -1.836218
H 0.697623 3.636481 -0.956146
H 1.277087 2.137988 -0.195932
C 3.411100 0.061688 0.859207
O 2.707667 1.060784 1.112035
O 3.538092 -1.017692 1.457224
C 4.241582 0.222221 -0.453722
F 4.977227 -0.854206 -0.770530
F 3.431421 0.460936 -1.511640
F 5.090602 1.267477 -0.379840
C -3.903480 2.184628 -0.483967
H -3.525602 3.118841 -0.907658
H -4.880936 1.958661 -0.913646
H -4.019069 2.340224 0.594308
35
TS-IV_2-notfa Eopt -
1204.920832
C -0.257700 -0.009458 -1.045868
C -0.789558 1.289775 -0.913409
C -2.126826 1.584768 -1.172876
C -2.951967 0.543275 -1.597972
C -2.441111 -0.746639 -1.755860
C -1.099963 -1.037131 -1.479296
C 1.119789 -0.069066 -0.556755
C 1.609709 1.199792 -0.205763
H -2.513695 2.591841 -1.050341
H -3.997115 0.742278 -1.809720
H -3.094490 -1.543961 -2.095893
H -0.733242 -2.048756 -1.608694
S 0.421771 2.409035 -0.348362
O 0.015813 1.413661 2.312783
C -0.410350 0.312329 1.981677
C 0.548289 -0.791835 1.665964
C -1.850618 0.040740 1.866871
C 0.051137 -2.070422 1.401195
H 1.592725 -0.624226 1.917297
C -2.270251 -1.196960 1.525623
H -2.529353 0.860865 2.078878
C -1.314314 -2.252932 1.282483
H 0.731071 -2.903936 1.256645
H -3.327120 -1.426022 1.437301
H -1.697348 -3.242974 1.049894
C 2.105742 -1.190618 -0.778546
O 3.048143 -1.312456 -0.018338
C 1.912208 -2.106673 -1.953584
H 1.285068 -2.951380 -1.642433
H 1.426822 -1.606849 -2.794337
H 2.885549 -2.501047 -2.249856
C 2.963570 1.562294 0.302710
H 3.072397 1.206707 1.333678
H 3.741641 1.082180 -0.295188
H 3.110377 2.643635 0.291432
42
TS-IV_2 Eopt -
1731.129568
C 1.395314 0.049567 -1.076048
C 0.741741 1.281949 -0.874871
C -0.610441 1.473974 -1.152436
C -1.324424 0.395135 -1.671506
C -0.694115 -0.829572 -1.899497
C 0.659958 -1.015683 -1.604285
C 2.760009 0.080006 -0.541107
C 3.120796 1.371147 -0.103328
H -1.093842 2.428476 -0.968087
H -2.377620 0.511162 -1.902694
H -1.267243 -1.657886 -2.303564
H 1.116890 -1.980402 -1.787896
S 1.830314 2.468040 -0.202904
O 1.512750 1.224282 2.388205
C 1.199133 0.123334 1.947762
C -0.199989 -0.283745 1.775461
C 2.275493 -0.842485 1.545458
C -0.484201 -1.528243 1.332955
H -0.983926 0.429510 2.010930
C 1.918003 -2.153009 1.194746
H 3.289383 -0.594438 1.850708
C 0.581824 -2.466096 1.049496
H -1.518026 -1.832612 1.182940
H 2.685591 -2.897964 1.010308
H 0.305407 -3.474722 0.752991
C 3.860335 -0.912191 -0.834746
O 4.839547 -0.945055 -0.112586
C 3.725606 -1.821008 -2.022525
H 3.210315 -1.337398 -2.855078
H 4.721525 -2.149818 -2.322839
H 3.147581 -2.705396 -1.726105
C -3.746906 -0.564860 0.604007
O -3.571619 -1.726986 0.199265
O -3.244371 0.059653 1.555421
C -4.817854 0.259315 -0.181969
F -4.434965 1.535272 -0.395843
F -5.121779 -0.254369 -1.387087
F -5.976344 0.320125 0.513975
C 4.424636 1.827951 0.456079
H 5.249662 1.516409 -0.189351
H 4.442388 2.912722 0.573887
H 4.584710 1.366367 1.436617
42
TS-IV_3 Eopt -
1731.131616
C -1.179752 -0.323683 -0.957281
C -2.540698 -0.445560 -1.290869
C -3.120688 -1.663246 -1.640084
C -2.293729 -2.786895 -1.667856
C -0.935299 -2.680279 -1.357760
C -0.360317 -1.455553 -0.997264
C -0.863458 1.023722 -0.480057
C -1.960504 1.897651 -0.616163
H -4.175796 -1.735753 -1.884876
H -2.713333 -3.749999 -1.939199
H -0.307851 -3.565620 -1.393195
H 0.696211 -1.388700 -0.752660
S -3.353829 1.094821 -1.163861
O -3.459472 0.717997 1.707304
C -2.474169 -0.011372 1.759701
C -2.586076 -1.474658 1.784075
C -1.102330 0.593960 1.793526
C -1.467335 -2.224366 1.878223
H -3.583983 -1.898574 1.733078
C 0.014253 -0.235351 1.982034
H -1.068655 1.657941 2.013066
C -0.160113 -1.601568 1.956263
H -1.522960 -3.308163 1.898516
H 1.017203 0.179747 2.074507
H 0.719788 -2.233282 2.033053
C 0.572016 1.501996 -0.427836
O 1.388952 0.878851 -1.076052
C 0.987018 2.703894 0.375354
H 0.246528 3.038574 1.100524
H 1.921450 2.440408 0.880790
H 1.194341 3.524612 -0.320409
C 3.179599 -0.393365 0.711734
O 3.123135 0.485562 1.592374
O 2.603586 -1.492073 0.618213
C 4.196768 -0.097314 -0.433306
F 5.407347 -0.623687 -0.109310
F 3.854487 -0.641843 -1.612324
F 4.403964 1.212453 -0.649807
C -2.041829 3.354489 -0.302043
H -1.230593 3.903522 -0.786660
H -2.994057 3.774694 -0.630124
H -1.958970 3.503445 0.781277
42
TS-IV_4 Eopt -
1731.132329
C -2.099187 -0.195137 0.963847
C -3.070090 -0.975329 0.305942
C -4.424296 -0.648741 0.312555
C -4.815838 0.483787 1.026909
C -3.871964 1.249972 1.716016
C -2.512478 0.925345 1.691386
C -0.743716 -0.692878 0.705960
C -0.757495 -1.872308 -0.057058
H -5.151849 -1.257084 -0.215849
H -5.864129 0.762346 1.052525
H -4.197133 2.119338 2.279040
H -1.788144 1.527560 2.226151
S -2.332285 -2.319082 -0.526802
O -1.689774 -0.546228 -2.634042
C -1.595895 0.464243 -1.942599
C -2.699296 1.426552 -1.821648
C -0.327699 0.736785 -1.200171
C -2.518582 2.551105 -1.096480
H -3.624933 1.194035 -2.338692
C -0.171855 1.962460 -0.539204
H 0.540572 0.131840 -1.443029
C -1.248707 2.818383 -0.452315
H -3.313460 3.282797 -0.992900
H 0.782369 2.202051 -0.086108
H -1.136110 3.760688 0.077064
C 0.396088 -0.264887 1.610577
O 0.387915 0.867171 2.054381
C 1.392644 -1.290859 2.066803
H 1.992954 -1.665836 1.235419
H 2.048945 -0.826960 2.804826
H 0.864205 -2.138144 2.518831

C 3.469238 -0.393591 -0.364147
O 2.407504 0.260947 -0.386311
O 3.670607 -1.612851 -0.471529
C 4.732992 0.474450 -0.080183
F 4.833650 0.726732 1.246172
F 5.882894 -0.113209 -0.448475
F 4.693656 1.670684 -0.696148
C 0.396122 -2.690345 -0.536743
H 0.681269 -3.425961 0.223790
H 0.133956 -3.230214 -1.449664
H 1.266304 -2.055344 -0.733172
42
TS-IV_5 Eopt -
1731.133473
C -2.238728 0.700450 0.439621
C -2.322325 1.485618 -0.723768
C -2.208440 2.874393 -0.714477
C -2.024493 3.496601 0.517145
C -1.975258 2.740119 1.693018
C -2.081125 1.349475 1.670369
C -2.243188 -0.739927 0.123489
C -2.485607 -0.956845 -1.258125
H -2.263090 3.449967 -1.633097
H -1.930372 4.576578 0.562358
H -1.848955 3.243790 2.646335
H -2.045043 0.782060 2.590191
S -2.556778 0.488090 -2.136891
O 0.248066 -0.143133 -1.998327
C 0.401892 -0.027184 -0.791235
C 0.972803 1.179614 -0.175695
C -0.037078 -1.134749 0.131798
C 1.139640 1.236305 1.161508
H 1.249139 1.992046 -0.840631
C 0.246579 -1.039216 1.519207
H -0.159039 -2.104821 -0.340449
C 0.788827 0.111351 2.013864
H 1.567743 2.117915 1.628515
H 0.051778 -1.886532 2.168537
H 1.020665 0.182986 3.072069
C -2.589874 -1.755664 1.189819
O -2.883205 -1.347011 2.297210
C -2.579293 -3.232421 0.901469
H -1.762329 -3.541368 0.247005
H -2.511733 -3.758759 1.854455
H -3.523382 -3.511427 0.420775
C 3.317206 -0.947669 0.389549
O 2.550552 -1.650439 -0.300336
O 3.561603 -0.958243 1.605163
C 4.140003 0.107225 -0.418569
F 4.298772 1.268798 0.244775
F 5.384506 -0.363568 -0.667496
F 3.600549 0.413869 -1.610710
C -2.616879 -2.236143 -2.016199
H -3.479698 -2.809388 -1.668051
H -2.736791 -2.043896 -3.083704
H -1.718978 -2.847877 -1.879586
42
TS-IV_6 Eopt -
1731.128634
C 1.323277 -0.123495 -1.120314
C 0.394883 0.927443 -0.994220
C -0.952449 0.800653 -1.326119
C -1.376259 -0.427887 -1.830559
C -0.467773 -1.474512 -1.998631
C 0.880248 -1.339858 -1.648510
C 2.630153 0.239130 -0.541969
C 2.656199 1.606357 -0.156147
H -1.647498 1.622841 -1.189418
H -2.417668 -0.568577 -2.100060
H -0.813750 -2.419807 -2.404966
H 1.567202 -2.162187 -1.791249
S 1.151887 2.354436 -0.341100
O 1.112508 1.175866 2.300046
C 1.062420 0.027312 1.874131
C -0.207326 -0.685752 1.688967
C 2.337595 -0.679063 1.508723
C -0.189054 -1.980137 1.305661
H -1.131877 -0.136520 1.859555
C 2.289342 -2.060087 1.224900
H 3.248753 -0.218428 1.883761
C 1.066076 -2.672681 1.087591
H -1.125198 -2.510920 1.163701
H 3.206950 -2.623206 1.092764
H 1.029940 -3.732522 0.849929
C 3.868118 -0.566956 -0.863508
O 3.738766 -1.580544 -1.523732
C 5.225585 -0.134842 -0.384134
H 5.223252 0.161445 0.666639
H 5.914601 -0.966593 -0.534425
H 5.569817 0.717867 -0.978632
C -3.497892 -0.085295 0.854466
O -3.009806 0.941884 1.361299
O -3.262896 -1.289287 1.049671
C -4.621155 0.223950 -0.188506
F -4.238201 1.159431 -1.085867
F -5.017339 -0.845097 -0.900238
F -5.724848 0.711410 0.420809
C 3.751830 2.418004 0.454447
H 4.588609 2.536507 -0.238415
H 3.387904 3.410180 0.727596
H 4.120780 1.930200 1.362593
42
TS-IV_7 Eopt -
1731.131521
C -1.700855 0.580531 -0.950933
C -0.390090 0.559301 -1.470195
C 0.303742 1.714391 -1.820249
C -0.360637 2.934902 -1.677784
C -1.671580 2.976986 -1.204827
C -2.352612 1.811024 -0.831200
C -2.124383 -0.759204 -0.517136
C -1.177963 -1.740714 -0.893053
H 1.330002 1.650272 -2.165749
H 0.152794 3.853563 -1.942181
H -2.178515 3.932855 -1.114115
H -3.369616 1.863437 -0.468078
S 0.225609 -1.071192 -1.559111
O 0.819038 -0.874607 1.137389
C -0.052809 -0.065532 1.450464
C 0.218261 1.372795 1.541678
C -1.437247 -0.549864 1.731839
C -0.771047 2.207192 1.933666
H 1.221520 1.699816 1.276972
C -2.394558 0.356450 2.216482
H -1.541396 -1.622680 1.877349
C -2.083154 1.698290 2.263311
H -0.597813 3.275703 2.012153
H -3.374649 0.006614 2.523902
H -2.832870 2.403500 2.611453
C -3.561463 -1.050440 -0.167809
O -4.377703 -0.153885 -0.282212
C -3.987207 -2.408228 0.319948
H -3.295974 -2.826799 1.054141
H -4.982696 -2.313285 0.754858
H -4.039515 -3.101619 -0.525956
C 3.161018 0.475081 -0.495518
O 2.898458 0.027815 -1.628862
O 3.014919 1.610798 -0.012625
C 3.856911 -0.552822 0.451097
F 5.197717 -0.533463 0.232932
F 3.685688 -0.287537 1.756913
F 3.462565 -1.821129 0.251198
C -1.208885 -3.222385 -0.696864
H -2.040458 -3.673897 -1.243920
H -0.279651 -3.676113 -1.046592
H -1.324986 -3.460631 0.365111
42
TS-IV_8 Eopt -
1731.131805
C -1.724107 0.490064 -0.943674
C -0.406162 0.497987 -1.451094
C 0.251713 1.670478 -1.816604
C -0.450756 2.872363 -1.701202
C -1.762992 2.885183 -1.229655
C -2.407085 1.704420 -0.838481
C -2.103426 -0.848926 -0.487361
C -1.125582 -1.804636 -0.807645
H 1.282034 1.633234 -2.154953
H 0.035814 3.801252 -1.979818
H -2.297189 3.827082 -1.151526
H -3.422831 1.751618 -0.465933
S 0.266725 -1.111503 -1.490217
O 0.834626 -0.830208 1.202422
C -0.041051 -0.018831 1.499224
C 0.224053 1.424488 1.529360
C -1.417866 -0.499078 1.812228
C -0.769053 2.272155 1.885502
H 1.226596 1.743410 1.251651
C -2.379878 0.418702 2.239883
H -1.550857 -1.570216 1.940751
C -2.075012 1.768895 2.229012
H -0.601448 3.343758 1.918408
H -3.362483 0.078046 2.550433
H -2.833631 2.483344 2.537421
C -3.481427 -1.311156 -0.093861
O -3.602003 -2.320297 0.577765
C -4.680114 -0.528737 -0.551238
H -4.532914 -0.088000 -1.539631
H -5.547055 -1.191033 -0.549706
H -4.866700 0.281145 0.165085
C 3.164097 0.526933 -0.536729
O 2.922029 0.091217 -1.679029
O 2.990990 1.653207 -0.040489
C 3.864744 -0.505048 0.401729
F 5.201545 -0.502636 0.160552
F 3.718509 -0.234996 1.709200
F 3.450828 -1.768555 0.210334
C -1.150040 -3.275034 -0.550602
H -2.098537 -3.708209 -0.876934
H -0.326417 -3.773994 -1.064800
H -1.055384 -3.464404 0.524150
42
TS-IV_9 Eopt -
1731.134133
C -2.252323 0.667400 0.409606
C -2.336165 1.451821 -0.756476
C -2.231389 2.841312 -0.743784
C -2.056774 3.465328 0.488179

C -2.000032 2.709955 1.664611
C -2.092964 1.319084 1.638445
C -2.238660 -0.767212 0.090078
C -2.467006 -0.990231 -1.287910
H -2.284542 3.417261 -1.662292
H -1.970756 4.545895 0.533494
H -1.873683 3.213569 2.617789
H -2.030478 0.762612 2.565191
S -2.552453 0.453157 -2.173401
O 0.270958 -0.183545 -2.013187
C 0.426842 -0.048289 -0.808970
C 0.987991 1.175665 -0.215897
C -0.007398 -1.139722 0.134969
C 1.144374 1.264507 1.120772
H 1.264510 1.974267 -0.897345
C 0.253092 -1.006103 1.521311
H -0.170606 -2.120108 -0.303594
C 0.790668 0.157521 1.994037
H 1.564383 2.158443 1.571261
H 0.049903 -1.837668 2.188510
H 1.009763 0.255681 3.052998
C -2.556871 -1.893894 1.045298
O -2.289999 -3.037717 0.727129
C -3.207702 -1.575334 2.361146
H -3.924145 -0.755086 2.278762
H -3.695159 -2.477626 2.732907
H -2.430249 -1.284674 3.078230
C 3.318336 -0.927222 0.423667
O 2.559959 -1.635527 -0.270448
O 3.537197 -0.921268 1.644036
C 4.163149 0.111216 -0.382630
F 4.311130 1.282190 0.266819
F 5.410682 -0.367537 -0.596432
F 3.650739 0.401381 -1.590539
C -2.599750 -2.289123 -2.006125
H -3.331010 -2.928804 -1.504898
H -2.899117 -2.133684 -3.044074
H -1.641944 -2.820513 -1.993344
35
TS-I_1-notfa Eopt -
1204.913077
C -0.011270 -0.151536 1.167804
C -0.598147 -1.378937 0.797614
C -1.878666 -1.761268 1.190236
C -2.587911 -0.878481 2.008239
C -2.022023 0.338212 2.387036
C -0.733475 0.715604 1.976217
C 1.346817 -0.013114 0.605291
C 1.726052 -1.062464 -0.170604
H -2.309044 -2.707046 0.874972
H -3.585191 -1.142653 2.342565
H -2.588325 1.014523 3.019542
H -0.314983 1.667327 2.280353
S 0.405543 -2.210546 -0.334755
O -0.324145 -1.296700 -2.050426
C -0.804977 -0.112643 -1.793042
C 0.044267 1.034370 -1.856892
C -2.173735 0.029826 -1.415192
C -0.469146 2.279073 -1.539309
H 1.071234 0.901967 -2.184726
C -2.660933 1.278246 -1.093866
H -2.801260 -0.856189 -1.403449
C -1.804148 2.394169 -1.131531
H 0.161770 3.159698 -1.586938
H -3.699283 1.404724 -0.805789
H -2.194133 3.370688 -0.860346
C 2.194600 1.195494 0.894210
O 1.636639 2.262566 1.067614
C 3.688555 1.054794 0.979854
H 3.994994 0.043311 1.252428
H 4.127564 1.303491 0.007320
H 4.060479 1.775821 1.710200
C 2.956238 -1.317024 -0.981053
H 3.455735 -0.381071 -1.232108
H 3.656054 -1.953559 -0.429409
H 2.703171 -1.825140 -1.916105
42
TS-I_1 Eopt -
1731.125558
C -0.400107 -0.087660 0.630117
C 0.191031 -0.615101 -0.538375
C 0.689815 -1.915145 -0.612954
C 0.611110 -2.705319 0.535874
C 0.023964 -2.202930 1.696876
C -0.490265 -0.898554 1.755525
C -0.884297 1.282228 0.404770
C -0.642269 1.740102 -0.858179
H 1.142513 -2.286757 -1.526292
H 1.006670 -3.715054 0.518908
H -0.035750 -2.831031 2.580266
H -0.947079 -0.541852 2.671555
S 0.073707 0.501770 -1.843991
O -2.045008 -0.172566 -2.382797
C -2.595175 -0.685769 -1.353290
C -3.338153 0.143383 -0.432337
C -2.443705 -2.090034 -1.071059
C -3.890299 -0.419518 0.703842
H -3.476756 1.191898 -0.680578
C -2.988400 -2.617493 0.072589
H -1.902867 -2.696775 -1.790992
C -3.687672 -1.779308 0.970087
H -4.460733 0.191188 1.395967
H -2.880580 -3.673032 0.299829
H -4.103933 -2.210182 1.876201
C -1.498570 2.169985 1.447836
O -2.404486 2.921021 1.134398
C -0.956939 2.122725 2.850445
H -1.572991 1.436547 3.443700
H 0.079562 1.780618 2.882701
H -1.045068 3.118169 3.289851
C 3.106410 0.382236 -0.856625
O 2.580861 1.505506 -0.728273
O 3.136719 -0.399338 -1.819671
C 3.793741 -0.119998 0.452631
F 2.902404 -0.200797 1.466399
F 4.356366 -1.334339 0.341815
F 4.765575 0.721530 0.863748
C -0.964633 3.052339 -1.492585
H -1.926208 2.993729 -2.015092
H -1.038435 3.832052 -0.733646
H -0.194973 3.330612 -2.216545
35
TS-I_2-notfa Eopt -
1204.912685
C 0.007352 0.137926 -1.168181
C 0.660458 -1.111371 -1.116673
C 1.966711 -1.305614 -1.555288
C 2.635486 -0.201284 -2.090623
C 2.010036 1.043230 -2.141730
C 0.696762 1.228075 -1.681248
C -1.342397 0.059801 -0.582091
C -1.671532 -1.175072 -0.120690
H 2.447428 -2.276516 -1.486280
H 3.650143 -0.315452 -2.455875
H 2.548264 1.894464 -2.546238
H 0.244820 2.212966 -1.722261
S -0.301802 -2.262767 -0.256033
O 0.372514 -1.775840 1.650318
C 0.778132 -0.539642 1.714153
C -0.146168 0.503068 2.034611
C 2.141767 -0.227668 1.433268
C 0.298257 1.814361 2.077514
H -1.174698 0.238743 2.263896
C 2.558096 1.084801 1.472794
H 2.821347 -1.044799 1.211483
C 1.633555 2.103710 1.774116
H -0.388557 2.614481 2.332344
H 3.593107 1.338790 1.269268
H 1.971345 3.135590 1.792534
C -2.327885 1.197862 -0.529213
O -2.994428 1.352870 0.475226
C -2.468046 2.083807 -1.735236
H -1.890716 3.000651 -1.568268
H -2.109284 1.601779 -2.646629
H -3.518130 2.365008 -1.839047
C -2.893997 -1.666064 0.579462
H -2.742329 -1.628181 1.664597
H -3.746630 -1.031889 0.333698
H -3.121485 -2.697168 0.299295
42
TS-I_2 Eopt -
1731.125244
C -0.412200 -0.176485 0.650745
C 0.194356 -0.652939 -0.531769
C 0.667832 -1.957510 -0.677148
C 0.549128 -2.813195 0.418496
C -0.043069 -2.360582 1.597044
C -0.528487 -1.049473 1.728397
C -0.859079 1.223289 0.494880
C -0.558554 1.741938 -0.736386
H 1.132158 -2.279997 -1.603242
H 0.921984 -3.829352 0.349324
H -0.127061 -3.034798 2.443951
H -0.975326 -0.722971 2.657560
S 0.141853 0.535069 -1.769533
O -1.988640 -0.029493 -2.394279
C -2.575104 -0.588480 -1.409207
C -2.452460 -2.007097 -1.195249
C -3.333405 0.201897 -0.467398
C -3.026217 -2.581464 -0.088149
H -1.906850 -2.586688 -1.933558
C -3.913431 -0.405152 0.630429
H -3.463282 1.259624 -0.679028
C -3.727761 -1.778473 0.838099
H -2.936246 -3.648152 0.089409
H -4.490910 0.177696 1.340574
H -4.161928 -2.244996 1.717667
C -1.534000 1.958173 1.614779
O -1.523918 1.467948 2.731152
C -2.235673 3.262560 1.356691
H -2.847875 3.226091 0.452456
H -2.858532 3.491934 2.222065
H -1.496829 4.061430 1.234046
C 3.153431 0.403861 -0.787660
O 2.596132 1.513341 -0.668698
O 3.161008 -0.406416 -1.727316
C 3.884896 -0.062338 0.509172
F 2.979135 -0.469957 1.429563

F 4.725949 -1.091825 0.316842
F 4.601287 0.921188 1.085779
C -0.789494 3.086786 -1.353153
H -0.439100 3.882300 -0.693258
H -0.245271 3.172535 -2.295831
H -1.852850 3.244659 -1.561817
35
TS-I_3-notfa Eopt -1204.911579
C 0.008856 -0.201361 1.192428
C -0.576160 -1.413212 0.763646
C -1.861974 -1.813093 1.119033
C -2.589281 -0.970476 1.962736
C -2.026821 0.222806 2.410117
C -0.730862 0.616056 2.039124
C 1.372327 -0.044820 0.644224
C 1.754636 -1.075073 -0.161919
H -2.282110 -2.746419 0.756483
H -3.591709 -1.250141 2.267487
H -2.597851 0.867488 3.070618
H -0.329972 1.543806 2.427337
S 0.448004 -2.222836 -0.364828
O -0.278302 -1.245216 -2.099047
C -0.766695 -0.080680 -1.812689
C 0.071338 1.080255 -1.859386
C -2.142121 0.043091 -1.437721
C -0.467767 2.317733 -1.560181
H 1.103754 0.959548 -2.174980
C -2.646876 1.282540 -1.113023
H -2.758731 -0.850409 -1.441368
C -1.807471 2.412044 -1.157622
H 0.144517 3.211266 -1.619040
H -3.687661 1.395473 -0.828481
H -2.216040 3.384636 -0.899340
C 2.320843 1.089863 0.924053
O 3.517580 0.874396 0.877904
C 1.780003 2.455222 1.240633
H 0.809057 2.638129 0.776623
H 1.669588 2.544350 2.327902
H 2.507219 3.199775 0.911808
C 3.002826 -1.301974 -0.949468
H 3.455185 -0.348001 -1.223600
H 3.732068 -1.857820 -0.351731
H 2.791580 -1.873267 -1.857237
42
TS-I_4 Eopt -1731.124614
C -1.538031 -0.038952 1.113625
C -0.179718 -0.290335 0.813182
C 0.466677 -1.467580 1.186504
C -0.270243 -2.413520 1.904127
C -1.612808 -2.186637 2.198483
C -2.259349 -1.004309 1.805357
C -1.980482 1.248057 0.551431
C -0.993389 1.904655 -0.120995
H 1.504090 -1.625238 0.914265
H 0.208322 -3.334345 2.219836
H -2.176660 -2.937372 2.743631
H -3.307803 -0.862758 2.041888
S 0.465986 0.949144 -0.206223
O -0.287645 0.074451 -2.069578
C -1.289454 -0.691999 -1.824337
C -2.627557 -0.164464 -1.842514
C -1.075636 -2.070515 -1.488592
C -3.693182 -0.992942 -1.537418
H -2.770604 0.871967 -2.134844
C -2.150559 -2.867366 -1.173018
H -0.056974 -2.446025 -1.505125
C -3.452236 -2.323669 -1.174227
H -4.708333 -0.610389 -1.561527
H -2.005072 -3.911451 -0.915777
H -4.291023 -2.962645 -0.913192
C -3.340442 1.859335 0.726622
O -3.819535 2.512144 -0.183001
C -4.066144 1.669472 2.030274
H -4.744337 0.813384 1.934616
H -3.384238 1.484367 2.862620
H -4.671978 2.557047 2.222533
C 3.459035 0.006289 0.266178
O 2.840230 -0.606524 -0.628164
O 3.051801 0.504716 1.327474
C 4.978729 0.172547 -0.039059
F 5.172027 0.941725 -1.132908
F 5.673084 0.740288 0.959416
F 5.567063 -1.014221 -0.295712
C -1.027613 3.201738 -0.860656
H -1.351471 3.035832 -1.894393
H -1.735155 3.888898 -0.394008
H -0.038519 3.665186 -0.881788
42
TS-I_5 Eopt -1731.125914
C -0.951209 -0.353557 0.845958
C -0.994669 -1.529061 0.065923
C -1.754849 -2.643082 0.405629
C -2.484017 -2.588865 1.597180
C -2.459182 -1.437519 2.380524
C -1.703309 -0.312199 2.014037
C -0.119828 0.678062 0.201015
C 0.420586 0.272848 -0.986132
H -1.774828 -3.525077 -0.227543
H -3.074392 -3.445324 1.904196
H -3.037338 -1.403340 3.298563
H -1.719363 0.567020 2.646581
S -0.139644 -1.304229 -1.417017
O -1.879248 -0.411186 -2.464493
C -2.647576 0.164122 -1.610874
C -3.720319 -0.565400 -0.996790
C -2.411293 1.531345 -1.226087
C -4.496886 0.041631 -0.040472
H -3.893357 -1.586706 -1.321782
C -3.219543 2.123863 -0.268346
H -1.624860 2.083142 -1.732837
C -4.230498 1.375658 0.340973
H -5.311978 -0.497439 0.431247
H -3.056620 3.157481 0.019271
H -4.846295 1.838413 1.106794
C 0.231645 2.024251 0.771808
O 0.472482 2.937491 0.002146
C 0.261343 2.218762 2.260558
H 0.981784 3.006608 2.487535
H -0.729746 2.556298 2.588632
H 0.530011 1.301660 2.784573
C 2.879044 -0.535013 0.617786
O 2.381811 -1.600409 0.207949
O 2.458394 0.304254 1.432664
C 4.283776 -0.226165 0.007595
F 4.669778 1.051814 0.159812
F 5.233936 -0.989604 0.595317
F 4.344100 -0.492094 -1.312634
C 1.343525 0.981758 -1.921955
H 2.091167 1.542568 -1.354018
H 1.851813 0.273285 -2.579638
H 0.790033 1.698197 -2.537871
42
TS-I_6 Eopt -1731.122098
C -1.926503 -1.117476 0.459005
C -2.553324 -1.096668 -0.805250
C -3.927200 -0.956692 -0.971742
C -4.711041 -0.856859 0.181399
C -4.113301 -0.883867 1.439645
C -2.724535 -1.018470 1.592935
C -0.465945 -1.277923 0.329232
C -0.036674 -1.314433 -0.961389
H -4.374657 -0.931484 -1.960686
H -5.787115 -0.756522 0.090713
H -4.732940 -0.807743 2.327455
H -2.296492 -1.049169 2.588247
S -1.375177 -1.094964 -2.071463
O -1.209791 1.029176 -2.184368
C -1.478958 1.577274 -1.044921
C -0.434571 1.766932 -0.083695
C -2.821903 1.974005 -0.739183
C -0.736527 2.351850 1.133769
H 0.583978 1.484903 -0.338839
C -3.096607 2.534289 0.486618
H -3.585069 1.842912 -1.500066
C -2.058798 2.704614 1.427306
H 0.050895 2.518619 1.861415
H -4.105271 2.847061 0.735758
H -2.290674 3.143439 2.393652
C 0.459636 -1.553120 1.491926
O 1.250619 -2.469877 1.394360
C 0.322547 -0.747679 2.751730
H -0.208475 0.191027 2.587551
H -0.220064 -1.347460 3.491829
H 1.325282 -0.546130 3.130750
C 3.003717 0.882015 0.017144
O 2.829806 1.699664 -0.903620
O 2.268571 0.549640 0.964325
C 4.389017 0.162372 -0.048506
F 5.399052 1.030981 0.180317
F 4.528417 -0.833366 0.841517
F 4.615419 -0.375409 -1.266149
C 1.340649 -1.406479 -1.527137
H 2.045740 -1.625375 -0.725888
H 1.403110 -2.198993 -2.278631
H 1.616879 -0.456449 -1.999912
42
TS-V_1 Eopt -1731.159936
C 0.652360 0.612867 0.220002
C -0.327191 0.445415 -0.759790
C -1.289147 1.408422 -1.038482
C -1.257221 2.585772 -0.293465
C -0.293076 2.772952 0.700058
C 0.660369 1.790522 0.966456
C 1.567805 -0.581221 0.372029
C 1.237015 -1.511772 -0.784447
H -2.042050 1.248153 -1.804138
H -1.996474 3.357033 -0.483427
H -0.288774 3.690311 1.279337
H 1.388664 1.942028 1.756732
S -0.199019 -1.125754 -1.564556
O 2.743355 -0.542447 -1.980374
C 3.406743 0.044975 -1.117868
C 4.475154 0.974143 -1.412108

C 3.074192 -0.255384 0.323454
C 4.984842 1.722359 -0.403140
H 4.778943 1.101717 -2.445219
C 3.614582 0.703629 1.332255
H 3.611426 -1.200066 0.533819
C 4.543916 1.607024 0.979555
H 5.749272 2.460603 -0.629925
H 3.301464 0.587960 2.366653
H 4.988597 2.263290 1.719733
C 1.247386 -1.420620 1.677464
O 2.154212 -2.016845 2.208385
C -0.167624 -1.431196 2.156166
H -0.389990 -0.472695 2.638023
H -0.887420 -1.529025 1.333714
H -0.294474 -2.237374 2.879607
C 1.792090 -2.888529 -0.901424
H 1.210368 -3.551941 -0.248605
H 1.708076 -3.256123 -1.925678
H 2.835280 -2.927805 -0.584706
C -4.517335 0.202103 -0.164633
C -3.333400 -0.525630 0.553696
O -3.154203 -0.141834 1.720235
O -2.762611 -1.400538 -0.125137
F -5.705160 -0.229178 0.319378
F -4.554119 0.004390 -1.494825
F -4.492208 1.537490 0.022941
42
TS-V_2 Eopt -
1731.153093
C 1.120635 0.088153 0.399065
C 0.173721 -0.458074 -0.471136
C -1.127589 0.014944 -0.578958
C -1.484675 1.096127 0.226331
C -0.557096 1.660529 1.105795
C 0.742395 1.160689 1.203700
C 2.454211 -0.621500 0.374351
C 2.378911 -1.627717 -0.755013
H -1.860291 -0.422716 -1.249686
H -2.496613 1.488781 0.155618
H -0.849902 2.500177 1.727982
H 1.442282 1.602059 1.905277
S 0.840631 -1.817067 -1.390157
O 3.197316 0.012054 -2.120589
C 3.648766 0.782181 -1.271270
C 4.177155 2.098982 -1.574259
C 3.672985 0.307158 0.167349
C 4.460794 2.947379 -0.557953
H 4.253771 2.390945 -2.616002
C 3.896246 1.362530 1.203052
H 4.574624 -0.329149 0.229911
C 4.305281 2.589989 0.845987
H 4.804825 3.951904 -0.788986
H 3.804476 1.079297 2.248419
H 4.532249 3.338121 1.597718
C 2.696836 -1.494405 1.682318
O 3.832250 -1.675764 2.044902
C 1.494577 -2.042011 2.386981
H 1.022106 -1.236187 2.959335
H 0.747181 -2.417765 1.682022
H 1.810800 -2.832525 3.067515
C 3.437638 -2.622657 -1.061118
H 3.337300 -3.459916 -0.356596
H 3.324092 -3.014443 -2.073453
H 4.436851 -2.200231 -0.944129
C -6.174629 -0.040523 0.082208
C -4.895144 0.668989 -0.465817
O -4.744154 1.833351 -0.056737
O -4.180550 -0.048067 -1.188058
F -5.903760 -0.634853 1.270659
F -6.640764 -1.007514 -0.728608
F -7.199792 0.802095 0.303698
42
TS-V_3 Eopt -
1731.160890
C 0.538605 -0.644458 0.041664
C -0.249309 -0.425977 1.172975
C -1.102818 -1.386876 1.699335
C -1.154980 -2.621655 1.055286
C -0.383583 -2.863272 -0.083935
C 0.457386 -1.878039 -0.602667
C 1.357176 0.560546 -0.367980
C 1.213846 1.566434 0.764637
H -1.725308 -1.175170 2.562163
H -1.814570 -3.393942 1.437703
H -0.444758 -3.825674 -0.581427
H 1.030886 -2.076160 -1.502289
S -0.037360 1.206510 1.823909
O 2.956578 0.726957 1.709155
C 3.471301 0.102110 0.774280
C 4.618342 -0.767145 0.916698
C 2.856685 0.285172 -0.592484
C 4.959519 -1.571298 -0.119936
H 5.119882 -0.807949 1.877175
C 3.235971 -0.730721 -1.619940
H 3.298964 1.226842 -0.970206
C 4.256426 -1.574453 -1.394797
H 5.786424 -2.265661 0.002689
H 2.724741 -0.701960 -2.578239
H 4.580984 -2.269235 -2.161691
C 0.748983 1.269938 -1.643666
O 1.506642 1.768008 -2.442032
C -0.740849 1.272914 -1.790035
H -1.040824 0.311062 -2.222184
H -1.261963 1.372047 -0.830516
H -1.030640 2.073029 -2.472317
C 1.732209 2.959844 0.682478
H 1.060888 3.542376 0.038324
H 1.751941 3.425082 1.669473
H 2.734487 2.987497 0.250422
C -3.947822 -0.072598 -0.704419
C -3.651606 0.232421 0.799600
O -4.220638 -0.526396 1.598051
O -2.840475 1.162485 0.977117
F -3.849409 1.010010 -1.499421
F -5.169036 -0.593867 -0.915232
F -3.057458 -0.977485 -1.181183
42
TS-V_4 Eopt -
1731.160803
C -1.506059 1.112718 -0.357845
C -0.994064 1.930603 0.652122
C -1.215635 3.302265 0.692762
C -1.985045 3.864109 -0.325248
C -2.500803 3.066925 -1.349442
C -2.260584 1.692559 -1.375752
C -1.100281 -0.338841 -0.228427
C -0.407809 -0.465134 1.120540
H -0.804461 3.915966 1.487867
H -2.179543 4.931656 -0.319637
H -3.091772 3.519610 -2.138834
H -2.651229 1.090945 -2.190035
S -0.016217 1.021869 1.816871
O -2.300744 -0.894442 2.033769
C -2.987559 -1.213663 1.055951
C -4.407514 -1.480936 1.117851
C -2.275000 -1.337654 -0.268569
C -5.099622 -1.594558 -0.042188
H -4.888452 -1.500359 2.089461
C -3.149722 -1.347777 -1.479184
H -1.834140 -2.352687 -0.249563
C -4.477640 -1.507792 -1.355482
H -6.175618 -1.741056 -0.000944
H -2.670890 -1.318062 -2.453750
H -5.110621 -1.584826 -2.232798
C -0.027771 -0.738860 -1.323649
O -0.113866 -1.826250 -1.839088
C 1.019074 0.276559 -1.662705
H 0.556119 1.102505 -2.213311
H 1.467250 0.687910 -0.750787
H 1.785857 -0.196374 -2.275428
C 0.302616 -1.689842 1.545335
H 1.262223 -1.717376 0.997636
H 0.523047 -1.668234 2.614142
H -0.266364 -2.589523 1.300961
C 4.876833 -0.150491 -0.218394
C 3.493974 -0.625055 0.326079
O 3.085036 -1.696187 -0.156039
O 2.957707 0.167077 1.121961
F 5.582503 0.563146 0.676785
F 4.695380 0.653876 -1.294659
F 5.669748 -1.157289 -0.622378
42
TS-V_5 Eopt -
1731.159314
C 0.870973 1.043556 0.210719
C 0.095957 1.508577 -0.852942
C -0.168733 2.855100 -1.067180
C 0.376711 3.773394 -0.170911
C 1.157550 3.335474 0.899360
C 1.405169 1.975668 1.098278
C 0.995114 -0.465664 0.267042
C 0.390850 -0.976954 -1.035641
H -0.780473 3.181297 -1.902311
H 0.189326 4.833268 -0.310196
H 1.575813 4.057969 1.592701
H 1.996655 1.655630 1.947475
S -0.499228 0.195022 -1.872323
O 2.256371 -0.808578 -1.996470
C 3.050107 -0.739755 -1.051638
C 4.463941 -0.481593 -1.198403
C 2.475947 -0.960373 0.327392
C 5.189692 -0.198367 -0.088999
H 4.880398 -0.436504 -2.198530
C 3.334650 -0.501775 1.461617
H 2.455441 -2.064840 0.418279
C 4.623647 -0.186508 1.251379
H 6.240858 0.054986 -0.197339
H 2.913431 -0.512224 2.462705
H 5.267442 0.083683 2.081271
C 0.338676 -1.029818 1.597234
O 0.465165 -0.348616 2.584387
C -0.216401 -2.422435 1.640334
H -1.111977 -2.487468 1.017553
H 0.522651 -3.143932 1.276714
H -0.468755 -2.650133 2.676721
C 0.174302 -2.400094 -1.415600
H -0.828204 -2.685713 -1.074717
H 0.203224 -2.497796 -2.503138

H 0.921717 -3.061242 -0.975063
C -4.274396 -0.006036 0.170034
C -2.894751 -0.730006 0.225721
O -2.832244 -1.806681 -0.394492
O -2.020501 -0.096486 0.845614
F -4.245934 0.970981 -0.767742
F -4.599517 0.587689 1.332629
F -5.296134 -0.816311 -0.150891
42
TS-V_6 Eopt -1731.162002
C 0.772189 0.681267 0.313100
C -0.276084 0.556205 -0.601648
C -1.166524 1.590153 -0.866214
C -1.001551 2.782505 -0.163024
C 0.023337 2.921411 0.775339
C 0.912122 1.874551 1.020048
C 1.618445 -0.565342 0.446264
C 1.148153 -1.501018 -0.658904
H -1.969405 1.460064 -1.583121
H -1.684407 3.606040 -0.345999
H 0.131869 3.852146 1.322561
H 1.694908 1.991506 1.762550
S -0.315877 -1.050295 -1.356404
O 2.612548 -0.681873 -1.980006
C 3.374219 -0.106174 -1.193296
C 4.468888 0.745194 -1.602108
C 3.133991 -0.336545 0.277468
C 5.096439 1.494455 -0.662404
H 4.700282 0.819444 -2.658899
C 3.804806 0.619749 1.206624
H 3.626333 -1.306381 0.483207
C 4.756232 1.452172 0.752457
H 5.883057 2.176845 -0.972857
H 3.564578 0.558186 2.264758
H 5.293608 2.104050 1.432585
C 1.349966 -1.328103 1.802730
O 2.259982 -1.941314 2.309543
C -0.028696 -1.247057 2.376954
H -0.150612 -0.267860 2.855163
H -0.805909 -1.320615 1.606486
H -0.149145 -2.029631 3.126874
C 1.596930 -2.917902 -0.763771
H 1.033677 -3.515228 -0.035342
H 1.393251 -3.314740 -1.760100
H 2.660731 -3.022414 -0.543079
C -4.747779 0.168406 0.076487
C -3.442964 -0.614954 -0.272240
O -2.821525 -1.071325 0.701297
O -3.183876 -0.625929 -1.490878
F -5.763093 -0.117422 -0.760953
F -4.533298 1.502415 -0.022065
F -5.199001 -0.055771 1.321334
42
TS-V_7 Eopt -1731.156264
C -1.038263 1.058964 -0.296843
C -0.425826 1.774509 0.730731
C -0.380042 3.162649 0.759453
C -0.977379 3.853961 -0.293313
C -1.592564 3.160227 -1.337063
C -1.618709 1.765253 -1.348213
C -0.926396 -0.444627 -0.155507
C -0.393694 -0.679952 1.258970
H 0.109012 3.691868 1.570920
H -0.957943 4.939156 -0.299760
H -2.047878 3.708485 -2.155379
H -2.075771 1.243095 -2.181800
S 0.266231 0.716745 1.965535
O -2.300475 -0.655862 2.022049
C -3.016463 -0.849184 1.029279
C -4.456458 -0.783486 1.036047
C -2.292691 -1.181751 -0.248658
C -5.106043 -0.791172 -0.155369
H -4.968753 -0.645071 1.981462
C -3.093045 -1.048534 -1.501353
H -2.094222 -2.268124 -0.151294
C -4.427462 -0.904685 -1.437958
H -6.187345 -0.684186 -0.164199
H -2.580824 -1.149986 -2.455425
H -5.024967 -0.869055 -2.342225
C -0.032052 -1.037856 -1.323569
O 0.249533 -0.315035 -2.245591
C 0.205016 -2.521761 -1.384147
H 0.263773 -3.002153 -0.410607
H -0.624746 -2.967387 -1.947469
H 1.130129 -2.691167 -1.935172
C -0.015827 -1.993679 1.855124
H 0.997372 -2.232845 1.515860
H -0.015961 -1.918165 2.944584
H -0.703110 -2.786532 1.555095
C 4.414593 -0.292306 -0.174293
C 2.883718 0.012828 -0.119960
O 2.559712 1.202095 -0.206726
O 2.195896 -1.023039 0.019686
F 4.716716 -1.148858 -1.173869
F 4.839401 -0.875638 0.969312
F 5.179629 0.795884 -0.356402
42
TS-V_8 Eopt -1731.153949
C 1.207282 0.773319 0.663875
C -0.120321 0.814438 0.235013
C -0.905886 1.956263 0.330836
C -0.325131 3.094484 0.889192
C 0.993870 3.071137 1.345646
C 1.763513 1.911690 1.242208
C 1.864665 -0.580388 0.497931
C 0.892874 -1.399704 -0.344564
H -1.930094 1.943425 -0.023926
H -0.911554 4.003834 0.973712
H 1.428235 3.961080 1.789637
H 2.779539 1.903994 1.622447
S -0.656719 -0.743432 -0.427819
O 1.825415 -0.702393 -2.105103
C 2.898199 -0.254289 -1.684755
C 3.838587 0.486485 -2.494020
C 3.226017 -0.532027 -0.233711
C 4.873223 1.111422 -1.880783
H 3.639549 0.587578 -3.555172
C 4.320724 0.302909 0.349379
H 3.637326 -1.559362 -0.266256
C 5.112112 1.040533 -0.446180
H 5.551588 1.715410 -2.477229
H 4.504317 0.234073 1.418862
H 5.944967 1.599685 -0.033932
C 1.933983 -1.314428 1.898214
O 1.152560 -0.983505 2.754871
C 2.962996 -2.389106 2.110249
H 3.030445 -3.073303 1.261144
H 3.946445 -1.923317 2.240439
H 2.709297 -2.938918 3.016891
C 1.069192 -2.852977 -0.631014
H 0.787389 -3.423309 0.263346
H 0.421482 -3.164180 -1.452721
H 2.105415 -3.088255 -0.882555
C -5.329704 -0.129652 -0.258426
C -3.786263 0.084785 -0.175103
O -3.257687 -0.174163 0.918011
O -3.288264 0.501026 -1.239479
F -5.659173 -0.982454 -1.252238
F -5.972073 1.029763 -0.520851
F -5.872381 -0.622932 0.866433
42
TS-V_9 Eopt -1731.158408
C 1.011027 -0.785695 -0.545435
C 2.115768 -1.567686 -0.890773
C 2.115853 -2.459046 -1.956793
C 0.944192 -2.558143 -2.705822
C -0.173641 -1.784477 -2.383201
C -0.151229 -0.898628 -1.305576
C 1.231677 0.069592 0.679156
C 2.692288 -0.063746 1.033596
H 2.992745 -3.050629 -2.199582
H 0.906089 -3.241199 -3.548075
H -1.079398 -1.869167 -2.974999
H -1.038423 -0.314710 -1.080713
S 3.488255 -1.270278 0.187136
O 3.046419 1.729086 -0.399482
C 1.890890 2.155462 -0.459724
C 1.456394 3.186493 -1.381900
C 0.873041 1.564593 0.497072
C 0.132230 3.442473 -1.515996
H 2.207916 3.666414 -1.999556
C -0.560514 1.863733 0.198756
H 1.073362 2.061408 1.463264
C -0.894579 2.770067 -0.732258
H -0.191008 4.173707 -2.252156
H -1.323072 1.386424 0.808014
H -1.940903 2.998490 -0.908008
C 0.451864 -0.491922 1.955889
O 0.136033 0.290723 2.814157
C 0.186975 -1.964392 2.010594
H 1.104399 -2.529028 1.810946
H -0.206281 -2.217567 2.995020
H -0.538941 -2.238882 1.239219
C -3.532560 0.537087 -0.511071
O -3.815831 1.603673 0.061764
O -3.237991 0.281337 -1.690113
C -3.520812 -0.688535 0.464291
F -3.260624 -1.864658 -0.132245
F -2.571828 -0.533516 1.423024
F -4.692431 -0.833618 1.113393
C 3.316619 0.551669 2.229540
H 3.093238 -0.083996 3.097853
H 4.400661 0.612331 2.119663
H 2.909522 1.545039 2.424710
20
thiophene-Ac-opt_NBO Eopt -859.350871
C 0.322247 -0.381655 -0.000140
C 1.256527 0.675759 0.000047
C 2.637712 0.454586 0.000134
C 3.087300 -0.857367 0.000047
C 2.174753 -1.927150 -0.000122
C 0.806083 -1.704372 -0.000222
C -1.044411 0.118602 -0.000221

C -1.090427 1.482710 -0.000127
H 3.336189 1.285993 0.000253
H 4.154143 -1.058171 0.000110
H 2.548770 -2.946365 -0.000179
H 0.106775 -2.531437 -0.000323
H -1.974281 2.109380 -0.000106
S 0.461679 2.234307 0.000080
C -2.263952 -0.726406 -0.000093
O -2.176394 -1.945580 0.000477
C -3.607657 -0.040788 -0.000153
H -3.712993 0.592589 0.886220
H -3.711113 0.597955 -0.882823
H -4.392265 -0.797736 -0.003134
23
thiophene-AcMe-opt_NBO Eopt
-898.664378
C -0.602824 -0.440960 -0.009084
C -1.316221 0.774796 0.002551
C -2.712794 0.830476 0.042167
C -3.413503 -0.366071 0.073034
C -2.726447 -1.591822 0.067822
C -1.340553 -1.640795 0.026472
C 0.841024 -0.223344 -0.025649
C 1.172879 1.109902 -0.036913
H -3.232086 1.783918 0.050085
H -4.498607 -0.352970 0.102791
H -3.290217 -2.519344 0.094542
H -0.823725 -2.591770 0.015304
S -0.231306 2.138249 -0.032726
C 1.807322 -1.353764 -0.047251
O 1.431676 -2.469088 -0.382583
C 3.241324 -1.139011 0.367371
H 3.788305 -0.610665 -0.419817
H 3.309533 -0.546776 1.283334
H 3.703084 -2.115698 0.518590
C 2.506389 1.798753 -0.086932
H 3.063274 1.648332 0.841969
H 3.110679 1.422513 -0.915861
H 2.377674 2.874219 -0.228200
17
thiophene-COHH-opt_NBO Eopt
-820.028019
C 0.022221 -0.386396 0.000119
C -0.912837 0.669227 -0.000113
C -2.292325 0.440082 -0.000311
C -2.732688 -0.875817 -0.000083
C -1.815980 -1.942163 0.000193
C -0.448414 -1.711821 0.000225
C 1.383178 0.118725 0.000204
C 1.434343 1.483055 -0.000164
H -2.996998 1.266164 -0.000489
H -3.798366 -1.082602 -0.000102
H -2.185961 -2.962745 0.000355
H 0.257681 -2.534347 0.000255
S -0.117159 2.231087 0.000100
C 2.615500 -0.673159 0.000123
O 2.650143 -1.891078 -0.000418
H 3.549695 -0.081840 0.000648
H 2.329373 2.096206 -0.000077
20
thiophene-COHMe-opt_NBO Eopt
-859.344906
C -0.451529 0.444005 -0.004844
C -0.848453 -0.908160 0.001808
C -2.191850 -1.292425 0.006468
C -3.155208 -0.292604 0.004830
C -2.781632 1.061853 -0.001893
C -1.445823 1.438775 -0.006915
C 0.996201 0.572951 -0.010027
C 1.646410 -0.637353 -0.010716
H -2.473847 -2.340831 0.011148
H -4.206640 -0.562459 0.008503
H -3.551650 1.827285 -0.003439
H -1.161135 2.484173 -0.012262
S 0.539617 -1.973447 -0.001539
C 1.733725 1.834788 -0.002128
O 1.210151 2.937771 0.011411
C 3.121003 -0.911455 0.005977
H 3.639872 -0.294942 -0.731989
H 3.538313 -0.690777 0.993555
H 3.328342 -1.959097 -0.221672
H 2.834620 1.747382 -0.005873
21
thiophene-COOMeH-opt_NBO
Eopt -934.595265
C -0.651101 -0.366283 0.000005
C -1.682851 0.595942 -0.000010
C -3.035181 0.238035 0.000022
C -3.352024 -1.112048 0.000076
C -2.338337 -2.087281 0.000100
C -0.998969 -1.730412 0.000063
C 0.654136 0.268398 -0.000007
C 0.576290 1.628530 -0.000024
H -3.813059 0.995413 0.000002
H -4.393696 -1.417328 0.000100
H -2.610119 -3.138323 0.000147
H -0.220120 -2.483724 0.000071
S -1.042121 2.223715 -0.000077
C 1.935326 -0.467356 -0.000046
O 2.031138 -1.678587 -0.000413
O 2.994659 0.350335 0.000315
C 4.277416 -0.290334 0.000088
H 5.007060 0.517725 0.000020
H 4.392346 -0.907956 0.893266
H 4.392083 -0.907888 -0.893166
H 1.404840 2.325493 -0.000031
24
thiophene-COOMeMe-opt_NBO
Eopt -973.911363
C -0.825139 -0.467408 -0.001219
C -1.767719 0.580677 0.001917
C -3.146968 0.351591 0.005872
C -3.590399 -0.962724 0.006468
C -2.669842 -2.024648 0.003393
C -1.302170 -1.792667 -0.000397
C 0.538529 0.042376 -0.004061
C 0.603206 1.413603 -0.004705
H -3.849065 1.179984 0.008308
H -4.655942 -1.170125 0.009289
H -3.035281 -3.047030 0.003801
H -0.600118 -2.617132 -0.003154
S -0.977809 2.135785 -0.000609
C 1.709908 -0.856700 -0.006226
O 1.641403 -2.071068 -0.027469
C 1.783517 2.338327 -0.008699
H 2.392884 2.184766 0.885440
H 2.417512 2.146373 -0.877563
H 1.457603 3.380416 -0.035579
O 2.877988 -0.202440 0.018724
C 4.052979 -1.023683 0.015185
H 4.892797 -0.330749 0.035984
H 4.068296 -1.667984 0.896738
H 4.085725 -1.635485 -0.888726
21
thiophene-MeMe-opt_NBO Eopt
-785.320597
C -0.433892 0.582141 -0.000006
C -0.663296 -0.808215 0.000000
C -1.954686 -1.342882 0.000004
C -3.030825 -0.463198 0.000010
C -2.822861 0.926899 0.000011
C -1.537476 1.451767 -0.000001
C 0.978538 0.920852 -0.000011
C 1.772285 -0.184827 -0.000022
H -2.114344 -2.417073 0.000003
H -4.042803 -0.856474 0.000016
H -3.678022 1.595958 0.000021
H -1.381475 2.527220 0.000002
S 0.847389 -1.683789 -0.000014
C 3.265967 -0.289381 0.000032
H 3.724636 0.700841 -0.000499
H 3.622041 -0.828046 0.884247
H 3.622052 -0.829008 -0.883585
C 1.435469 2.350119 -0.000009
H 1.055536 2.876740 -0.882535
H 1.055725 2.876693 0.882622
H 2.523081 2.434120 -0.000123

9. References

1. Jeong, Y. C.; Park, D. G.; Kim, E.; Yang, S. I.; Ahn, K. H. Polymerization of a Photochromic Diarylethene by Friedel−Crafts Alkylation. *Macromolecules* **2006**, 39, 3106–3109. DOI: 10.1021/ma0602167.

2. Vekariya, R. H; Aubé, J. Hexafluoro-2-propanol-Promoted Intermolecular Friedel–Crafts Acylation Reaction. *Org. Lett.* **2016**, *18*, 3534–3537. DOI: 10.1021/acs.orglett.6b01460.

3. Raghu, M.; Grover, J.; Ramasastry, S S V. Cyclopenta[b]annulation of Heteroarenes by Organocatalytic γ′[C(sp 3)−H] Functionalization of Ynones. *Chem. Eur. J.*, **2016**, *22*, 18316-18321. DOI: 10.1002/chem.201604562.

4. Sidduri, A.; Rozema, M. J.; Knochel, P., Selective mono- and polymethylene homologations of copper reagents using (iodomethyl)zinc iodide. *J. Org. Chem*. **1993**, *58*, 2694-2713. https://doi.org/10.1021/jo00062a010.

5. Bhushan, B.; Erdmann, A.; Zhang, Y.; Belle, R.; Johannson, C.; Oppermann, U.; Hopkinson, R. J.; Schofield, C. J.; Kawamura, A. Investigations on small molecule inhibitors targeting the histone H3K4 tri-methyllysine binding PHD-finger of JmjC histone demethylases. *Bioorganic & Medicinal Chemistry*, **2018**, *26*, 2984-2991.

6. Hamprecht, D.; Micheli, F. Preparation of 2,8-diaza- (or 8-thia-2-aza-) cyclopentaindenones having activity at 5-HT2C receptor. PCT Int. Appl. (2004), WO 2004081010 A1 Sep 23, 2004. 7. Mukumoto, F.; Tamaki, H.; Kusaka, S.; Iwakoshi, M. Preparation of benzo[b]thiophene-2-carboxylic acid derivatives and method for promoting plant growth. PCT Int. Appl., 2014073622, 15 May 2014.

8. Liu, K.; Jia, F.; Xi, H.; Li, Y.; Zheng, X.; Guo, Q.; Shen, B.; Li, Z. Direct Benzothiophene Formation via Oxygen-Triggered Intermolecular Cyclization of Thiophenols and Alkynes Assisted by Manganese/PhCOOH. *Org. Lett.* **2013**, *15*, 2026–2029. DOI: 10.1021/ol400719d.

9. Luyksaar, S. I.; Migulin, V. A; Nabatov, B. V; Krayushkin, M. M. Synthesis and photochromism of functionalized benzothiophene-based fulgides and fulgimides. *Russ Chem Bull* **2010**, *59*, 446–451. https://doi.org/10.1007/s11172-010-0099-y.

10. Y. Zhao, D. G. Truhlar, *Theor. Chem. Acc*. **2008**, *120*, 215 – 241.

11. a) W. J. Hehre, R. Ditchfield, J. A. Pople, *J. Chem. Phys.* **1972**, *56*, 2257 – 2261; b) P. C. Hariharan, J. A. Pople, *Theor. Chim. Acta.* **1973**, *28*, 213 – 222; c) R. Krishnan, J. S. Binkley, R. Seeger, J. A. Pople, *J. Chem. Phys.*, **1980**, *72*, 650 – 654; d) A. D. McLean, G. S. Chandler, *J. Chem. Phys.* **1980**, *72*, 5639 – 5648; e) M. M. Francl, W. J. Pietro, W. J. Hehre, J. S. Binkley, M. S. Gordon, D. J. DeFrees, J. A., Pople, *J. Chem. Phys.*, **1982**, *77*, 3654 – 3665.

12. S. Grimme, J. Antony, S. Ehrlich, H. Krieg, *J. Chem. Phys. 2010*, **132**, 154104 – 154119.

13. a) F. Weigend, R. Ahlrichs. *Phys. Chem. Chem. Phys.* **2005**, *7*, 3297 – 3305; b) F. Weigend *Phys. Chem. Chem. Phys.* **2006**, *8*, 1057 – 1065.

14. A. V. Marenich, C. J. Cramer, D. G. Truhlar, *J. Phys. Chem. B*, **2009**, *113*, 6378-6396.

15. Gaussian 16, Revision C.01, M. J. Frisch, G. W. Trucks, H. B. Schlegel, G. E. Scuseria, M. A. Robb, J. R. Cheeseman, G. Scalmani, V. Barone, G. A. Petersson, H. Nakatsuji, X. Li, M. Caricato, A. V. Marenich, J. Bloino, B. G. Janesko, R. Gomperts, B. Mennucci, H. P. Hratchian, J. V. Ortiz, A. F. Izmaylov, J. L. Sonnenberg, D. Williams-Young, F. Ding, F. Lipparini, F. Egidi, J. Goings, B. Peng, A. Petrone, T. Henderson, D. Ranasinghe, V. G. Zakrzewski, J. Gao, N. Rega, G. Zheng, W. Liang, M. Hada, M. Ehara, K. Toyota, R. Fukuda, J. Hasegawa, M. Ishida, T. Nakajima, Y. Honda, O. Kitao, H. Nakai, T. Vreven, K. Throssell, J. A. Jr. Montgomery, J. E. Peralta, F. Ogliaro, M. J. Bearpark, J. J. Heyd, E. N. Brothers, K. N. Kudin, V. N. Staroverov, T. A. Keith, R. Kobayashi, J. Normand, K. Raghavachari, A. P. Rendell, J. C. Burant, S. S. Iyengar, J. Tomasi, M. Cossi, J. M. Millam, M. Klene, C. Adamo, R. Cammi, J. W. Ochterski, R. L. Martin, K. Morokuma, O. Farkas, J. B. Foresman, D. J. Fox, Gaussian, Inc., Wallingford CT, 2016.

16. E. E. Kwan, R. Y. Liu, *J. Chem. Theory Comput.,* **2015**, *11,* 5083-5089.

17. E. D. Glendening, C. R. Landis, F. Weinhold, *J. Comput. Chem*. **2013**, *34*, 1429-1437.

18. R. G. Parr, W. Yang, *J. Am. Chem. Soc.*, **1984**, *106*, 4049-4050.

19. The PyMOL Molecular Graphics System, version 2.0.7, Schrodinger, LLC.

20. K. Fukui, *Acc. Chem. Res.* **1981**, *14*, 363−368.

21. S. Grimme, Chem. Eur. J. 2012, 18, 9955−9964.

22. G. Luchini, J. V. Alegre-Requena, I. Funes-Ardoiz, R. S. Paton, *F1000Research*, **2020**, *9*, 291.

23. V. S. Bryantsev, M. S. Diallo, W. A. Goddard III, *J. Phys. Chem. B,* **2008**, *112,* 9709–9719.

24. R. E. Plata, D. A. Singleton, *J. Am. Chem. Soc,* **2015**, *137*, 3811–3826.

10. NMR spectra

1n ^{1}H NMR (400 MHz, $CDCl_3$)

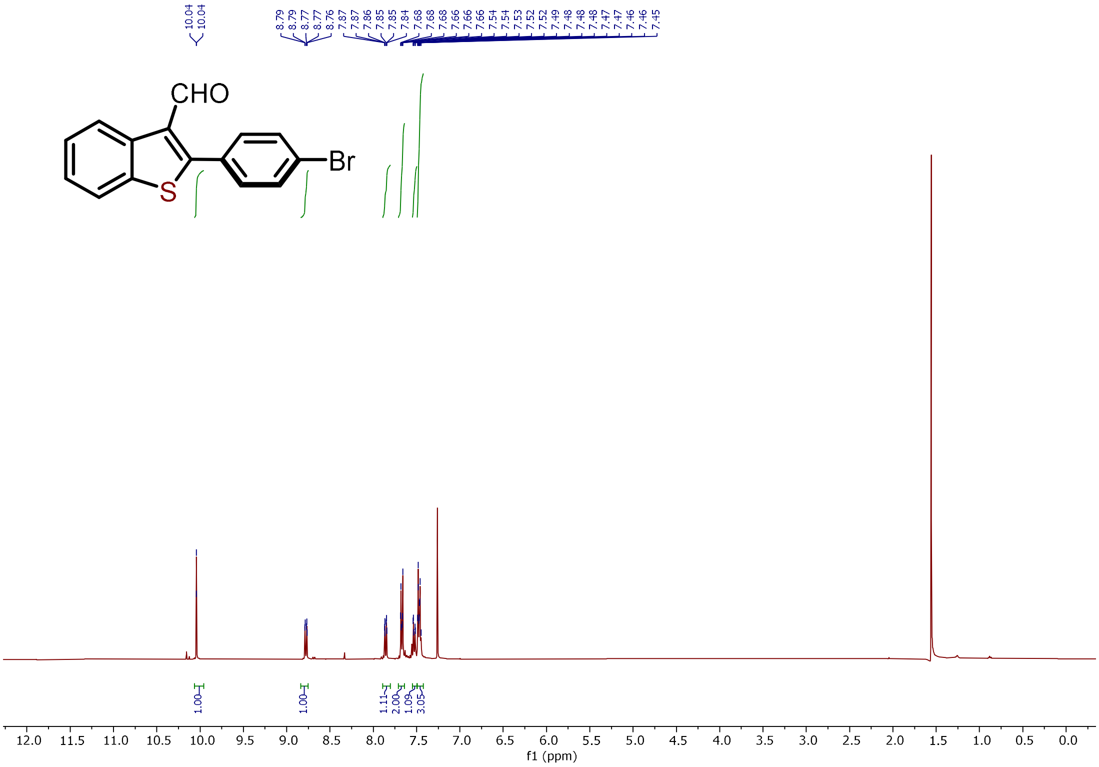

1n ^{13}C NMR (101 MHz, $CDCl_3$)

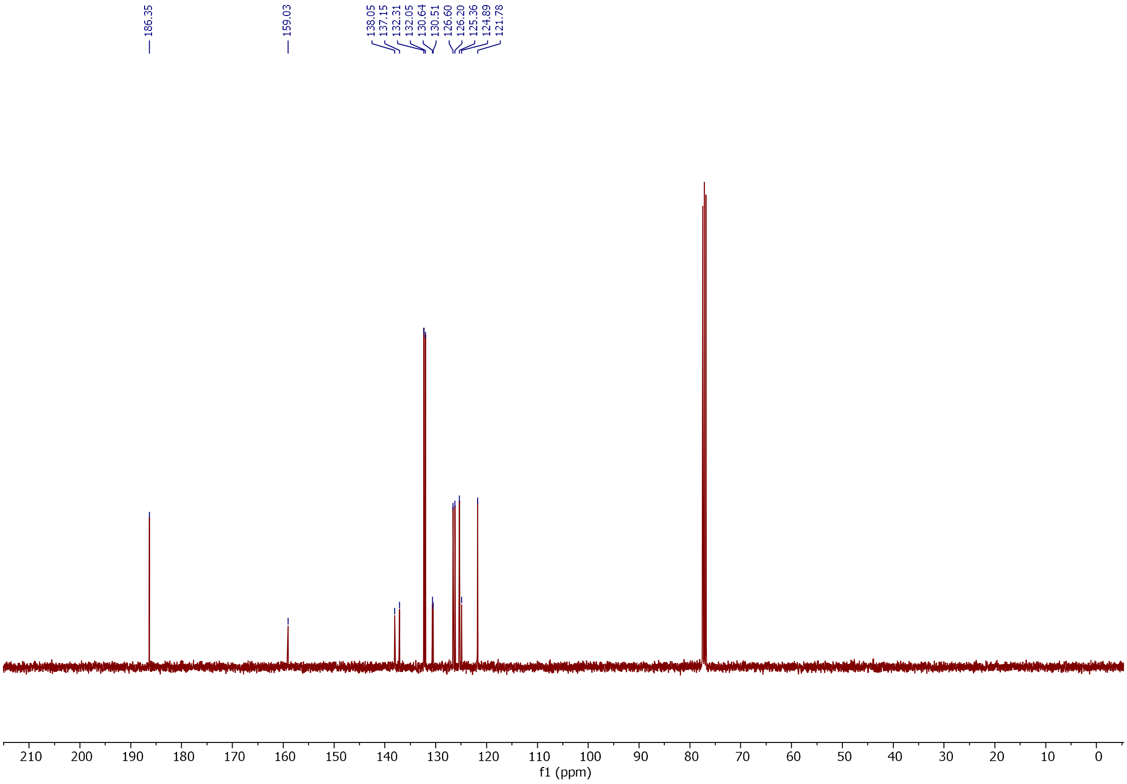

1z ^{1}H NMR (400 MHz, $CDCl_3$)

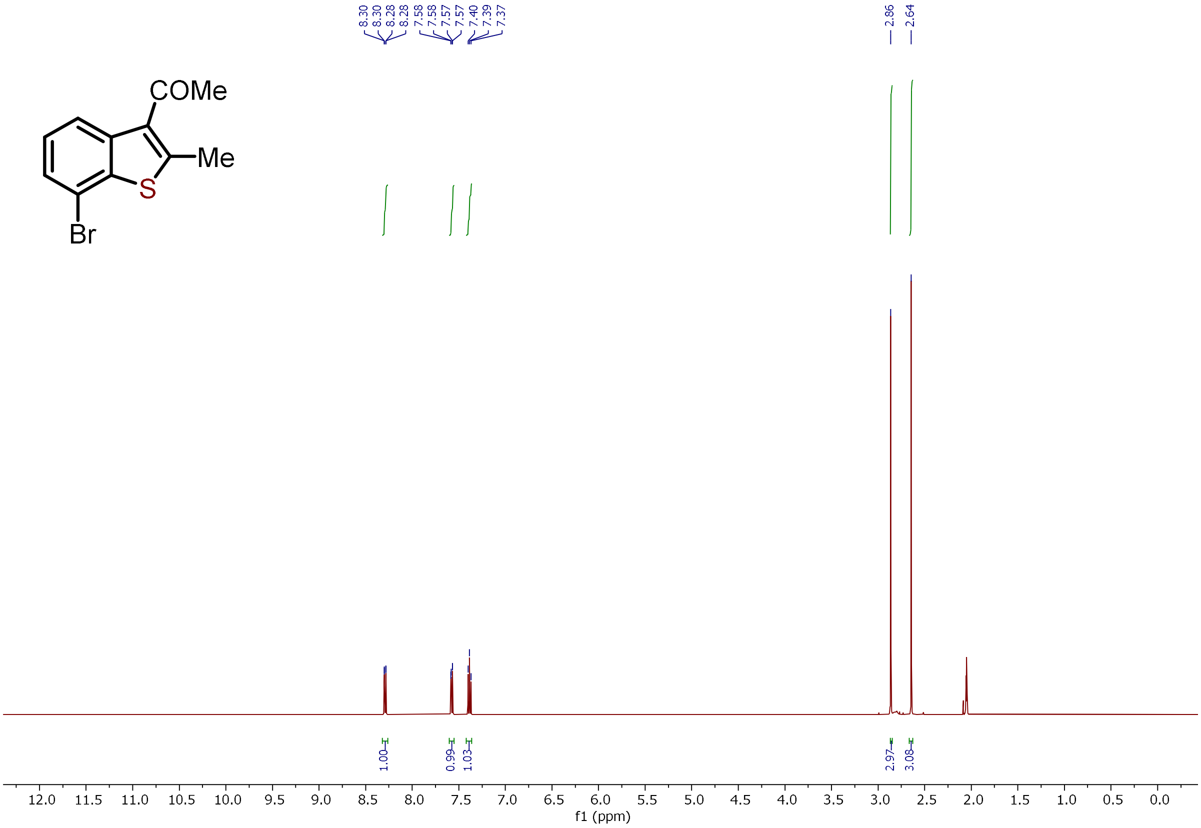

1z ^{13}C NMR (101 MHz, $CDCl_3$)

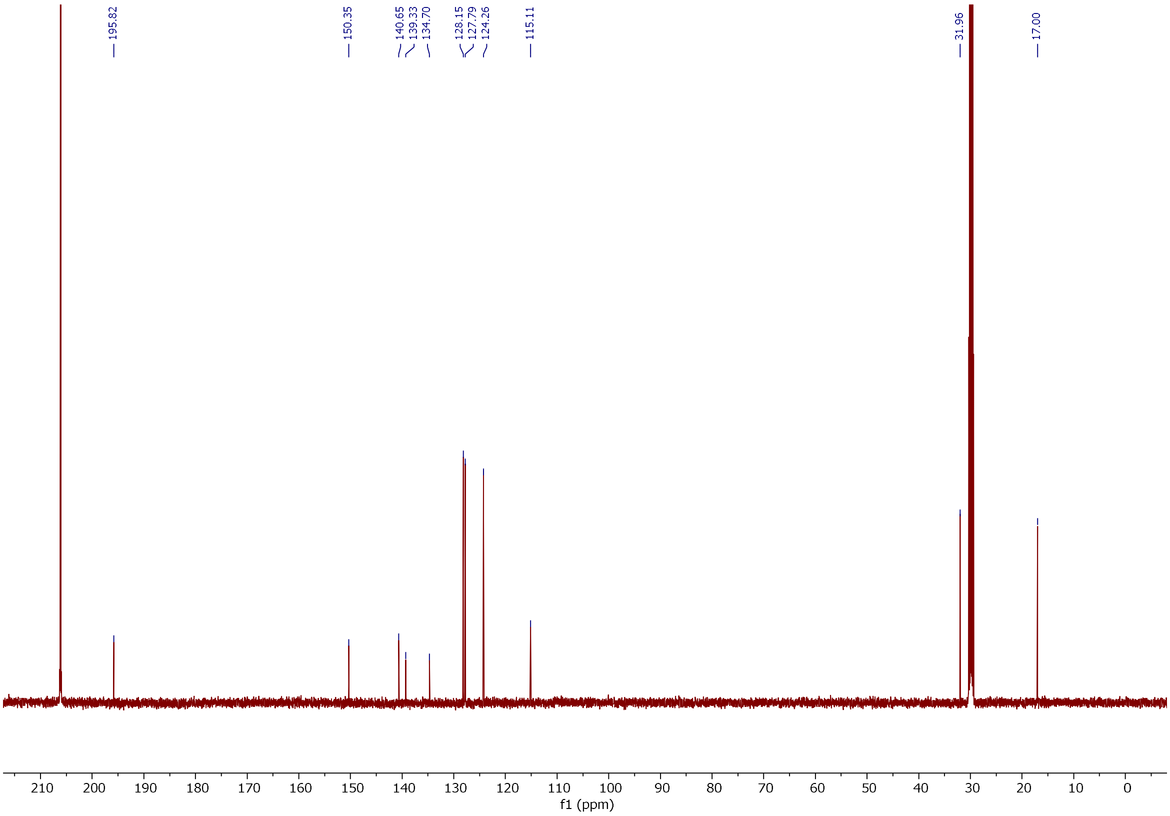

1ah ^{1}H NMR (400 MHz, $CDCl_3$)

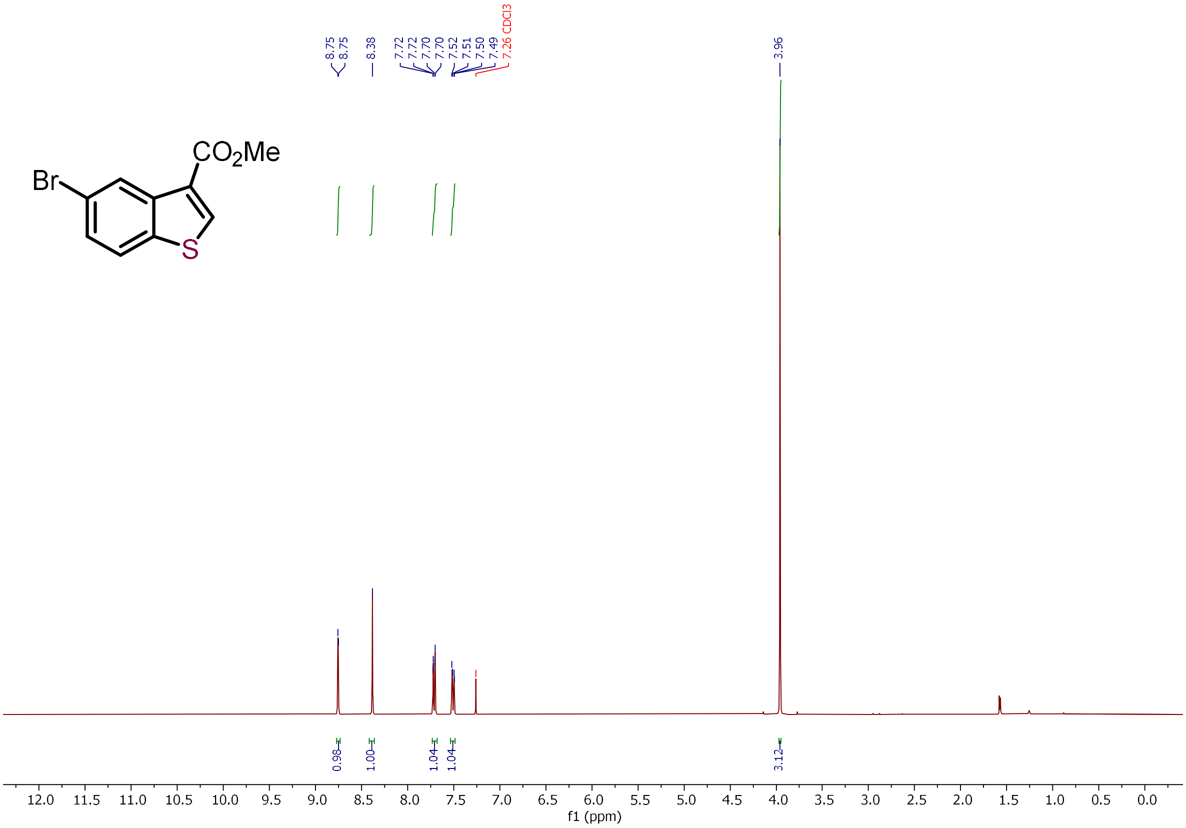

1ah ^{13}C NMR (101 MHz, $CDCl_3$)

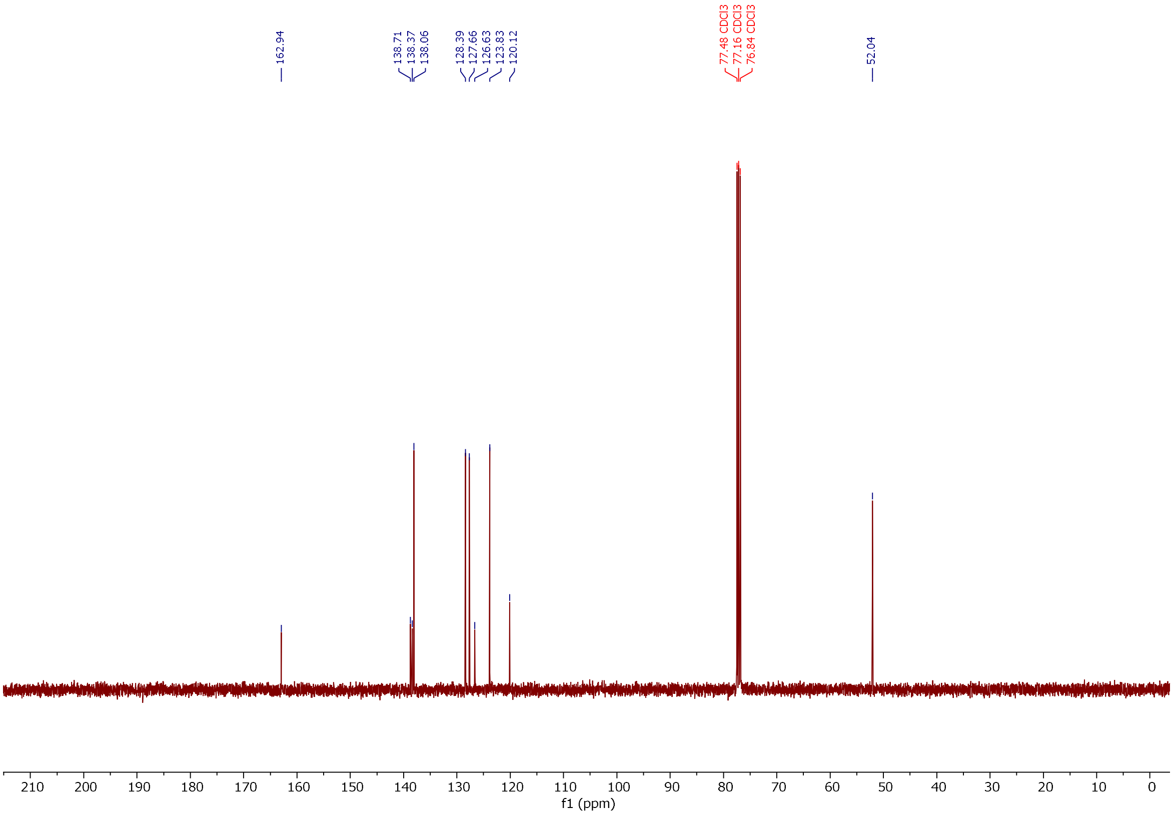

1ai ^{1}H NMR (400 MHz, $CDCl_3$)

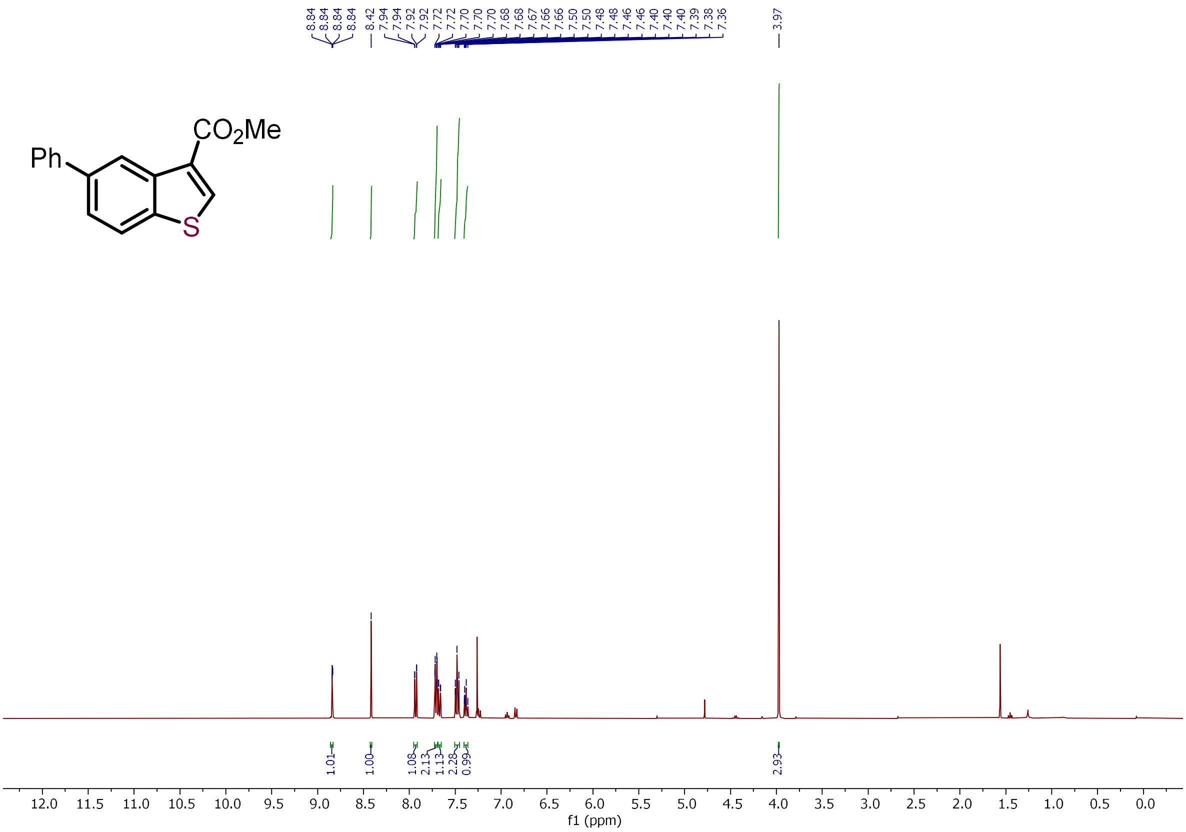

1ai ^{13}C NMR (101 MHz, $CDCl_3$)

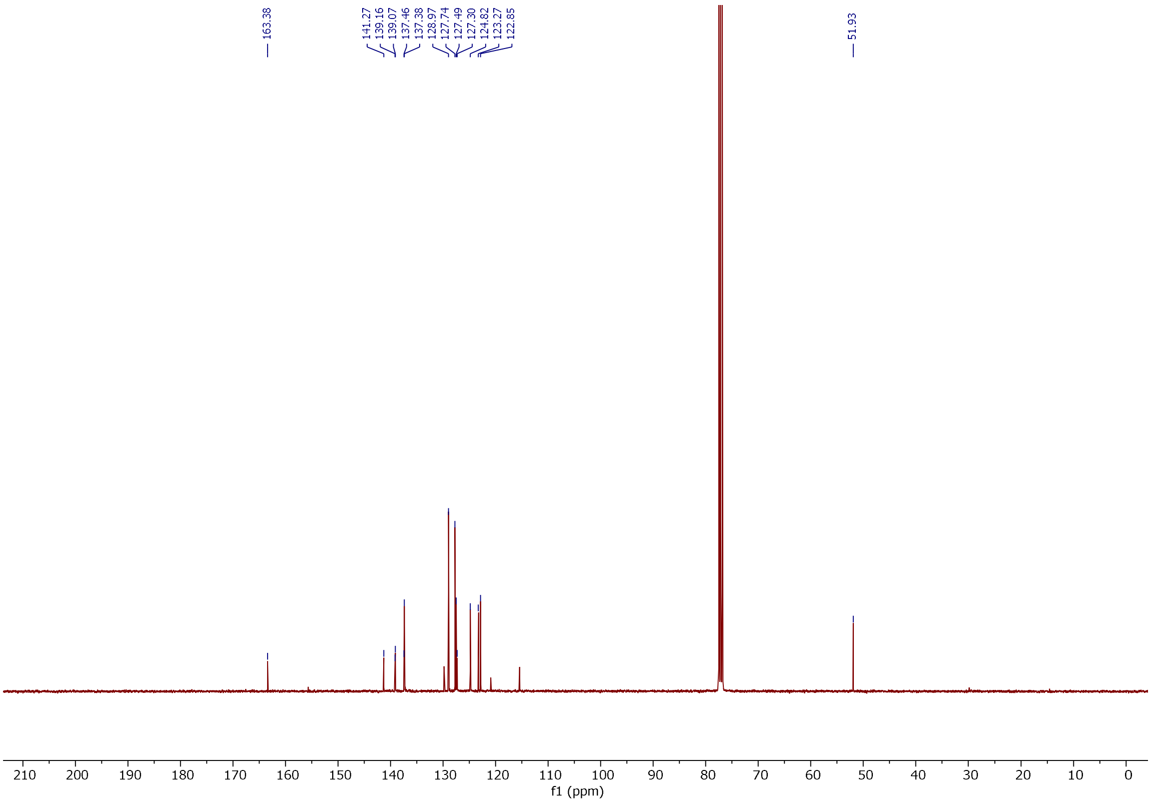

2 ^{1}H NMR (400 MHz, $CDCl_3$)

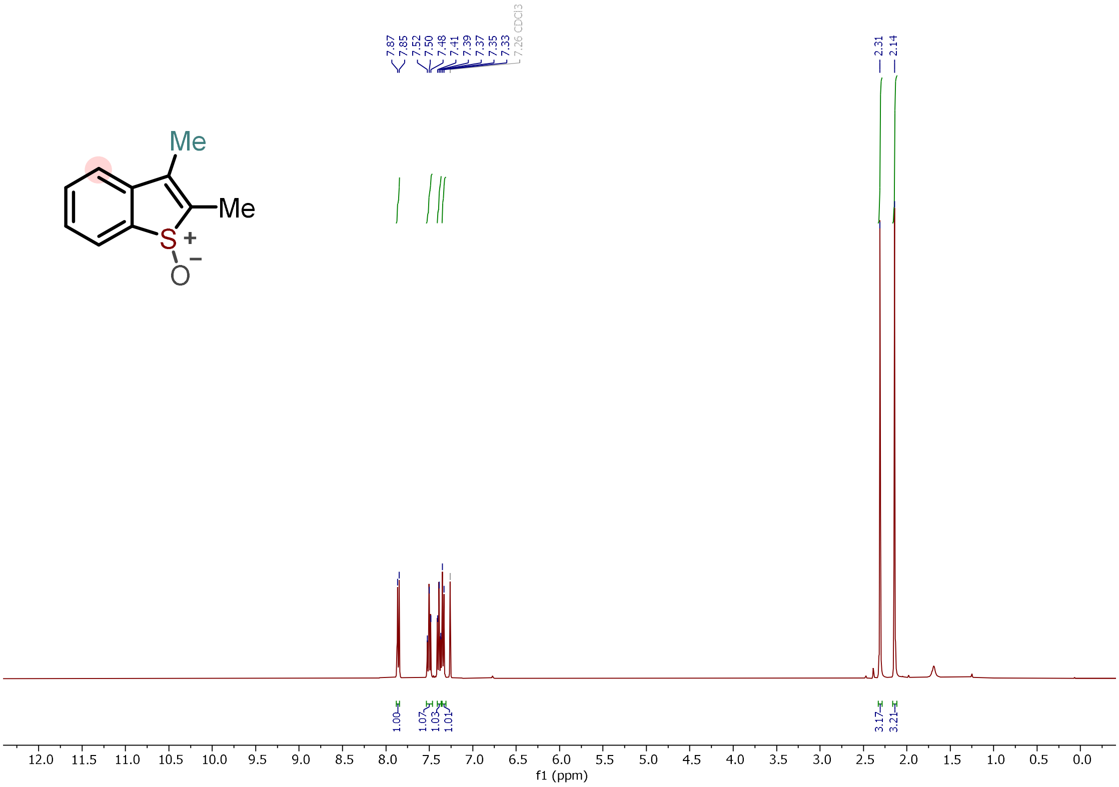

2 ^{13}C NMR (101 MHz, $CDCl_3$)

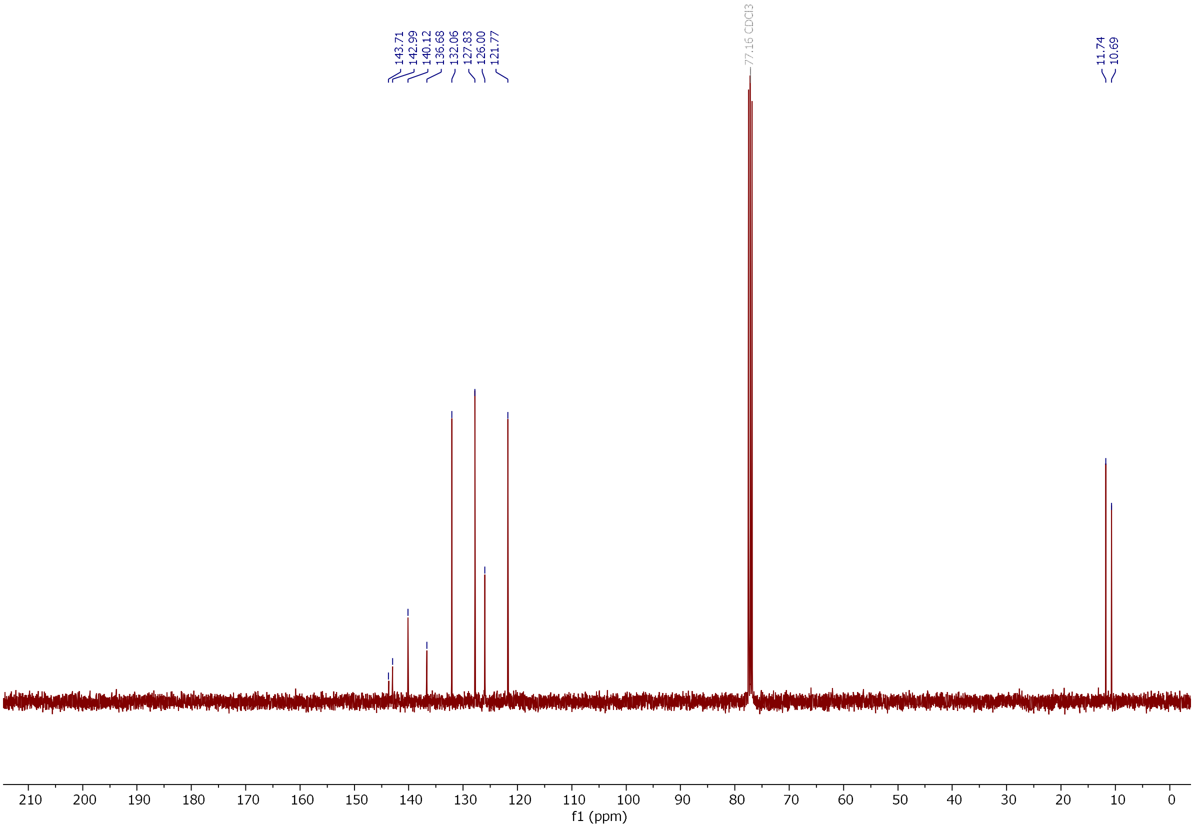

2a ^{1}H NMR (400 MHz, $CDCl_3$)

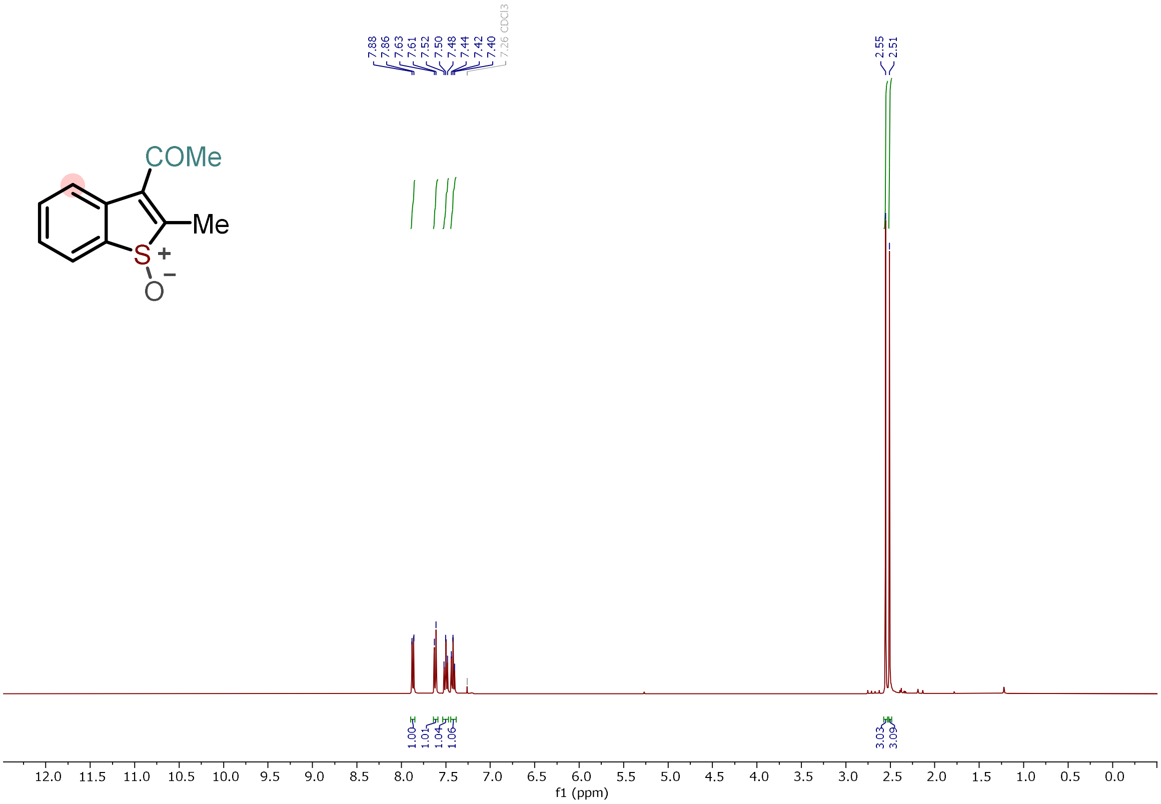

2a ^{13}C NMR (101 MHz, $CDCl_3$)

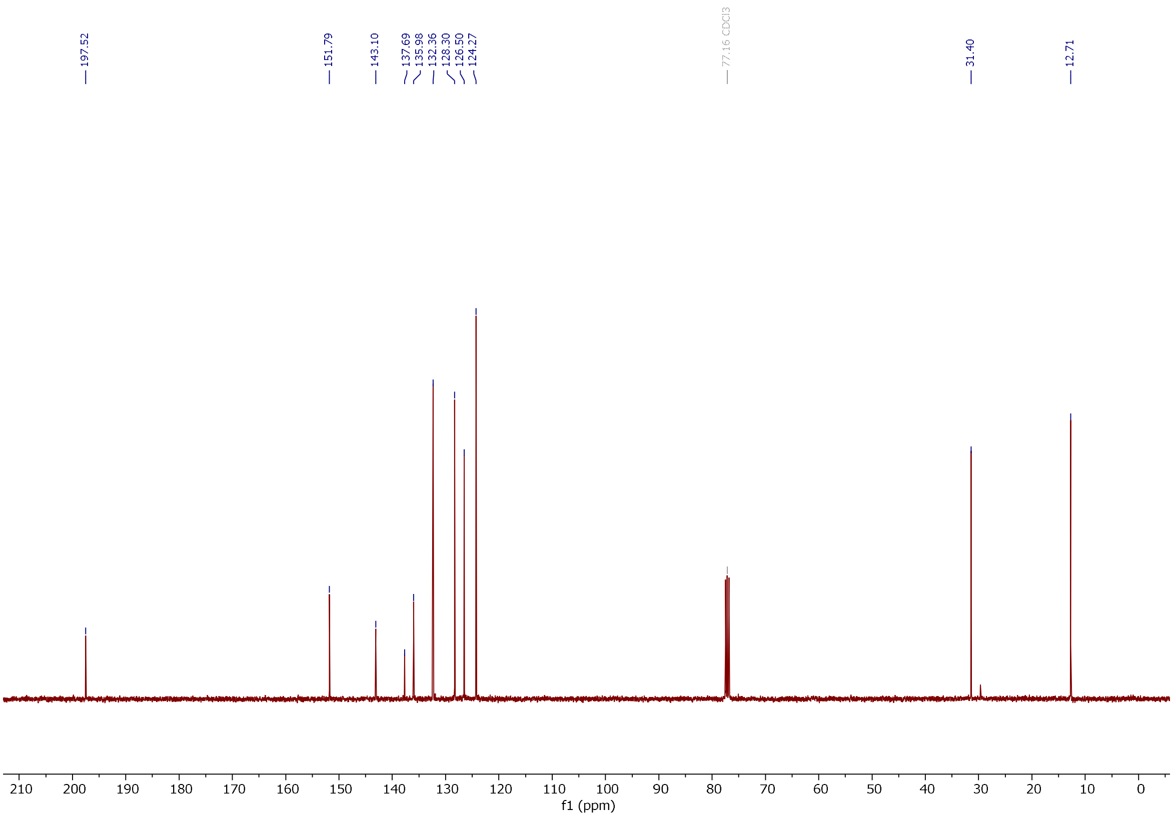

2b ^{1}H NMR (400 MHz, $CDCl_3$)

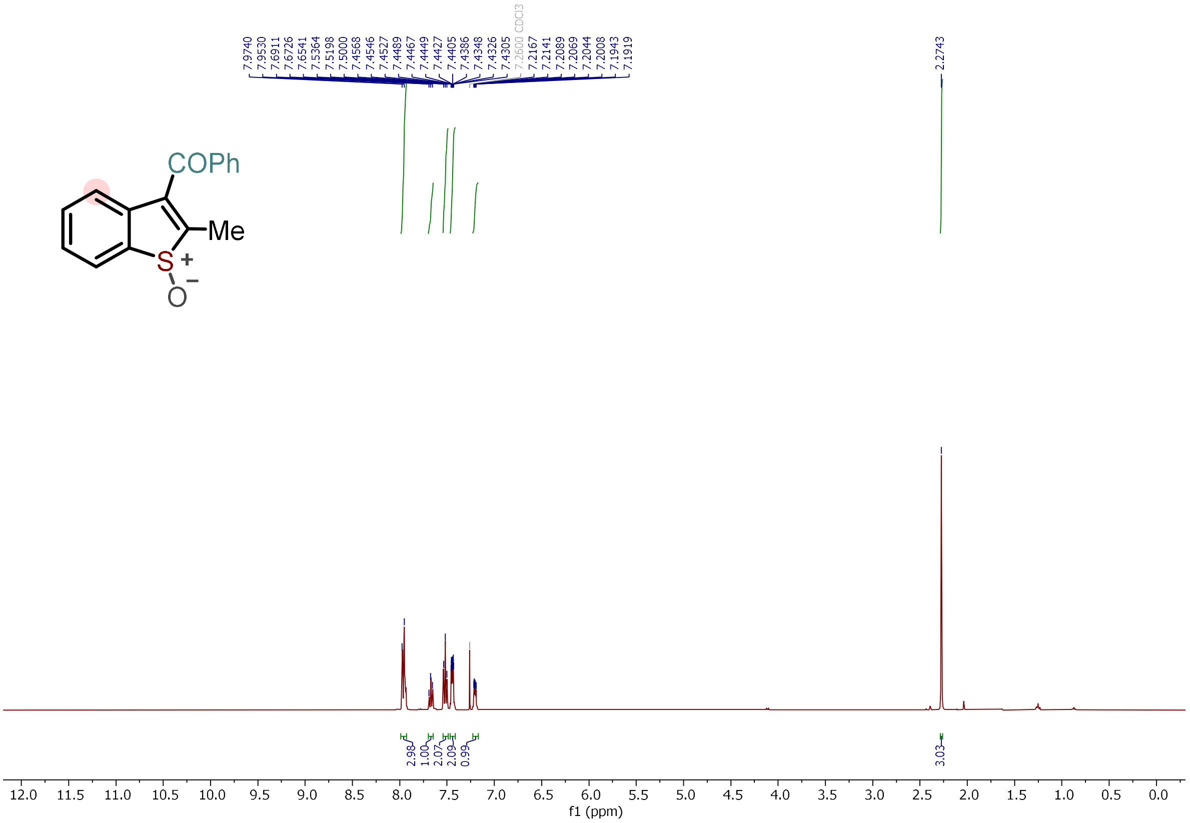

2b ^{13}C NMR (101 MHz, $CDCl_3$)

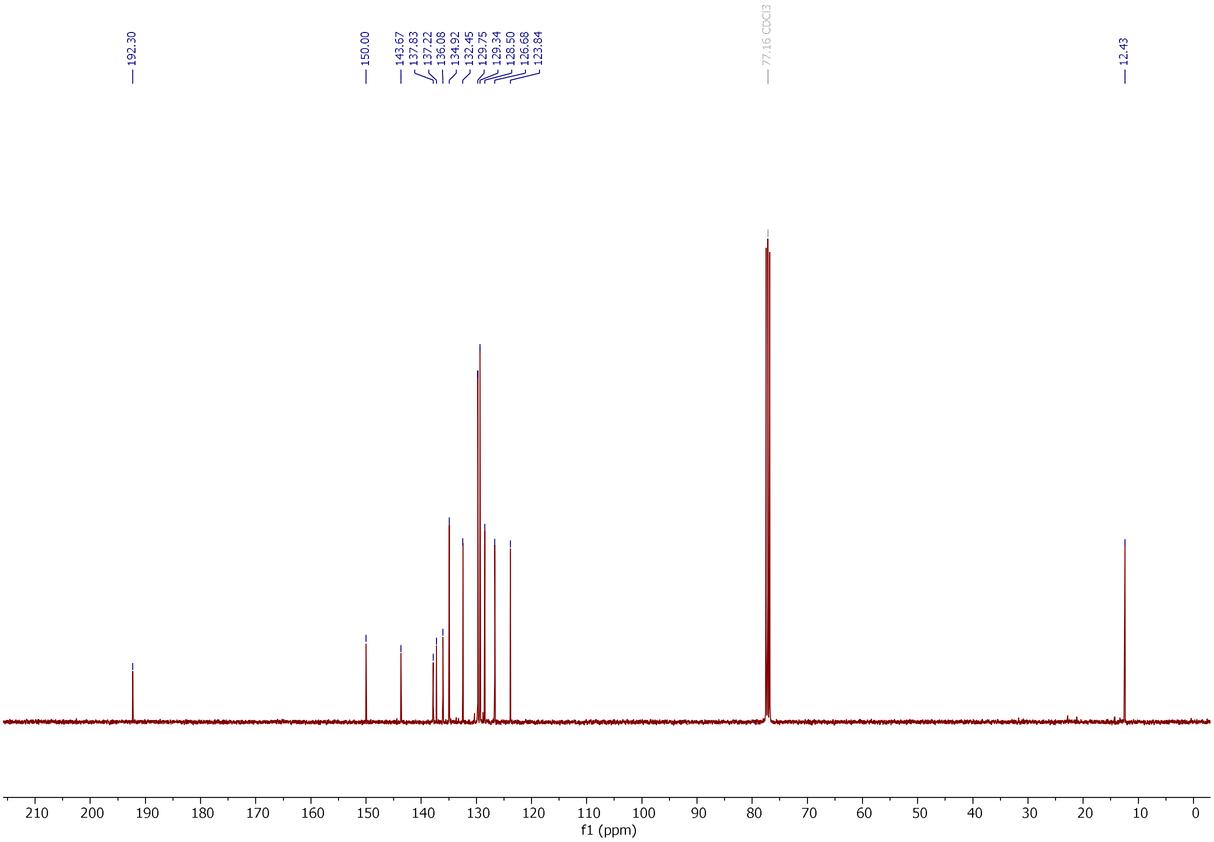

2c ^{1}H NMR (400 MHz, $CDCl_3$)

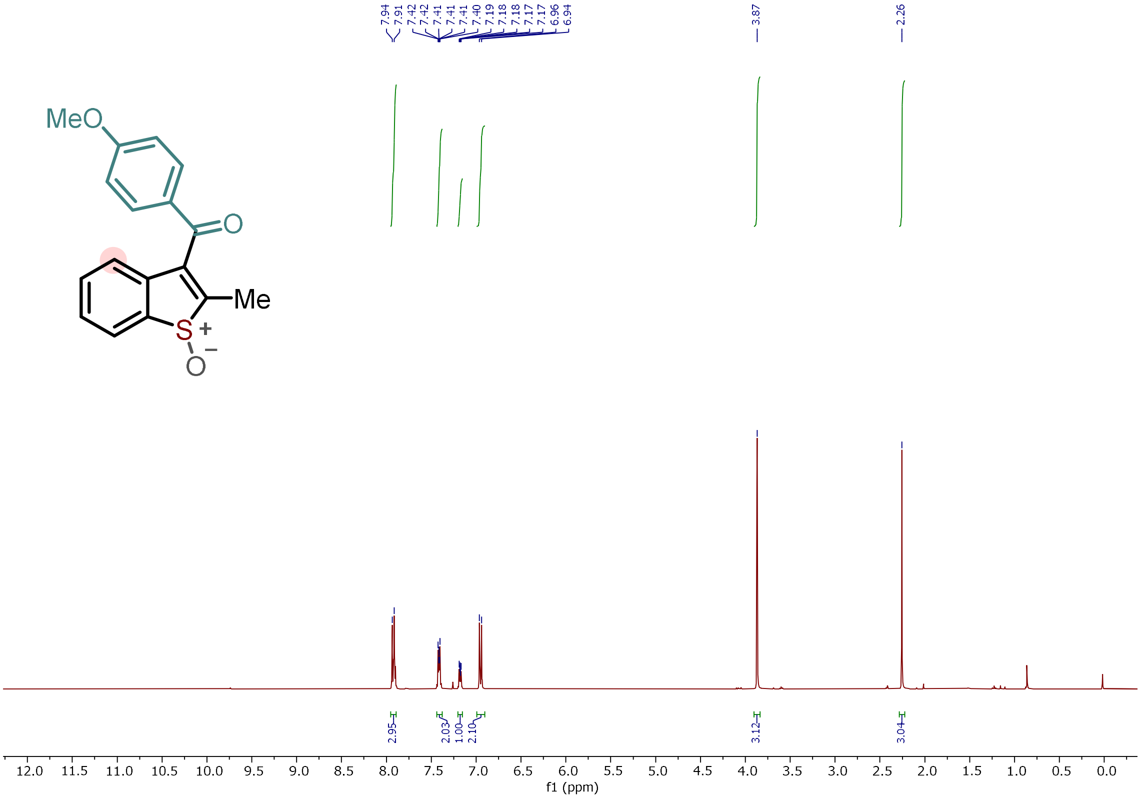

2c ^{13}C NMR (101 MHz, $CDCl_3$)

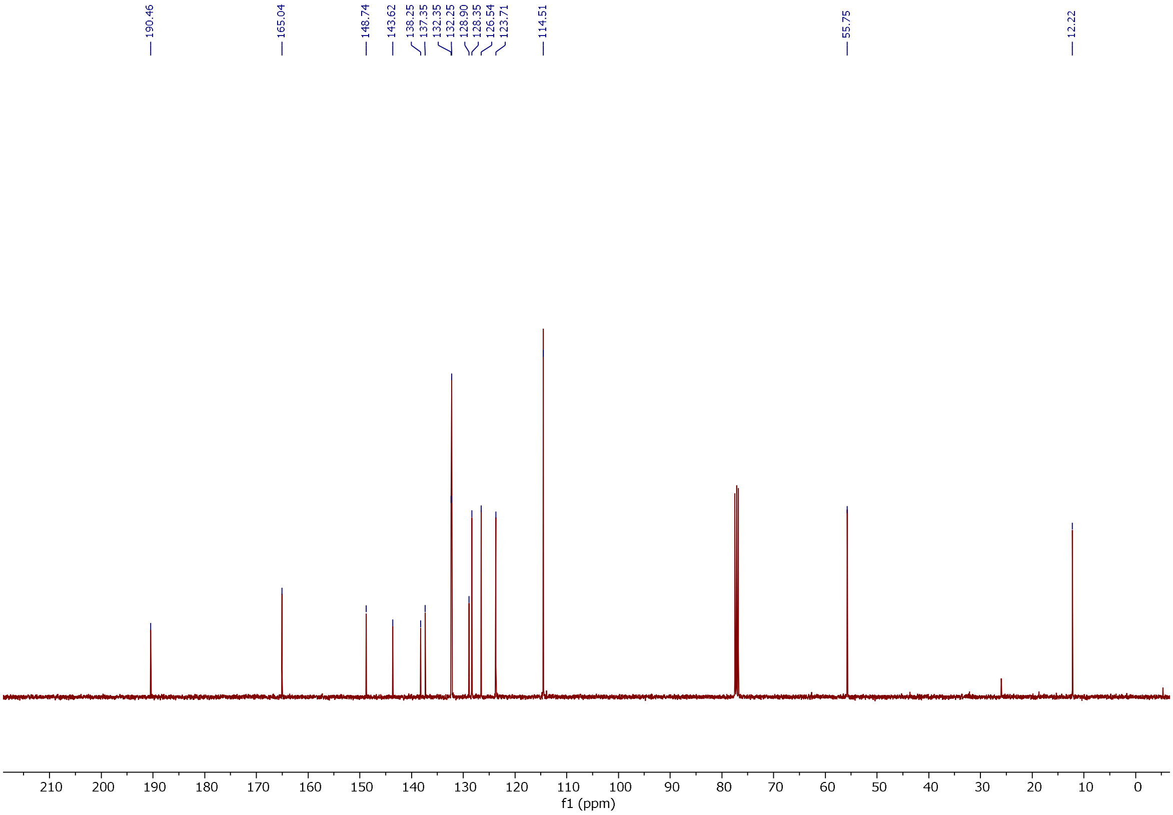

2d ^{1}H NMR (400 MHz, $CDCl_3$)

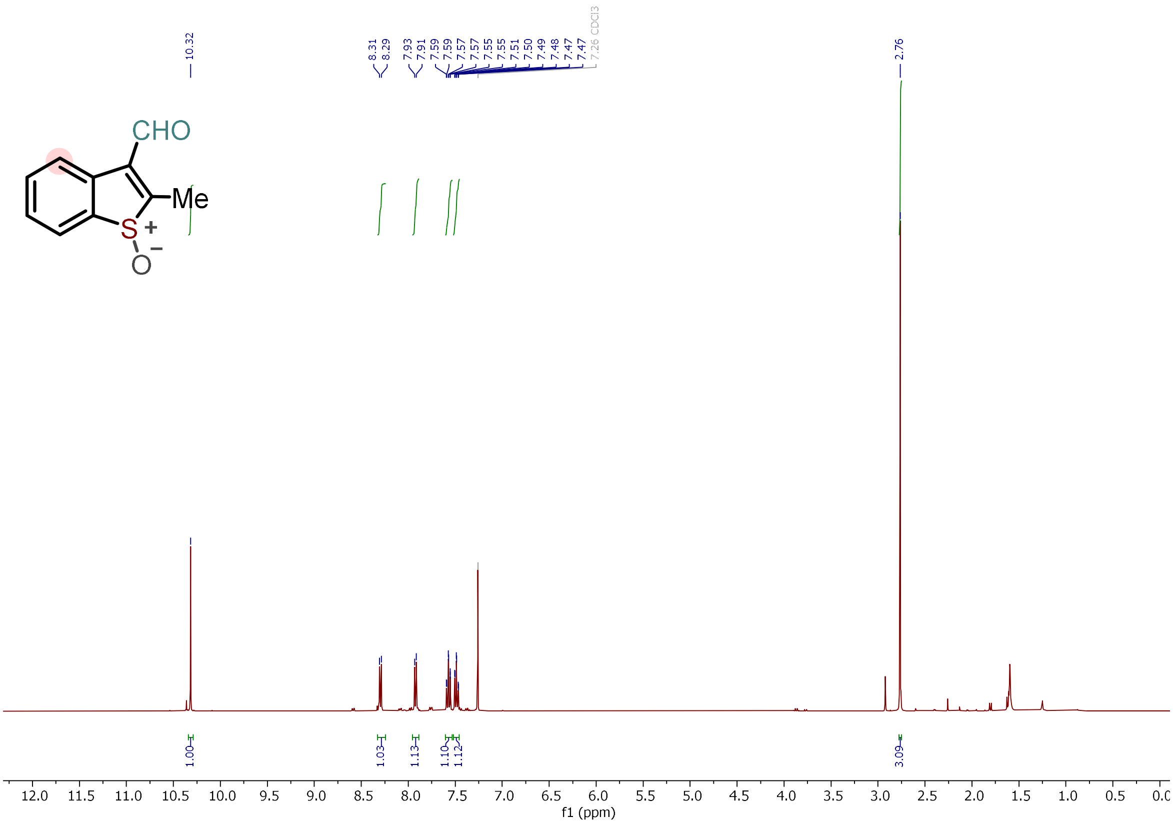

2d ^{13}C NMR (101 MHz, $CDCl_3$)

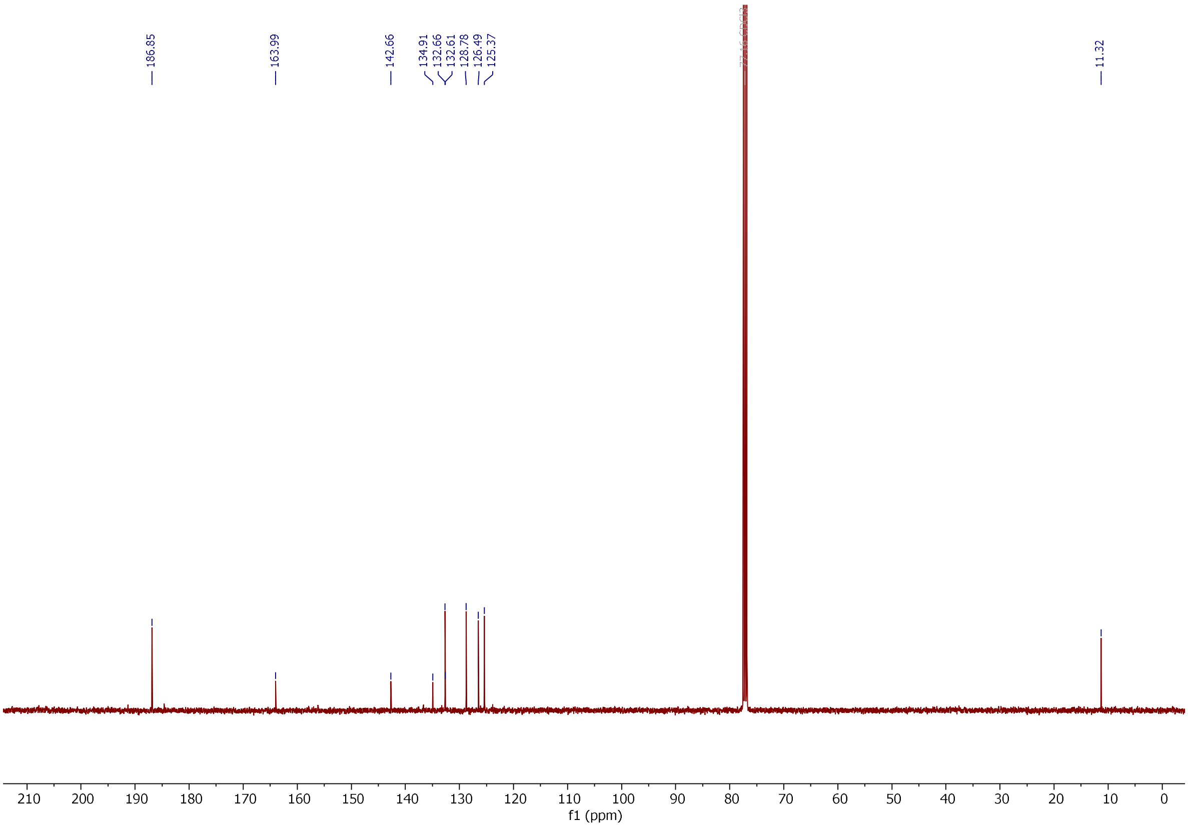

2n ^{1}H NMR (400 MHz, $CDCl_3$)

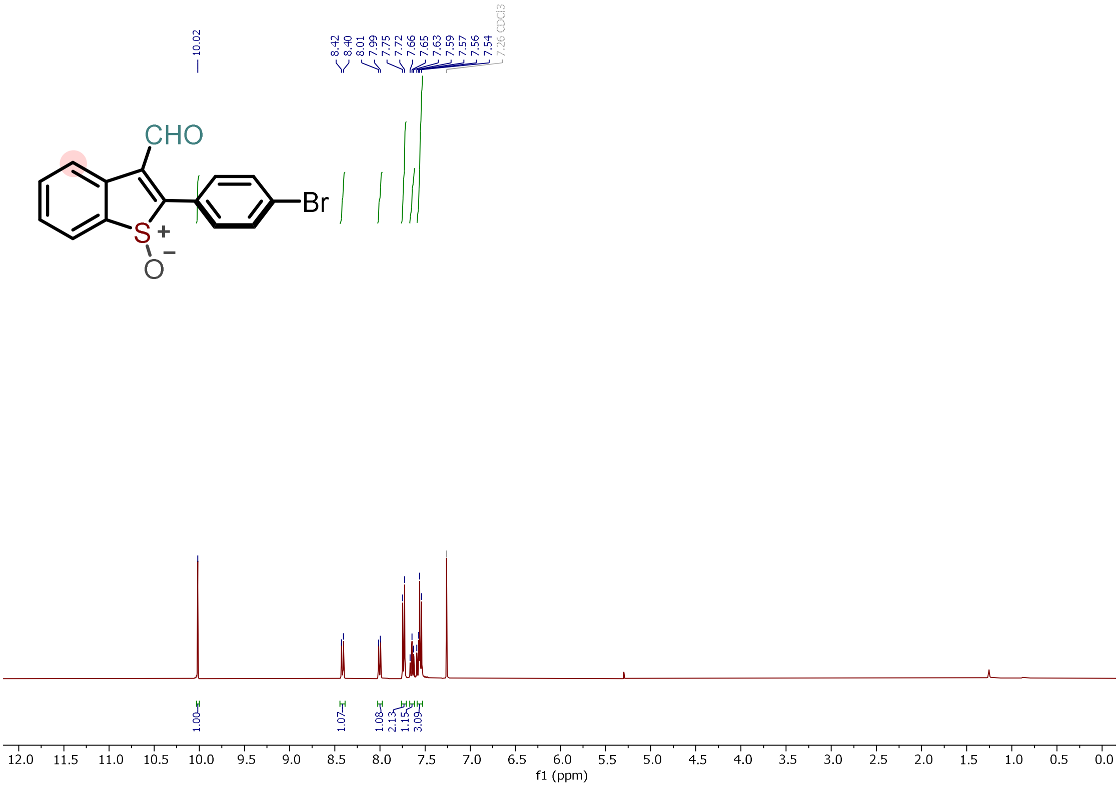

2n ^{13}C NMR (101 MHz, $CDCl_3$)

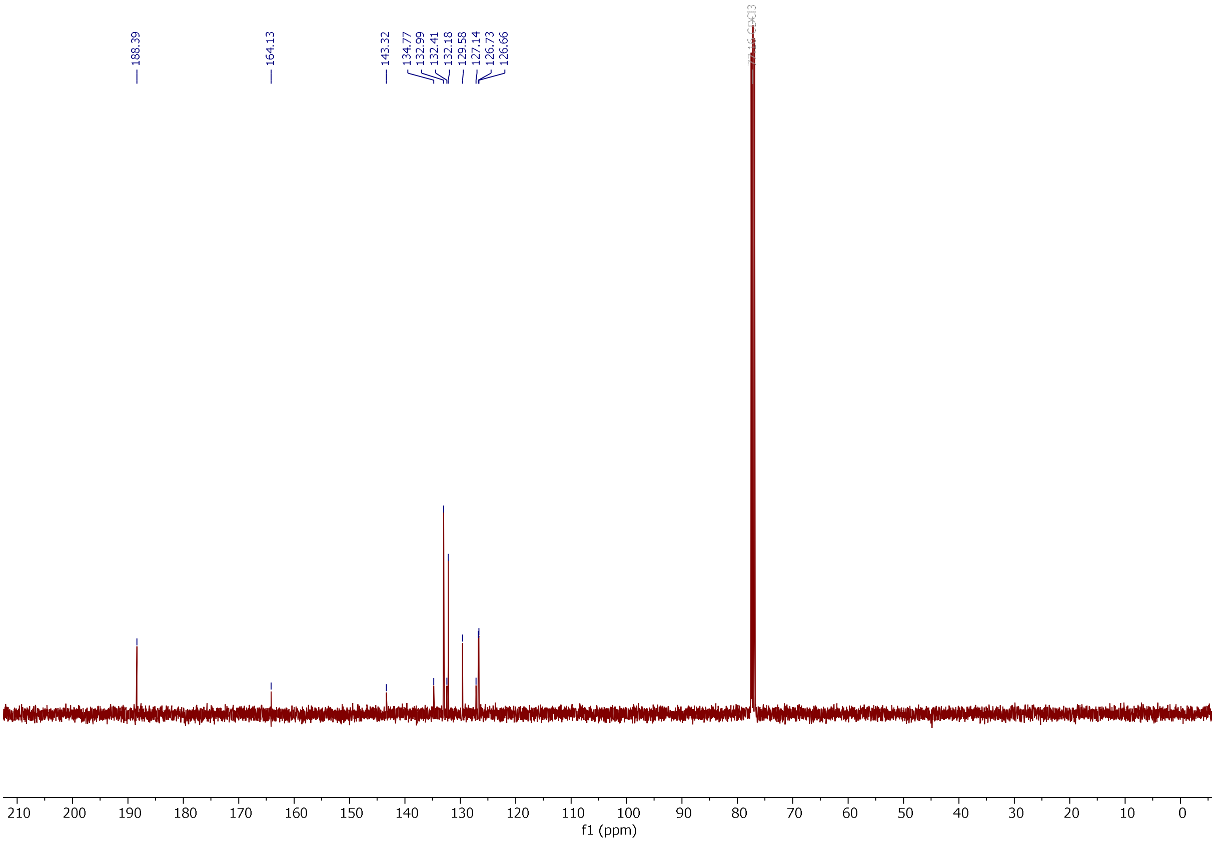

2s ^{1}H NMR (400 MHz, CDCl$_3$)

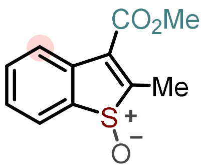

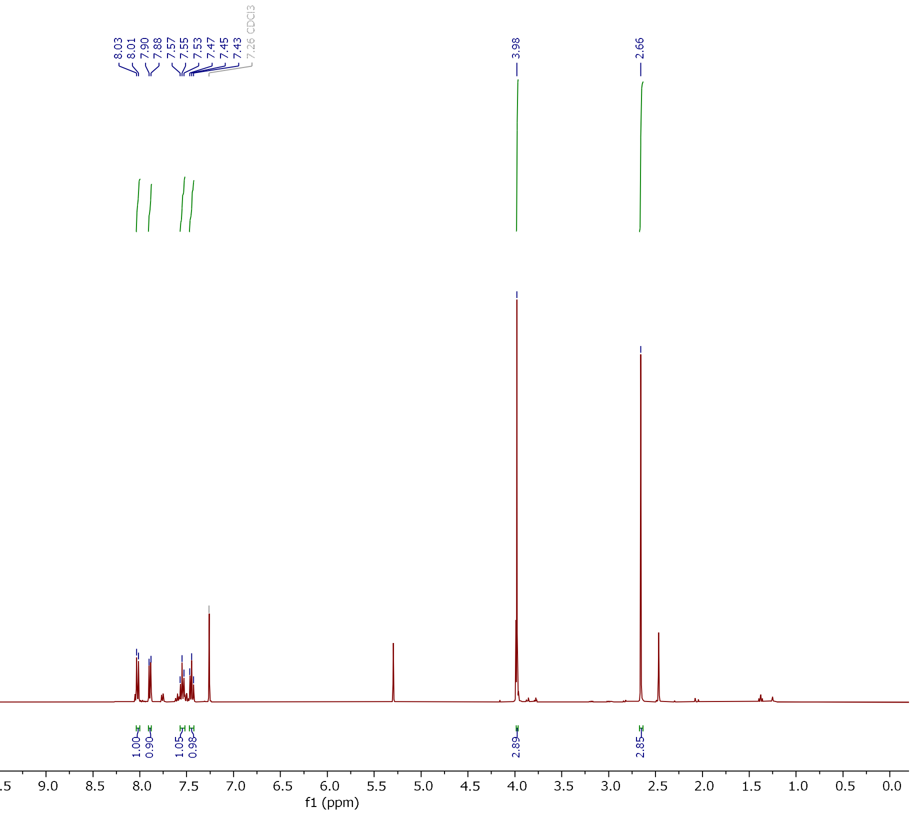

2s ^{13}C NMR (101 MHz, CDCl$_3$)

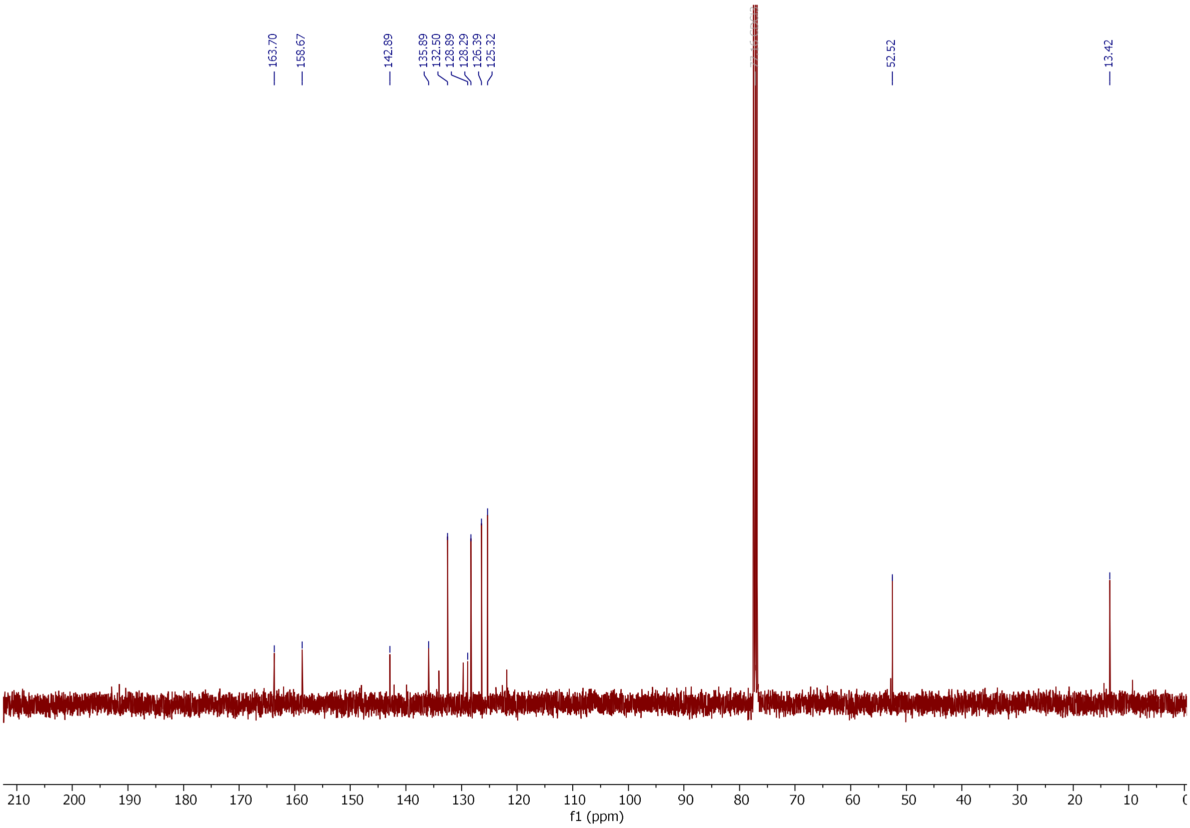

2u ^{1}H NMR (400 MHz, $CDCl_3$)

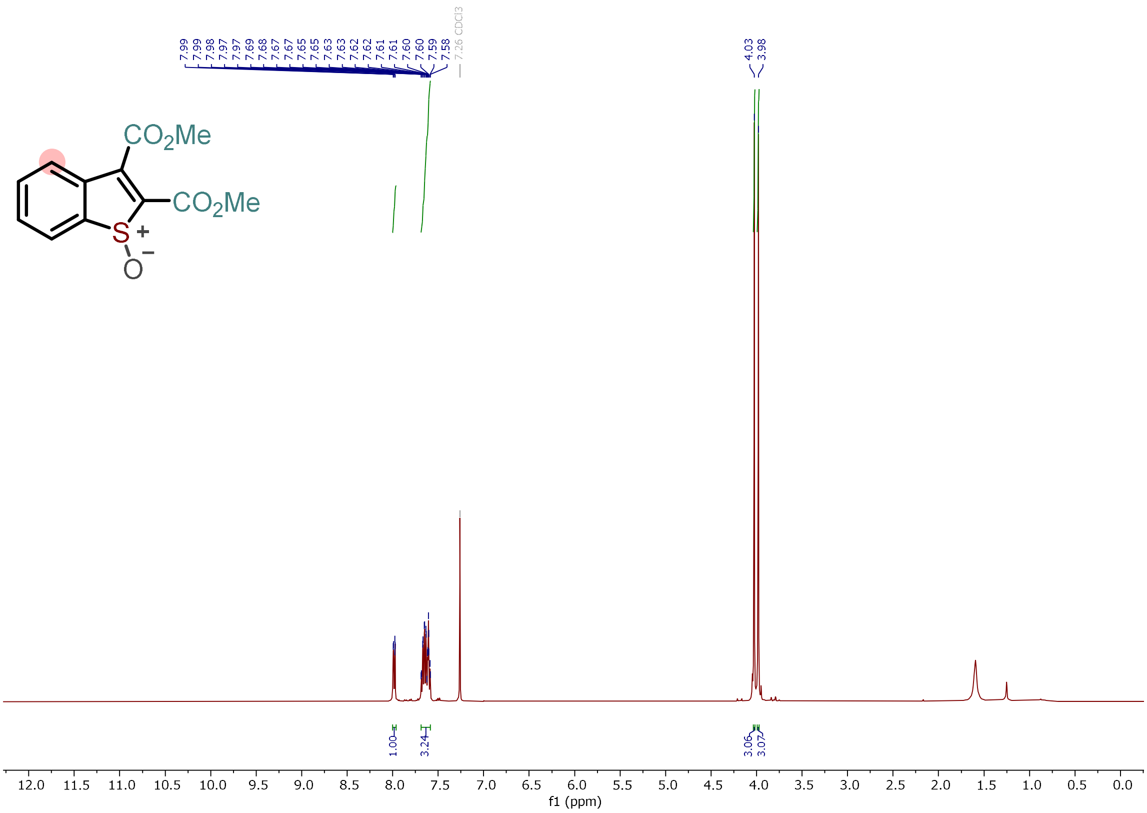

2u ^{13}C NMR (101 MHz, $CDCl_3$)

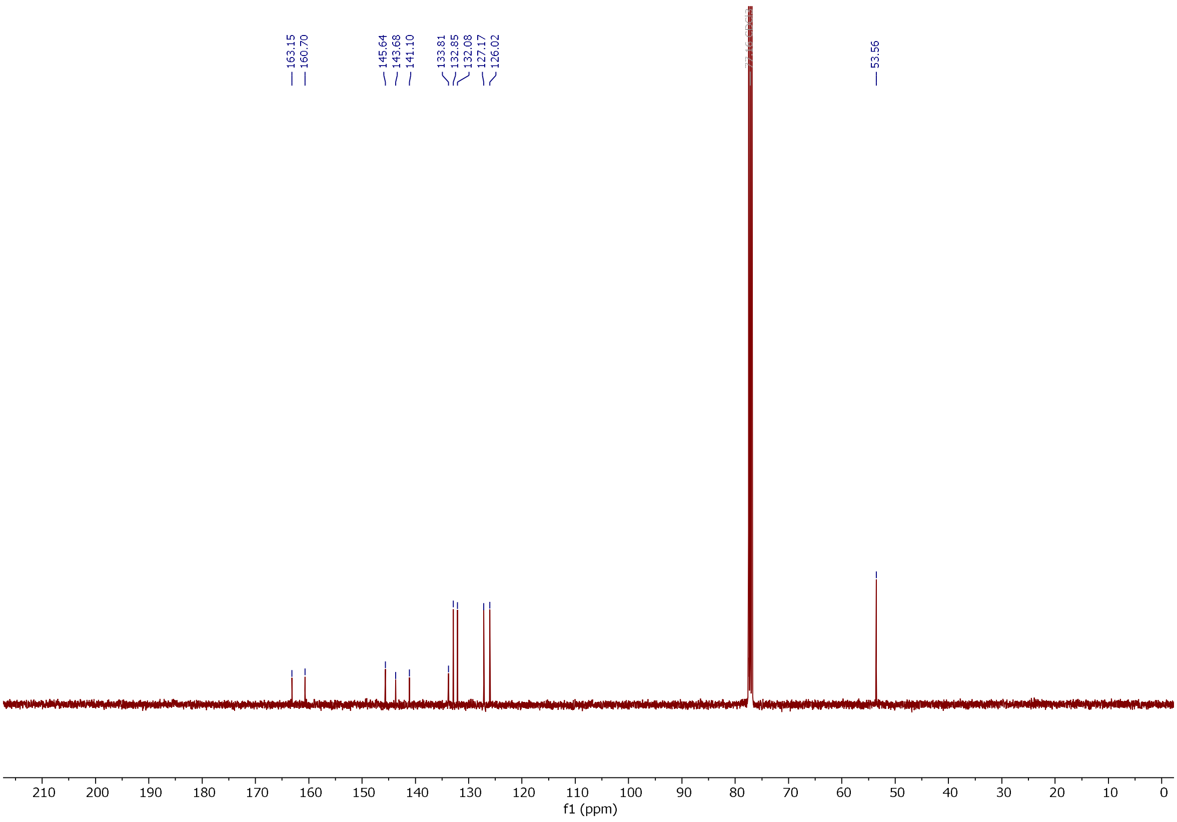

2w ^{1}H NMR (400 MHz, $CDCl_3$)

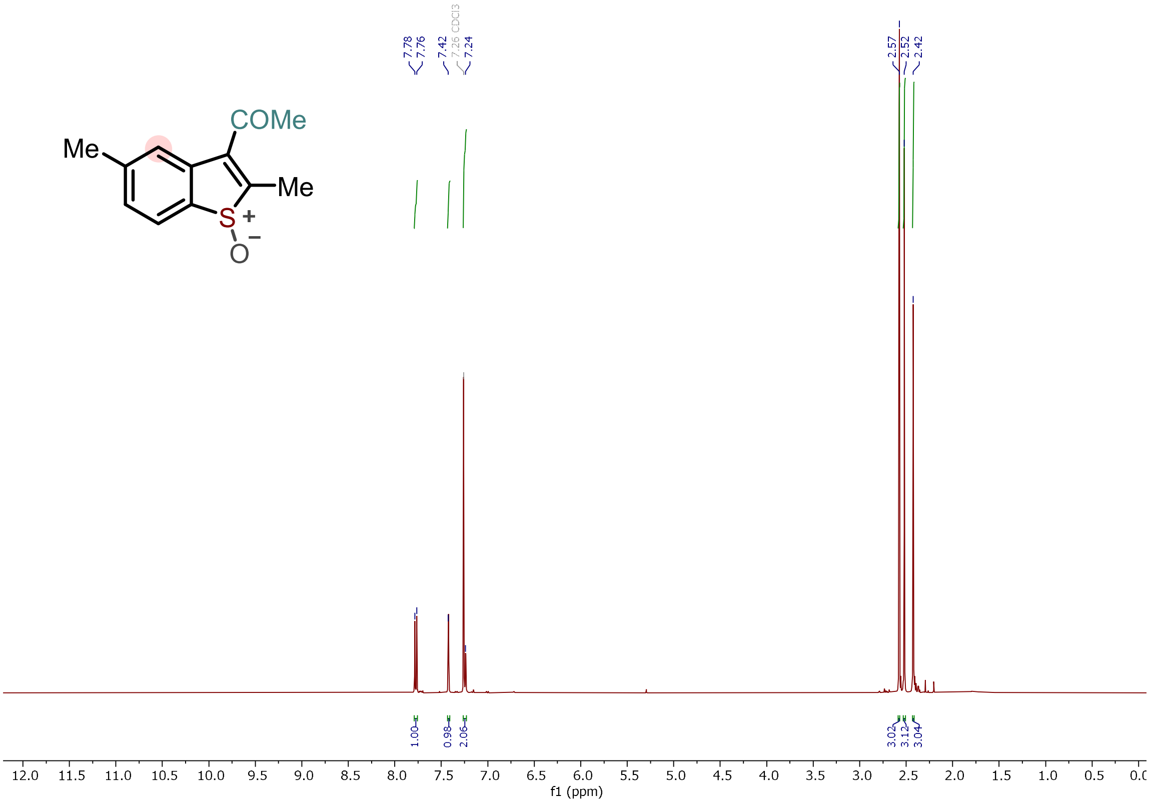

2w ^{13}C NMR (101 MHz, $CDCl_3$)

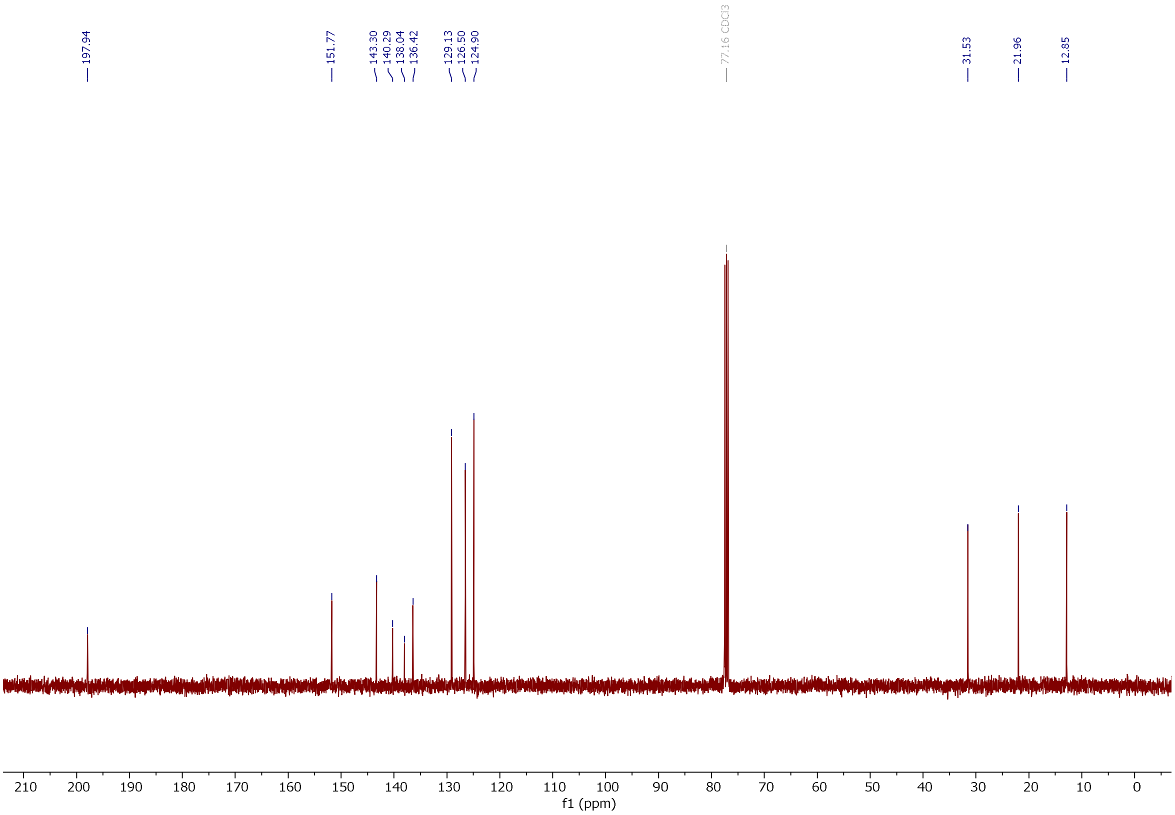

2x ^{1}H NMR (400 MHz, $CDCl_3$)

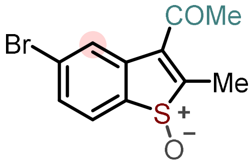

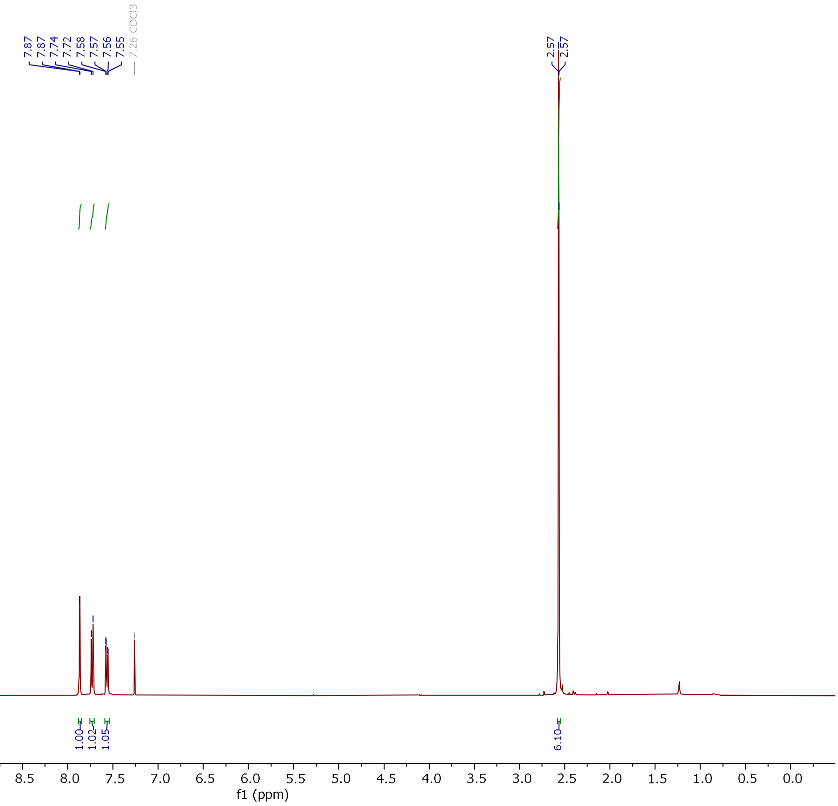

2x ^{13}C NMR (101 MHz, $CDCl_3$)

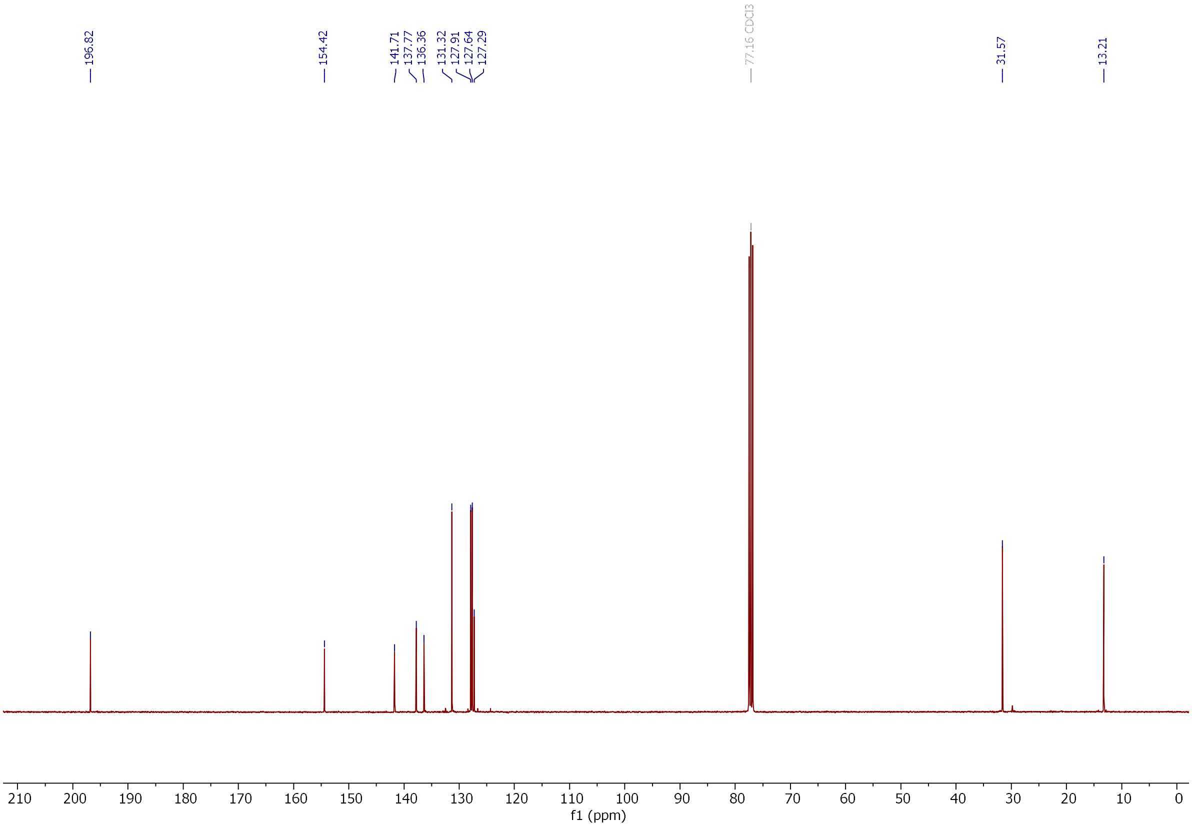

2z ^{1}H NMR (400 MHz, CDCl$_3$)

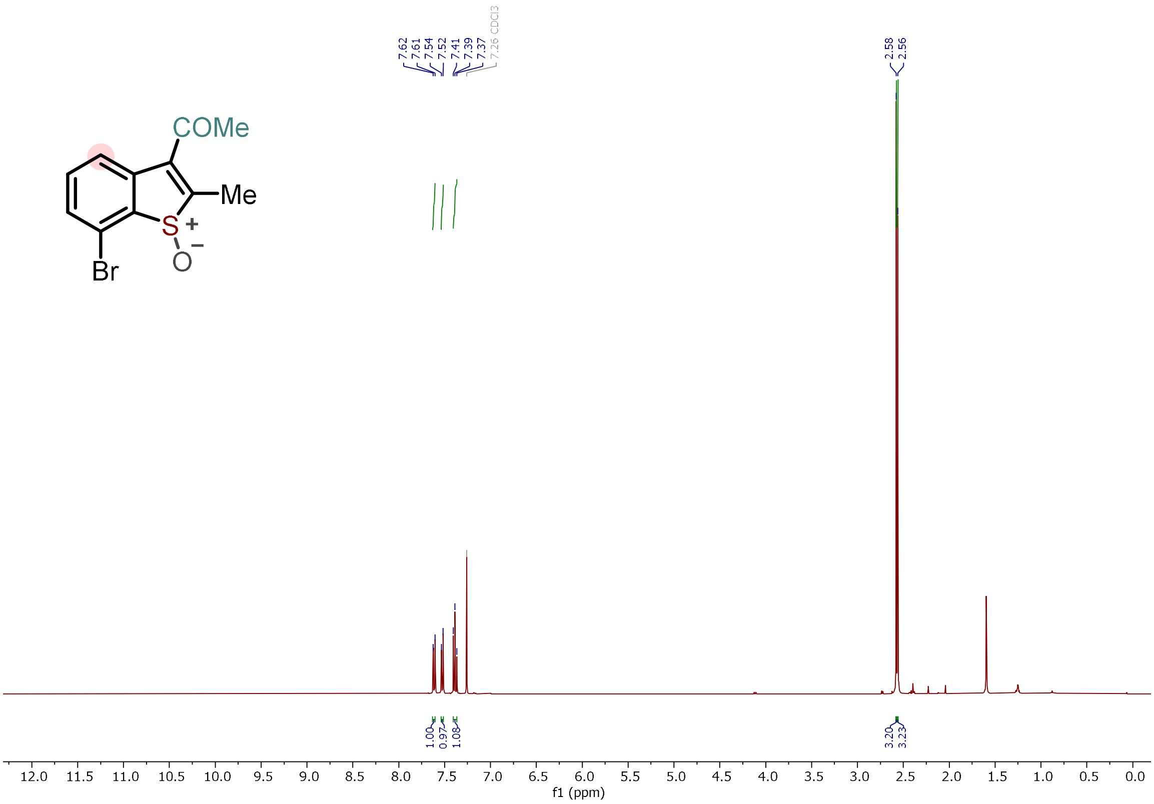

2z ^{13}C NMR (101 MHz, CDCl$_3$)

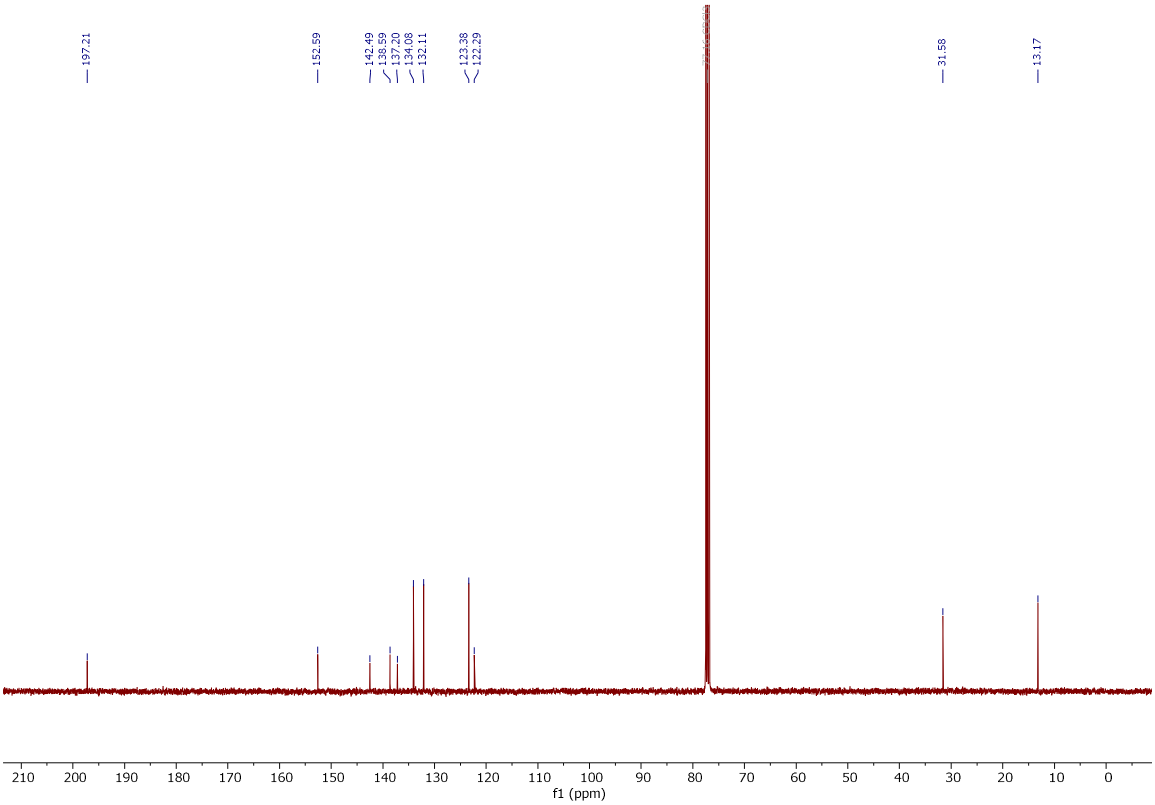

2aa ^{1}H NMR (400 MHz, $CDCl_3$)

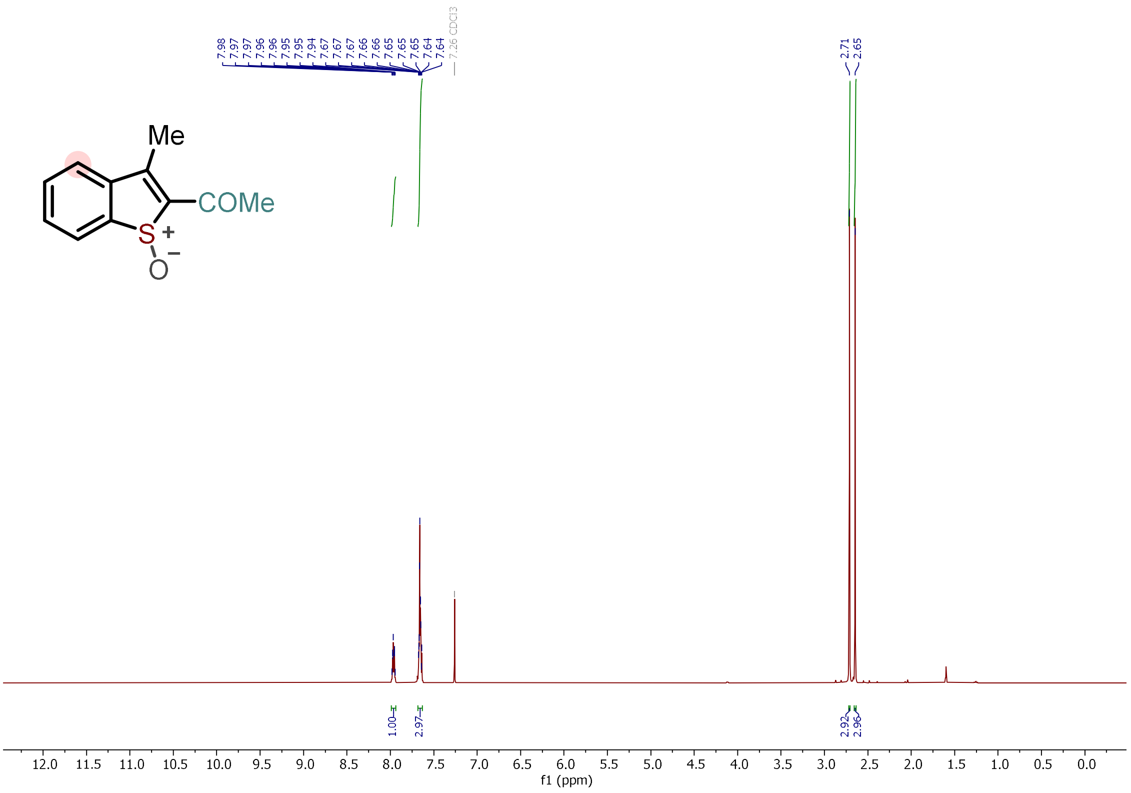

2aa- ^{13}C NMR (101 MHz, $CDCl_3$)

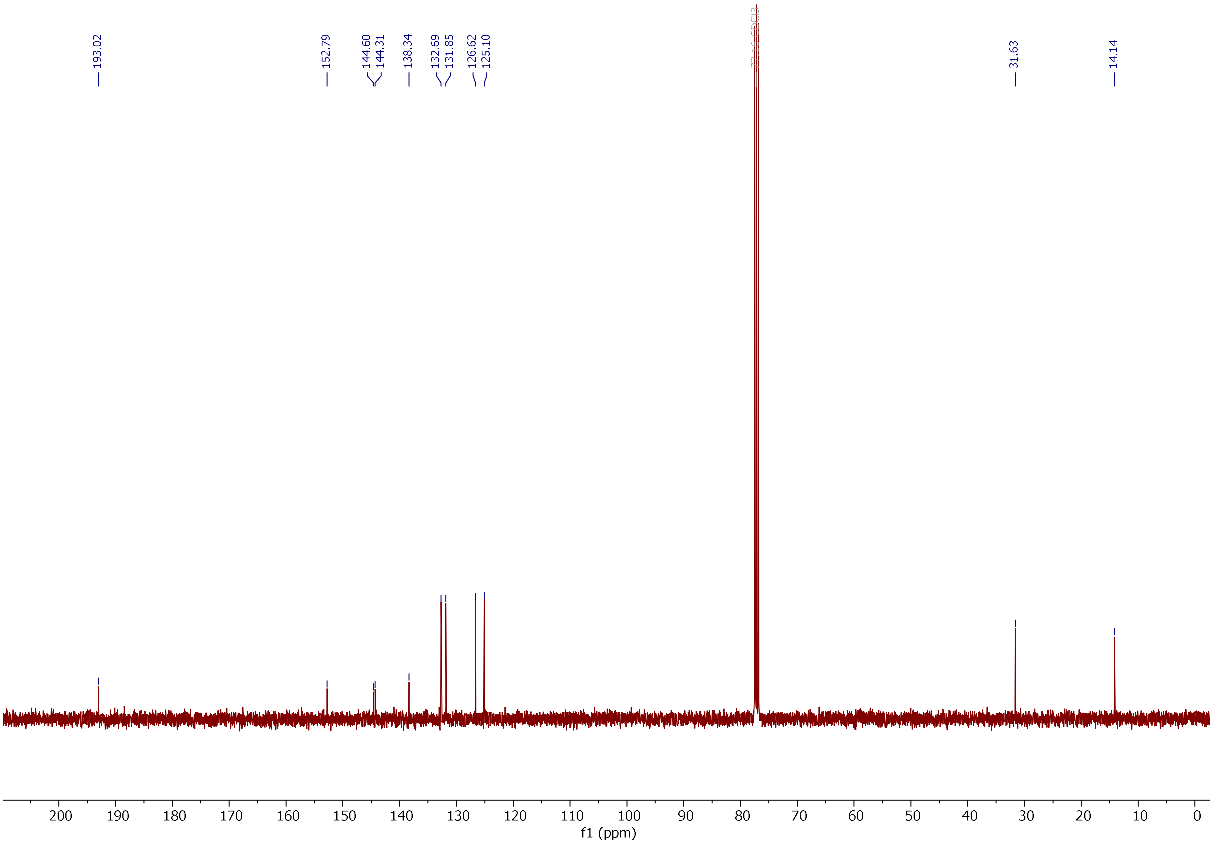

2ab ^{1}H NMR (500 MHz, $CDCl_3$)

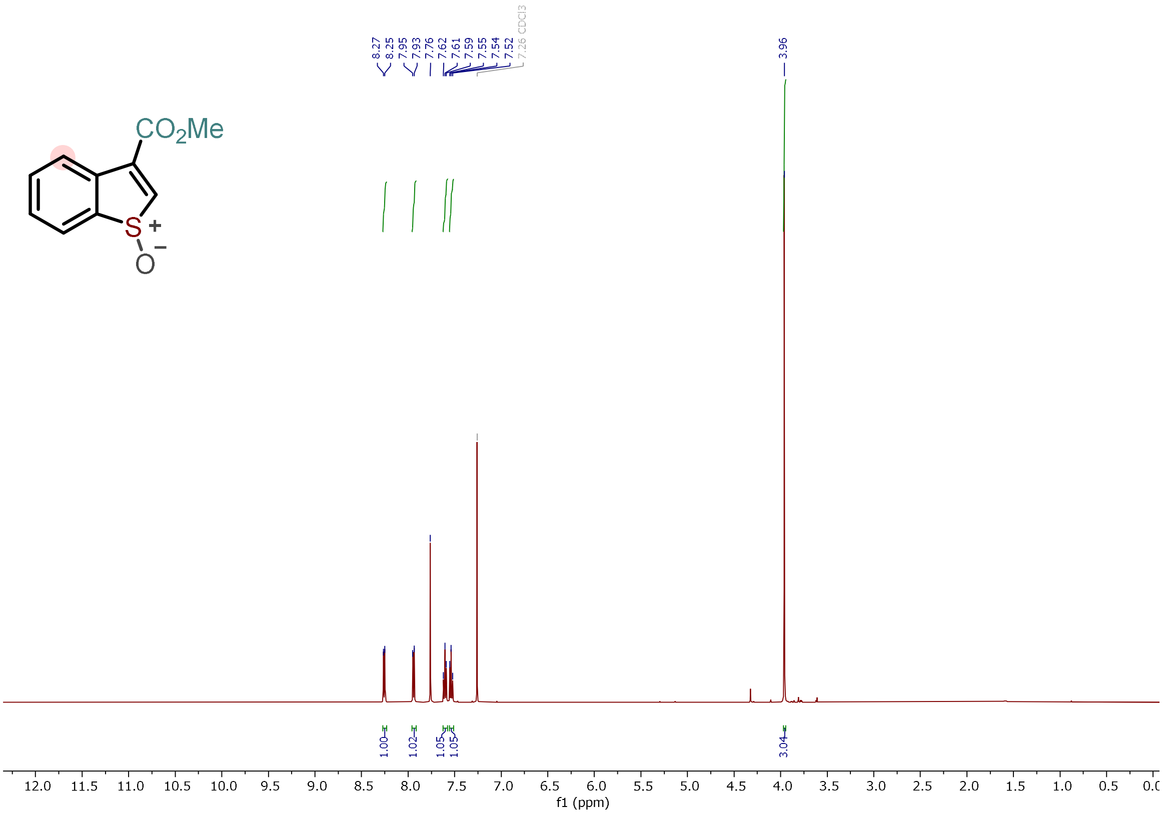

2ab ^{13}C NMR (126 MHz, $CDCl_3$)

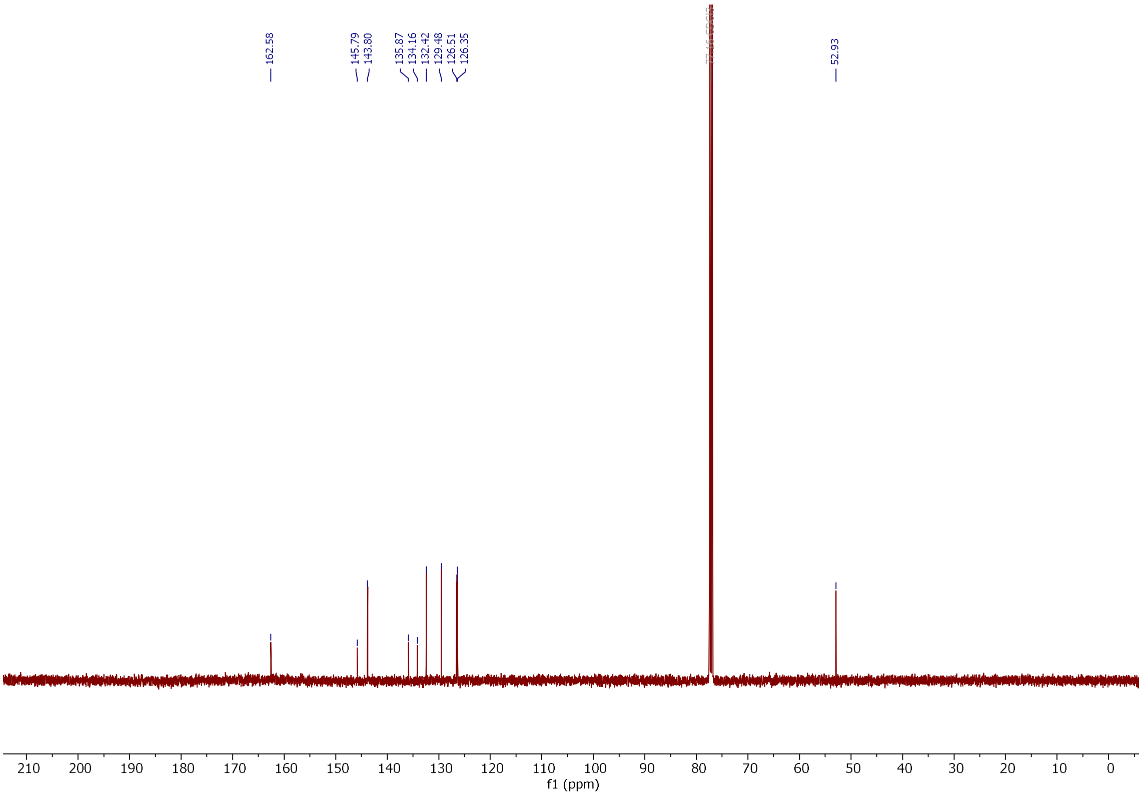

2ah ^{1}H NMR (400 MHz, $CDCl_3$)

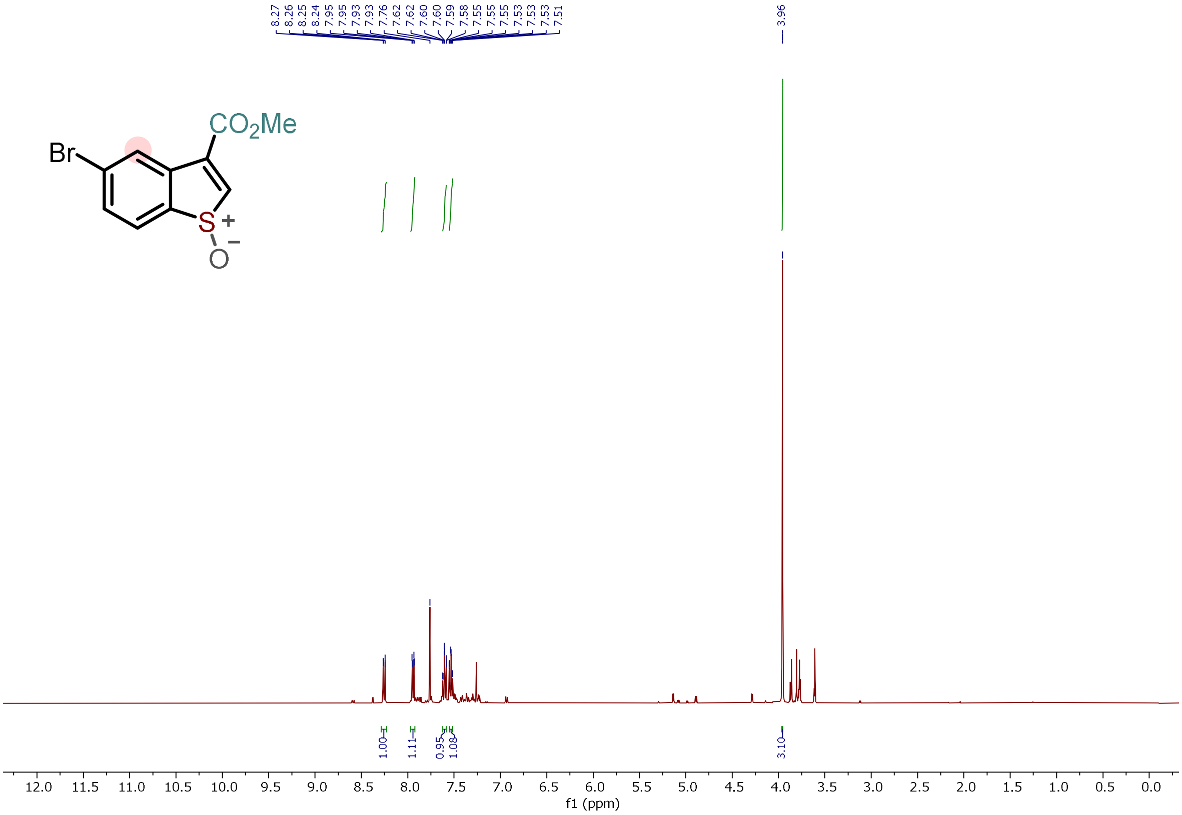

2ah ^{13}C NMR (101 MHz, $CDCl_3$)

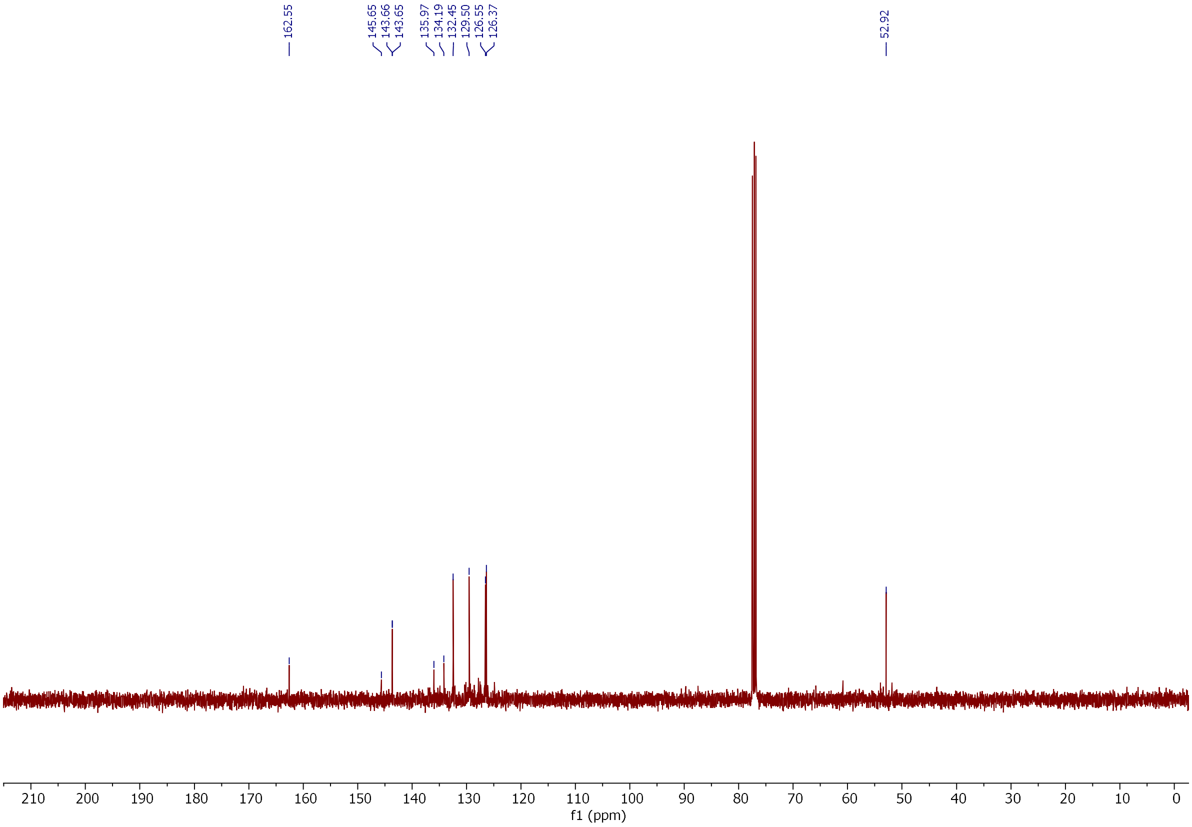

2ai ^{1}H NMR (400 MHz, $CDCl_3$)

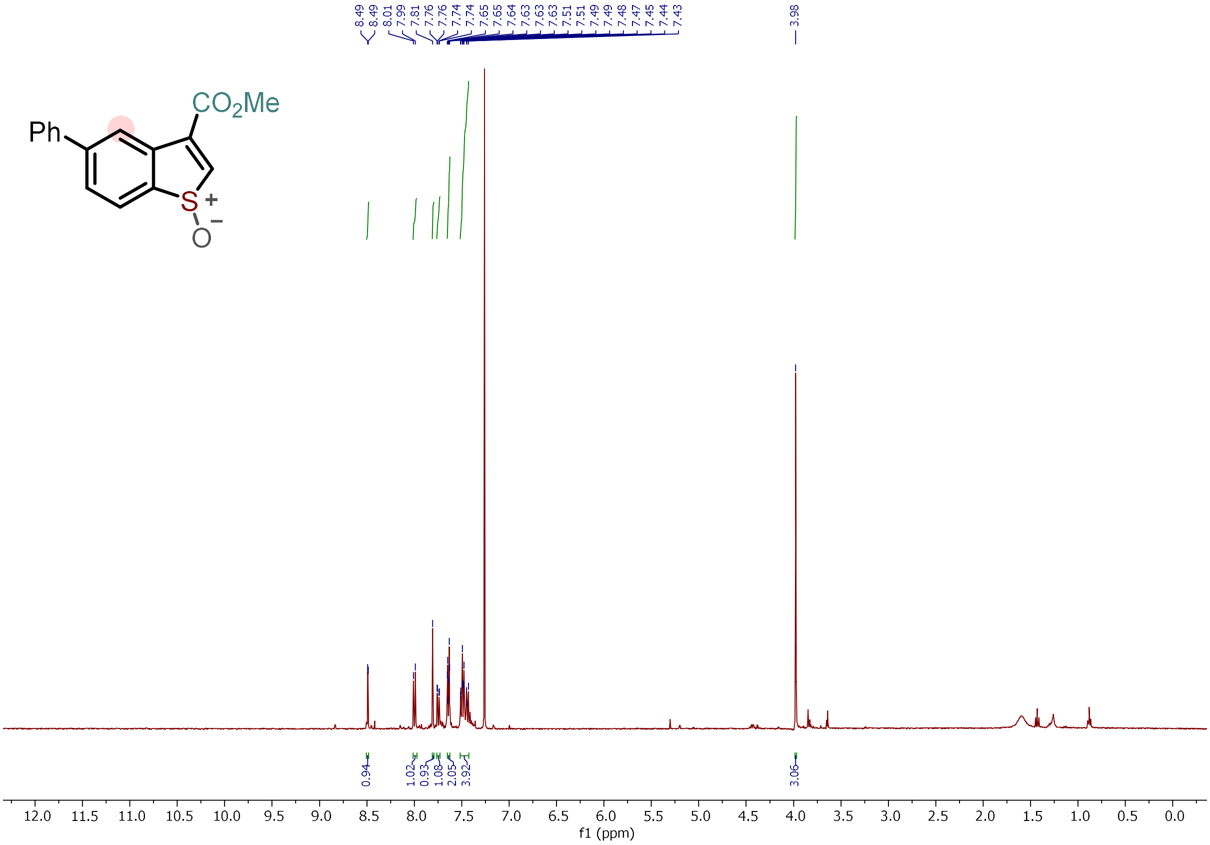

2aj ^{1}H NMR (400 MHz, $CDCl_3$)

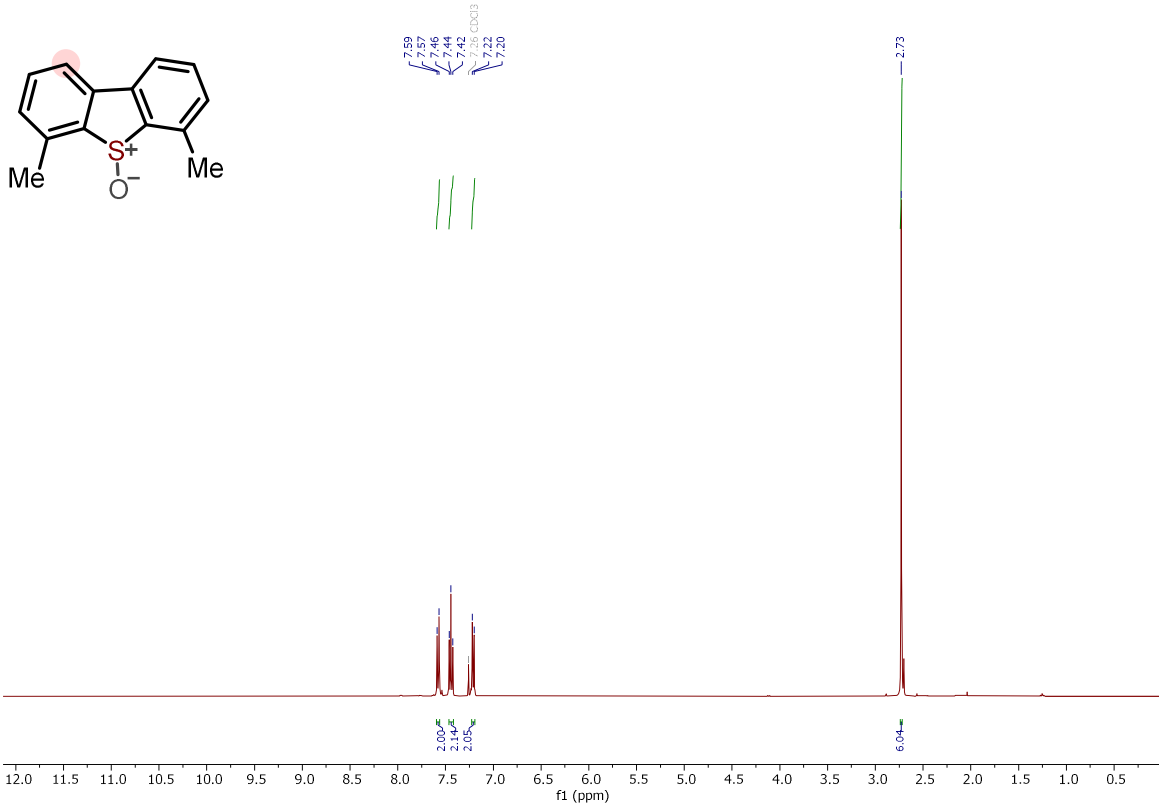

2aj ^{13}C NMR (101 MHz, $CDCl_3$)

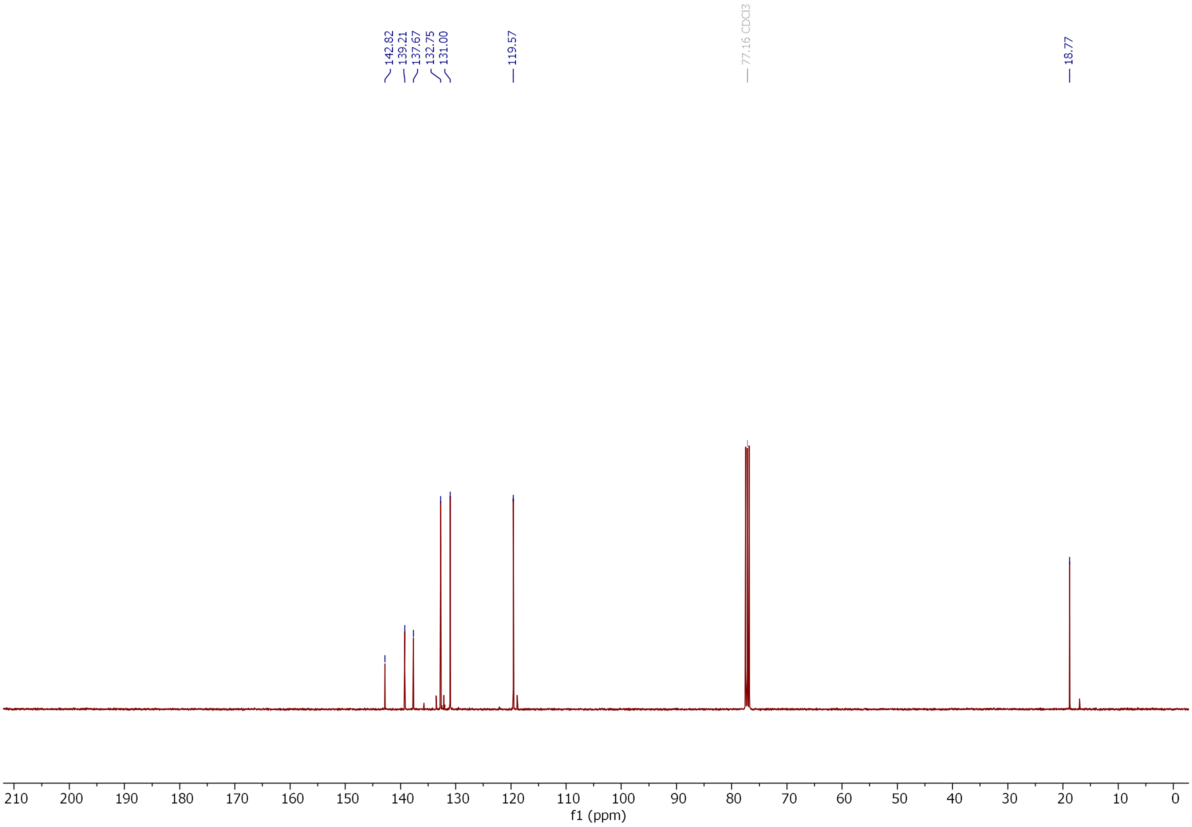

2ao ^{1}H NMR (400 MHz, $CDCl_3$)

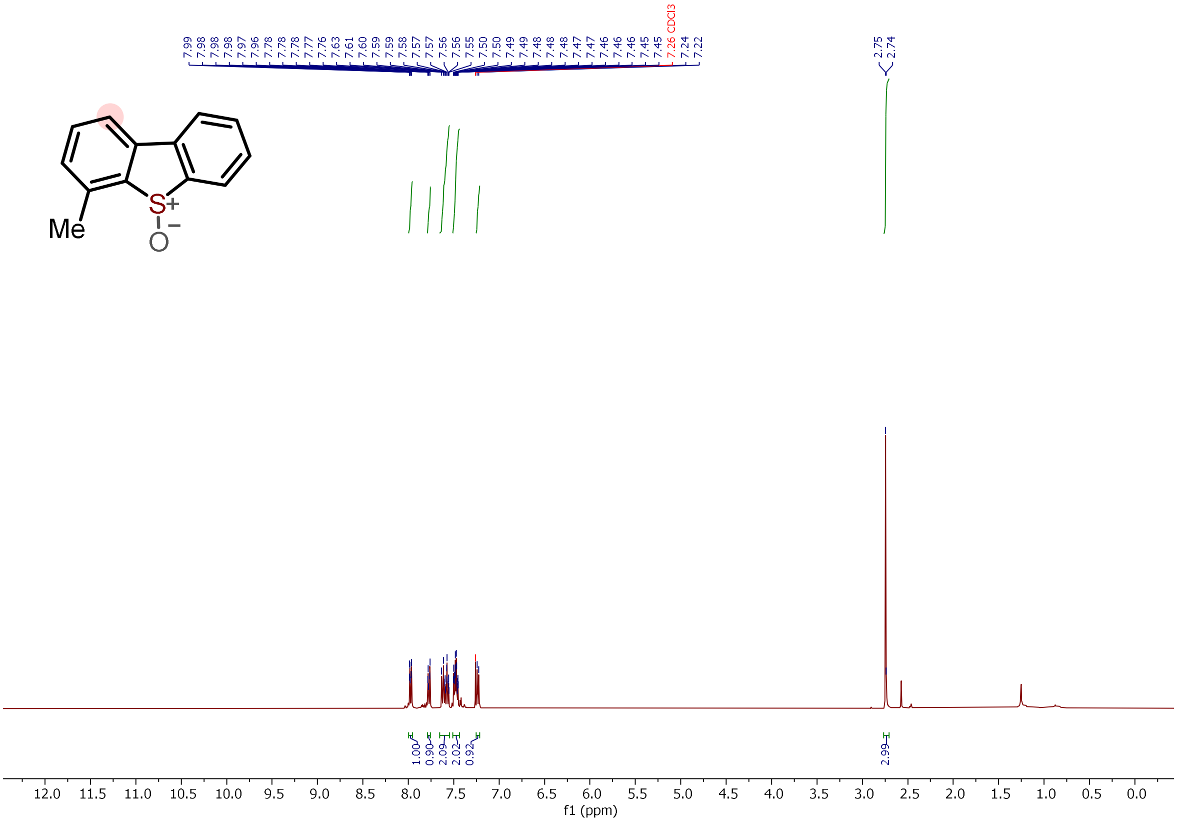

2ao ^{13}C NMR (101 MHz, $CDCl_3$)

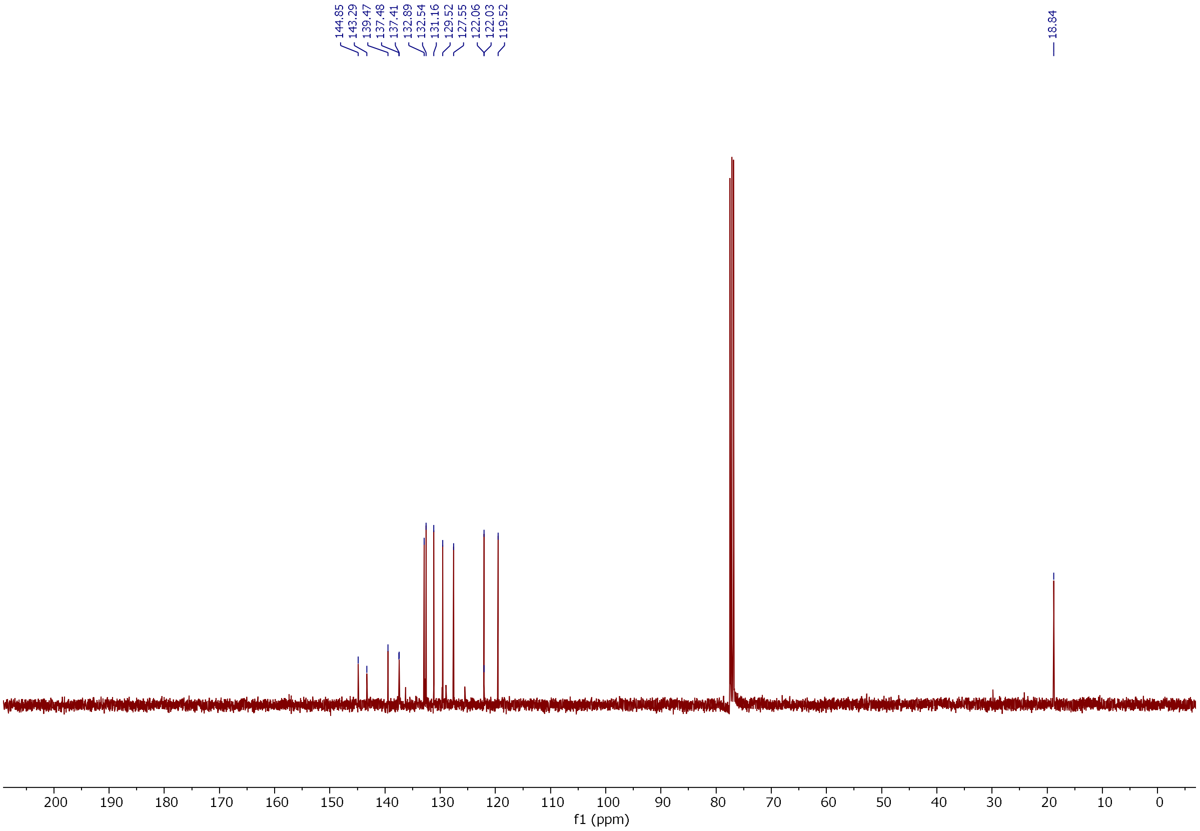

2ap ^{1}H NMR (400 MHz, $CDCl_3$)

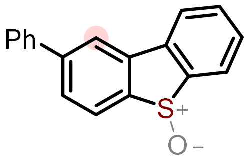

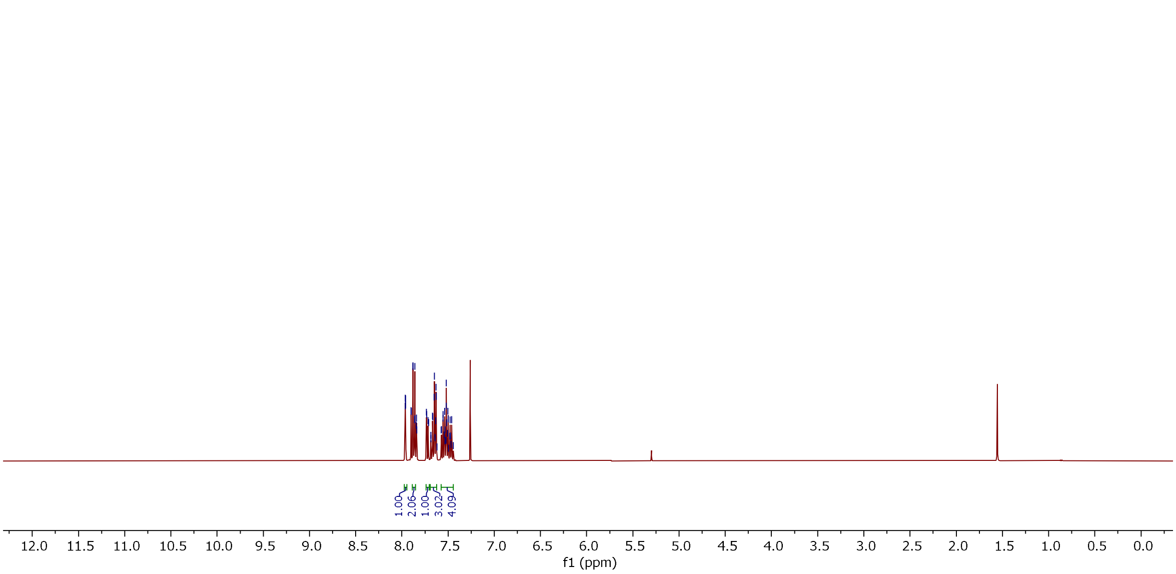

2ap ^{13}C NMR (101 MHz, $CDCl_3$)

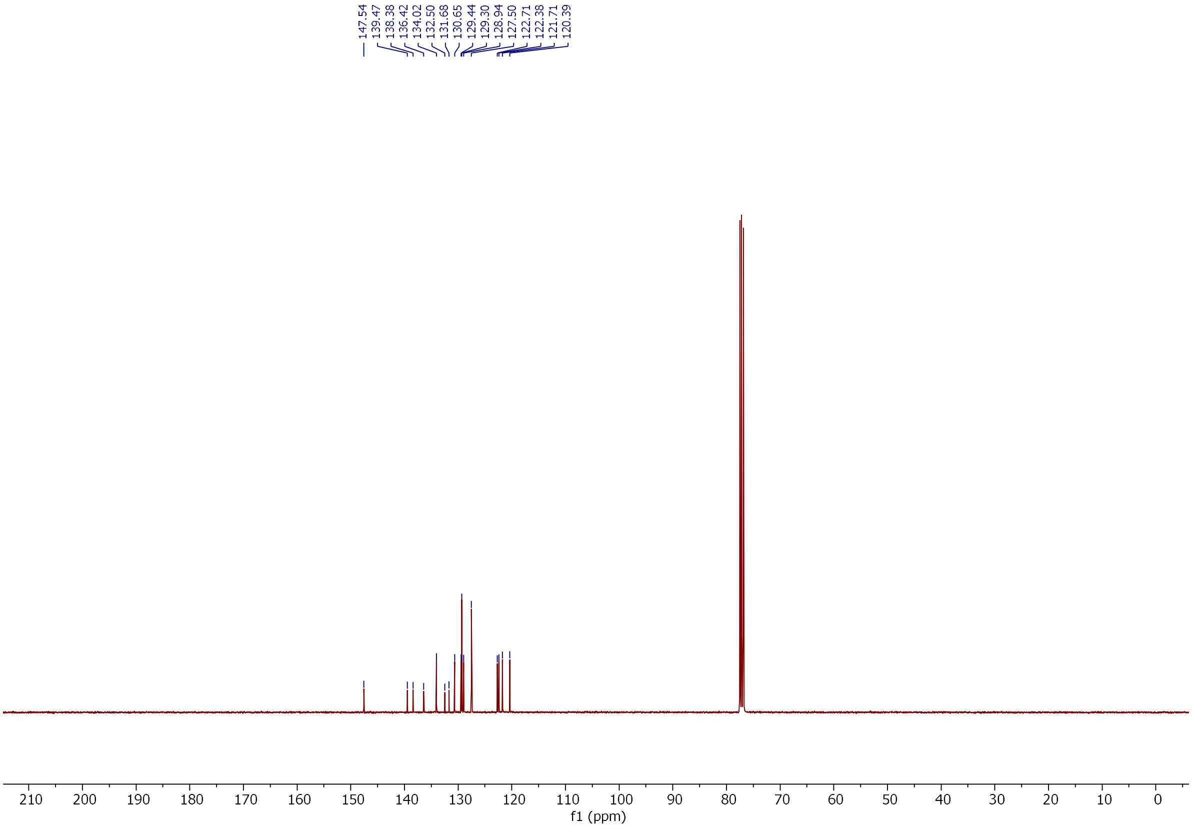

2aq ^{1}H NMR (400 MHz, $CDCl_3$)

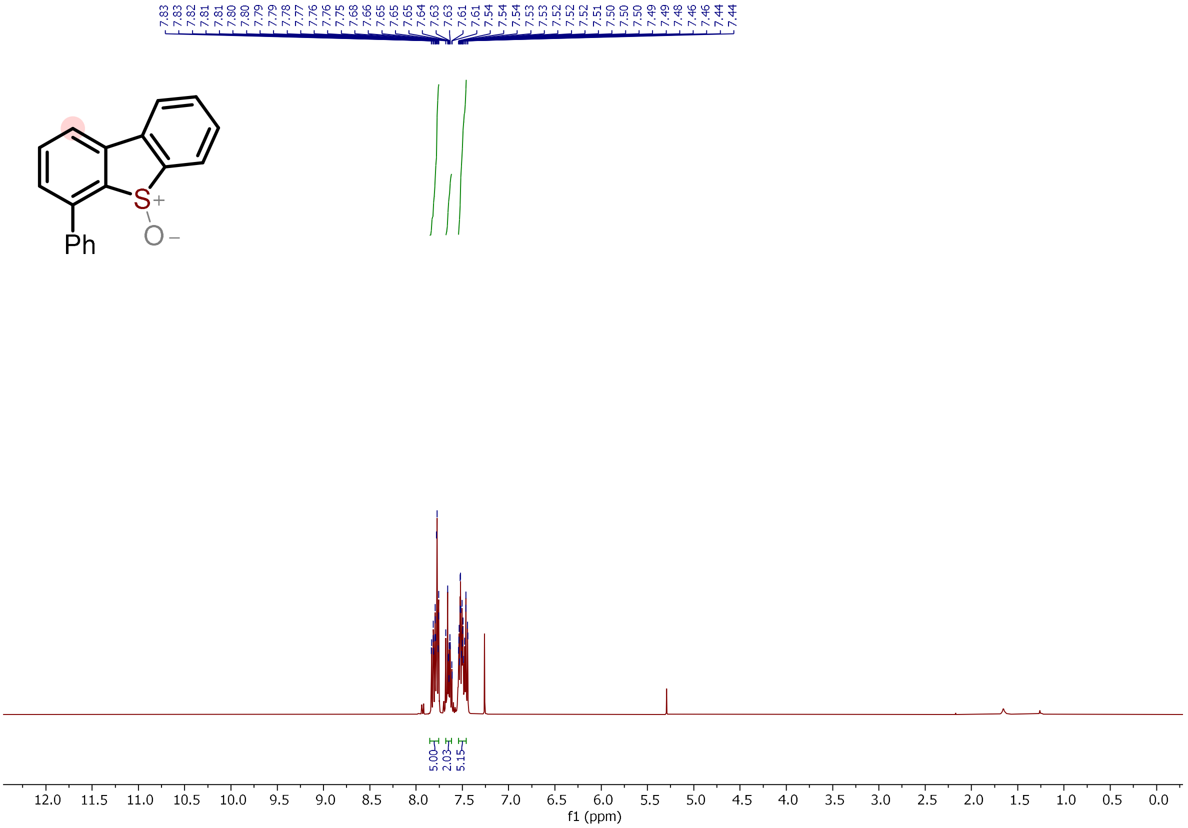

2aq ^{13}C NMR (101 MHz, $CDCl_3$)

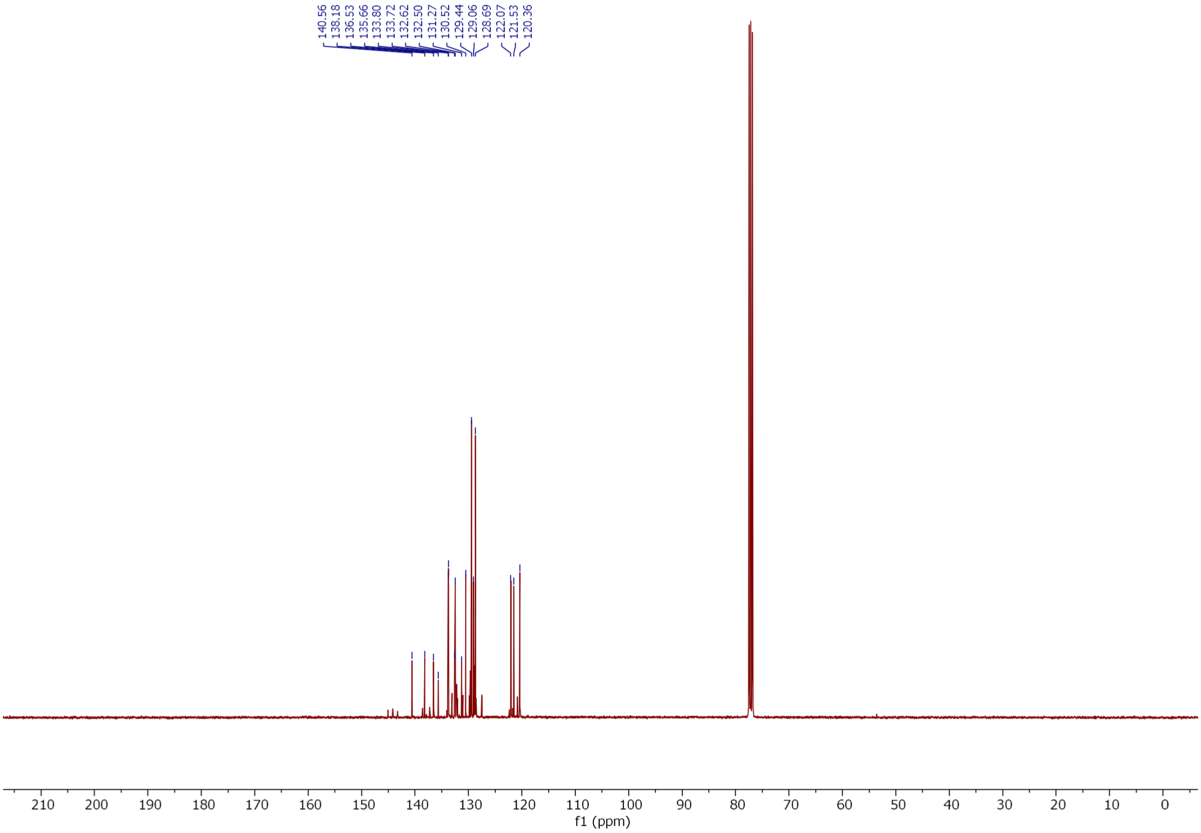

4a ^{1}H NMR (400 MHz, $CDCl_3$)

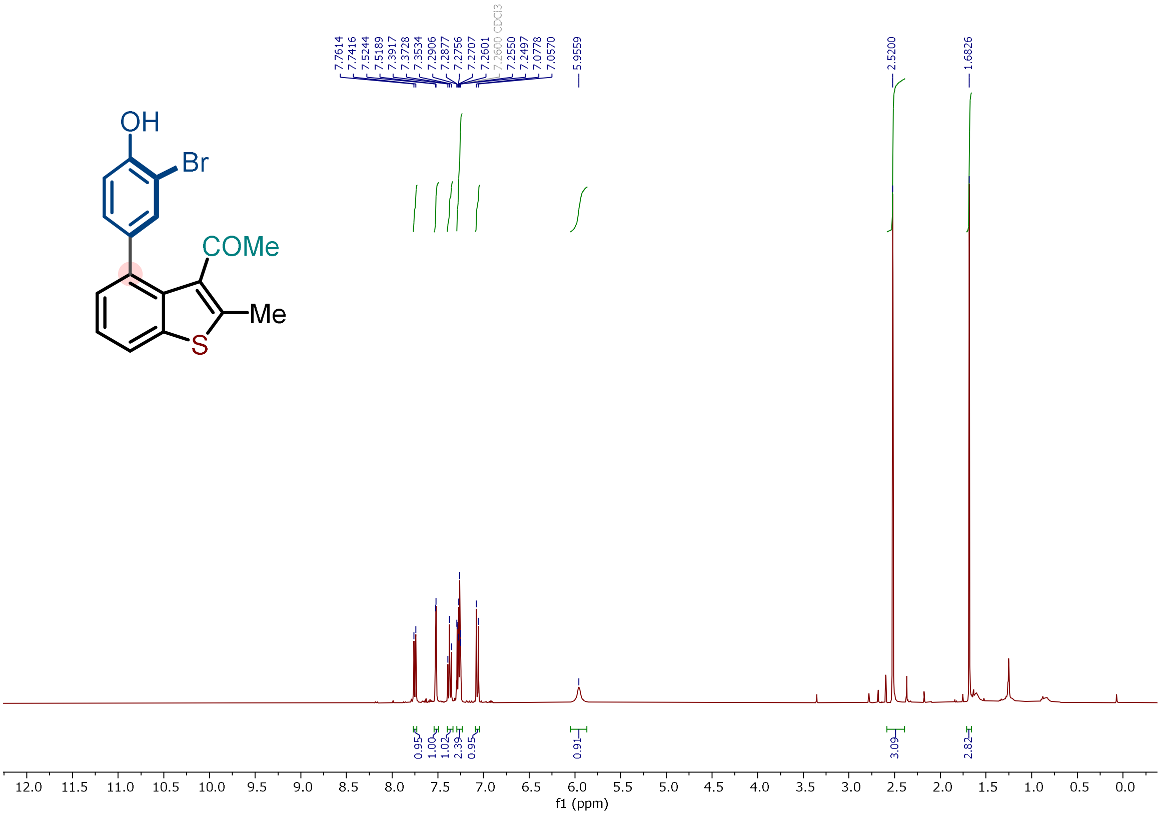

4a ^{13}C NMR (101 MHz, $CDCl_3$)

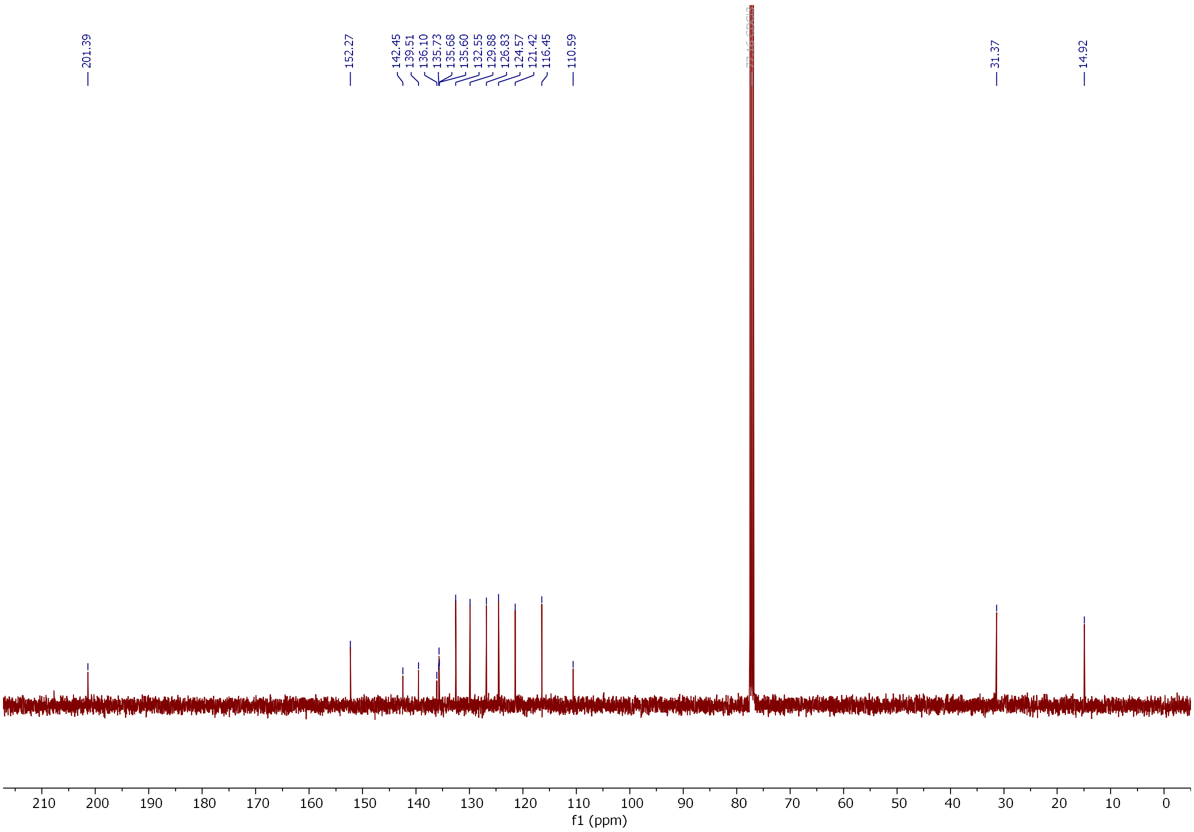

4b ^{1}H NMR (400 MHz, $CDCl_3$)

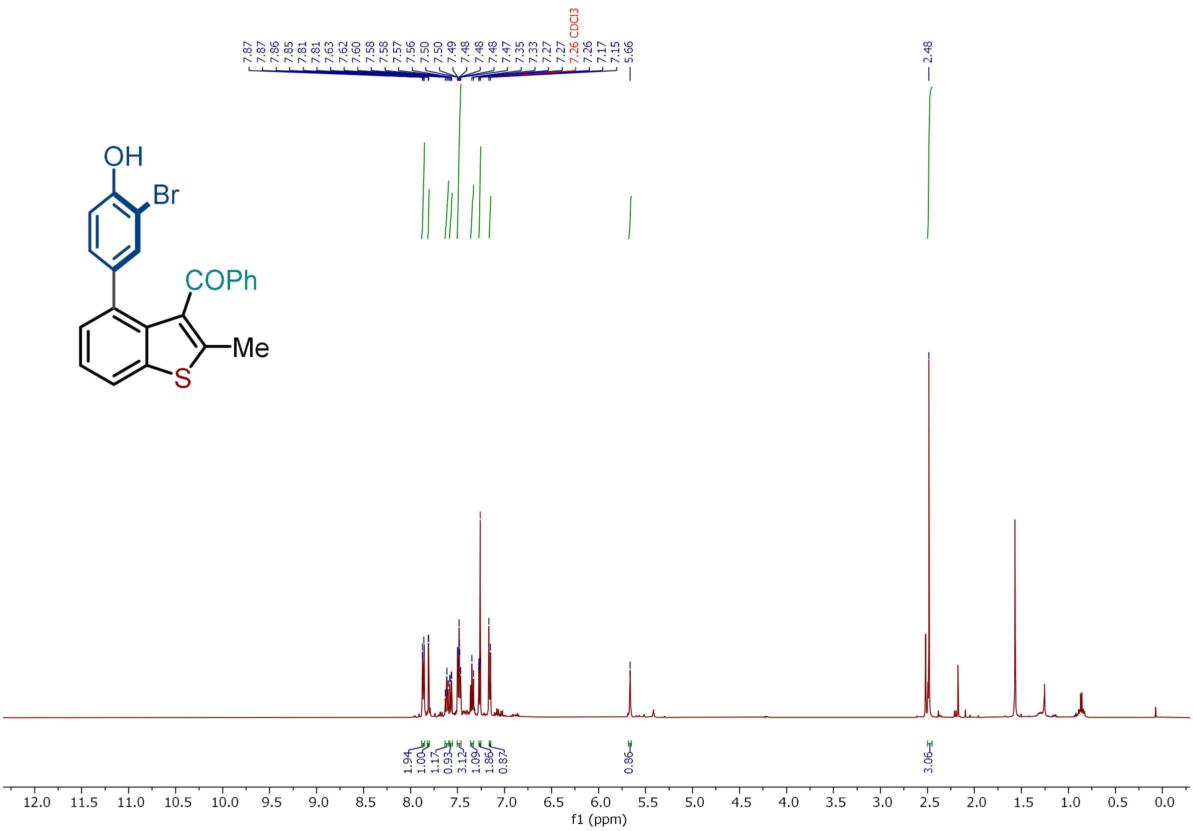

4b ^{13}C NMR (101 MHz, $CDCl_3$)

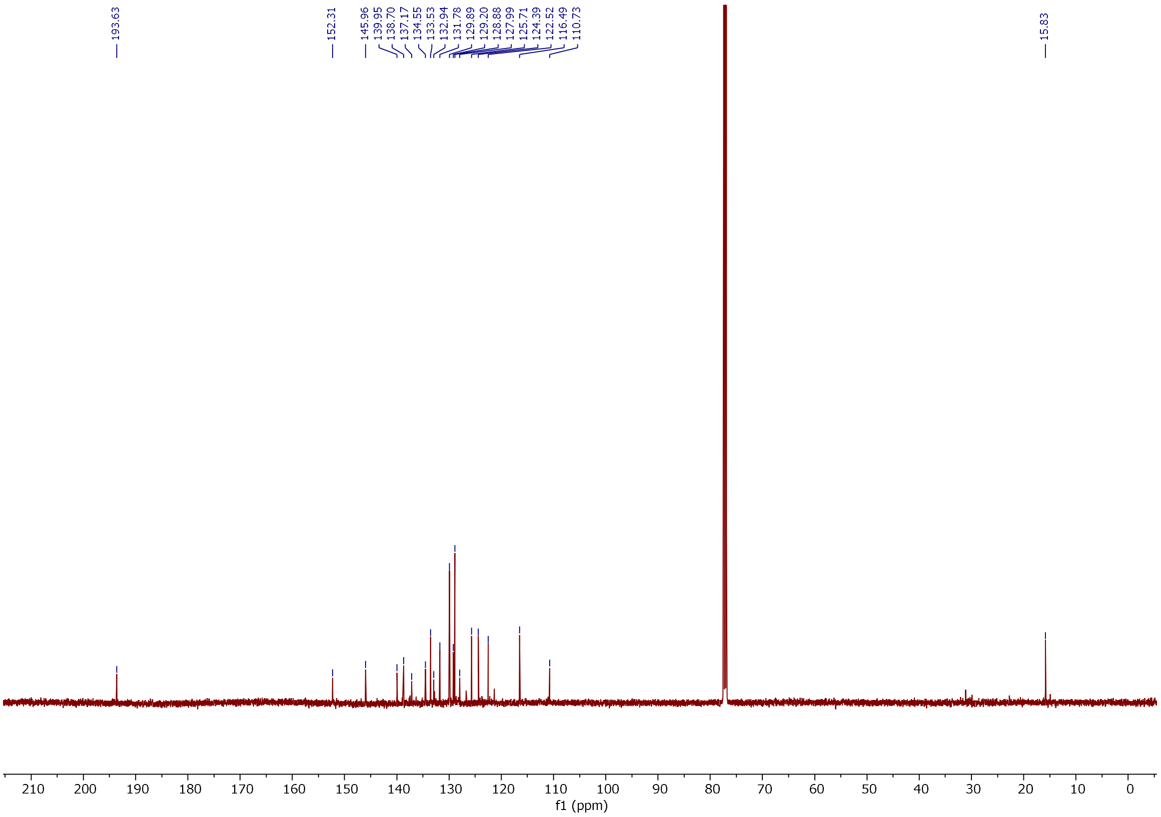

4c ^{1}H NMR (400 MHz, Acetone-d6)

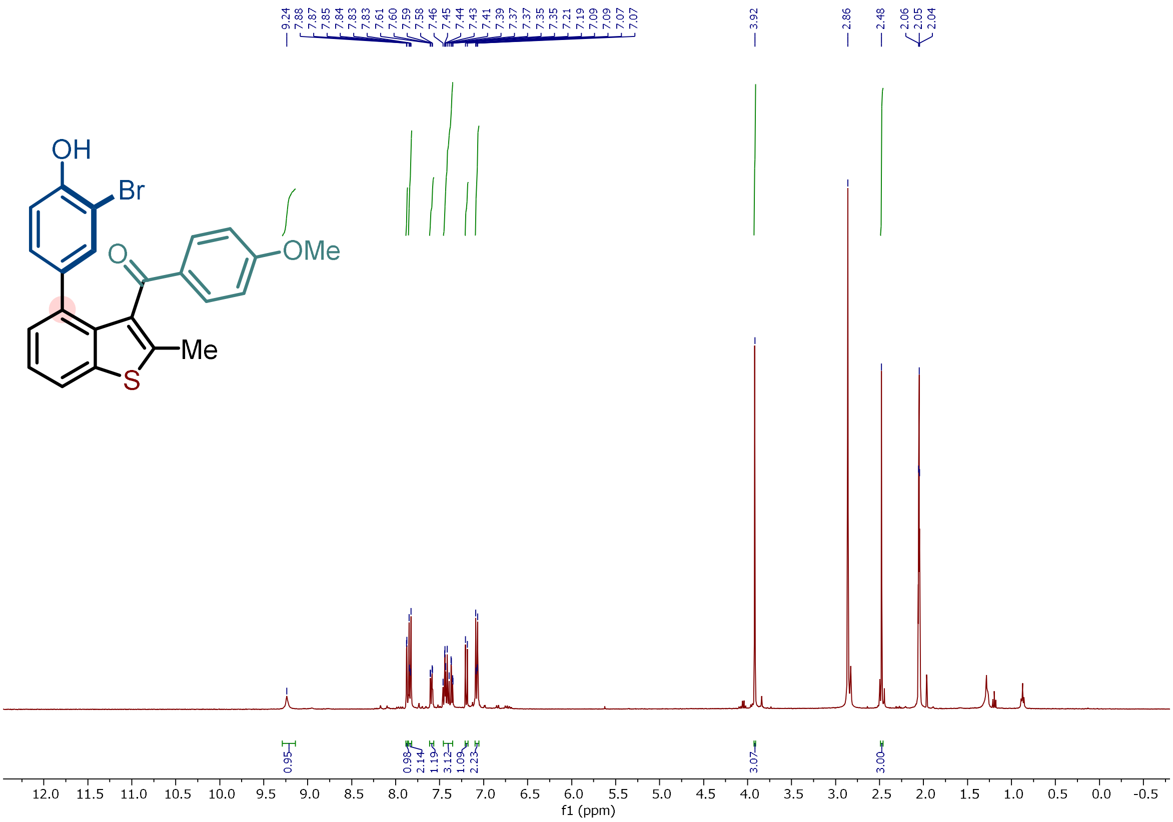

4c ^{13}C NMR (101 MHz, $CDCl_3$)

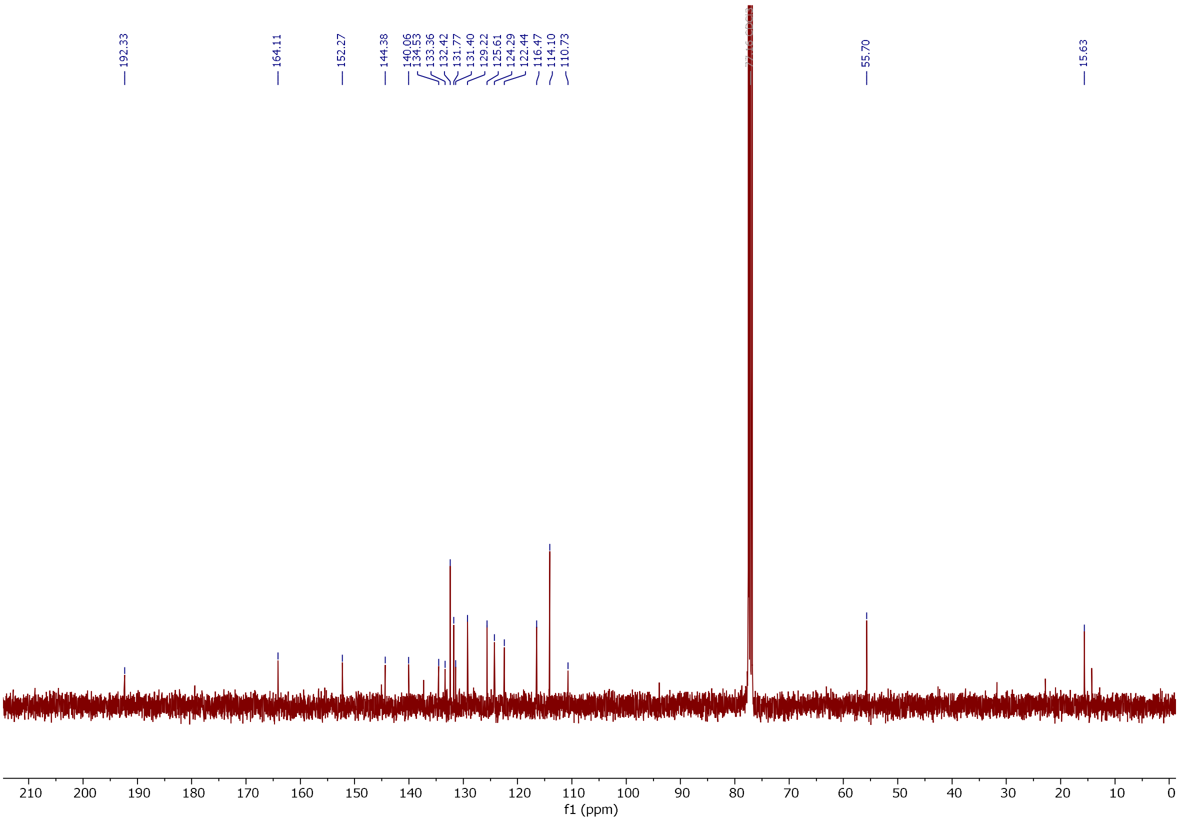

4d ^{1}H NMR (500 MHz, $CDCl_3$)

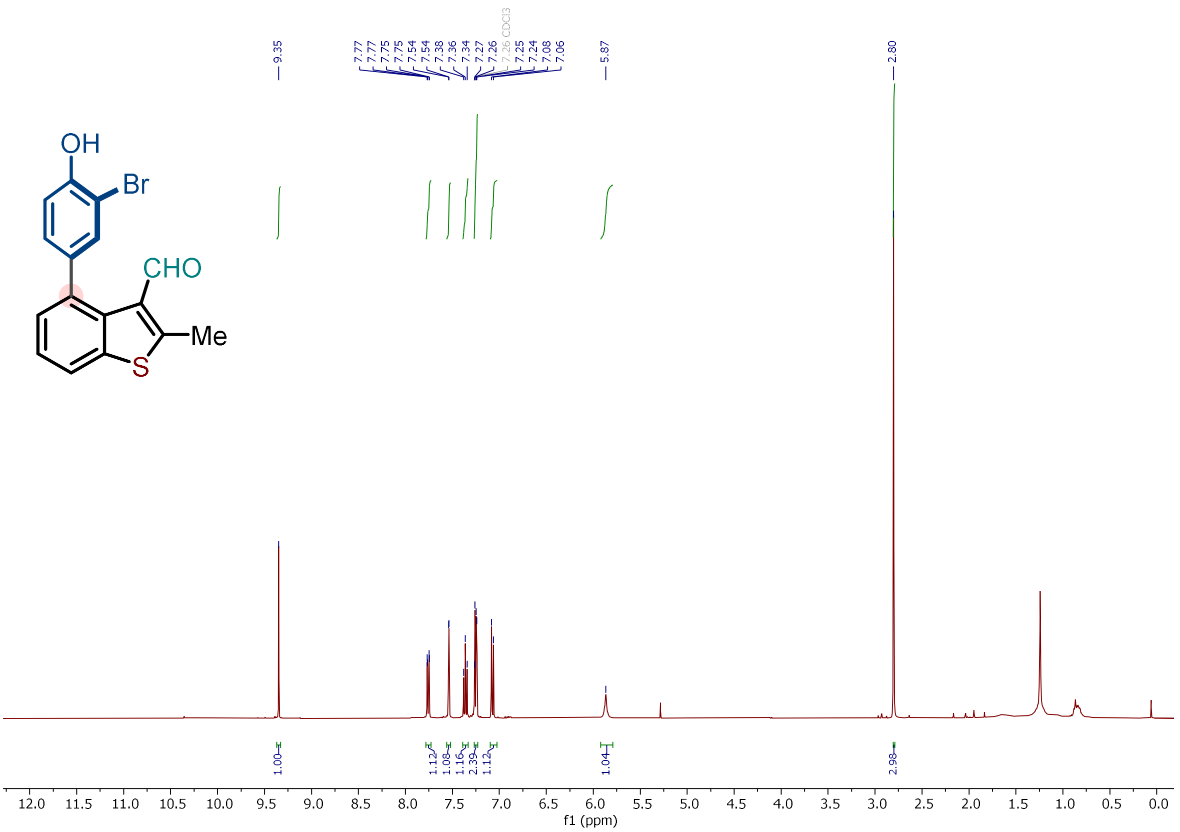

4d ^{13}C NMR (126 MHz, $CDCl_3$)

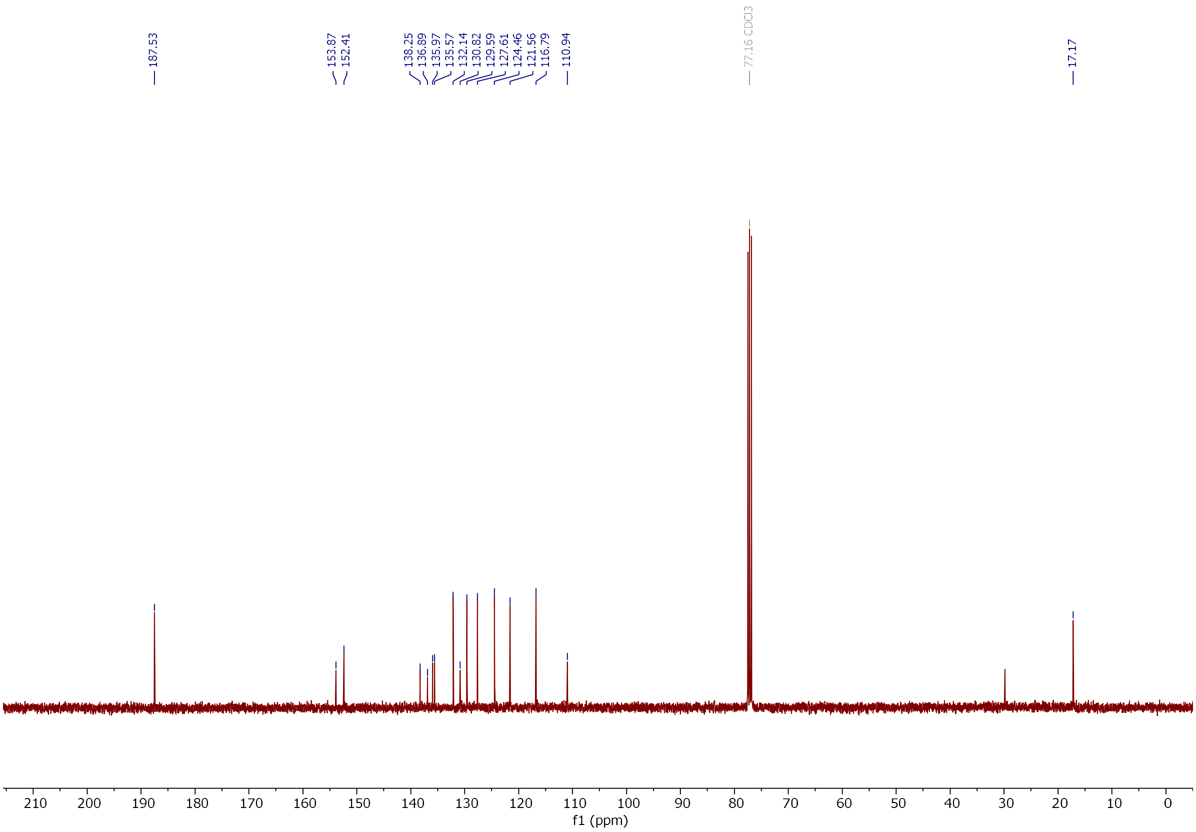

4e ^{1}H NMR (400 MHz, $CDCl_3$)

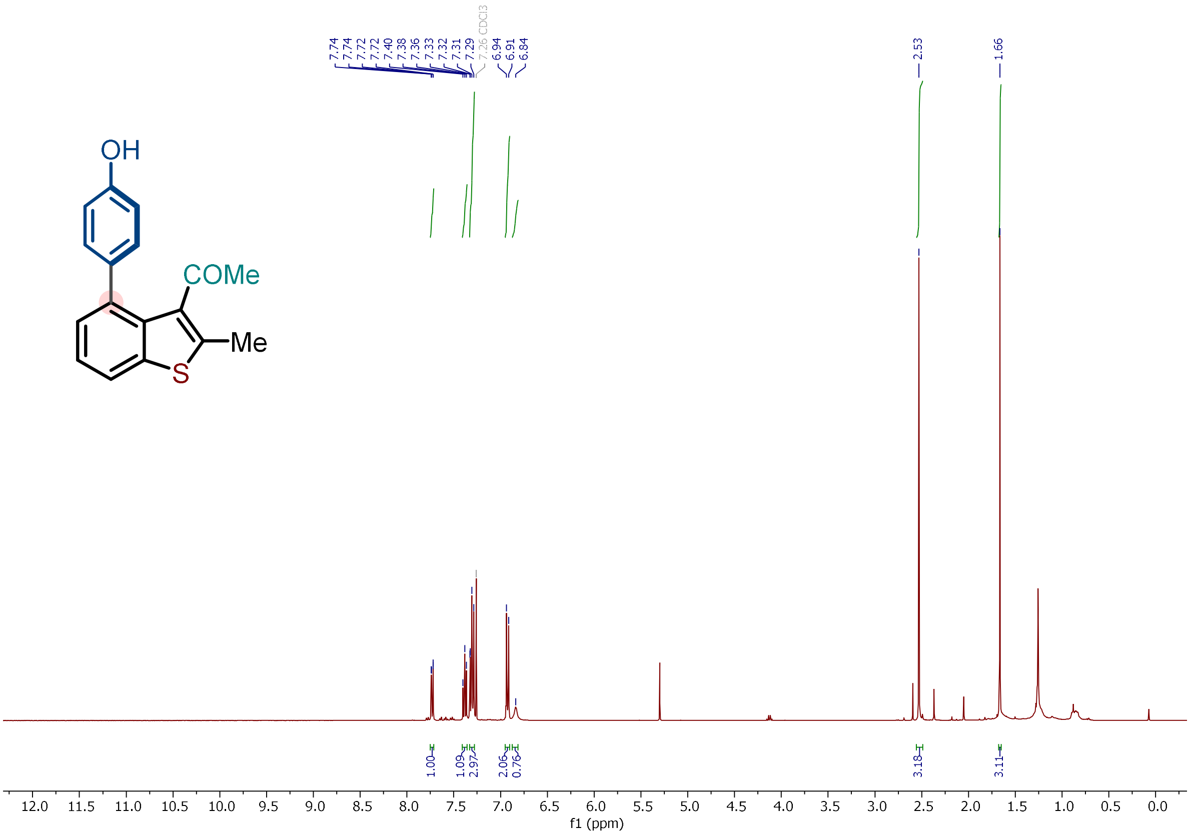

4e ^{13}C NMR (101 MHz, $CDCl_3$)

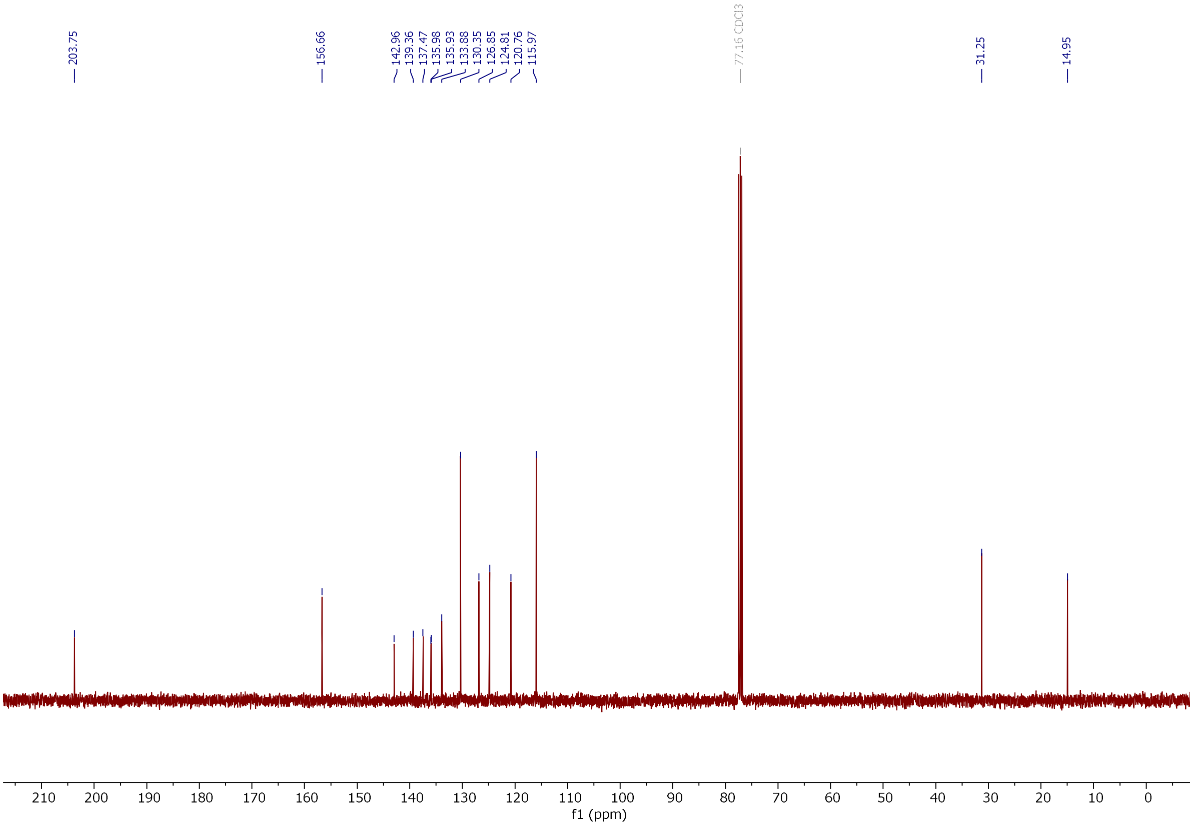

4f ^{1}H NMR (400 MHz, $CDCl_3$)

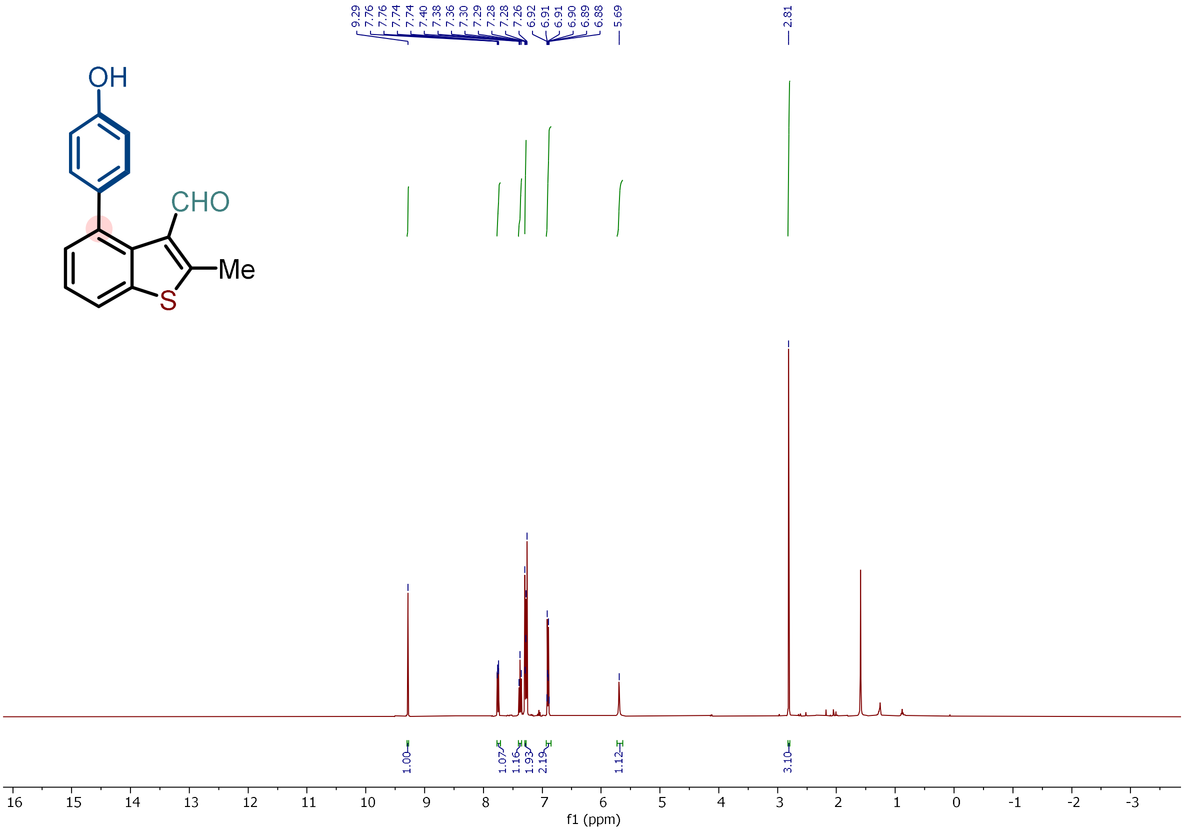

4f ^{13}C NMR (101 MHz, $CDCl_3$)

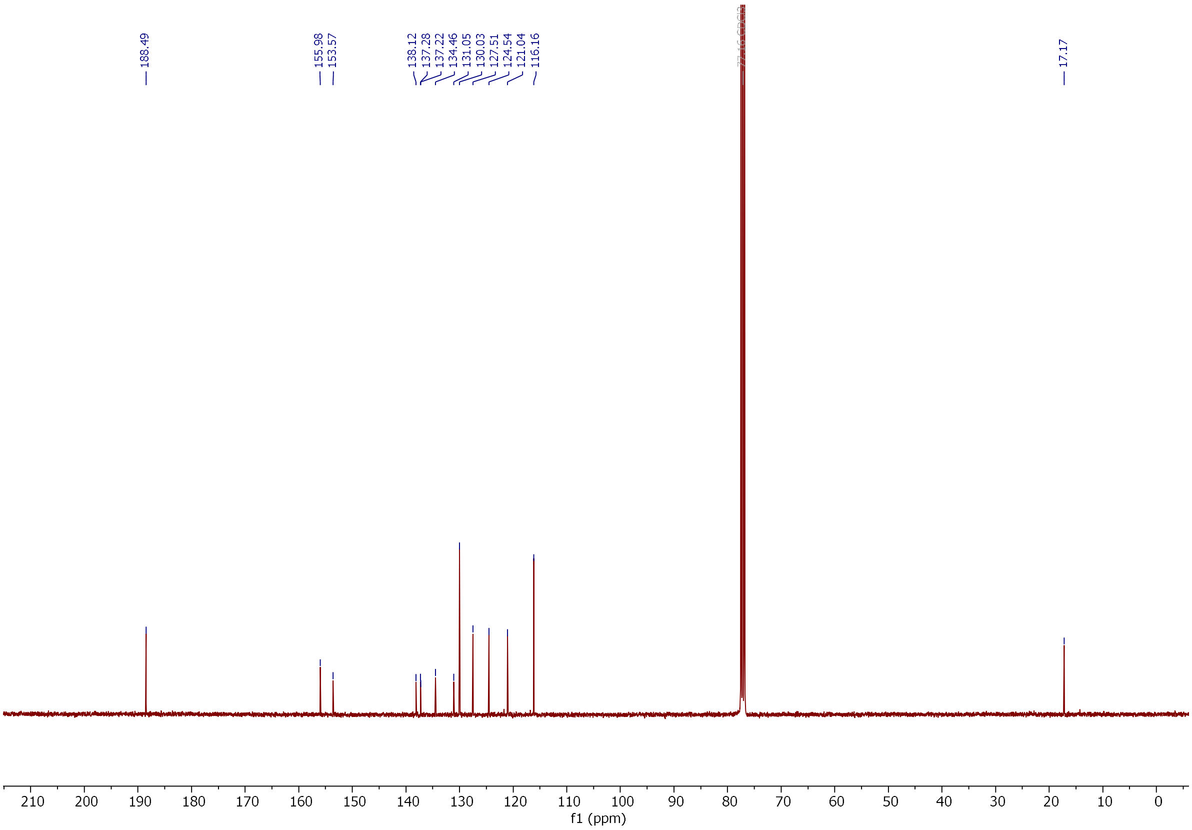

4g ^{1}H NMR (400 MHz, Acetone-d$_6$)

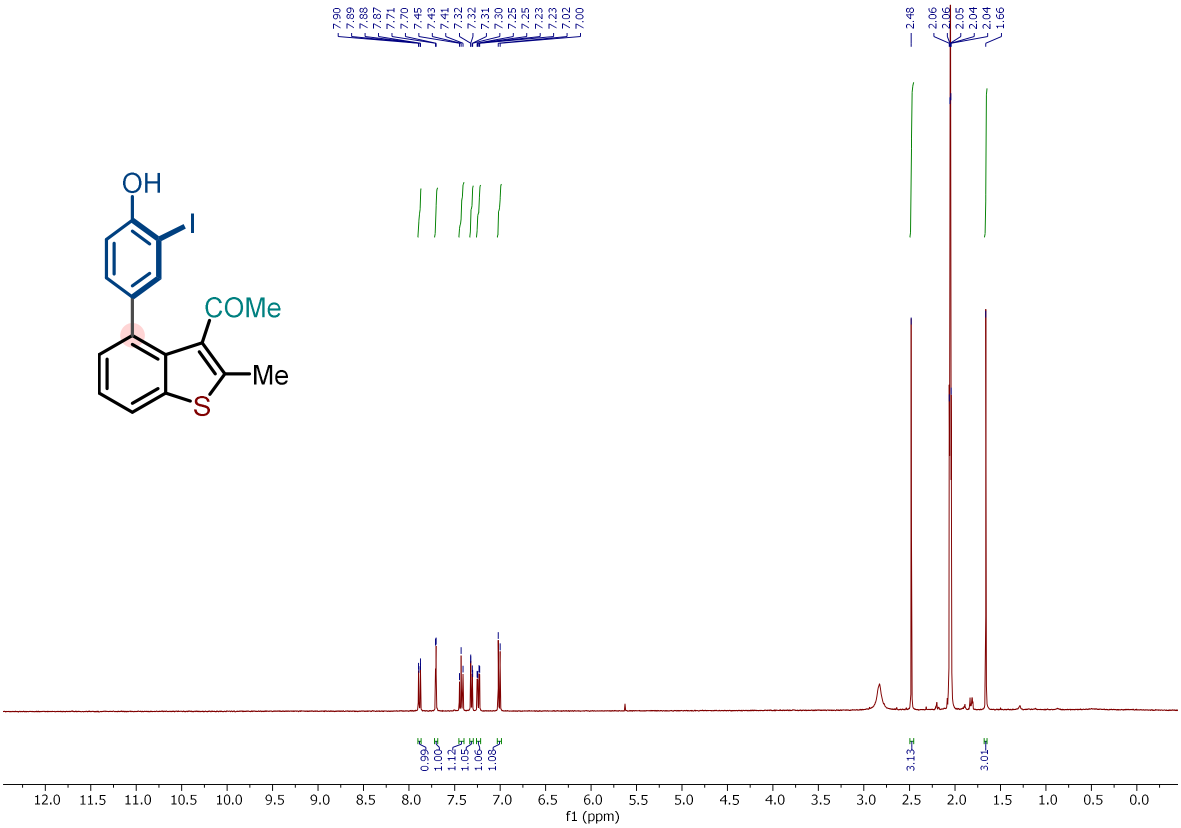

4g ^{13}C NMR (101 MHz, Acetone-d$_6$)

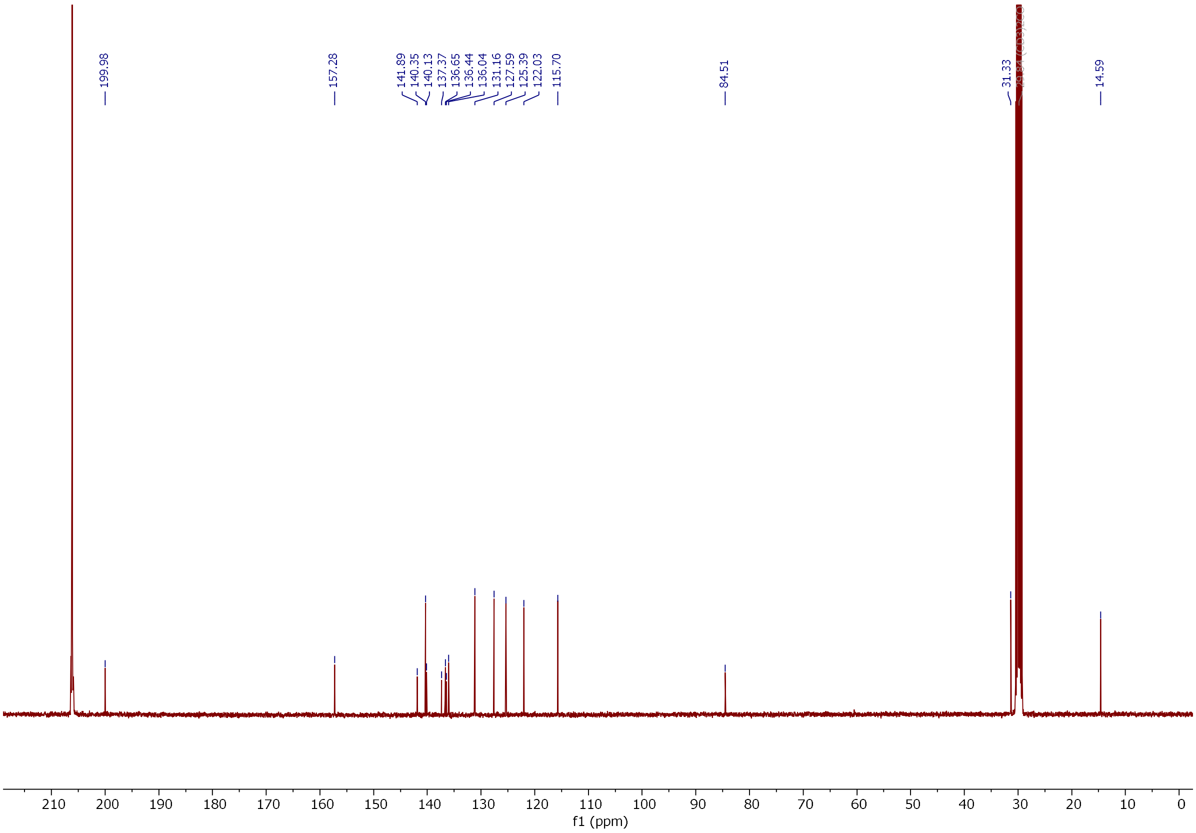

4h ^{1}H NMR (400 MHz, $CDCl_3$)

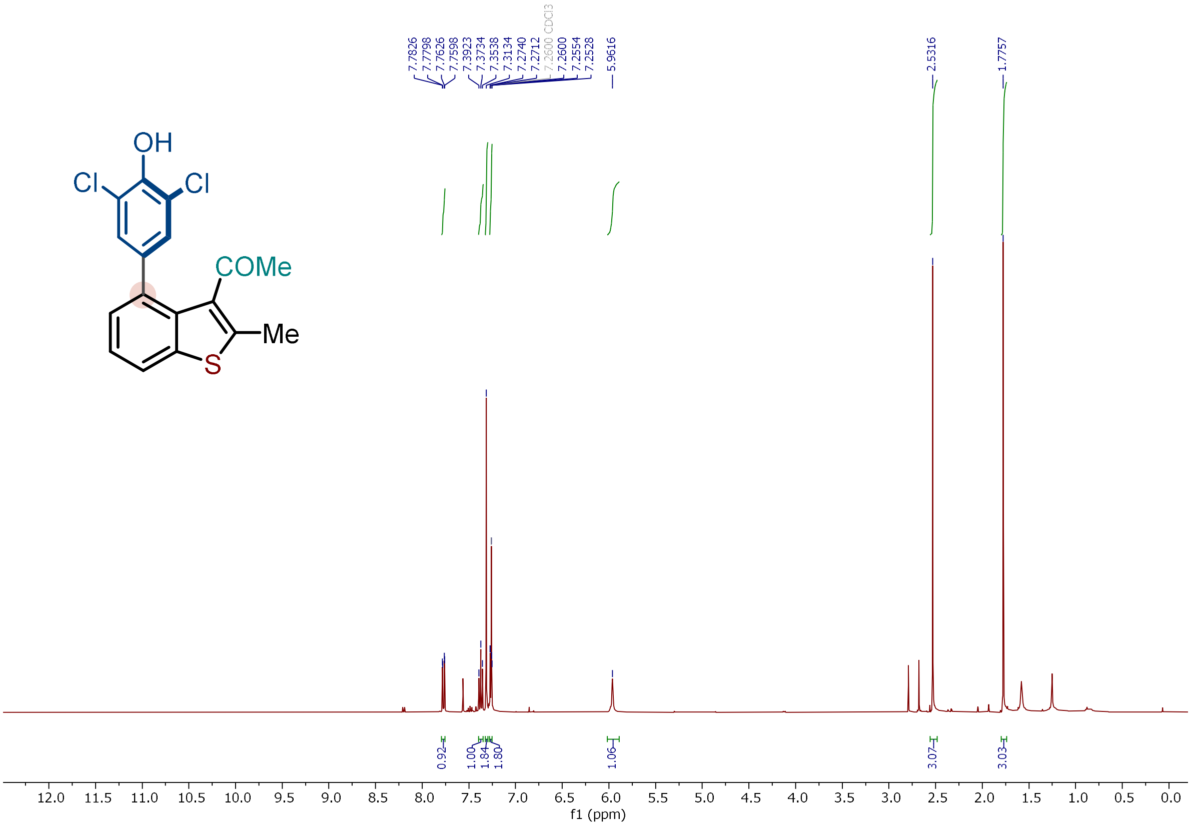

4h ^{13}C NMR (101 MHz, Acetone-d_6)

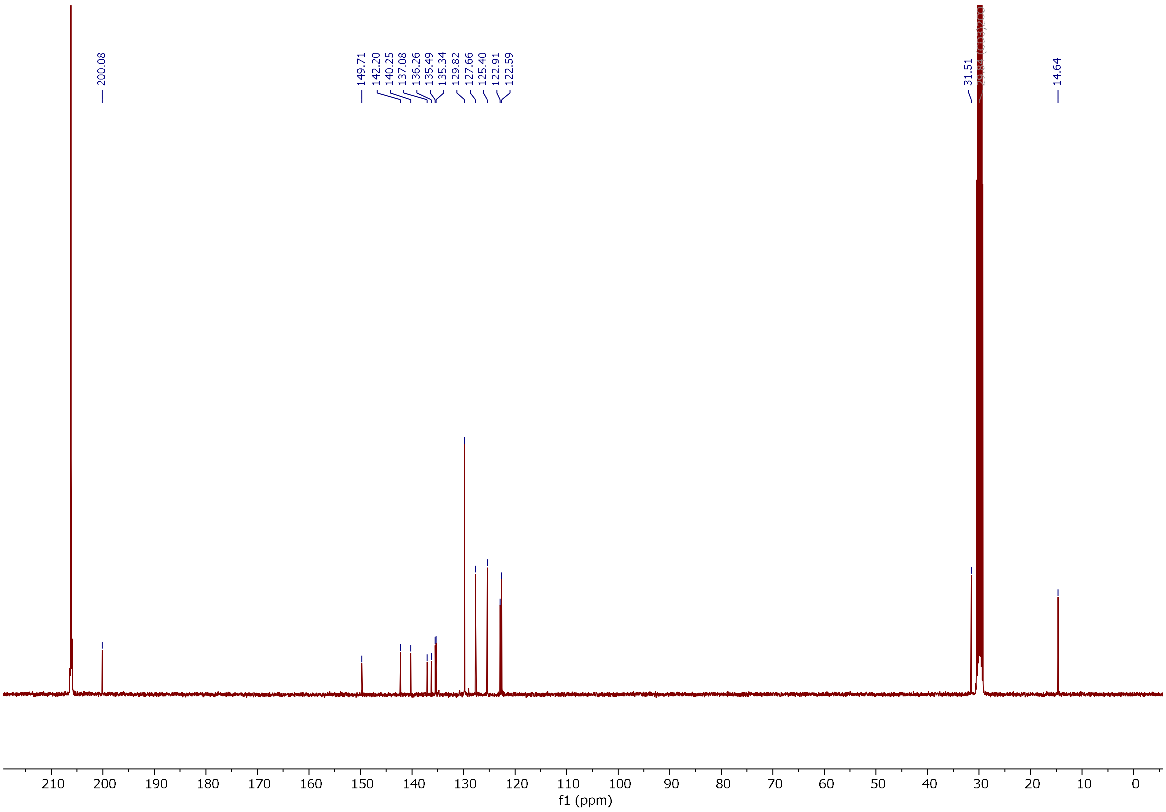

4i ^{1}H NMR (500 MHz, $CDCl_3$)

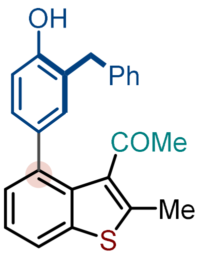

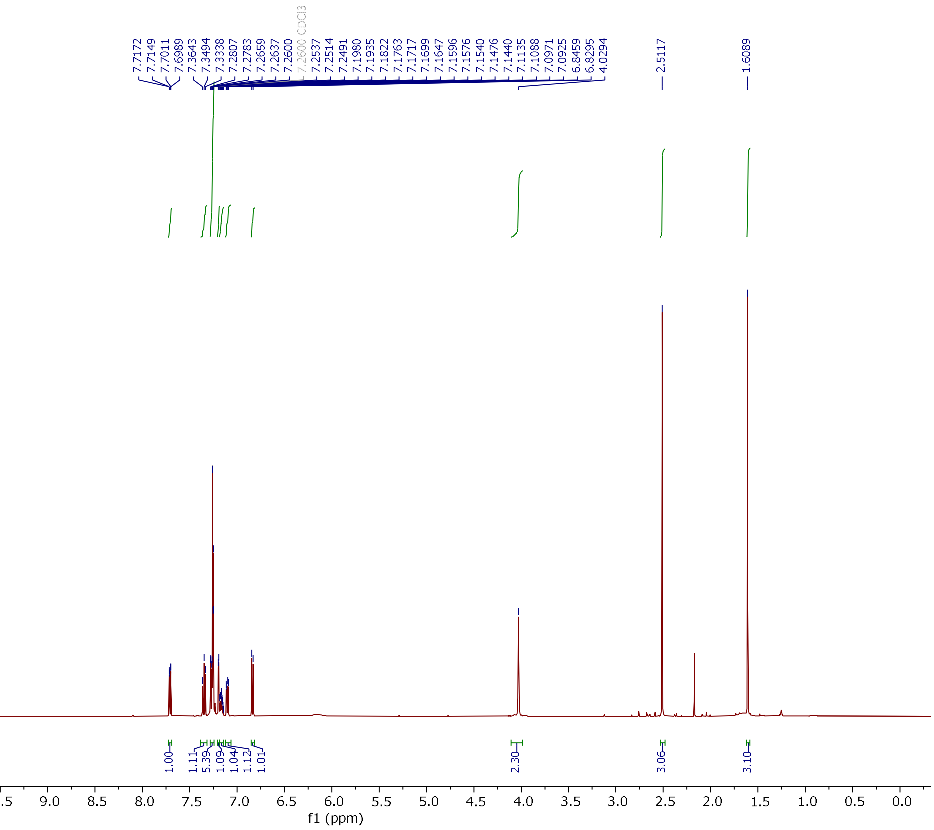

4i ^{13}C NMR (126 MHz, $CDCl_3$)

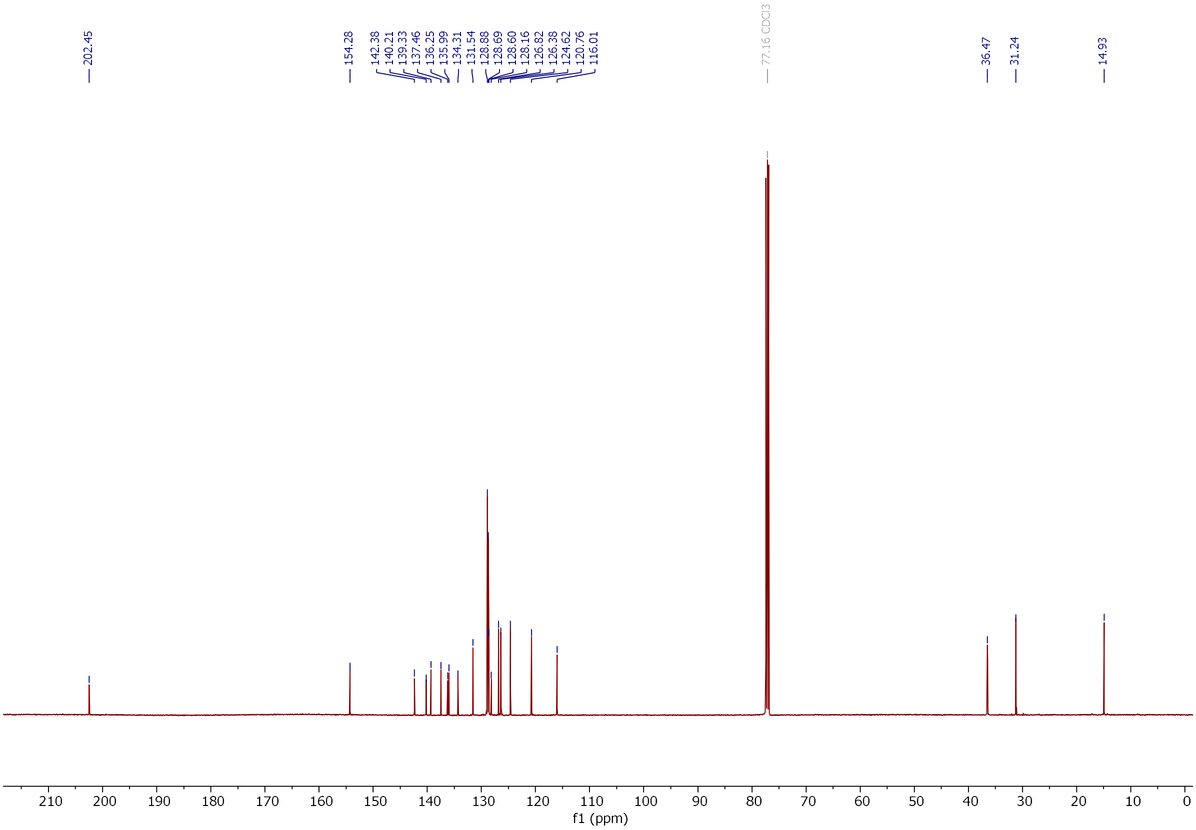

4j ^{1}H NMR (400 MHz, Acetone-d_6)

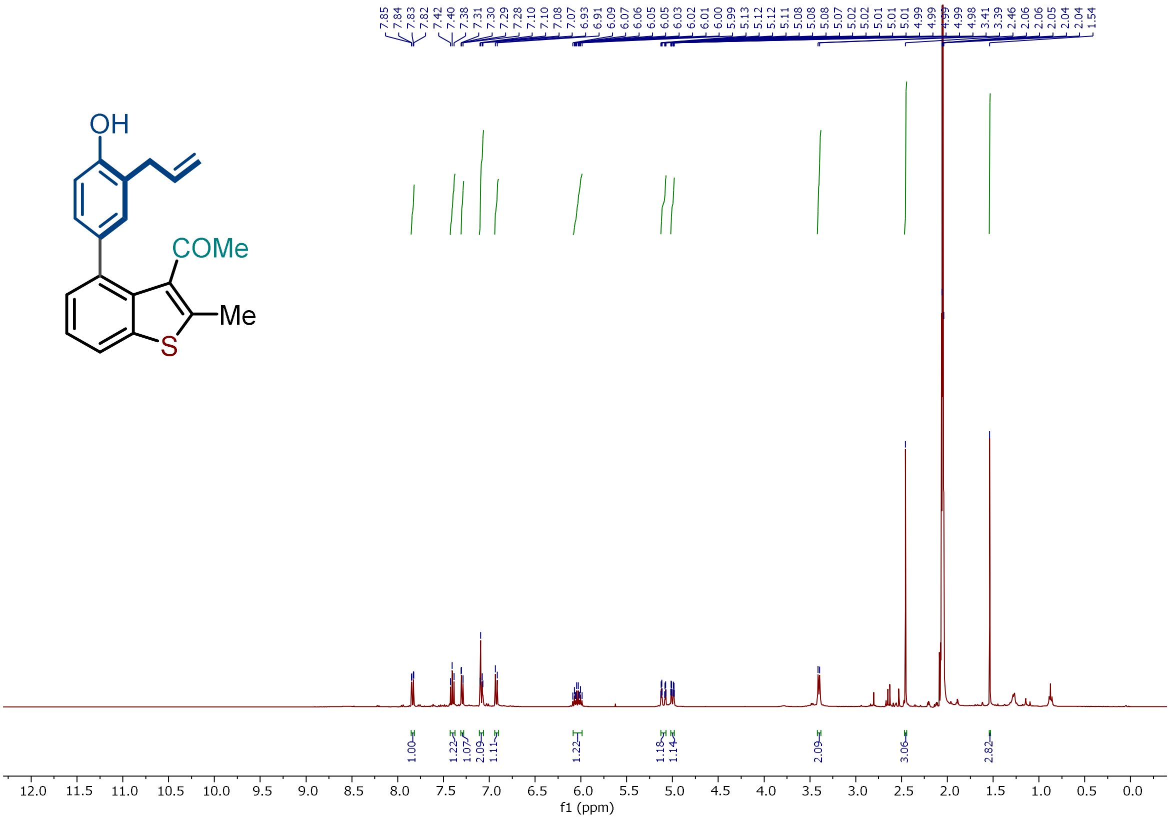

4j ^{13}C NMR (101 MHz, Acetone-d_6)

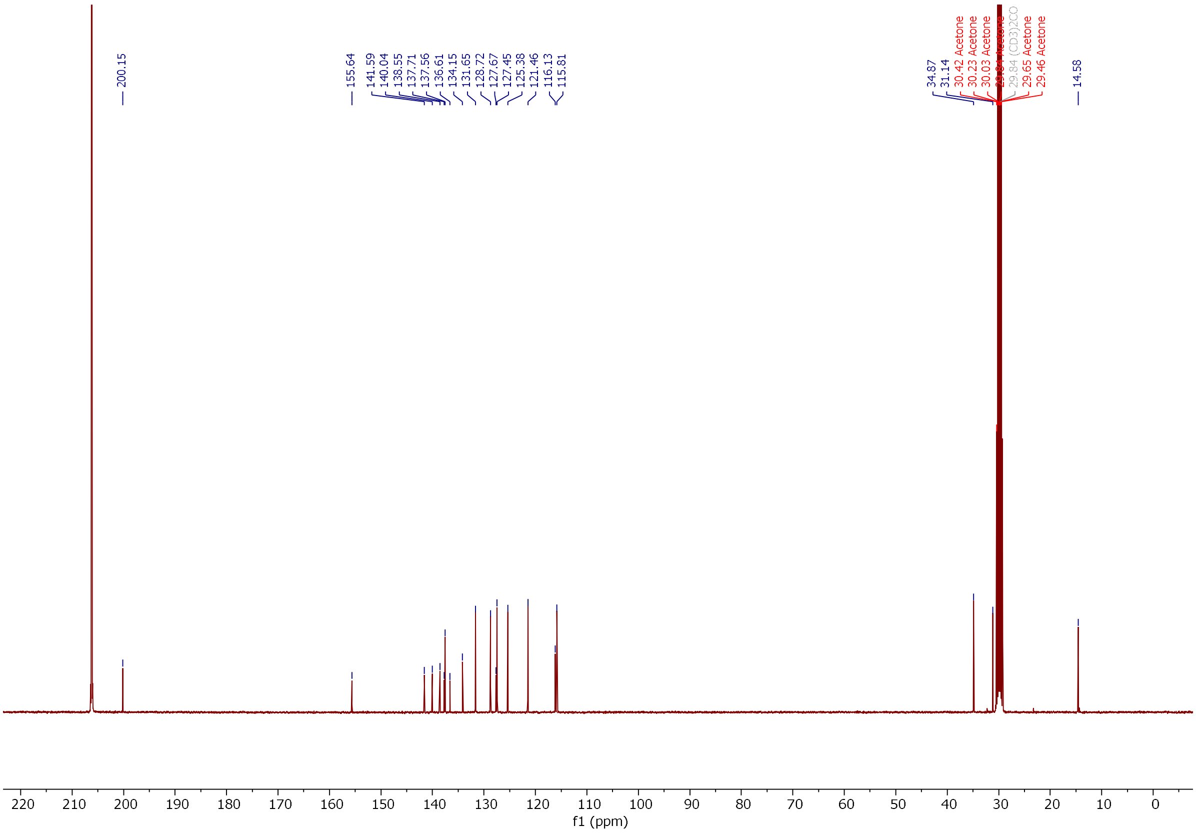

4k ^{1}H NMR (400 MHz, $CDCl_3$)

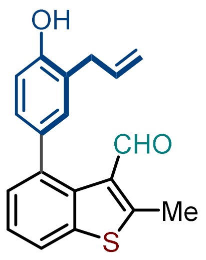

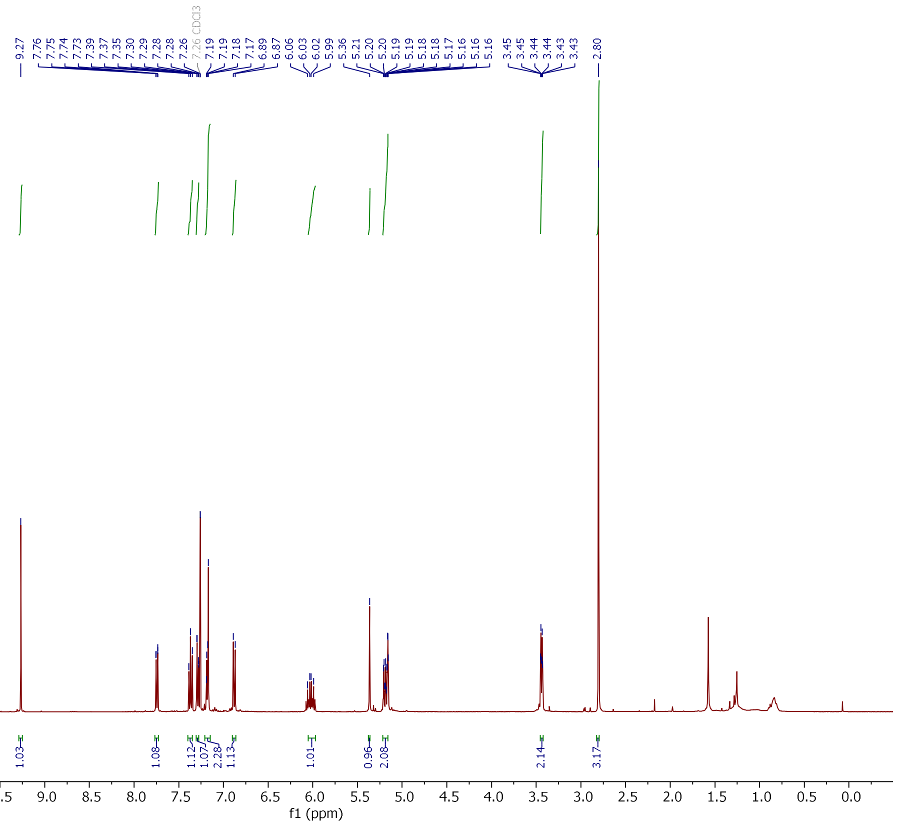

4k ^{13}C NMR (101 MHz, $CDCl_3$)

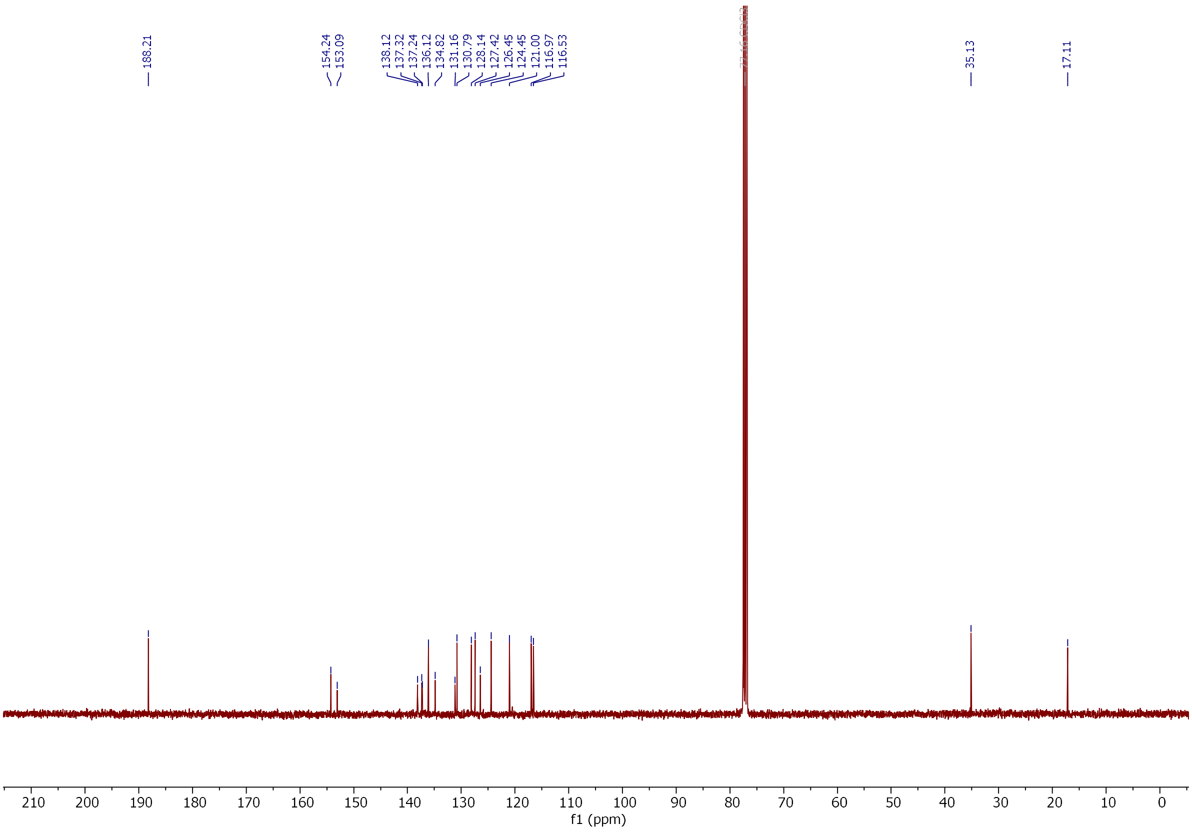

4l ^{1}H NMR (400 MHz, $CDCl_3$)

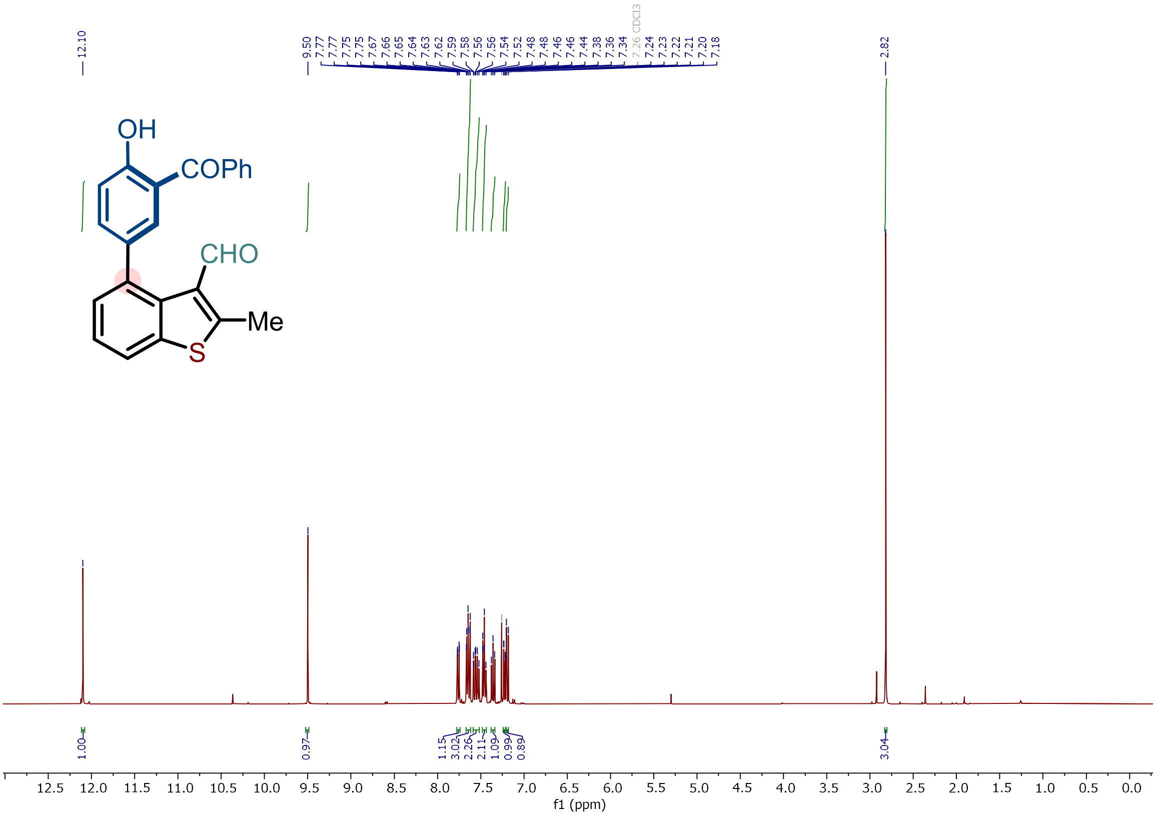

4l ^{13}C NMR (101 MHz, $CDCl_3$)

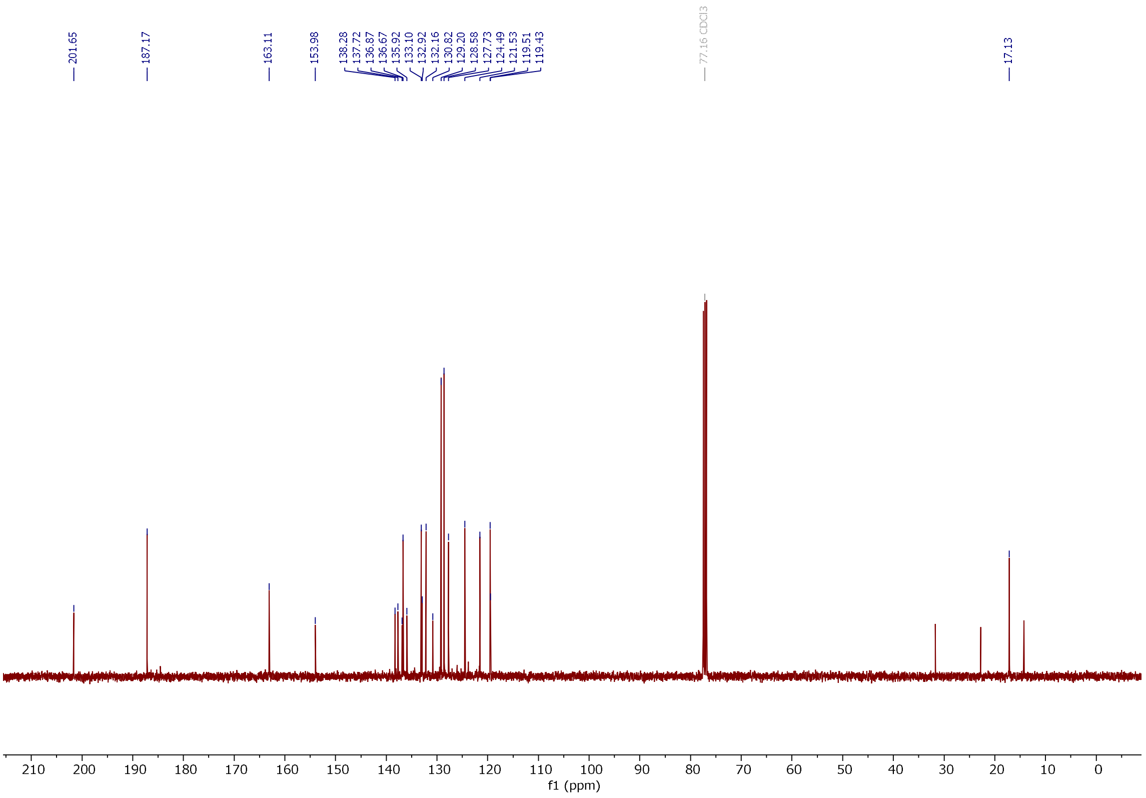

4m ^{1}H NMR (400 MHz, $CDCl_3$)

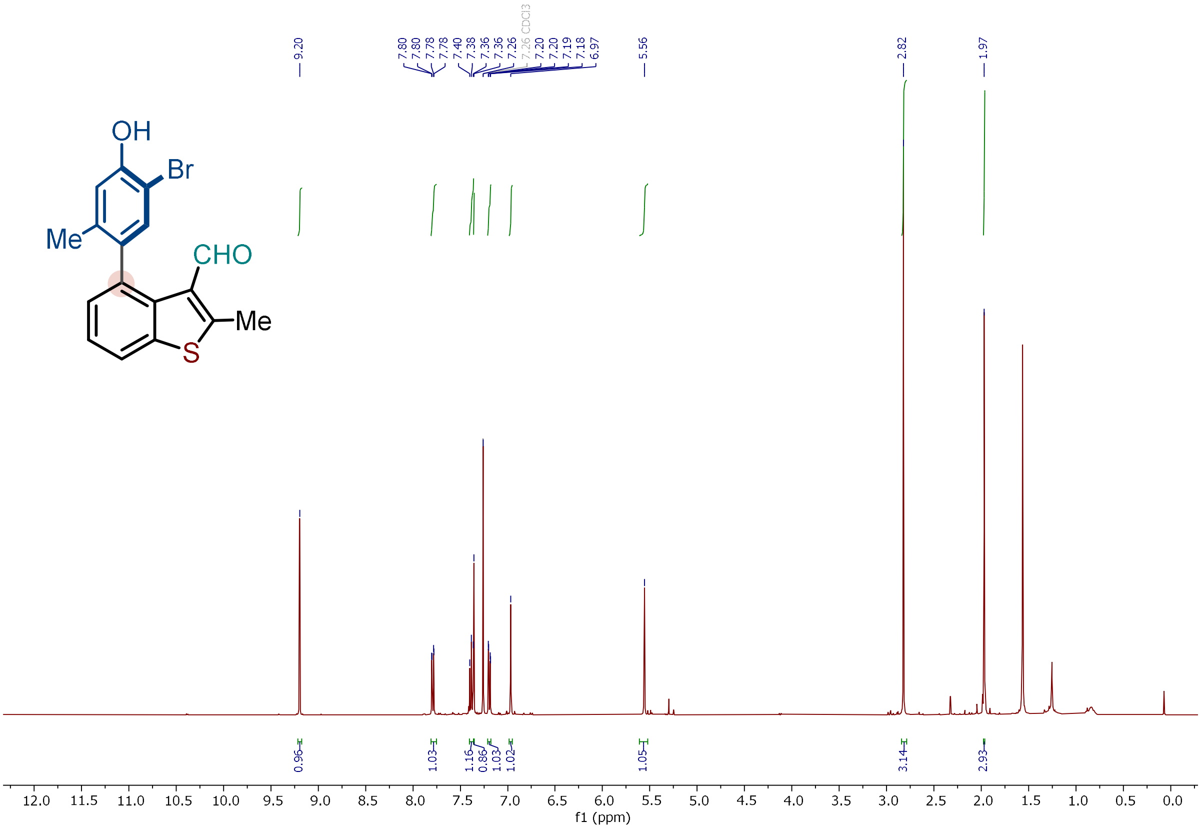

4m ^{13}C NMR (101 MHz, $CDCl_3$)

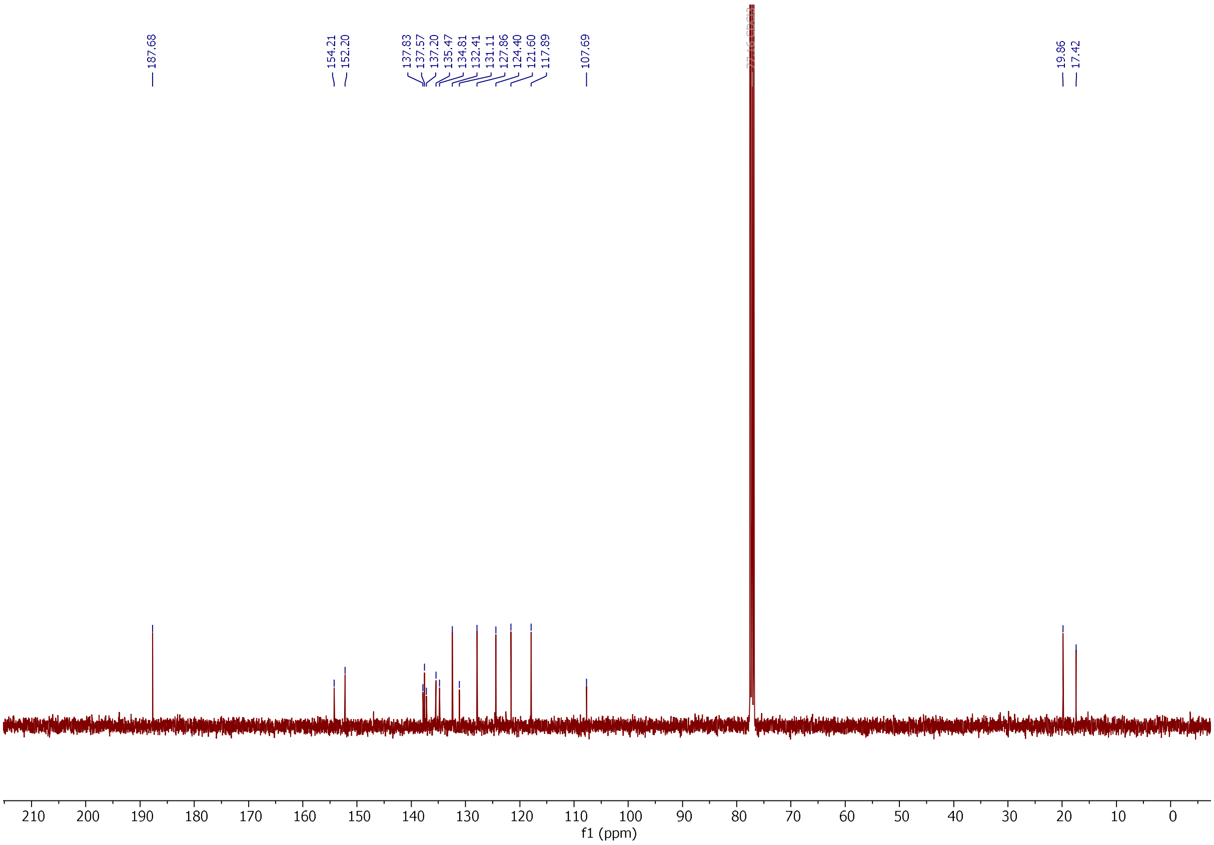

4n ^{1}H NMR (400 MHz, $CDCl_3$)

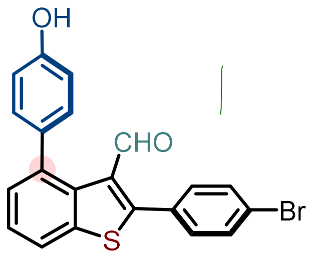

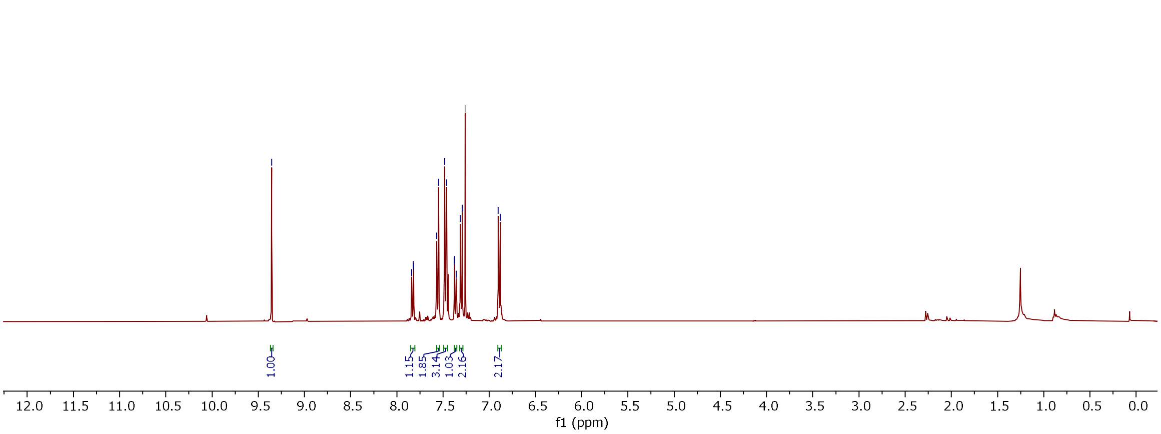

4n ^{13}C NMR (101 MHz, $CDCl_3$)

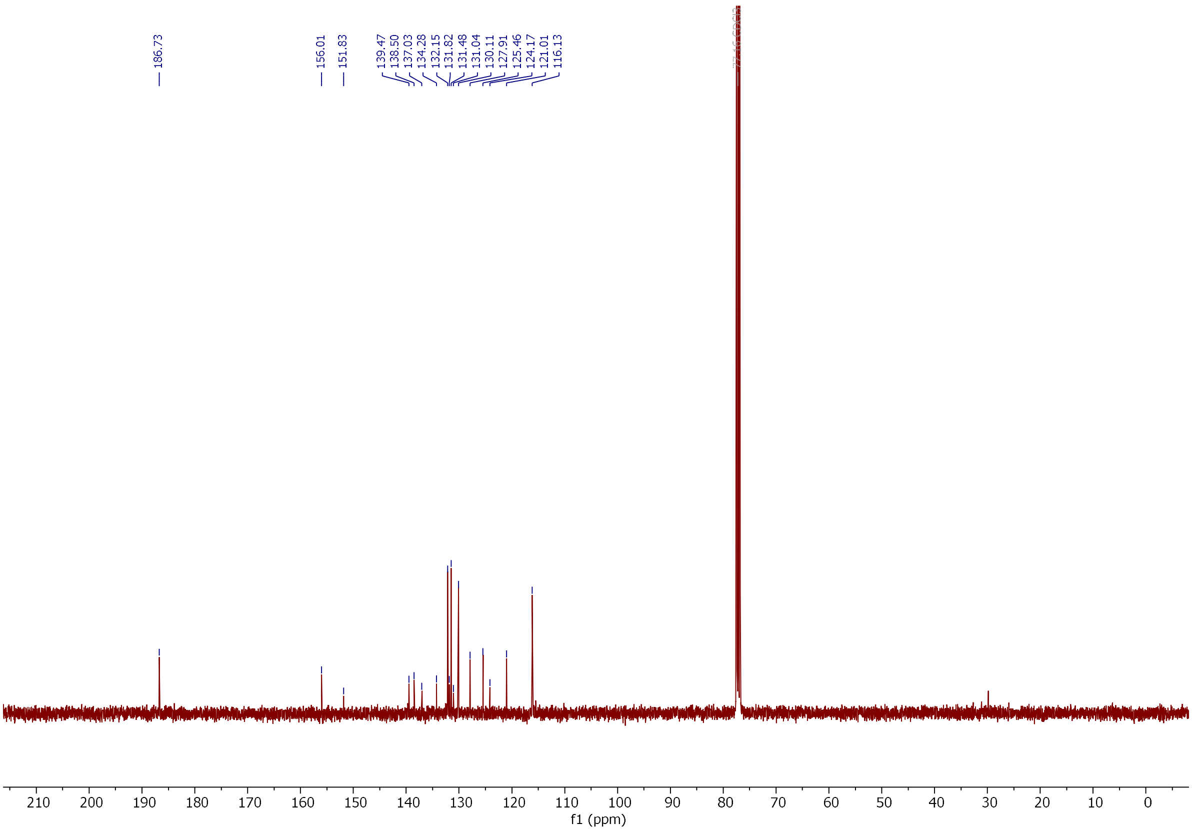

4o ^{1}H NMR (400 MHz, $CDCl_3$)

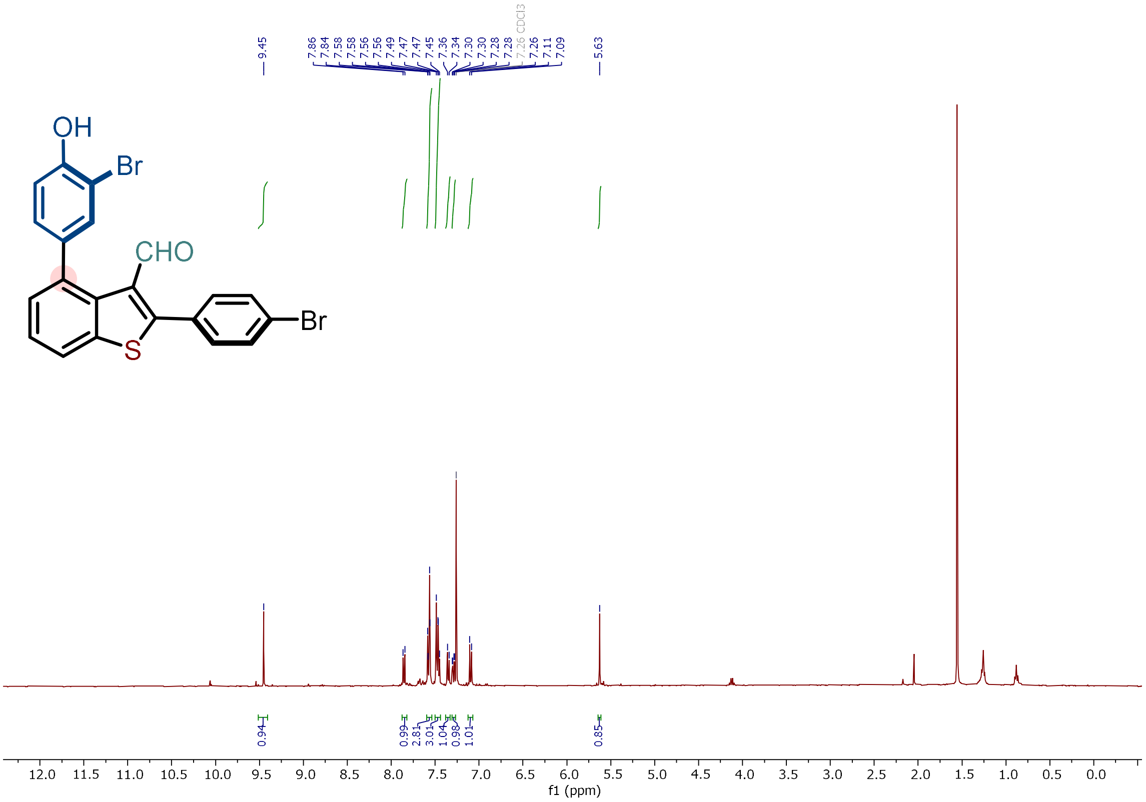

4o ^{13}C NMR (101 MHz, $CDCl_3$)

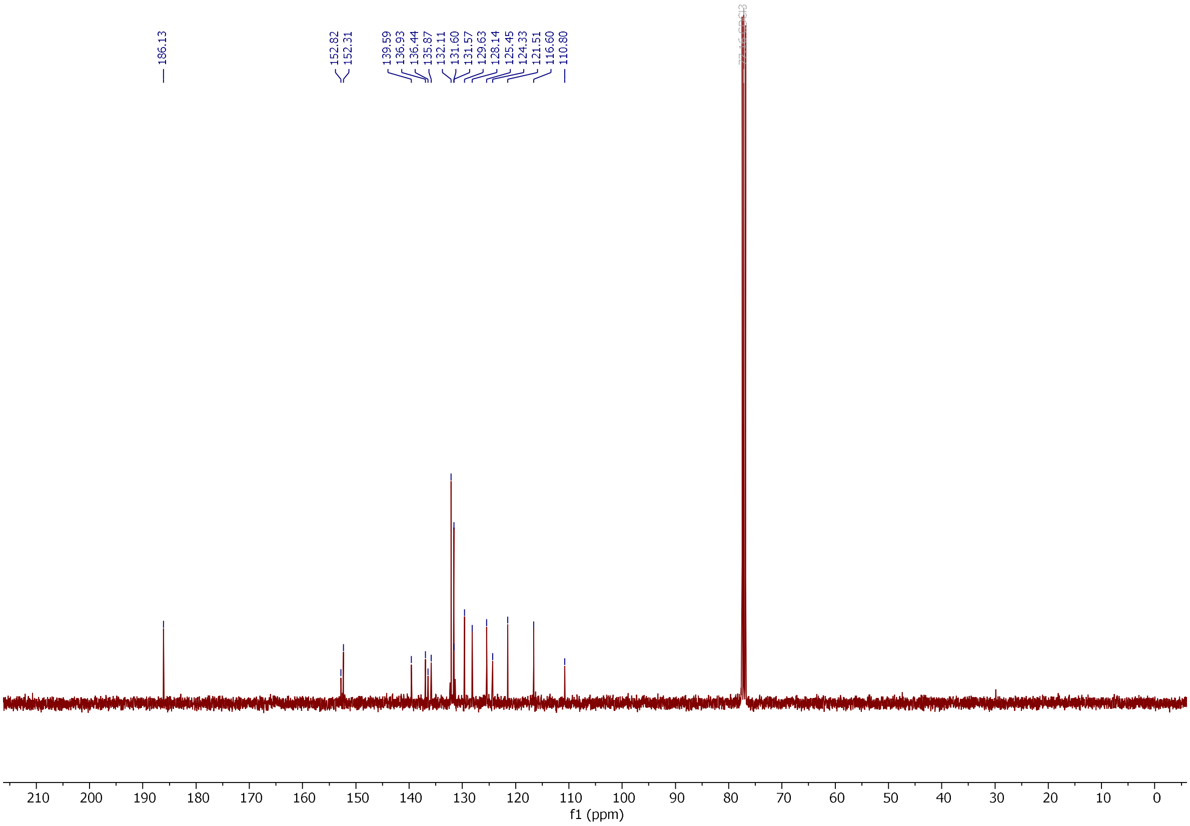

4p ^{1}H NMR (400 MHz, $CDCl_3$)

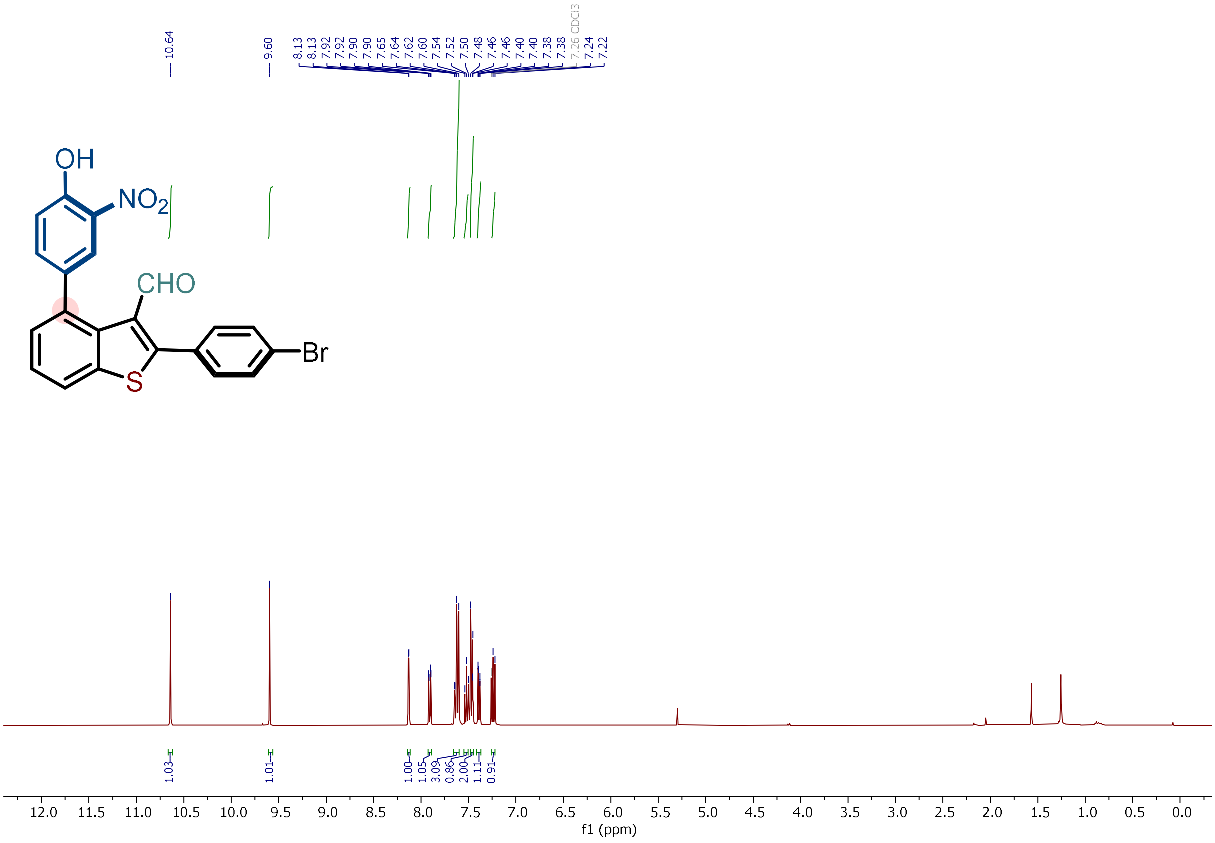

4p ^{13}C NMR (101 MHz, $CDCl_3$)

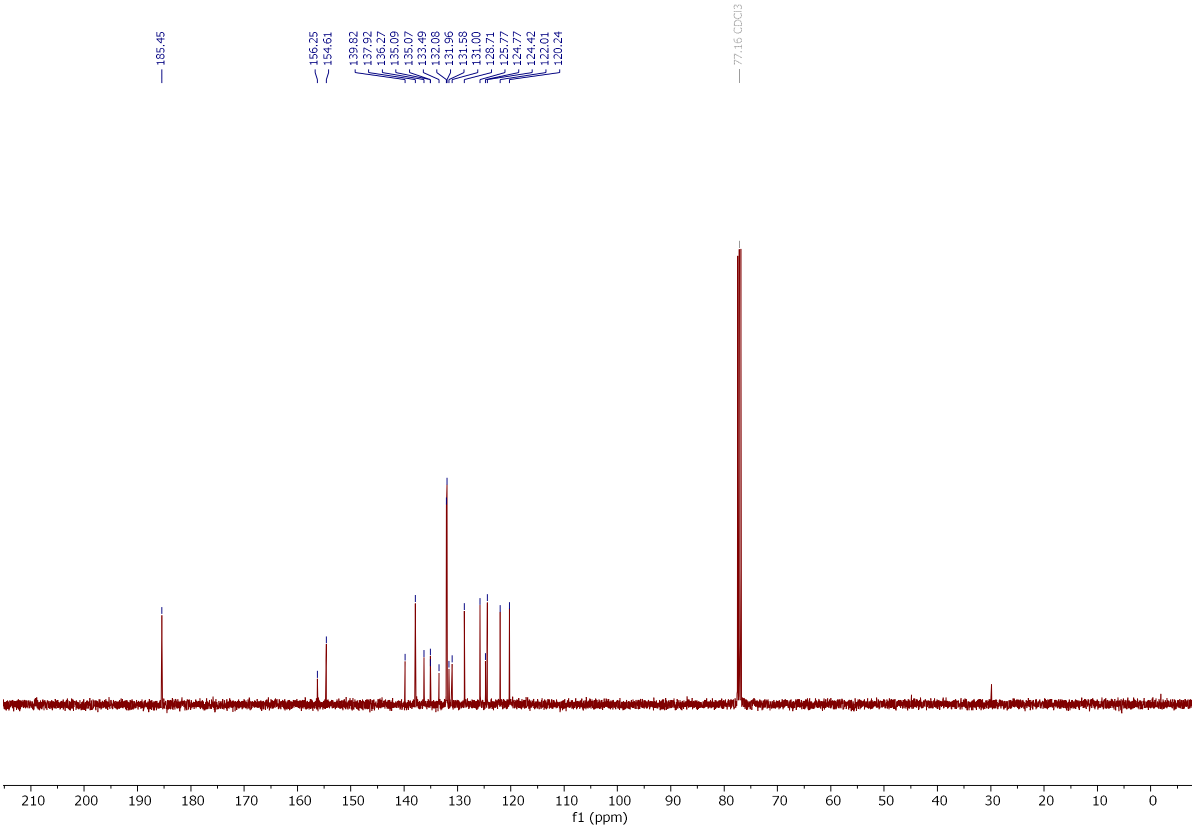

4q ^{1}H NMR (400 MHz, $CDCl_3$)

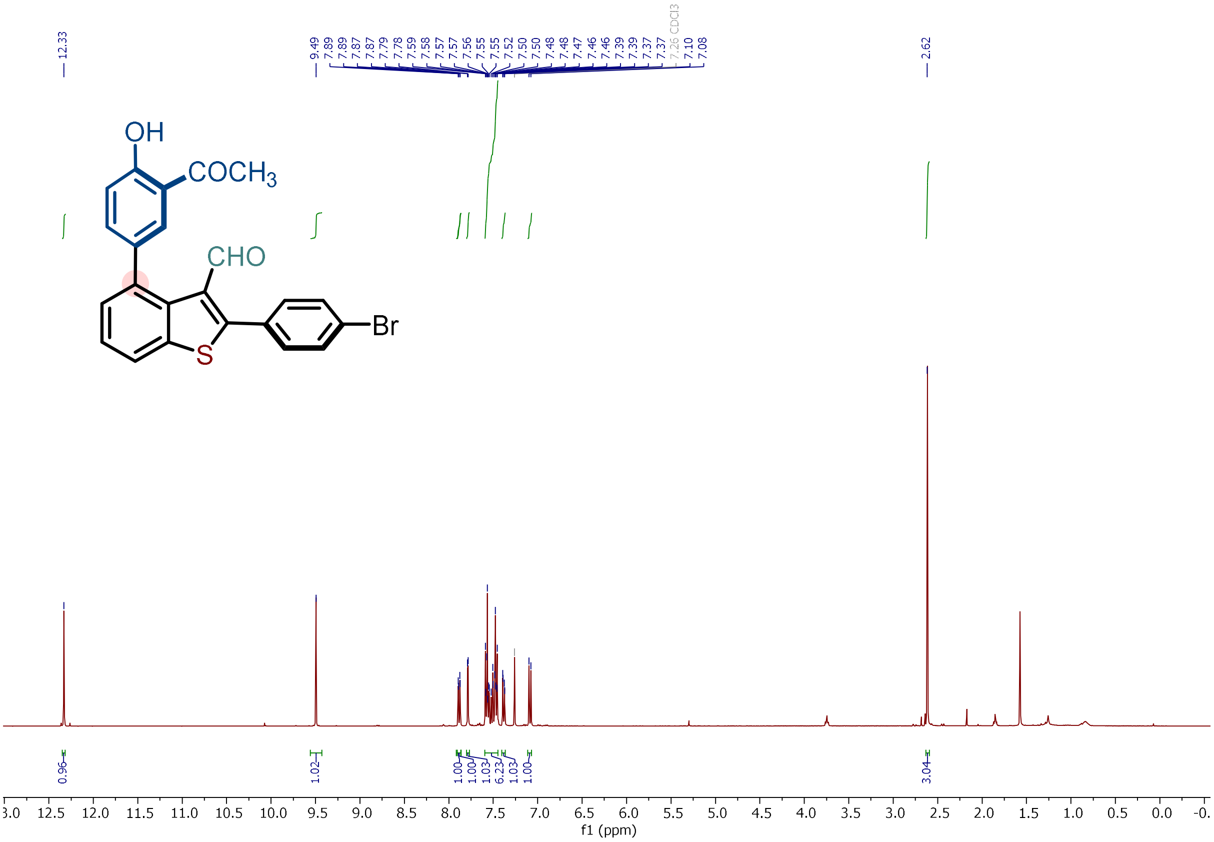

4q ^{13}C NMR (101 MHz, $CDCl_3$)

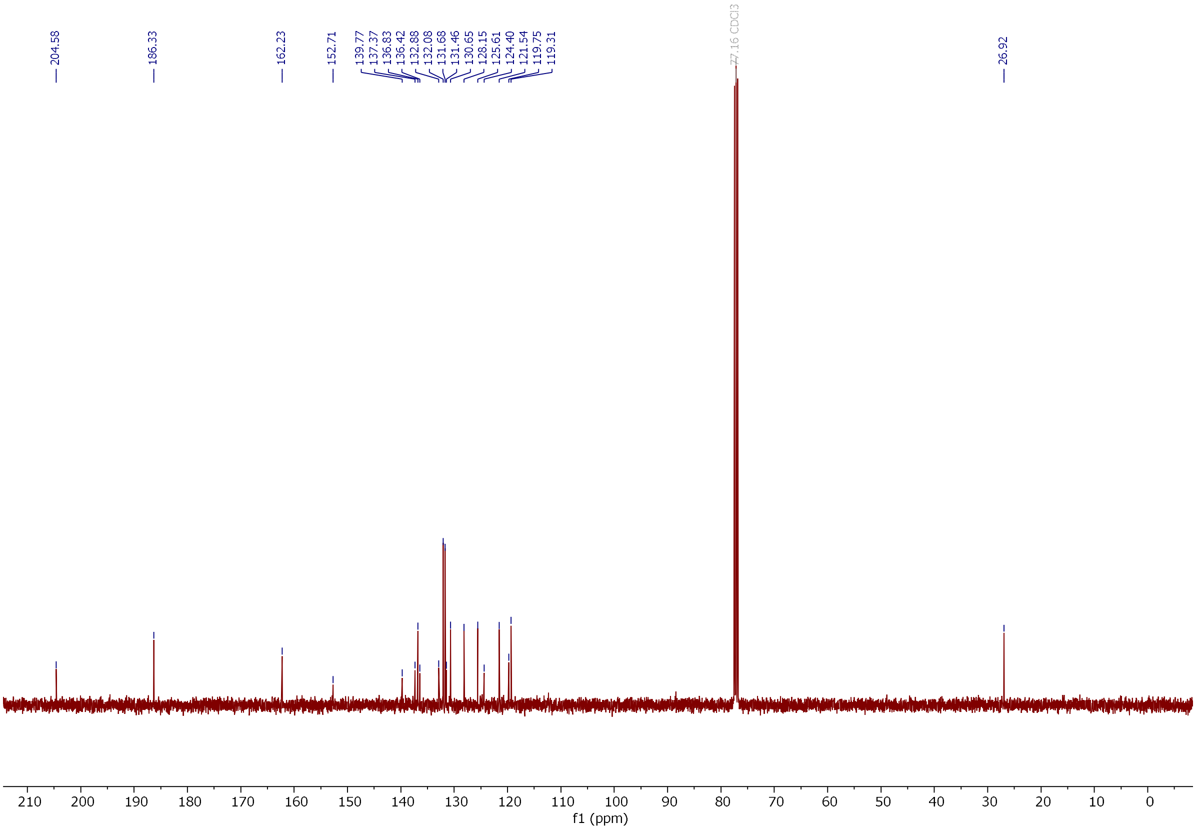

4r ^{1}H NMR (400 MHz, $CDCl_3$)

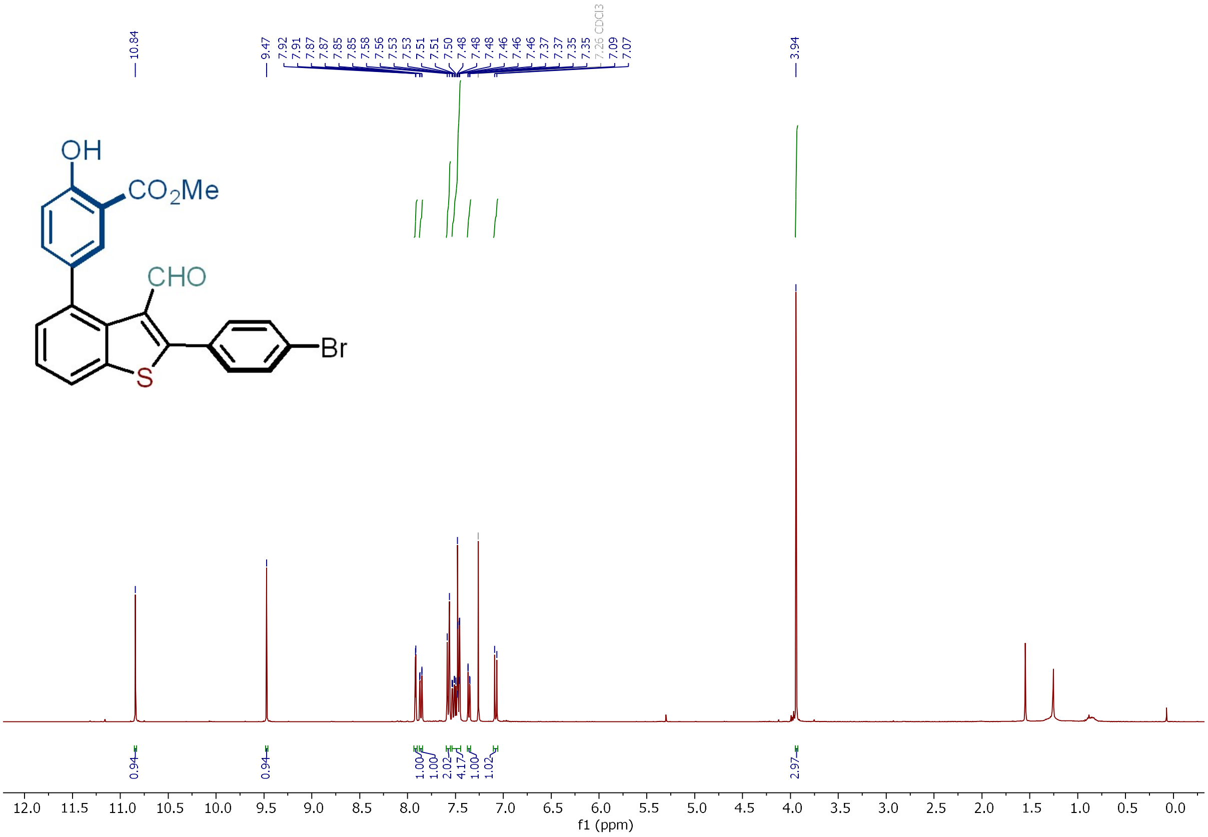

4r ^{13}C NMR (101 MHz, $CDCl_3$)

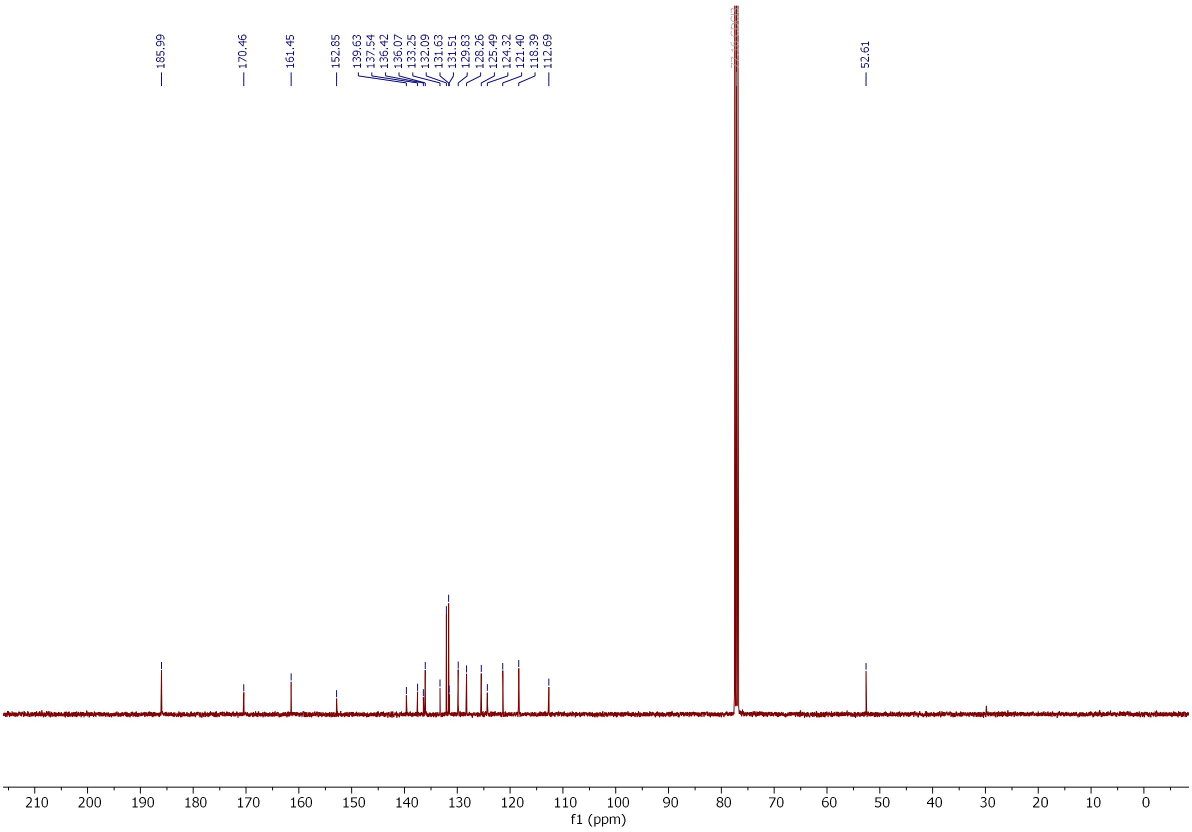

4s ^{1}H NMR (400 MHz, DMSO-d$_6$)

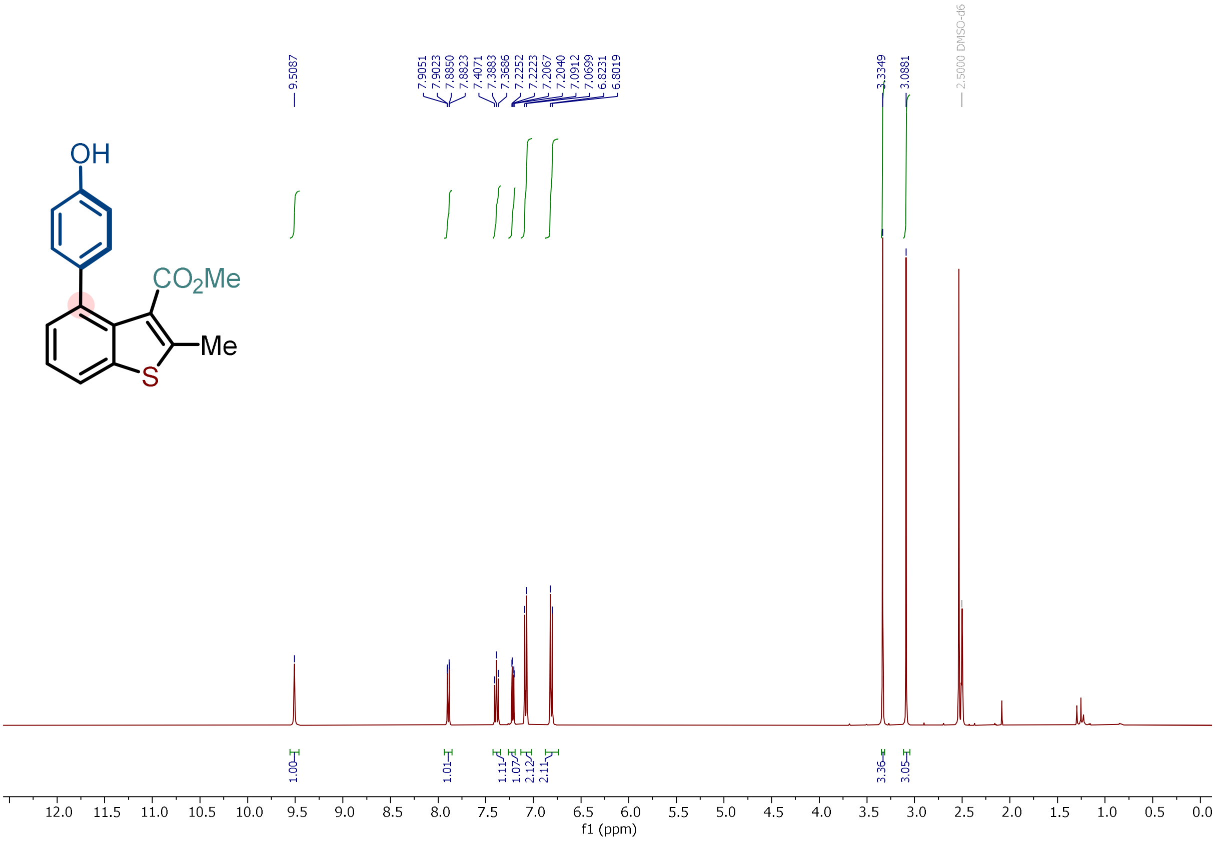

4s ^{13}C NMR (101 MHz, DMSO-d$_6$)

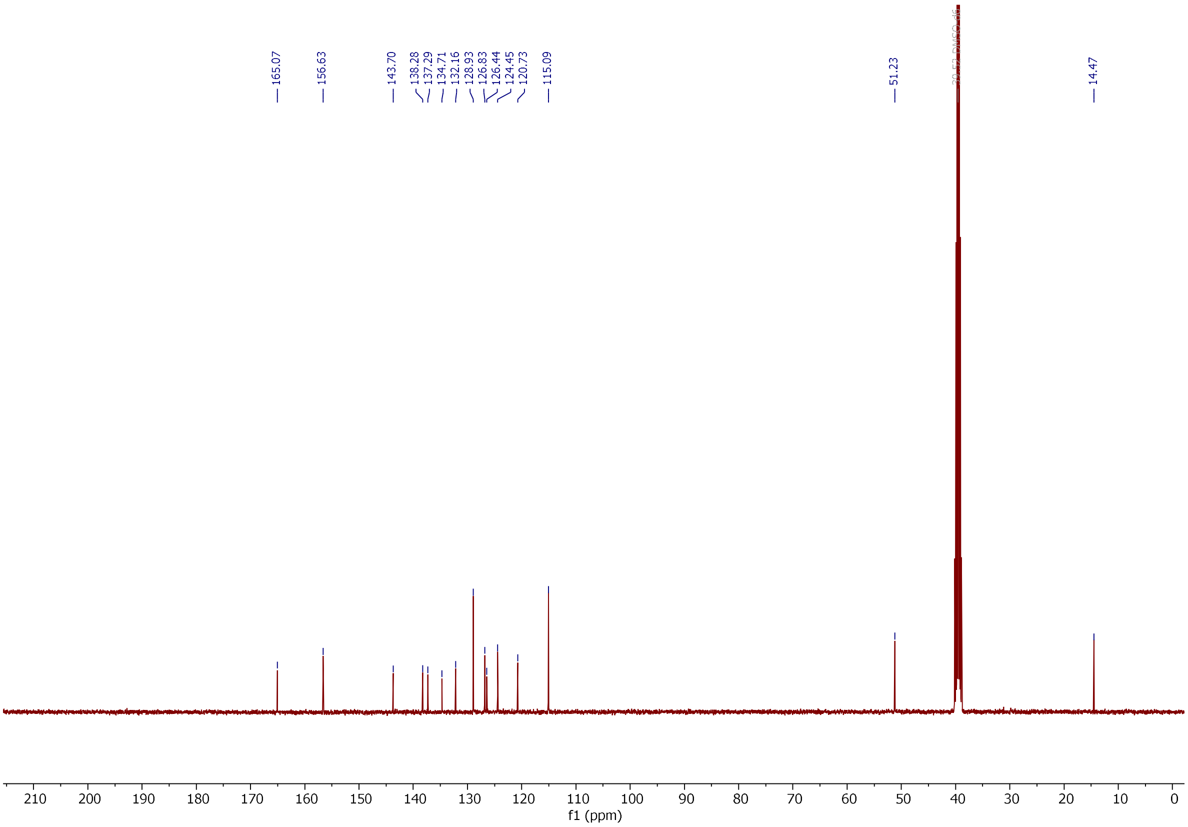

4t ^{1}H NMR (400 MHz, $CDCl_3$)

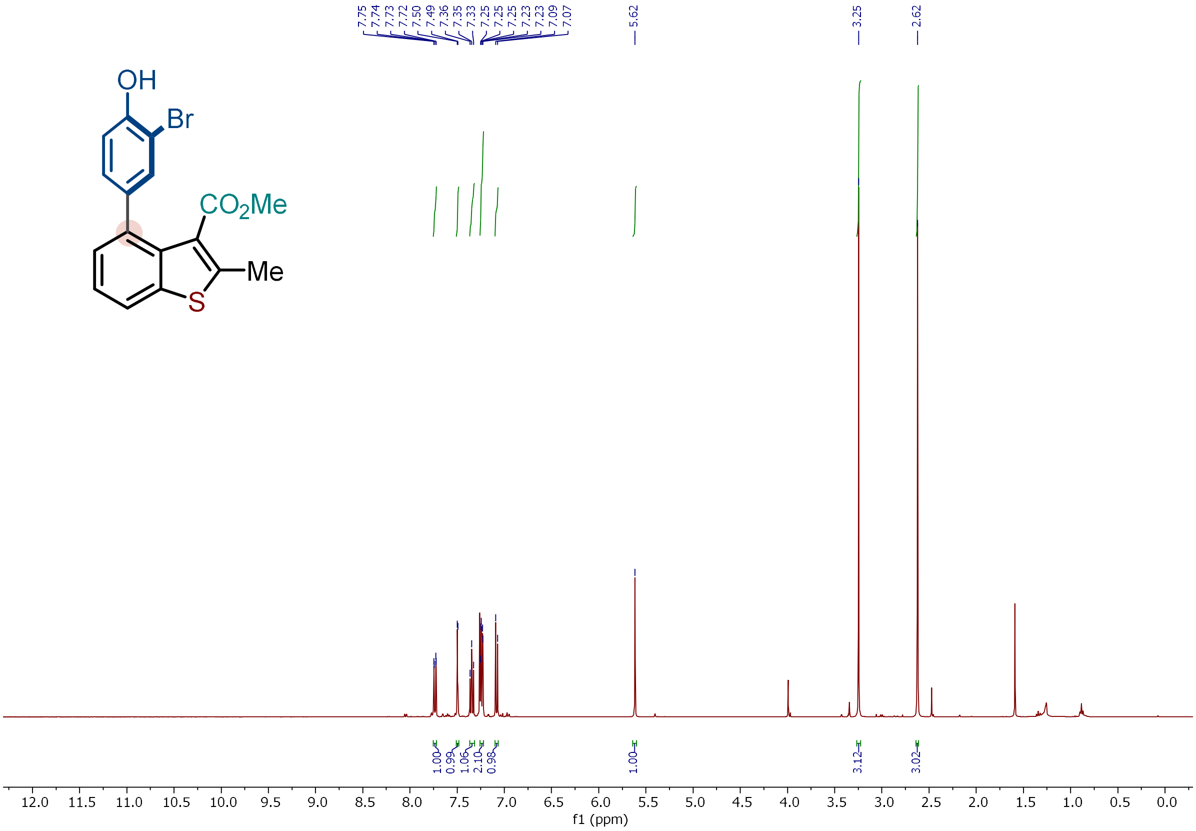

4t ^{13}C NMR (101 MHz, $CDCl_3$)

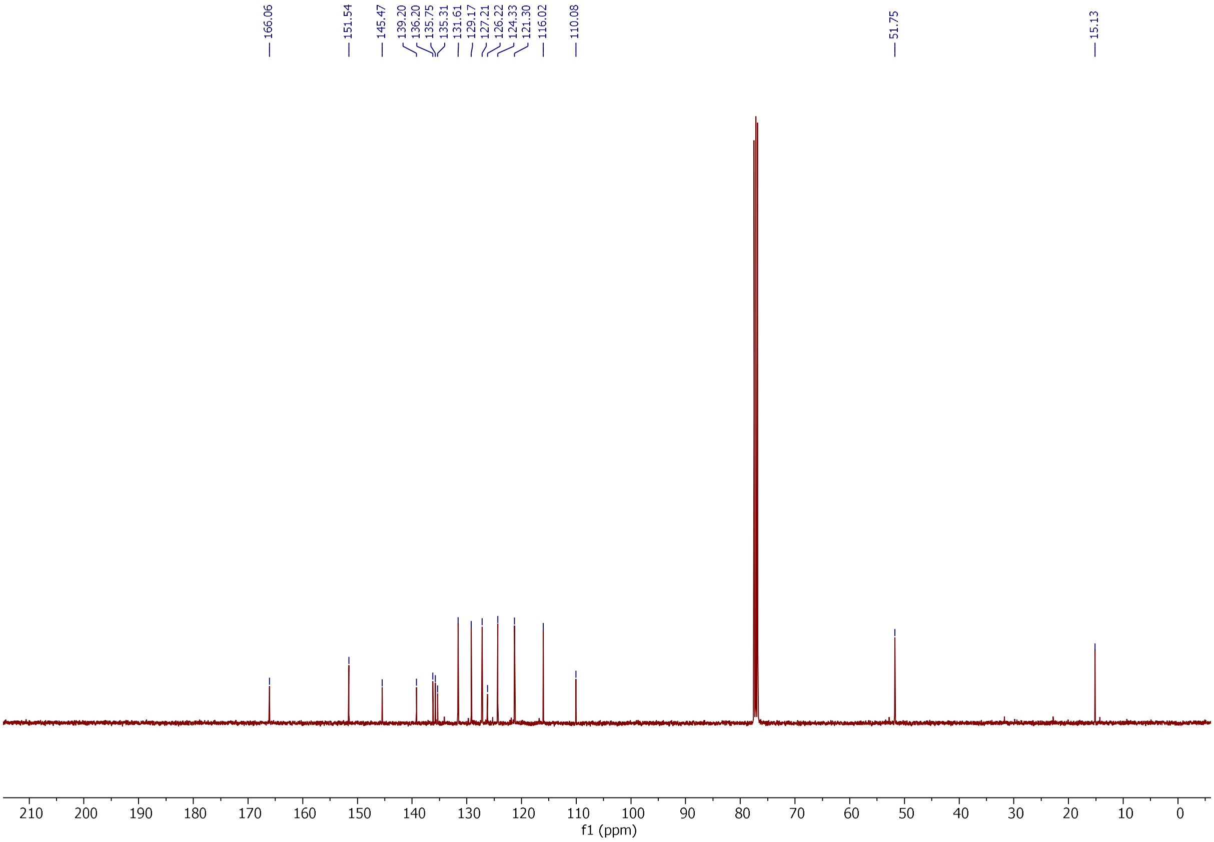

4u ^{1}H NMR (500 MHz, $CDCl_3$)

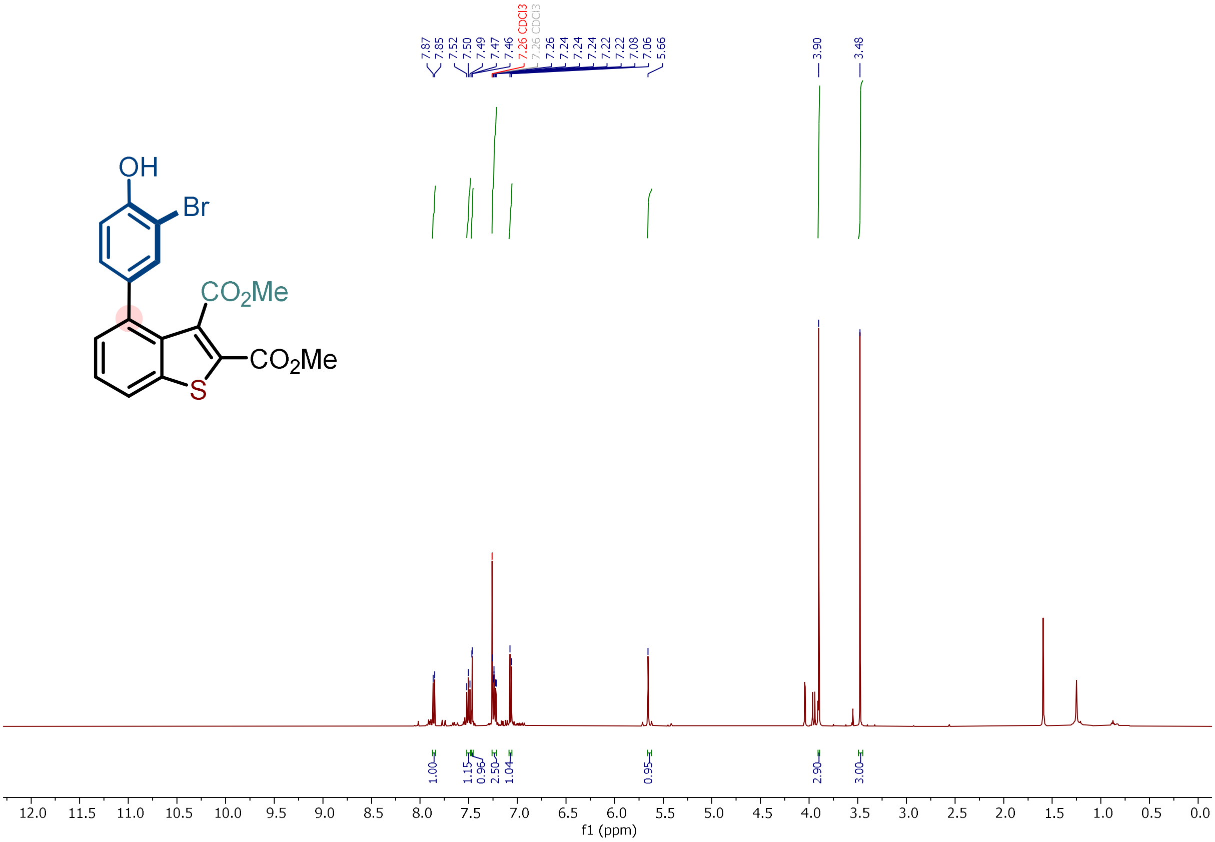

4u ^{13}C NMR (126 MHz, $CDCl_3$)

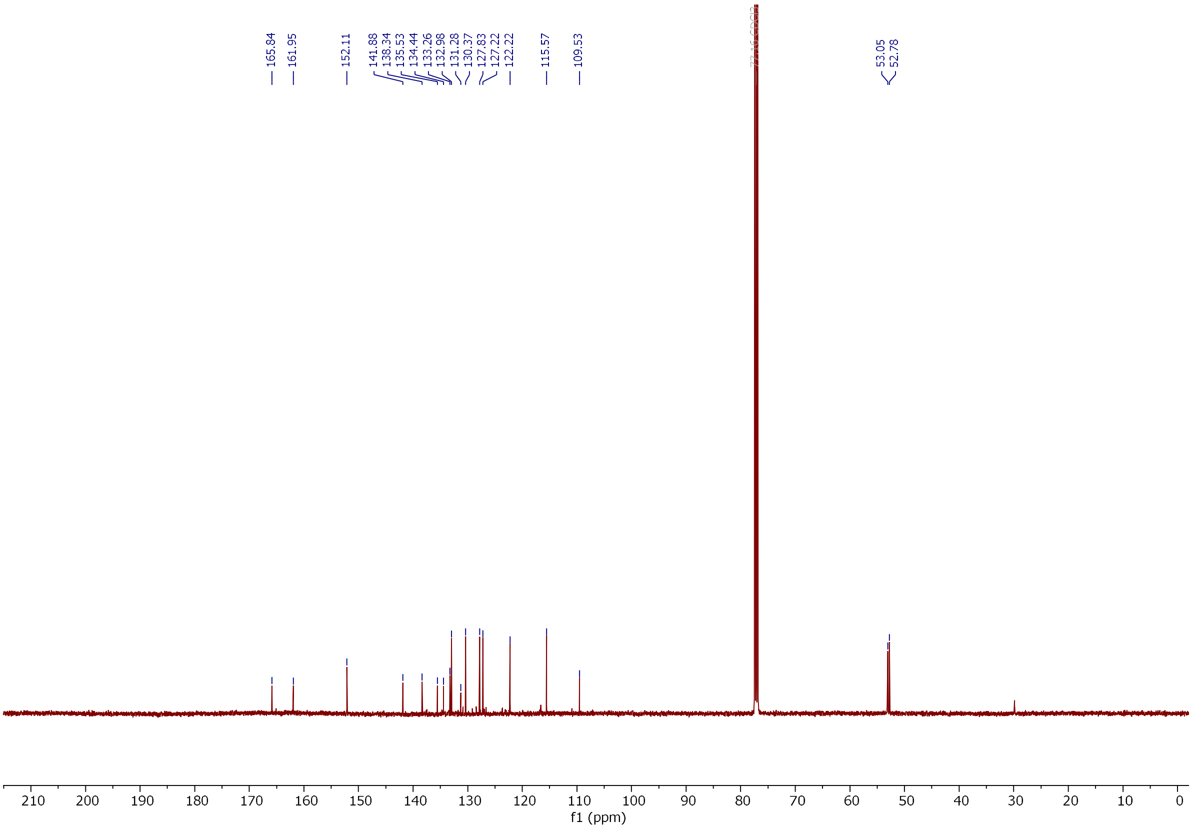

4v ^{1}H NMR (400 MHz, $CDCl_3$)

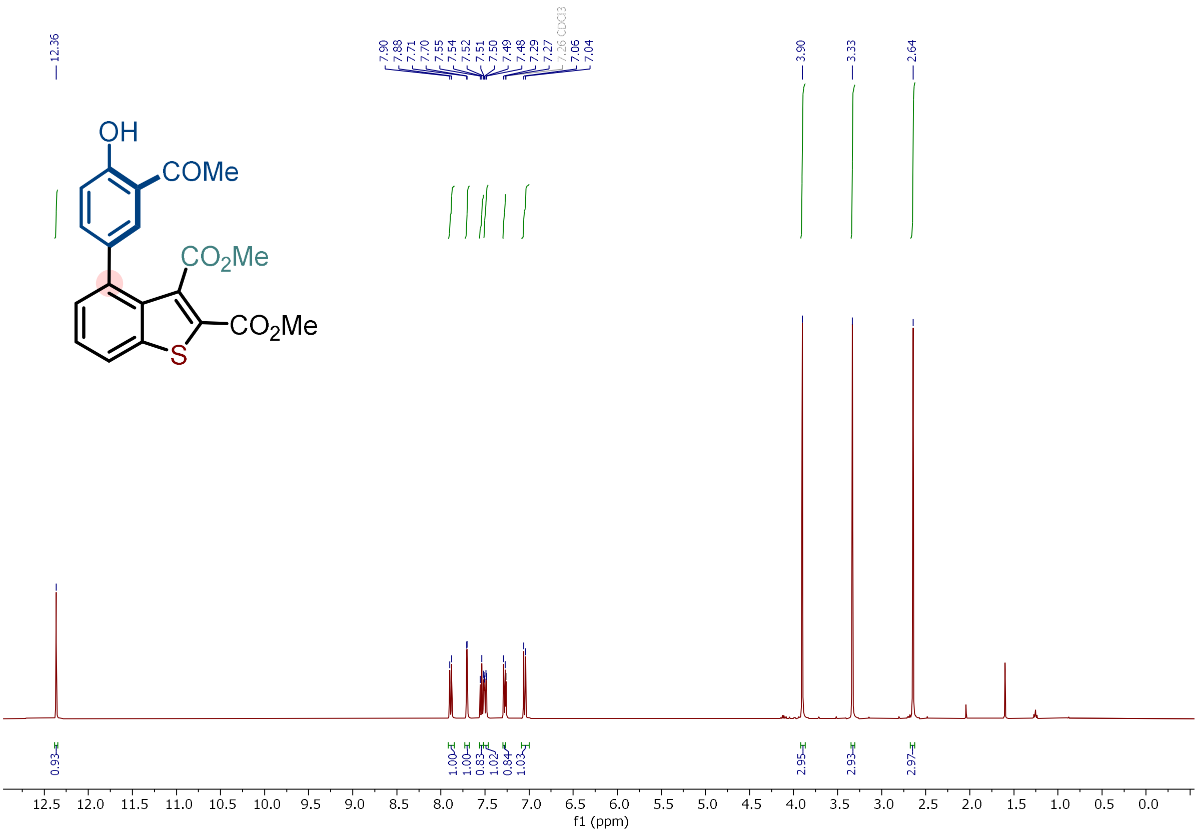

4v ^{13}C NMR (101 MHz, $CDCl_3$)

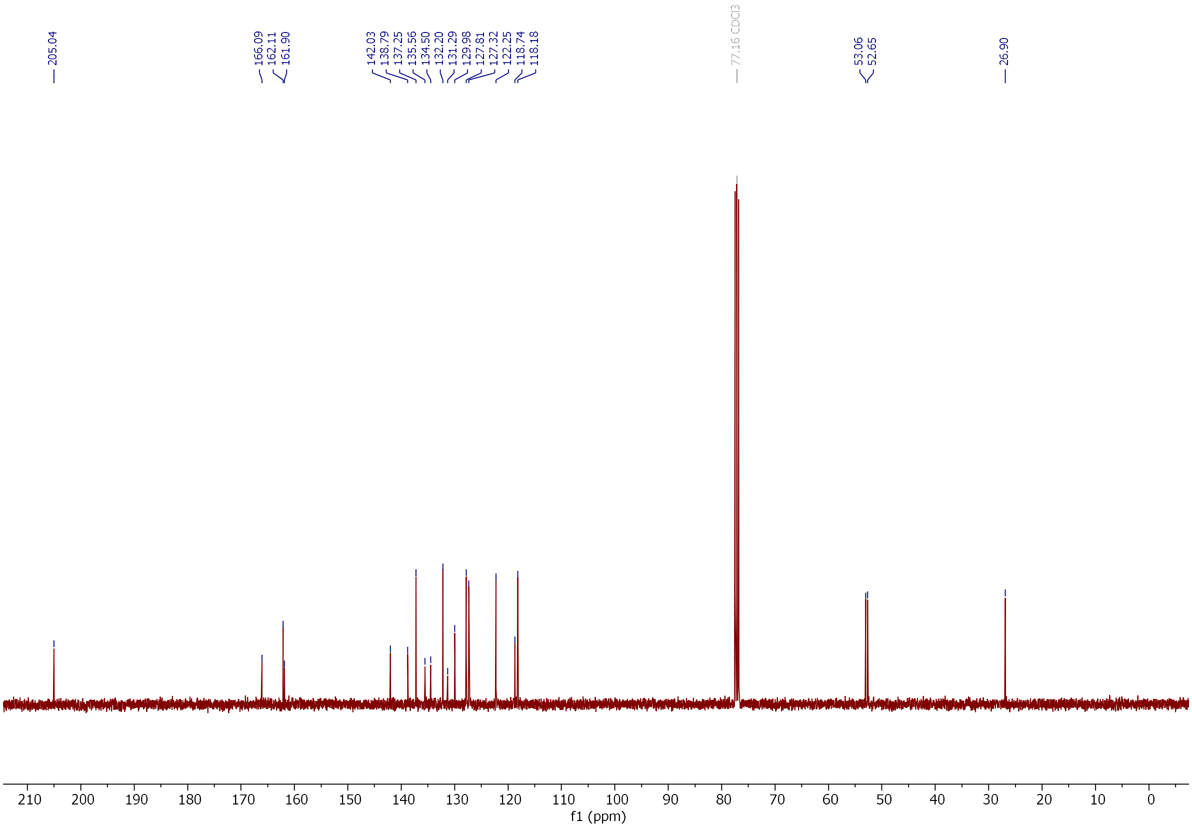

4w ^{1}H NMR (400 MHz, DMSO-d$_6$)

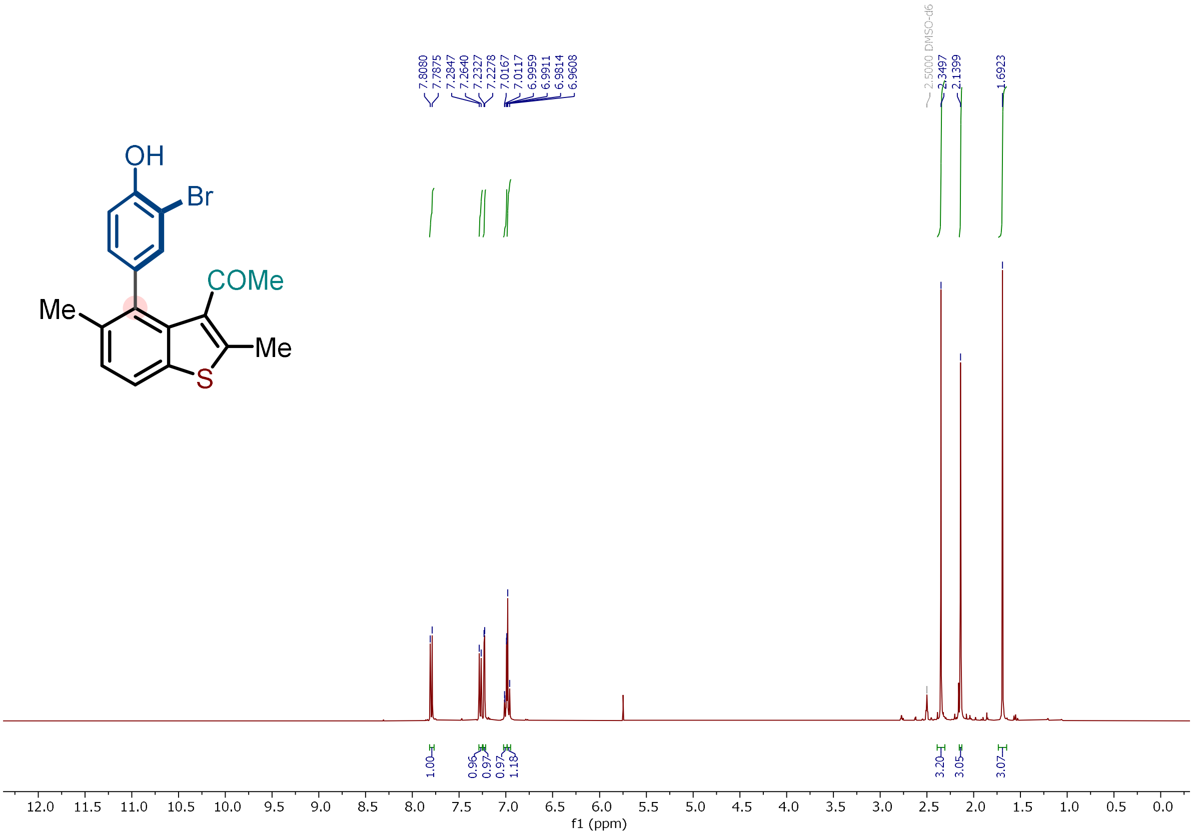

4w ^{13}C NMR (101 MHz, DMSO-d$_6$)

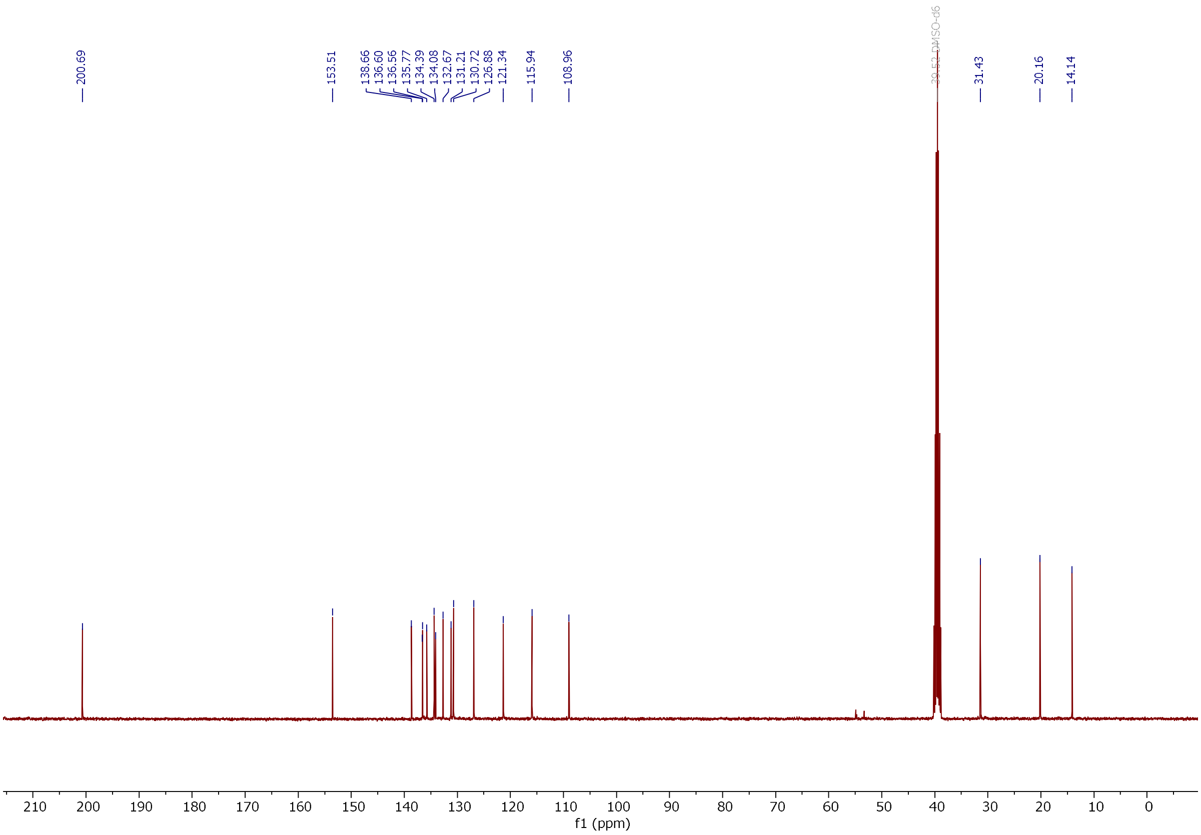

4x ^{1}H NMR (400 MHz, Acetone-d$_6$)

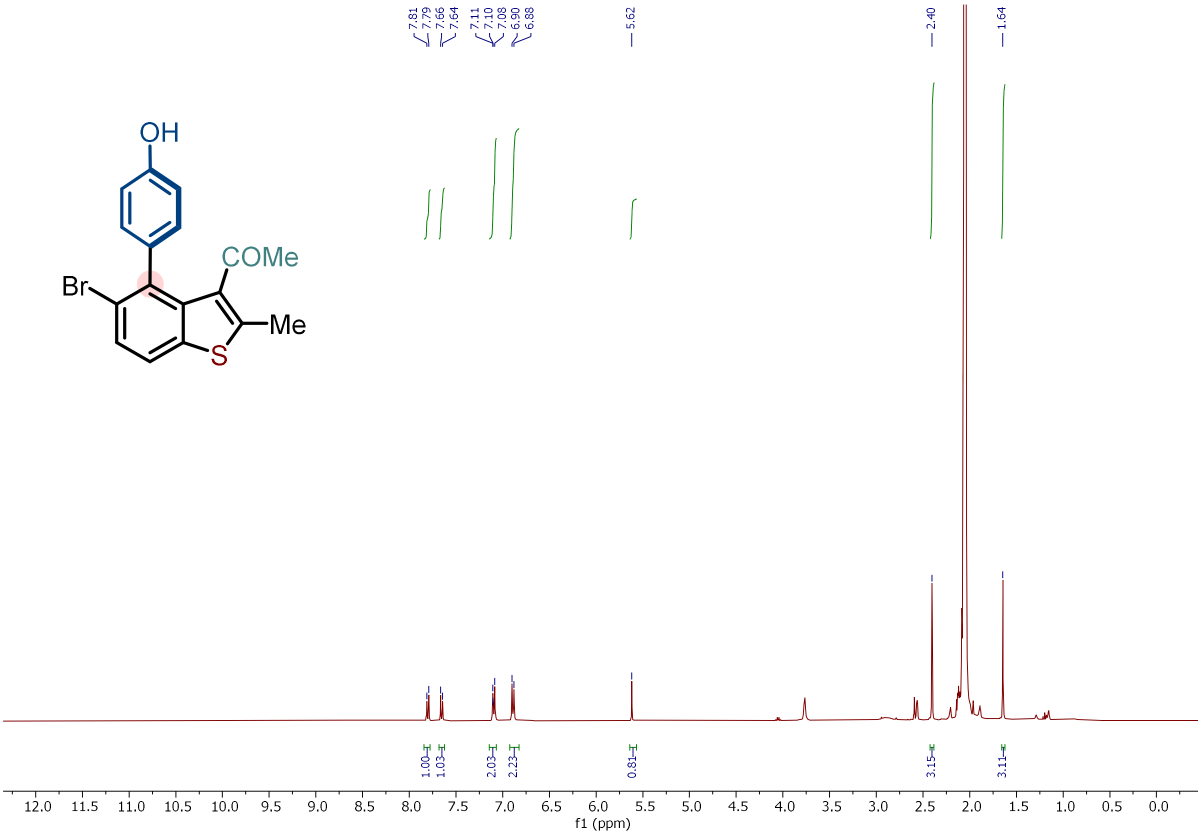

4x ^{13}C NMR (101MHz, Acetone-d$_6$)

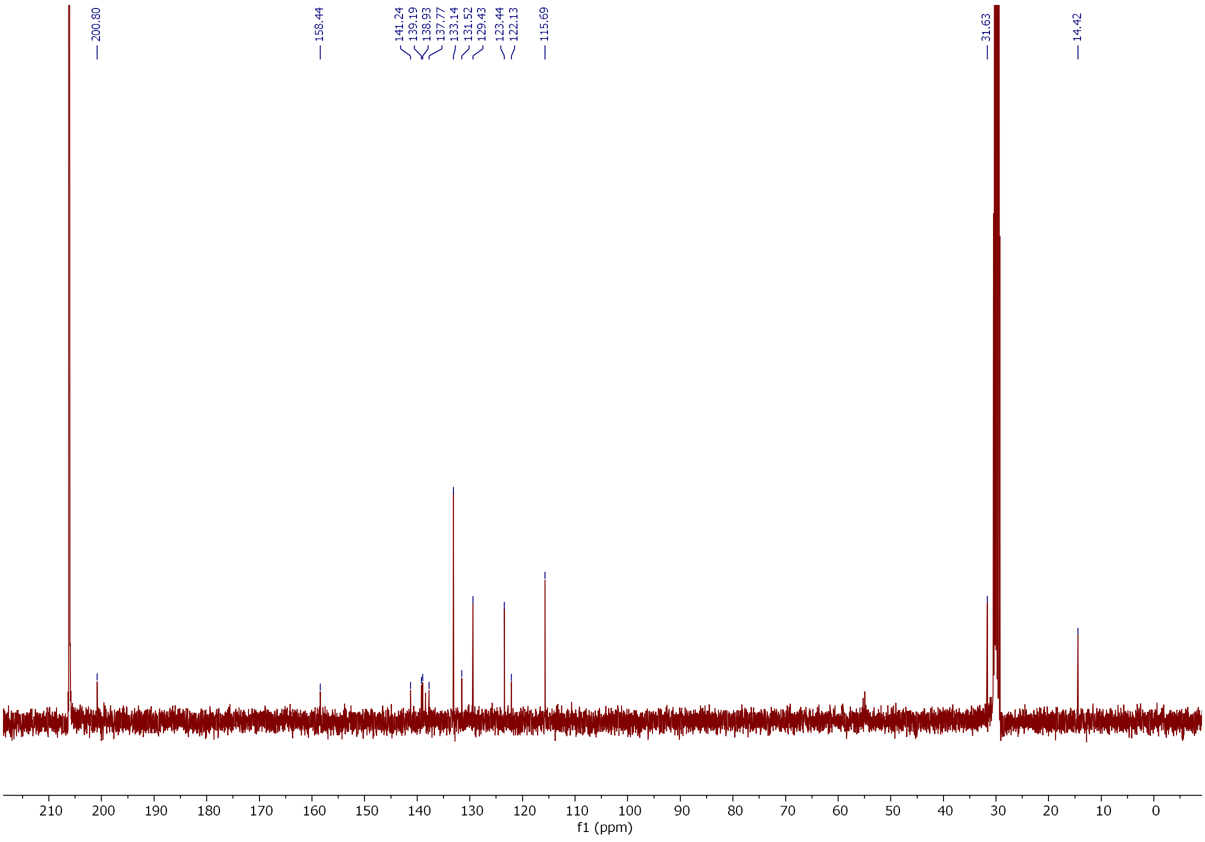

4y ^{1}H NMR (400 MHz, Acetone-d_6)

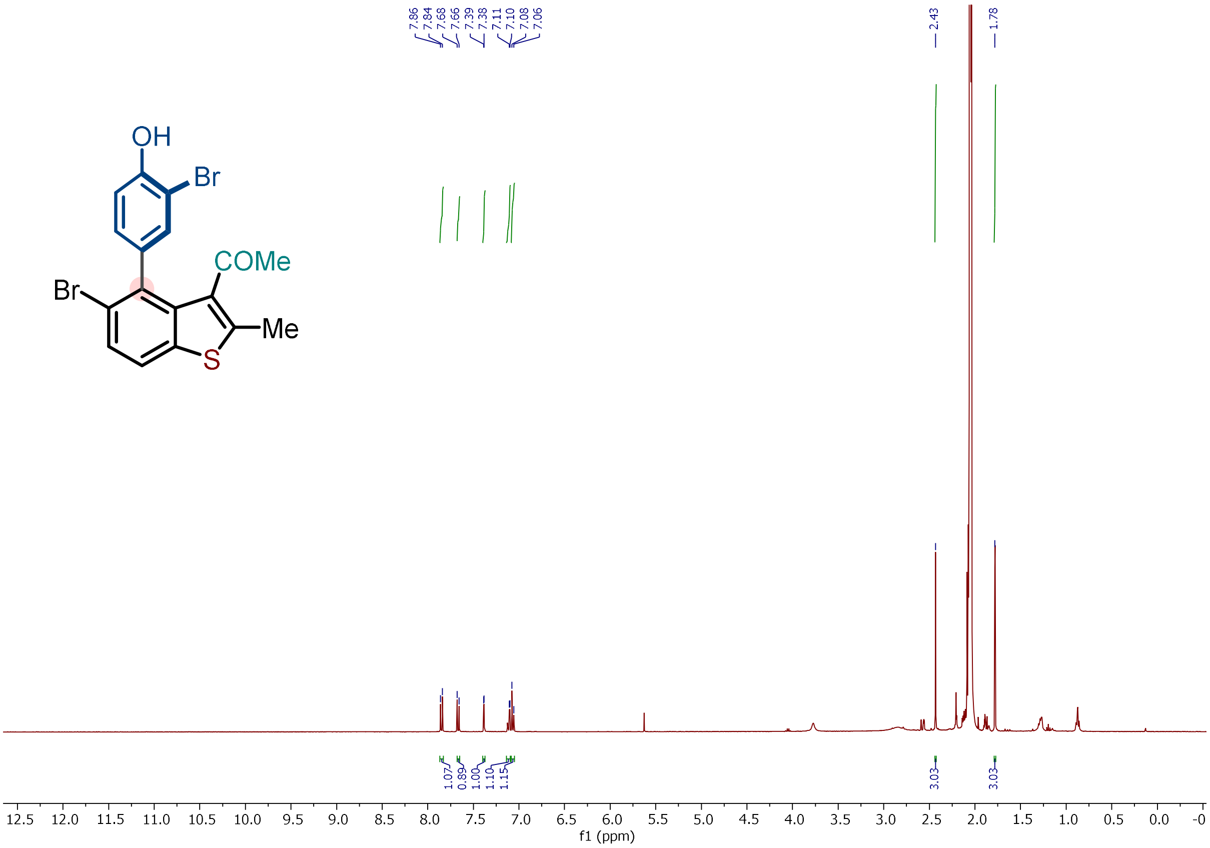

4y ^{13}C NMR (101 MHz, Acetone-d_6)

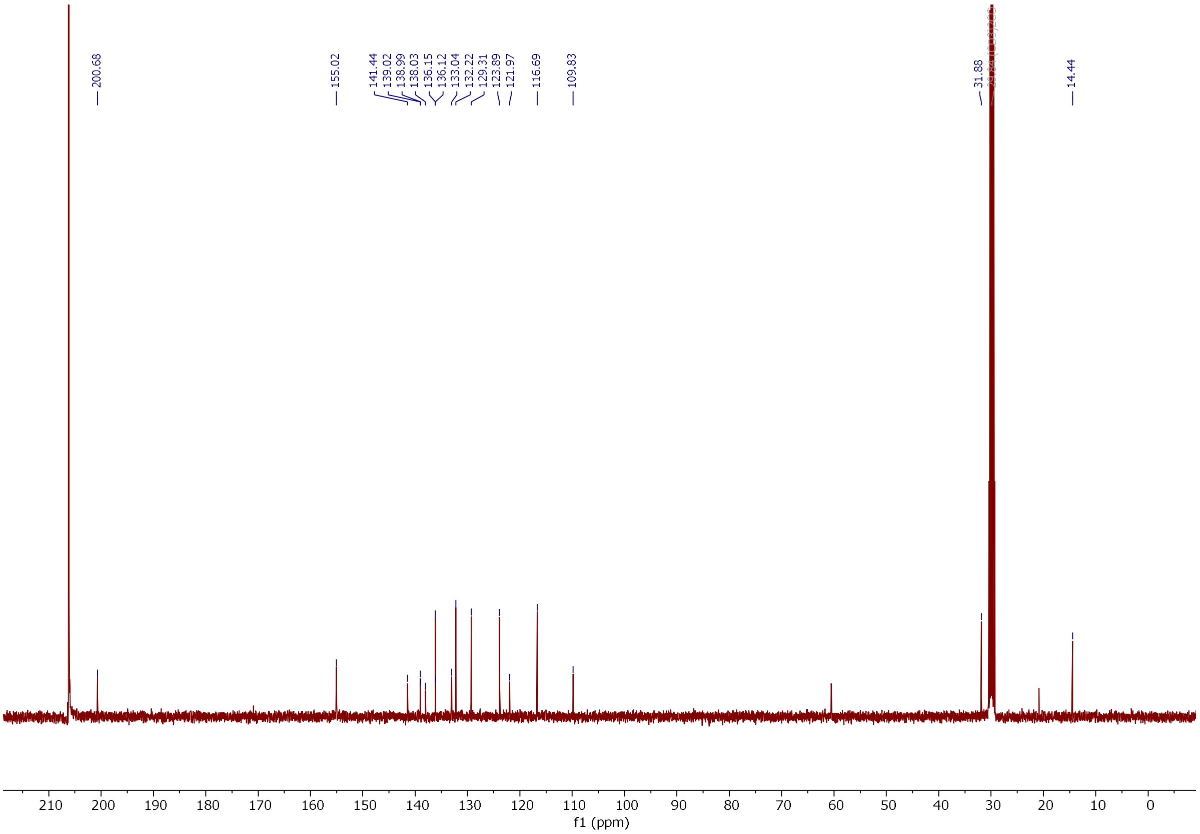

4z ^{1}H NMR (400 MHz, DMSO-d_6)

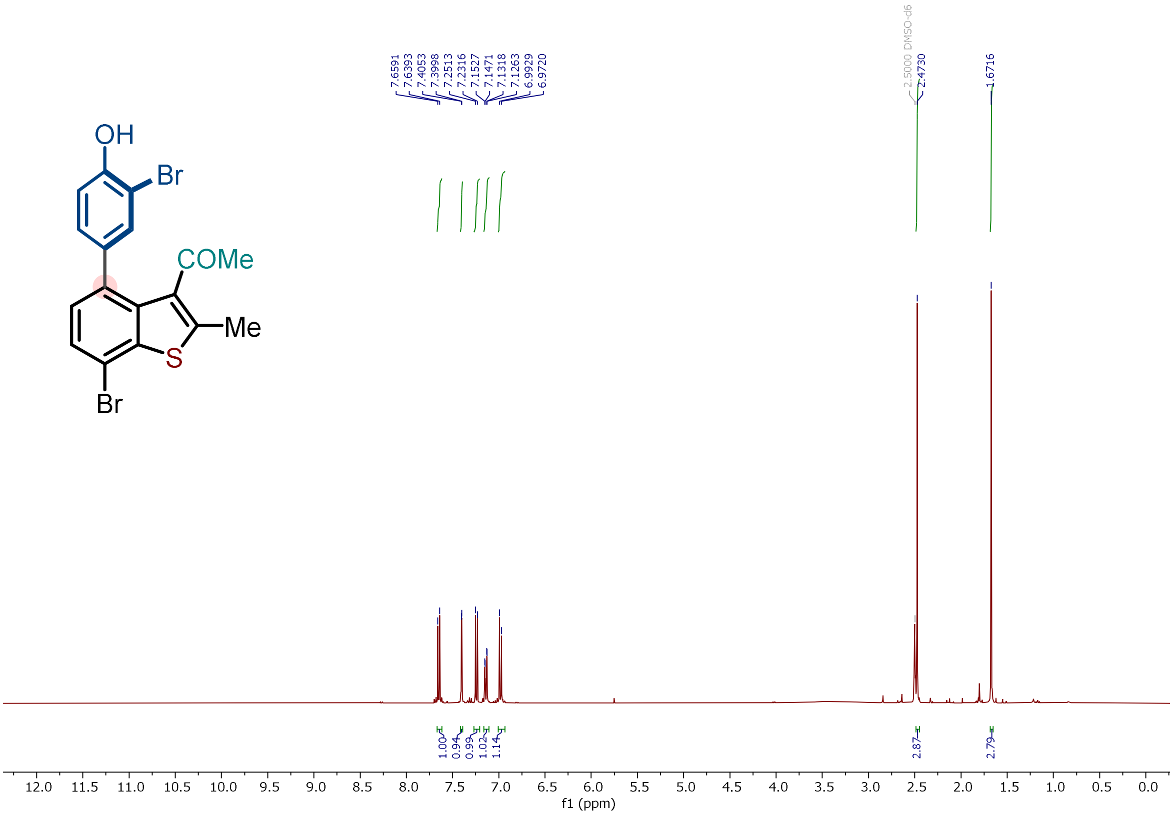

4z ^{13}C NMR (101 MHz, DMSO-d_6)

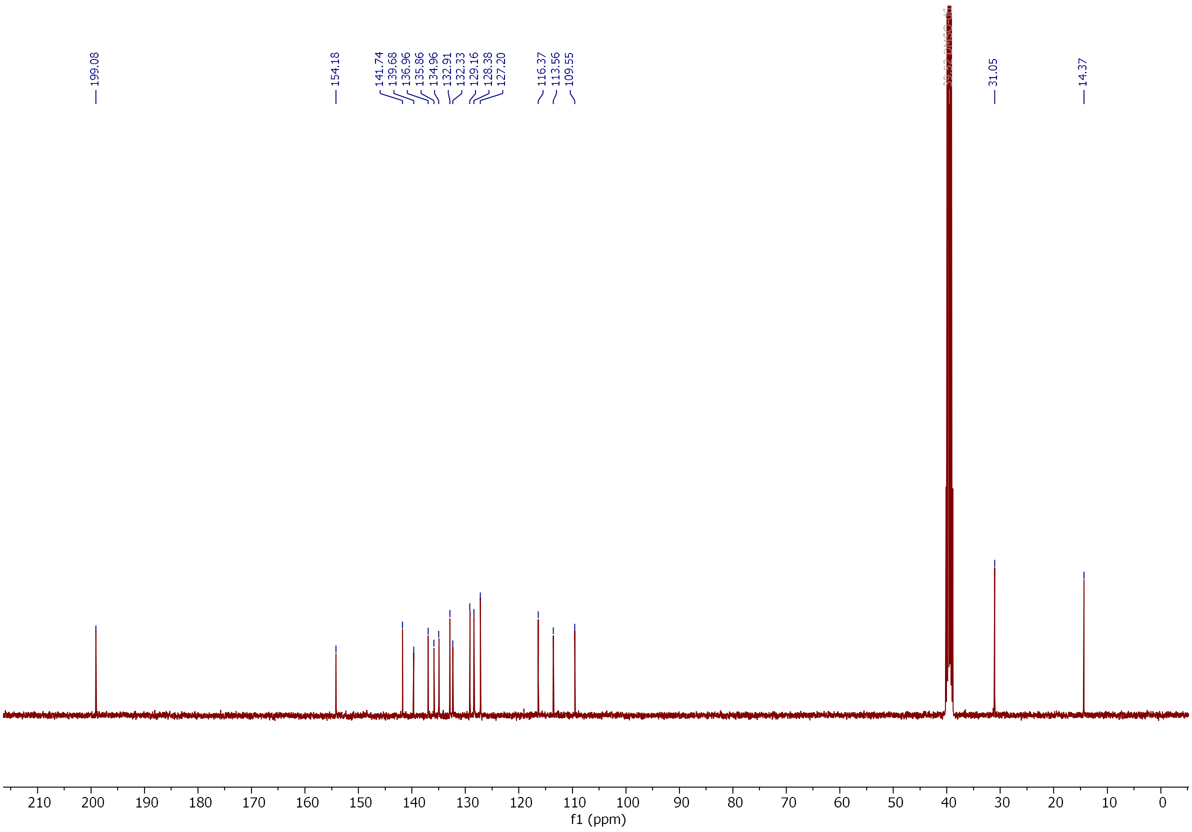

4aa ^{1}H NMR (500 MHz, $CDCl_3$)

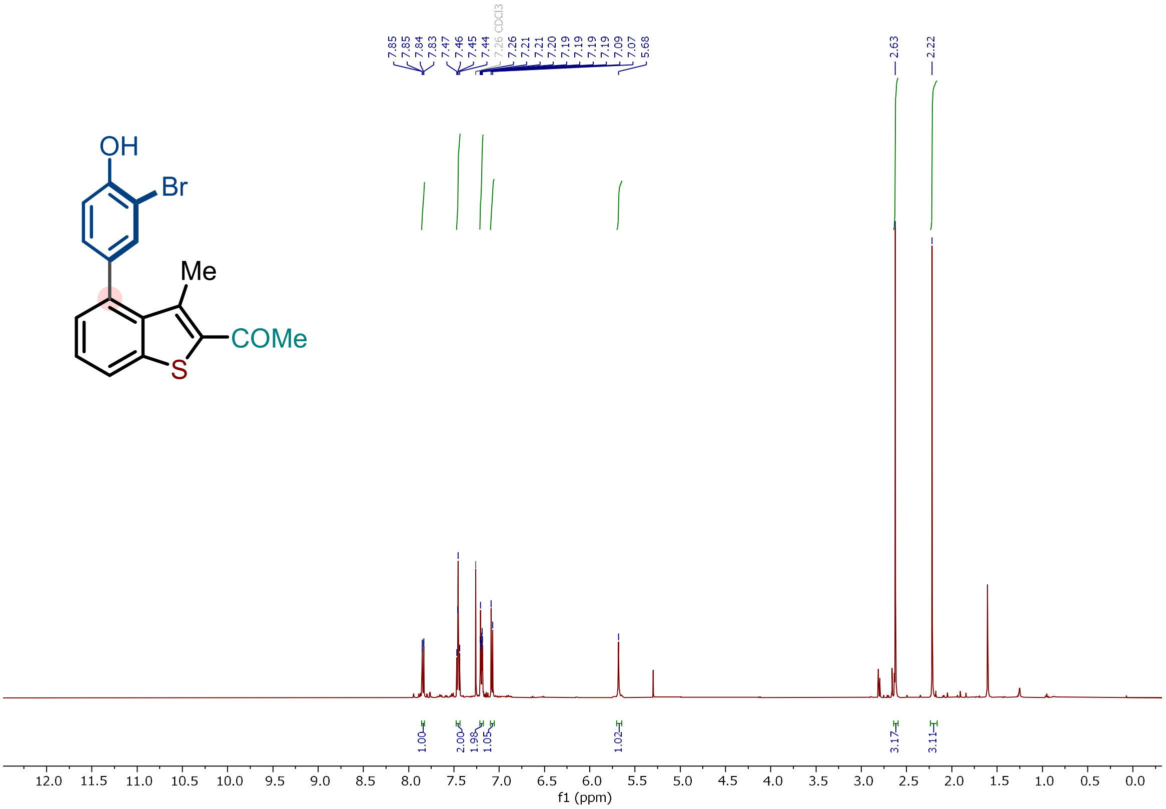

4aa ^{13}C NMR (126 MHz, $CDCl_3$)

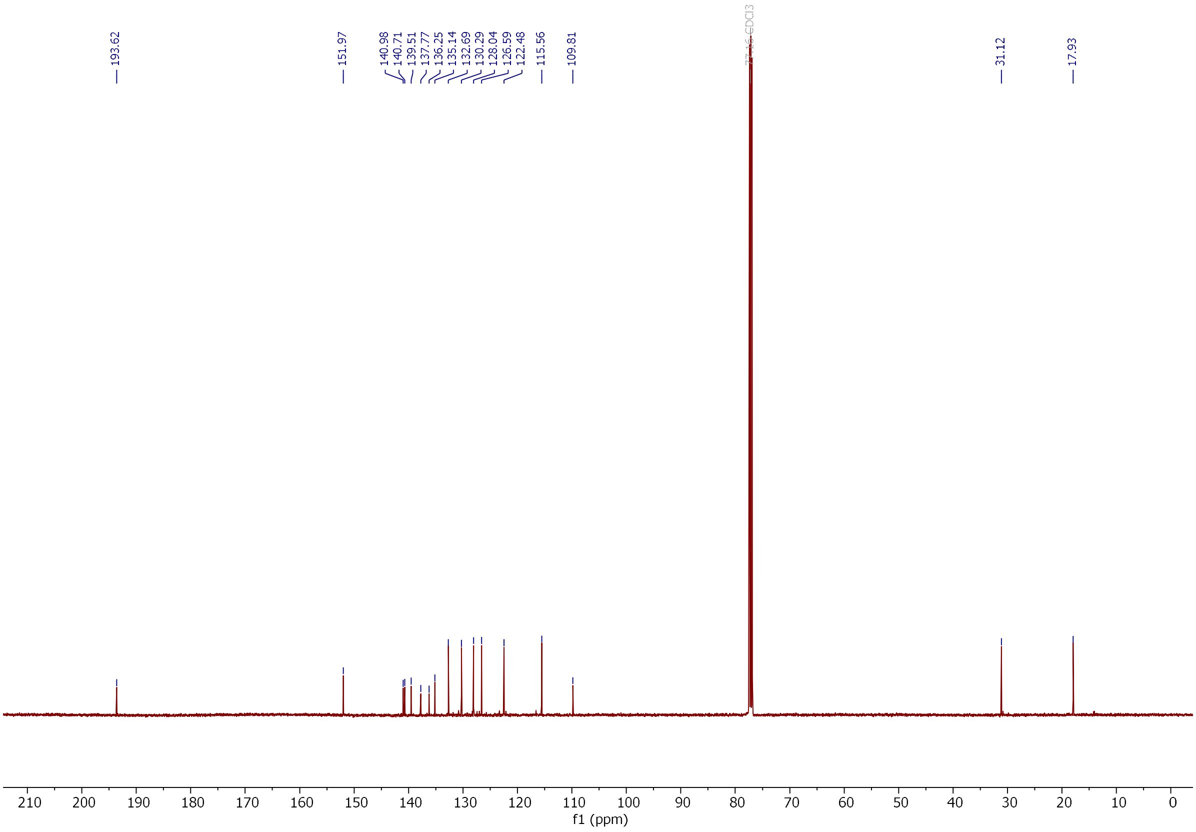

4ab ^{1}H NMR (400 MHz, Acetone-d_6)

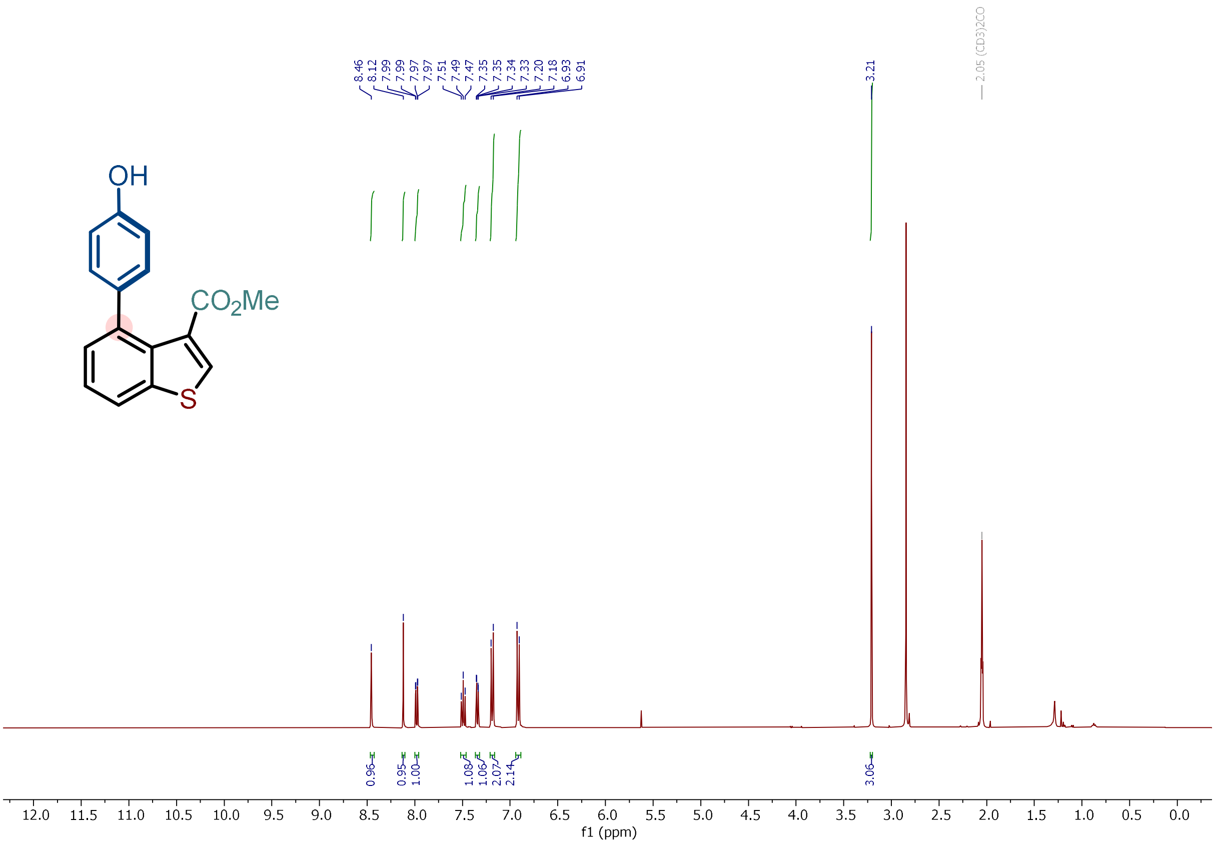

4ab ^{13}C NMR (101 MHz, Acetone-d_6)

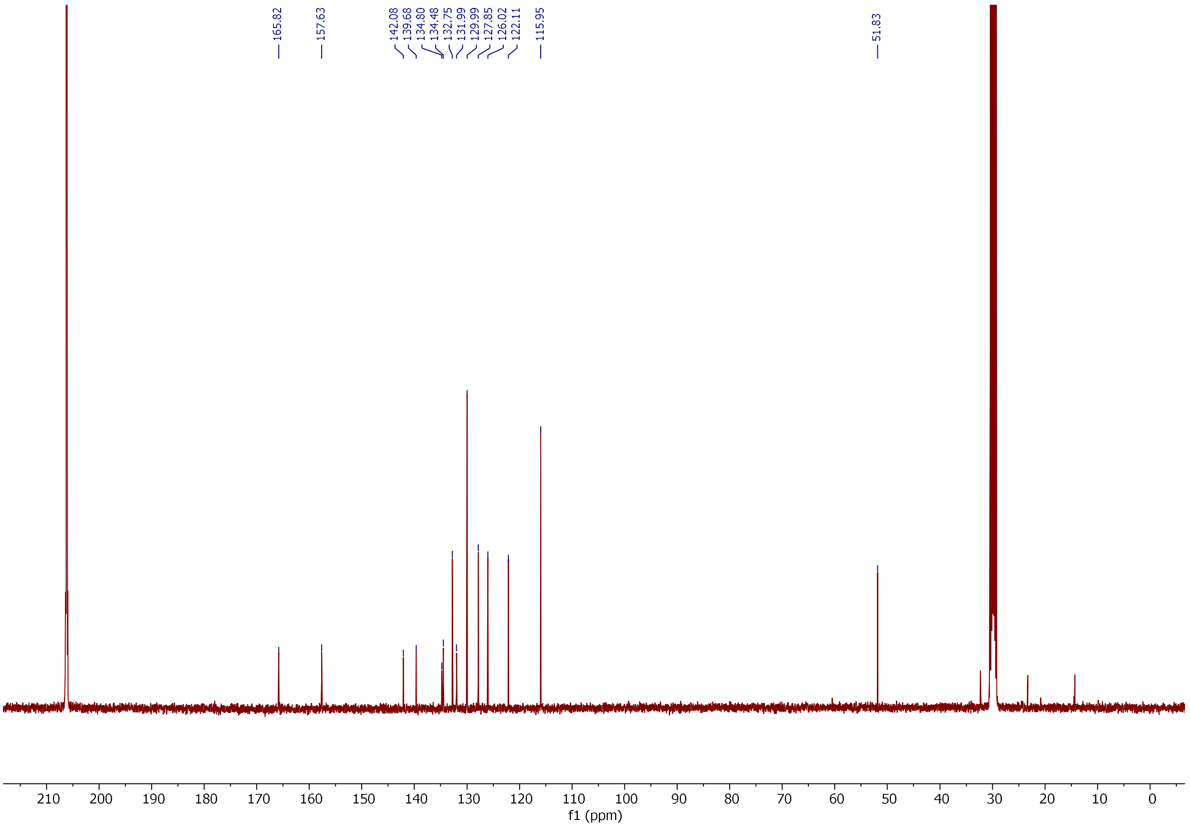

4ac ^{1}H NMR (400 MHz, Acetone-d$_6$)

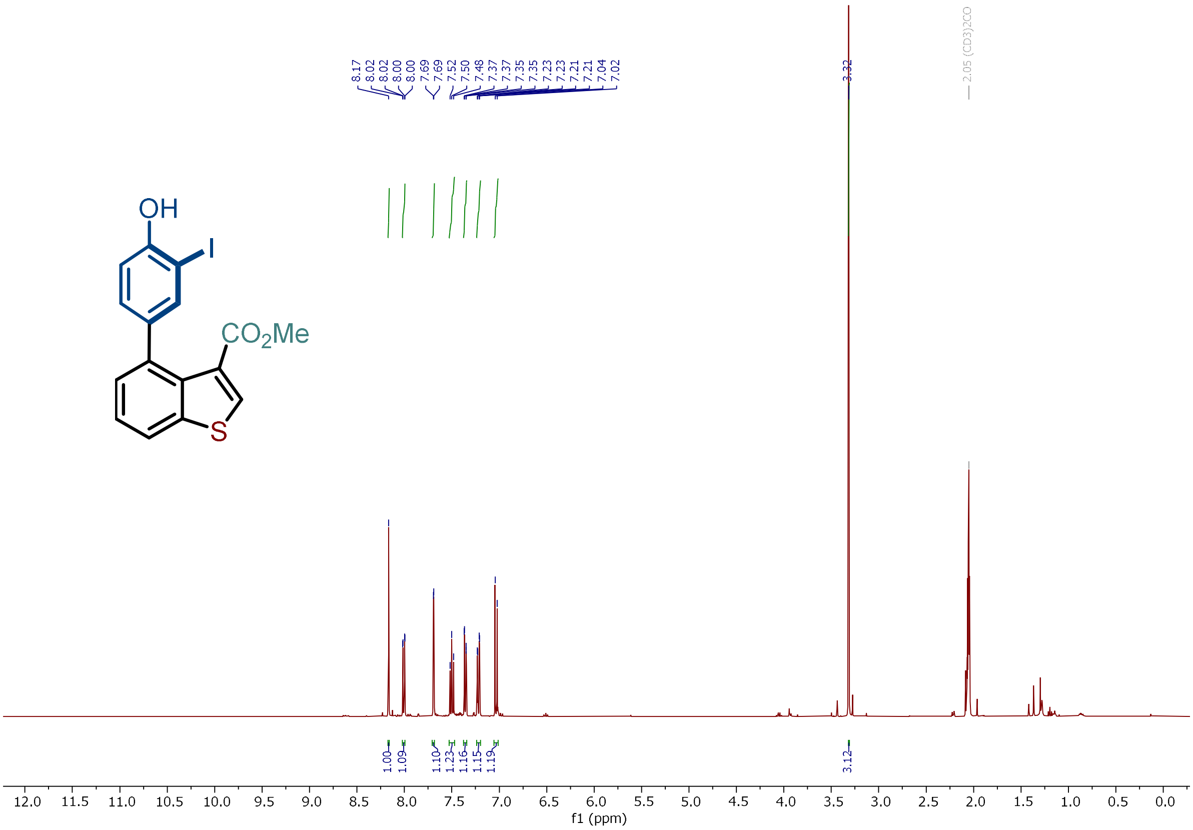

4ac ^{13}C NMR (101MHz, Acetone-d$_6$)

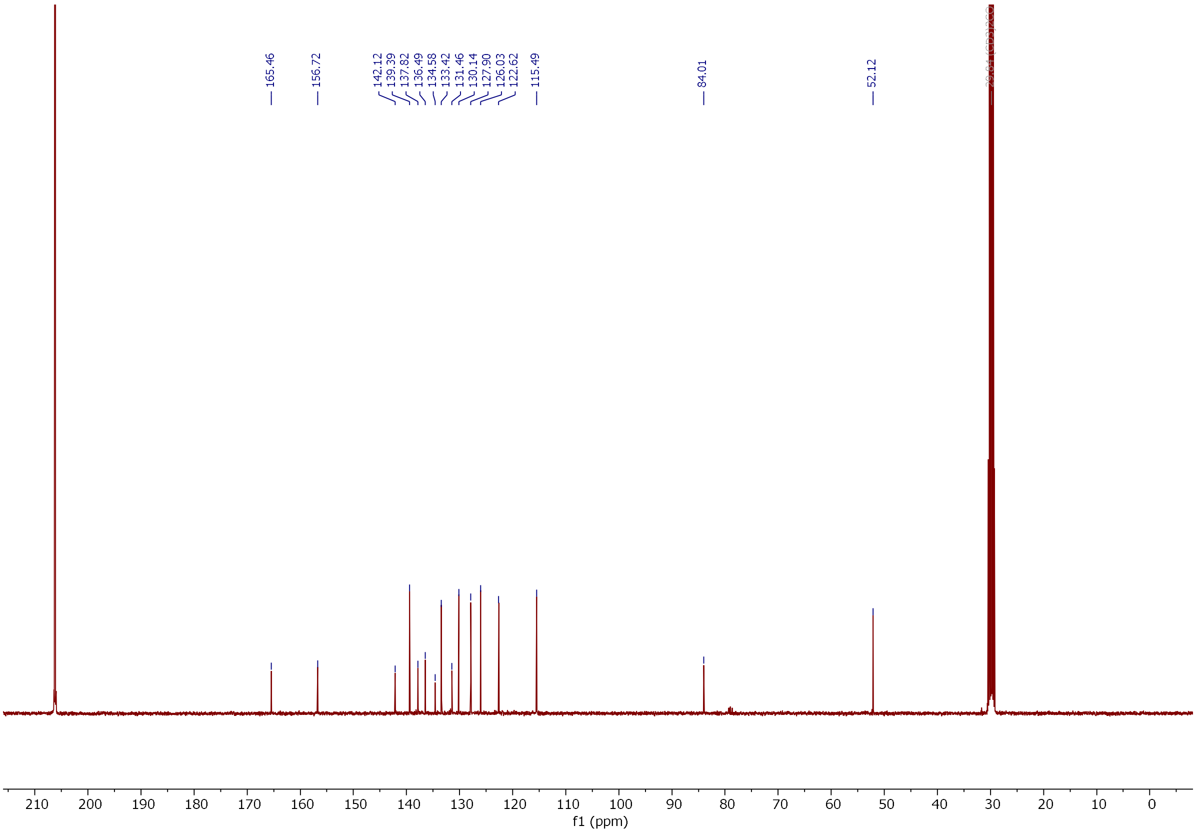

4ad ^{1}H NMR (400 MHz, Acetone-d$_6$)

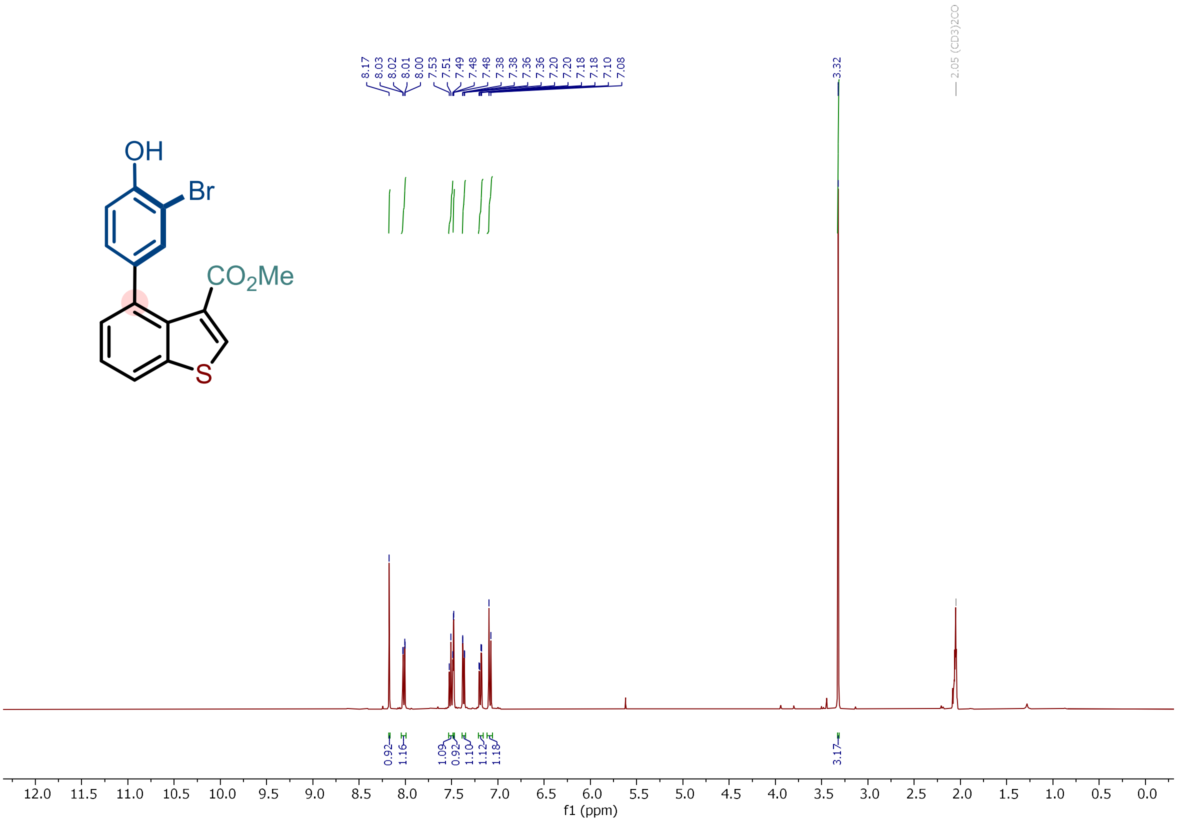

4ad ^{13}C NMR (101MHz, Acetone-d$_6$)

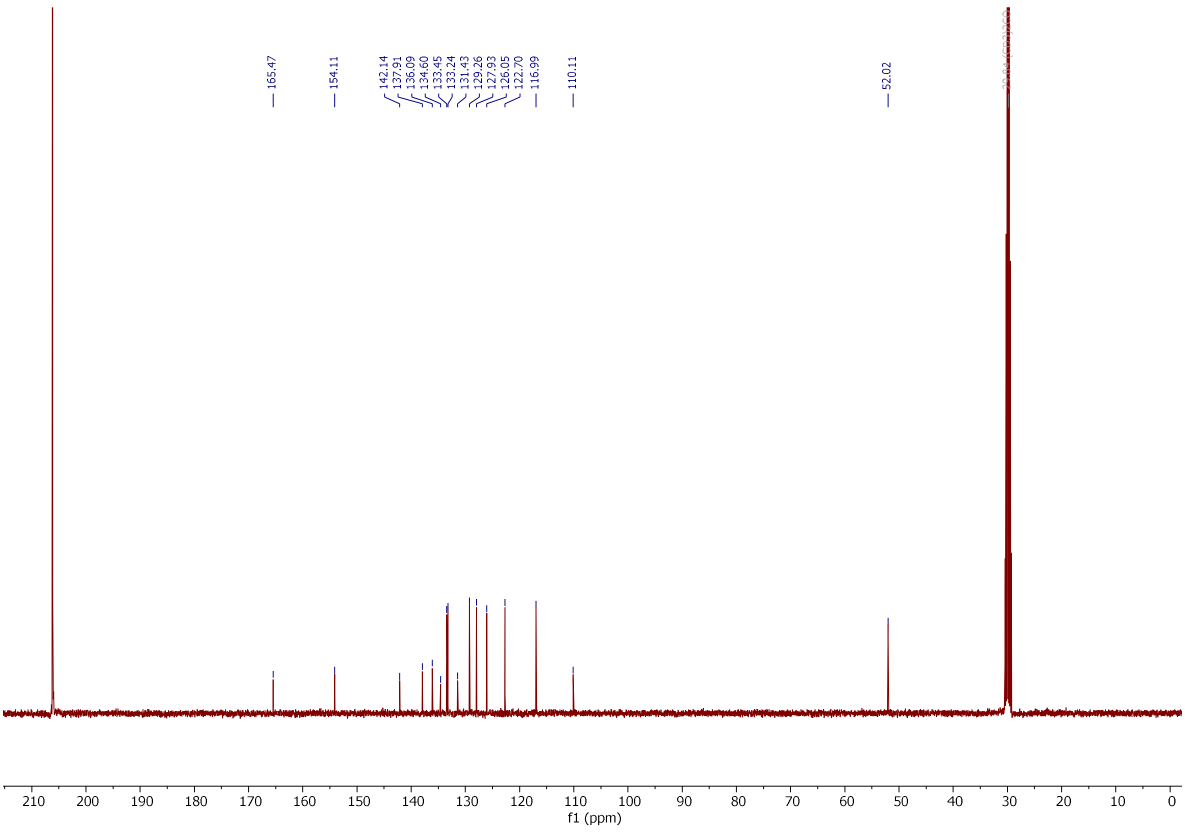

4ae ^{1}H NMR (400 MHz, $CDCl_3$)

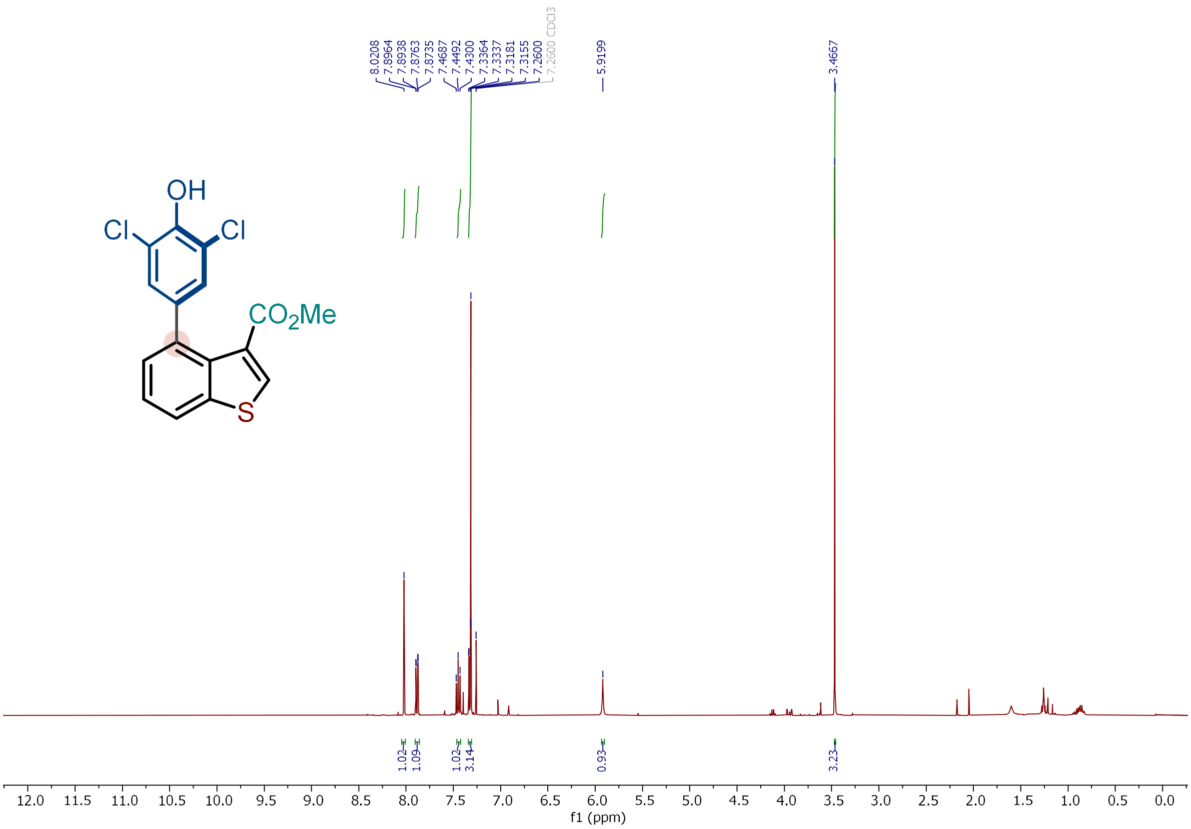

4ae ^{13}C NMR (101 MHz, $CDCl_3$)

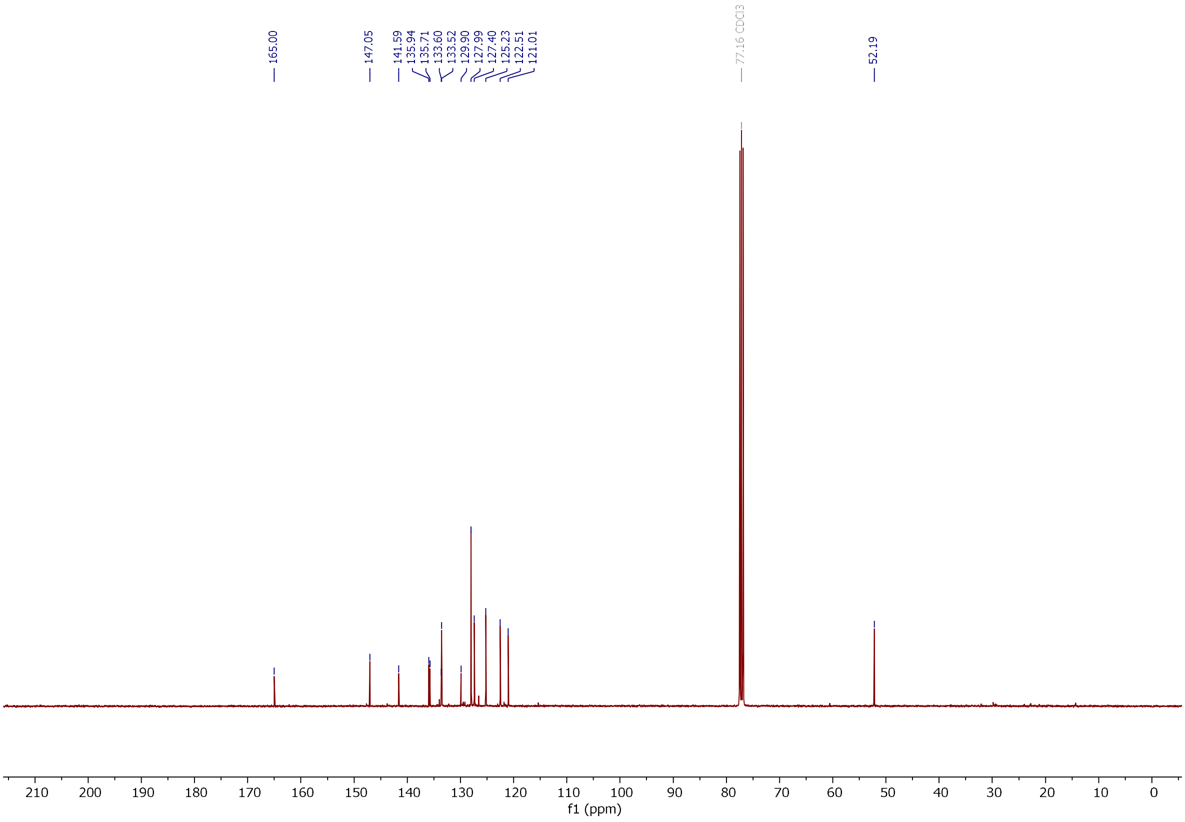

4af ^{1}H NMR (400 MHz, $CDCl_3$)

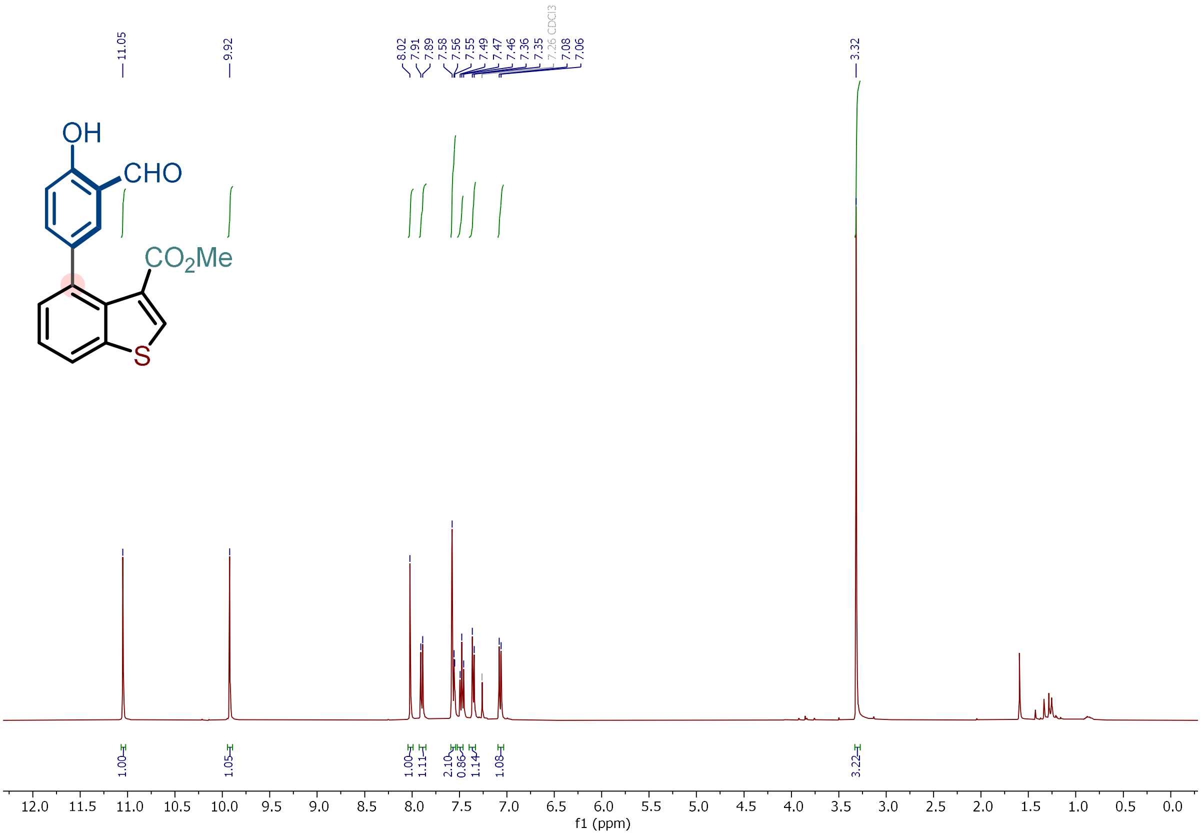

4af ^{13}C NMR (101 MHz, $CDCl_3$)

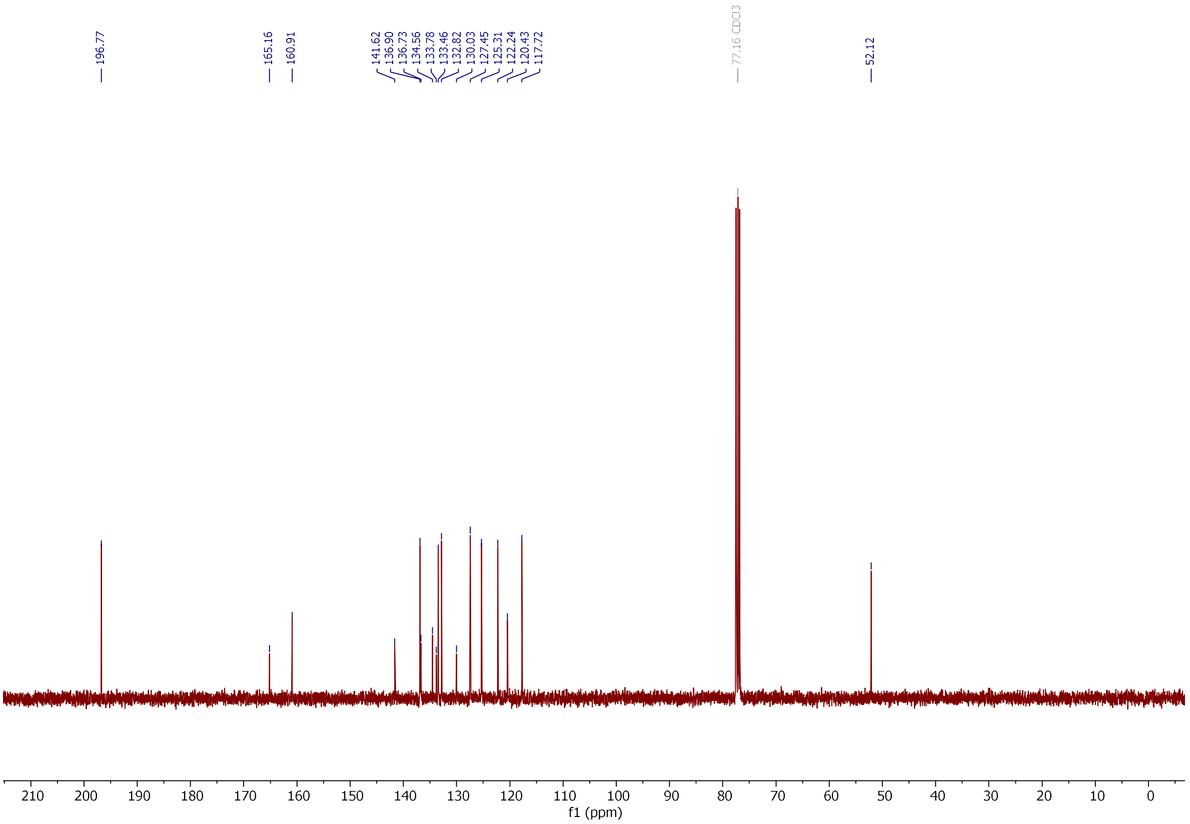

4ag ^{1}H NMR (400 MHz, Acetone-d$_6$)

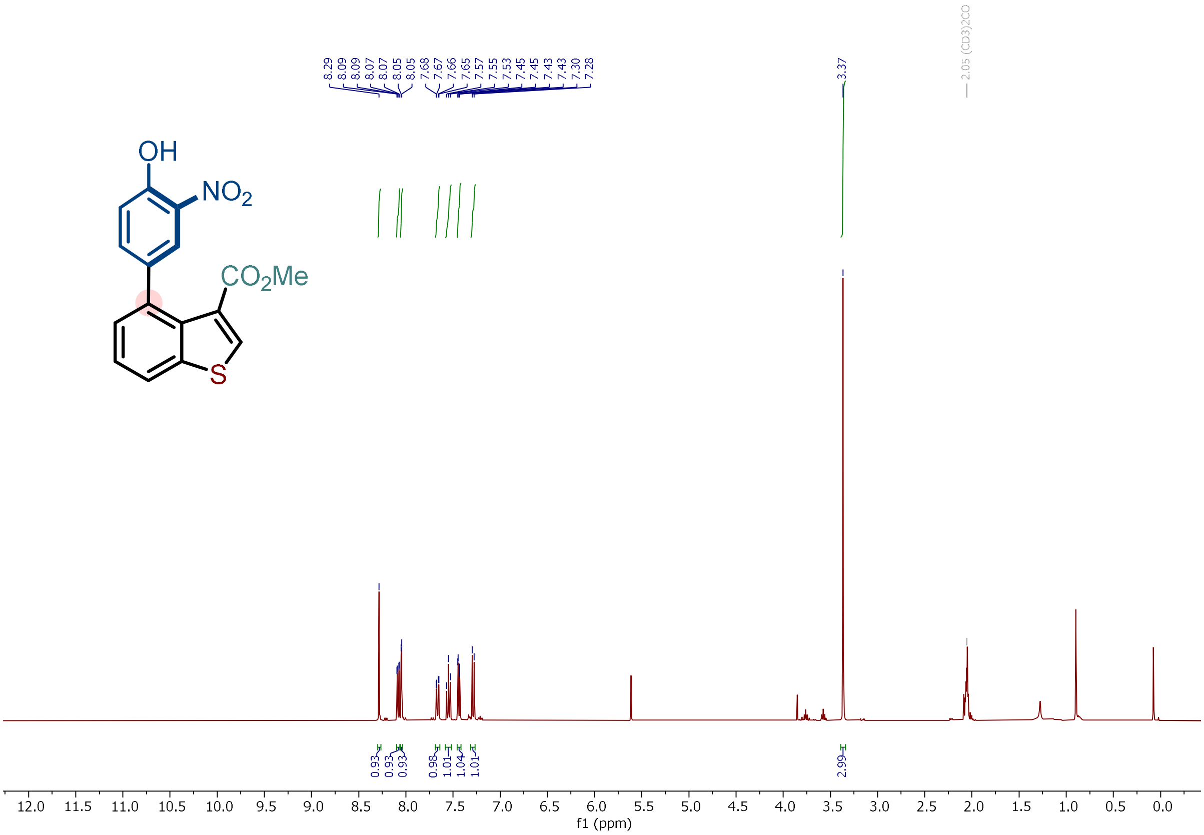

4ag ^{13}C NMR (101 MHz, Acetone-d$_6$)

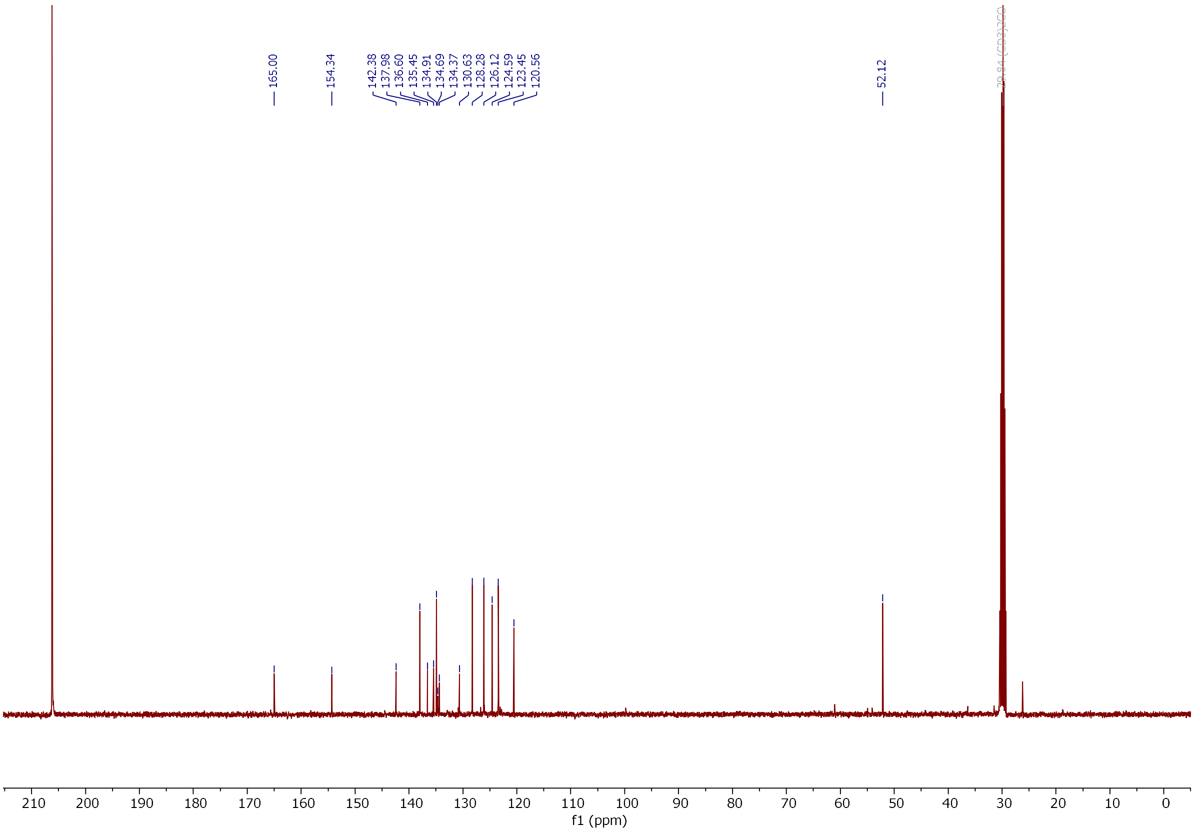

4ah ^{1}H NMR (400 MHz, $CDCl_3$)

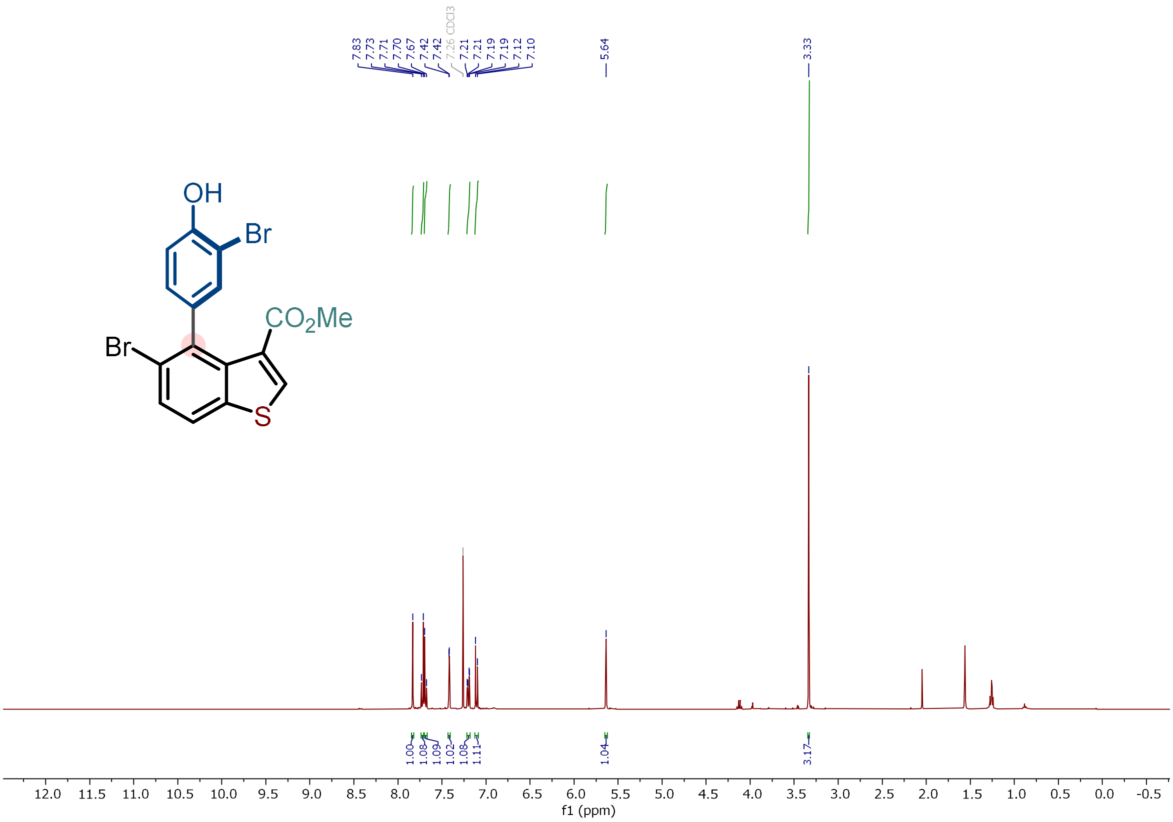

4ah ^{13}C NMR (101 MHz, $CDCl_3$)

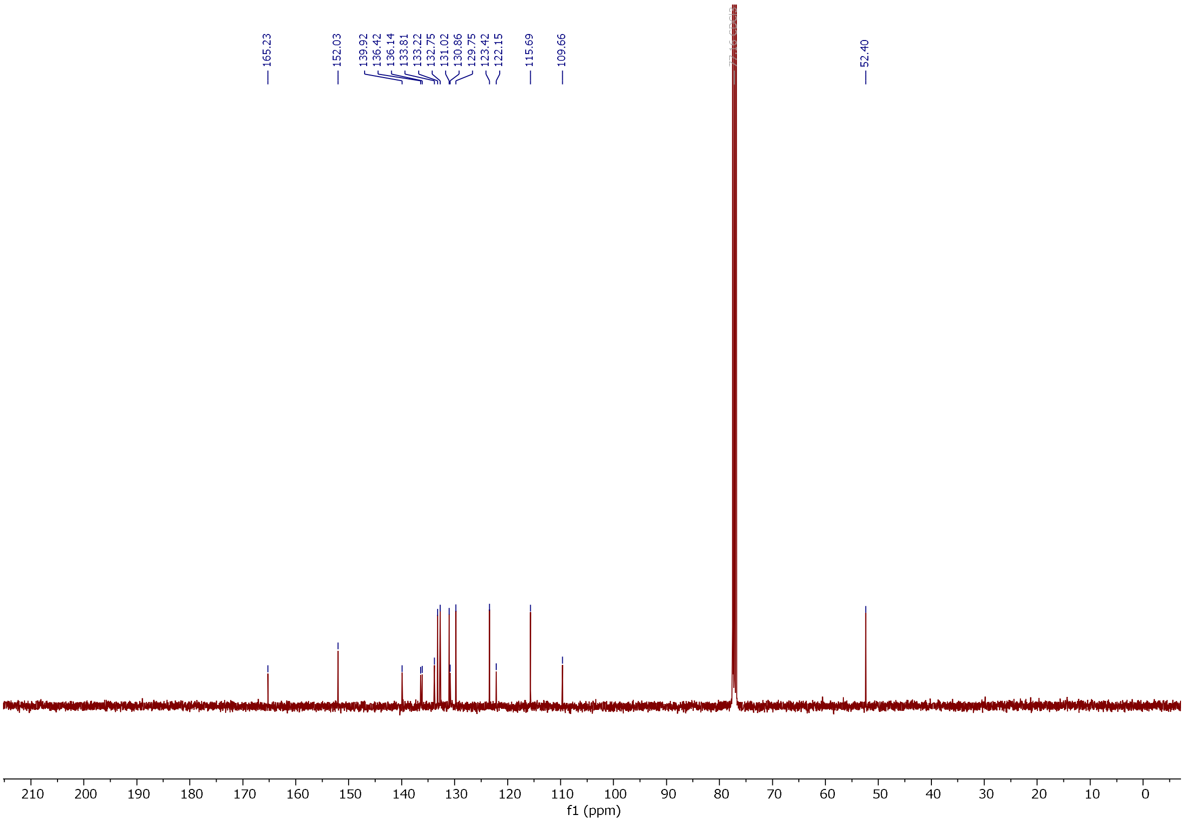

4ai ^{1}H NMR (400 MHz, Acetone-d_6)

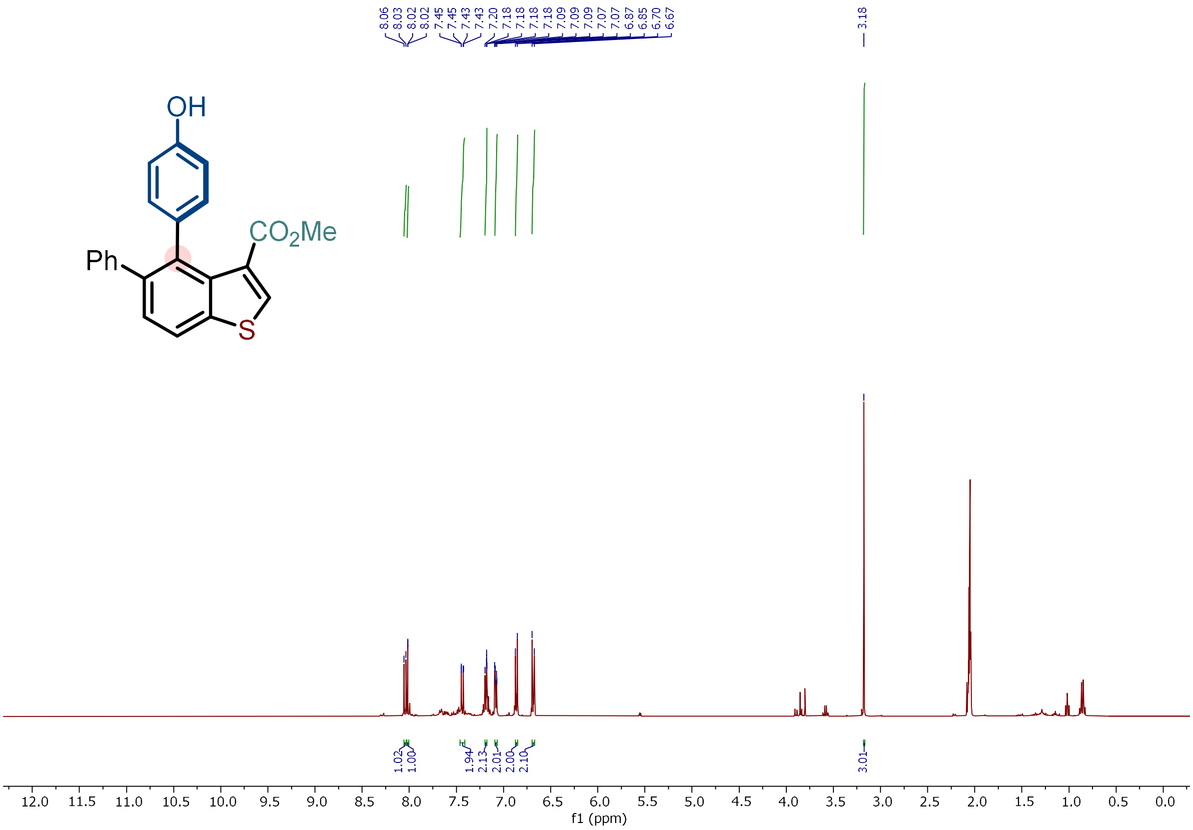

4ai ^{13}C NMR (101 MHz, Acetone-d_6)

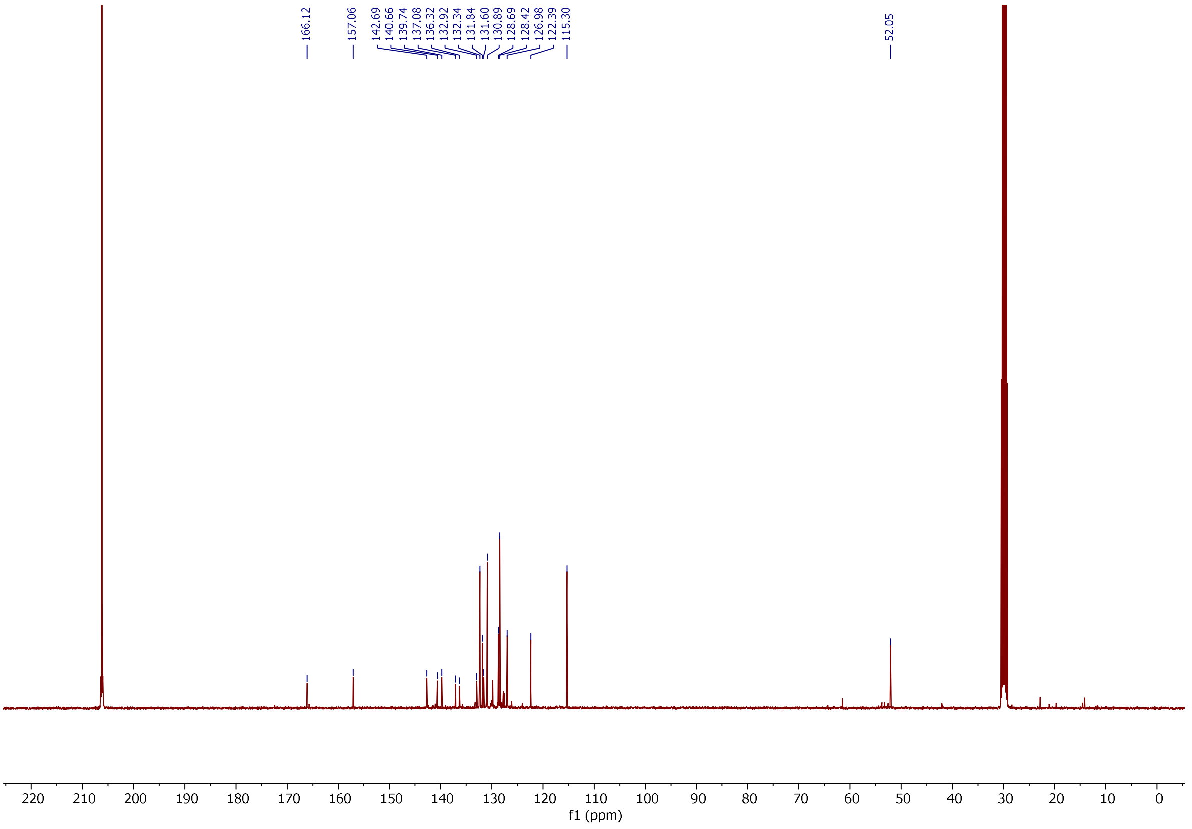

4aj ^{1}H NMR (400 MHz, $CDCl_3$)

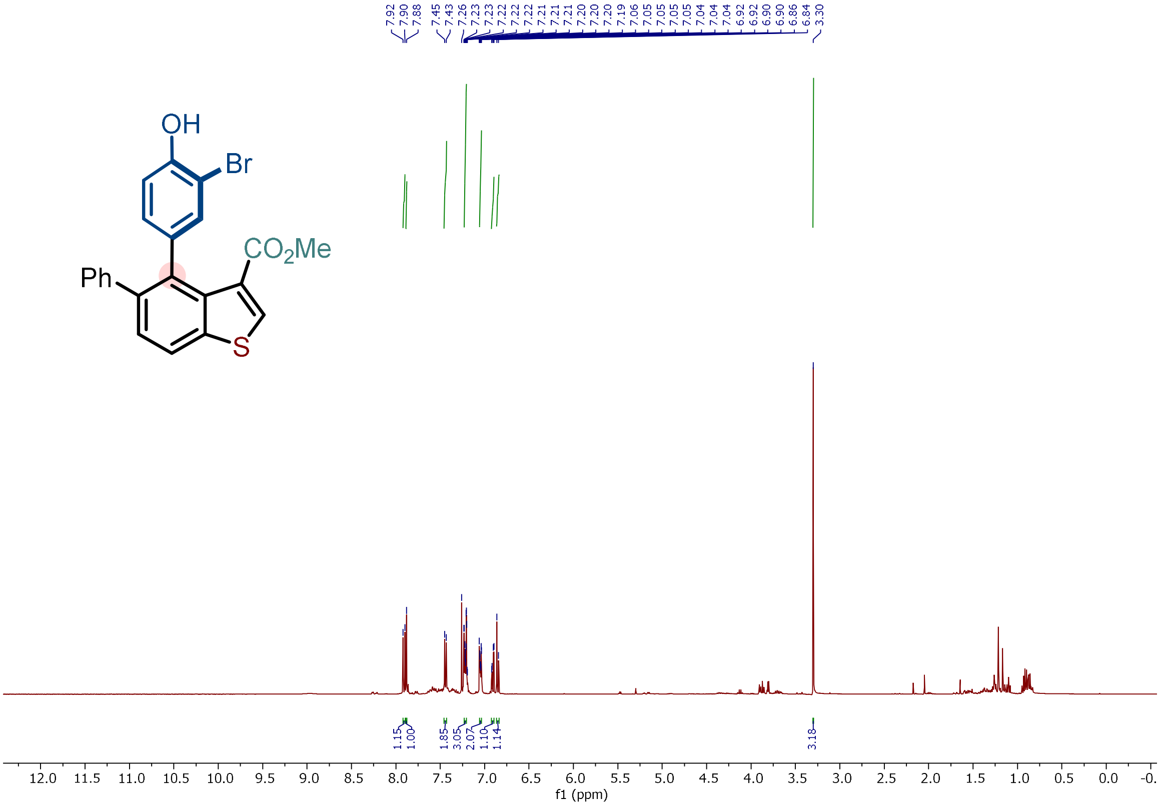

4aj ^{13}C NMR (101 MHz, $CDCl_3$)

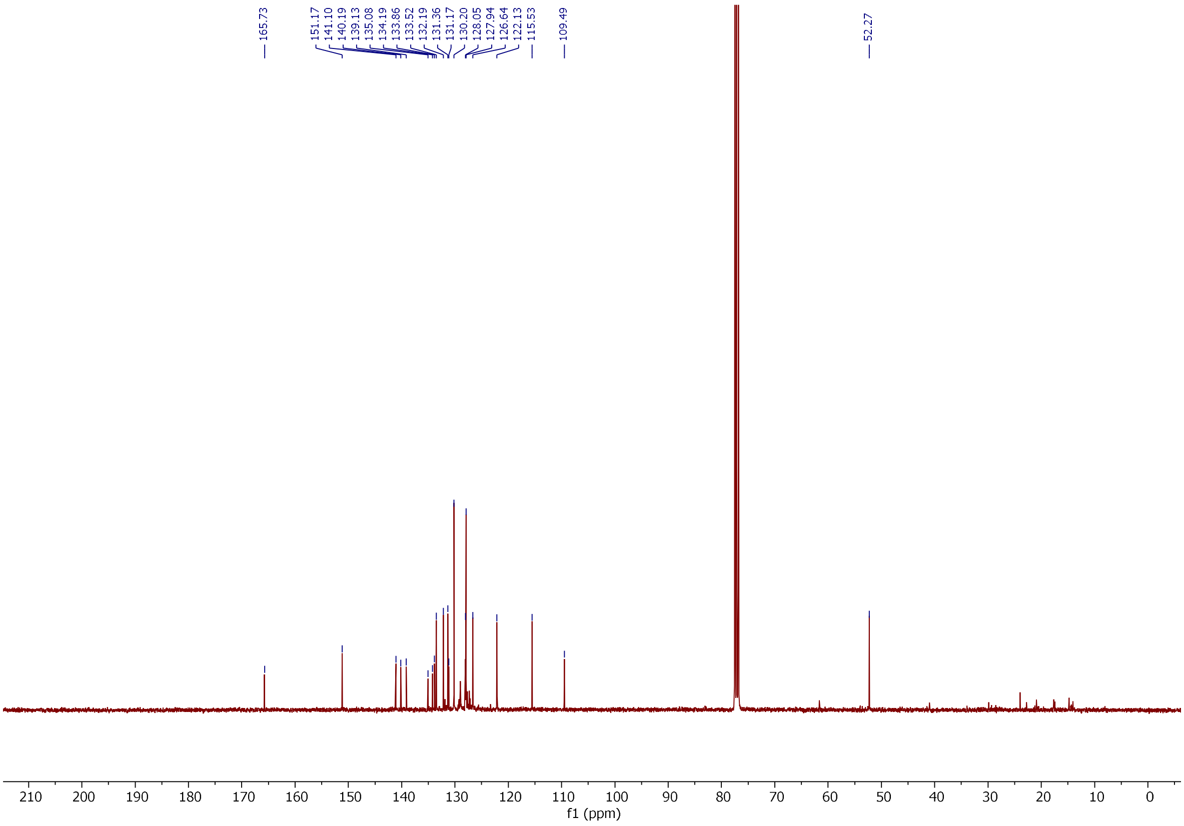

4ak ^{1}H NMR (400 MHz, $CDCl_3$)

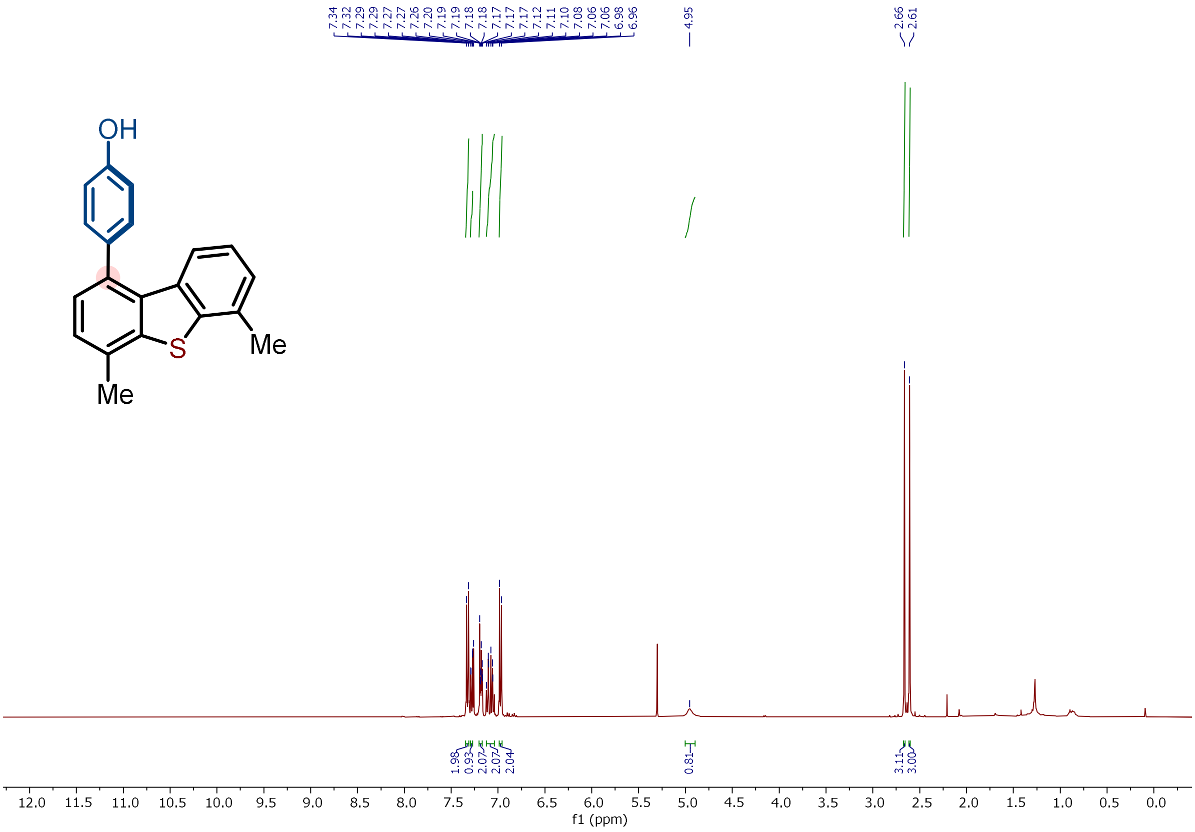

4ak ^{13}C NMR (101 MHz, $CDCl_3$)

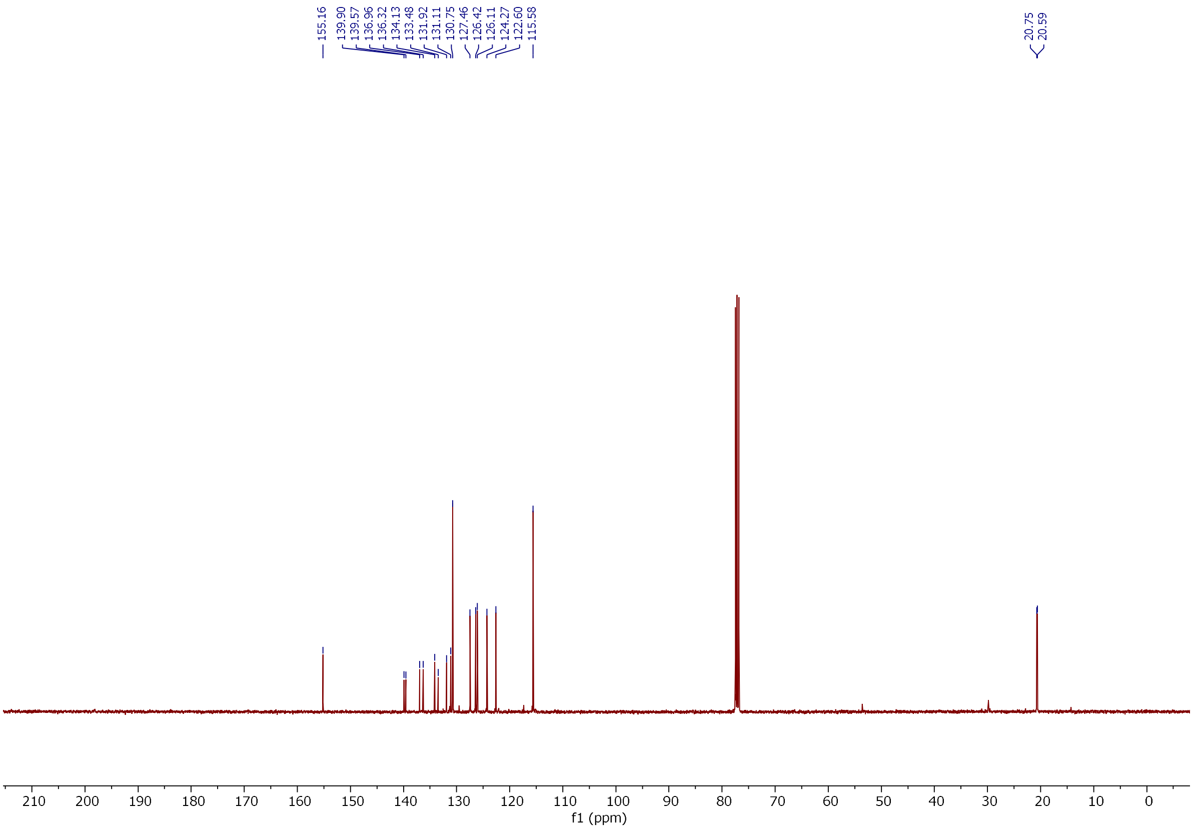

4al ^{1}H NMR (400 MHz, $CDCl_3$)

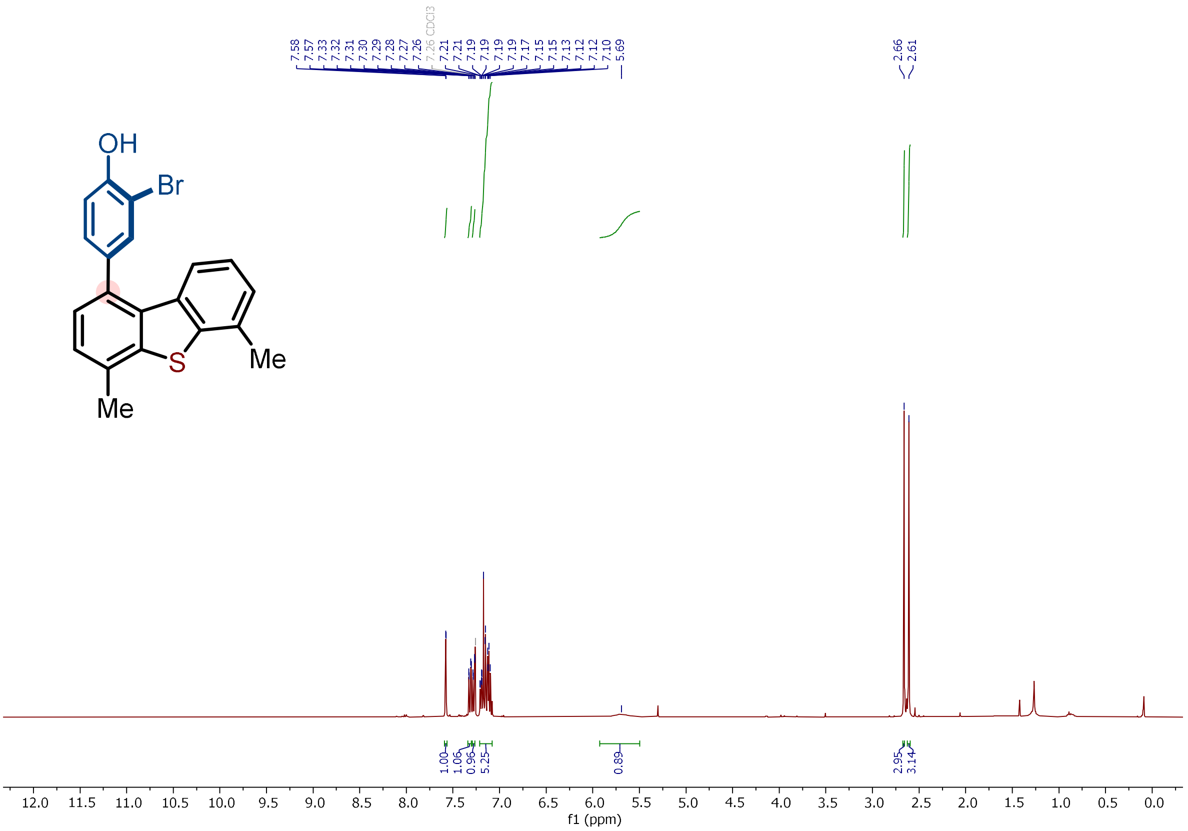

4al ^{13}C NMR (101 MHz, $CDCl_3$)

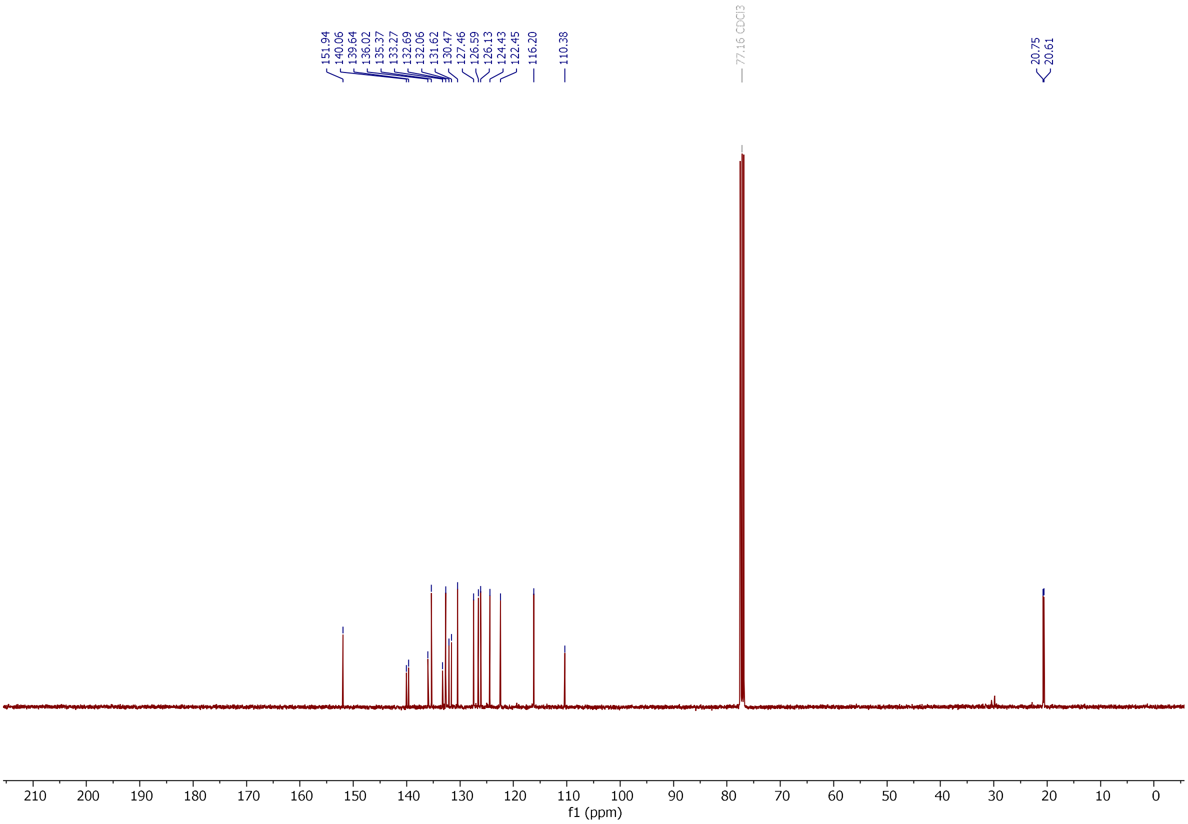

4am ^{1}H NMR (400 MHz, $CDCl_3$)

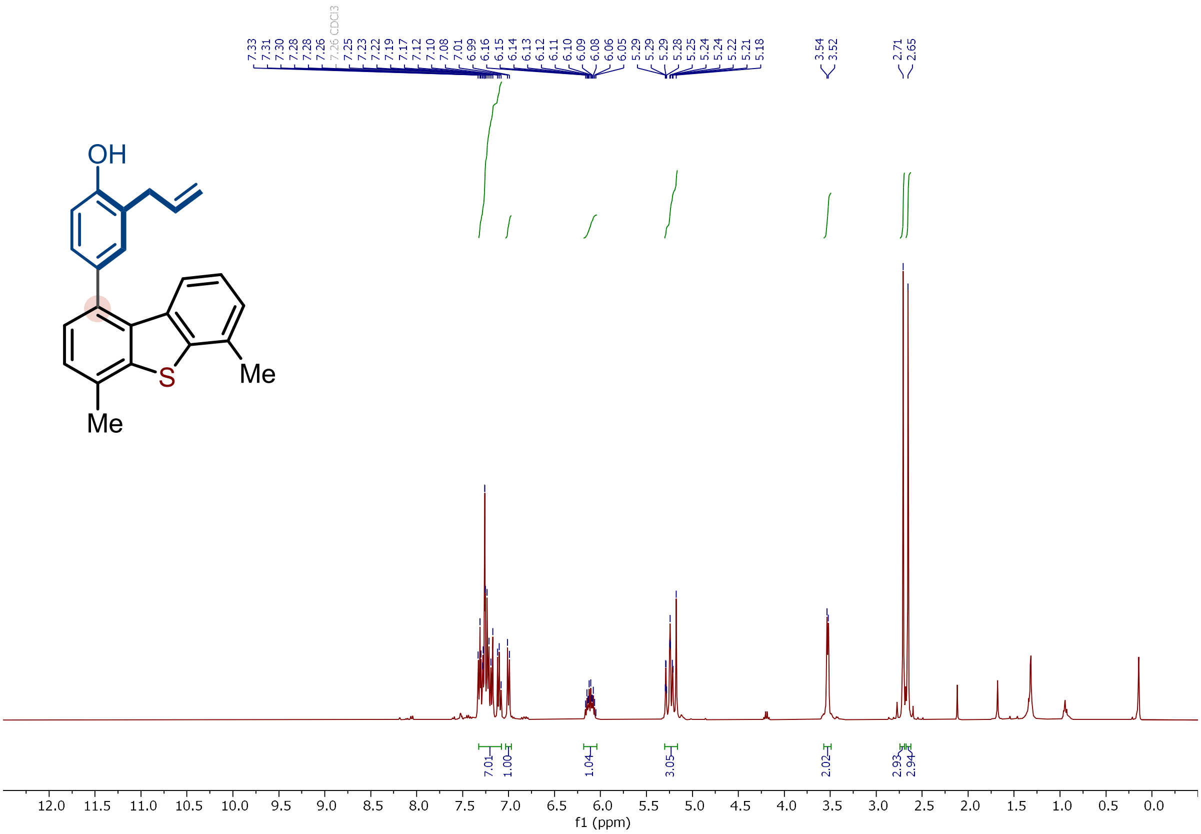

4am ^{13}C NMR (101 MHz, $CDCl_3$)

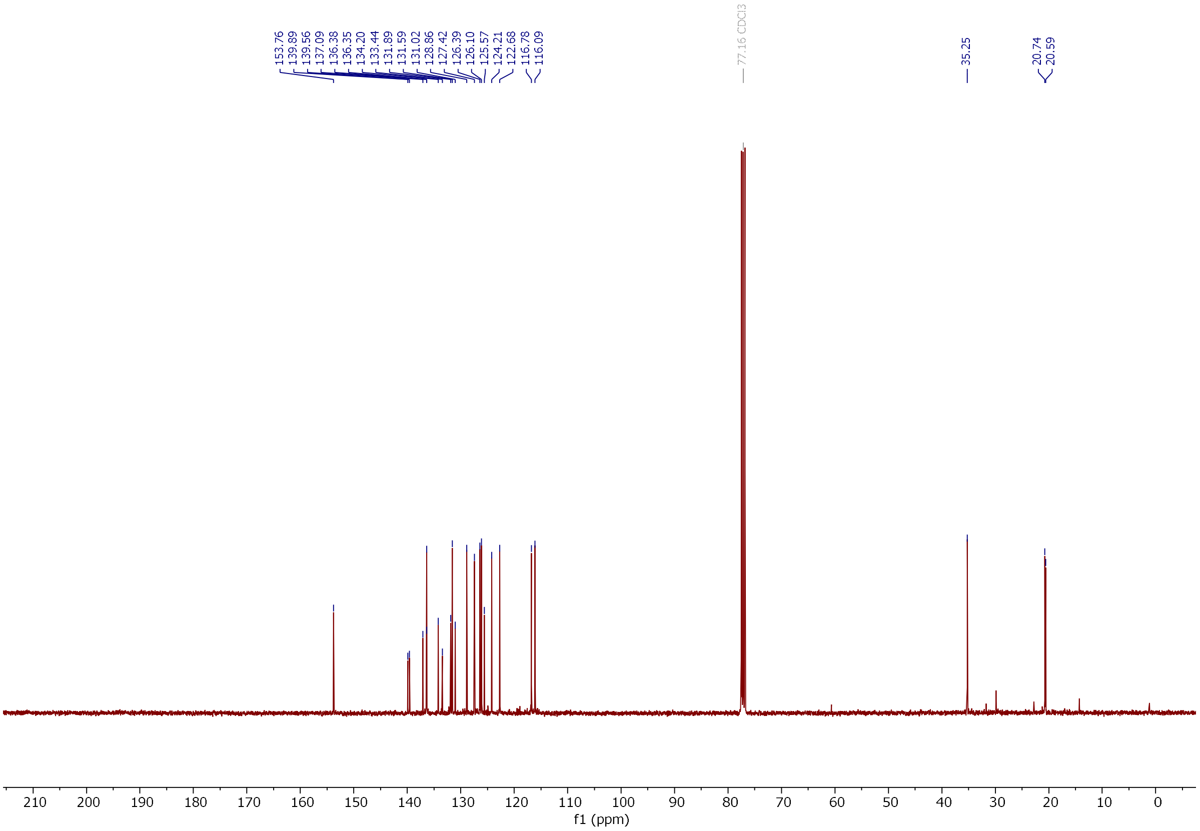

4an ^{1}H NMR (400 MHz, $CDCl_3$)

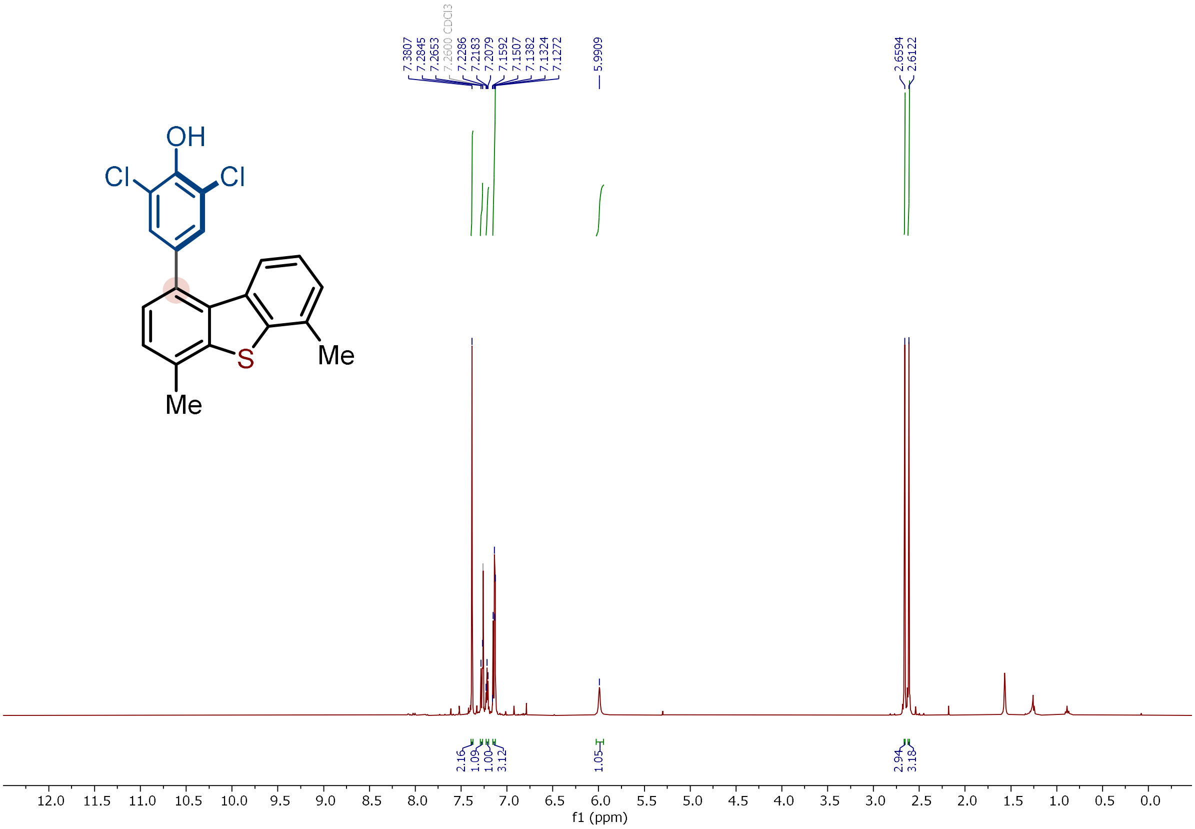

4an ^{13}C NMR (101 MHz, $CDCl_3$)

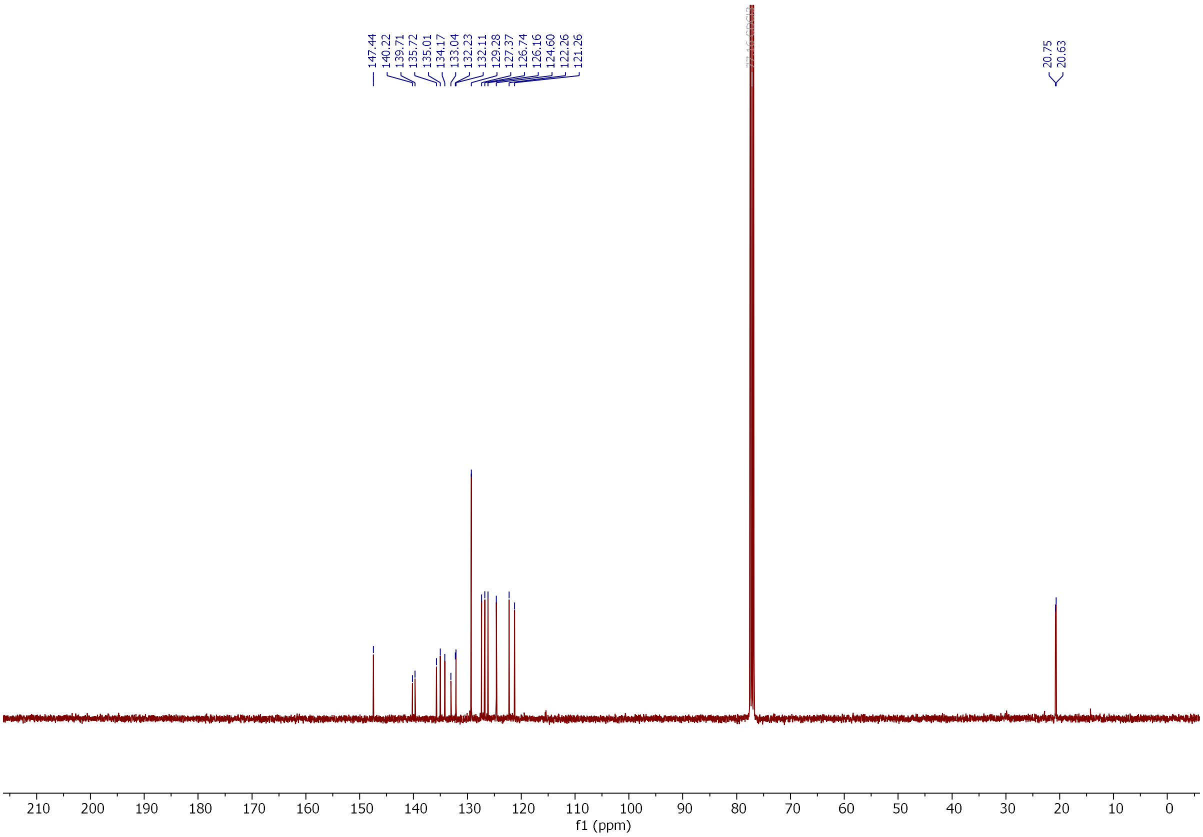

4ao ^{1}H NMR (400 MHz, $CDCl_3$)

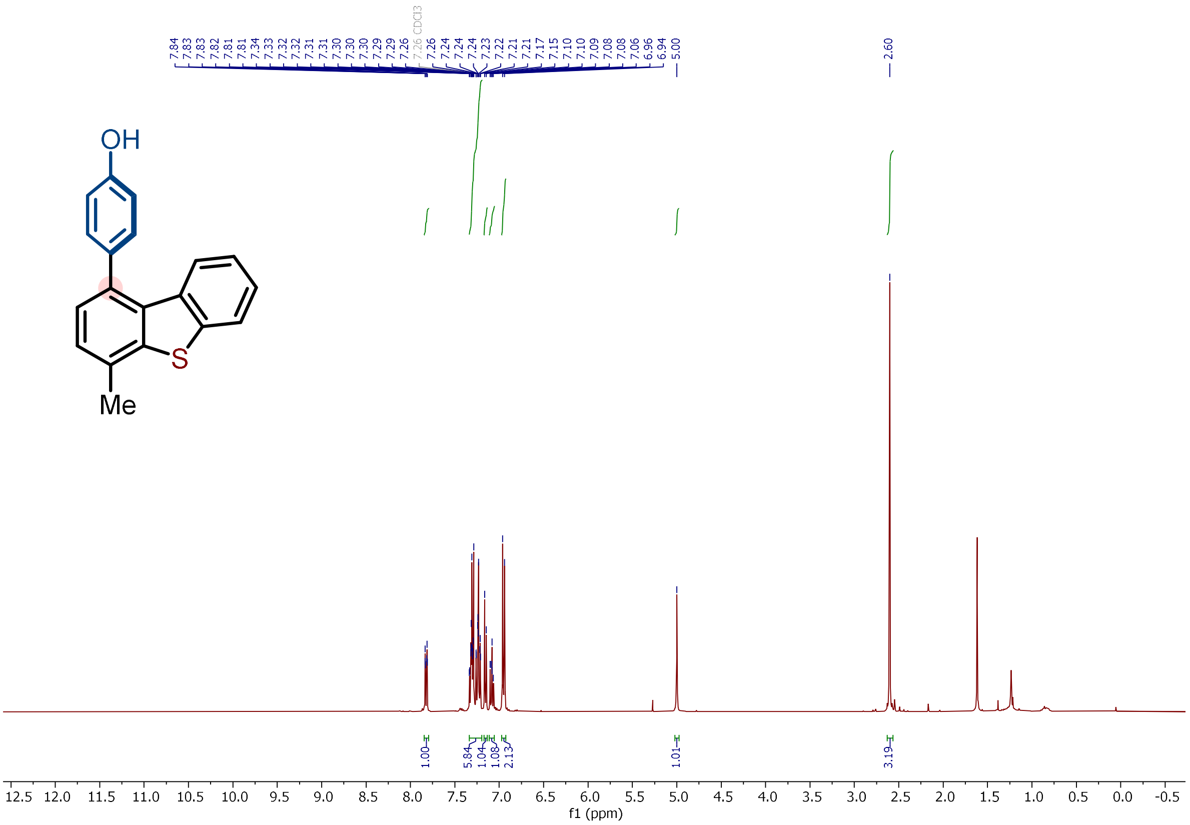

4ao ^{13}C NMR (101 MHz, $CDCl_3$)

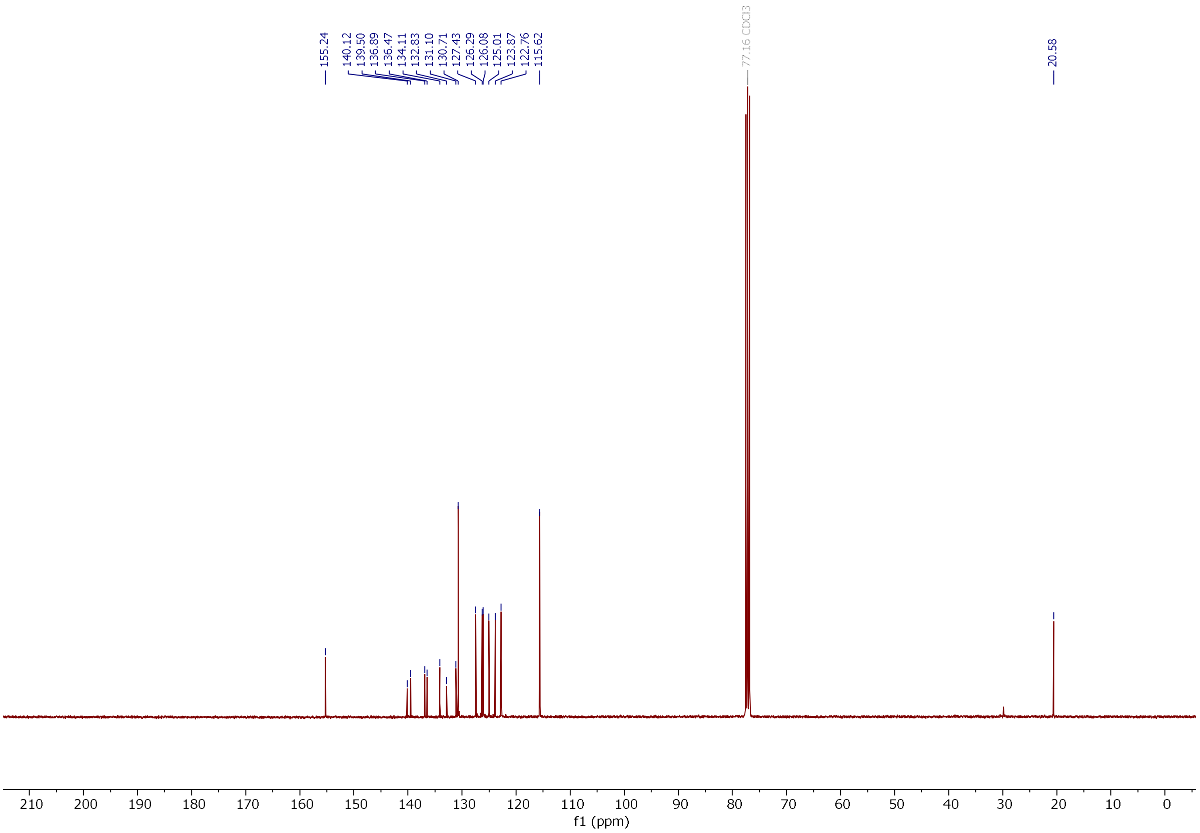

4ap ^{1}H NMR (400 MHz, $CDCl_3$)

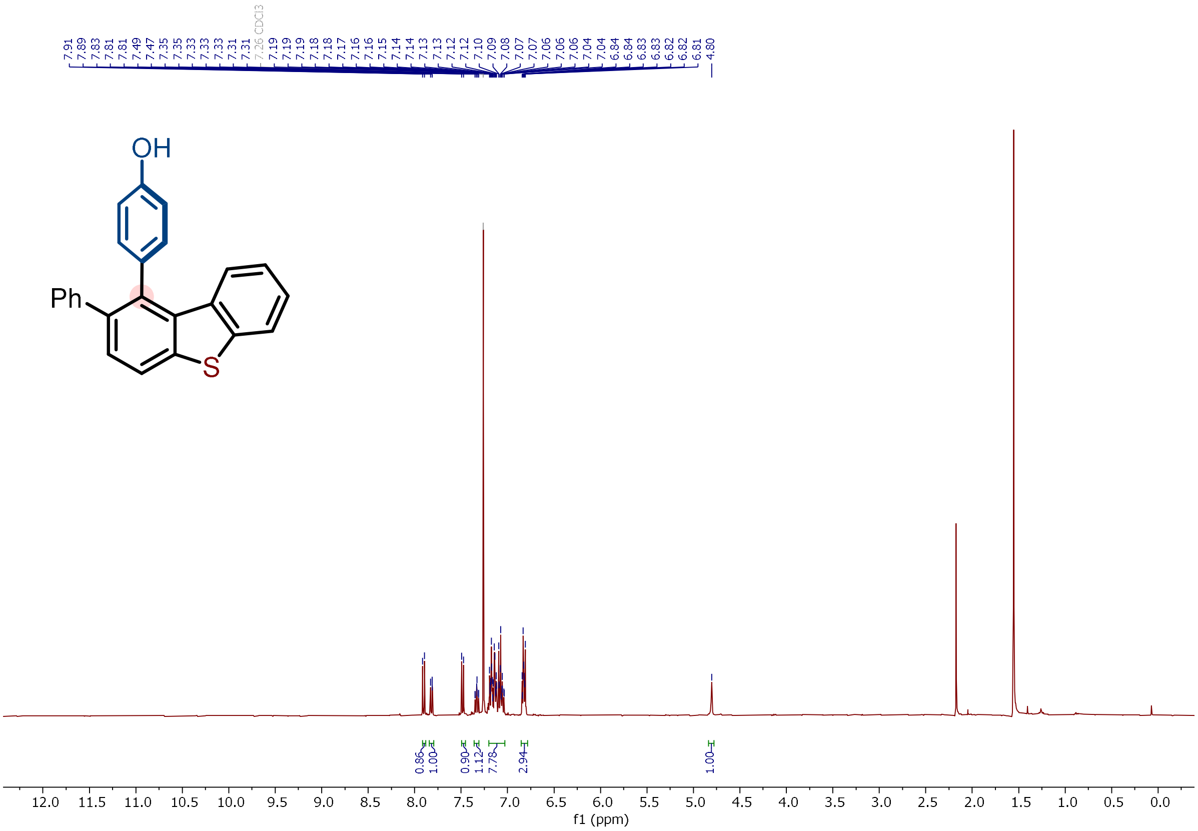

4an ^{13}C NMR (126 MHz, Acetone-d_6)

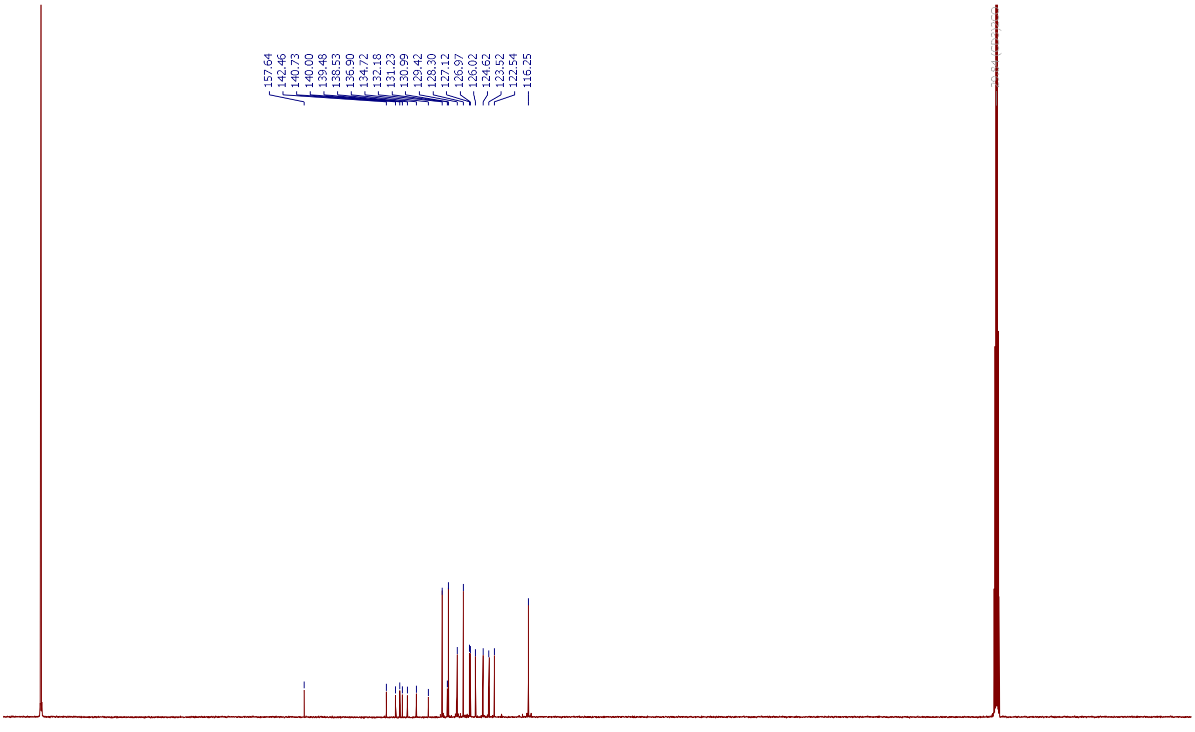

4aq ^{1}H NMR (400 MHz, $CDCl_3$)

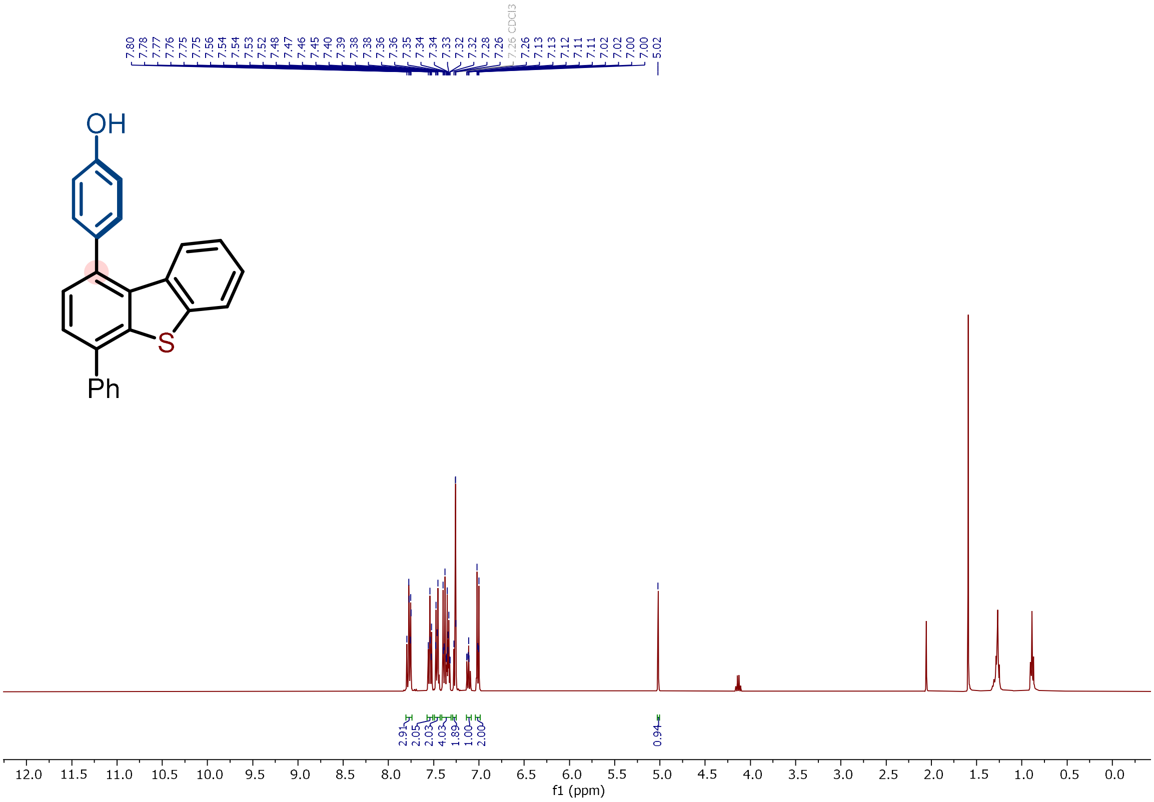

4aq ^{13}C NMR (126 MHz, Acetone-d_6)

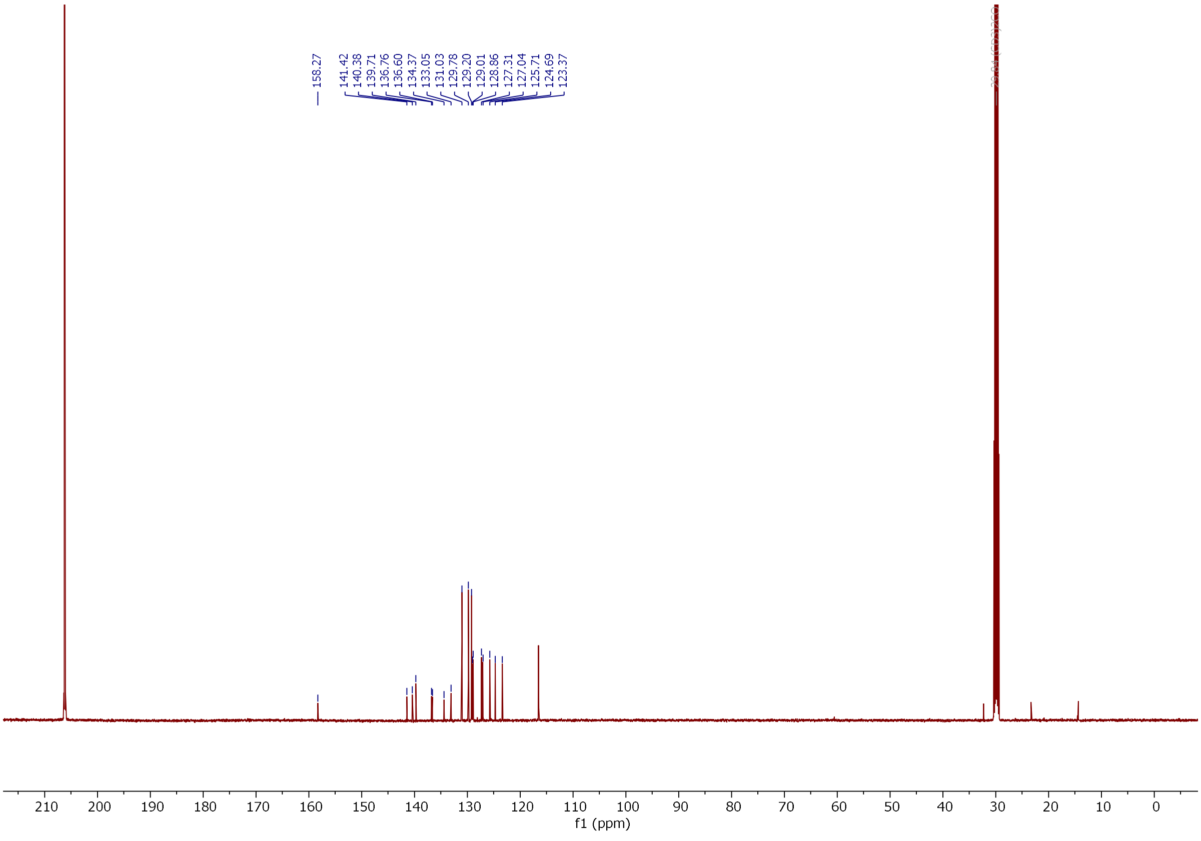

5 ^{1}H NMR (400 MHz, $CDCl_3$)

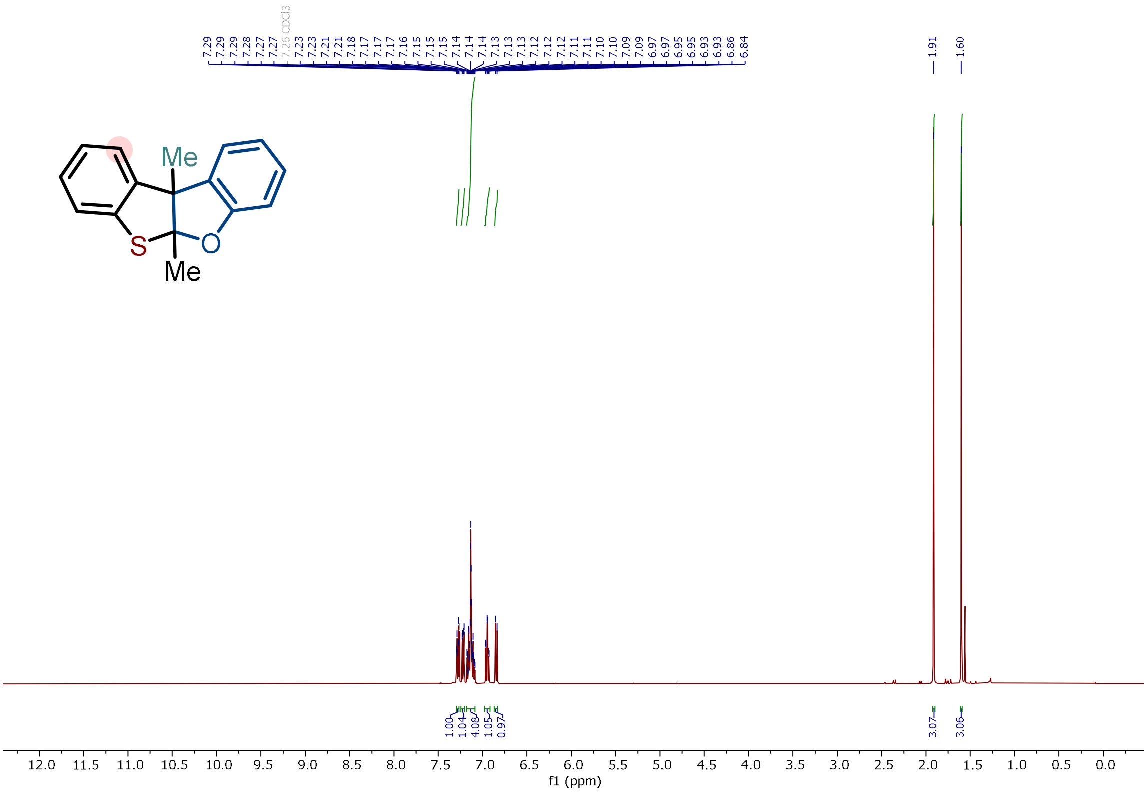

5 ^{13}C NMR (101 MHz, $CDCl_3$)

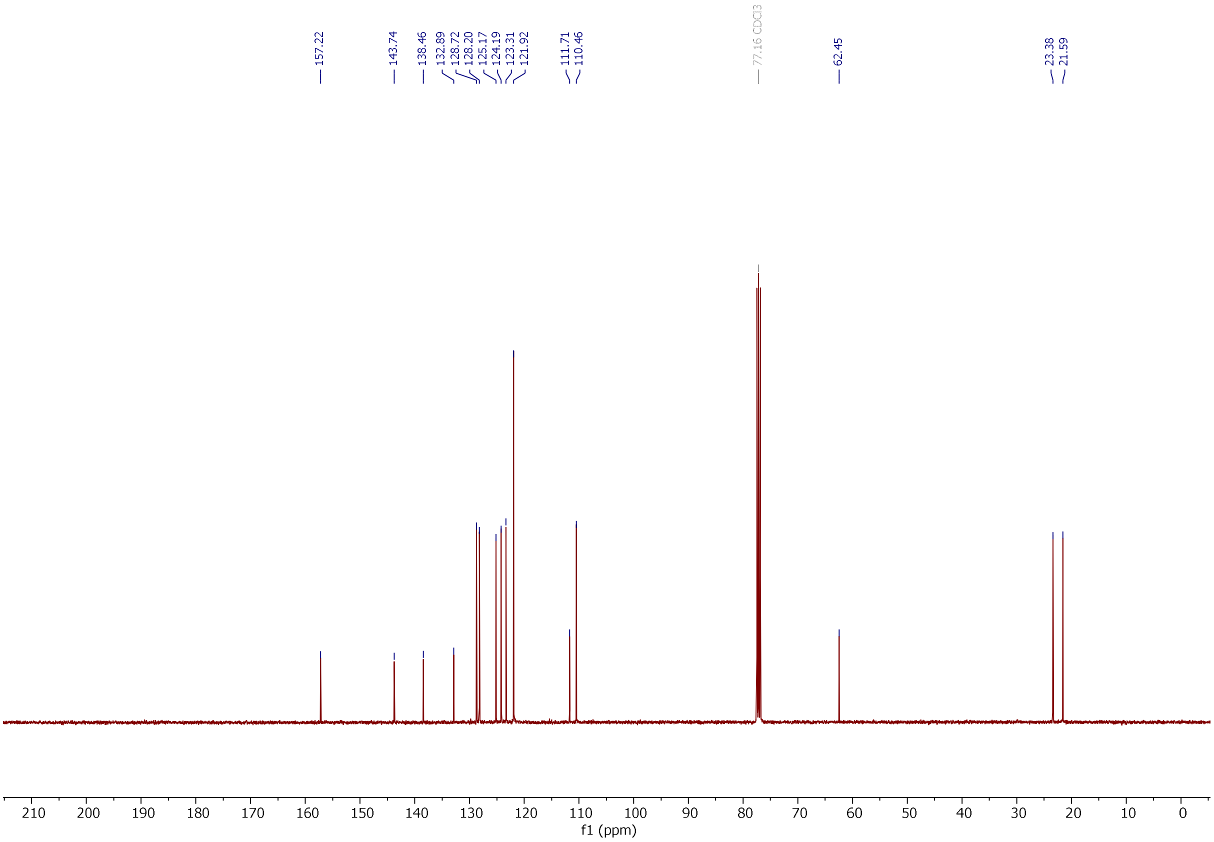

5a ^{1}H NMR (400 MHz, $CDCl_3$)

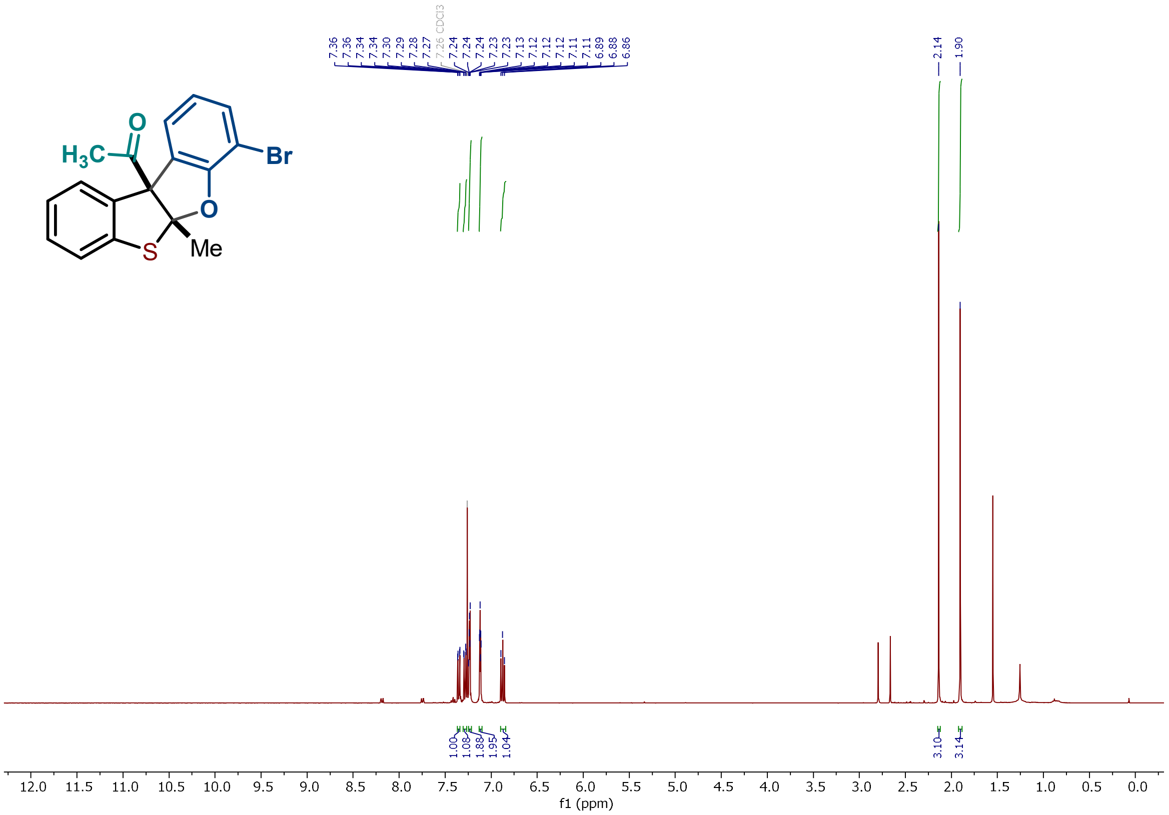

5a ^{13}C NMR (101 MHz, $CDCl_3$)

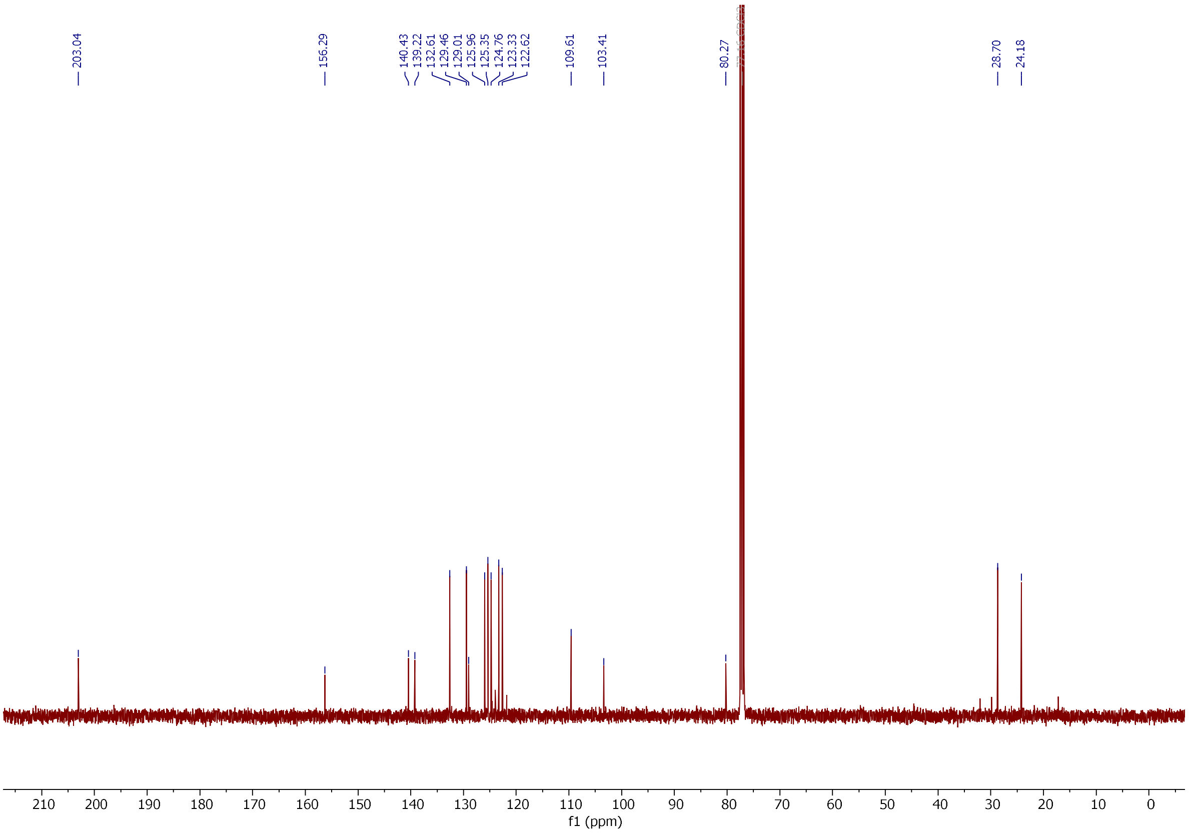

S1 ^{1}H NMR (400 MHz, $CDCl_3$)

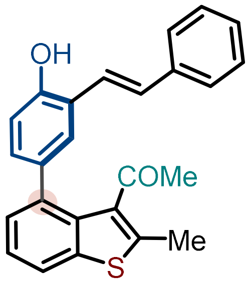

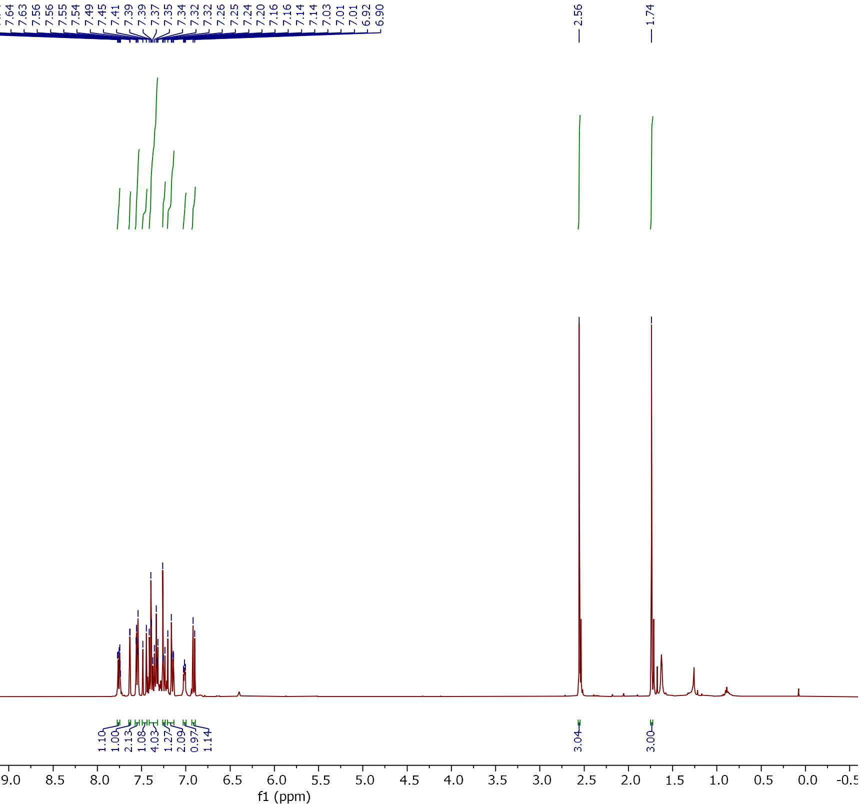

S1 ^{13}C NMR (101 MHz, $CDCl_3$)

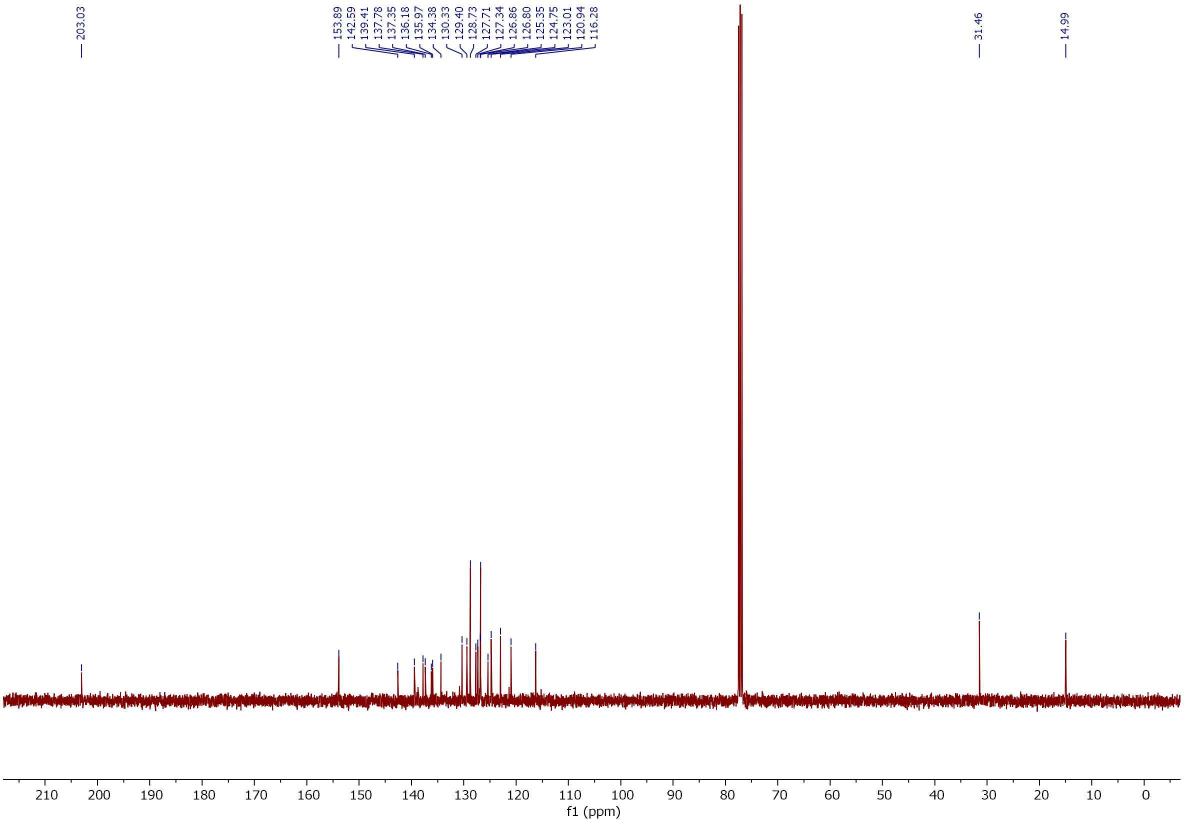

6 ^{1}H NMR (400 MHz, Acetone-d$_6$)

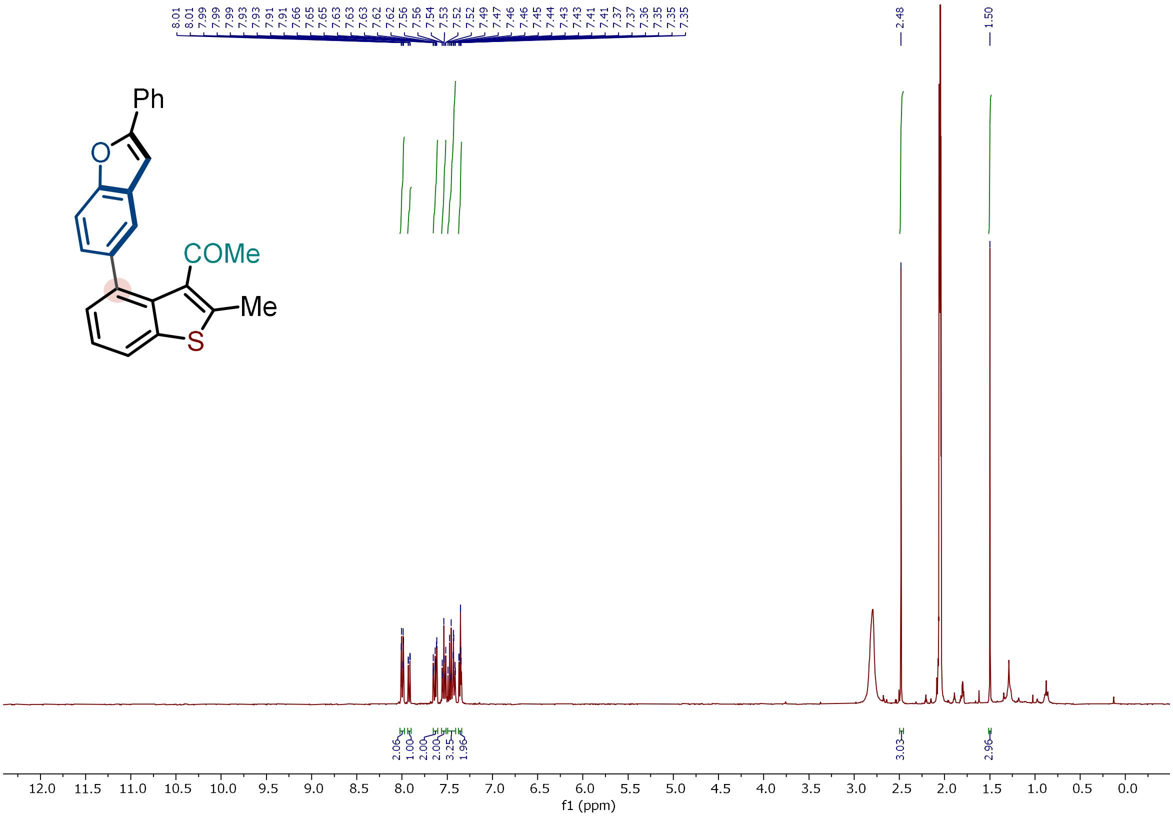

6 ^{13}C NMR (101 MHz, Acetone-d$_6$)

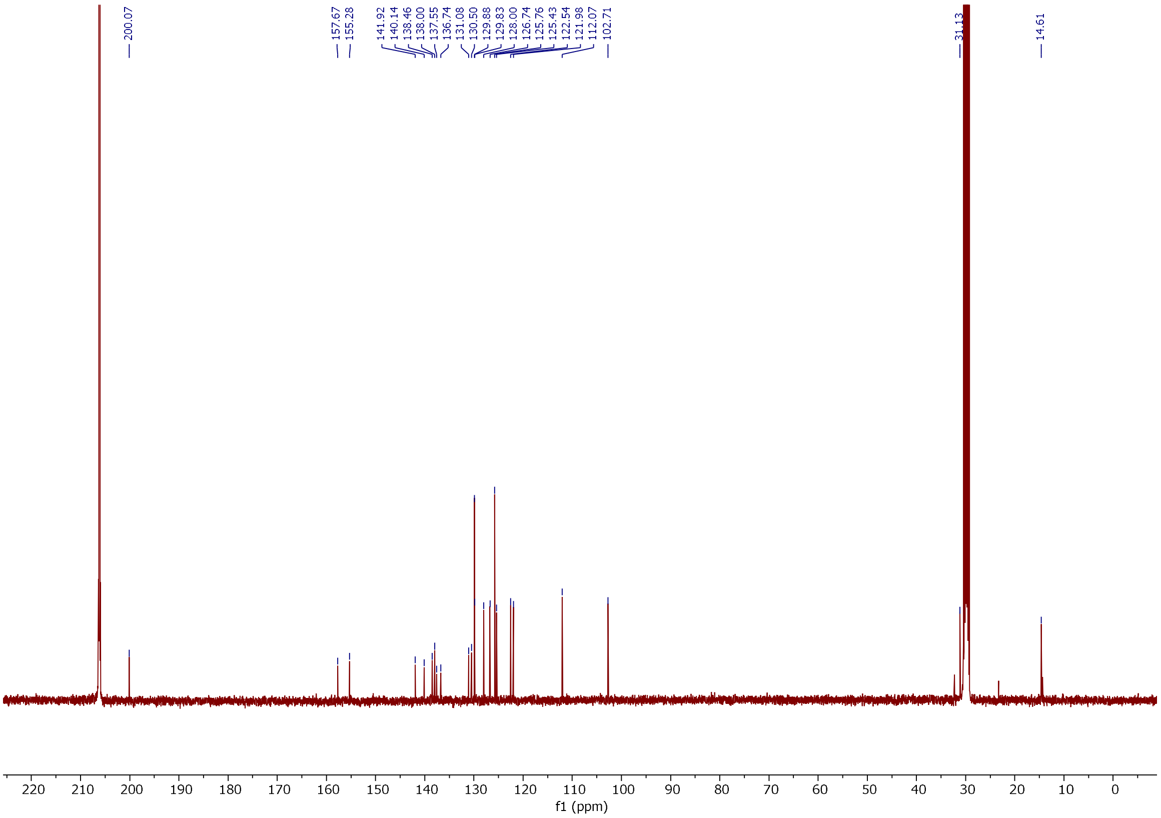

S2 ^{1}H NMR (400 MHz, Acetone-d_6)

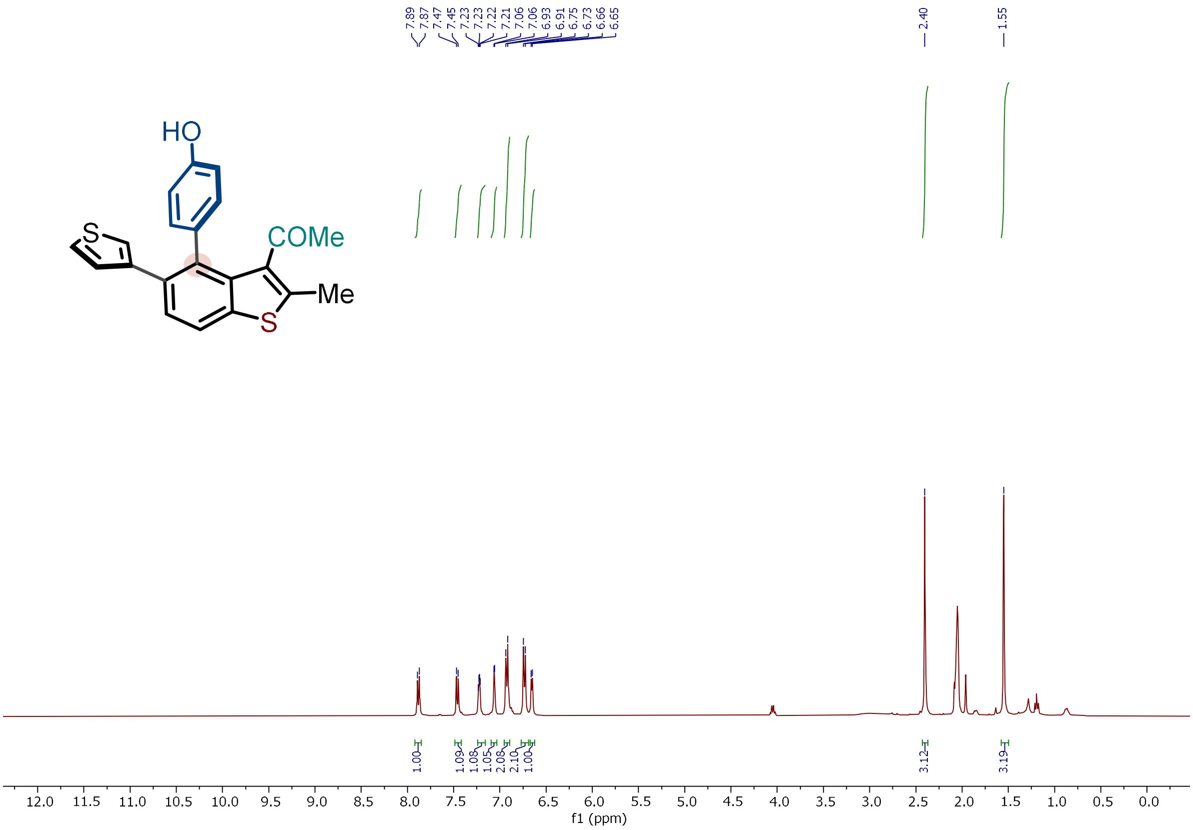

S2 ^{13}C NMR (101 MHz, Acetone-d_6)

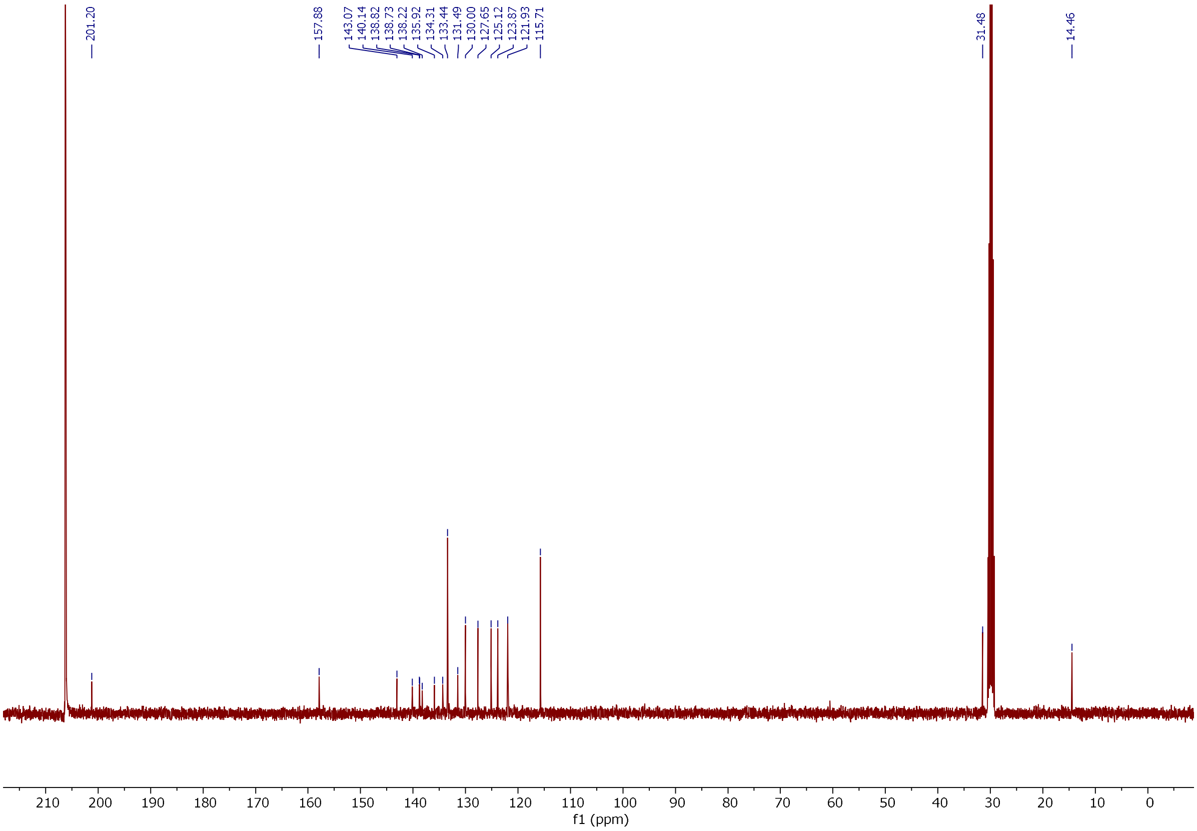

7 ^{1}H NMR (400 MHz, DMSO-d$_6$)

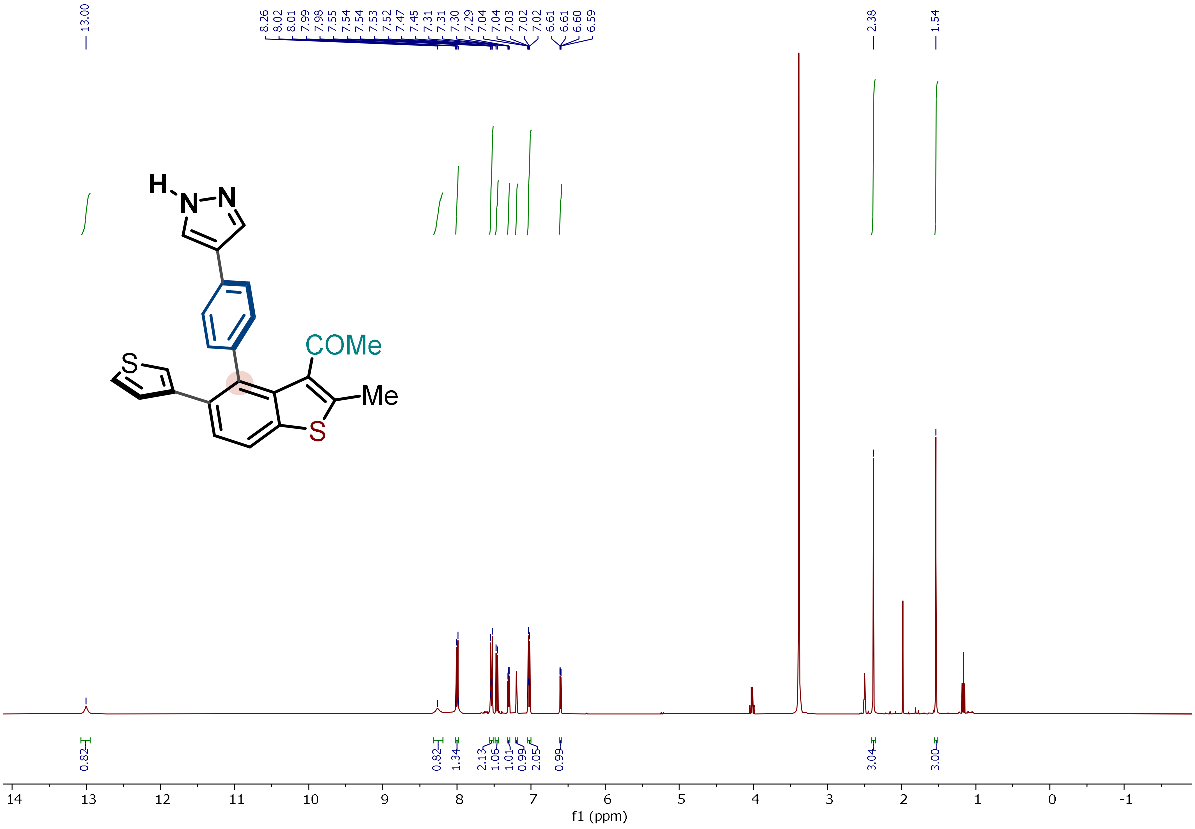

7 ^{13}C NMR (101 MHz, DMSO-d$_6$)

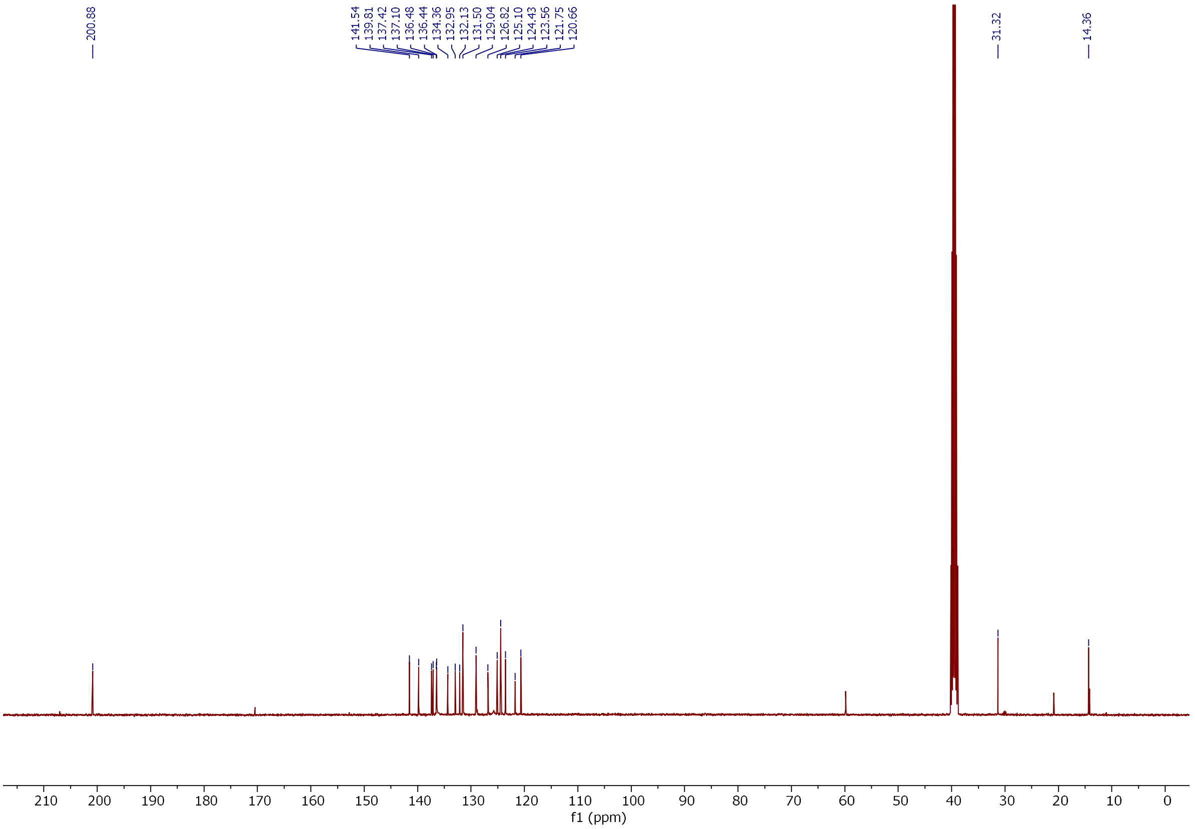

8 ^{1}H NMR (400 MHz, $CDCl_3$)

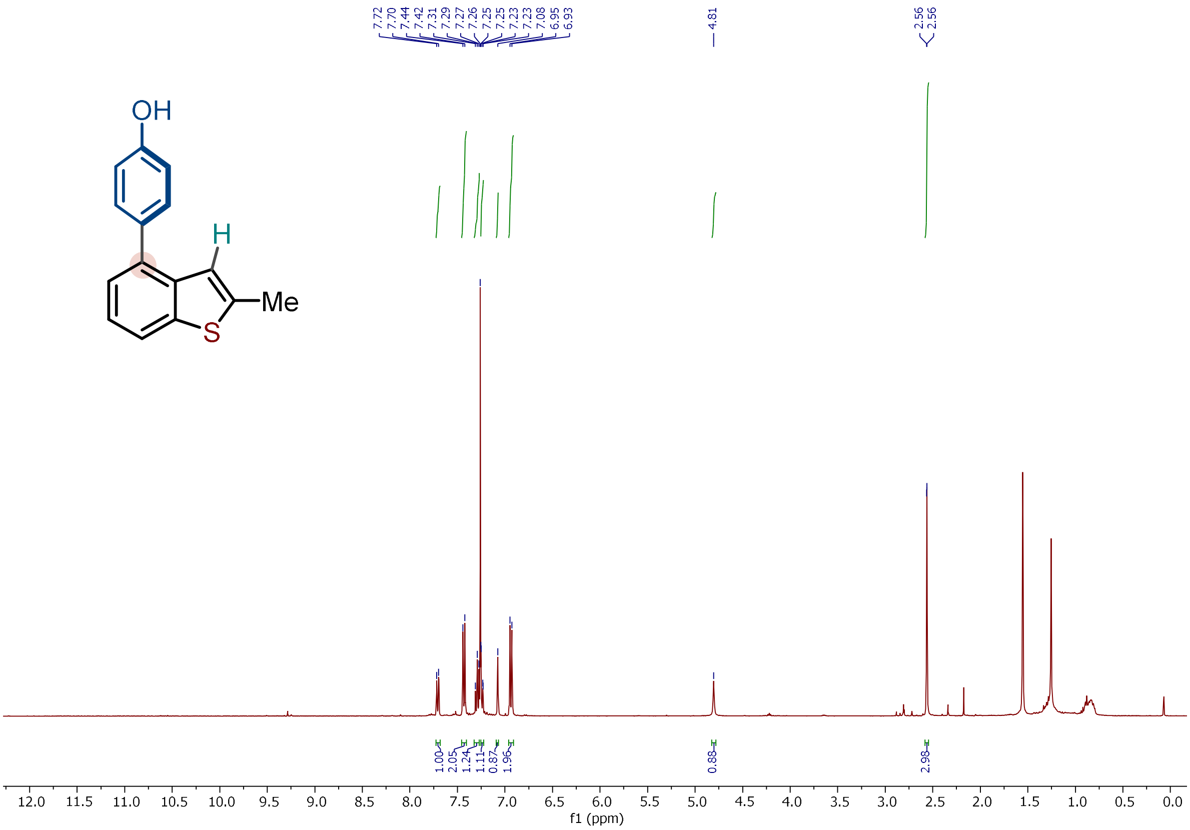

8 ^{13}C NMR (101 MHz, $CDCl_3$)

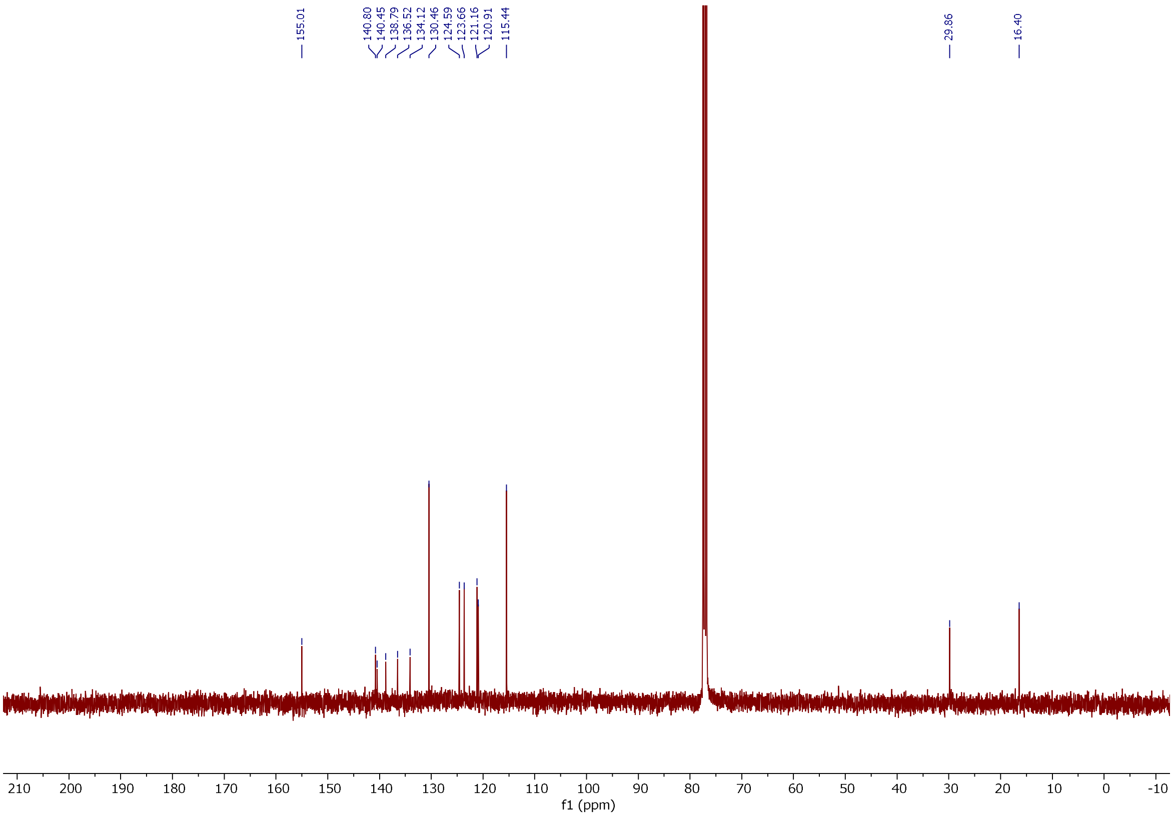

9 ^{1}H NMR (400 MHz, $CDCl_3$)

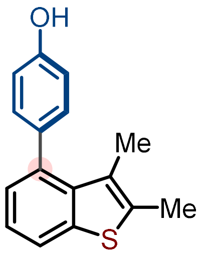

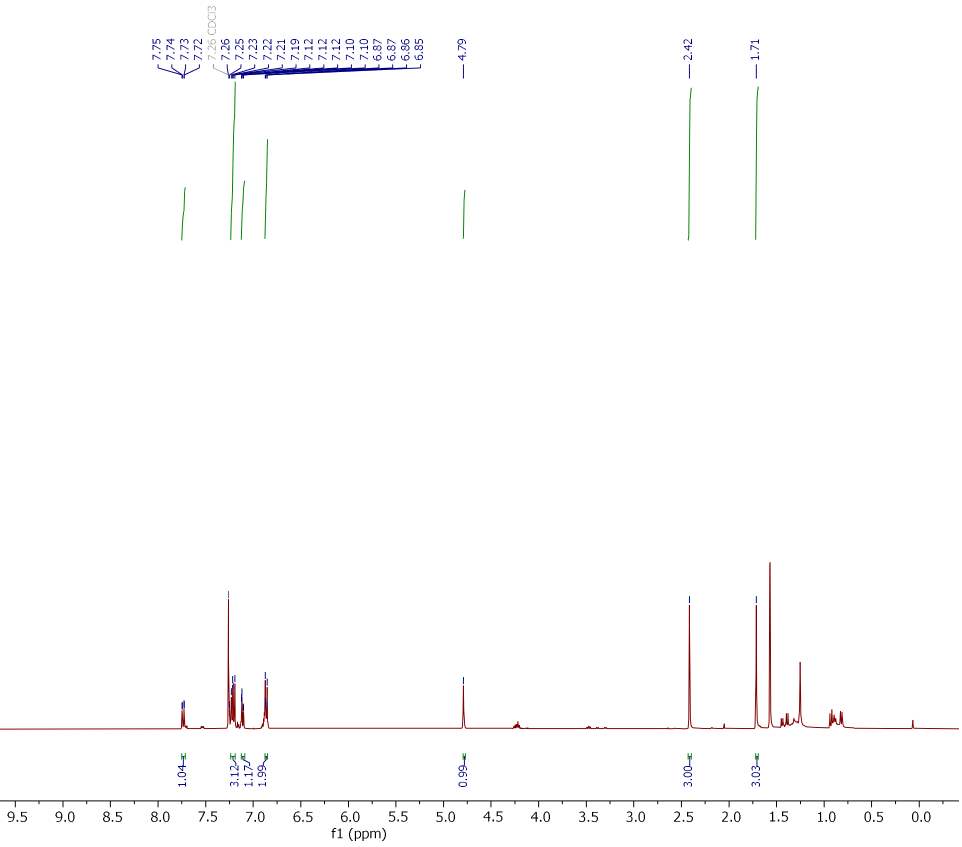

9 ^{13}C NMR (101 MHz, $CDCl_3$)

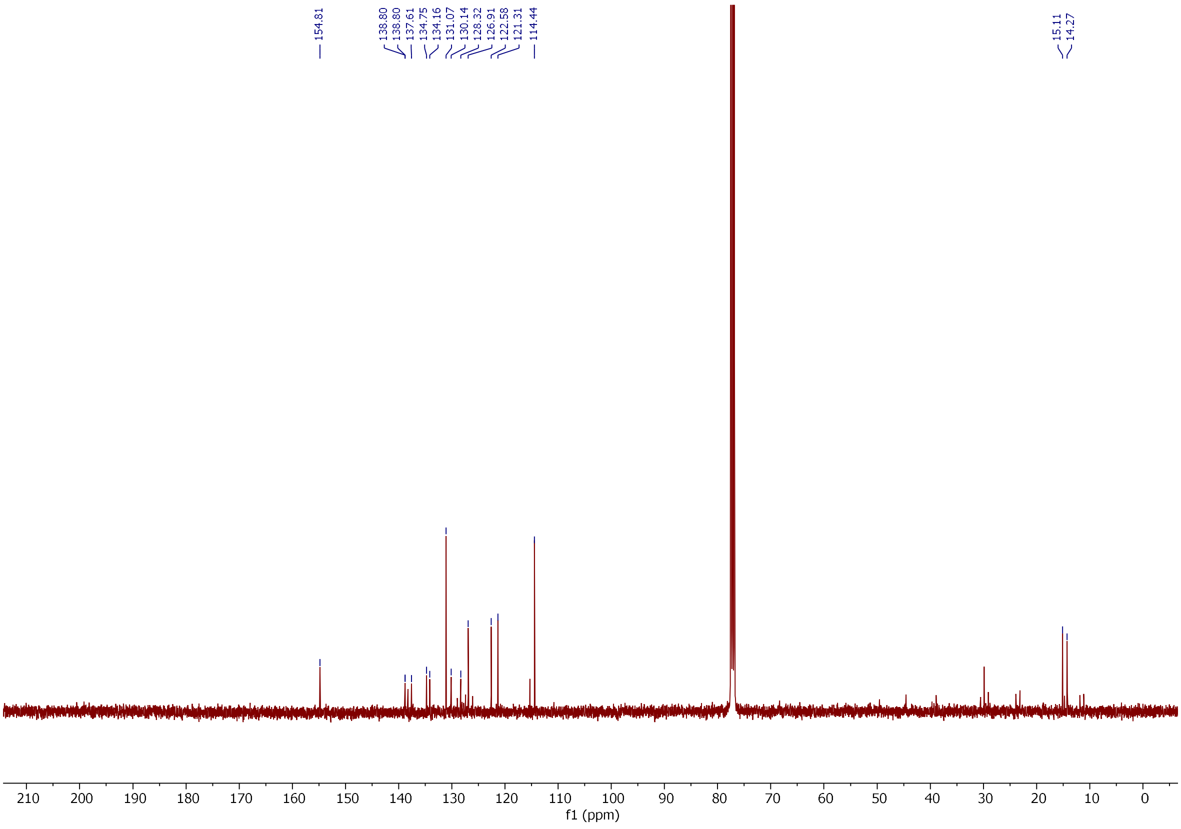

10 ^{1}H NMR (400 MHz, DMSO-d$_6$)

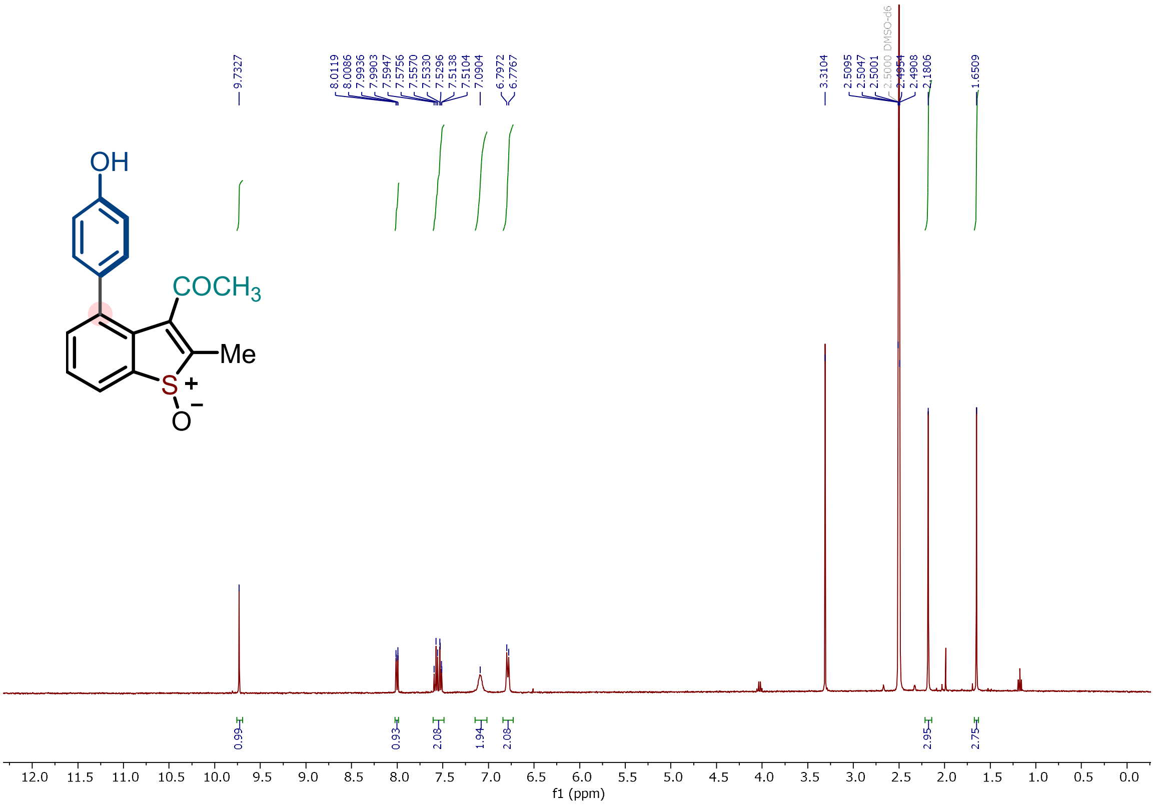

10 ^{13}C NMR (101 MHz, DMSO-d$_6$)

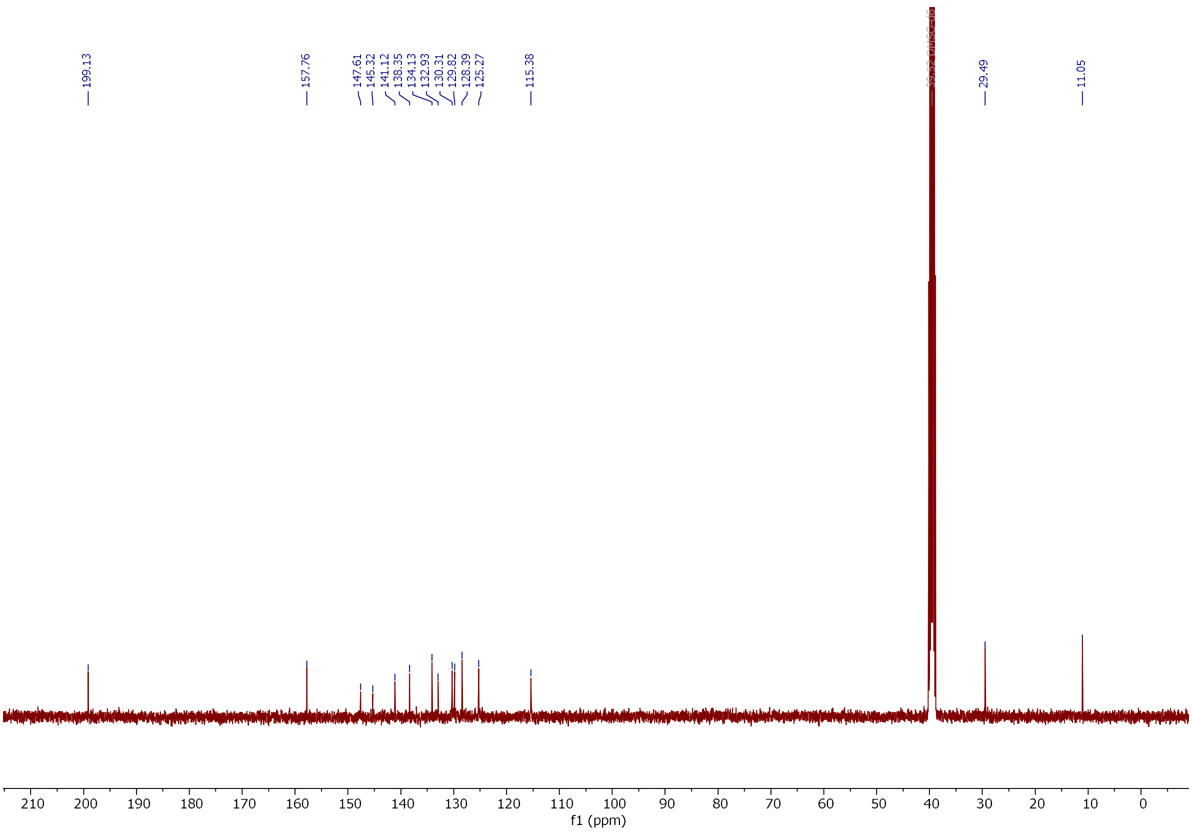

11 ^{1}H NMR (400 MHz, $CDCl_3$)

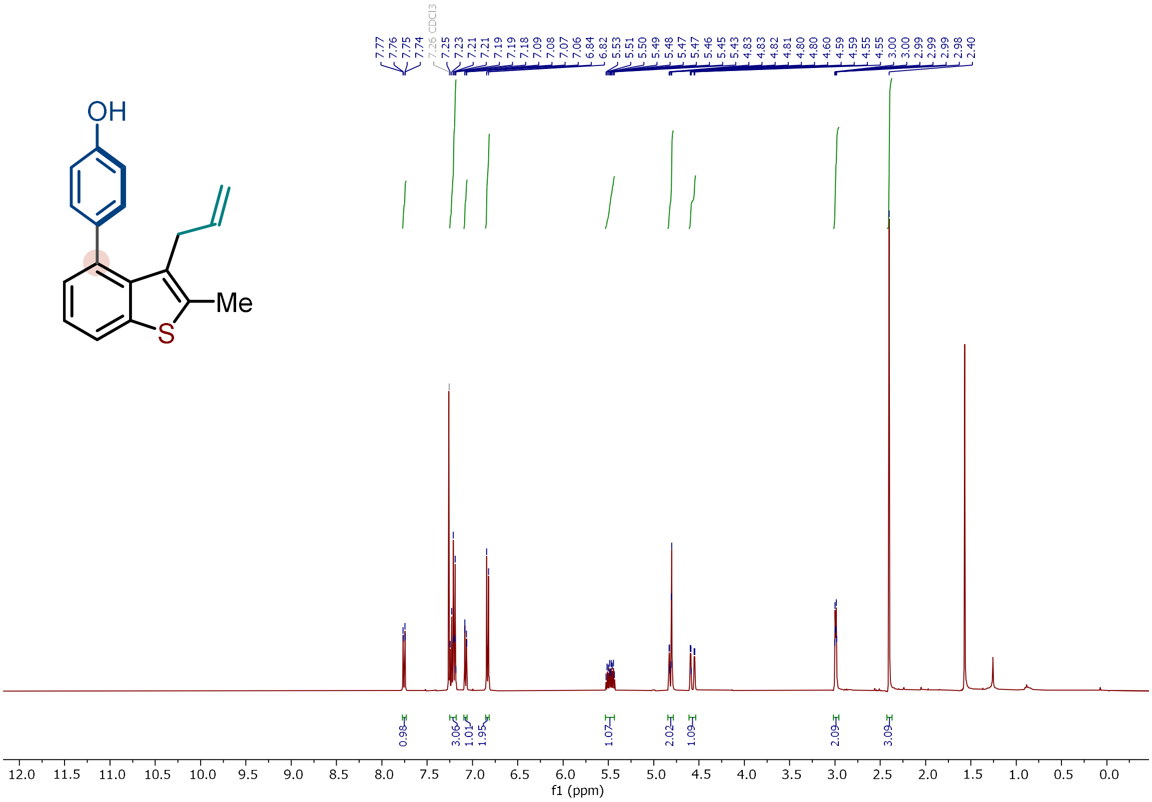

11 ^{13}C NMR (101 MHz, $CDCl_3$)

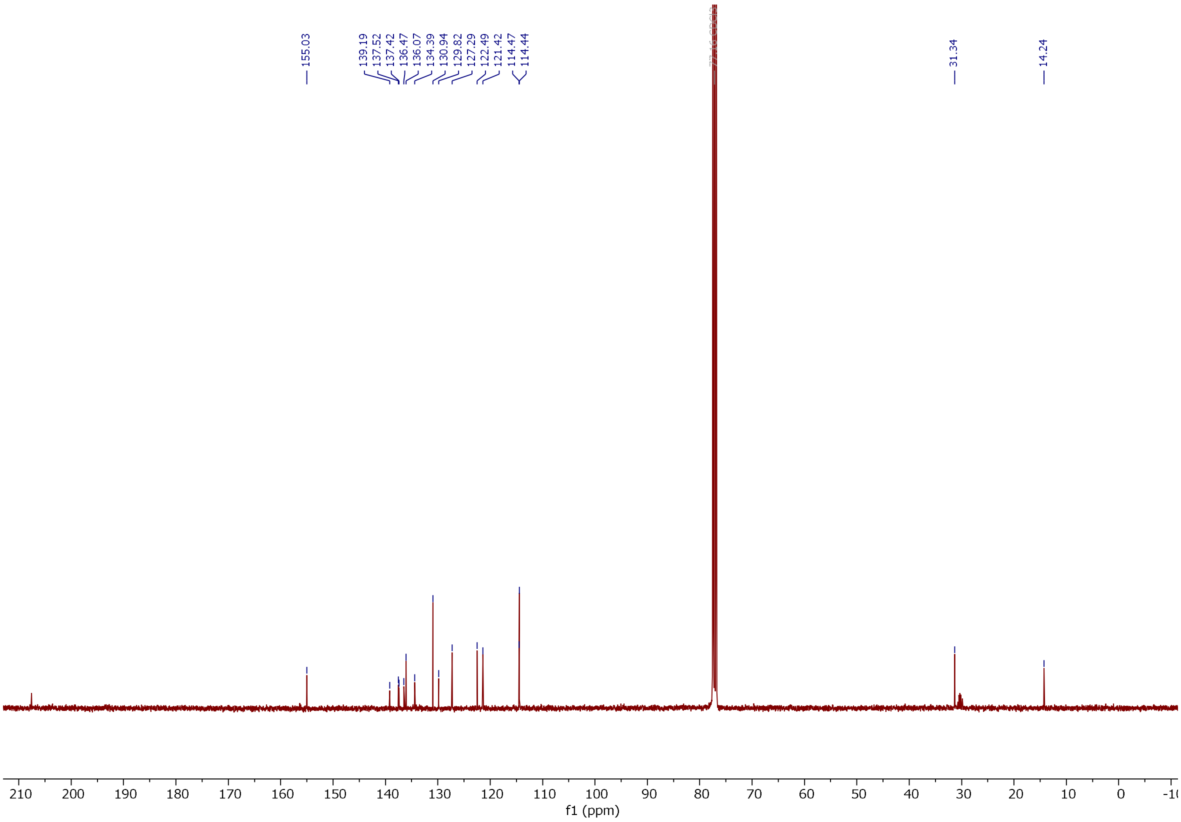

Supplement: Supplementary file 1 — Supporting Information [file ANIE-62-0-s001.pdf]
